# Stereoselective β-mannosylations and β-rhamnosylations from glycosyl hemiacetals mediated by lithium iodide†

**DOI:** 10.1039/d1sc01300a

**Published:** 2021-07-05

**Authors:** Imlirenla Pongener, Dionissia A. Pepe, Joseph J. Ruddy, Eoghan M. McGarrigle

**Affiliations:** Centre for Synthesis & Chemical Biology, UCD School of Chemistry, University College Dublin Belfield Dublin 4 Ireland eoghan.mcgarrigle@ucd.ie

## Abstract

Stereoselective β-mannosylation is one of the most challenging problems in the synthesis of oligosaccharides. Herein, a highly selective synthesis of β-mannosides and β-rhamnosides from glycosyl hemi-acetals is reported, following a one-pot chlorination, iodination, glycosylation sequence employing cheap oxalyl chloride, phosphine oxide and LiI. The present protocol works excellently with a wide range of glycosyl acceptors and with armed glycosyl donors. The method doesn't require conformationally restricted donors or directing groups; it is proposed that the high β-selectivities observed are achieved *via* an S_N_2-type reaction of α-glycosyl iodide promoted by lithium iodide.

## Abstract 2

Breaking from the current paradigm, a highly selective synthesis of hard-to-make β-mannosides and β-rhamnosides from simple glycosyl hemi-acetals has been achieved without using conformational restrictions.

## Graphical abstract

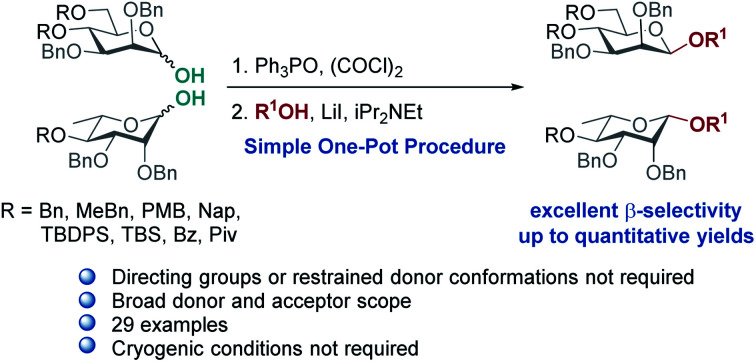

## Introduction

**Scheme 1:**
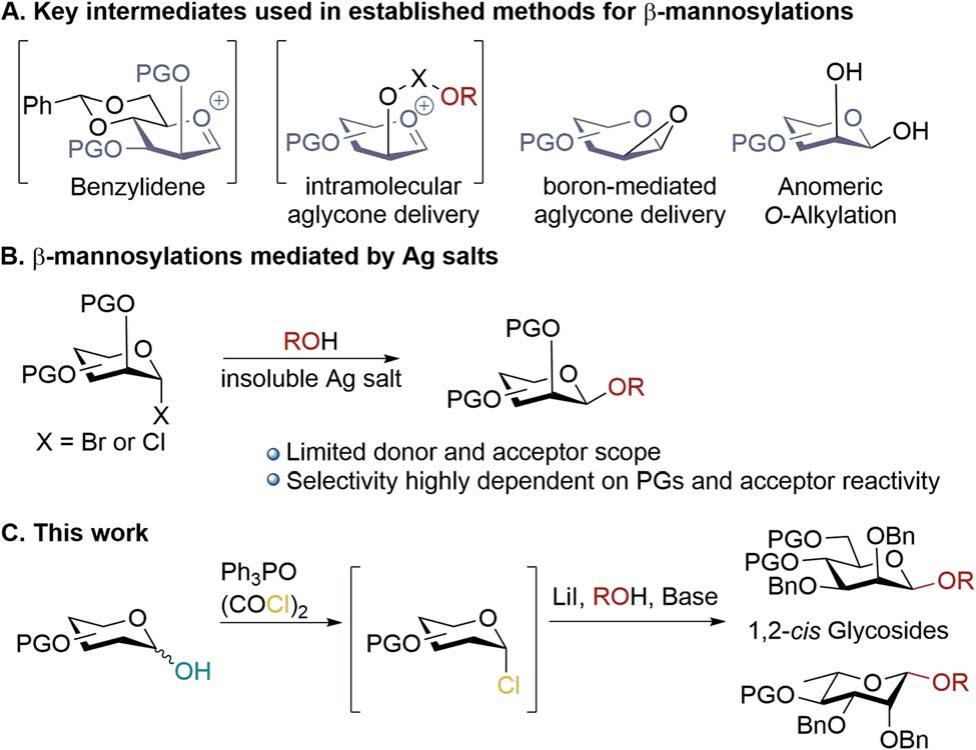
(A) Key intermediates used in established methods for accessing β-mannosides; (B) mannosylations mediated by insoluble silver salts; (C) β-mannosylations and rhamnosylations in this work.

Carbohydrates are ubiquitous in nature; the heterogeneity and complexity of biological glycosylation means that chemical synthesis is required to access pure oligosaccharides for study. A key challenge in the synthesis of oligosaccharides is the creation of 1,2-*cis*-linkages, and of these, β-mannosides and β-rhamnosides are particularly important but difficult to make.^1,2^ Biologically important examples include high mannose-type *N*-linked glycans,^3^ and pathogenic bacteria.^4–8^ Mannosylations and rhamnosylations tend to show a high preference for formation of α-glycosides unless special measures are taken, as both the anomeric effect and neighbouring group participation favour α-linkages, and approach to the β-face induces steric clashes with the 2-*O*-substituent.^9,10^ Established methodologies for achieving stereoselective β-mannosylations^11,12^ rely on: (a) conformational control^13–15^ (b) intramolecular aglycone delivery^16–23^ (c) use of directing groups^22^ and (d) anomeric *O*-alkylations (Scheme 1A).^24,25^ A general drawback of most of these approaches is the need for donors bearing specific protecting group patterns (often requiring long synthetic sequences), which can be restrictive in designing multi-step syntheses of complex molecules. Koenigs–Knorr glycosylations employing mannosyl halides using insoluble silver salts is not in widespread use, possibly because of the outcome being quite dependent on the donor/acceptor identities (Scheme 1B).^1,26^ The prevailing paradigm seems to be that in order to achieve high β-selectivity in mannosylation it is necessary to use protecting groups that enforce conformational control, or to use a directing group/intramolecular approach. Herein, we demonstrate proof-of-principle that β-mannosylations can be achieved without relying on specific protecting group patterns to enforce conformational control or directing group effects. A simple, practical, method for β-mannosylation and β-rhamnosylation with broad scope is reported.

## Results and discussion

Previously, we reported the transformation of glycosyl hemiacetals to chlorides using catalytic Appel conditions.^27–29^ Our efforts to intercept the postulated alkoxyphosphonium intermediate^30–37^ with an acceptor in direct glycosylations were unsuccessful (a one-pot protocol employing a urea catalyst^38–41^ was successful). We hypothesised that if glycosyl iodides could be generated *in situ* from hemiacetals using a modification of the catalytic Appel conditions, then a one-pot glycosylation could be developed. During these investigations we made a serendipitous discovery of conditions that led to highly selective β-mannosylation and β-rhamnosylation using simple donors (Scheme 1C).

Based on procedures by Denton^28^ and Konzer,^42^ we chose LiI in CHCl_3_ for halide metathesis.^43^ We had previously explored various solvents for the synthesis of glycosyl chlorides from glycosyl hemiacetals and found CHCl_3_ and CH_2_Cl_2_ to be the best but CHCl_3_ was found to be superior for the following reactions.^27^ We found that a one-pot, two-stage process generated mannoside **2a** in high yield and β-selectivity (Table 1). Thus, glycosyl chloride was generated using Ph_3_PO/(COCl)_2_ (Scheme 1C), and following removal of (COCl)_2_, LiI, base and alcohol were added. Following some exploration, we settled on the reaction conditions shown in Table 1 and tested the generality of the procedure for mannosyl hemi-acetal donors. Excellent selectivity and yields were obtained with armed mannose donors **1a,d–f** bearing orthogonal ether and silyl ether protecting groups. Donors **1b** and **1c**, bearing the 4-methylbenzyl group (MeBn) proved to be excellent substrates. The MeBn group has been reported to be orthogonal to Bn, Bz, and PMB groups^44^ and thus donor **1c** could be an efficient building block in 1,4-β-mannan synthesis. Donors **1g,h** gave only moderate yields, although high to excellent selectivity was still obtained. In the case of benzoyl-protected donor **1h**, the yield was lowered due to formation of elimination product, competing transesterification under glycosylation conditions and subsequent intramolecular reaction to give an anhydro sugar. Disarmed donors *e.g.*, peracetylated mannose showed poor reactivity and gave complex mixtures under our conditions, elimination to give glycals and migration of acetyl groups to acceptor **3a** were noted (see limitations section in ESI†). However, pivaloyl-protected donor **1i** gave excellent yield and no transesterification was observed. We demonstrated the method on 1 mmol scale for the synthesis of **2a**, which was successful at 30 °C (on this scale at 45 °C the α : β ratio was 10 : 90).

**Table 1:** Donor scope for β-mannosylations (isolated yield and α/β ratio)

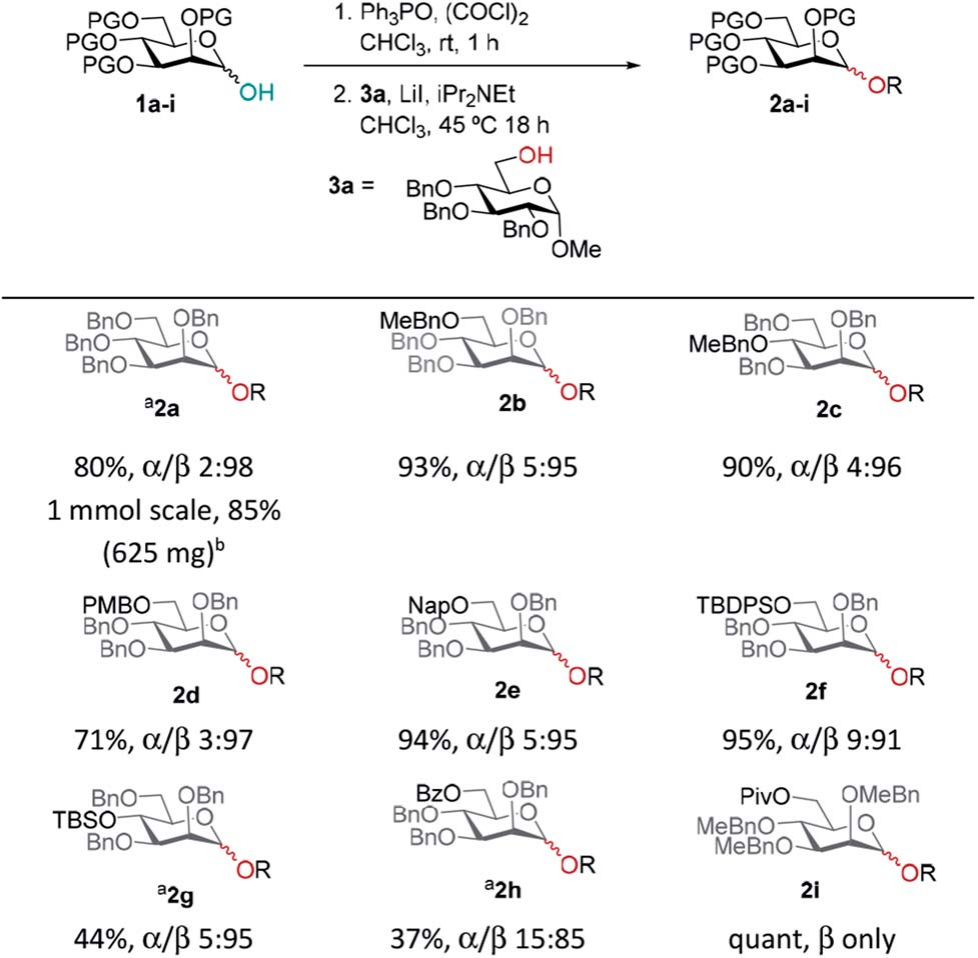

A range of acceptors were also tested to establish the scope of the method (Table 2). For secondary alcohols, a larger excess of iPr_2_NEt was found to give better conversion to the desired glycoside (more unreacted acceptor and elimination product resulted with lower amounts of base). High β-selectivity was obtained with a wide range of glycosyl acceptors. Monosaccharide acceptors bearing primary and secondary alcohols at positions 3 and 4 were tolerated. In the case of benzylidene-protected acceptor **3d**, some cleavage of the acetal was also observed which lowered the yield of **4d**. The excellent selectivity with glucosamine derivative **3h** enables the synthesis of a β-d-Man(1→4)-GlcN linkage. The lower yield in this case reflects the poor nucleophilicity of this acceptor and thus the elimination reaction on the donor competes. In addition, phenolic acceptors **3e–g** gave excellent selectivities and yields of the *O*-glycoside. 4-Nitrophenol β-mannopyranoside **4g**, which is of interest for enzymatic studies was synthesised in excellent yield and selectivity.^45^ A limitation was that ester-substituted acceptors either gave no reaction or gave complex mixtures; we believe the latter result arises, at least in part, from transesterification and thus multiple acceptors being present in solution. We confirmed that ester migration occurred on a benzoylated acceptor with iPr_2_NEt in CHCl_3_ at 45 °C in the absence of other reagents (see limitations section in ESI†).

**Table 2:** Acceptor scope for β-mannosylations (isolated yield and α/β ratio)

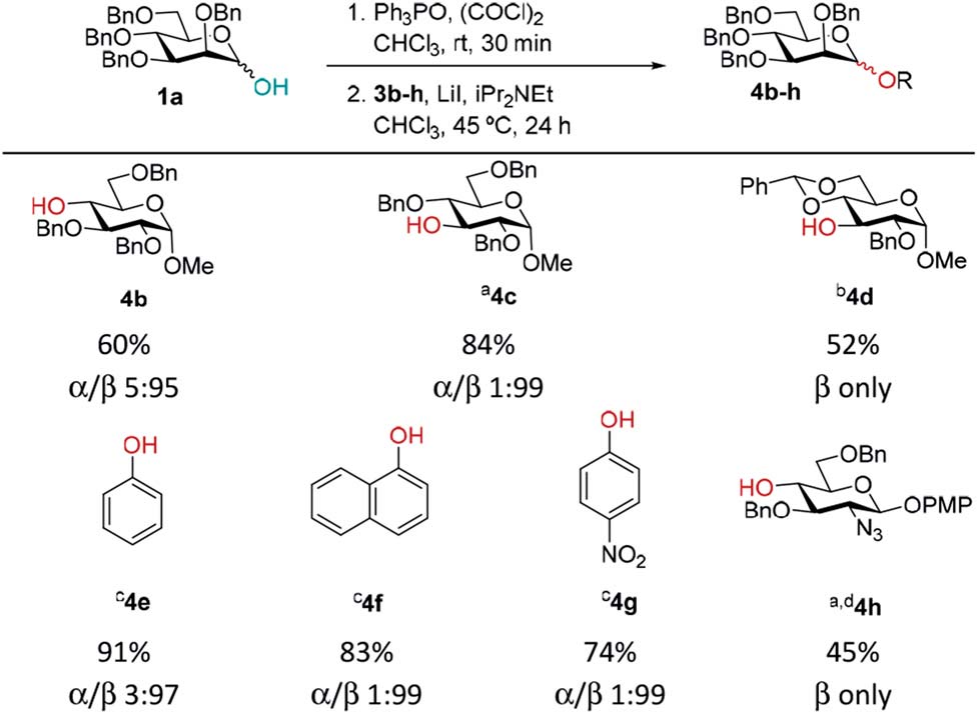

### Rhamnosylations

Delighted with the excellent results for β-mannosylations, we explored the reaction conditions for rhamnosylations (Table 3). Approaches to stereoselective β-rhamnosylations include use of an electron-withdrawing group on the 2-*O*-position,^46^ use of 2,3-*O*- and 3,4-*O*-tethered rhamnosyl donors.^47–49^

**Table 3:** Donor and acceptor scope for β-rhamnosylations (isolated yield and α/β ratio)

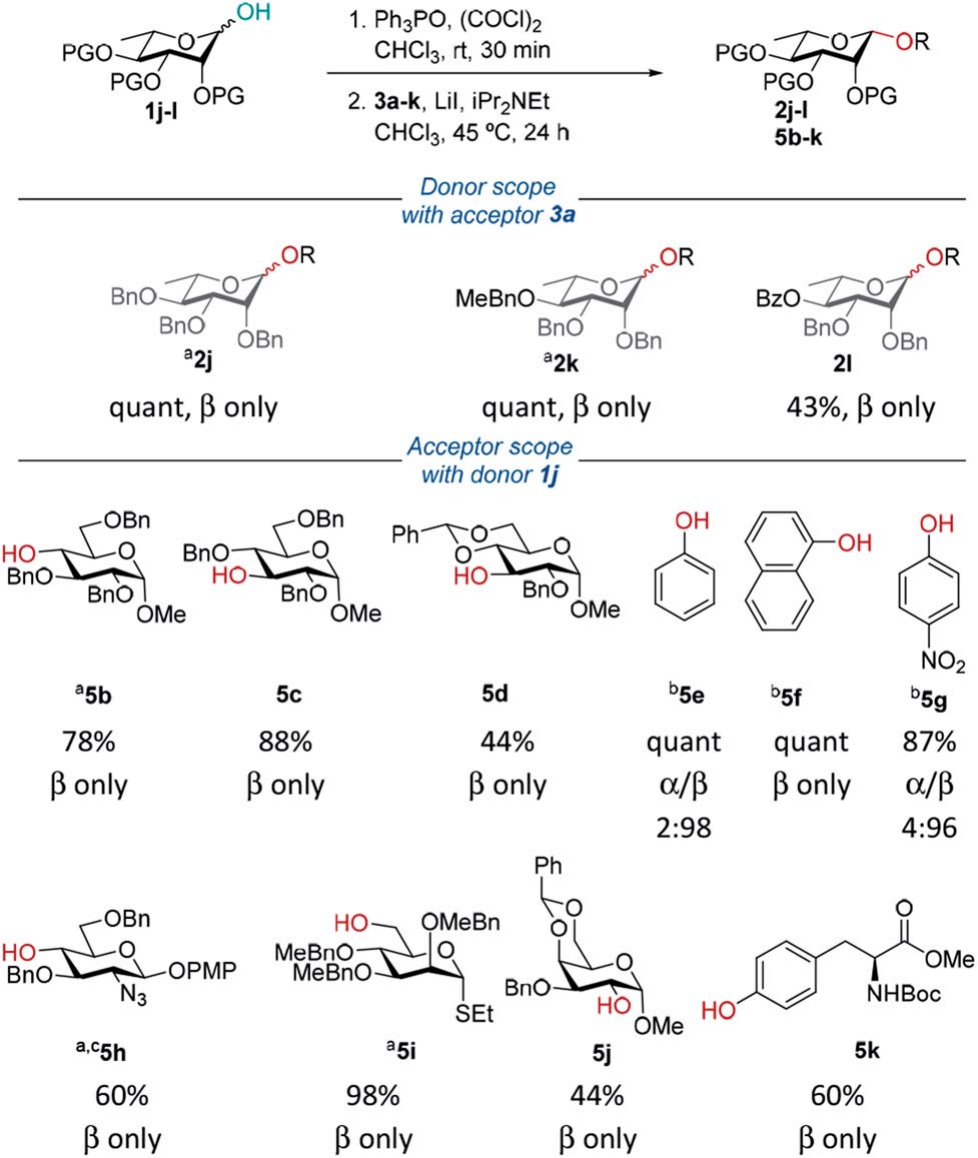

Recently, Bols and Pedersen showed that bulky silyl-protected rhamnosyl donors gave β-selectivity as they adopt an axially-rich conformation.^50^ Rhamnose donors **1j,k** gave excellent yields and selectivities. In the case of benzoyl-protected donor **1l**, excellent selectivity was achieved however the yield was only moderate due to transesterification. Excellent β-selectivity was obtained with a wide range of glycosyl acceptors, except for cholesterol (see ESI†). Thioglycosides, and acceptors bearing azido and NHBoc groups were tolerated, which could be helpful in complex molecule synthesis.

From a practical perspective, we believe the method is attractive as it doesn't require protecting groups that impose conformational restraints on the donor. All the reagents are commercially available and the hemi-acetal donors are straightforward to synthesize and are bench stable.^51^ The reaction is straight-forward to implement and cryogenic conditions are not required. Semi-orthogonal protecting group patterns and tolerance of thioglycosides should enable its use in oligosaccharide synthesis. Nonetheless, the method is not without limitations, *e.g.*, the poorer results with acyl and benzoyl ester protecting groups are a drawback (see ESI†).

### Preliminary mechanistic investigations

**Scheme 2:**
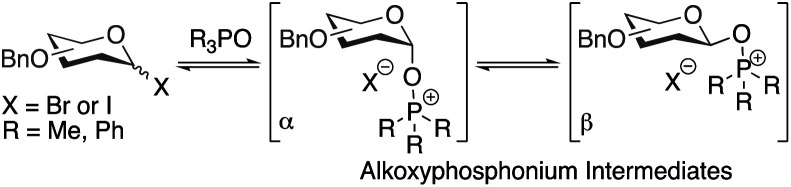
Alkoxyphosphonium intermediates previously invoked in rationalising outcomes of glycosylations.

The high selectivities observed in these glycosylations are notable and some preliminary mechanistic investigations are described here (Table 4). Previously, it has been suggested that α/β-mixtures of glycosyl bromides and iodides are equilibrated by phosphine oxides *via* alkoxyphosphonium intermediates and that α-selective glycosylations proceed through a β-alkoxyphosphonium salt (Scheme 2).^36–43^

**Table 4:** Experiments to probe mechanism

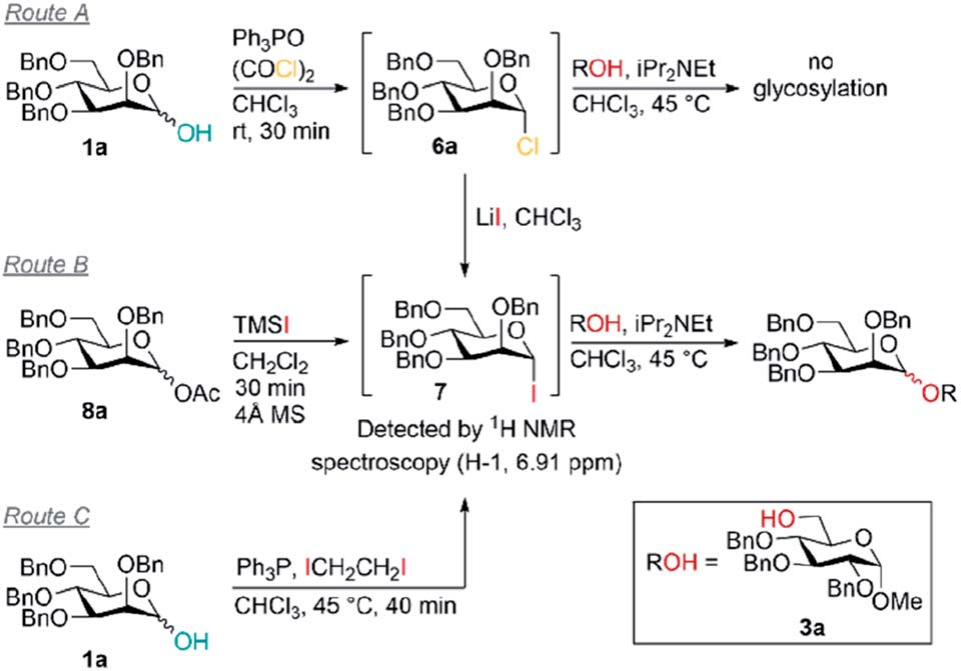			
Entry	Route	Deviation from standard procedure	β/α^a^
1	A	No Ph_3_PO^b^	≥20 : 1
2	A	0.5 eq. Ph_3_PO	≥20 : 1
3	A	No LiI	No glycosylation
4	A	NaI *in lieu* of LiI	1 : 1
5	A	Filtration of insoluble LiCl/LiI salts	1 : 1
6	A	Filtration of insoluble LiCl/LiI salts; no Ph_3_PO^b^	≥20 : 1
7	A	2 eq. LiI *in lieu* of 4 eq. LiI	2 : 3
8	A	2 eq. Ph_3_PO	1 : 1
9	A	2 eq. Ph_3_PO, 8 eq. LiI	10 : 1
10	A	No Ph_3_PO^b^ 4 Å MS	≥20 : 1
11	B	0.5 eq. Ph_3_PO	2 : 3
12	B	4 eq. LiI	3 : 1
13	B	4 eq. LiCl	2 : 3
14	C	—	2 : 3
15	C	4 eq. LiI	≥20 : 1

a Determined by ^1^H NMR spectroscopy of the reaction mixture.

b After synthesis of **6a**, the Ph_3_PO was removed by chromatography.

However, in contrast to our initial hypothesis, and to literature reports invoking these alkoxyphosphonium salts as key intermediates, we did not find that the phosphine oxide is beneficial in the glycosylation step in our system. When glycosyl chloride **6a** was synthesised as normal and then the Ph_3_PO removed prior to glycosylation, excellent β-selectivity was still obtained (entry 1).

Our evidence suggests that glycosyl iodides **7** are key intermediates and that the presence of LiI promotes the stereoselective glycosylation step. In the absence of LiI no glycosylation products were observed (entry 3). With NaI in place of LiI no stereoselectivity was obtained (entry 4) and some unreacted glycosyl chloride was present. In the absence of added alcohol, the α-glycosyl iodide **7** was formed with complete consumption of the chloride **6a**.^52,53^ Thus glycosyl iodides can be generated directly from glycosyl hemiacetals using the combination of Appel conditions and LiI in CHCl_3_ – this approach may be advantageous compared to other methods in many circumstances, especially in terms of practicality.^54–56^

Mannosyl iodides have been reported to react with cyclic ethers and thioethers to give β-selectivity^57–60^ but under *in situ* anomerisation conditions α-mannosides are usually obtained.^61,62^ We note that the β-mannosylations using cyclic ethers were conducted in the presence of MgO and that lithium and magnesium ions have similar ionic radii.^57–60^ The salts LiI, LiCl, NaI and NaCl are insoluble in CHCl_3_. Since glycosylations of mannosyl chlorides and bromides promoted by insoluble silver salts have been reported,^1,2,63^ a filtration of the reaction mixture was carried out prior to addition of alcohol to remove insoluble LiCl and LiI – this did not lead to a change in selectivity when carried out in the absence of Ph_3_PO (entry 5 *vs.* entry 6); it is possible that the excess LiI is important for preventing the erosion of β-selectivity due to the presence of Ph_3_PO (entry 7, 8 *vs.* 9), or that a lithium salt in solution may be activating glycosyl iodides, or that lithium alkoxides are the active nucleophiles. When TMSI was used to generate glycosyl iodide from glycosyl acetate **8a** (route B), the glycosylation was slightly α-selective (entry 11), however, when LiI was added it was moderately β-selective (entry 12); the moderate selectivity might arise because TMSOAc interferes. We confirmed that the presence of 4 Å MS doesn't impact on the selectivity (entry 10). Replacing LiI with LiCl also led to poor selectivity (entry 13). Route C was used to generate the glycosyl iodide through reaction of hemiacetal **1a** with an iodophosphonium salt generated *in situ*.^55,64^ As in route B, the glycosylation was slightly α-selective (entry 14) but exhibited high β-selectivity when LiI was added (entry 15). These preliminary experiments suggest LiI is important for more than one fundamental role in achieving the desired reactivity and β-selectivity during glycosylation. Investigation of the underlying mechanism and the role of LiI is the subject of ongoing work in our laboratory.

**Scheme 3:**
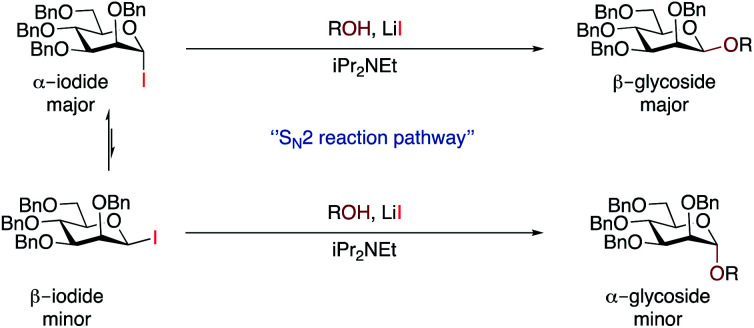
Proposed reaction pathways for glycosylation.

We discount an S_N_1-like mechanism as this would be expected to give α-selectivity. Although we did not observe β-iodide **7**, we cannot rule out the possibility that the α/β-anomers are in rapid equilibrium under reaction conditions.^65,66^ The minor α-glycoside could arise from an S_N_2-like pathway from β-iodide **7** (which would be expected to be more reactive), whereas the major β-glycoside could come from S_N_2-like reaction with the major α-iodide **7** promoted by LiI (Scheme 3). Given the high β-selectivity observed, it seems that either the position of equilibrium strongly favours the α-iodide, or that equilibration is very slow under our conditions. An increase in the rate of equilibration at higher temperatures may explain why some substrates exhibited better selectivity at 25 °C than 45 °C. Mechanistically our method may be related to the earlier work with insoluble silver salts^1,2^ but has greater applicability, especially with respect to scope. In part, this may come from the greater reactivity of glycosyl iodides, but it also suggests that other β-glycosylation methods could be developed that do not rely on restricting the conformation of the donor.

Previously, it has been shown that mannosyl chlorides can be converted to α-mannosides with excellent selectivity.^38,67,68^ Our new method enables β-mannosides to be accessed from the same starting material. Thus mannosyl hemiacetals or chlorides are ‘bimodal donors’,^69^ representing a common precursor for all mannoside linkages, reducing by half the number of building blocks required for the synthesis of a library of oligosaccharides where both α and β-linkages are required.^70^

## Conclusions

In summary, we have developed a highly β-selective mannosylation and rhamnosylation protocol starting from readily available glycosyl hemi-acetals. The method uses commercially available reagents and straight-forward reaction conditions. It breaks with the prevailing paradigms in that it doesn't rely on protecting groups to conformationally constrain the donor and doesn't require cryogenic conditions or molecular sieves. The reaction proceeds through glycosyl chloride and then iodide intermediates. The mechanism will be the subject of further investigations in our laboratory, as will the extension of these conditions to other glycosides. We believe the method will be of immediate practical use to the community, and anticipate that it could pave the way for the development of new glycosylation reactions.

## Data availability

Detailed experimental procedures, characterisation, scope limitations and copies of NMR spectra are provided in the ESI.

## Author contributions

IP, DAP and JJR carried out investigations in the laboratory. EMM provided supervision. All authors contributed to the writing of the manuscript.

## Conflicts of interest

There are no conflicts to declare.

## Supplementary Material

SC-012-D1SC01300A-s001

SC-012-D1SC01300A-s002
