# Supplementary material for: Stereoselective β-mannosylations and β-rhamnosylations from glycosyl hemiacetals mediated by lithium iodide
Source: Chem Sci. 2021 Jul 5;12(29):10070–5. doi: 10.1039/d1sc01300a (PMC8317664; doi:10.1039/d1sc01300a)
Supplement: SC-012-D1SC01300A-s001 [file SC-012-D1SC01300A-s001.pdf]

Electronic Supplementary Material (ESI) for Chemical Science.
This journal is © The Royal Society of Chemistry 2021

Stereoselective β-Mannosylations and β-Rhamnosylations from Glycosyl Hemiacetals Mediated by Lithium Iodide

Imlirenla Pongener, Dionissia A. Pepe, Joseph J. Ruddy and Eoghan M. McGarrigle*

Centre for Synthesis & Chemical Biology, UCD School of Chemistry, University College Dublin, Belfield, Dublin 4, Ireland.

Supporting Information – Part 1

Table of Contents

General Experimental 5

Synthesis of Mannopyranosyl Donors 6

1,2,3,4,6-Penta-*O*-acetyl-α/β-D-mannopyranose S1 7

Ethyl 2,3,4,6-tetra-*O*-acetyl-1-thio-α-D-mannopyranoside S2 7

Ethyl 4,6-*O*-benzylidene-1-thio-α-D-mannopyranoside S3 8

Ethyl 2,3-di-*O*-benzyl-4,6-*O*-benzylidene-1-thio-α/β-D-mannopyranoside S4 9

Ethyl 2,3,6-tri-*O*-benzyl-1-thio-α-D-mannopyranoside S5 10

Ethyl 6-*O*-triisopropylsilyl-1-thio-α/β-D-mannopyranoside S6 10

Ethyl 2,3,4-tri-*O*-benzyl-6-*O*-triisopropylsilyl-1-thio-α-D-mannopyranoside S7 11

Ethyl 2,3,4-tri-*O*-(4-methylbenzyl)-6-*O*-triisopropylsilyl-1-thio-α-D-mannopyranoside S8 12

Ethyl 2,3,4-tri-*O*-benzyl-6-*O*-(4-methylbenzyl)-1-thio-α-D-mannopyranoside S9 13

Ethyl 2,3-di-*O*-isopropylidene-6-*O*-triisopropylsilyl-1-thio-α-D-mannopyranoside S10 14

Ethyl 2,3-di-*O*-isopropylidene-4-*O*-(4-methylbenzyl)-6-*O*-triisopropylsilyl-1-thio-α-D-mannopyranoside S11 14

2,3,4,6-Tetra-*O*-benzyl-α/β-D-mannopyranose 1a 15

2,3,4-Tri-*O*-benzyl-6-*O*-(4-methylbenzyl)-α/β-D-mannopyranose 1b 16

2,3,6-Tri-*O*-benzyl-4-*O*-(4-methylbenzyl)-α/β-D-mannopyranose 1c 17

2,3,4-Tri-*O*-benzyl-6-*O*-(4-methoxybenzyl)-α-D-mannopyranose 1d 19

2,3,4-Tri-*O*-benzyl-6-*O*-(2-naphthyl)-α/β-D-mannopyranose 1e 20

2,3,4-Tri-*O*-benzyl-6-*O*-*tert*-butyldiphenylsilyl-α/β-D-mannopyranose 1f 22

2,3,6-Tri-*O*-benzyl-4-*O*-*tert*-butyldimethylsilyl-α/β-D-mannopyranose 1g 23

6-*O*-Benzoyl-2,3,4-tri-*O*-benzyl-α/β-D-mannopyranose 1h 25

4-*O*-Acetyl-2,3,6-tri-*O*-benzyl-α/β-D-mannopyranose S22 26

2,3,4-Tri-*O*-benzyl-6-*O*-triisopropylsilyl-α/β-D-mannopyranose S23 ____ 28

2,3,4,6-Tetra-*O*-acetyl-α-D-mannopyranose S24 ____ 29

6-*O*-Pivaloyl-2,3,4-tri-*O*-*p*-methylbenzyl-α/β-D-mannopyranose 1i ____ 29

Acetyl 2,3,4,6-Tetra-*O*-benzyl-α/β-D-mannopyranose 8a ____ 31

Synthesis of Rhamnosyl Donors ____ 32

Ethyl 1-thiol-L-rhamnoside S28 ____ 33

Ethyl 2,3-*O*-isopropylidene-1-thio-L-rhamnoside S29 ____ 34

2,3,4-Tri-*O*-benzyl-L-rhamnoside 1j ____ 34

2,3-Di-*O*-benzyl-4-*O*-*p*-methylbenzyl-L-rhamnoside 1k ____ 36

2,3-Di-*O*-benzyl-4-*O*-benzoyl-L-rhamnoside 1l ____ 38

2,3,4-Tri-*O*-acetyl-L-rhamnoside S35 ____ 39

Synthesis of Acceptors ____ 40

Methyl 2,3,4-tri-*O*-benzyl-α-D-glucopyranoside 3a ____ 40

Methyl 4,6-*O*-benzylidene-α-D-glucopyranoside S38 ____ 41

Methyl 2,3-di-*O*-benzyl-4,6-*O*-benzylidene-α-D-glucopyranoside S39 ____ 41

Methyl 2,3,6-tri-*O*-benzyl-α-D-glucopyranoside 3b ____ 42

Methyl 2-*O*-benzyl-4,6-*O*-benzylidene-α-D-glucopyranoside 3d and Methyl 3-*O*-benzyl-4,6-*O*-benzylidene-α-D-glucopyranoside S40 ____ 43

Methyl 3-*O*-acetyl-2-*O*-benzyl-4,6-*O*-benzylidene-α-D-glucopyranoside S41 and Methyl 2-*O*-acetyl-3-*O*-benzyl-4,6-*O*-benzylidene-α-D-glucopyranoside S42 ____ 44

Methyl 3-*O*-acetyl-2,6-di-*O*-benzyl-α-D-glucopyranoside S43 and Methyl 2-*O*-acetyl-3,6-di-*O*-benzyl-α-D-glucopyranoside S44 ____ 45

Methyl 2,4,6-tri-*O*-benzyl-α-D-glucopyranoside 3c ____ 46

Methyl 4,6-*O*-benzylidene-α-D-galactopyranoside S45 ____ 46

Methyl 2-*O*-benzyl-3-*O*-acetyl-4,6-*O*-benzylidene-α-D-galactopyranoside S47 and Methyl 3-*O*-benzyl-2-*O*-acetyl-4,6-*O*-benzylidene-α-D-galactopyranoside S48 ____ 47

Methyl 2-*O*-benzyl-4,6-*O*-benzylidene-α-D-galactopyranoside S46 ____ 49

Methyl 3-*O*-benzyl-4,6-*O*-benzylidene-α-D-galactopyranoside 3j ____ 49

Ethyl 2,3,4-tri-*O*-(4-methylbenzyl)-1-thio-α-D-mannopyranoside 3i ____ 50

Methyl 2,3,4-tri-*O*-benzoyl-α-D-glucopyranoside S49 ____ 51

β-Mannosylations and β-Rhamnosylations ____ 52

Methyl (2,3,4,6-tetra-*O*-benzyl-β-D-mannopyranosyl)-(1→6)-2,3,4-tri-*O*-benzyl-α-D-glucopyranoside 2a ____ 54

Optimisation reactions (isolated yields and α/β ratios) ____ 55

Methyl (2,3,4-tri-*O*-benzyl-6-*O*-(4-methylbenzyl)-β-D-mannopyranosyl)-(1→6)-2,3,4-tri-*O*-benzyl-α-D-glucopyranoside 2b ____ 55

Methyl (2,3,6-tri-*O*-benzyl-4-*O*-(4-methylbenzyl)-β-D-mannopyranosyl)-(1→6)-2,3,4-tri-*O*-benzyl-α-D-glucopyranoside 2c ____ 56

Methyl (2,3,4-tri-*O*-benzyl-6-*O*-(4-methoxylbenzyl)-β-D-mannopyranosyl)-(1→6)-2,3,4-tri-*O*-benzyl-α-D-glucopyranoside 2d 57
Methyl (2,3,4-tri-*O*-benzyl-6-*O*-(2-naphthyl)-β-D-mannopyranosyl)-(1→6)-2,3,4-tri-*O*-benzyl-α-D-glucopyranoside 2e 58
Methyl (2,3,4-tri-*O*-benzyl-6-*O*-*tert*-butyldiphenylsilyl-β-D-mannopyranosyl)-(1→6)-2,3,4-tri-*O*-benzyl-α-D-glucopyranoside 2f 59
Methyl (2,3,6-tri-*O*-benzyl-4-*O*-*tert*-butyldimethylsilyl-β-D-mannopyranosyl)-(1→6)-2,3,4-tri-*O*-benzyl-α-D-glucopyranoside 2g 60
Methyl (2,3,4-tri-*O*-benzyl-6-*O*-(benzoyl)-α/β-D-mannopyranosyl)-(1→6)-2,3,4-tri-*O*-benzyl-α-D-glucopyranoside 2h 61
Methyl (2,3,4-tri-*O*-*p*-methylbenzyl-6-*O*-(pivaloyl)-β-D-mannopyranosyl)-(1→6)-2,3,4-tri-*O*-benzyl-α-D-glucopyranoside 2i 62
Methyl 2,3,4-tri-*O*-benzyl-6-*O*-(2,3,4-tri-*O*-benzyl-β-L-rhamnosyl)-α-D-glucopyranoside 2j 63
Methyl 2,3,4-tri-*O*-benzyl-6-*O*-(2,3-di-*O*-benzyl-4-*O*-*p*-methylbenzyl-β-L-rhamnosyl)-α-D-glucopyranoside 2k 64
Methyl 2,3,4-tri-*O*-benzyl-6-*O*-(2,3-di-*O*-benzyl-4-*O*-benzoyl-β-L-rhamnosyl)-α-D-glucopyranoside 2l 65
Methyl (2,3,4,6-tetra-*O*-benzyl-β-D-mannopyranosyl)-(1→4)-2,3,6-tri-*O*-benzyl-α-D-glucopyranoside 4b 66
Methyl (2,3,4,6-tetra-*O*-benzyl-β-D-mannopyranosyl)-(1→3)-2,4,6-tri-*O*-benzyl-α-D-glucopyranoside 4c 67
Methyl (2,3,4,6-tetra-*O*-benzyl-β-D-mannopyranosyl)-(1→3)-2-*O*-benzyl-4,6-*O*-benzylidene-α-D-glucopyranoside 4d 68
Phenyl 2,3,4,6-tetra-*O*-benzyl-β-D-mannopyranoside 4e 68
1-Naphthyl 2,3,4,6-tetra-*O*-benzyl-β-D-mannopyranoside 4f 69
p-Nitrophenyl 2,3,4,6-tetra-*O*-benzyl-β-D-mannopyranoside 4g 70
p-Methoxyphenyl (2,3,4,6-tetra-*O*-benzyl-β-D-mannopyranosyl)-(1→4)-2-azido-3,6-di-*O*-benzyl-β-D-glucopyranoside 4h 71
Methyl 2,3,6-tri-*O*-benzyl-4-*O*-(2,3,4-tri-*O*-benzyl-β-D-rhamnosyl)-α-D-glucopyranoside 5b 72
Methyl 2,4,6-tri-*O*-benzyl-3-*O*-(2,3,4-tri-*O*-benzyl-β-L-rhamnosyl)-α-D-glucopyranoside 5c 73
Methyl 2-*O*-benzyl-4,6-benzylidene-3-*O*-(2,3,4-tri-*O*-benzyl-β-L-rhamnosyl)-α-D-glucopyranoside 5d 74
Phenyl 2,3,4-tri-*O*-benzyl-β-L-rhamnoside 5e 74
Naphthyl 2,3,4-tri-*O*-benzyl-β-L-rhamnoside 5f 75
p-Nitrophenyl 2,3,4-tri-*O*-benzyl-β-L-rhamnoside 5g 76
p-Methoxyphenyl 2-azido-3,6-di-*O*-benzyl-4-*O*-(2,3,4-tri-*O*-benzyl-β-L-rhamnosyl)-β-D-glucopyranoside 5h 76
Ethyl 1-thiol 2,3,4-tri-*O*-benzyl-6-*O*-(2,3,4-tri-*O*-*p*-methylbenzyl-β-L-rhamnosyl)-α-D-mannopyranoside 5i 77
Methyl 3-*O*-benzyl-4,6-benzylidene-2-*O*-(2,3,4-tri-*O*-benzyl-β-L-rhamnosyl)-α-D-galactopyranoside 5j 78
Boc-L-tyrosine methyl ester 2,3,4-tri-*O*-benzyl-β-L-rhamnoside 5k 79

Cholesteryl 2,3,4-tri-*O*-benzyl-α/β-L-rhamnoside S50 ____ 80

Donor and Acceptor Limitations ____ 81

Mechanistic Investigations ____ 82

References ____ 87

General Experimental

The reagents and solvents used in the following experiments were bought commercially and used without further purification. Oxalyl chloride from a fresh bottle was immediately stored in a Young's tube under a nitrogen atmosphere. In a glove-box, anhydrous lithium iodide beads were powdered. The powdered LiI was stored in capped vials on the bench for several weeks before use. It should be a free-flowing white solid. Dry solvents were obtained using equipment based on Grubb's design[1] and stored in Strauss flask over 4 Å molecular sieves. A Karl Fischer Titrator was used to determine the amount of water in dry solvents. For air-sensitive reactions, solvents were added *via* syringe through rubber septa. Reactions were monitored by thin layer chromatography using silica-coated aluminium plates and the eluents outlined in the respective experiments; spots were detected under 254 nm UV light. Flash column chromatography was performed using silica gel [Davisil, 400–230 mesh (63–40 μm)]. ^{1}H NMR, ^{13}C NMR and 2D NMR were carried out on 400 MHz, 500 MHz or 600 MHz spectrometers using deuterated chloroform ($CDCl_3$). Chemical shifts are reported in parts per million (ppm), coupling constants (*J*) are reported in Hertz (Hz) and multiplicities are abbreviated as; s (singlet), d (doublet), t (triplet) or m (multiplet) or combinations thereof. Chemical shifts were referenced to the residual proton of TMS for ^{1}H NMR spectra and to the ^{13}C signal of deuterated chloroform ($CDCl_3$) for ^{13}C NMR spectra. For compounds not reported in literature, NMR assignments have been made using COSY, HSQC and HMBC. Mass Spectra were recorded by the University College Dublin, School of Chemistry mass spectrometry service using ESI-MS and GCMS techniques.

4-Methoxyphenyl 2-azido-3,6-di-O-benzyl-2-deoxy-β-D-glucopyranoside **3h** was purchased from Carbosynth.

Synthesis of Mannopyranosyl Donors

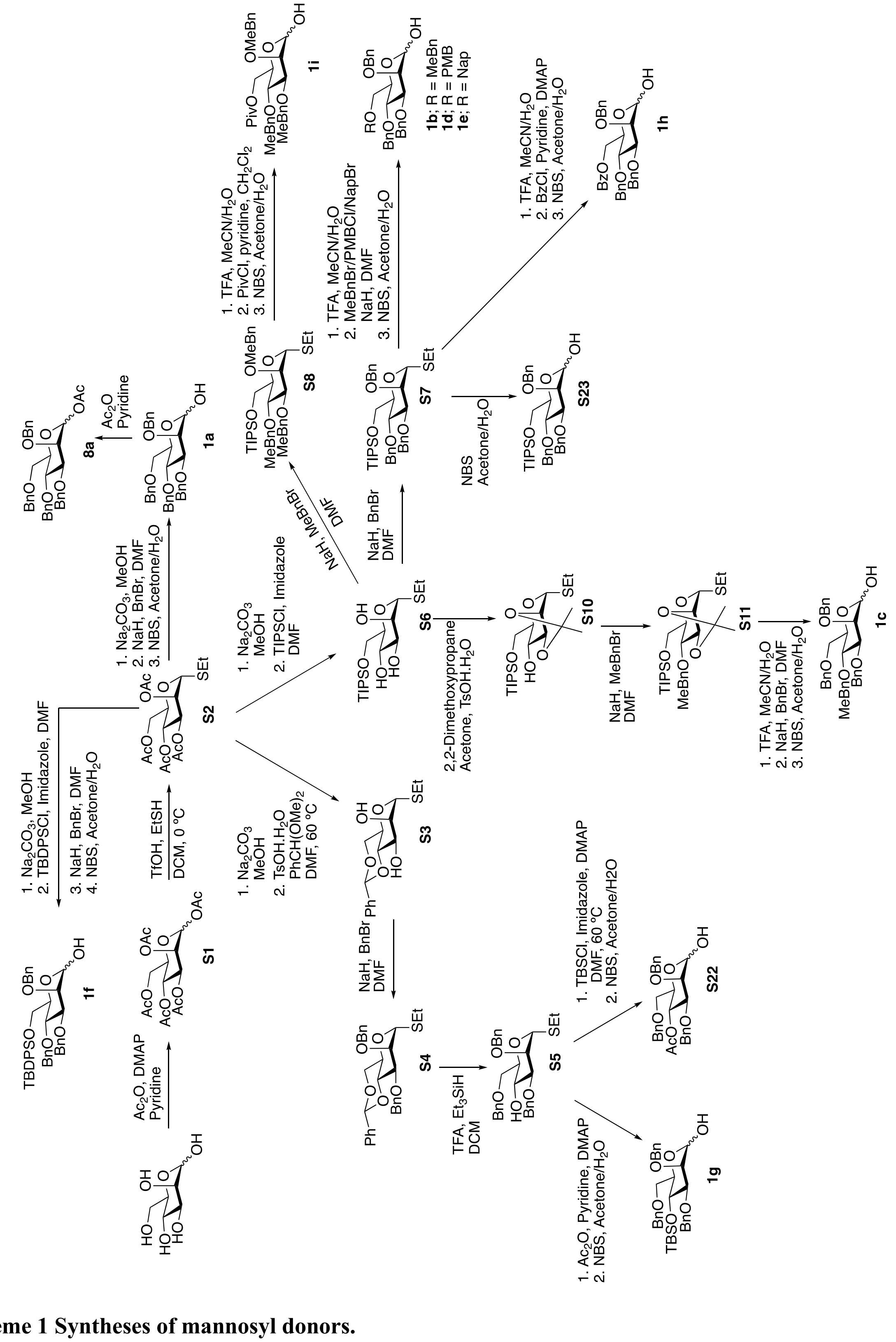

Scheme 1 Syntheses of mannosyl donors.

1,2,3,4,6-Penta-*O*-acetyl-α/β-D-mannopyranose S1

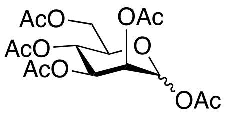

A solution of D-mannose (1.0 g, 5.5 mmol) in pyridine (2 mL) was treated with acetic anhydride (5.2 mL, 55 mmol) and DMAP (67 mg, 0.55 mmol) and stirred at room temperature. TLC (1:1; cyclohexane/EtOAc) analysis after 3 h showed complete consumption of starting material. The reaction mixture was diluted with CH_2Cl_2 and washed with 1 M HCl, saturated $NaHCO_3$, and brine. The organic layer was dried over anhydrous $MgSO_4$, filtered and concentrated *in vacuo*
to give **S1** as a colourless syrup (2.1 g, quantitative crude, α/β = 78:22). NMR data were consistent with literature data.[2]

The following were observed for α/β anomers:

^{1}H NMR (500 MHz, Chloroform-*d*): δ 2.06 (s, 3H, CH_3), 2.02 (s, 3H, CH_3).

α-anomer

^{1}H NMR (500 MHz, Chloroform-*d*): δ 6.05 (d, *J* = 1.9 Hz, 1H, H-1), 5.34 – 5.29 (m, 2H, H-3, H-4), 5.24 – 5.22 (m, 1H, H-2), 4.25 (dd, *J* = 12.4, 4.8 Hz, 1H, H-6a), 4.07 (dd, *J* = 12.4, 2.5 Hz, 1H, H-6b), 4.05 – 3.99 (m, 1H, H-5), 2.15 (s, 3H, CH_3), 2.14 (s, 3H, CH_3), 1.975 (s, 3H, CH_3).

β-anomer

^{1}H NMR (500 MHz, Chloroform-*d*): δ 5.84 (d, *J* = 1.2 Hz, 1H, H-1), 5.45 (dd, *J* = 3.3, 1.2 Hz, 1H, H-2), 5.30 – 5.22 (m, 1H, H-4), 5.11 (dd, *J* = 10.0, 3.3 Hz, 1H, H-3), 4.31 – 4.25 (m, 1H, H-6a), 4.11 (dd, *J* = 12.4, 2.4 Hz, 1H, H-6b), 3.78 (ddd, *J* = 9.9, 5.3, 2.4 Hz, 1H, H-5), 2.18 (s, 3H, CH_3), 2.07 (s, 3H, CH_3), 1.972 (s, 3H, CH_3).

Ethyl 2,3,4,6-tetra-*O*-acetyl-1-thio-α-D-mannopyranoside S2

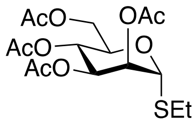

Under a N_2 atmosphere, a solution of pentaacetate mannose **S1** (22 g, 56 mmol) and 4Å powdered molecular sieves in anhydrous CH_2Cl_2 (200 mL) was treated with ethanethiol (12.0 mL, 161 mmol) at room temperature. The reaction mixture was stirred at 0 °C for 30 min after which $BF_3.Et_2O$ (21.0 mL, 169 mmol) was slowly added. After stirring the reaction mixture at room temperature for 18 h, the

reaction was quenched with saturated $NaHCO_3$. The organic layer was washed with water and brine, dried over anhydrous $MgSO_4$ and concentrated *in vacuo* to give a yellow paste. Purification by column chromatography (2:1; cyclohexane/EtOAc) gave the title compound **S2** as a white solid (15 g, 68% yield). ^{1}H NMR (500 MHz, Chloroform-*d*): δ 5.34 (dd, *J* = 3.2, 1.6 Hz, 1H, H-2), 5.32 (t, *J* = 9.9 Hz, 1H, H-4), 5.29 (d, *J* = 0.9 Hz, 1H, H-1), 5.27 (dd, *J* = 9.9, 3.3 Hz, 1H, H-3), 4.40 (ddd, *J* = 9.4, 5.3, 2.3 Hz, 1H, H-5), 4.32 (dd, *J* = 12.2, 5.4 Hz, 1H, H-6a), 4.10 (dd, *J* = 12.2, 2.4 Hz, 1H, H-6b), 2.74 – 2.55 (m, 2H, $SC\mathit{H}_2CH_3$), 2.17 (s, 3H, CH_3), 2.10 (s, 3H, CH_3), 2.05 (s, 3H, CH_3), 1.99 (s, 3H, CH_3), 1.31 (t, *J* = 7.4 Hz, 3H, $SCH_2C\mathit{H}_3$). ^{13}C NMR (126 MHz, Chloroform-*d*): δ 170.7 (C=O), 170.1 (C=O), 169.91 (C=O), 169.86 (C=O), 82.4 (C-1), 71.3 (C-2), 69.6 (C-3), 69.1 (C-5), 66.5 (C-4), 62.6 (C-6), 25.6 ($S\mathit{C}H_2CH_3$), 21.1 (CH_3), 20.86 (CH_3), 20.85 (CH_3), 20.78 (CH_3), 14.90 ($SCH_2\mathit{C}H_3$). NMR data were consistent with literature data.[3]

Ethyl 4,6-*O*-benzylidene-1-thio-α-D-mannopyranoside S3

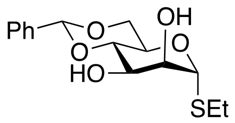

A solution of **S2** (10.9 g, 27.8 mmol) in methanol (100 mL) was treated with Na_2CO_3 (883 mg, 8.34 mmol) and stirred at room temperature for 4 h. The reaction mixture was neutralised with resin IR-120, filtered and concentrated *in vacuo* to give a brown syrup. A solution of the syrup in MeCN (100 mL) was treated with $TsOH.H_2O$ (529 mg, 2.78 mmol), followed by benzaldehyde dimethyl acetal (5.0 mL, 34 mmol) and stirred at 60 °C for 8 h. The reaction mixture was concentrated *in vacuo*, re-dissolved in CH_2Cl_2, washed with saturated $NaHCO_3$ and brine, dried over anhydrous $MgSO_4$ and concentrated *in vacuo*. After purification by column chromatography (100:0 to 80:20; CH_2Cl_2/Et_2O), the title compound **S3** was obtained as a white solid (2.4 g, 28% yield over 2 steps). ^{1}H NMR (500 MHz, Chloroform-*d*): δ 7.54 – 7.45 (m, 2H, ArCH), 7.44 – 7.31 (m, 3H, ArCH), 5.56 (s, 1H, PhC*H*), 5.36 (d, *J* = 1.1 Hz, 1H, H-1), 4.28 – 4.18 (m, 2H H-6a, H-3), 4.11 (dt, *J* = 3.1, 1.4 Hz, 1H, H-2), 4.05 (dt, *J* = 9.7, 3.3 Hz, 1H, H-5), 3.96 (t, *J* = 9.3 Hz, 1H, H-4), 3.89 – 3.79 (m, 1H, H-6b), 2.88 (d, *J* = 2.0 Hz, 1H, OH), 2.80 (d, *J* = 3.3 Hz, 1H, OH), 2.73 – 2.52 (m, 2H, $SC\mathit{H}_2CH_3$), 1.30 (t, *J* = 7.4 Hz, 3H, $SCH_2C\mathit{H}_3$). NMR data were consistent with literature data.[4]

Ethyl 2,3-di-*O*-benzyl-4,6-*O*-benzylidene-1-thio-α/β-D-mannopyranoside S4

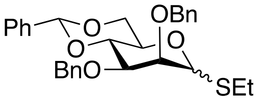

Under a N_2 atmosphere, a solution of mannopyranoside **S3** (2.4 g, 7.7 mmol) in anhydrous DMF (17 mL) was cooled to 0 °C and NaH (60% dispersion in mineral oil) (1.1 g, 27 mmol) was added. The reaction mixture was stirred at room temperature for 30 min after which it was again cooled down to 0 °C. Benzyl bromide (3.2 mL, 27 mmol) was added dropwise and the reaction mixture was left to stir at room temperature for 4 h. The reaction was quenched with MeOH and the mixture was concentrated *in vacuo* to give a yellow slurry. The slurry was diluted with CH_2Cl_2 (150 mL) and washed with 1 M HCl, followed by saturated $NaHCO_3$ and brine, dried over anhydrous $MgSO_4$ and concentrated *in vacuo*. Purification by column chromatography (95:5 to 0:100; Pentane/Et_2O) gave **S4** as clear yellowish syrup (3.1 g, 81% yield, α/β = 93:7).

The following were observed for α/β anomers:

^{1}H NMR (500 MHz, Chloroform-*d*): δ 7.52 – 7.48 (m, 2H, ArCH), 7.41 – 7.26 (m, 13H, ArCH).

α-anomer

^{1}H NMR (500 MHz, Chloroform-*d*): δ 5.64 (s, 1H, PhC*H*), 5.30 (d, *J* = 1.3 Hz, 1H, H-1), 4.79 (d, *J* = 12.2 Hz, 1H, C*H*HPh), 4.76 (d, *J* = 12.3 Hz, 1H, C*H*HPh), 4.72 (d, *J* = 12.2 Hz, 1H, C*H*HPh), 4.62 (d, *J* = 12.1 Hz, 1H, C*H*HPh), 4.27 (t, *J* = 9.5 Hz, 1H, H-4), 4.23 – 4.15 (m, 2H, H-6a, H-5), 3.95 – 3.85 (m, 3H, H-3, H-6b, H-2), 2.65 – 2.48 (m, 2H, SCH_2CH_3), 1.23 (t, *J* = 7.4 Hz, 3H, SCH_2CH_3). ^{13}C NMR (126 MHz, Chloroform-*d*) δ 138.6 (C), 138.1 (C), 137.8 (C), 129.0 (CH), 128.6 (CH), 128.4 (CH), 128.3 (CH), 128.2 (CH), 128.0 (CH), 127.8 (CH), 127.7 (CH), 126.2 (CH), 101.6 (Ph*C*H), 83.7 (C-1), 79.4 (C-4), 78.3 (C-2), 76.6 (C-3), 73.3 ($PhCH_2$), 73.2 ($PhCH_2$), 68.8 (C-6), 64.8 (C-5), 25.5 (SCH_2CH_3), 15.1 (SCH_2CH_3). NMR data were consistent with literature data.[5]

β-anomer

^{1}H NMR (500 MHz, Chloroform-*d*) selected signals: δ 5.62 (s, 1H, PhC*H*), 5.01 (d, *J* = 11.2 Hz, 1H, C*H*HPh), 4.86 (d, *J* = 12.4 Hz, 1H, C*H*HPh), 4.81 (d, *J* = 11.2 Hz, 1H, C*H*HPh), 4.72 (d, *J* = 12.4 Hz, 1H, C*H*HPh), 4.64 (d, *J* = 1.2 Hz, 1H, H-1), 4.31 – 4.24 (m, 1H, H-4), 4.01 (dd, *J* = 3.2, 1.2 Hz, 1H, H-2), 3.72 (dd, *J* = 9.9, 3.1 Hz, 1H, H-3), 3.40 (ddd, *J* = 10.1, 9.2, 5.0 Hz, 1H, H-5), 1.28 (t, *J* = 7.4 Hz, 3H, SCH_2CH_3).

Ethyl 2,3,6-tri-*O*-benzyl-1-thio-α-D-mannopyranoside S5

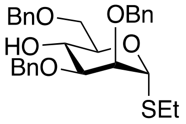

Based on the literature procedure,[6] under a N_2 atmosphere, a solution of **S4** (2.0 g, 4.1 mmol) in anhydrous CH_2Cl_2 (14 mL) was cooled to 0 °C and treated slowly with trifluoroacetic acid (1.9 mL, 25 mmol) followed by triethylsilane (4.0 mL, 25 mmol). The reaction mixture was warmed up to room temperature and stirred for 45 minutes. The reaction was diluted with CH_2Cl_2 and quenched with saturated $NaCHO_3$. The organic layer was washed with brine, dried over anhydrous $MgSO_4$ and concentrated *in vacuo* to give a yellow syrup. Purification by column chromatography (3:1 to 2:1; Pentane/Et_2O) gave **S5** as a yellowish syrup (1.7 g, 85% yield). ^{1}H NMR (500 MHz, Chloroform-*d*): δ 7.41 – 7.21 (m, 15H, ArCH), 5.41 (d, *J* = 1.4 Hz, 1H, H-1), 4.70 (d, *J* = 12.2 Hz, 1H, C*H*HPh), 4.63 (d, *J* = 12.1 Hz, 1H, C*H*HPh), 4.58 (d, *J* = 12.2 Hz, 1H, C*H*HPh), 4.56 (d, *J* = 12.1 Hz, 1H, C*H*HPh), 4.54 (d, *J* = 11.7 Hz, 1H, C*H*HPh), 4.47 (d, *J* = 11.7 Hz, 1H, C*H*HPh), 4.15 – 4.05 (m, 2H, H-4, H-5), 3.84 (dd, *J* = 3.2, 1.5 Hz, 1H, H-2), 3.81 – 3.75 (m, 2H, H-6a, H-6b), 3.66 (dd, *J* = 9.1, 3.1 Hz, 1H, H-3), 2.70 – 2.53 (m, 2H, SCH_2CH_3), 2.48 (d, *J* = 1.8 Hz, 1H, OH), 1.26 (t, *J* = 7.4 Hz, 3H, SCH_2CH_3). ^{13}C NMR (126 MHz, Chloroform-*d*): δ 138.4 (C), 138.1 (C), 138.0 (C), 128.6 (CH), 128.5 (CH), 128.4 (CH), 128.1 (CH), 128.0 (CH), 127.9 (CH), 127.7 (CH), 127.6 (CH), 82.1 (C-1), 79.9 (C-3), 75.8 (C-2), 73.6 ($PhCH_2$), 72.2 ($PhCH_2$), 71.9 ($PhCH_2$), 71.8 (C-5), 70.3 (C-6), 68.0 (C-4), 25.5 (SCH_2CH_3), 15.1 (SCH_2CH_3). NMR data were consistent with literature data.[4]

Ethyl 6-*O*-triisopropylsilyl-1-thio-α/β-D-mannopyranoside S6

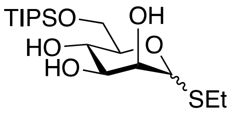

A solution of **S2** (13 g, 33 mmol) in methanol (250 mL) was treated with Na_2CO_3 (1.0 g, 9.9 mmol) and stirred at room temperature for 2 h. The reaction mixture was neutralised with resin IR-120, filtered and concentrated *in vacuo* to give a brown paste. Under a N_2 atmosphere, a solution of the crude material and imidazole (6.7 g, 99 mmol) in anhydrous DMF (100 mL) was treated with triisopropylsilyl chloride (11 mL, 52 mmol) and left to stir at room temperature for 2 h. The reaction mixture was diluted with CH_2Cl_2 and washed with water and brine, dried over anhydrous $MgSO_4$ and concentrated *in vacuo* to give a brown syrup. Purification by column chromatography (100:0 to 95:5; CH_2Cl_2/MeOH) gave **S6** as

a white solid (11 g, 85% yield over 2 steps, α/β = 90:10). ESI-HRMS for $C_{17}H_{36}O_5SSiNa^+$ $(M+Na)^+$ calculated: 403.1945; found: 403.1948.

α-anomer

^{1}H NMR (600 MHz, Chloroform-*d*): δ 5.30 (d, *J* = 1.4 Hz, 1H, H-1), 4.07 – 4.00 (m, 2H, H-2, H-5), 4.00 – 3.92 (m, 2H, H-6a, H-6b), 3.89 – 3.80 (m, 2H, H-3, H-4), 3.62 (s, 1H, OH), 3.02 (d, *J* = 3.8 Hz, 1H, OH), 2.80 (d, *J* = 3.5 Hz, 1H, OH), 2.66 (dq, *J* = 12.9, 7.4 Hz, 1H, SC*H*HCH$_3$), 2.58 (dq, *J* = 13.0, 7.4 Hz, 1H, SC*H*HCH$_3$), 1.29 (t, *J* = 7.4 Hz, 3H, SCH$_2$C*H*$_3$), 1.18 – 1.11 (m, 3H, SiC*H*(CH$_3$)$_2$), 1.08 (s, 12H, SiCH(C*H*$_3$)$_2$), 1.07 (s, 6H, SiCH(C*H*$_3$)$_2$). ^{13}C NMR (151 MHz, Chloroform-*d*): δ 83.9 (C-1), 72.25 (C-3/4), 72.22 (C-3/4), 71.8 (C-2), 70.2 (C-5), 66.0 (C-6), 25.1 (S*C*H$_2$CH$_3$), 18.01 (SiCH(*C*H$_3$)$_2$), 15.0 (SCH$_2$*C*H$_3$), 11.87 (Si*C*H(CH$_3$)$_2$).

β-anomer

^{1}H NMR (600 MHz, Chloroform-*d*) selected signals: δ 4.68 (d, J = 1.0 Hz, 1H, H-1), 4.07 – 4.00 (m, 2H, H-2, H-6a), 4.00 – 3.92 (m, 1H, H-6b), 3.89 – 3.80 (m, 1H, H-4), 3.72 (s, 1H, OH), 3.62 (br s, 1H, H-3), 3.36 (ddd, J = 9.3, 7.1, 5.2 Hz, 1H, H-5), 2.73 (dqd, J = 11.5, 7.4, 3.7 Hz, 2H, SC*H*$_2$CH$_3$). ^{13}C NMR (151 MHz, Chloroform-*d*) selected signals: δ 83.8 (C-1), 77.9 (C-5), 75.2 (C-3), 71.7 (C-2 or C-4), 65.9 (C-6), 25.4 (S*C*H$_2$CH$_3$), 15.2 (SCH$_2$*C*H$_3$).

Ethyl 2,3,4-tri-*O*-benzyl-6-*O*-triisopropylsilyl-1-thio-α-D-mannopyranoside S7

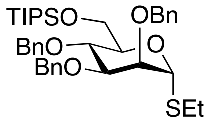

Under a N_2 atmosphere, a solution of mannopyranoside **S6** (2.00 g, 5.2 mmol) in anhydrous DMF (8.4 mL) was cooled to 0 °C and NaH (60% dispersion in mineral oil) (757 mg, 18.9 mmol) was added. The reaction mixture was stirred at room temperature for 30 min after which it was again cooled down to 0 °C. Benzyl bromide (2.2 mL, 19 mmol) was added dropwise and the reaction mixture was left to stir at room temperature for 2 h. The reaction was quenched with MeOH and diluted with pentane (150 mL). The aqueous layer was washed with pentane (2 x 40 mL). The organic layers were combined and neutralised with 1 M HCl. The organic layer was washed with water and brine, dried over anhydrous $MgSO_4$ and concentrated *in vacuo* to give a yellow syrup. Purification by column chromatography (98:2 to 95:5; Pentane/Et_2O) gave **S7** as a clear colourless syrup (3.0 g, 88% yield). ^{1}H NMR (600 MHz, Chloroform-*d*): δ 7.39 – 7.23 (m, 15H, ArCH), 5.33 (d, *J* = 1.5 Hz, 1H, H-1), 4.92 (d, *J* = 11.0 Hz, 1H, C*H*HPh), 4.66 (s, 2H, 2 x C*H*HPh), 4.65 – 4.60 (m, 2H, 2 x C*H*HPh), 4.58 (d, *J* = 11.7 Hz, 1H, C*H*HPh), 3.98 (ddd, *J* = 9.5, 4.5, 2.4 Hz, 1H, H-5), 3.96 – 3.91 (m, 3H, H-4, H-6a, H-6b), 3.86 (dd, *J* = 8.9, 3.2

Hz, 1H, H-3), 3.80 (dd, *J* = 3.2, 1.6 Hz, 1H, H-2), 2.62 (dq, *J* = 12.8, 7.4 Hz, 1H, SC*H*HCH$_3$), 2.53 (dq, *J* = 12.8, 7.5 Hz, 1H, SC*H*HCH$_3$), 1.22 (t, *J* = 7.4 Hz, 3H, SCH$_2$C*H*$_3$), 1.12 – 1.03 (m, 3H, SiC*H*(CH$_3$)$_2$), 1.06 (s, 12H, SiCH(C*H*$_3$)$_2$), 1.05 (s, 6H, SiCH(C*H*$_3$)$_2$). ^{13}C NMR (151 MHz, Chloroform-*d*): δ 138.9 (C), 138.5 (C), 138.4 (C), 128.49 (CH), 128.46 (CH), 128.42 (CH), 128.2 (CH), 128.0 (CH), 127.9 (CH), 127.8 (CH), 127.7 (CH), 81.3 (C-1), 80.6 (C-3), 76.8 (C-2), 75.28 (Ph*C*H$_2$), 75.25 (C-4), 73.8 (C-5), 72.3 (Ph*C*H$_2$), 72.1 (Ph*C*H$_2$), 63.3 (C-6), 25.0 (S*C*H$_2$CH$_3$), 18.14 (SiCH(*C*H$_3$)$_2$), 18.11 (SiCH(*C*H$_3$)$_2$), 14.9 (SCH$_2$*C*H$_3$), 12.2 (Si*C*H(CH$_3$)$_2$). ESI-HRMS for $C_{38}H_{54}O_5SSiNa^+$ $(M+Na)^+$ calculated: 673.3353; found: 673.3351.

Ethyl 2,3,4-tri-*O*-(4-methylbenzyl)-6-*O*-triisopropylsilyl-1-thio-α-D-mannopyranoside S8

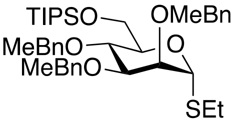

Based on the literature procedure,[7] under a N_2 atmosphere, a solution of mannopyranoside **S6** (1.2 g, 3.2 mmol) in anhydrous DMF (11 mL) was cooled to 0 °C and NaH (60% dispersion in mineral oil) (577 mg, 14.4 mmol) was added. The reaction mixture was stirred at room temperature for 30 min after which it was again cooled down to 0 °C. 4-Methylbenzyl bromide (2.66 g, 14.4 mmol) was added and the reaction mixture was left to stir at room temperature for 5 h. The reaction was quenched with MeOH and diluted with Et_2O (100 mL). The aqueous layer was washed with Et_2O (2 x 40 mL). The organic layers were combined and neutralised with 1 M HCl. The organic layer was washed with water and brine, dried over anhydrous $MgSO_4$ and concentrated *in vacuo* to give a yellow oil. Purification by column chromatography (99:1 to 95:5; Pentane/Et_2O) gave **S8** as a clear colourless syrup (1.7 g, 77% yield). ^{1}H NMR (600 MHz, Chloroform-*d*): δ 7.28 – 7.16 (m, 6H, ArCH), 7.14 – 7.07 (m, 6H, ArCH), 5.30 (d, *J* = 1.5 Hz, 1H, H-1), 4.86 (d, *J* = 10.7 Hz, 1H, C*H*HPh), 4.62 (s, 2H, 2 x C*H*HPh), 4.55 (d, *J* = 11.3 Hz, 2H, 2 x C*H*HPh), 4.52 (d, *J* = 11.5 Hz, 1H, C*H*HPh), 3.94 (ddd, *J* = 9.4, 5.7, 1.9 Hz, 1H, H-5), 3.91 – 3.83 (m, 3H, H-4, H-6a, H-6b), 3.82 (dd, *J* = 9.2, 3.1 Hz, 1H, H-3), 3.76 (dd, *J* = 3.1, 1.6 Hz, 1H, H-2), 2.60 (dq, *J* = 12.8, 7.3 Hz, 1H, SC*H*HCH$_3$), 2.51 (dq, *J* = 12.9, 7.5 Hz, 1H, SC*H*HCH$_3$), 2.343 (s, 3H, CH$_3$), 2.341 (s, 3H, CH$_3$), 2.33 (s, 3H, CH$_3$), 1.21 (t, *J* = 7.4 Hz, 3H, SCH$_2$C*H*$_3$), 1.10 – 1.05 (m, 3H, SiC*H*(CH$_3$)$_2$), 1.05 (s, 12H, SiCH(C*H*$_3$)$_2$), 1.04 (s, 6H, SiCH(C*H*$_3$)$_2$). ^{13}C NMR (151 MHz, Chloroform-*d*): δ 137.37 (C), 137.35 (2 x C), 135.9 (C), 135.6 (C), 135.4 (C), 129.2 (CH), 129.13 (CH), 129.11 (CH), 128.3 (CH), 128.1 (CH), 81.2 (C-1), 80.5 (C-3), 76.4 (C-2), 75.1 (Ph*C*H$_2$ and C-4), 73.8 (C-5), 72.1 (Ph*C*H$_2$), 71.9 (Ph*C*H$_2$), 63.3 (C-6), 24.9 (S*C*H$_2$CH$_3$), 21.34 (CH$_3$), 21.33 (CH$_3$), 21.31 (CH$_3$), 18.13 (SiCH(*C*H$_3$)$_2$), 18.11 (SiCH(*C*H$_3$)$_2$), 14.9 (SCH$_2$*C*H$_3$), 12.1 (Si*C*H(CH$_3$)$_2$). ESI-HRMS for $C_{41}H_{64}O_5SSiN^+$ $(M+NH_4)^+$ calculated: 710.4269; found: 710.4268.

Ethyl 2,3,4-tri-*O*-benzyl-6-*O*-(4-methylbenzyl)-1-thio-α-D-mannopyranoside S9

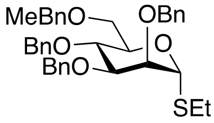

Based on the literature procedure,[8] a solution of **S7** (1.0 g, 1.5 mmol) in MeCN/H_2O (4:1, 15 mL) was treated with trifluoroacetic acid (0.94 mL, 12 mmol) at room temperature. After stirring for 16 h at room temperature, the reaction was quenched with saturated $NaHCO_3$ and diluted with CH_2Cl_2 (100 mL). The aqueous layer was washed with CH_2Cl_2 (2 x 100 mL). The combined organic layers were washed with brine, dried over anhydrous $MgSO_4$ and concentrated *in vacuo* to give a clear syrup. The crude material was used in the next step without further purification. Based on the literature procedure,[7] under a N_2 atmosphere, a solution of the crude mannopyranoside in anhydrous DMF (5 mL) was cooled to 0 °C and NaH (60% dispersion in mineral oil) (154 mg, 3.85 mmol) was added. The reaction mixture was stirred at room temperature for 30 min after which it was again cooled down to 0 °C. 4-Methylbenzyl bromide (712 mg, 3.85 mmol) was added and the reaction mixture was left to stir at room temperature for 2 h. The reaction was quenched with MeOH and diluted with Et_2O (100 mL). The aqueous layer was washed with Et_2O (2 x 40 mL). The organic layers were combined and neutralised with 1 M HCl. The organic layer was washed with water and brine, dried over anhydrous $MgSO_4$ and concentrated *in vacuo* to give a yellow oil. Purification by column chromatography (90:10 to 60:40; Pentane/Et_2O) gave **S9** as a yellowish syrup (850 mg, 92% yield over 2 steps). ^{1}H NMR (500 MHz, Chloroform-*d*): δ 7.41 – 7.35 (m, 2H, ArCH), 7.34 – 7.20 (m, 13H, ArCH), 7.15 (dd, *J* = 7.5, 2.1 Hz, 2H, ArCH), 7.10 (d, *J* = 7.8 Hz, 2H, ArCH), 5.40 (d, *J* = 1.3 Hz, 1H, H-1), 4.86 (d, *J* = 10.8 Hz, 1H, C*H*HPh), 4.73 (d, *J* = 12.4 Hz, 1H, C*H*HPh), 4.68 – 4.61 (m, 2H, 2 x C*H*HPh), 4.58 (d, *J* = 11.8 Hz, 1H, C*H*HPh), 4.55 (d, *J* = 11.8 Hz, 1H, C*H*HPh), 4.47 (d, *J* = 10.8 Hz, 1H, C*H*HPh), 4.46 (d, *J* = 11.9 Hz, 1H, C*H*HPh), 4.11 (ddd, *J* = 9.8, 4.8, 1.9 Hz, 1H, H-5), 4.05 – 3.98 (m, 1H, H-4), 3.86 – 3.81 (m, 2H, H-2, H-3), 3.80 (dd, *J* = 10.8, 4.7 Hz, 1H, H-6a), 3.68 (dd, *J* = 10.8, 1.9 Hz, 1H, H-6b), 2.69 – 2.49 (m, 2H, SCH_2CH_3), 2.31 (s, 3H, CH_3), 1.24 (t, *J* = 7.4 Hz, 3H, SCH_2CH_3). ^{13}C NMR (126 MHz, Chloroform-*d*): δ 138.7 (C), 138.4 (C), 138.3 (C), 137.2 (C), 135.4 (C), 129.1 (CH), 128.49 (CH), 128.48 (CH), 128.4 (CH), 128.09 (CH), 128.06 (CH), 128.0 (CH), 127.9 (CH), 127.8 (CH), 127.74 (CH), 127.65 (CH), 82.0 (C-1), 80.5 (C-2/3), 76.5 (C-2/3), 75.21 (PhCH_2), 75.19 (C-4), 73.3 (PhCH_2), 72.19 (PhCH_2), 72.15 (C-5), 72.07 (PhCH_2), 69.0 (C-6), 25.4 (SCH_2CH_3), 21.3 (CH_3), 15.1 (SCH_2CH_3). ESI-HRMS for $C_{37}H_{42}O_5SNa^+$ $(M+Na)^+$ calculated: 621.2645; found: 621.2648.

Ethyl 2,3-di-*O*-isopropylidene-6-*O*-triisopropylsilyl-1-thio-α-D-mannopyranoside S10

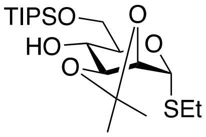

Based on the literature procedure,[9] a solution of **S6** (4.00 g, 10.5 mmol) in acetone (11 mL) was treated with 2,2-dimethoxypropane (52 mL, 0.42 mol) followed by TsOH.H_2O (400 mg, 2.10 mmol). After stirring the reaction mixture for 18 h at room temperature, the reaction was quenched with saturated NaHCO3 and the product was extracted with Et_2O (3 x 150 mL). The combined organic layers were dried over anhydrous $MgSO_4$ and concentrated *in vacuo* to give a yellow oil. Purification by column chromatography (90:10 to 80:20; Pentane/Et_2O) gave **S10** as a colourless syrup (3.7 g, 84% yield). ^{1}H NMR (500 MHz, Chloroform-*d*): δ 5.53 (s, 1H, H-1), 4.16 (dd, *J* = 5.6, 1.0 Hz, 1H, H-2), 4.11 (dd, *J* = 7.3, 5.7 Hz, 1H, H-3), 3.99 – 3.89 (m, 3H, H-6a, H-6b, H-5), 3.80 (ddd, *J* = 9.4, 7.3, 2.3 Hz, 1H, H-4), 3.09 (d, *J* = 2.4 Hz, 1H, OH), 2.69 (dq, *J* = 13.0, 7.3 Hz, 1H, SC*H*HCH_3), 2.54 (dq, *J* = 13.0, 7.5 Hz, 1H, SC*H*HCH_3), 1.54 (s, 3H, O2C(C*H*3)2), 1.35 (s, 3H, O2C(C*H*3)2), 1.29 (t, *J* = 7.4 Hz, 3H, SCH_2C*H*$_3$), 1.18 – 1.09 (m, 3H, SiC*H*(CH_3)$_2$), 1.08 (s, 12H, SiCH(C*H*$_3$)$_2$), 1.07 (s, 6H, SiCH(C*H*$_3$)$_2$). ^{13}C NMR (126 MHz, Chloroform-*d*): δ 109.7 (O2*C*(CH3)2), 79.5 (C-1), 78.3 (C-3), 76.3 (C-2), 72.9 (C-4), 69.1 (C-5), 65.3 (C-6), 28.3 (O2C(*C*H3)2), 26.5 (O2C(*C*H3)2), 24.3 (S*C*H_2CH_3), 18.0 (SiCH(*C*H_3)$_2$), 14.6 (SCH_2*C*H_3), 11.9 (Si*C*H(CH_3)$_2$). ESI-HRMS for $C_{20}H_{40}O_5SSiNa^+$ $(M+Na)^+$ calculated: 443.2258; found: 443.2258.

Ethyl 2,3-di-*O*-isopropylidene-4-*O*-(4-methylbenzyl)-6-*O*-triisopropylsilyl-1-thio-α-D-mannopyranoside S11

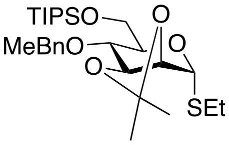

Based on the literature procedure,[7] under a N_2 atmosphere, a solution of mannopyranoside **S10** (1.1 g, 2.6 mmol) in anhydrous DMF (5 mL) was cooled to 0 °C and NaH (60% dispersion in mineral oil) (208 mg, 5.21 mmol) was added. The reaction mixture was stirred at room temperature for 30 min after which it was again cooled down to 0 °C. 4-Methylbenzyl bromide (962 mg, 5.20 mmol) was added and the reaction mixture was left to stir at room temperature for 2 h. The reaction was quenched with MeOH and diluted with Et_2O (100 mL). The aqueous layer was washed with Et_2O (2 x 75 mL). The organic

layers were combined and neutralised with 1 M HCl. The organic layer was washed with water and brine, dried over anhydrous $MgSO_4$ and concentrated *in vacuo* to give a yellow oil. Purification by column chromatography (98:2 to 95:5; Pentane/Et_2O) gave **S11** as a clear yellowish syrup (1.3 g, 93% yield). ^{1}H NMR (500 MHz, Chloroform-*d*): δ 7.24 – 7.18 (m, 2H, ArCH), 7.13 (d, *J* = 7.8 Hz, 2H, ArCH), 5.54 (s, 1H, H-1), 4.84 (d, *J* = 11.3 Hz, 1H, C*H*HPh), 4.57 (d, *J* = 11.3 Hz, 1H, C*H*HPh), 4.29 (dd, *J* = 7.2, 5.7 Hz, 1H, H-3), 4.15 (dd, *J* = 5.7, 0.9 Hz, 1H, H-2), 3.96 (qd, *J* = 5.6, 1.9 Hz, 1H, H-5), 3.94 (dd, *J* = 11.1, 1.8 Hz, 1H, H-6a), 3.80 (dd, *J* = 11.0, 5.7 Hz, 1H, H-6b), 3.57 (dd, *J* = 10.2, 7.2 Hz, 1H, H-4), 2.71 (dq, *J* = 12.9, 7.3 Hz, 1H, SC*H*HCH$_3$), 2.51 (dq, *J* = 12.9, 7.5 Hz, 1H, SC*H*HCH$_3$), 2.33 (s, 3H, CH$_3$), 1.52 (s, 3H, O2C(C*H*3)2), 1.36 (s, 3H, O2C(C*H*3)2), 1.26 (t, *J* = 7.4 Hz, 3H, SCH$_2$C*H*$_3$), 1.13 – 1.02 (m, 21H, SiC*H*(C*H*$_3$)$_2$). ^{13}C NMR (126 MHz, Chloroform-*d*): δ 137.4 (C), 135.5 (C), 129.1 (CH), 128.2 (CH), 109.4 (O2*C*(CH3)2), 79.0 (C-3), 78.9 (C-1), 76.8 (C-2), 76.1 (C-4), 73.1 (Ph*C*H$_2$), 70.7 (C-5), 63.2 (C-6), 28.2 (O2C(*C*H3)2), 26.6 (O2C(*C*H3)2), 23.8 (S*C*H$_2$CH$_3$), 21.3 (CH$_3$), 18.1 (SiCH(*C*H$_3$)$_2$), 14.4 (SCH$_2$*C*H$_3$), 12.1 (Si*C*H(CH$_3$)$_2$). ESI-HRMS for $C_{28}H_{48}O_5SSiNa^+$ $(M+Na)^+$ calculated: 547.2884; found: 547.2884.

2,3,4,6-Tetra-*O*-benzyl-α/β-D-mannopyranose 1a

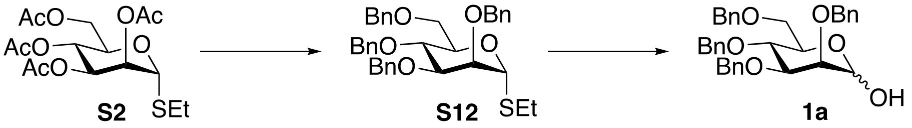

A solution of **S2** (2.8 g, 7.1 mmol) in methanol (30 mL) was treated with Na_2CO_3 (222 mg, 2.09 mmol) and stirred at room temperature for 14 h. The reaction mixture was neutralised with resin IR-120, filtered and concentrated *in vacuo* to give a brown syrup. Under a N_2 atmosphere, a solution of the syrup in anhydrous DMF (18 mL) was cooled to 0 °C and NaH (60% dispersion in mineral oil) (1.6 g, 39 mmol) was added. The reaction mixture was stirred at room temperature for 30 min after which it was again cooled down to 0 °C. Benzyl bromide (4.7 mL, 39 mmol) was added dropwise and the reaction mixture was left to stir at room temperature for 4 h. The reaction was quenched with MeOH and the mixture was concentrated *in vacuo* to give a yellow slurry. The slurry was diluted with CH_2Cl_2 and washed with 1 M HCl, followed by saturated $NaHCO_3$ and brine, dried over anhydrous $MgSO_4$ and concentrated *in vacuo*. Purification by column chromatography (90:10 to 80:20; Pentane/Et_2O) gave **S12** as a clear yellow oil (3.8 g, 90% yield, α only). ^{1}H NMR (400 MHz, Chloroform-*d*): δ 7.41 – 7.22 (m, 18H, ArCH), 7.21 – 7.11 (m, 2H, ArCH), 5.40 (d, *J* = 1.4 Hz, 1H, H-1), 4.88 (d, *J* = 10.8 Hz, 1H, C*H*HPh), 4.73 (d, *J* = 12.4 Hz, 1H, C*H*HPh), 4.66 (d, *J* = 12.4 Hz, 2H, 2 x C*H*HPh), 4.59 (d, *J* = 11.9 Hz, 1H, C*H*HPh), 4.55 (d, *J* = 11.9 Hz, 1H, C*H*HPh), 4.53 – 4.46 (m, 2H, 2 x C*H*HPh), 4.13 (ddd, *J* = 9.8, 4.8, 1.9 Hz, 1H, H-5), 4.06 – 3.99 (m, 1H, H-4), 3.87 – 3.78 (m, 3H, H-2, H-3, H-6a), 3.71 (dd, *J* = 10.8, 1.9 Hz, 1H, H-6b),

2.70 – 2.48 (m, 2H, SCH_2CH$_3$), 1.24 (t, J = 7.4 Hz, 3H, SCH$_2$CH_3). ^{13}C NMR (126 MHz, Chloroform-*d*): δ 138.7 (C), 138.5 (C), 138.4 (C), 138.3 (C), 128.49 (CH), 128.48 (CH), 128.42 (CH), 128.39 (CH), 128.1 (CH), 128.0 (CH), 127.93 (CH), 127.85 (CH), 127.8 (CH), 127.7 (CH), 127.6 (CH), 82.0 (C-1), 80.5 (C-), 76.5 (C-), 75.24 (Ph*C*H), 75.20 (C-4), 73.4 (Ph*C*H), 72.2 (Ph*C*H), 72.14 (C-5), 72.09 (Ph*C*H), 69.3 (C-6), 25.5 (S*C*H$_2$CH$_3$), 15.1 (SCH$_2$*C*H$_3$). NMR data were consistent with literature data.[3]

S12 was dissolved in 9:1 acetone/water (30 mL) and treated with NBS (3.5 g, 20 mmol) at room temperature. After 5 h the reaction was quenched with saturated $Na_2S_2O_3$ and diluted with CH_2Cl_2. The aqueous layer was washed with CH_2Cl_2. The combined organic layers were washed with water and brine, dried over anhydrous $MgSO_4$, filtered and concentrated *in vacuo* to give a yellow syrup. Purification by column chromatography (2:1 to 1:1; Pentane/Et_2O) afforded the hydrolysed product **1a** as a colourless syrup (3.1 g, 89% yield, α/β = 90:10). ^{1}H NMR (500 MHz, Chloroform-*d*): δ 7.40 – 7.20 (m, 18H, ArCH), 7.20 – 7.11 (m, 2H, ArCH), 5.24 (dd, J = 3.4, 1.9 Hz, 1H, H-1), 4.87 (d, J = 10.9 Hz, 1H, C*H*HPh), 4.74 (d, J = 12.4 Hz, 1H, C*H*HPh), 4.70 (d, J = 12.5 Hz, 1H, C*H*HPh), 4.61 (s, 2H, 2 x C*H*HPh), 4.58 (d, J = 12.2 Hz, 1H, C*H*HPh), 4.52 (d, J = 12.2 Hz, 1H, C*H*HPh), 4.49 (d, J = 10.9 Hz, 1H, C*H*HPh), 4.03 (ddd, J = 9.9, 6.4, 2.1 Hz, 1H, H-5), 3.95 (dd, J = 9.3, 3.0 Hz, 1H, H-3), 3.85 (t, J = 9.6 Hz, 1H, H-4), 3.79 (dd, J = 3.0, 1.9 Hz, 1H, H-2), 3.71 (dd, J = 10.5, 2.1 Hz, 1H, H-6a), 3.66 (dd, J = 10.5, 6.4 Hz, 1H, H-6b), 3.23 (d, J = 3.4 Hz, 1H, OH).

2,3,4-Tri-*O*-benzyl-6-*O*-(4-methylbenzyl)-α/β-D-mannopyranose 1b

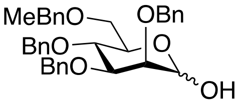

A solution of **S9** (829 mg, 1.38 mmol) in 9:1 acetone/water (14 mL) and treated with NBS (737 mg, 4.14 mmol) at room temperature. After 2 h the reaction was quenched with saturated $Na_2S_2O_3$ and diluted with CH_2Cl_2. The aqueous layer was washed with CH_2Cl_2. The combined organic layers were washed with water and brine, dried over anhydrous $MgSO_4$, filtered and concentrated *in vacuo* to give a colourless syrup. Purification by column chromatography (100:0 to 90:10; CH_2Cl_2/Et_2O) afforded the hydrolysed product **1b** as a white solid (681 mg, 89% yield, α/β = 80:20). ESI-HRMS for $C_{35}H_{38}O_6Na^+$ $(M+Na)^+$ calculated: 577.2561; found: 577.2559.

The following were observed for α/β anomers:

^{1}H NMR (600 MHz, Chloroform-*d*): δ 7.39 – 7.05 (m, 19H, ArCH), 4.60 (s, 2H, 2 x C*H*HPh), 2.31 (s, 3H, CH_3).

α-anomer

^{1}H NMR (600 MHz, Chloroform-*d*): δ 5.24 (dd, *J* = 3.4, 1.9 Hz, 1H, OH), 4.86 (d, *J* = 10.9 Hz, 1H, C*H*HPh), 4.73 (d, *J* = 12.5 Hz, 1H, C*H*HPh), 4.70 (d, *J* = 12.4 Hz, 1H, C*H*HPh), 4.54 (d, *J* = 12.0 Hz, 1H, C*H*HPh), 4.48 (d, *J* = 12.0 Hz, 1H, C*H*HPh), 4.47 (d, *J* = 10.9 Hz, 1H, C*H*HPh), 4.02 (ddd, *J* = 9.9, 6.5, 2.1 Hz, 1H, H-5), 3.95 (dd, *J* = 9.3, 3.1 Hz, 1H, H-3), 3.87 – 3.81 (m, 1H, H-4), 3.78 (dd, *J* = 3.1, 1.9 Hz, 1H, H-2), 3.69 (dd, *J* = 10.4, 2.1 Hz, 1H, H-6a), 3.64 (dd, *J* = 10.5, 6.5 Hz, 1H, H-6b), 3.35 (d, *J* = 3.4 Hz, 1H, OH). ^{13}C NMR (151 MHz, Chloroform-*d*): δ 138.6 (C), 138.54 (C), 138.50 (C), 137.39 (C), 135.1 (C), 129.14 (CH), 128.47 (CH), 128.46 (CH), 128.4 (CH), 128.30 (CH), 128.11 (CH), 127.98 (CH), 127.76 (CH), 127.72 (CH), 127.70 (CH), 127.67 (CH), 92.9 (C-1), 79.9 (C-3), 75.38 (C-4), 75.2 (Ph*C*H$_2$), 75.0 (C-2), 73.3 (Ph*C*H$_2$), 72.8 (Ph*C*H$_2$), 72.3 (Ph*C*H$_2$), 71.7 (C-5), 69.5 (C-6), 21.31 (CH$_3$).

β-anomer

^{1}H NMR (600 MHz, Chloroform-*d*): δ 5.07 (d, *J* = 11.7 Hz, 1H, C*H*HPh), 4.83 (d, *J* = 10.7 Hz, 1H, C*H*HPh), 4.76 – 4.67 (m, 3H, 3 x C*H*HPh), 4.64 (dd, *J* = 11.5, 1.4 Hz, 1H, H-1), 4.51 (d, *J* = 10.7 Hz, 1H, C*H*HPh), 3.94 – 3.90 (m, 1H, H-4), 3.85 – 3.80 (m, 2H, H-2, OH), 3.71 (d, *J* = 3.5 Hz, 2H, H-6a,H-6b), 3.58 (dd, *J* = 9.4, 2.8 Hz, 1H, H-3), 3.43 (dt, *J* = 9.5, 3.5 Hz, 1H, H-5). ^{13}C NMR (151 MHz, Chloroform-*d*): δ 138.4 (C), 138.3 (C), 138.2 (C), 137.37 (C), 135.2 (C), 129.13 (CH), 128.7 (CH), 128.6 (CH), 128.45 (CH), 128.33 (CH), 128.32 (CH), 128.12 (CH), 128.06 (CH), 127.96 (CH), 127.80 (CH), 93.9 (C-1), 83.2 (C-3), 76.2 (C-2), 75.35 (C-5), 75.1 (Ph*C*H$_2$), 74.8 (Ph*C*H$_2$), 74.7 (C-4), 73.5 (Ph*C*H$_2$), 73.0 (Ph*C*H$_2$), 68.9 (C-6), 21.30 (CH$_3$).

2,3,6-Tri-*O*-benzyl-4-*O*-(4-methylbenzyl)-α/β-D-mannopyranose 1c

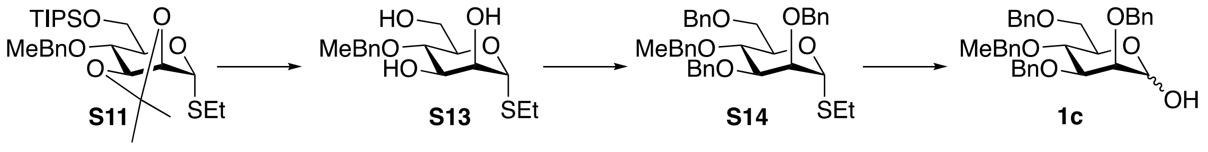

Based on the literature procedure,[8] a solution of **S11** (1.3 g, 2.5 mmol) in MeCN/H$_2$O (4:1, 13 mL) was treated with trifluoroacetic acid (2.0 mL, 26 mmol) at room temperature. After stirring for 7 h at room temperature, the reaction was quenched with saturated NaHCO$_3$ and diluted with CH$_2$Cl$_2$ (150 mL). The aqueous layer was washed with CH$_2$Cl$_2$ (2 x 100 mL). The combined organic layers were washed with brine, dried over anhydrous MgSO$_4$ and concentrated *in vacuo* to give a bright yellow oil. The crude material was used in the next step without further purification.

Under a N$_2$ atmosphere, a solution of crude mannopyranoside **S13** in anhydrous DMF (5 mL) was cooled to 0 °C and NaH (60% dispersion in mineral oil) (462 mg, 11.3 mmol) was added. The reaction mixture

was stirred at room temperature for 15 min after which it was again cooled down to 0 °C. Benzyl bromide (1.3 mL, 11 mmol) was added and the reaction mixture was left to stir at room temperature for 3 h. The reaction was quenched with MeOH and diluted with Et_2O (100 mL). The aqueous layer was washed with Et_2O (2 x 75 mL). The organic layers were combined and dried over anhydrous $MgSO_4$ and concentrated *in vacuo* to give a yellow oil.

A solution of crude **S14** in 9:1 acetone/water (13 mL) and treated with NBS (1.3 g, 7.5 mmol) at room temperature. After 2.5 h the reaction was quenched with saturated $Na_2S_2O_3$ and diluted with CH_2Cl_2. The aqueous layer was washed with CH_2Cl_2 (2 x 100 mL). The combined organic layers were washed with water and brine, dried over anhydrous $MgSO_4$, filtered and concentrated *in vacuo* to give a yellow syrup. Purification by column chromatography (100:0 to 90:10; CH_2Cl_2/Et_2O) afforded the hydrolysed product **1c** as a yellowish syrup (1.1 g, 79% yield over 3 steps, α/β = 85:15). ESI-HRMS for $C_{35}H_{38}O_6Na^+$ $(M+Na)^+$ calculated: 577.2561; found: 577.2565.

The following were observed for α/β anomers:

^{1}H NMR (500 MHz, Chloroform-*d*): δ 7.39 – 7.23 (m, 15H, ArCH), 7.11 – 6.96 (m, 4H, ArCH), 2.32 (s, 3H, CH_3). ^{13}C NMR (126 MHz, Chloroform-*d*): δ 75.1 (Ph*C*H_2), 21.3 (CH_3).

α-anomer

^{1}H NMR (500 MHz, Chloroform-*d*): δ, 5.25 (dd, *J* = 3.4, 1.9 Hz, 1H, H-1), 4.83 (d, *J* = 10.6 Hz, 1H, C*H*HPh), 4.75 (d, *J* = 12.6 Hz, 1H, C*H*HPh), 4.70 (d, *J* = 12.6 Hz, 1H, C*H*HPh), 4.64 (d, *J* = 11.9 Hz, 1H, C*H*HPh), 4.60 (d, *J* = 11.9 Hz, 1H, C*H*HPh), 4.58 (d, *J* = 12.3 Hz, 1H, C*H*HPh), 4.53 (d, *J* = 12.3 Hz, 1H, C*H*HPh), 4.45 (d, *J* = 10.6 Hz, 1H, C*H*HPh), 4.01 (ddd, *J* = 9.8, 6.2, 2.1 Hz, 1H, H-5), 3.94 (dd, *J* = 9.3, 3.0 Hz, 1H, H-3), 3.85 (t, *J* = 9.6 Hz, 1H, H-4), 3.79 (dd, *J* = 3.0, 2.0 Hz, 1H, H-2), 3.71 (dd, *J* = 10.5, 2.2 Hz, 1H, H-6a), 3.66 (dd, *J* = 10.5, 6.3 Hz, 1H, H-6b), 2.92 (d, *J* = 3.3 Hz, 1H, OH). ^{13}C NMR (126 MHz, Chloroform-*d*): δ 138.7 (C), 138.5 (C), 138.2 (C), 137.4 (C), 135.5 (C), 129.1 (CH), 128.48 (CH), 128.45 (CH), 128.28 (CH), 128.09 (CH), 127.97 (CH), 127.8 (CH), 127.71 (CH), 127.66 (CH), 92.9 (C-1), 79.9 (C-3), 75.2 (C-4), 75.0 (C-2), 73.5 (Ph*C*H_2), 72.8 (Ph*C*H_2), 72.4 (Ph*C*H_2), 71.8 (C-5), 69.8 (C-6).

β-anomer

^{1}H NMR (500 MHz, Chloroform-*d*): δ 5.09 (d, *J* = 11.8 Hz, 1H, C*H*HPh), 4.80 (d, *J* = 10.7 Hz, 1H, C*H*HPh), 4.74 – 4.66 (m, 3H, 3 x C*H*HPh), 4.66 – 4.60 (m, 3H, H-1, 2 x C*H*HPh), 4.50 (d, *J* = 10.9 Hz, 1H, C*H*HPh), 3.92 (t, *J* = 9.4 Hz, 1H, H-4), 3.84 – 3.82 (m, 1H, H-2), 3.80 – 3.74 (m, 1H, OH), 3.74 – 3.70 (m, 2H, H-6a, H-6b), 3.59 (dd, *J* = 9.4, 2.8 Hz, 1H, H-3), 3.43 (ddd, *J* = 9.5, 4.2, 2.9 Hz, 1H, H-5). ^{13}C NMR (126 MHz, Chloroform-*d*): δ 138.34 (C), 138.29 (C), 137.6 (C), 135.3 (C), 129.2 (CH), 128.7 (CH), 128.6 (CH), 128.31 (CH), 128.05 (CH), 127.95 (CH), 127.1 (CH), 93.8 (C-1), 83.2 (C-3), 76.3 (C-2), 75.4 (C-5), 74.8 (Ph*C*H_2), 74.6 (C-4), 73.7 (Ph*C*H_2), 73.0 (Ph*C*H_2), 69.2 (C-6).

2,3,4-Tri-*O*-benzyl-6-*O*-(4-methoxybenzyl)-α-D-mannopyranose 1d

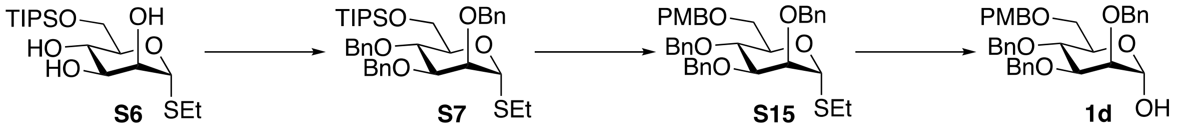

Under a N_2 atmosphere, a solution of mannopyranoside **S6** (0.50 g, 1.3 mmol) in anhydrous DMF (2.6 mL) was cooled to 0 °C and NaH (60% dispersion in mineral oil) (0.21 g, 5.2 mmol) was added followed by benzyl bromide (0.62 mL, 5.2 mmol). After stirring the reaction mixture for 11 h, it was quenched with MeOH and diluted with Et_2O (40 mL). The aqueous layer was washed with Et_2O (2 x 40 mL). The organic layers were combined and neutralised with 1 M HCl. The organic layer was washed with water and brine, dried over anhydrous $MgSO_4$ and concentrated *in vacuo* to give a yellow oil.

Based on the literature procedure,[8] a solution of crude **S7** in MeCN/H_2O (4:1, 6.5 mL) was treated with trifluoroacetic acid (0.74 mL, 10 mmol) at room temperature. After stirring for 8 h at room temperature, the reaction was quenched with saturated $NaHCO_3$ and diluted with CH_2Cl_2 (50 mL). The aqueous layer was washed with CH_2Cl_2 (2 x 50 mL). The combined organic layers were washed with brine, dried over anhydrous $MgSO_4$ and concentrated *in vacuo* to give a yellow syrup. The crude material was used in the next step without further purification. Under a N_2 atmosphere, a solution of the crude mannopyranoside in anhydrous DMF (2.5 mL) was cooled to 0 °C and NaH (60% dispersion in mineral oil) (130 mg, 3.25 mmol). After 15 minutes, 4-methoxylbenzyl bromide (0.45 mL, 3.3 mmol) was added and the reaction mixture was left to stir at room temperature for 2 h. The reaction was quenched with MeOH and diluted with Et_2O (100 mL). The aqueous layer was washed with Et_2O (2 x 40 mL). The organic layers were combined and neutralised with 1 M HCl. The organic layer was washed with water and brine, dried over anhydrous $MgSO_4$ and concentrated *in vacuo* to give a yellow oil.

A solution of **S15** in 9:1 acetone/water (13 mL) and treated with NBS (0.69 g, 3.9 mmol) at room temperature. After 2 h the reaction was quenched with saturated $Na_2S_2O_3$ and diluted with CH_2Cl_2. The aqueous layer was washed with CH_2Cl_2. The combined organic layers were washed with water and brine, dried over anhydrous $MgSO_4$, filtered and concentrated *in vacuo* to give a colourless syrup. Purification by column chromatography (96:4; CH_2Cl_2/Et_2O) afforded the hydrolysed product along with an aromatic impurity. Trituration from Pentane/Et_2O (90:10) afforded pure **1d** as a white solid (471 mg, 63% yield over 4 steps, α-only). ^{1}H NMR (600 MHz, Chloroform-*d*): δ 7.49 – 7.03 (m, 17H, ArCH), 6.82 (d, *J* = 8.1 Hz, 2H, ArCH), 5.30 – 5.13 (m, 1H, H-1), 4.86 (d, *J* = 10.9 Hz, 1H, C*H*HPh), 4.74 (d, *J* = 12.5 Hz, 1H, C*H*HPh), 4.70 (d, *J* = 12.6 Hz, 1H, C*H*HPh), 4.60 (s, 2H, 2 x C*H*HPh), 4.54 – 4.39 (m, 3H, 3 x C*H*HPh), 4.02 (br t, *J* = 8.1 Hz, 1H, H-5), 3.94 (dd, *J* = 9.4, 3.1 Hz, 1H, H-3), 3.83 (t, *J* = 9.5

Hz, 1H, H-4), 3.78 (s, 1H, H-2), 3.75 (s, 3H, OCH_3), 3.68 (d, *J* = 10.4 Hz, 1H, H-6a), 3.63 (dd, *J* = 10.4, 6.7 Hz, 1H, H-6b), 3.39 (d, *J* = 3.4 Hz, 1H, OH). ^{13}C NMR (151 MHz, Chloroform-*d*): δ 159.3 (C), 138.6 (C), 138.54 (C), 138.51 (C), 130.2 (C), 129.8 (CH), 128.48 (CH), 128.46 (CH), 128.4 (CH), 128.1 (CH), 127.98 (CH), 127.76 (CH), 127.73 (CH), 127.72 (CH), 127.67 (CH), 113.9 (CH), 92.9 (C-1), 79.9 (C-3), 75.4 (C-4), 75.2 (Ph*C*H_2), 75.0 (C-2), 73.0 (Ph*C*H_2), 72.8 (Ph*C*H_2), 72.3 (Ph*C*H_2), 71.6 (C-5), 69.3 (C-6), 55.3 (OCH_3). NMR data were consistent with literature data.[10]

2,3,4-Tri-*O*-benzyl-6-*O*-(2-naphthyl)-α/β-D-mannopyranose 1e

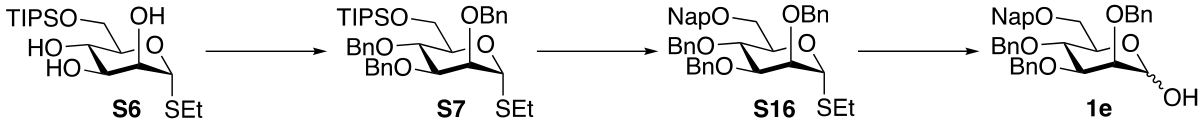

Under a N_2 atmosphere, a solution of mannopyranoside **S6** (0.50 g, 1.3 mmol) in anhydrous DMF (2.6 mL) was cooled to 0 °C and NaH (60% dispersion in mineral oil) (0.21 g, 5.2 mmol) was added followed by benzyl bromide (0.62 mL, 5.2 mmol). After stirring the reaction mixture for 11 h, it was quenched with MeOH and diluted with Et_2O (40 mL). The aqueous layer was washed with Et_2O (2 x 40 mL). The organic layers were combined and neutralised with 1 M HCl. The organic layer was washed with water and brine, dried over anhydrous $MgSO_4$ and concentrated *in vacuo* to give a yellow oil.

Based on the literature procedure,[8] a solution of crude **S7** in MeCN/H_2O (4:1, 6.5 mL) was treated with trifluoroacetic acid (0.74 mL, 10 mmol) at room temperature. After stirring for 8 h at room temperature, the reaction was quenched with saturated $NaHCO_3$ and diluted with CH_2Cl_2 (50 mL). The aqueous layer was washed with CH_2Cl_2 (2 x 50 mL). The combined organic layers were washed with brine, dried over anhydrous $MgSO_4$ and concentrated *in vacuo* to give a yellow syrup. The crude material was used in the next step without further purification. Under a N_2 atmosphere, a solution of the crude mannopyranoside in anhydrous DMF (2.5 mL) was cooled to 0 °C and NaH (60% dispersion in mineral oil) (130 mg, 3.25 mmol). After 5 minutes, 2-(bromomethyl)naphthalene (719 mg, 3.25 mmol) was added and the reaction mixture was left to stir at room temperature for 1 h. The reaction was quenched with MeOH and diluted with Et_2O (100 mL). The aqueous layer was washed with Et_2O (2 x 40 mL). The organic layers were combined and neutralised with 1 M HCl. The organic layer was washed with water and brine, dried over anhydrous $MgSO_4$ and concentrated *in vacuo* to give a yellow oil.

A solution of **S16** in 9:1 acetone/water (13 mL) and treated with NBS (0.69 g, 3.9 mmol) at room temperature. After 2 h the reaction was quenched with saturated $Na_2S_2O_3$ and diluted with CH_2Cl_2. The aqueous layer was washed with CH_2Cl_2. The combined organic layers were washed with water and

brine, dried over anhydrous $MgSO_4$, filtered and concentrated *in vacuo* to give a colourless syrup. Purification by column chromatography (1:1 to 1:2; Pentane/Et_2O) afforded the hydrolysed product along with an aromatic impurity. Trituration from Pentane/Et_2O (90:10) afforded pure **1e** as a white solid (523 mg, 68% yield over 4 steps, α/β = 87:13). ESI-HRMS for $C_{38}H_{38}O_6Na^+$ $(M+Na)^+$ calculated: 613.2561; found: 613.2559.

The following were observed for α/β anomers:

^{1}H NMR (600 MHz, Chloroform-*d*): δ 7.82 – 7.72 (m, 4H, ArCH), 7.49 – 7.42 (m, 3H, ArCH), 7.39 – 7.22 (m, 10H, ArCH), 7.21 – 7.08 (m, 3H, ArCH), 7.05 (d, *J* = 7.1 Hz, 2H, ArCH), 4.76 – 4.67 (m, 4H, 4 x C*H*HPh).

α-anomer

^{1}H NMR (600 MHz, Chloroform-*d*): δ 5.26 (dd, *J* = 3.4, 1.9 Hz, 1H, H-1), 4.85 (d, *J* = 10.9 Hz, 1H, C*H*HPh), 4.57 (s, 2H, 2 x C*H*HPh), 4.45 (d, *J* = 10.9 Hz, 1H, C*H*HPh), 4.07 (ddd, *J* = 9.1, 6.6, 2.0 Hz, 1H, H-5), 3.94 (dd, *J* = 9.4, 2.9 Hz, 1H, H-3), 3.85 (t, *J* = 9.6 Hz, 1H, H-4), 3.78 (t, *J* = 2.5 Hz, 1H, H-2), 3.70 (dd, *J* = 10.5, 2.0 Hz, 1H, H-6a), 3.70 (dd, *J* = 10.5, 6.6 Hz, 1H, H-6b), 3.33 (d, *J* = 3.4 Hz, 1H, OH). ^{13}C NMR (151 MHz, Chloroform-*d*): δ 138.6 (C), 138.5 (C), 138.4 (C), 135.6 (C), 133.37 (C), 133.15 (C), 128.5 (CH), 128.37 (CH), 128.30 (CH), 128.1 (CH), 128.04 (CH), 127.98 (CH), 127.83 (CH), 127.77 (CH), 127.74 (CH), 127.68 (CH), 127.67 (CH), 126.9 (CH), 126.18 (CH), 126.0 (CH), 92.9 (C-1), 79.9 (C-3), 75.4 (C-4), 75.18 (Ph*C*H_2), 75.0 (C-2), 73.6 (Ph*C*H_2), 72.8 (Ph*C*H_2), 72.3 (Ph*C*H_2), 71.7 (C-5), 69.8 (C-6).

β-anomer

^{1}H NMR (600 MHz, Chloroform-*d*): δ 5.07 (d, *J* = 11.8 Hz, 1H, C*H*HPh), 4.82 (d, *J* = 10.7 Hz, 1H, C*H*HPh), 4.79 (d, *J* = 12.3 Hz, 1H, C*H*HPh), 4.65 (dd, *J* = 11.6, 1.4 Hz, 1H, H-1), 4.51 (d, *J* = 10.7 Hz, 1H, C*H*HPh), 3.96 – 3.91 (m, 1H, H-4), 3.82 (d, *J* = 11.4 Hz, 1H, OH), 3.81 (d, *J* = 2.2 Hz, 1H, H-2), 3.80 – 3.74 (m, 2H, H-6a, H-6b), 3.56 (dd, *J* = 9.4, 2.8 Hz, 1H, H-3), 3.46 (ddd, *J* = 9.6, 4.5, 2.8 Hz, 1H, H-5). ^{13}C NMR (151 MHz, Chloroform-*d*): δ 138.3 (C), 138.20 (C), 138.15 (C), 135.8 (C), 133.38 (C), 133.14 (C), 128.7 (CH), 128.6 (CH), 128.41 (CH), 128.26 (CH), 126.23 (CH), 125.9 (CH), 93.9 (C-1), 83.2 (C-3), 76.2 (C-2), 75.4 (C-5), 75.16 (Ph*C*H_2), 74.8 (Ph*C*H_2), 74.7 (C-4), 73.8 (Ph*C*H_2), 72.9 (Ph*C*H_2), 69.2 (C-6).

2,3,4-Tri-*O*-benzyl-6-*O*-*tert*-butyldiphenylsilyl-α/β-D-mannopyranose 1f

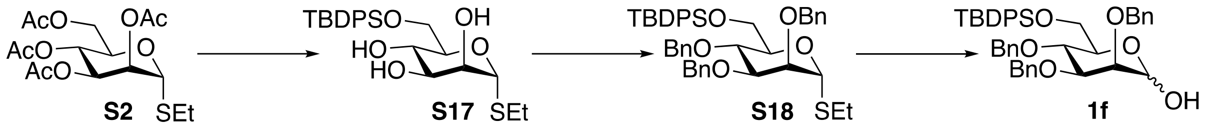

A solution of **S2** (500 mg, 1.27 mmol) in methanol (10 mL) was treated with Na_2CO_3 (40 mg, 0.38 mmol) and stirred at room temperature for 1.5 h. The reaction mixture was neutralised with resin IR-120, filtered and concentrated *in vacuo* to give a brown paste. Under a N_2 atmosphere, a solution of the crude material and imidazole (259 mg, 3.81 mmol) in anhydrous DMF (2.5 mL) was treated with TBDPSCl (0.50 mL, 1.9 mmol) and left to stir at room temperature for 2.5 h. The reaction mixture was diluted with Et_2O and washed with water and brine, dried over anhydrous $MgSO_4$ and concentrated *in vacuo* to give a yellow syrup.

Under a N_2 atmosphere, a solution of crude mannopyranoside **S17** in anhydrous DMF (2.5 mL) was cooled to 0 °C and NaH (60% dispersion in mineral oil) (228 mg, 5.72 mmol) was added. The reaction mixture was stirred at room temperature for 15 min after which it was again cooled down to 0 °C. Benzyl bromide (0.68 mL, 5.7 mmol) was added and the reaction mixture was left to stir at room temperature for 6 h. The reaction was quenched with MeOH and diluted with Et_2O (100 mL). The aqueous layer was washed with Et_2O (2 x 75 mL). The organic layers were combined and dried over anhydrous $MgSO_4$ and concentrated *in vacuo* to give a yellow oil.
A solution of crude **S18** in 9:1 acetone/water (13 mL) and treated with NBS (678 mg, 3.81 mmol) at room temperature. After 50 minutes the reaction was quenched with saturated $Na_2S_2O_3$ and diluted with CH_2Cl_2. The aqueous layer was washed with CH_2Cl_2 (2 x 100 mL). The combined organic layers were washed with water and brine, dried over anhydrous $MgSO_4$, filtered and concentrated *in vacuo* to give a yellow syrup. Purification by column chromatography (3:1 to 0:1; Pentane/Et_2O) afforded the hydrolysed product **1f** as a yellowish syrup (388 mg, 44% yield over 4 steps, α/β = 85:15).

The following were observed for α/β anomers:
^{1}H NMR (500 MHz, Chloroform-*d*): δ 7.77 – 7.72 (m, 2H, ArCH), 7.72 – 7.66 (m, 2H, ArCH), 7.41 – 7.22 (m, 19H, ArCH), 7.20 – 7.12 (m, 2H, ArCH). ^{13}C NMR (126 MHz, Chloroform-*d*): δ 135.8 (CH), 19.49 (Si*C*(CH_3)$_3$).
α-anomer
^{1}H NMR (500 MHz, Chloroform-*d*): δ 5.22 (dd, *J* = 3.3, 1.9 Hz, 1H, H-1), 4.92 (d, *J* = 10.8 Hz, 1H, C*H*HPh), 4.81 (d, *J* = 12.4 Hz, 1H, C*H*HPh), 4.69 – 4.64 (m, 3H, 3 x C*H*HPh), 4.61 (d, *J* = 10.9 Hz, 1H, C*H*HPh), 4.13 (t, *J* = 9.6 Hz, 1H, H-4), 4.01 (dd, *J* = 11.3, 4.6 Hz, 1H, H-6a), 3.97 (dd, *J* = 9.5, 3.1 Hz,

1H, H-3), 3.91 – 3.83 (m, 2H, H-6b, H-5), 3.79 (dd, *J* = 3.1, 1.9 Hz, 1H, H-2), 2.58 (d, *J* = 3.4 Hz, 1H, OH), 1.05 (s, 9H, SiC(C*H*3)3). ^{13}C NMR (126 MHz, Chloroform-*d*): δ 138.8 (C), 138.74 (C), 138.73 (C), 136.08 (CH), 134.1 (C), 133.6 (C), 129.66 (CH), 129.64 (CH), 128.49 (CH), 128.43 (CH), 128.0 (CH), 127.86 (CH), 127.76 (CH), 127.63 (CH), 127.60 (CH), 92.9 (C-1), 79.8 (C-3), 75.7 (C-2), 75.23 (Ph*C*H2), 74.85 (C-4), 73.3 (C-5), 72.9 (Ph*C*H2), 72.4 (Ph*C*H2), 63.6 (C-6), 26.98 (SiC(*C*H3)3). NMR data were consistent with literature data.[11]

β-anomer

^{1}H NMR (500 MHz, Chloroform-*d*) selected signals: δ 5.15 (d, *J* = 11.5 Hz, 1H, C*H*HPh), 4.91 (d, *J* = 10.8 Hz, 1H, C*H*HPh), 4.76 (s, 2H, 2 x C*H*HPh), 4.68 – 4.63 (m, 1H, H-1), 4.18 (t, *J* = 9.3 Hz, 1H, H-4), 3.95 – 3.91 (m, 1H, H-6a), 3.88 – 3.84 (m, 1H, H-2), 3.66 – 3.61 (m, 2H, OH, H-3), 3.33 (ddd, *J* = 9.2, 3.7, 2.1 Hz, 1H, H-5), 1.04 (s, 9H, SiC(C*H*3)3). ^{13}C NMR (126 MHz, Chloroform-*d*): δ 138.6 (C), 138.5 (C), 138.3 (C), 136.13 (CH), 134.0 (C), 133.4 (C), 128.7 (CH), 128.6 (CH), 128.51 (CH), 127.91 (CH), 127.82 (CH), 127.69 (CH), 93.6 (C-1), 83.1(C-3), 77.1 (C-2), 76.2 (C-5), 75.21 (Ph*C*H2), 74.94 (Ph*C*H2), 74.4 (C-4), 73.0 (Ph*C*H2), 63.1 (C-6), 26.95 (SiC(*C*H3)3).

2,3,6-Tri-*O*-benzyl-4-*O*-*tert*-butyldimethylsilyl-α/β-D-mannopyranose 1g

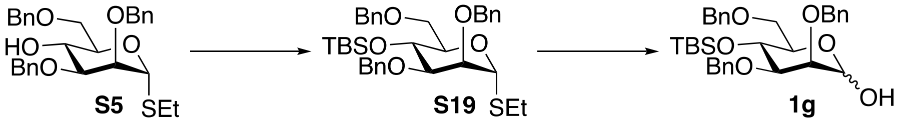

Under a N_2 atmosphere, a solution of **S5** (800 mg, 1.62 mmol), TBSCl (488 mg, 3.24 mmol), imidazole (441 mg, 6.48 mmol) and DMAP (20 mg, 0.16 mmol) in anhydrous DMF (2 mL) was stirred at room temperature for 15 h. The reaction mixture diluted with CH_2Cl_2 and washed with water and brine. The organic layer was dried over anhydrous $MgSO_4$, filtered and concentrated *in vacuo* to give a colourless oil. The crude material was used in the next step without further purification. ^{1}H NMR (500 MHz, Chloroform-*d*): δ 7.38 – 7.22 (m, 15H, ArCH), 5.39 (d, *J* = 1.8 Hz, 1H, H-1), 4.66 – 4.56 (m, 4H, 4 x C*H*HPh), 4.53 (s, 2H, 2 x C*H*HPh), 4.10 (ddd, *J* = 8.4, 6.1, 2.0 Hz, 1H, H-5), 4.07 – 4.00 (m, 1H, H-4), 3.81 – 3.75 (m, 2H, H-2, H-6a), 3.72 (dd, *J* = 10.7, 6.3 Hz, 1H, H-6b), 3.62 (dd, *J* = 8.6, 3.0 Hz, 1H, H-3), 2.73 – 2.54 (m, 2H, SC*H*2CH3), 1.28 (t, *J* = 7.4 Hz, 3H, SCH2C*H*3), 0.82 (s, 9H, SiC(C*H*3)3), 0.01 (s, 6H, 2 x SiCH3). ^{13}C NMR (126 MHz, Chloroform-*d*): δ 138.7 (C), 138.45 (C), 138.43 (C), 128.4 (CH), 128.353 (CH), 128.345 (CH), 127.9 (CH), 127.8 (CH), 127.7 (CH), 127.6 (CH), 127.5 (CH), 127.4 (CH), 81.92 (C-1), 80.6 (C-3), 76.4 (C-2), 73.6 (C-5), 73.2 (Ph*C*H2), 72.2 (Ph*C*H2), 71.7 (Ph*C*H2), 69.9 (C-6), 68.5 (C-4), 26.1 (SiC(*C*H3)3), 25.3 (S*C*H2CH3), 18.3 (Si*C*(CH3)3), 15.1 (SCH2*C*H3), -3.7 (SiCH3), -4.8 (SiCH3).

The crude material **S19** was dissolved in 9:1 acetone/water (10 mL) and treated with NBS (865 mg, 4.86 mmol) at room temperature. TLC analysis (2:1; Pentane/Et_2O) of the reaction after 2.5 h showed the hydrolysed product along with de-silylated hydrolysed product. The reaction was quenched with saturated $Na_2S_2O_3$ and diluted with CH_2Cl_2. The organic layer was washed with water and brine, dried over anhydrous $MgSO_4$, filtered and concentrated *in vacuo*. Purification by column chromatography (3:1 to 1:1, Pentane/Et_2O) afforded the hydrolysed product **1g** as a syrup (308 mg, 34% yield over 2 steps, α/β = 86:14). ESI-HRMS for $C_{33}H_{44}O_6SiNa^+$ $(M+Na)^+$ calculated: 587.2799; found: 587.2799.

The following were observed for α/β anomers:

^{1}H NMR (500 MHz, Chloroform-*d*): δ 7.39 – 7.19 (m, 15H, ArCH). ^{13}C NMR (126 MHz, Chloroform-*d*): 26.0 (SiC(CH_3)$_3$).

α-anomer

^{1}H NMR (500 MHz, Chloroform-*d*): δ 5.26 (t, *J* = 2.5 Hz, 1H, H-1), 4.69 (d, *J* = 12.3 Hz, 1H, C*H*HPh), 4.65 (d, *J* = 12.3 Hz, 1H, C*H*HPh), 4.64 (d, *J* = 12.3 Hz, 1H, C*H*HPh), 4.59 (d, *J* = 11.9 Hz, 1H, C*H*HPh), 4.55 (d, *J* = 12.0 Hz, 1H, C*H*HPh), 4.52 (d, *J* = 12.3 Hz, 1H, C*H*HPh), 4.01 (ddd, *J* = 9.7, 8.1, 2.0 Hz, 1H, H-5), 3.91 (t, *J* = 9.1 Hz, 1H, H-4), 3.79 – 3.75 (m, 2H, H-2, H-6a), 3.72 (dd, *J* = 8.8, 2.9 Hz, 1H, H-3), 3.58 (dd, *J* = 10.2, 8.0 Hz, 1H, H-6b), 3.48 (br s, 1H, OH), 0.79 (s, 9H, SiC(CH_3)$_3$), -0.01 (s, 3H, $SiCH_3$), -0.03 (s, 3H, $SiCH_3$). ^{13}C NMR (126 MHz, Chloroform-*d*): δ 138.63 (C), 138.59 (C), 138.2 (C), 128.47 (CH), 128.40 (CH), 128.3 (CH), 128.04 (CH), 127.8 (CH), 127.65 (CH), 127.63 (CH), 127.5 (CH), 93.0 (C-1), 79.9 (C-3), 74.8 (C-2), 73.4 (Ph*C*H_2), 73.0 (C-5), 72.9 (Ph*C*H_2), 71.8 (Ph*C*H_2), 70.3 (C-6), 68.7 (C-4), 18.22 (Si*C*(CH_3)$_3$), -3.7 ($SiCH_3$), -4.8 ($SiCH_3$).

β-anomer

^{1}H NMR (500 MHz, Chloroform-*d*) selected signals: δ 4.98 (d, *J* = 11.7 Hz, 1H, C*H*HPh), 4.77 – 4.72 (m, 1H, H-1), 3.97 (t, *J* = 8.7 Hz, 1H, H-4), 3.83 – 3.80 (m, 2H, H-2, H-6a), 3.62 (dd, *J* = 10.4, 6.7 Hz, 1H, H-6b), 3.47 (ddd, *J* = 8.9, 6.4, 2.5 Hz, 1H, H-5), 3.41 (dd, *J* = 8.7, 2.7 Hz, 1H, H-3), 0.81 (s, 9H, SiC(CH_3)$_3$), 0.02 (s, 6H, 2 x $SiCH_3$). ^{13}C NMR (126 MHz, Chloroform-*d*) selected signals: δ 128.6 (CH), 128.55 (CH), 128.42 (CH), 128.02 (CH), 127.9 (CH), 127.69 (CH), 93.8 (C-1), 83.2 (C-3), 76.9 (C-5), 75.6 (C-2), 74.5 (Ph*C*H_2), 68.0 (C-4), 18.19 (Si*C*(CH_3)$_3$), -3.8 ($SiCH_3$), -4.7 ($SiCH_3$).

6-*O*-Benzoyl-2,3,4-tri-*O*-benzyl-α/β-D-mannopyranose 1h

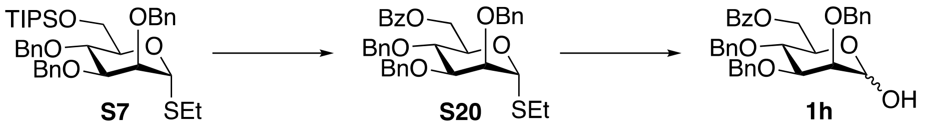

Based on the literature procedure,[8] a solution of **S7** (930 mg, 1.43 mmol) in MeCN/H_2O (4:1, 10 mL) was treated with trifluoroacetic acid (0.89 mL, 12 mmol) at room temperature. After stirring for 11 h at room temperature, the reaction was quenched with saturated $NaHCO_3$ and diluted with CH_2Cl_2 (100 mL). The aqueous layer was washed with CH_2Cl_2 (2 x 40 mL). The combined organic layers were washed with brine, dried over anhydrous $MgSO_4$ and concentrated *in vacuo* to give a colourless syrup. The crude material was used in the next step without further purification. Under a N_2 atmosphere, a solution of the crude material and DMAP (17 mg, 0.14 mmol) in anhydrous CH_2Cl_2 (2.9 mL) was treated with anhydrous pyridine (0.11 mL, 1.4 mmol) followed by benzoyl chloride (0.33 mL, 2.8 mmol) at room temperature. TLC (CH_2Cl_2) analysis of the reaction after 50 minutes showed complete consumption of starting material. The reaction was quenched with water and diluted with CH_2Cl_2. The organic layer was washed with 1 M HCl, saturated $NaHCO_3$ and brine. The organic layer was dried over anhydrous $MgSO_4$, filtered and concentrated *in vacuo* to give a colourless oil. ^{1}H NMR (500 MHz, Chloroform-*d*) selected signals: δ 5.39 (d, *J* = 1.6 Hz, 1H, H-1), 4.94 (d, *J* = 10.8 Hz, 1H, C*H*HPh), 4.70 (m, 2H, 2 x C*H*HPh), 4.63 (s, 2H, 2 x C*H*HPh), 4.60 (d, *J* = 10.8 Hz, 1H, C*H*HPh), 4.59 – 4.56 (m, 1H, H-6a), 4.54 (dd, *J* = 11.8, 2.4 Hz, 1H, H-6b), 4.29 (ddd, *J* = 9.8, 4.3, 2.4 Hz, 1H, H-5), 4.12 (t, *J* = 9.5 Hz, 1H, H-4), 3.91 (dd, *J* = 9.2, 3.1 Hz, 1H, H-3), 3.87 (dd, *J* = 3.1, 1.7 Hz, 1H, H-2), 2.71 – 2.47 (m, 2H, SCH_2CH$_3$), 1.24 (t, *J* = 7.4 Hz, 3H, SCH$_2$CH_3).

The crude material **S20** was dissolved in 9:1 acetone/water (14 mL) and treated with NBS (1.02 g, 5.72 mmol) at room temperature. After 3 h the reaction was quenched with saturated $Na_2S_2O_3$ and diluted with CH_2Cl_2. The aqueous layer was washed with CH_2Cl_2. The combined organic layers were washed with water and brine, dried over anhydrous $MgSO_4$, filtered and concentrated *in vacuo* to give a colourless syrup. Purification by column chromatography (2:1 to 1:1; Pentane/Et_2O) afforded the hydrolysed product **1h** as a colourless syrup (645 mg, 81% yield over 3 steps, α/β = 77:23).

The following were observed for α/β anomers:

^{1}H NMR (500 MHz, Chloroform-*d*): δ 8.05 – 7.97 (m, 2H, ArCH), 7.55 – 7.47 (m, 1H, ArCH), 7.42 – 7.20 (m, 17H, ArCH), 4.62 (d, *J* = 10.8 Hz, 1H, C*H*HPh). ^{13}C NMR (126 MHz, Chloroform-*d*): δ 75.4 (Ph*C*H$_2$), 63.9 (C-6).

α-anomer

^{1}H NMR (500 MHz, Chloroform-*d*): δ 5.27 (d, *J* = 2.0 Hz, 1H, H-1), 4.95 (d, *J* = 10.8 Hz, 1H, C*H*HPh), 4.77 (d, *J* = 12.2 Hz, 1H, C*H*HPh), 4.68 (s, 2H, 2 x C*H*HPh), 4.65 (d, *J* = 12.2 Hz, 1H, C*H*HPh), 4.58 (dd, *J* = 11.9, 1.6 Hz, 1H, H-6a), 4.52 (dd, *J* = 12.0, 3.4 Hz, 1H, H-6b), 4.16 – 4.10 (m, 2H, H-4, H-5), 4.06 – 4.01 (m, 1H, H-3), 3.83 (dd, *J* = 3.0, 2.0 Hz, 1H, H-2), 2.92 (br s, 1H, OH). ^{13}C NMR (126 MHz, Chloroform-*d*): δ 166.6 (C=O), 138.48 (C), 138.47 (C),138.2 (C), 133.1(CH), 129.9 (CH), 128.55 (CH), 128.53 (CH), 128.46 (CH), 128.44 (CH), 128.29 (CH), 127.88 (CH), 127.73 (CH), 92.8 (C-1), 79.8 (C-3), 75.3 (C-2), 74.6 (C-4), 72.9 (Ph*C*H$_2$), 72.4 (Ph*C*H$_2$), 70.6 (C-5).

β-anomer

^{1}H NMR (500 MHz, Chloroform-*d*): δ 5.14 (d, *J* = 11.5 Hz, 1H, C*H*HPh), 4.91 (d, *J* = 10.8 Hz, 1H, C*H*HPh), 4.78 (s, 2H, 2 x C*H*HPh), 4.72 (d, *J* = 1.3 Hz, 1H, H-1), 4.67 (d, *J* = 11.6 Hz, 1H, C*H*HPh), 4.59 (dd, *J* = 11.9, 2.3 Hz, 1H, H-6a), 4.49 (dd, *J* = 11.7, 4.5 Hz, 1H, H-6b), 4.06 – 4.01 (m, 1H, H-4), 3.89 (dd, *J* = 2.8, 1.4 Hz, 1H, H-2), 3.69 (dd, *J* = 9.3, 2.7 Hz, 1H, H-3), 3.63 (ddd, *J* = 9.5, 4.6, 2.3 Hz, 1H, H-5). ^{13}C NMR (126 MHz, Chloroform-*d*): δ 166.5 (C=O), 138.3 (C), 137.92 (C), 137.86 (C), 130.2 (CH), 128.73 (CH), 128.71 (CH), 128.59 (CH), 128.32 (CH), 128.12 (CH), 128.06 (CH), 127.95 (CH), 127.82 (CH), 127.69 (CH), 93.8 (C-1), 83.2 (C-3), 76.6 (C-2), 75.0 (Ph*C*H$_2$), 74.2 (C-4), 73.6 (C-5), 73.0 (Ph*C*H$_2$). NMR data were consistent with literature data.[12]

4-*O*-Acetyl-2,3,6-tri-*O*-benzyl-α/β-D-mannopyranose S22

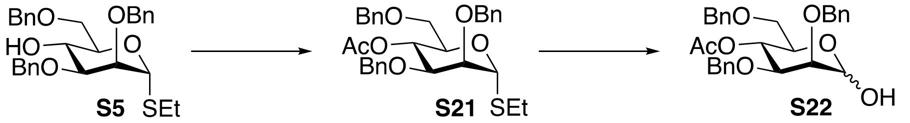

A solution of **S5** (800 mg, 1.62 mmol), acetic anhydride (0.31 mL, 1.6 mmol) and DMAP (20 mg, 0.16 mmol) in pyridine (0.13 mL, 1.6 mmol) (little bit of CH_2Cl_2 was added to get a clear solution) was stirred at room temperature for 2 h. The reaction mixture was diluted with CH_2Cl_2 and washed with 1 M HCl, saturated $NaHCO_3$ and brine. The organic layer was dried over anhydrous $MgSO_4$, filtered and concentrated *in vacuo* to give a yellowish syrup. The crude material was used in the next step without further purification. ^{1}H NMR (500 MHz, Chloroform-*d*): δ 7.44 – 7.14 (m, 15H, ArCH), 5.38 (t, *J* = 9.7 Hz, 1H, H-4), 5.37 (s, 1H, H-1), 4.69 (d, *J* = 12.4 Hz, 1H, C*H*HPh), 4.66 (d, *J* = 12.4 Hz, 1H, C*H*HPh), 4.54 (d, *J* = 11.9 Hz, 1H, C*H*HPh), 4.53 (d, *J* = 12.2 Hz, 1H, C*H*HPh), 4.51 (d, *J* = 12.0 Hz, 1H, C*H*HPh), 4.43 (d, *J* = 12.1 Hz, 1H, C*H*HPh), 4.23 – 4.16 (m, 1H, H-5), 3.82 (dd, *J* = 3.1, 1.8 Hz, 1H, H-2), 3.76 (dd, *J* = 9.5, 3.1 Hz, 1H, H-3), 3.62 (dd, *J* = 10.8, 6.1 Hz, 1H, H-6a), 3.55 (dd, *J* = 10.8, 3.1 Hz, 1H, H-6b), 2.70 – 2.52 (m, 2H, SC*H*$_2$CH$_3$), 1.92 (s, 3H, CH$_3$), 1.26 (t, *J* = 7.4 Hz, 3H, SCH$_2$C*H*$_3$). ^{13}C NMR (126 MHz, Chloroform-*d*): δ 17.0 (C=O), 138.3 (C), 138.2 (C), 138.1 (C), 128.49 (CH), 128.46 (CH),

128.4 (CH), 128.0 (CH), 127.81 (CH), 127.79 (CH), 127.75 (CH), 127.6 (CH), 82.14 (C-1), 77.4 (C-3), 76.0 (C-2), 73.5 (Ph*C*H_2), 72.4 (Ph*C*H_2), 71.9 (Ph*C*H_2), 70.7 (C-5), 69.9 (C-6), 69.2 (C-4), 25.4 (S*C*H_2CH_3), 21.1 (CH_3), 15.0 (SCH_2*C*H_3). NMR data were consistent with literature data.[4]

The crude material **S21** was dissolved in 9:1 acetone/water (10 mL) and treated with NBS (865 mg, 4.86 mmol) at room temperature. After 5 h the reaction was quenched with saturated $Na_2S_2O_3$ and diluted with CH_2Cl_2. The organic layer was washed with water and brine, dried over anhydrous $MgSO_4$, filtered and concentrated *in vacuo*. Purification by column chromatography (2:1 to 1:1, Pentane/Et_2O) afforded the hydrolysed product **S22** as a syrup (632 mg, 79% yield over 2 steps, α/β = 79:21).

The following were observed for α/β anomers:

^{1}H NMR (500 MHz, Chloroform-*d*): δ 7.41 – 7.16 (m, 15H, ArCH), 4.57 (d, *J* = 11.9 Hz, 1H, C*H*HPh), 4.52 (d, *J* = 12.0 Hz, 1H, C*H*HPh), 4.49 (d, *J* = 12.1 Hz, 1H, C*H*HPh). ^{13}C NMR (126 MHz, Chloroform-*d*): 21.1 (CH_3).

α-anomer

^{1}H NMR (500 MHz, Chloroform-*d*): δ 5.28 (t, *J* = 9.8 Hz, 1H, H-4), 5.23 (d, *J* = 2.5 Hz, 1H, H-1), 4.75 (d, *J* = 12.4 Hz, 1H, C*H*HPh), 4.67 (d, *J* = 12.4 Hz, 1H, C*H*HPh), 4.46 (d, *J* = 12.2 Hz, 1H, C*H*HPh), 4.06 (ddd, *J* = 10.1, 7.5, 2.6 Hz, 1H, H-5), 3.86 (dd, *J* = 9.6, 3.0 Hz, 1H, H-3), 3.77 (dd, *J* = 3.0, 2.1 Hz, 1H, H-2), 3.60 (dd, *J* = 10.5, 7.5 Hz, 1H, H-6a), 3.47 (dd, *J* = 10.5, 2.6 Hz, 1H, H-6b), 3.38 (d, *J* = 3.4 Hz, 1H, OH), 1.92 (s, 3H, CH_3). ^{13}C NMR (126 MHz, Chloroform-*d*): δ 170.1 (C=O), 138.4 (C), 138.3 (C), 137.89 (C), 128.47 (CH), 128.45 (CH), 128.2 (CH), 128.0 (CH), 127.81 (CH), 127.77 (CH), 127.73 (CH), 127.6 (CH), 93.1 (C-1), 76.9 (C-3), 74.4 (C-2), 73.6 (Ph*C*H_2), 73.0 (Ph*C*H_2), 72.0 (Ph*C*H_2), 70.4 (C-5), 70.2 (C-6), 69.2 (C-4).

β-anomer

^{1}H NMR (500 MHz, Chloroform-*d*): δ 5.33 (t, *J* = 9.3 Hz, 1H, H-4), 5.04 (d, *J* = 11.7 Hz, 1H, C*H*HPh), 4.69 – 4.65 (m, 1H, H-1), 4.66 (d, *J* = 12.1 Hz, 1H, C*H*HPh), 4.64 (d, *J* = 11.7 Hz, 1H, C*H*HPh), 3.83 (dd, *J* = 2.8, 1.5 Hz, 1H, H-2), 3.58 – 3.51 (m, 4H, H-6a, H-6b, H-3, H-5), 1.91 (s, 3H, CH_3). ^{13}C NMR (126 MHz, Chloroform-*d*) selected signals: δ 170.0 (C=O), 138.0 (C), 137.93 (C), 137.8 (C), 128.70 (CH), 128.66 (CH), 128.4 (CH), 128.1 (CH), 127.72 (CH), 93.7 (C-1), 80.1 (C-3), 75.4 (C-2), 74.7 (Ph*C*H_2), 73.7 (C-5), 72.7 (Ph*C*H_2), 70.0 (C-6), 68.9 (C-4). NMR data were consistent with literature data.[13]

2,3,4-Tri-*O*-benzyl-6-*O*-triisopropylsilyl-α/β-D-mannopyranose S23

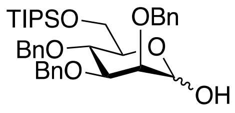

A solution of **S7** (1.00 g, 1.54 mmol) in 9:1 acetone/water (15 mL) and treated with NBS (822 mg, 4.62 mmol) at room temperature. TLC (8:2; Pentane/Et_2O) analysis of reaction after 3 minutes showed complete consumption of starting material and the desired hydrolysed product along with de-silylated hydrolysed product were observed. The reaction was immediately quenched with saturated $Na_2S_2O_3$ and diluted with CH_2Cl_2. The aqueous layer was washed with CH_2Cl_2. The combined organic layers were washed with water and brine, dried over anhydrous $MgSO_4$, filtered and concentrated *in vacuo* to give a colourless syrup. Purification by column chromatography (9:1 to 7:3; Pentane/Et_2O) afforded the hydrolysed product **S23** as a colourless syrup (564 mg, 60% yield, α/β = 72:28). ESI-HRMS for $C_{36}H_{50}O_6SiNa^+$ $(M+Na)^+$ calculated: 629.3269; found: 629.3269.

The following were observed for α/β anomers:

^{1}H NMR (600 MHz, Chloroform-*d*): δ 7.40 – 7.25 (m, 15H, ArCH), 1.16 – 1.03 (m, 3H, $SiCH(CH_3)_2$), 1.06 (s, 12H, $SiCH(CH_3)_2$), 1.05 (s, 6H, $SiCH(CH_3)_2$). ^{13}C NMR (151 MHz, Chloroform-*d*): δ 18.2 ($SiCH(CH_3)_2$), 18.1 ($SiCH(CH_3)_2$).

α-anomer

^{1}H NMR (600 MHz, Chloroform-*d*): δ 5.23 (dd, *J* = 3.6, 2.0 Hz, 1H, H-1), 4.92 (d, *J* = 10.9 Hz, 1H, C*H*HPh), 4.75 (d, *J* = 12.5 Hz, 1H, C*H*HPh), 4.67 – 4.63 (m, 4H, 4 x C*H*HPh), 4.00 – 3.96 (m, 2H, H-4, H-3), 3.94 (dd, *J* = 11.0, 4.8 Hz, 1H, H-6a), 3.91 (dd, *J* = 11.1, 2.1 Hz, 1H, H-6b), 3.87 – 3.81 (m, 1H, H-5), 3.78 (t, *J* = 2.4 Hz, 1H, H-2), 2.80 (d, *J* = 3.5 Hz, 1H, OH).

^{13}C NMR (151 MHz, Chloroform-*d*): δ 138.9 (C), 138.8 (C), 138.7 (C), 128.47 (CH), 128.46 (CH), 128.4 (CH), 128.2 (CH), 127.86 (CH), 127.82 (CH), 127.81 (CH), 127.79 (CH), 92.8 (C-1), 79.8 (C-3), 75.6 (C-2), 75.2 ($PhCH_2$), 74.9 (C-4), 73.8 (C-5), 72.8 ($PhCH_2$), 72.40 ($PhCH_2$), 63.4 (C-6), 12.17 ($SiCH(CH_3)_2$).

β-anomer

^{1}H NMR (600 MHz, Chloroform-*d*): δ 5.11 (d, *J* = 11.6 Hz, 1H, C*H*HPh), 4.87 (d, *J* = 10.8 Hz, 1H, C*H*HPh), 4.79 – 4.74 (m, 2H, 2 x C*H*HPh), 4.73 (d, *J* = 10.9 Hz, 1H, C*H*HPh), 4.69 – 4.66 (m, 1H, H-1), 4.61 (d, *J* = 11.5 Hz, 1H, C*H*HPh), 4.08 (t, *J* = 9.2 Hz, 1H, H-4), 4.01 – 3.95 (m, 2H, H-6a, H-6b), 3.87 – 3.81 (m, 1H, H-2), 3.65 (d, *J* = 12.2 Hz, 1H, OH), 3.64 (dd, *J* = 9.3, 2.7 Hz, 1H, H-3), 3.31 (dt, *J* = 9.1, 3.0 Hz, 1H, H-5). ^{13}C NMR (151 MHz, Chloroform-*d*): δ 138.5 (C), 138.3 (C), 128.6 (CH), 128.6 (CH), 128.53 (CH), 128.1 (CH), 127.94 (CH), 127.84 (CH), 127.68 (CH), 127.67 (CH), 127.6 (CH),

93.5 (C-1), 83.0 (C-3), 77.0 (C-2), 76.6 (C-5), 75.1 (Ph*C*H_2), 74.8 (Ph*C*H_2), 74.3 (C-4), 73.1 (Ph*C*H_2), 62.9 (C-6), 12.19 (Si*C*H(CH_3)$_2$).

2,3,4,6-Tetra-*O*-acetyl-α-D-mannopyranose S24

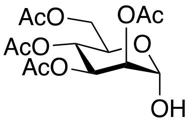

Following the literature procedure,[14,15] the penta-*O*-acetate mannose **S1** (2.17 g, 5.55 mmol), and $FeCl_3.6H_2O$ (749 mg, 2.77 mmol) were dissolved in bench MeCN (10 mL) and heated to 90 °C using MW 150W. TLC analysis (2:1; cyclohexane/EtOAc) after 30 min showed complete consumption of starting material. The reaction mixture was diluted with CH_2Cl_2 and washed with water, saturated $NaHCO_3$, and brine. The organic layer was dried over anhydrous $MgSO_4$, filtered and concentrated *in vacuo*. Purification by column chromatography (R_f = 0.4, 5:1; CH_2Cl_2/EtOAc) gave the product **S24** as a yellow syrup (290 mg, 63% yield, α/β ≥ 95:5). ^{1}H NMR (500 MHz, Chloroform-*d*): δ 5.43 (dd, *J* = 10.0, 3.4 Hz, 1H, H-3), 5.31 (t, *J* = 10.0 Hz, 1H, H-4), 5.27 (dd, *J* = 3.4, 1.9 Hz, 1H, H-2), 5.25 (br s, 1H, H-1), 4.28 – 4.21 (m, 2H, H-6a, H-5), 4.17 – 4.12 (m, 1H, H-6b), 3.64 (br s, 1H, OH), 2.17 (s, 3H, CH_3), 2.11 (s, 3H, CH_3), 2.06 (s, 3H, CH_3), 2.01 (s, 3H, CH_3). ^{13}C NMR (126 MHz, Chloroform-*d*): δ 171.0 (C=O), 170.3 (C=O), 170.2 (C=O), 169.9 (C=O), 92.3 (C-1), 70.14 (C-2), 68.9 (C-3), 68.6 (C-5), 66.3 (C-4), 62.71 (C-6), 21.0 (CH_3), 20.9 (CH_3), 20.85 (CH_3), 20.83 (CH_3). NMR data were consistent with literature data.[16]

6-*O*-Pivaloyl-2,3,4-tri-*O*-*p*-methylbenzyl-α/β-D-mannopyranose 1i

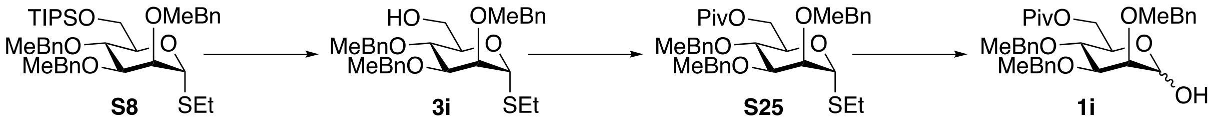

For the synthesis of **3i** see p.54.

Under a N_2 atmosphere, **3i** (200 mg, 0.37 mmol) was dissolved in anhydrous CH_2Cl_2 (3.7 mL) and the reaction was treated with anhydrous pyridine (0.11 mL, 1.4 mmol) followed by pivaloyl chloride (0.146 mL, 1.20 mmol) at room temperature. TLC (cyclohexane:EtOAc) analysis of the reaction after 1 h showed complete consumption of starting material. The reaction was quenched with water and diluted

with CH_2Cl_2. The organic layer was washed with 1 M HCl, saturated $NaHCO_3$ and brine. The organic layer was dried over anhydrous $MgSO_4$, filtered and concentrated *in vacuo* to give a colourless oil.

The crude material **S25** was dissolved in 9:1 acetone/water (4 mL) and treated with NBS (214 mg, 1.20 mmol) at room temperature. After 5 minutes the reaction was quenched with saturated $Na_2S_2O_3$ and diluted with CH_2Cl_2. The aqueous layer was washed with CH_2Cl_2. The combined organic layers were washed with water and brine, dried over anhydrous $MgSO_4$, filtered and concentrated *in vacuo* to give a colourless syrup. Purification by column chromatography (2:1 to 1:1; Pentane/Et_2O) afforded the hydrolysed product **1i** as a colourless syrup (176 mg, 83% yield over 2 steps, α/β = 71:29). ESI-HRMS for $C_{35}H_{44}O_7NH_4^+$ $(M+NH_4)^+$ calculated: 594.3431; found: 594.3420.

The following were observed for α/β anomers:
^{1}H NMR (500 MHz, Chloroform-*d*): δ 7.29 – 7.07 (m, 17H, ArCH), 4.70 (d, *J* = 12.0 Hz, 2H 2 x C*H*HPh), 4.61 – 4.51 (m, 5H, 5 x C*H*HPh), 2.37 – 2.31 (m, 13H, CH_3). ^{13}C NMR (126 MHz, Chloroform-*d*) δ 129.3 (ArCH), 129.23 (ArCH), 129.19 (ArCH), 129.11 (ArCH), 129.08 (ArCH), 129.0 (ArCH), 128.3 (ArCH), 128.2 (ArCH), 128.0 (ArCH), 127.93 (ArCH), 127.90 (ArCH), 127.85 (ArCH), 75.1 (*C*HHPh), 72.6 (*C*HHPh).

α-anomer
^{1}H NMR (500 MHz, Chloroform-*d*): δ 5.17 (d, *J* = 1.6 Hz, 1H, H-1), 4.90 (d, *J* = 10.5 Hz, 1H, C*H*HPh), 4.47 – 4.43 (m, 1H, H-6a), 4.23 (dd, *J* = 11.9, 3.1 Hz, 1H, H-6b), 3.99 – 3.90 (m, *J* = 3.7 Hz, 3H, H-2, H-3, H-4), 3.76 (br s, 1H, H-2), 3.10 (br s, 1H, OH), 1.19 (s, 9H, CH_3). ^{13}C NMR (126 MHz, Chloroform-*d*): δ 178.6 (C=O), 137.5 (4º C), 137.3 (4º C), 137.2 (4º C), 135.4 (4º C), 135.3 (2 x 4º C), 92.7 (C-1), 79.5 (C-3), 74.9 (C-2), 74.5 (C-4), 72.1 (*C*HHPh), 70.6 (C-5), 62.9 (C-6), 38.9 (*C*$(CH_3)_3$), 27.2 (2 x CH_3), 21.2 (CH_3).

β-anomer
^{1}H NMR (500 MHz, Chloroform-*d*): δ 5.06 (d, *J* = 11.4 Hz, 1H, C*H*HPh), 4.87 (d, *J* = 10.5 Hz, 1H, C*H*HPh), 4.62 (d, *J* = 4.8 Hz, 1H, H-1), 4.41 (dd, *J* = 11.9, 2.1 Hz, 1H, H-6a), 4.23 (dd, *J* = 11.9, 3.1 Hz, 1H, H-6b), 3.87 (t, *J* = 9.4 Hz, 1H, H-4), 3.80 (dd, *J* = 2.5, 1.3 Hz, 1H, H-2), 3.60 (dd, *J* = 9.3, 2.7 Hz, 1H, H-3), 3.49 – 3.43 (m, 1H, H-5), 1.18 (s, 9H, CH_3). ^{13}C NMR (126 MHz, Chloroform-*d*): δ 178.3 (C=O), 137.72 (4º C), 137.68 (2 x 4º C), 135.2 (4º C), 135.0 (4º C), 134.9 (4º C), 93.5 (C-1), 82.8 (C-3), 76.3 (C-2), 74.6 (*C*HHPh), 74.2 (C-4), 73.6 (C-5), 72.7 (*C*HHPh), 63.1 (C-6), 38.9 (*C*$(CH_3)_3$), 27.2 (2 x CH_3), 21.2 (CH_3).

Acetyl 2,3,4,6-Tetra-*O*-benzyl-α/β-D-mannopyranose 8a

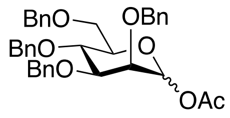

A solution of **1a** (150 mg, 0.28 mmol) in pyridine (200 μL) was treated with acetic anhydride (200 μL, 2.00 mmol) and stirred at room temperature. TLC (4:1; cyclohexane/EtOAc) analysis after 3 h showed complete consumption of starting material. The reaction mixture was diluted with CH_2Cl_2 and washed with 1 M HCl, saturated $NaHCO_3$, and brine. The organic layer was dried over anhydrous $MgSO_4$, filtered and concentrated *in vacuo* to give **8a** as a yellowish syrup (163 mg, quantitative yield, α/β = 89:11).

α-anomer

^{1}H NMR (500 MHz, Chloroform-*d*): δ 7.42 – 7.37 (m, 2H, ArCH), 7.36 – 7.22 (m, 16H, ArCH), 7.20 – 7.15 (m, 2H, ArCH), 6.22 (d, *J* = 2.1 Hz, 1H, H-1), 4.89 (d, *J* = 10.6 Hz, 1H, C*H*HPh), 4.78 (d, *J* = 12.3 Hz, 1H, C*H*HPh), 4.73 (d, *J* = 12.4 Hz, 1H, C*H*HPh), 4.66 (d, *J* = 12.1 Hz, 1H, C*H*HPh), 4.59 (d, *J* = 11.9 Hz, 1H, C*H*HPh), 4.58 – 4.50 (m, 3H, 3 x C*H*HPh), 4.08 (t, *J* = 9.6 Hz, 1H, H-4), 3.88 – 3.82 (m, 2H, H-3, H-5), 3.78 (dd, *J* = 11.0, 4.7 Hz, 1H, H-6a), 3.73 (dd, *J* = 3.2, 2.1 Hz, 1H, H-2), 3.71 (dd, *J* = 11.0, 1.9 Hz, 1H, H-6b), 2.01 (s, 3H, CH_3). ^{13}C NMR (126 MHz, Chloroform-*d*): δ 169.1 (C=O), 138.4 (2 x C), 138.3 (C), 138.0 (C), 128.51 (CH), 128.45 (CH), 128.2 (CH), 128.1 (CH), 128.0 (CH), 127.89 (CH), 127.85 (CH), 127.84 (CH), 127.7 (CH), 92.0 (C-1), 79.3 (C-3), 75.4 (Ph*C*H), 74.6 (C-5), 74.4 (C-4), 73.6 (Ph*C*H), 73.5 (C-2), 72.6 (Ph*C*H), 72.2 (Ph*C*H), 69.0 (C-6), 21.2 (CH_3).

β-anomer

^{1}H NMR (500 MHz, Chloroform-*d*) selected signals: 5.59 (d, *J* = 1.1 Hz, 1H, H-1), 3.99 (t, *J* = 9.4 Hz, 1H, H-4), 3.94 (dd, *J* = 2.8, 1.1 Hz, 1H, H-2), 3.62 (dd, *J* = 9.3, 2.8 Hz, 1H, H-3), 3.56 (dt, *J* = 9.6, 3.6 Hz, 1H, H-5), 2.07 (s, 3H, CH_3). NMR data were consistent with literature data.[17]

Synthesis of Rhamnosyl Donors

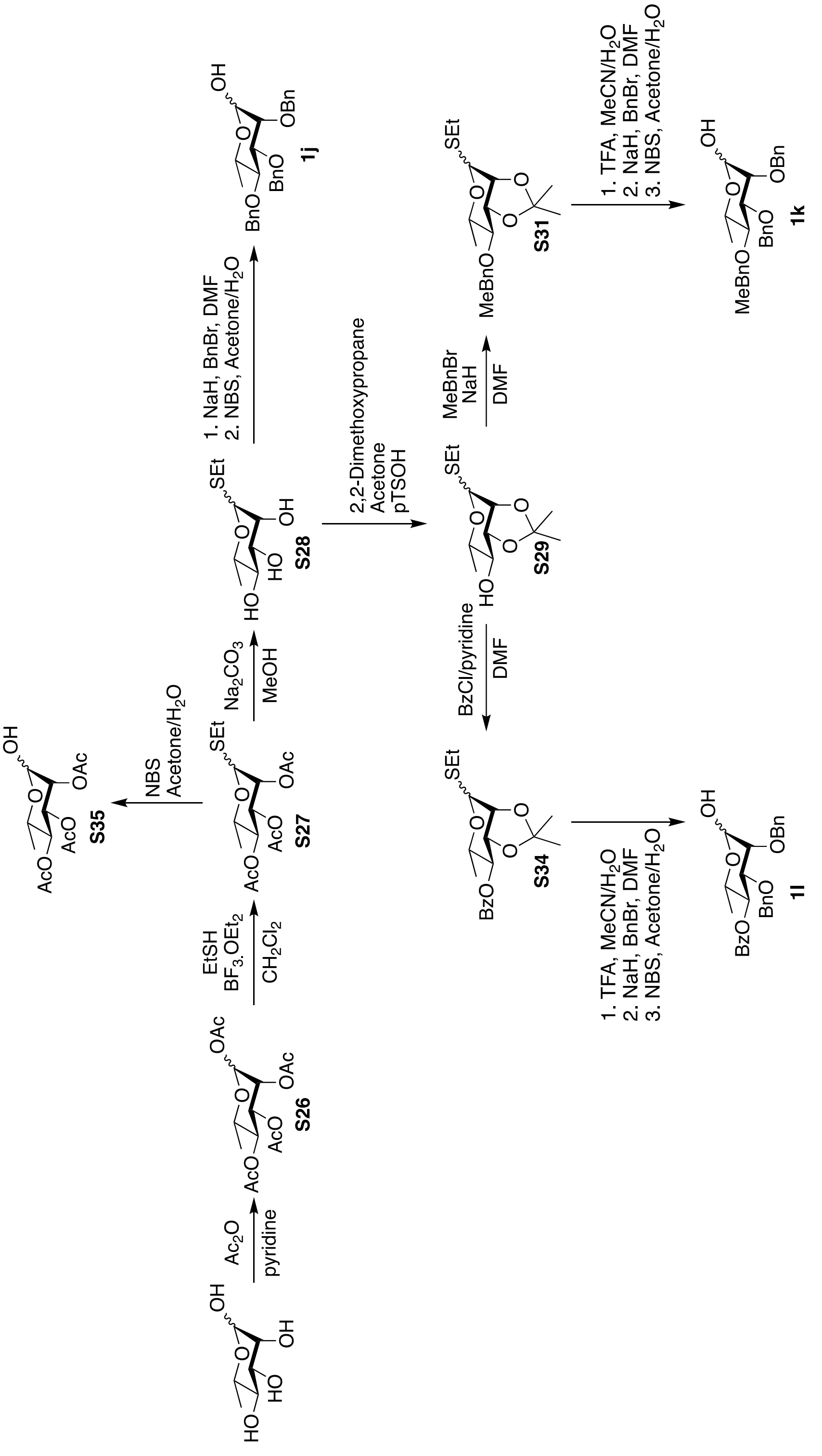

Scheme 2. Syntheses of rhamnosyl donors

Ethyl 1-thiol-L-rhamnoside S28

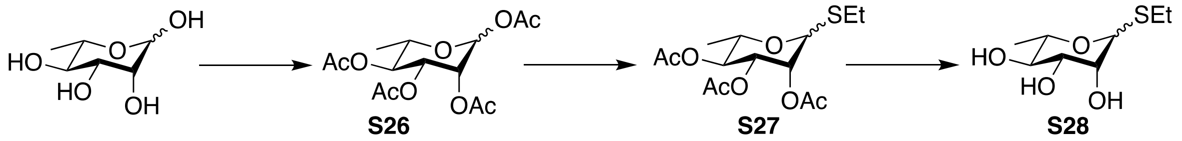

L-Rhamnose (5.00 g, 30.4 mmol) was added to acetic anhydride (70.0 mL, 735 mmol) giving a cloudy solution. Pyridine (70.0 mL, 863 mmol) was added, and the mixture was stirred at room temperature for 15 mins, after which the cloudy solution had turned clear. TLC analysis (cyclohexane: EtOAc; 6:4; R_f= 0.6) showed full conversion of the starting material into a single product. The reaction was diluted with CH_2Cl_2 (150 mL) and 1 M HCl (50 mL) was added and stirring was continued for 0.5 h. The organic layer was washed with saturated $NaHCO_3$ solution (3 x 50 mL), water (3 x 50 mL), brine (1 x 50 mL), dried over anhydrous $MgSO_4$ and filtered. The resulting solution was concentrated in *vacuo* to give the product **S26** as a pale-yellow oil (8.70 g, 26.2 mmol, 88%, α/β 84:16). ^{1}H NMR (600 MHz, Chloroform-*d*) δ 6.02 (d, *J* = 2.0 Hz, 1H), 5.32 – 5.29 (m, 1H), 5.25 (dd, *J* = 3.6, 2.0 Hz, 1H), 5.12 (t, *J* = 10.0 Hz, 1H), 3.94 (dq, *J* = 9.7, 6.2 Hz, 1H), 2.17 (s, 3H), 2.16 (s, 3H), 2.07 (s, 3H), 2.01 (s, 3H), 1.24 (d, *J* = 6.2 Hz, 3H). ^{13}C NMR (151 MHz, Chloroform-*d*) δ 170.0, 169.79, 169.77, 168.3, 90.6, 70.4, 68.74, 68.68, 68.6, 20.9, 20.8, 20.72, 20.65, 17.4. NMR data are consistent with the literature.[18]

Under a N_2 atmosphere, a solution of tetraacetate rhamnose **S26** (8.70 g, 26.2 mmol) in anhydrous CH_2Cl_2 (262 mL) was treated with ethanethiol (4.90 mL, 68.1 mmol) at room temperature. The reaction mixture was stirred at 0 °C for 30 min after which $BF_3.Et_2O$ (16.2 mL, 131 mmol) was slowly added. After stirring the reaction mixture at room temperature for 20 h, TLC analysis (cyclohexane: EtOAc; 6:4; R_f= 0.7) showed full conversion of the starting material into a single product. The reaction mixture was then carefully quenched with saturated $NaHCO_3$. The organic layer was washed with water and brine, dried over anhydrous $MgSO_4$ and concentrated in *vacuo* to give **S27** as a yellow oil (9.00 g, 26.2 mmol, quant., α/β 80:20). ^{1}H NMR (500 MHz, Chloroform-*d*) δ 5.34 (dd, *J* = 3.4, 1.6 Hz, 1H), 5.23 (dd, *J* = 10.1, 3.4 Hz, 1H), 5.20 (d, *J* = 1.5 Hz, 1H), 5.10 (t, *J* = 9.9 Hz, 1H), 4.29 – 4.18 (m, 1H), 2.69 – 2.57 (m, 2H), 2.16 (s, 3H), 2.06 (s, 3H), 1.99 (s, 3H), 1.30 (t, *J* = 7.4 Hz, 3H), 1.24 (d, *J* = 6.3 Hz, 3H). NMR data are consistent with the literature.[19]

Thiorhamnoside **S27** (9.0 g, 26.2 mmol) was dissolved in MeOH (300 mL). Na_2CO_3 (0.5 g, 5.0 mmol) was added to the solution and the mixture was left to stir at room temperature for 2 h, after which TLC analysis (EtOAc; R_f = 0) indicated that the reaction had gone to completion. The reaction mixture was neutralised with resin IR-120 and the mixture was filtered and was concentrated in *vacuo* to give **S28** as a yellow oil, which was used in the next step without further purification.

Ethyl 2,3-*O*-isopropylidene-1-thio-L-rhamnoside S29

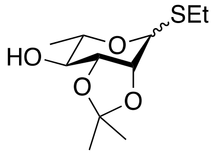

Thiorhamnoside **S28** (3.00 g, 14.4 mmol) was dissolved in acetone (21 mL) and *p*TsOH.H_2O (0.68 g, 2.64 mmol) was added to the reaction mixture. 2,2-Dimethoxypropane (31.8 mL, 259 mmol) was added and the reaction left to stir at room temperature overnight. TLC analysis (CH_2Cl_2:MeOH; 97:3; R_f =0.7) indicated that the reaction had gone to completion. Et_3N (2 mL) was added to quench the reaction mixture and the solvent was concentrated in *vacuo*. The crude residue was dissolved in CH_2Cl_2 (50 mL) and washed with water, dried over anhydrous Na_2SO_4, filtered and concentrated in *vacuo*. Purification by column chromatography (pentane: EtOAc; 95:5 to 80:20) led to separation of the two anomers of **S29** (α anomer; 2.23 g, 8.99 mmol, 63%, β anomer; 0.35 g, 1.4 mmol, 16%).

α-anomer:

^{1}H NMR (500 MHz, Chloroform-*d*) δ 5.52 (d, J = 0.9 Hz, 1H, H-1), 4.17 (dd, J = 5.5, 0.8 Hz, 1H, H-2), 4.05 (dd, J = 7.6, 5.5 Hz, 1H, H-3), 4.00 – 3.93 (m, 1H, H-5), 3.43 (ddd, J = 9.7, 7.6, 4.0 Hz, 1H, H-4), 2.91 (dd, J = 4.6, 1.9 Hz, 1H, OH), 2.67 (dq, J = 13.0, 7.4 Hz, 1H, SCH_2CH_3), 2.54 (dq, J = 13.0, 7.4 Hz, 1H, SCH_2CH_3), 1.54 (s, 3H, CH_3), 1.35 (s, 3H, CH_3), 1.33 – 1.28 (m, 6H, SCH_2CH_3, H-6). NMR data are consistent with the literature.[19]

β-anomer:

^{1}H NMR (500 MHz, Chloroform-*d*) δ 4.83 (d, J = 2.2 Hz, 1H, H-1), 4.28 (dd, J = 5.5, 2.2 Hz, 1H, H-2), 3.99 (dd, J = 7.3, 5.4 Hz, 1H, H-3), 3.45 (ddd, J = 9.5, 7.3, 3.4 Hz, 1H, H-4), 3.30 – 3.23 (m, 1H, H-5), 2.96 – 2.89 (m, 1H, OH), 2.77 (q, J = 7.5 Hz, 2H, SCH_2CH_3), 1.55 (s, 3H, CH_3), 1.38 (s, 3H, CH_3), 1.36 – 1.30 (m, 6H, SCH_2CH_3, H-6). NMR data are consistent with the literature.[20]

2,3,4-Tri-*O*-benzyl-L-rhamnoside 1j

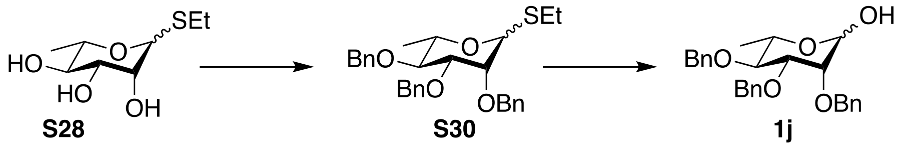

Under a N_2 atmosphere, thiorhamnoside **S28** (3.00 g, 14.4 mmol) was dissolved in anhydrous DMF (72 mL). The flask was cooled to 0 °C (50:50; ice/water), and NaH (60% dispersion in mineral oil) (2.60 g, 108 mmol) was added to the reaction flask. The ice bath was removed, and the reaction was left to stir

at room temperature for 30 min. The flask was again cooled to 0 °C, and BnBr (6.0 mL, 50.4 mmol) and TBAI (0.44 g, 1.2 mmol) were added to the reaction mixture. The ice bath was removed, and the reaction mixture was left to stir at room temperature overnight, after which TLC analysis (cyclohexane: EtOAc; 9:1; R_f = 0.5) showed that the starting material had been consumed. The reaction was quenched with MeOH (5 mL), and the solvents were removed using rotary evaporation. The crude mixture was dissolved in CH_2Cl_2 (100 mL) and washed with 1 M HCl (2 × 50 mL), deionised water (1 × 50 mL), saturated $NaHCO_3$ (2 × 50 mL), deionised H_2O (1 × 50 mL) and brine (1 × 50 mL). The organic layer was dried over anhydrous Na_2SO_4, filtered off and concentrated in *vacuo*.

Crude rhamnoside **S30** (~14.7 mmol) was dissolved in a 9:1 mixture of acetone:water (140 mL) and NBS (7.70 g, 44.1 mmol) was added. The reaction left to stir at room temperature for 2 h when TLC analysis (cyclohexane: EtOAc; 8:2; R_f = 0.3) showed the complete conversion of the starting material to the desired product. The mixture was quenched with saturated $Na_2S_2O_3$ (50 mL), concentrated in *vacuo* to remove acetone and diluted with CH_2Cl_2 (250 mL). The organic phase was then washed with saturated $NaHCO_3$ (50 mL), deionised H_2O (50 mL) and brine (50 mL). The organic layer was dried over anhydrous Na_2SO_4, filtered off and concentrated in *vacuo*. Purification by column chromatography (pentane: EtOAc; 9:1 to 8:2) gave product **1j** as a white solid (6.0 g, 13.8 mmol, 96%, α/β 1:1).

Signals observed for both anomers:

^{1}H NMR (500 MHz, Chloroform-*d*) δ 7.41 – 7.25 (m, 30H, Ar-CH), 5.10 (d, *J* = 11.6 Hz, 1H, $PhCH_2$), 4.94 (d, *J* = 10.9, 1H, $PhCH_2$), 4.93 (*J* = 10.8, 1H, $PhCH_2$), 4.81 – 4.62 (m, 9H, 9 x $PhCH_2$). ^{13}C NMR (126 MHz, Chloroform-*d*) δ 138.60 (4° C), 138.56 (4° C), 138.33 (4° C), 138.28 (4° C), 138.07 (4° C), 138.05 (4° C), 128.63 (Ar-*C*H), 128.55 (Ar-*C*H), 128.43 (Ar-*C*H), 128.36 (Ar-*C*H), 128.2 (Ar-*C*H), 128.09 (Ar-*C*H), 128.06 (Ar-*C*H), 128.02 (Ar-*C*H), 128.01 (Ar-*C*H), 127.9 (Ar-*C*H), 127.8 (Ar-*C*H), 127.7 (Ar-*C*H), 127.63 (Ar-*C*H), 127.55 (Ar-*C*H), 75.43 (Ph*C*H_2), 75.35 (Ph*C*H_2), 74.9 (Ph*C*H_2), 72.9 (Ph*C*H_2), 72.3 (Ph*C*H_2).

α-anomer:

^{1}H NMR (500 MHz, Chloroform-*d*) δ 5.15 (d, *J* = 1.9 Hz, 1H, H-1), 3.96 – 3.89 (m, 2H, H-3, H-5), 3.80 (dd, *J* = 3.1, 2.0 Hz, 1H, H-2), 3.63 (t, *J* = 9.4 Hz, 1H, H-4), 2.71 (s, 1H, OH), 1.31 (d, *J* = 6.2 Hz, 3H, H-6). ^{13}C NMR (126 MHz, Chloroform-*d*) δ 93.0 (C-1), 80.5 (C-4), 79.7 (C-3), 75.0 (C-2), 68.1 (C-5), 18.1 (C-6).

β-anomer:

^{1}H NMR (500 MHz, Chloroform-*d*) δ 4.61 (t, *J* = 4.5 Hz, 1H, H-1), 3.84 (d, *J* = 1.9 Hz, 1H, H-2), 3.59 – 3.54 (m, 2H, H-3, H-4), 3.40 – 3.32 (m, 1H, H-5), 1.34 (d, *J* = 6.2 Hz, 1H, H-6). ^{13}C NMR (126 MHz, Chloroform-*d*) δ 93.4 (C-1), 83.1 (C-3), 78.0 (C-4), 76.55 (C-2), 71.61 (C-5), 17.9 (C-6).

NMR data are consistent with the literature.[21]

2,3-Di-*O*-benzyl-4-*O*-*p*-methylbenzyl-L-rhamnoside 1k

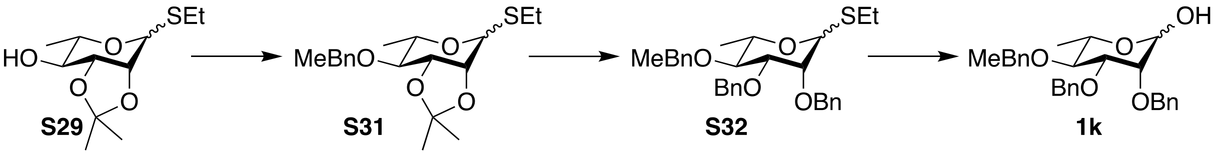

Under an N_2 atmosphere, thiorhamnoside **S29** (1 g, 4 mmol) was dissolved in anhydrous DMF (20 mL, 0.5 M) and NaH (60% dispersion in mineral oil) (192 mg, 8.00 mmol) was added. The ice bath was removed, and the reaction was left to stir at room temperature for 30 min. The flask was again cooled to 0 °C, and *p*MeBnBr (0.93 g, 5.0 mmol) was added to the reaction mixture. The ice bath was removed, and the reaction mixture was left to stir at room temperature overnight, after which TLC analysis (cyclohexane:EtOAc; 4:1; R_f = 0.8) showed that the starting material had been consumed. The reaction was quenched with MeOH (2 mL), and the solvents were removed using rotary evaporation. The crude mixture was dissolved in CH_2Cl_2 (100 mL) and washed with 1 M HCl (2 × 50 mL), deionised water (1 × 50 mL), saturated $NaHCO_3$ (2 × 50 mL), deionised H_2O (1 × 50 mL) and brine (1 × 50 mL). The organic layer was dried over anhydrous Na_2SO_4, filtered off and concentrated in *vacuo*.

Crude thiorhamnoside **S31** was dissolved in CH_2Cl_2 (12 mL) and H_2O (0.5 mL) was added. TFA (4.0 mL, 52 mmol) was then added and the reaction left to stir at RT overnight. TLC analysis (cyclohexane: EtOAc; 7:3; R_f =0.3) indicated that the reaction had gone to completion. The reaction mixture was quenched saturated $NaHCO_3$ (20 mL), diluted with CH_2Cl_2 (50 mL) and the two layers were separated. The aqueous phase was extracted with CH_2Cl_2 (100 mL) and the combined organic layers were washed with water (20 mL), brine (20 mL), dried over anhydrous Na_2SO_4, filtered and concentrated in *vacuo*. The crude material was used in the next step without further purification. Under a N_2, the crude thiorhamnoside was dissolved in anhydrous DMF (20 mL, 0.2 M). The flask was cooled to 0 °C (50:50; ice/water), and NaH (60% dispersion in mineral oil) (384 mg, 16.0 mmol) was added to the reaction flask. The ice bath was removed, and the reaction was left to stir at room temperature for 30 min. The flask was again cooled to 0 °C, and BnBr (1.2 mL, 10 mmol) was added to the reaction mixture. The ice bath was removed, and the reaction mixture was left to stir at room temperature for 4 h, after which TLC analysis (cyclohexane: EtOAc; 4:1; R_f = 0.6) showed that the starting material had been consumed. The reaction was quenched with MeOH (2 mL), and the solvents were removed using rotary evaporation. The crude mixture was dissolved in CH_2Cl_2 (100 mL) and washed with 1 M HCl (2 × 50 mL), deionised water (1 × 50 mL), saturated $NaHCO_3$ (2 × 50 mL), deionised H_2O (1 × 50 mL) and brine (1 × 50 mL). The organic layer was dried over anhydrous Na_2SO_4, filtered off and concentrated in *vacuo*.

Crude rhamnoside **S32** was dissolved in a 9:1 mixture of acetone:water (20 mL) and NBS (1.1 mg, 6.0 mmol) was added. The reaction left to stir at room temperature for 1 h when TLC analysis (cyclohexane: EtOAc; 6:4; R_f = 0.6) showed the complete conversion of the starting material to the desired product. The crude mixture was dissolved in CH_2Cl_2 (100 mL) and washed with saturated $Na_2S_2O_3$ (50 mL), saturated $NaHCO_3$ (50 mL), deionised H_2O (50 mL) and brine (50 mL). The organic layer was dried over anhydrous Na_2SO_4, filtered off and concentrated in *vacuo*. Purification by column chromatography (pentane: EtOAc; 9:1 to 8:2) gave product **1k** as an off-white solid (757 mg, 1.75 mmol, 87%, α/β 76:24). ESI-HRMS for $C_{28}H_{32}O_5NH_4^+$ $(M+NH_4)^+$ calculated: 466.2588; found: 466.2588.

Signals observed for both anomers:

^{1}H NMR (500 MHz, Chloroform-*d*) δ 7.40 – 7.25 (m, 11H, Ar-CH), 7.21 (d, *J* = 7.8 Hz, 3H, Ar-CH), 7.16 – 7.10 (m, 3H, Ar-CH), 5.10 (d, *J* = 11.6 Hz, 0.5H, $PhCH_2$), 4.90 (d, *J* = 10.6, 1H, $PhCH_2$), 4.88 (d, *J* = 10.5, 0.5H, $PhCH_2$), 4.81 – 4.71 (m, 3H, 3 x $PhCH_2$), 4.69 (d, *J* = 11.1 Hz, 1H, $PhCH_2$), 4.66 – 4.63 (m, 2H, 2 x $PhCH_2$), 4.60 (d, *J* = 10.8 Hz, 1H, $PhCH_2$). ^{13}C NMR (126 MHz, Chloroform-*d*) δ 138.6 (4° C), 138.3 (4° C), 138.13 (4° C), 138.05 (4° C), 137.5 (4° C), 137.3 (4° C), 135.5 (4° C), 135.2 (4° C), 129.1 (Ar-*C*H), 129.0 (Ar-*C*H), 128.6 (Ar-*C*H), 128.5 (Ar-*C*H), 128.4 (Ar-*C*H), 128.3 (Ar-*C*H), 128.20 (Ar-*C*H), 128.15 (Ar-*C*H), 128.1 (Ar-*C*H), 128.0 (Ar-*C*H), 127.8 (Ar-*C*H), 127.67 (Ar-*C*H), 127.66 (Ar-*C*H), 127.62 (Ar-*C*H), 127.5 (Ar-*C*H), 75.3 (Ph*C*H_2), 75.2 (Ph*C*H_2), 74.9 (Ph*C*H_2), 72.94 (Ph*C*H_2), 72.90 (Ph*C*H_2), 72.3 (Ph*C*H_2).

α-anomer:

^{1}H NMR (500 MHz, Chloroform-*d*) δ 5.15 (dd, *J* = 3.4, 1.9 Hz, 1H, H-1), 3.95 – 3.86 (m, 2H, H-5, H-3), 3.80 (dd, *J* = 3.1, 1.9 Hz, 1H, H-2), 3.66 – 3.57 (m, 1H, H-4), 2.62 – 2.60 (m, 1H, OH), 2.33 (s, 3H, CH_3), 1.31 (d, *J* = 6.3 Hz, 3H, H-6). ^{13}C NMR (126 MHz, Chloroform-*d*) δ 93.0 (C-1), 80.4 (C-4), 79.6 (C-3), 75.1 (C-2), 68.3 (C-5), 21.2 (*C*H_3), 18.1 (C-6).

β-anomer:

^{1}H NMR (500 MHz, Chloroform-*d*) δ 4.62 (s, 1H, H-1), 3.85 – 3.82 (m, 1H, H-2), 3.57 – 3.52 (m, 2H, H-3, H-4), 3.39 – 3.30 (m, 1H, H-5), 2.46 (d, *J* = 3.5 Hz, 1H, OH), 2.34 (s, 3H, CH_3), 1.33 (d, *J* = 6.2 Hz, 3H, H-6). ^{13}C NMR (126 MHz, Chloroform-*d*) δ 93.3 (C-1), 83.1 (C-3), 79.8 (C-4), 76.6 (C-2), 71.6 (C-5), 21.2 (*C*H_3), 17.9 (C-6).

2,3-Di-*O*-benzyl-4-*O*-benzoyl-L-rhamnoside 1l

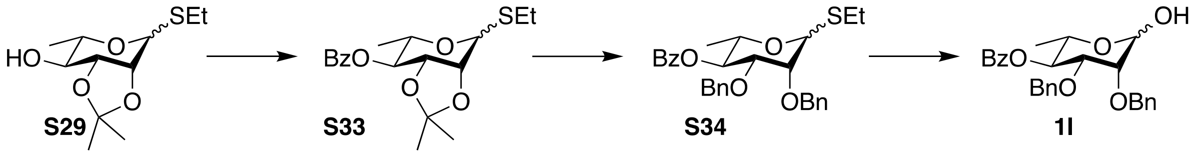

Under an N_2 atmosphere, thiorhamnoside **S29** (1 g, 4 mmol) was dissolved in anhydrous DMF (20 mL, 0.5 M) and BzCl (0.9 mL, 8.0 mmol) was added. Pyridine (1.29 mL, 16.0 mmol) was then added and the reaction left to stir at RT overnight. TLC analysis (cyclohexane: EtOAc; 4:1; R_f =0.8) indicated that the reaction had gone to completion. The reaction mixture was diluted with CH_2Cl_2 (100 mL) and washed with 1 M HCl (50 mL). The aqueous phase was extracted with CH_2Cl_2 (100 mL) and the combined organic layers were washed with saturated $NaHCO_3$ (50 mL), water (50 mL) and brine (50 mL). The organic phase was dried over anhydrous Na_2SO_4, filtered and concentrated in *vacuo*.

Crude thiorhamnoside **S33** was dissolved in CH_2Cl_2 (12 mL) and H_2O (0.5 mL) was added. TFA (4.0 mL, 52 mmol) was then added and the reaction left to stir at RT overnight. TLC analysis (cyclohexane:EtOAc; 7:3; R_f = 0.4) indicated that the reaction had gone to completion. The reaction mixture was quenched saturated $NaHCO_3$ (20 mL), diluted with CH_2Cl_2 (50 mL) and the two layers were separated. The aqueous phase was extracted with CH_2Cl_2 (100 mL) and the combined organic layers were washed with water (20 mL), brine (20 mL), dried over anhydrous Na_2SO_4, filtered and concentrated in *vacuo*. The crude material was used in the next step without further purification. Under a N_2, the crude thiorhamnoside was dissolved in anhydrous DMF (20 mL). The flask was cooled to 0 °C (50:50; ice/water), and NaH (60% dispersion in mineral oil) (384 mg, 16.0 mmol) was added to the reaction flask. The ice bath was removed, and the reaction was left to stir at room temperature for 30 min. The flask was again cooled to 0 °C, and BnBr (1.2 mL, 10 mmol) was added to the reaction mixture. The ice bath was removed, and the reaction mixture was left to stir at room temperature for 4 h, after which TLC analysis (cyclohexane: EtOAc; 4:1; R_f= 0.6) showed that the starting material had been consumed. The reaction was quenched with MeOH (2 mL), and the solvents were removed using rotary evaporation. The crude mixture was dissolved in CH_2Cl_2 (100 mL) and washed with 1 M HCl (2 × 50 mL), deionised water (1 × 50 mL), saturated $NaHCO_3$ (2 × 50 mL), deionised H_2O (1 × 50 mL) and brine (1 × 50 mL). The organic layer was dried over anhydrous Na_2SO_4, filtered off and concentrated in *vacuo*.

Crude rhamnoside **S34** (~1.0 mmol) was dissolved in a 9:1 mixture of acetone:water (9:1 mL) and NBS (534 mg, 3 mmol) was added. The reaction left to stir at room temperature for 1 h when TLC analysis (cyclohexane: EtOAc; 6:4; R_f= 0.5) showed the complete conversion of the starting material to the desired product. The crude mixture was dissolved in CH_2Cl_2 (50 mL) and washed with saturated $Na_2S_2O_3$

(25 mL), saturated $NaHCO_3$ (25 mL), deionised H_2O (25 mL) and brine (25 mL). The organic layer was dried over anhydrous Na_2SO_4, filtered off and concentrated in *vacuo*. Purification by column chromatography (pentane: EtOAc; 9:1 to 8:2) gave product **1l** as a yellowish syrup (251 mg, 0.56 mmol, 56%, α/β 80:20). ^{1}H NMR (500 MHz, Chloroform-*d*) δ 8.02 (d, *J* = 7.8 Hz, 3H, Ar-CH), 7.62 – 7.54 (m, 2H, Ar-CH), 7.48 – 7.15 (m, 10H, Ar-CH), 5.50 (t, *J* = 9.7 Hz, 1H, H-4), 5.24 (s, 1H, H-1), 4.83 (d, *J* = 12.3 Hz, 1H, $PhCH_2$), 4.75 – 4.70 (m, 1H, $PhCH_2$), 4.56 (d, *J* = 12.1 Hz, 1H, $PhCH_2$), 4.45 (d, *J* = 12.2 Hz, 1H, $PhCH_2$), 4.14 – 4.06 (m, 1H, H-5), 3.99 (dd, *J* = 9.7, 2.8 Hz, 1H, H-3), 3.87 (d, *J* = 2.4 Hz, 1H, H-2), 2.89 (d, *J* = 3.5 Hz, 1H, OH), 1.25 (d, *J* = 6.4 Hz, 3H, H-6). NMR data are consistent with the literature.[22]

2,3,4-Tri-*O*-acetyl-L-rhamnoside S35

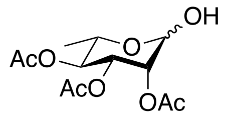

Crude rhamnoside **S27** (1 g, 3 mmol) was dissolved in a 9:1 mixture of acetone:water (30 mL) and NBS (1.6 g, 9.0 mmol) was added. The reaction was left to stir at room temperature overnight. TLC analysis (cyclohexane: EtOAc; 1:1; R_f = 0.4) showed the complete conversion of the starting material to the desired product. Purification by column chromatography (pentane: EtOAc; 1:1) gave product **S35** as a white solid (400 mg, 1.38 mmol, 46% α/β 89:11). ^{1}H NMR (500 MHz, Chloroform-*d*) δ 5.37 (dd, *J* = 10.1, 3.4 Hz, 1H, H-3), 5.26 (dd, *J* = 3.2, 2.0 Hz, 1H, H-2), 5.15 (br s, 1H, H-1), 5.07 (t, *J* = 10.0 Hz, 1H, H-4), 4.18 – 4.09 (m, 1H, H-5), 3.86 (s, 1H, OH), 2.17 – 2.15 (m, 3H, CH_3), 2.06 (s, 3H, CH_3), 2.01 (s, 3H, CH_3), 1.22 (d, *J* = 6.3 Hz, 3H, H-6). ^{13}C NMR (151 MHz, Chloroform-*d*) δ 170.4 (C=O), 170.23 (C=O), 170.17 (C=O), 92.00 (C-1), 71.1 (C-4), 70.4 (C-2), 68.9 (C-3), 66.3 (C-5), 20.88 (CH_3), 20.8 (CH_3), 20.7 (CH_3), 17.4 (C-6). NMR data are consistent with the literature.[23]

Synthesis of Acceptors

Methyl 2,3,4-tri-*O*-benzyl-α-D-glucopyranoside 3a

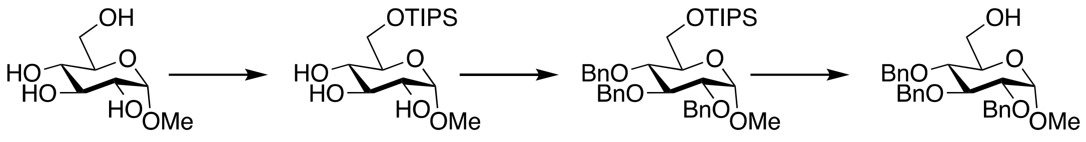

Under a N_2 atmosphere, methyl α-D-glucopyranoside (3.66 g, 18.9 mmol) was dissolved in anhydrous DMF (30 mL) and imidazole (3.86 g, 56.6 mmol) was added. TIPSCl (4.43 mL, 20.7 mmol) was added dropwise over a period of 15 minutes. After-stirring the reaction at RT for 24 h, it was diluted with water (100 mL) and extracted with CH_2Cl_2 (3 × 60 mL). The combined organic layer was washed with brine, dried over anhydrous Na_2SO_4, filtered and concentrated *in vacuo.*

A solution of the crude product **S36** was dissolved in anhydrous DMF (100 mL) and the flask was cooled to 0 °C. NaH (60% dispersion in mineral oil) (3.77 g, 94.3 mmol) was added to the solution and the ice-bath was removed. The reaction was stirred at room temperature for 1 h, after which it was again cooled to 0 °C and treated slowly with BnBr (11.2 mL, 94.3 mmol). The ice-bath was removed, and the reaction mixture was left to stir at room temperature. TLC analysis (cyclohexane:EtOAc; 9:1) after 12 h showed complete consumption of starting material. The reaction mixture was quenched with MeOH (10 mL) and was extracted wit Et_2O (3 × 200 mL). The combined organic layer was washed with 1 M HCl (100 mL) followed by saturated $NaHCO_3$ (100 mL) and brine (30 mL). The organic layer was dried over anhydrous Na_2SO_4, filtered and concentrated *in vacuo* to give a yellow oil.

The crude product **S37** was dissolved in MeOH (10 mL) and 1.25 M HCl in MeOH (10 mL) was added. The reaction was left to stir over the weekend, after which TLC analysis (cyclohexane:EtOAc; 9:1, R_f= 0.1) showed that the reaction had gone to completion. The reaction mixture was quenched with saturated $NaHCO_3$ (20 mL), diluted with CH_2Cl_2 (250 mL) and the two phases were separated. The organic phase was washed with saturated $NaHCO_3$ (50 mL), water (50 mL), brine (50 mL), dried over anhydrous Na_2SO_4, filtered and concentrated *in vacuo* to give a yellow oil. Purification by column chromatography (pentane:EtOAc; 98:2 to 90:10 to 80:20) afforded the desired product **3a** as a white solid (6.0 g, 12.9 mmol, 68% over 3 steps).
R_f= 0.4 (pentane:EtOAc; 4:1; H_2SO_4 (15-20% EtOH) stain); ^{1}H NMR (500 MHz, Chloroform-*d*): δ 7.40 – 7.25 (m, 15H, Ar-CH), 4.99 (d, *J* = 10.9 Hz, 1H, $PhCH_2$), 4.91 – 4.77 (m, 3H, 3 x $PhCH_2$), 4.70 – 4.63 (m, 2H, 2 x $PhCH_2$), 4.57 (d, *J* = 3.5 Hz, 1H, H-1), 4.00 (t, *J* = 9.3 Hz, 1H, H-3), 3.80 – 3.62 (m, 3H, H-6a, H-6b, H-5), 3.56 – 3.47 (m, 2H, H-4, H-2), 3.37 (s, 1H, OCH_3), 1.64 (br s, 1H, OH). ^{13}C NMR (126

MHz, Chloroform-*d*): δ 138.8 (4º C), 138.2 (4º C), 138.1 (4º C), 128.50 (Ar-*C*H), 128.49 (Ar-*C*H), 128.4 (Ar-*C*H), 128.13 (Ar-*C*H), 128.05 (Ar-*C*H), 127.98 (Ar-*C*H), 127.96 (Ar-*C*H), 127.89 (Ar-*C*H), 127.6 (Ar-*C*H), 98.2 (C-1), 82.0 (C-3), 78.0 (C-2), 77.4 (C-4), 75.8 (Ph*C*H_2), 75.1 (Ph*C*H_2), 73.5 (Ph*C*H_2), 70.7 (C-5), 61.9 (C-6), 55.2 (O*C*H_3). NMR data are consistent with the literature.[24]

Methyl 4,6-*O*-benzylidene-α-D-glucopyranoside S38

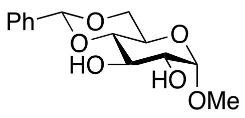

Under a N_2 atmosphere, a solution of methyl-α-D-glucopyranoside (5.0 g, 26 mmol) in anhydrous DMF (52 mL) was treated with TsOH.H_2O (0.25 g, 1.3 mmol), followed by benzaldehyde dimethyl acetal (4.6 mL, 31 mmol) and stirred at 60 °C for 18 h. The reaction mixture was concentrated *in vacuo*, re-dissolved in CH_2Cl_2, washed with saturated NaHCO3 and brine, dried over anhydrous MgSO4 and concentrated *in vacuo* to give a yellow slurry. After purification by column chromatography (95:5 to 90:10; CH_2Cl_2/MeOH), the title compound **S38** was obtained as a white solid (5.0 g, 68% yield). ^{1}H NMR (500 MHz, Chloroform-*d*): δ 7.51 – 7.45 (m, 2H, ArCH), 7.43 – 3.31 (m, 3H, ArCH), 5.51 (s, 1H, PhC*H*), 4.74 (d, *J* = 3.9 Hz, 1H, H-1), 4.27 (dd, *J* = 9.9, 4.5 Hz, 1H, H-6a), 3.90 (td, *J* = 9.2, 2.0 Hz, 1H, H-3), 3.78 (td, *J* = 9.7, 4.5 Hz, 1H, H-5), 3.75 – 3.67 (m, 1H, H-6b), 3.59 (td, *J* = 9.0, 3.8 Hz, 1H, H-2), 3.46 (t, *J* = 9.4 Hz, 1H, H-4), 3.42 (s, 1H, OCH_3), 3.22 (d, *J* = 2.4 Hz, 1H, OH), 2.65 (d, *J* = 9.0 Hz, 1H, OH). ^{13}C NMR (126 MHz, Chloroform-*d*): δ 137.2 (C), 129.4 (CH), 128.4 (CH), 126.5 (CH), 102.1 (Ph*C*H), 99.9 (C-1), 81.1 (C-4), 72.9 (C-2), 71.7 (C-3), 69.0 (C-6), 62.5 (C-5), 55.7 (OCH_3). NMR data were consistent with literature data.[25]

Methyl 2,3-di-*O*-benzyl-4,6-*O*-benzylidene-α-D-glucopyranoside S39

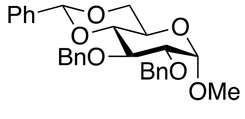

Under a N_2 atmosphere, a solution of glucopyranoside **S38** (5.0 g, 18 mmol) in anhydrous DMF (40 mL) was cooled to 0 °C and NaH (60% dispersion in mineral oil) (2.3 g, 58 mmol) was added. The reaction mixture was stirred at room temperature for 30 min after which it was again cooled down to 0

°C. Benzyl bromide (6.4 mL, 54 mmol) was added dropwise to the reaction mixture. The reaction mixture was left to stir at room temperature for 9 h. The reaction was quenched with MeOH and the mixture was concentrated *in vacuo* to give a yellow slurry. The slurry was diluted with CH_2Cl_2 and washed with 1 M HCl, followed by saturated $NaHCO_3$ and brine, dried over anhydrous $MgSO_4$ and concentrated *in vacuo*. Purification by column chromatography (90:10; Pentane/Et_2O) gave **S39** as a white solid (7.9 g, 95% yield). ^{1}H NMR (500 MHz, Chloroform-*d*): δ 7.52 – 7.46 (m, 2H, ArCH), 7.42 – 7.24 (m, 13H, ArCH), 5.55 (s, 1H, PhC*H*), 4.91 (d, *J* = 11.3 Hz, 1H, C*H*HPh), 4.85 (d, *J* = 12.2 Hz, 1H, C*H*HPh), 4.85 (d, *J* = 11.3 Hz, 1H, C*H*HPh), 4.70 (d, *J* = 12.2 Hz, 1H, C*H*HPh), 4.60 (d, *J* = 3.7 Hz, 1H, H-1), 4.26 (dd, *J* = 10.1, 4.8 Hz, 1H, H-6a), 4.05 (t, *J* = 9.3 Hz, 1H, H-3), 3.83 (td, *J* = 9.9, 4.8 Hz, 1H, H-5), 3.70 (t, *J* = 10.3 Hz, 1H, H-6b), 3.60 (t, *J* = 9.4 Hz, 1H, H-4), 3.56 (dd, *J* = 9.3, 3.7 Hz, 1H, H-2), 3.40 (s, 1H, OCH_3). ^{13}C NMR (126 MHz, Chloroform-*d*): δ 138.9 (C), 138.3 (C), 137.5 (C), 129.0 (CH), 128.6 (CH), 128.44 (CH), 128.35 (CH), 128.3 (CH), 128.2 (CH), 128.1 (CH), 127.7 (CH), 126.2 (CH), 101.4 (Ph*C*H), 99.4 (C-1), 82.3 (C-4), 79.3 (C-2), 78.7 (C-3), 75.5 (Ph*C*H_2), 73.9 (Ph*C*H_2), 69.2 (C-6), 62.5 (C-5), 55.5 (OCH_3). NMR data were consistent with literature data.[25]

Methyl 2,3,6-tri-*O*-benzyl-α-D-glucopyranoside 3b

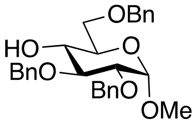

Based on the literature procedure,[6] under a N_2 atmosphere, a solution of **S39** (3.6 g, 7.8 mmol) in anhydrous CH_2Cl_2 (29 mL) was cooled to 0 °C and treated slowly with trifluoroacetic acid (3.6 mL, 47 mmol) followed by triethylsilane (7.4 mL, 47 mmol). The reaction mixture was warmed up to room temperature and stirred for 45 minutes. The reaction was diluted with CH_2Cl_2 and quenched with saturated $NaCHO_3$. The organic layer was washed with brine, dried over anhydrous $MgSO_4$ and concentrated *in vacuo* to give a yellow syrup. Purification by column chromatography (2:1; Pentane/Et_2O) gave **3b** as a yellowish syrup (3.0 g, 83% yield). ^{1}H NMR (500 MHz, Chloroform-*d*): δ 7.41 – 7.21 (m, 15H, ArCH), 5.00 (d, *J* = 11.5 Hz, 1H, C*H*HPh), 4.77 (d, *J* = 12.1 Hz, 1H, C*H*HPh), 4.73 (d, *J* = 11.4 Hz, 1H, C*H*HPh), 4.66 (d, *J* = 12.1 Hz, 1H, C*H*HPh), 4.63 (d, *J* = 3.5 Hz, 1H, H-1), 4.59 (d, *J* = 12.2 Hz, 1H, C*H*HPh), 4.54 (d, *J* = 12.1 Hz, 1H, C*H*HPh), 3.78 (t, *J* = 9.2 Hz, 1H, H-3), 3.73 – 3.68 (m, 1H, H-5), 3.68 – 3.65 (m, 2H, H-6a, H-6b), 3.60 (td, *J* = 9.2, 2.4 Hz, 1H, H-4), 3.53 (dd, *J* = 9.6, 3.5 Hz, 1H, H-2), 3.38 (s, 3H, OCH_3), 2.31 (t, *J* = 2.4 Hz, 1H, OH). ^{13}C NMR (126 MHz, Chloroform-*d*): δ 138.9 (C), 138.2 (C), 138.1 (C), 128.7 (CH), 128.6 (CH), 128.5 (CH), 128.3 (CH), 128.12 (CH), 128.08 (CH), 128.0 (CH), 127.76 (CH), 127.75 (CH), 98.3 (C-1), 81.6 (C-3), 79.7 (C-2),

75.6 (Ph*C*H$_2$), 73.7 (Ph*C*H$_2$), 73.3 (Ph*C*H$_2$), 70.9 (C-4), 70.0 (C-5), 69.6 (C-6), 55.4 (OCH_3). NMR data were consistent with literature data.[8]

Methyl 2-*O*-benzyl-4,6-*O*-benzylidene-α-D-glucopyranoside 3d and Methyl 3-*O*-benzyl-4,6-*O*-benzylidene-α-D-glucopyranoside S40

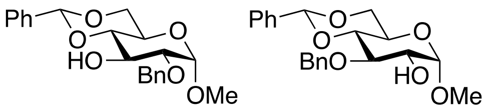

A solution of glucopyranoside **S38** (6.7 g, 24 mmol) in anhydrous CH_2Cl_2 (240 mL) was treated with Bu_4NHSO_4 (4.1 g, 12 mmol), followed by benzyl bromide (3.4 mL, 29 mmol) and 1 M NaOH (80 mL) at room temperature. The reaction mixture was then left to stir at reflux for 41 h. The organic and aqueous layers were separated and the aqueous layer was washed with CH_2Cl_2. The combined organic layers were dried over anhydrous $MgSO_4$ and concentrated *in vacuo* to give a white solid. Purification by column chromatography (90:10; Pentane/Et_2O) gave **3d** and **S40** as white solid (6.6 g, 74% yield, **3d**:**S40** = 2:1).

3d (3-OH) (R_f = 0.38; 2:1 cyclohexane/EtOAc)

^{1}H NMR (400 MHz, Chloroform-*d*): δ 7.61 – 7.43 (m, 2H, ArCH), 7.43 – 7.26 (m, 8H, ArCH), 5.52 (s, 1H, PhC*H*), 4.79 (d, *J* = 12.2 Hz, 1H, C*H*HPh), 4.70 (d, *J* = 12.1 Hz, 1H, C*H*HPh), 4.62 (d, *J* = 3.6 Hz, 1H, H-1), 4.26 (dd, *J* = 10.1, 4.7 Hz, 1H, H-6a), 4.15 (td, *J* = 9.3, 2.2 Hz, 1H, H-3), 3.81 (td, *J* = 9.9, 4.7 Hz, 1H, H-5), 3.70 (t, *J* = 10.2 Hz, 1H, H-6b), 3.49 (t, *J* = 9.4 Hz, 1H, H-4), 3.47 (dd, J = 9.2, 3.6 Hz, 1H, H-2), 3.38 (s, 3H, OCH_3), 2.55 (d, *J* = 2.2 Hz, 1H, OH). ^{13}C NMR (101 MHz, Chloroform-*d*): δ 138.1 (C), 137.2 (C), 129.4 (CH), 128.7 (CH), 128.5 (CH), 128.30 (CH), 128.27 (CH), 126.5 (CH), 102.1 (Ph*C*H), 98.8 (C-1), 81.4 (C-4), 79.7 (C-2), 73.5 (Ph*C*H$_2$), 70.4 (C-3), 69.2 (C-6), 62.2 (C-5), 55.5 (OCH_3). NMR data were consistent with literature data.[25]

S40 (2-OH) (R_f = 0.25 ; 2:1 cyclohexane/EtOAc)

^{1}H NMR (500 MHz, Chloroform-*d*): δ 7.55 – 7.44 (m, 2H, ArCH), 7.44 – 7.14 (m, 8H, ArCH), 5.57 (s, 1H, PhC*H*), 4.96 (d, *J* = 11.6 Hz, 1H, C*H*HPh), 4.81 (d, *J* = 3.9 Hz, 1H, H-1), 4.79 (d, *J* = 11.6 Hz, 1H, C*H*HPh), 4.30 (dd, *J* = 10.0, 4.6 Hz, 1H, H-6a), 3.88 – 3.79 (m, 2H, H-3, H-5), 3.79 – 3.70 (m, 2H, H-6b, H-2), 3.64 (t, *J* = 9.2 Hz, 1H, H-4), 3.45 (s, 3H, OCH_3), 2.30 (d, *J* = 7.4 Hz, 1H, OH). ^{13}C NMR (126 MHz, Chloroform-*d*): δ 138.6 (C), 137.5 (C), 129.1 (CH), 128.5 (CH), 128.4 (CH), 128.1 (CH), 127.9 (CH), 126.2 (CH), 101.4 (Ph*C*H), 100.01 (C-1), 82.1 (C-4), 79.0 (C-3), 75.0 (Ph*C*H$_2$), 72.6 (C-2), 69.2 (C-6), 62.7 (C-5), 55.6 (OCH_3). NMR data were consistent with literature data.[25]

Methyl 3-*O*-acetyl-2-*O*-benzyl-4,6-*O*-benzylidene-α-D-glucopyranoside S41 and Methyl 2-*O*-acetyl-3-*O*-benzyl-4,6-*O*-benzylidene-α-D-glucopyranoside S42

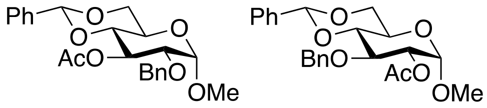

A solution of **3d** and **S40** (6.6 g, 18 mmol), acetic anhydride (3.4 mL, 36 mmol) and DMAP (22 mg, 0.18 mmol) in pyridine (1.4 mL, 18 mmol) (little bit of CH_2Cl_2 was added to get a clear solution) was stirred at room temperature for 4 h. The reaction mixture was diluted with CH_2Cl_2 and washed with 1 M HCl, saturated $NaHCO_3$, and brine. The organic layer was dried over anhydrous $MgSO_4$, filtered and concentrated *in vacuo* to give a white solid. The crude material was used in the next step without further purification.

S41 (3-OAc)

^{1}H NMR (600 MHz, Chloroform-*d*): δ 7.46 – 7.41 (m, 2H, ArCH), 7.40 – 7.28 (m, 8H, ArCH), 5.56 (t, *J* = 9.7 Hz, 1H, H-3), 5.45 (s, 1H, PhC*H*), 4.69 (d, *J* = 3.5 Hz, 1H, H-1), 4.66 (d, *J* = 12.4 Hz, 1H, C*H*HPh), 4.63 (d, *J* = 12.4 Hz, 1H, C*H*HPh), 4.26 (dd, *J* = 10.3, 4.9 Hz, 1H, H-6a), 3.89 (td, *J* = 10.0, 4.9 Hz, 1H, H-5), 3.70 (t, *J* = 10.3 Hz, 1H, H-6b), 3.57 (dd, *J* = 9.7, 3.6 Hz, 1H, H-2), 3.53 (t, *J* = 9.6 Hz, 1H, H-4), 3.402 (s, 3H, OCH_3), 2.04 (s, 3H, CH_3). ^{13}C NMR (151 MHz, Chloroform-*d*): δ 169.8 (C=O), 138.0 (C), 137.2 (C), 129.1 (CH), 128.6 (CH), 128.3 (CH), 128.1 (CH), 128.0 (CH), 126.3 (CH), 101.6 (Ph*C*H), 98.9 (C-1), 79.7 (C-4), 77.8 (C-2), 73.14 (Ph*C*H_2), 70.7 (C-3), 69.12 (C-6), 62.48 (C-5), 55.5 (OCH_3), 21.14 (CH_3). NMR data were consistent with literature data.[26]

S42 (2-OAc)

^{1}H NMR (600 MHz, Chloroform-*d*): δ 7.51 – 7.47 (m, 2H, ArCH), 7.40 – 7.28 (m, 8H, ArCH), 5.59 (s, 1H, PhC*H*), 4.93 – 4.86 (m, 3H, H-1, H-2, C*H*HPh), 4.71 (d, *J* = 11.2 Hz, 1H, C*H*HPh), 4.30 (dd, *J* = 10.1, 4.7 Hz, 1H, H-6a), 4.03 (t, *J* = 9.4 Hz, 1H, H-3), 3.88 – 3.83 (m, 1H, H-5), 3.78 (t, *J* = 10.3 Hz, 1H, H-6b), 3.75 – 3.68 (m, 1H, H-4), 3.396 (s, 3H, OCH_3), 2.08 (s, 3H, OCH_3). ^{13}C NMR (151 MHz, Chloroform-*d*) δ 170.5 (C=O), 138.6 (C), 137.4 (C), 128.41 (CH), 128.37 (CH), 127.8 (CH), 127.7 (CH), 126.2 (CH), 101.5 (Ph*C*H), 97.9 (C-1), 82.2 (C-4), 76.3 (C-3), 75.0 (Ph*C*H_2), 73.12 (C-2), 69.08 (C-6), 62.45 (C-5), 55.4 (OCH_3), 21.05 (CH_3). NMR data were consistent with literature data.[27]

Methyl 3-*O*-acetyl-2,6-di-*O*-benzyl-α-D-glucopyranoside S43 and Methyl 2-*O*-acetyl-3,6-di-*O*-benzyl-α-D-glucopyranoside S44

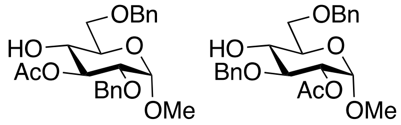

Under a N_2 atmosphere, a solution of **S41** and **S42** (6.7 g, 16 mmol) in anhydrous CH_2Cl_2 (59 mL) was cooled to 0 °C and treated slowly with trifluoroacetic acid (7.4 mL, 96 mmol) followed by triethylsilane (15 mL, 96 mmol). The reaction mixture was warmed up to room temperature and stirred for 1 h. The reaction was diluted with CH_2Cl_2 and quenched with saturated $NaCHO_3$. The organic layer was washed with brine, dried over anhydrous $MgSO_4$ and concentrated *in vacuo* to give a yellowish syrup. Purification by column chromatography (3:1 to 1:1; Pentane/Et_2O) gave **S43** as a colourless syrup (3.2 g, 48% yield) and **S44** as a colourless syrup (0.98 g, 15% yield).

S43 (3-OAc)

^{1}H NMR (500 MHz, Chloroform-*d*): δ 7.38 – 7.25 (m, 10H, ArCH), 5.23 (t, *J* = 9.5 Hz, 1H, H-3), 4.68 (d, *J* = 3.6 Hz, 1H, H-1), 4.66 (d, *J* = 12.4 Hz, 1H, C*H*HPh), 4.62 (d, *J* = 12.4 Hz, 1H, C*H*HPh), 4.60 (d, *J* = 12.1 Hz, 1H, C*H*HPh), 4.56 (d, *J* = 12.1 Hz, 1H, C*H*HPh), 3.77 – 3.67 (m, 3H, H-4, H-6a, H-6b), 3.64 (td, *J* = 9.3, 4.7 Hz, 1H, H-5), 3.53 (dd, *J* = 9.9, 3.6 Hz, 1H, H-2), 3.38 (s, 3H, OCH_3), 2.79 (d, *J* = 4.7 Hz, 1H, OH), 2.09 (s, 3H, CH_3). ^{13}C NMR (126 MHz, Chloroform-*d*): δ 172.4 (C=O), 138.1 (C), 138.0 (C), 128.6 (CH), 128.5 (CH), 128.1 (CH), 128.0 (CH), 127.9 (CH), 127.8 (CH), 98.0 (C-1), 76.8 (C-2), 75.8 (C-3), 73.8 (PhCH_2), 73.2 (PhCH_2), 70.6 (C-5), 70.4 (C-4), 69.4 (C-6), 55.4 (OCH_3), 21.2 (CH_3). ESI-HRMS for $C_{23}H_{28}O_7Na^+$ $(M+Na)^+$ calculated: 439.1727; found: 439.1729.

S44 (2-OAc)

^{1}H NMR (500 MHz, Chloroform-*d*): δ 7.37 – 7.26 (m, 10H, ArCH), 4.91 (d, *J* = 3.7 Hz, 1H, H-1), 4.85 (dd, *J* = 9.9, 3.7 Hz, 1H, H-2), 4.80 (d, *J* = 11.7 Hz, 1H, C*H*HPh), 4.73 (d, *J* = 11.7 Hz, 1H, C*H*HPh), 4.62 (d, *J* = 12.1 Hz, 1H, C*H*HPh), 4.56 (d, *J* = 12.1 Hz, 1H, C*H*HPh), 3.84 (dd, *J* = 9.9, 8.5 Hz, 1H, H-3), 3.78 – 3.67 (m, 4H, H-4, H-5, H-6a, H-6b), 3.39 (s, 3H, OCH_3), 2.49 (d, *J* = 2.5 Hz, 1H, OH), 2.07 (s, 3H, CH_3). ^{13}C NMR (126 MHz, Chloroform-*d*): δ 170.4 (C=O), 138.7 (C), 138.0 (C), 128.7 (CH), 128.6 (CH), 128.0 (CH), 127.9 (CH), 127.80 (CH), 127.75 (CH), 97.3 (C-1), 79.9 (C-3), 75.3 (PhCH_2), 73.8 (PhCH_2), 73.5 (C-2), 71.6 (C-4/5), 69.9 (C-4/5 and C-6), 55.3 (OCH_3), 21.1 (CH_3). NMR data were consistent with literature data.[28]

Methyl 2,4,6-tri-*O*-benzyl-α-D-glucopyranoside 3c

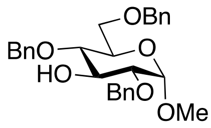

Under a N_2 atmosphere, a solution of glucopyranoside **S43** (2.1 g, 5.0 mmol) in anhydrous DMF (17 mL) was cooled to 0 °C and benzyl bromide (0.72 mL, 6.1 mmol) was added. After 15 minutes, NaH (60% dispersion in mineral oil) (242 g, 6.05 mmol) was added in one portion and the reaction mixture was stirred at 0 °C for 30 minutes. The reaction mixture was warmed to room temperature and stirred for 3.5 h. The reaction was quenched with MeOH and the mixture was concentrated *in vacuo* to give a yellowish slurry. The slurry was diluted with CH_2Cl_2 and washed with 1 M HCl, followed by saturated $NaHCO_3$ and brine, dried over anhydrous $MgSO_4$ and concentrated *in vacuo*. A solution of the crude in MeOH was treated with MeONa (0.14 g, 2.5 mmol) and the mixture was left to stir at room temperature for 3 days. Purification by column chromatography (98:2 to 95:5; CH_2Cl_2/Et_2O) gave **3c** as a colourless syrup (1.3 g, 56% yield over 2 steps). ^{1}H NMR (500 MHz, Chloroform-*d*): δ 7.38 – 7.25 (m, 13H, ArCH), 7.24 – 7.18 (m, 2H, ArCH), 4.83 (d, *J* = 11.1 Hz, 1H, C*H*HPh), 4.70 (d, *J* = 12.2 Hz, 1H, C*H*HPh), 4.67 (d, *J* = 12.1 Hz, 1H, C*H*HPh), 4.65 (d, *J* = 3.5 Hz, 1H, H-1), 4.61 (d, *J* = 12.1 Hz, 1H, C*H*HPh), 4.52 (d, *J* = 11.2 Hz, 1H, C*H*HPh), 4.49 (d, *J* = 12.2 Hz, 1H, C*H*HPh), 4.07 (ddd, *J* = 9.5, 8.7, 2.2 Hz, 1H, H-3), 3.74 – 3.69 (m, 2H, H-5, H-6a), 3.67 – 3.62 (m, 1H, H-6b), 3.58 – 3.51 (m, 1H, H-4), 3.41 (dd, *J* = 9.6, 3.5 Hz, 1H, H-2), 3.33 (s, 3H, OCH_3), 2.42 (d, *J* = 2.2 Hz, 1H, OH). ^{13}C NMR (126 MHz, Chloroform-*d*): δ 138.6 (C), 138.13 (C), 138.07 (C), 128.7 (CH), 128.52 (CH), 128.51 (CH), 128.3 (CH), 128.2 (CH), 128.1 (CH), 128.0 (CH), 127.9 (CH), 127.8 (CH), 97.7 (C-1), 79.6 (C-2), 77.6 (C-4), 74.7 (Ph*C*H_2), 73.8 (C-3), 73.7 (Ph*C*H_2), 73.2 (Ph*C*H_2), 69.8 (C-5), 68.7 (C-6), 55.3 (OCH_3). NMR data were consistent with literature data.[29]

Methyl 4,6-*O*-benzylidene-α-D-galactopyranoside S45

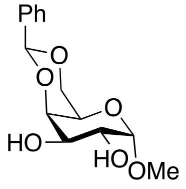

Following the literature procedure,[30] a solution of methyl α-D-galactopyranose (1.1 g, 5.7 mmol), benzaldehyde dimethyl acetal (1.0 ml, 6.7 mmol) and *p*TsOH.H_2O (0.02 g, 0.1 mmol, 2 mol%) in anhydrous DMF (12 ml) was heated on a rotary-evaporator (50 °C, 250 mbar) for four hours. TLC

analysis (9:1; ethyl acetate/methanol; H_2SO_4 (10-15% EtOH)) of the reaction mixture against a sample of pure product showed the desired product had been formed (R_f = 0.64) and some starting material remained (R_f = 0.49, faint spot). The DMF was removed using rotary evaporation. The white solid obtained was dissolved in CH_2Cl_2 (20 ml) and washed with saturated $NaHCO_3$ solution (20 ml). The aqueous layer was extracted with CH_2Cl_2 (3 × 20 ml). The combined organic layers were washed with brine (20 ml) and dried over Na_2SO_4, filtered and concentrated *in vacuo*. Purification by column chromatography (95:5; dichloromethane/methanol) gave the desired product **S45** as a white solid (0.87 g, 54% yield); R_f = 0.24 (95:5; CH_2Cl_2/MeOH), mp 172−174 °C (lit.[30] 170.3−171.0 °C (cyclohexane/ethyl acetate)); ^{1}H NMR (400 MHz, Chloroform-*d*) δ 7.58 – 7.44 (m, 2H, Ar-CH), 7.44 – 7.30 (m, 3H, Ar-CH), 5.54 (s, 1H, PhC*H*), 4.91 (d, *J* = 3.1 Hz, 1H, H-1), 4.27 (dd, *J* = 12.5, 1.6 Hz, 1H, H-6a), 4.24 (br d, *J* = 2.0 Hz, 1H, H-4), 4.06 (dd, *J* = 12.6, 1.8 Hz, 1H, H-6b), 3.99 – 3.83 (m, 2H, H-2, H-3), 3.67 (br s, 1H, H-5), 3.44 (s, 3H, OCH_3), 2.64 (d, *J* = 7.3 Hz, 1H, OH), 2.42 (d, *J* = 6.3 Hz, 1H, OH); ^{13}C NMR (101 MHz, Chloroform-*d*) δ 137.7 (4 °C), 129.3 (Ar-*C*H), 128.4 (Ar-*C*H), 126.4 (Ar-*C*H), 101.4 (Ph*C*H), 100.4 (C-1), 76.0 (C-4), 69.9, 69.8 (C-2, C-3), 69.4 (C-6), 62.8 (C-5), 55.8 (OCH_3); ESI-HRMS for $C_{14}H_{18}NaO_6^+$ $(M+Na)^+$ calculated: 305.1001; found: 305.0986. NMR data were consistent with the literature.[30]

Methyl 2-*O*-benzyl-3-*O*-acetyl-4,6-*O*-benzylidene-α-D-galactopyranoside S47 and Methyl 3-*O*-benzyl-2-*O*-acetyl-4,6-*O*-benzylidene-α-D-galactopyranoside S48

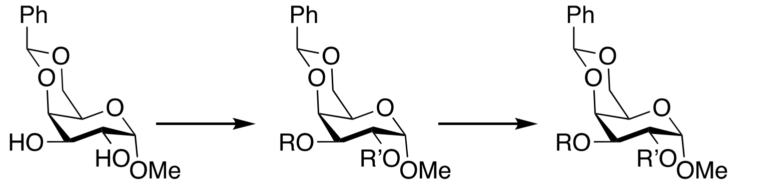

S45

S46: R=H, R'=Bn
3h: R=Bn, R'=H
S46/3h
54:46

S47: R=Ac, R'=Bn
S48: R=Bn, R'=Ac

Based on the literature procedure,[30] ^{n}Bu$_4$NHSO$_4$ (16 g, 47 mmol) followed by aq. NaOH (150 mL, 1.2M) were added to a stirred solution of methyl 4,6-*O*-benzylidene-α-D-galactopyranoside **S45** (13.5 g, 47.8 mmol) in CH_2Cl_2 (500 mL). The mixture was stirred for 30 min at room temperature. BnBr (7.0 mL, 59 mmol) was then added to the reaction mixture. The reaction was heated at reflux temperature for three days (time unoptimised). TLC analysis (9:1; dichloromethane/methanol) showed that some of the galactopyranoside starting material remained (R_f = 0.45) and two new spots were detected in the reaction mixture (R_f = 0.82, 0.86). The aqueous and organic layers were separated. The aqueous layer was extracted with CH_2Cl_2 (2 × 150 mL). The combined organic layers were washed with $NaHCO_3$ (200

mL), brine (200 mL), dried over $MgSO_4$, filtered and concentrated *in vacuo*. Column chromatography was performed (99:1 to 90:10; cyclohexane/ EtOAc) which afforded a mixture of methyl 2-*O*-benzyl-4,6-*O*-benzylidene-α-D-galactopyranoside **S46** and methyl 3-*O*-benzyl-4,6-*O*-benzylidene-α-D-galactopyranoside **3j** as a white solid (12 g, 67% yield). Based on the relative integrations of PhC*H* in the ^{1}H NMR spectrum the ratio of the two products was determined as 54:46 (**S46**:**3j**, 3-OH/2-OH product). The mixture of **S46** and **3j** (12 g, 32 mmol) was added to a solution of acetic anhydride (12.0 mL, 127 mmol) and anhydrous pyridine (50 mL), under a N_2 atmosphere. The reaction mixture was stirred at room temperature for three days (time unoptimised). TLC analysis (EtOAc) showed that the starting materials had been consumed (R_f = 0.53) and a new spot appeared (R_f = 0.66). The reaction mixture was diluted with CH_2Cl_2 (200 mL) and washed with 1M HCl (2 × 100 mL), brine (100 mL), dried over anhydrous Na_2SO_4, filtered and concentrated *in vacuo*. Purification by column chromatography (95:5 to 60:40; cyclohexane/ EtOAc) afforded **S47 (3-OAc)** as an amorphous white solid (6.9 g, 52% yield) and **S48 (2-OAc)** as a white solid (3.4 g, 26% yield); 1.6 g (12% yield) remained as mixed fractions.

Methyl 2-*O*-benzyl-3-*O*-acetyl-4,6-*O*-benzylidene-α-D-galactopyranoside S47: R_f = 0.3 (4:1; cyclohexane/ EtOAc); ^{1}H NMR (400 MHz, Chloroform-*d*) δ 7.52 – 7.45 (m, 2H, Ar-CH), 7.43 – 7.27 (m, 8H, Ar-CH), 5.50 (s, 1H, PhC*H*), 5.29 (dd, *J* = 10.5, 3.5 Hz, 1H, H-3), 4.80 (d, *J* = 3.5 Hz, 1H, H-1), 4.75 (d, *J* = 12.2 Hz, 1H, $PhCH_2$), 4.62 (d, *J* = 12.2 Hz, 1H, $PhCH_2$), 4.46 (dd, *J* = 3.6, 1.2 Hz, 1H, H-4), 4.23 (dd, *J* = 12.5, 1.6 Hz, 1H, H-6a), 4.09 (dd, *J* = 10.5, 3.5 Hz, 1H, H-2), 4.04 (dd, *J* = 12.5, 1.8 Hz, 1H, H-6b), 3.75 – 3.69 (m, 1H, H-5), 3.40 (s, 3H, OCH_3), 2.09 (s, 3H, ($H_3CC(O)O$)); ^{13}C NMR (101 MHz, Chloroform-*d*) δ 170.8 (C=O), 138.4 (4 °C), 137.9 (4 °C), 129.1 (Ar-*C*H), 128.5 (Ar-*C*H), 128.3 (Ar-*C*H), 127.9 (Ar-*C*H), 126.3 (Ar-*C*H), 100.9 (Ph*C*H), 99.3 (C-1), 74.4 (C-4), 73.7, 73.6 (C-2, Ph*C*H_2), 71.0 (C-3), 69.4 (C-6), 62.2 (C-5), 55.7 (OCH_3), 21.3 (H_3*C*C(O)O); ESI-HRMS for $C_{23}H_{26}NaO_7^+$ $(M+Na)^+$ calculated: 437.1576; found: 437.1561. NMR data were consistent with literature data.[30]

Methyl 3-*O*-benzyl-2-*O*-acetyl-4,6-*O*-benzylidene-α-D-galactopyranoside S48: R_f = 0.27 (4:1; cyclohexane/ EtOAc); ^{1}H NMR (500 MHz, Chloroform-*d*) δ 7.61 – 7.47 (m, 2H, Ar-CH), 7.41 – 7.22 (m, 8H, Ar-CH), 5.48 (s, 1H, PhC*H*), 5.34 (dd, *J* = 10.5, 3.6 Hz, 1H, H-2), 5.06 (d, *J* = 3.6 Hz, 1H, H-1), 4.72 (d, *J* = 12.5 Hz, 1H, $PhCH_2$), 4.68 (d, *J* = 12.5 Hz, 1H, $PhCH_2$), 4.24 (dd, *J* = 12.4, 1.6 Hz, 1H, H-6a), 4.20 (br d, *J* = 3.3 Hz, 1H, H-4), 4.04 – 3.95 (m, 2H, H-3, H-6b), 3.63 – 3.56 (m, 1H, H-5), 3.38 (s, 3H, OCH_3), 2.09 (s, 3H, ($H_3CC(O)O$)); ^{13}C NMR (126 MHz, Chloroform-*d*) δ 170.4 (C=O), 138.5 (4 °C), 137.8 (4 °C), 129.0 (Ar-*C*H), 128.4 (Ar-*C*H), 128.2 (Ar-*C*H), 127.7 (Ar-*C*H), 127.5 (Ar-*C*H), 126.4 (Ar-*C*H), 101.1 (Ph*C*H), 98.0 (C-1), 74.3 (C-4), 73.7 (C-3), 71.9 (Ph*C*H_2), 70.2 (C-2), 69.3 (C-6), 62.5 (C-5), 55.5 (OCH_3), 21.1 (H_3*C*C(O)O); ESI-HRMS for $C_{23}H_{26}NaO_7^+$ $(M+Na)^+$ calculated: 437.1576; found: 437.1598.

Methyl 2-*O*-benzyl-4,6-*O*-benzylidene-α-D-galactopyranoside S46

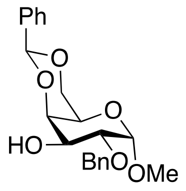

Using Zemplén conditions,[31] **S47** (1.5 g, 3.6 mmol) was dissolved in MeOH (36 mL) and NaOMe was added (0.08 g, 1.5 mmol). After 24 h TLC analysis (5:3; cyclohexane/ EtOAc) showed the starting material was consumed (R_f = 0.35) and a new spot had appeared (R_f = 0.09) in the reaction mixture. The mixture was neutralised with Amberlyst® IR 120, filtered and concentrated *in vacuo*. Purification by column chromatography (62:38 to 24:75; cyclohexane/ EtOAc) afforded the desired product **S46** as a white foam (1.12 g, 84% yield). R_f = 0.09 (5:3; cyclohexane/ EtOAc); ^{1}H NMR (400 MHz, Chloroform-*d*) δ 7.55 – 7.43 (m, 2H, Ar-CH), 7.41 – 7.25 (m, 8H, Ar-CH), 5.55 (s, 1H, PhC*H*), 4.81 (d, *J* = 12.3 Hz, 1H, PhCH$_2$), 4.79 (d, *J* = 3.6 Hz, 1H, H-1), 4.66 (d, *J* = 12.1 Hz, 1H, PhCH$_2$), 4.28 (dd, *J* = 3.9, 1.3 Hz, 1H, H-4), 4.25 (dd, *J* = 12.5, 1.6 Hz, 1H, H-6a), 4.14 (br dd, *J* = 10.1, 3.6 Hz, 1H, H-3), 4.07 (dd, *J* = 12.6, 1.9 Hz, 1H, H-6b), 3.82 (dd, *J* = 10.0, 3.5 Hz, 1H, H-2), 3.71 – 3.64 (m, 1H, H-5), 3.37 (s, 3H, OCH$_3$), 2.41 (s, 1H, OH); ^{13}C NMR (101 MHz, Chloroform-*d*) δ 138.4 (4° C), 137.7 (4° C), 129.3 (Ar-*C*H), 128.6 (Ar-*C*H), 128.3 (Ar-*C*H), 128.2 (Ar-*C*H), 128.0 (Ar-*C*H), 126.4 (Ar-*C*H), 101.4 (Ph*C*H), 99.1 (C-1), 76.9 (C-2), 76.2 (C-4), 73.5 (Ph*C*H$_2$), 69.5 (C-6), 68.7 (C-3), 62.5 (C-5), 55.7 (OCH$_3$); ESI-HRMS for $C_{21}H_{24}NaO_6^+$ (M+Na)$^+$ calculated: 395.1471; found: 395.1467. ^{1}H NMR data were consistent with the literature.[30]

Methyl 3-*O*-benzyl-4,6-*O*-benzylidene-α-D-galactopyranoside 3j

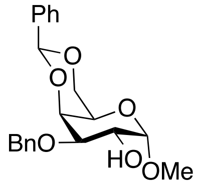

Using Zemplén conditions,[31] **S48** (1.5 g, 3.6 mmol) was dissolved in MeOH (36 mL) and NaOMe was added (0.09 g, 1.7 mmol). As the reaction proceeded a white precipitate formed. After 24 h this was filtered using Hirsch filtration. The filter cake was washed with cold MeOH. Desired product **3j** was obtained as a white solid without further purification (0.8 g, 60% yield). A second crop was obtained

from the filtrate (0.4 g, 30% yield). mp 200–202 °C (lit.[32] 185–186 °C (CH_2Cl_2/hexane)); ^{1}H NMR (400 MHz, Chloroform-*d*) δ 7.58 – 7.45 (m, 2H, Ar-CH), 7.44 – 7.21 (m, 8H, Ar-CH), 5.45 (s, 1H, PhC*H*), 4.95 (d, *J* = 3.8 Hz, 1H, H-1), 4.73 (s, 2H, 2 × $PhCH_2$), 4.26 (dd, *J* = 12.5, 1.6 Hz, 1H, H-6a), 4.23 – 4.16 (m, 2H, H-2, H-4), 4.02 (dd, *J* = 12.4, 1.8 Hz, 1H, H-6b), 3.79 (dd, *J* = 10.0, 3.5 Hz, 1H, H-3), 3.65 – 3.57 (m, 1H, H-5), 3.44 (s, 3H, OCH_3), 2.26 (d, *J* = 6.2 Hz, 1H, OH); ^{13}C NMR (101 MHz, Chloroform-*d*) δ 138.5 (4 °C), 137.9 (4 °C), 129.0 (Ar-*C*H), 128.6 (Ar-*C*H), 128.3 (Ar-*C*H), 127.94 (Ar-*C*H), 127.92 (Ar-*C*H), 126.4 (Ar-*C*H), 101.1 (Ph*C*H), 100.3 (C-1), 76.6 (C-3), 73.7 (C-4), 71.5 (Ph*C*H_2), 69.6 (C-6), 68.1 (C-2), 62.9 (C-5), 55.7 (OCH_3); ESI-HRMS for $C_{21}H_{24}NaO_6^+$ $(M+Na)^+$ calculated: 395.1471; found: 395.1456. Proton NMR data were consistent with the literature data with the exception of the assignments for H-4 and H-5.[32]

Ethyl 2,3,4-tri-*O*-(4-methylbenzyl)-1-thio-*α*-D-mannopyranoside 3i

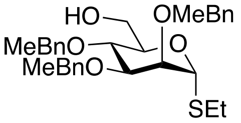

Based on the literature procedure,[8] a solution of **S8** (0.69 g, 1.0 mmol) in MeCN/H_2O (4:1, 15 mL) was treated with trifluoroacetic acid (0.940 mL, 12.2 mmol) at room temperature. After stirring for 4 h at room temperature, the reaction was quenched with saturated $NaHCO_3$ and diluted with CH_2Cl_2 (100 mL). The aqueous layer was washed with CH_2Cl_2 (2 x 50 mL). The combined organic layers were washed with brine, dried over anhydrous Na_2SO_4 and concentrated *in vacuo* to give a bright yellow oil. Purification by column chromatography (9:1 to 1:1; pentane/Et_2O) gave **3i** as a colourless syrup (0.54 g, quantitative yield). ESI-HRMS for $C_{32}H_{44}O_5SN^+$ $(M+NH_4)^+$ calculated: 554.2935; found: 554.2939. ^{1}H NMR (500 MHz, Chloroform-*d*): δ 7.27 – 7.18 (m, 6H, ArCH), 7.15 – 7.09 (m, 6H, ArCH), 5.26 (d, *J* = 1.3 Hz, 1H, H-1), 4.88 (d, *J* = 10.7 Hz, 1H, C*H*HPh), 4.67 (d, *J* = 12.2 Hz, 1H, C*H*HPh), 4.63 (d, *J* = 12.1 Hz, 1H, C*H*HPh), 4.58 (d, *J* = 10.7 Hz, 1H, C*H*HPh), 4.55 (d, *J* = 11.5 Hz, 1H, C*H*HPh), 4.52 (d, *J* = 11.6 Hz, 1H, C*H*HPh), 3.99 – 3.90 (m, 2H, H-4, H-5), 3.85 – 3.72 (m, 4H, H-2, H-3, H-6a, H-6b), 2.61 – 2.45 (m, 2H, SC*H*$_2$CH$_3$), 2.34 (s, 3H, CH_3), 2.34 (s, 3H, CH_3), 2.33 (s, 3H, CH_3), 1.24 – 1.17 (m, 4H, SCH$_2$C*H*$_3$, OH). ^{13}C NMR (126 MHz, Chloroform-*d*): δ 137.6 (C), 137.5 (C), 137.4 (C), 135.5 (C), 135.3 (C), 135.1 (C), 129.18 (CH), 129.16 (CH), 129.15 (CH), 128.3 (CH), 128.2 (CH), 128.0 (CH), 82.40 (C-1), 80.3 (C-3), 76.4 (C-2), 75.2 (Ph*C*H_2), 75.0 (C-4), 72.5 (C-5), 72.4 (Ph*C*H_2), 72.1 (Ph*C*H_2), 62.5 (C-6), 25.4 (S*C*H$_2$CH$_3$), 21.29 (CH_3), 21.28 (CH_3), 21.27 (CH_3), 15.0 (SCH$_2$*C*H$_3$).

Methyl 2,3,4-tri-*O*-benzoyl-α-D-glucopyranoside S49

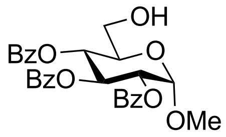

Methyl α-D-glucopyranoside (2.0 g, 10 mmol), was dissolved in anhydrous pyridine (34 mL) under a N2 atmosphere and treated with imidazole (1.40 g, 20.6 mmol) and TIPSCl (2.2 mL, 10 mmol). The reaction mixture was stirred at room temperature for 17 hours and monitored by TLC (9:1; CH_2Cl_2/methanol). When the starting material was consumed, BzCl (7.38 mL, 61.8 mmol) and catalytic DMAP (0.1 g, 1 mmol) were added and the reaction was stirred for 7.5 hours. The reaction mixture turned a yellow colour. TLC analysis (8:2; pentane/Et_2O) showed complete conversion of intermediate. The reaction was quenched with water (5 mL), diluted with CH_2Cl_2 (200 mL) and the two phases separated. The organic layer was subsequently washed with 1 M HCl, water, saturated $NaHCO_3$ and brine, dried over anhydrous $MgSO_4$, filtered and concentrated *in vacuo* to give a light brown syrup. The crude material was dissolved in CH_3CN and water (100 mL, 7:1) and treated with TFA (16 mL, 0.21 mol). The reaction mixture was stirred at room temperature for 21 hours and monitored by TLC analysis (8:2; pentane/Et_2O). The reaction mixture was concentrated *in vacuo* and the residue was diluted with CH_2Cl_2 (200 mL), washed with brine, dried over anhydrous MgSO4 and concentrated *in vacuo* to give a light brown syrup. Purification by column chromatography (1:1 pentane/Et_2O followed by 9:1 CH_2Cl_2/Et_2O) afforded the product **S49** (3.23 g, 48% yield) as a white crystalline solid. ^{1}H NMR (400 MHz, Chloroform-*d*): δ 8.01 – 7.94 (m, 4H, ArCH), 7.90 – 7.85 (m, 2H, ArCH), 7.57 – 7.48 (m, 2H, ArCH), 7.45 – 7.34 (m, 5H, ArCH), 7.32 – 7.24 (m, 2H, ArCH), 6.23 (t, *J* = 9.7 Hz, 1H, H-3), 5.50 (t, *J* = 9.9 Hz, 1H, H-4), 5.32 – 5.24 (m, 2H, H-2, H-1), 4.04 (ddd, *J* = 10.1, 3.8, 2.3 Hz, 1H, H-5), 3.83 (ddd, *J* = 13.0, 8.7, 2.3 Hz, 1H, H-6a), 3.74 (ddd, *J* = 12.9, 5.5, 3.8 Hz, 1H, H-6b), 3.47 (s, 3H, OCH_3), 2.68 (dd, *J* = 8.7, 5.6 Hz, 1H, OH). ^{13}C NMR (101 MHz, Chloroform-*d*): δ 166.6 (C=O), 166.0 (C=O), 165.9 (C=O), 133.9 (C), 133.5 (C), 133.3 (C), 130.13 (CH), 130.05 (CH), 129.8 (CH), 129.3 (CH), 129.2 (CH), 128.72 (CH), 128.66 (CH), 128.6 (CH), 128.4 (CH), 97.3 (C-1), 72.2 (C-2), 70.2 (C-3), 69.9 (C-5), 69.7 (C-4), 61.2 (C-6), 55.8 (CH_3). NMR data were consistent with literature data.[8]

β-Mannosylations and β-Rhamnosylations

General Procedure A for Mannosylation Donor Scope

A 25 mL crimp-top vial charged with a stir-bar, hemiacetal (0.10 mmol, 1 eq) and Ph_3PO (14 mg, 0.050 mmol, 0.5 eq) was placed under three cycles of vacuum and nitrogen. The solids were dissolved in anhydrous $CHCl_3$ (0.2 mL, 0.5 M), treated with oxalyl chloride (10 µL, 0.12 mmol, 1.2 eq) and left to stir at room temperature. After 1 h, the solvent and excess oxalyl chloride were removed by applying vacuum. Solid acceptor (0.07 mmol, 0.7 eq) and powdered LiI (53 mg, 0.40 mmol, 4 eq) were added to the vial and placed under three cycles of vacuum and nitrogen. The contents were re-dissolved in anhydrous $CHCl_3$ (0.25 mL, 0.4 M w.r.t. donor) and treated with iPr_2NEt (42 µL, 0.25 mmol, 2.5 eq). The reaction was stirred at 45 °C for 24 h or 30 °C for 36 h. The reaction mixture was diluted with CH_2Cl_2 (1 mL) and treated with 1 M HCl (1 mL). The aqueous layer was washed with CH_2Cl_2 (2 x 1 mL) and the combined organic layers were dried over anhydrous $MgSO_4$ and concentrated *in vacuo* to give a yellow or brown syrup. The α/β ratio was determined by ^1H NMR spectroscopy of the crude reaction mixture.

General Procedure B for Mannosylation Acceptor Scope

A 25 mL crimp-top vial charged with a stir-bar, hemiacetal (0.10 mmol, 1 eq) and Ph_3PO (28 mg, 0.10 mmol, 1 eq) was placed under three cycles of vacuum and nitrogen. The solids were dissolved in anhydrous $CHCl_3$ (0.2 mL, 0.5 M), treated with oxalyl chloride (10 µL, 0.12 mmol, 1.2 eq) and left to stir at room temperature. After 30 minutes, the solvent and excess oxalyl chloride were removed by applying vacuum. Solid acceptor (0.07 mmol, 0.7 eq) and powdered LiI (53 mg, 0.40 mmol, 4 eq) were added to the vial and placed under three cycles of vacuum and nitrogen. The contents were re-dissolved in anhydrous $CHCl_3$ (0.25 mL, 0.4 M w.r.t. donor) and treated with iPr_2NEt (69 µL, 0.40 mmol, 4 eq). The reaction was stirred at 45 °C for 24 h. The reaction mixture was diluted with CH_2Cl_2 (1 mL) and treated with 1 M HCl (1 mL). The aqueous layer was washed with CH_2Cl_2 (2 x 1 mL) and the combined organic layers were dried over anhydrous $MgSO_4$ and concentrated *in vacuo* to give a yellow or brown syrup. The α/β ratio was determined by ^1H NMR spectroscopy of the crude reaction mixture.

General Procedure C for Mannosylation Acceptor Scope

A 25 mL crimp-top vial charged with a stir-bar, hemiacetal (0.10 mmol, 1 eq) and Ph_3PO (28 mg, 0.10 mmol, 1 eq) was placed under three cycles of vacuum and nitrogen. The solids were dissolved in anhydrous $CHCl_3$ (0.2 mL, 0.5 M), treated with oxalyl chloride (10 µL, 0.12 mmol, 1.2 eq) and left to stir at room temperature. After 30 minutes, the solvent and excess oxalyl chloride were removed by applying vacuum. Powdered LiI (53 mg, 0.40 mmol, 4 eq) was added to the vial and placed under three cycles of vacuum and nitrogen. A stock solution of the acceptor in anhydrous $CHCl_3$ (0.4 M w.r.t. donor

or 0.28 M w.r.t acceptor) was added followed by iPr_2NEt (69 μL, 0.40 mmol, 4 eq). The reaction was stirred at 45 °C for 24 h. The reaction mixture was diluted with CH_2Cl_2 (1 mL) and treated with 1 M HCl (1 mL). The aqueous layer was washed with CH_2Cl_2 (2 x 1 mL) and the combined organic layers were dried over anhydrous $MgSO_4$ and concentrated *in vacuo* to give a yellow or brown syrup. The α/β ratio was determined by ^1H NMR spectroscopy of the crude reaction mixture.

General procedure D for Rhamnosylation Donor Scope

A 25 mL crimp-top vial charged with a stir-bar, hemiacetal (0.10 mmol, 1 eq) and Ph_3PO (28 mg, 0.10 mmol, 1 eq) was placed under three cycles of vacuum and nitrogen. The solids were dissolved in anhydrous $CHCl_3$ (0.2 mL, 0.5 M), treated with oxalyl chloride (10 μL, 0.12 mmol, 1.2 eq) and left to stir at room temperature. After 30 minutes, the solvent and excess oxalyl chloride were removed by applying vacuum. Solid acceptor (0.07 mmol, 0.7 eq) and powdered LiI (53 mg, 0.40 mmol, 4 eq) were added to the vial and placed under three cycles of vacuum and nitrogen. The contents were re-dissolved in anhydrous $CHCl_3$ (0.25 mL, 0.4 M w.r.t. donor) and treated with iPr_2NEt (69 μL, 0.40 mmol, 4 eq). The reaction was stirred at 45 °C or 30 °C for 24 h. The reaction mixture was diluted with CH_2Cl_2 (15 ml) and washed with 1M HCl (2 × 5 ml), brine (10 ml), dried using Na_2SO_4, filtered and concentrated *in vacuo*. The α/β ratio was determined by ^1H NMR spectroscopy of the crude reaction mixture.

General procedure E for Rhamnosylation Acceptor Scope

A 25 mL crimp-top vial charged with a stir-bar, hemiacetal (0.10 mmol, 1 eq) and Ph_3PO (14 mg, 0.050 mmol, 0.5 eq) was placed under three cycles of vacuum and nitrogen. The solids were dissolved in anhydrous $CHCl_3$ (0.2 mL, 0.5 M), treated with oxalyl chloride (10 μL, 0.12 mmol, 1.2 eq) and left to stir at room temperature. After 1 h, the solvent and excess oxalyl chloride were removed by applying vacuum. Solid acceptor (0.07 mmol, 0.7 eq) and powdered LiI (53 mg, 0.40 mmol, 4 eq) were added to the vial and placed under three cycles of vacuum and nitrogen. The contents were re-dissolved in anhydrous $CHCl_3$ (0.25 mL, 0.4 M w.r.t. donor) and treated with iPr_2NEt (42 μL, 0.25 mmol, 2.5 eq). The reaction was stirred at 45 °C or 30 °C for 24 h. The reaction mixture was diluted with CH_2Cl_2 (15 ml) and washed with 1M HCl (2 × 5 ml), brine (10 ml), dried using Na_2SO_4, filtered and concentrated *in vacuo*. The α/β ratio was determined by ^1H NMR spectroscopy of the crude reaction mixture.

Methyl (2,3,4,6-tetra-*O*-benzyl-β-D-mannopyranosyl)-(1→6)-2,3,4-tri-*O*-benzyl-α-D-glucopyranoside 2a

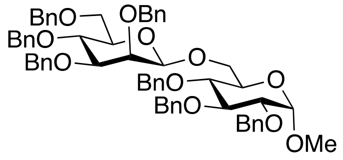

Following the general procedure A, hemiacetal **1a** (54 mg, 0.10 mmol), Ph_3PO (28 mg, 0.10 mmol, 1 eq) and acceptor **3a** (33 mg, 0.070 mmol) were used. The reaction was stirred at 45 °C for 18 h. ^{1}H NMR spectroscopy of the crude reaction mixture gave an α/β = 2:98. Purification by column chromatography (97:3; CH_2Cl_2/Et_2O) gave **2a** as a white solid (55 mg, 80% yield).

1 mmol scale

In a slight modification of general procedure A, hemiacetal **1a** (580 mg, 1.07 mmol), Ph_3PO (149 mg, 0.535 mmol, 0.05 eq) and acceptor **3a** (348 mg, 0.750 mmol) were used. The reaction was stirred at 30 °C for 18 h. ^{1}H NMR spectroscopy of the crude reaction mixture gave an α/β = 4:96. Purification by column chromatography (85:15; Cyclohexane/EtOAc) gave **2a** as a white solid (615 mg, 85% yield). Reaction was also performed at 45 °C leading to the desired product **2a** as a white solid **(**α/β = 10:90, 90% yield).

β-anomer

^{1}H NMR (500 MHz, Chloroform-*d*): δ 7.48 – 7.05 (m, 35H, ArCH), 5.01 (d, *J* = 10.9 Hz, 1H, C*H*HPh), 4.93 (d, *J* = 12.5 Hz, 1H, C*H*HPh), 4.88 (d, *J* = 10.8 Hz, 1H, C*H*HPh), 4.83 (d, *J* = 10.9 Hz, 1H, C*H*HPh), 4.81 (d, *J* = 11.5 Hz, 1H, C*H*HPh), 4.81 – 4.75 (m, 2H, 2 x C*H*HPh), 4.66 (d, *J* = 12.1 Hz, 1H, C*H*HPh), 4.61 – 4.54 (m, 3H, H-1, 2 x C*H*HPh), 4.53 (d, *J* = 11.9 Hz, 1H, C*H*HPh), 4.52 (d, *J* = 10.7 Hz, 1H, C*H*HPh), 4.51 (d, *J* = 11.5 Hz, 1H, C*H*HPh), 4.47 (d, *J* = 11.9 Hz, 1H, C*H*HPh), 4.16 (dd, *J* = 10.5, 2.0 Hz, 1H, H-6a), 4.12 (s, 1H, H-1'), 4.02 (t, *J* = 9.2 Hz, 1H, H-3), 3.83 (t, *J* = 9.5 Hz, 1H, H-4'), 3.81 – 3.77 (m, 1H, H-5), 3.77 (dd, *J* = 10.9, 2.2 Hz, 1H, H-6a'), 3.75 – 3.68 (m, 2H, H-2', H-6b'), 3.50 (dd, *J* = 9.7, 3.5 Hz, 1H, H-2), 3.49 – 3.41 (m, 2H, H-6b, H-4), 3.41 (dd, *J* = 9.4, 3.1 Hz, 1H, H-3'), 3.38 (dd, *J* = 9.7, 6.2, 2.1 Hz, 1H, H-5'), 3.32 (s, 3H, OCH_3). ^{13}C NMR (126 MHz, Chloroform-*d*): δ 139.0 (C), 138.9 (C), 138.6 (C), 138.4 (2 x C), 138.3 (C), 138.2 (C), 128.6 (CH), 128.52 (CH), 128.49 (CH), 128.48 (CH), 128.46 (CH), 128.40 (CH), 128.39 (CH), 128.3 (CH), 128.21 (CH), 128.18 (CH), 128.1 (CH), 127.9 (CH), 127.79 (CH), 127.75 (CH), 127.73 (CH), 127.7 (CH), 127.6 (CH), 127.5 (CH), 101.6 (C-1'), 97.9 (C-1), 82.4 (C-3'), 82.3 (C-3), 80.0 (C-2), 77.8 (C-4), 76.1 (C-5'), 75.8 (Ph*C*H_2), 75.3 (Ph*C*H_2), 75.1 (C-4'), 74.9 (Ph*C*H_2), 73.8 (Ph*C*H_2), 73.7 (C-2'), 73.6 (Ph*C*H_2), 73.5 (Ph*C*H_2), 71.7 (Ph*C*H_2), 69.91 (C-5), 69.88 (C-6'), 68.4 (C-6), 55.2 (OCH_3). NMR data were consistent with literature data.[33]

α-anomer

^{1}H NMR (500 MHz, Chloroform-*d*): δ 3.30 (s, 3H, OCH_3).

Optimisation reactions (isolated yields and α/β ratios)

Donor	Ph_3PO 1 eq 45 °C, 24 h	Ph_3PO 1 eq 30 °C, 36 h	Ph_3PO 2 eq 45 °C, 18 h	Ph_3PO 0.5 eq 45 °C, 18 h
**1b**	α/β = 21:79<br>71%	α/β = 5:95<br>78%		α/β = 5:95<br>93%
**1c**	α/β = 19:81<br>70%	α/β = 4:96<br>93%		α/β = 4:97<br>90%
**1d**	α/β = 14:86<br>50% conv	α/β = 5:95<br>46%		α/β = 3:97<br>71%
**1e**	α/β = 11:89<br>77%	α/β = 12:88<br>77%	α/β = 59:41<br>67% conv	α/β = 5:95<br>94%

Methyl (2,3,4-tri-*O*-benzyl-6-*O*-(4-methylbenzyl)-β-D-mannopyranosyl)-(1→6)-2,3,4-tri-*O*-benzyl-α-D-glucopyranoside 2b

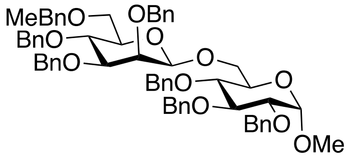

Following the general procedure A, hemiacetal **1b** (55 mg, 0.10 mmol) and acceptor **3a** (33 mg, 0.070 mmol) were used. The reaction was stirred at 45 °C for 18 h. ^{1}H NMR spectroscopy of the crude reaction mixture gave an α/β = 5:95. Purification by column chromatography (97:3; CH_2Cl_2/Et_2O) gave **2b** as a white solid (64 mg, 93% yield). ESI-HRMS for $C_{63}H_{68}O_{11}Na^+$ $(M+Na)^+$ calculated: 1023.4654; found: 1023.4659.

β-anomer

^{1}H NMR (600 MHz, Chloroform-*d*): δ 7.53 – 7.11 (m, 32H, ArCH), 7.07 (d, *J* = 7.7 Hz, 2H, ArCH), 5.01 (d, *J* = 10.9 Hz, 1H, C*H*HPh), 4.93 (d, *J* = 12.5 Hz, 1H, C*H*HPh), 4.87 (d, *J* = 10.7 Hz, 1H, C*H*HPh), 4.82 (d, *J* = 11.0 Hz, 1H, C*H*HPh), 4.81 (d, *J* = 11.4 Hz, 1H, C*H*HPh), 4.78 (d, *J* = 12.5 Hz, 1H, C*H*HPh), 4.78 (d, *J* = 12.2 Hz, 1H, C*H*HPh), 4.66 (d, *J* = 12.1 Hz, 1H, C*H*HPh), 4.58 (d, *J* = 3.5 Hz, 1H, H-1), 4.55 (d, *J* = 11.9 Hz, 1H, C*H*HPh), 4.53 – 4.49 (m, 4H, 4 x C*H*HPh), 4.47 (d, *J* = 11.7 Hz, 1H, C*H*HPh),

4.16 (dd, J = 10.5, 2.0 Hz, 1H, H-6a), 4.11 (s, 1H, H-1'), 4.02 (t, J = 9.2 Hz, 1H, H-3), 3.82 (t, J = 9.5 Hz, 1H, H-4'), 3.80 – 3.77 (m, 1H, H-5), 3.75 (dd, J = 10.9, 1.9 Hz, 1H, H-6a'), 3.72 (d, J = 3.0 Hz, 1H, H-2'), 3.70 (dd, J = 10.8, 5.9 Hz, 1H, H-6b'), 3.50 (dd, J = 9.6, 3.5 Hz, 1H, H-2), 3.47 – 3.42 (m, 2H, H-6b, H-4), 3.40 (dd, J = 9.4, 3.0 Hz, 1H, H-3'), 3.37 (ddd, J = 9.6, 6.1, 2.0 Hz, 1H, H-5'), 3.33 (s, 3H, OCH_3), 2.30 (s, 3H, CH_3). ^{13}C NMR (151 MHz, Chloroform-*d*): δ 139.0 (C), 138.9 (C), 138.43 (C), 138.42 (C), 138.3 (C), 138.2 (C), 137.2 (C), 135.5 (C), 129.1 (CH), 128.6 (CH), 128.51 (CH), 128.47 (CH), 128.42 (CH), 128.40 (CH), 128.28 (CH), 128.27 (CH), 128.20 (CH), 128.18 (CH), 128.10 (CH), 128.05 (CH), 127.77 (CH), 127.76 (CH), 127.73 (CH), 127.71 (CH), 127.66 (CH), 127.5 (CH), 101.6 (C-1', $^1J_{CH}$ = 154.0 Hz, from coupled HSQC), 97.9 (C-1, $^1J_{CH}$ = 170.0 Hz, from coupled HSQC), 82.4 (C-3'), 82.3 (C-3), 80.0 (C-2), 77.8 (C-4), 76.1 (C-5'), 75.8 (Ph*C*H_2), 75.3 (Ph*C*H_2), 75.1 (C-4'), 74.9 (Ph*C*H_2), 73.8 (Ph*C*H_2), 73.7 (C-2'), 73.46 (Ph*C*H_2), 73.45 (Ph*C*H_2), 71.7 (Ph*C*H_2), 69.9 (C-5), 69.6 (C-6'), 68.4 (C-6), 55.2 (OCH_3), 21.3 (CH_3).

α-anomer

^{1}H NMR (600 MHz, Chloroform-*d*): δ 4.96 (d, J = 1.8 Hz, 1H, H-1'), 3.30 (s, 3H, OCH_3). ^{13}C NMR (151 MHz, Chloroform-*d*): δ 98.4 (C-1', $^1J_{CH}$ = 170.0 Hz, from coupled HSQC).

Methyl (2,3,6-tri-*O*-benzyl-4-*O*-(4-methylbenzyl)-β-D-mannopyranosyl)-(1→6)-2,3,4-tri-*O*-benzyl-α-D-glucopyranoside 2c

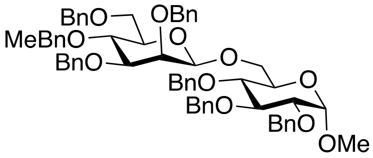

Following the general procedure A, hemiacetal **1c** (55 mg, 0.10 mmol) and acceptor **3a** (32 mg, 0.069 mmol) were used. The reaction was stirred at 45 °C for 18 h. ^{1}H NMR spectroscopy of the crude reaction mixture gave an α/β = 4:96. Purification by column chromatography (97:3; CH_2Cl_2/Et_2O) gave **2c** as a white solid (62 mg, 90% yield). ESI-HRMS for $C_{63}H_{68}O_{11}Na^+$ $(M+Na)^+$ calculated: 1023.4654; found: 1023.4659.

β-anomer

^{1}H NMR (500 MHz, Chloroform-*d*): δ 7.43 – 7.15 (m, 32H, ArCH), 7.07 (s, 2H, ArCH), 5.01 (d, J = 11.0 Hz, 1H, C*H*HPh), 4.93 (d, J = 12.5 Hz, 1H, C*H*HPh), 4.83 (d, J = 10.5 Hz, 1H, C*H*HPh), 4.82 (d, J = 10.9 Hz, 1H, C*H*HPh), 4.81 (d, J = 11.5 Hz, 1H, C*H*HPh), 4.78 (d, J = 12.5 Hz, 1H, C*H*HPh), 4.78 (d, J = 12.1 Hz, 1H, C*H*HPh), 4.66 (d, J = 12.1 Hz, 1H, C*H*HPh), 4.62 – 4.54 (m, 3H, 2 x C*H*HPh, H-1), 4.53 (d, J = 11.9 Hz, 1H, C*H*HPh), 4.51 (d, J = 11.5 Hz, 1H, C*H*HPh), 4.49 (d, J = 11.9 Hz, 1H, C*H*HPh), 4.48 (d, J = 10.5 Hz, 1H, C*H*HPh), 4.15 (dd, J = 10.5, 2.0 Hz, 1H, H-6a), 4.11 (d, J = 0.7 Hz,

1H, H-1'), 4.01 (dd, J = 9.7, 8.8 Hz, 1H, H-3), 3.81 (t, J = 9.5 Hz, 1H, H-4'), 3.81 – 3.74 (m, 2H, H-5, H-6a'), 3.73 – 3.67 (m, 2H, H-2', H-6b'), 3.50 (dd, J = 9.7, 3.5 Hz, 1H, H-2), 3.49 – 3.40 (m, 2H, H-6b, H-4), 3.40 (dd, J = 9.4, 3.0 Hz, 1H, H-3'), 3.37 (ddd, J = 9.7, 6.1, 1.9 Hz, 1H, H-5'), 3.32 (s, 3H, OCH_3), 2.32 (s, 3H, CH_3). ^{13}C NMR (126 MHz, Chloroform-*d*): δ 139.0 (C), 138.9 (C), 138.6 (C), 138.42 (C), 138.35 (C), 138.2 (C), 137.5 (C), 129.1 (CH), 128.6 (CH), 128.52 (CH), 128.48 (CH), 128.39 (CH), 128.38 (CH), 128.29 (CH), 128.27 (CH), 128.2 (CH), 128.1 (CH), 127.9 (CH), 127.8 (CH), 127.72 (CH), 127.67 (CH), 127.6 (CH), 127.5 (CH), 101.6 (C-1', $^1J_{1CH}$ = 154.8 Hz, from coupled HSQC), 97.9 (C-1, $^1J_{1CH}$ = 169.8 Hz, from coupled HSQC), 82.4 (C-3'), 82.3 (C-3), 80.0 (C-2), 77.8 (C-4), 76.1 (C-5'), 75.8 (PhCH$_2$), 75.2 (PhCH$_2$), 74.94 (C-4'), 74.85 (PhCH$_2$), 73.8 (PhCH$_2$ and C-2'), 73.6 (PhCH$_2$), 73.5 (PhCH$_2$), 71.7 (PhCH$_2$), 69.91 (C-5), 69.87 (C-6'), 68.4 (C-6), 55.2 (OCH_3), 21.3 (CH_3).

α-anomer

^{1}H NMR (500 MHz, Chloroform-*d*): δ 4.95 (d, J = 1.9 Hz, 1H, H-1'), 3.30 (s, 3H, OCH_3). ^{13}C NMR (126 MHz, Chloroform-*d*): δ 98.4 (C-1, $^1J_{1CH}$ = 171.2 Hz, from coupled HSQC).

Methyl (2,3,4-tri-*O*-benzyl-6-*O*-(4-methoxylbenzyl)-β-D-mannopyranosyl)-(1→6)-2,3,4-tri-*O*-benzyl-α-D-glucopyranoside 2d

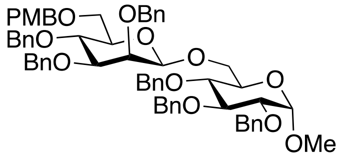

Following the general procedure A, hemiacetal **1d** (57 mg, 0.10 mmol) and acceptor **3a** (32 mg, 0.069 mmol) were used. The reaction was stirred at 45 °C for 18 h. ^{1}H NMR spectroscopy of the crude reaction mixture gave an α/β = 3:97. Purification by column chromatography (97:3; CH_2Cl_2/Et_2O) gave **2d** as a white solid (49 mg, 71% yield). ESI-HRMS for $C_{63}H_{68}O_{12}Na^+$ $(M+Na)^+$ calculated: 1039.4603; found: 1039.4613.

β-anomer

^{1}H NMR (600 MHz, Chloroform-*d*): δ 7.45 – 7.14 (m, 32H, ArCH), 6.82 – 6.74 (m, 2H, ArCH), 5.01 (d, J = 10.9 Hz, 1H, C*H*HPh), 4.93 (d, J = 12.5 Hz, 1H, C*H*HPh), 4.87 (d, J = 10.7 Hz, 1H, C*H*HPh), 4.82 (d, J = 10.9 Hz, 1H, C*H*HPh), 4.81 (d, J = 11.4 Hz, 1H, C*H*HPh), 4.78 (d, J = 12.5 Hz, 1H, C*H*HPh), 4.78 (d, J = 12.1 Hz, 1H, C*H*HPh),4.66 (d, J = 12.1 Hz, 1H, C*H*HPh), 4.58 (d, J = 3.5 Hz, 1H, H-1), 4.55 – 4.45 (m, 6H, 6 x C*H*HPh), 4.17 (dd, J = 10.5, 2.0 Hz, 1H, H-6a), 4.12 (s, 1H, H-1'), 4.02 (t, J = 9.2 Hz, 1H, H-3), 3.82 (t, J = 9.5 Hz, 1H, H-4'), 3.80 (ddd, J = 10.1, 5.5, 1.9 Hz, 1H, H-5), 3.75 (s, 3H, OCH_3), 3.75 – 3.73 (m, 1H, H-6a'), 3.73 – 3.71 (m, 1H, H-2'), 3.69 (dd, J = 10.9, 5.9 Hz, 1H, H-6b'), 3.50 (dd, J = 9.7, 3.5 Hz, 1H, H-2), 3.46 (dd, J = 10.6, 5.4 Hz, 1H, H-6b), 3.44 (dd, J = 10.0, 8.8 Hz,

1H, H-4), 3.41 (dd, J = 9.4, 3.0 Hz, 1H, H-3'), 3.36 (ddd, J = 9.8, 5.9, 2.0 Hz, 1H, H-5'), 3.33 (s, 3H, OCH_3). ^{13}C NMR (151 MHz, Chloroform-d): δ 159.2 (C), 139.0 (C), 138.9 (C), 138.43 (C), 138.42 (C), 138.3 (C), 138.2 (C), 130.6 (C), 129.6 (CH), 128.6 (CH), 128.51 (CH), 128.48 (CH), 128.43 (CH), 128.39 (CH), 128.3 (CH), 128.19 (CH), 128.18 (CH), 128.1 (CH), 127.78 (CH), 127.77 (CH), 127.73 (CH), 127.72 (CH), 127.67 (CH), 127.5 (CH), 113.8 (CH), 101.6 (C-1', $^1J_{1CH}$ = 155.0 Hz, from coupled HSQC), 97.90 (C-1, $^1J_{1CH}$ = 170.1 Hz, from coupled HSQC), 82.4 (C-3'), 82.3 (C-3), 80.0 (C-2), 77.8 (C-4), 76.1 (C-5'), 75.8 (PhCH$_2$), 75.3 (PhCH$_2$), 75.1 (C-4'), 74.9 (PhCH$_2$), 73.8 (PhCH$_2$), 73.8 (C-2'), 73.5 (PhCH$_2$), 73.22 (PhCH$_2$), 71.7 (PhCH$_2$), 69.9 (C-5), 69.4 (C-6'), 68.4 (C-6), 55.3 (OCH_3), 55.2 (OCH_3).

α-anomer

^1H NMR (500 MHz, Chloroform-d): δ 4.96 (d, J = 1.7 Hz, 1H, H-1'), 3.30 (s, 3H, OCH_3). ^{13}C NMR (126 MHz, Chloroform-d) from HSQC: δ 98.3 (C-1').

Methyl (2,3,4-tri-*O*-benzyl-6-*O*-(2-naphthyl)-β-D-mannopyranosyl)-(1→6)-2,3,4-tri-*O*-benzyl-α-D-glucopyranoside 2e

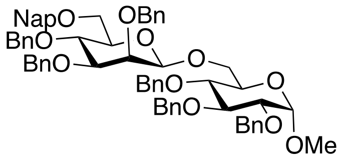

Following the general procedure A, hemiacetal **1e** (59 mg, 0.10 mmol) and acceptor **3a** (32 mg, 0.069 mmol) were used. The reaction was stirred at 45 °C for 18 h. ^1H NMR spectroscopy of the crude reaction mixture gave an α/β = 5:95. Purification by column chromatography (97:3; CH_2Cl_2/Et_2O) gave **2e** as a white solid (68 mg, 94% yield). ESI-HRMS for $C_{66}H_{68}O_{11}Na^+$ $(M+Na)^+$ calculated: 1059.4654; found: 1059.4660.

β-anomer

^1H NMR (500 MHz, Chloroform-d): δ 7.86 – 7.70 (m, 4H, ArCH), 7.52 – 7.07 (m, 33H, ArCH), 5.01 (d, J = 10.9 Hz, 1H, C*H*HPh), 4.94 (d, J = 12.5 Hz, 1H, C*H*HPh), 4.87 (d, J = 10.8 Hz, 1H, C*H*HPh), 4.82 (d, J = 11.0 Hz, 1H, C*H*HPh), 4.80 (d, J = 11.5 Hz, 1H, C*H*HPh), 4.79 (d, J = 12.5 Hz, 1H, C*H*HPh), 4.78 (d, J = 12.0 Hz, 1H, C*H*HPh), 4.75 (d, J = 12.3 Hz, 1H, C*H*HPh), 4.72 (d, J = 12.3 Hz, 1H, C*H*HPh), 4.66 (d, J = 12.1 Hz, 1H, C*H*HPh), 4.58 (d, J = 3.5 Hz, 1H, H-1), 4.56 – 4.56 (m, 4H, 4 x C*H*HPh), 4.18 (dd, J = 10.5, 2.0 Hz, 1H, H-6a), 4.13 (s, 1H, H-1'), 4.02 (t, J = 9.2 Hz, 1H, H-3), 3.88 – 3.81 (m, 1H, H-4'), 3.83 – 3.78 (m, 2H, H-5, H-6a'), 3.75 (dd, J = 10.9, 6.0 Hz, 1H, H-6b'), 3.73 (d, J = 2.9 Hz, 1H, H-2'), 3.51 (dd, J = 9.6, 3.5 Hz, 1H, H-2), 3.50 – 3.37 (m, 4H, H-6b, H-4, H-3', H-5'), 3.32 (s, 3H, OCH_3). ^{13}C NMR (126 MHz, Chloroform-d): δ 139.0 (C), 138.9 (C), 138.4 (C), 138.31 (C), 138.29 (C),

138.2 (C), 136.1 (C), 133.4 (C), 133.1 (C), 128.6 (CH), 128.51 (CH), 128.49 (CH), 128.47 (CH), 128.40 (CH), 128.3 (CH), 128.17 (CH), 128.15 (CH), 128.05 (CH), 128.02 (CH), 127.8 (CH), 127.74 (CH), 127.67 (CH), 127.5 (CH), 126.6 (CH), 126.1 (CH), 125.9 (CH), 101.7 (C-1', $^1J_{1CH}$ = 154.1 Hz, from coupled HSQC), 97.9 (C-1, $^1J_{1CH}$ = 169.1 Hz, from coupled HSQC), 82.4 (C-3'), 82.3 (C-3), 80.0 (C-2), 77.8 (C-4), 76.1 (C-5'), 75.8 (Ph*C*H$_2$), 75.3 (Ph*C*H$_2$), 75.1 (C-4'), 74.8 (Ph*C*H$_2$), 73.82 (Ph*C*H$_2$), 73.72 (C-2'), 73.69 (Ph*C*H$_2$), 73.5 (Ph*C*H$_2$), 71.7 (Ph*C*H$_2$), 69.9 (C-5), 69.8 (C-6'), 68.4 (C-6), 55.2 (OCH_3).

α-anomer

^{1}H NMR (500 MHz, Chloroform-*d*): δ 4.98 (d, *J* = 1.9 Hz, 1H, H-1'), 3.30 (s, 3H, OCH_3). ^{13}C NMR (126 MHz, Chloroform-*d*) from short HSQC: δ 98.5 (C-1').

Methyl (2,3,4-tri-*O*-benzyl-6-*O*-*tert*-butyldiphenylsilyl-β-D-mannopyranosyl)-(1→6)-2,3,4-tri-*O*-benzyl-α-D-glucopyranoside 2f

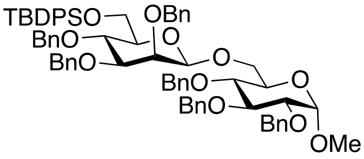

Following the general procedure A, hemiacetal **1f** (69 mg, 0.10 mmol) and acceptor **3a** (32 mg, 0.069 mmol) were used. The reaction was stirred at 45 °C for 18 h. ^{1}H NMR spectroscopy of the crude reaction mixture gave an α/β = 9:91. Purification by column chromatography (100:0 to 97:3; CH_2Cl_2/Et_2O) gave **2f** as a white solid (74 mg, 95% yield). ESI-HRMS for $C_{71}H_{82}O_{11}SiN^+$ $(M+NH_4)^+$ calculated: 1152.5652; found: 1152.5654.

β-anomer

^{1}H NMR (500 MHz, Chloroform-*d*): δ 7.83 – 7.76 (m, 2H, ArCH), 7.77 – 7.66 (m, 2H, ArCH), 7.49 – 7.12 (m, 36H, ArCH), 5.03 (d, *J* = 10.9 Hz, 1H, C*H*HPh), 4.96 (d, *J* = 12.2 Hz, 1H, C*H*HPh), 4.93 (d, *J* = 10.8 Hz, 1H, C*H*HPh), 4.89 – 4.75 (m, 4H, 4 x C*H*HPh), 4.68 (d, *J* = 12.1 Hz, 1H, C*H*HPh), 4.64 – 4.53 (m, 5H, H-1, 4 x C*H*HPh), 4.19 (dd, *J* = 10.6, 2.0 Hz, 1H, H-6a), 4.15 (s, 1H, H-1'), 4.05 (t, *J* = 9.2 Hz, 1H, H-3), 4.02 (t, *J* = 9.5 Hz, 1H, H-4'), 3.98 (dd, *J* = 11.1, 4.8 Hz, 1H, H-6a'), 3.95 (dd, *J* = 11.1, 2.3 Hz, 1H, H-6b'), 3.82 (ddd, *J* = 10.0, 5.2, 2.0 Hz, 1H, H-5), 3.76 (d, *J* = 3.1 Hz, 1H, H-2'), 3.54 – 3.45 (m, 3H, H-2, H-4, H-6b), 3.46 (dd, *J* = 9.4, 2.9 Hz, 1H, H-3'), 3.37 (s, 3H, OCH_3), 3.29 (ddd, *J* = 9.6, 4.9, 2.2 Hz, 1H, H-5'), 1.03 (s, 9H, SiC(C*H*$_3$)$_3$). ^{13}C NMR (126 MHz, Chloroform-*d*): δ 139.1 (C), 138.9 (C), 138.6 (C), 138.5 (C), 138.4 (C), 138.2 (C), 136.0 (CH), 135.7 (CH), 134.1 (C), 133.6 (C), 129.60 (CH), 129.55 (CH), 128.6 (CH), 128.51 (CH), 128.50 (CH), 128.45 (CH), 128.3 (CH), 128.19 (CH), 128.16 (CH), 128.10 (CH), 128.05 (CH), 127.76 (CH), 127.75 (CH), 127.73 (CH), 127.67 (CH),

127.3 (CH), 101.8 (C-1', ${}^{1}J_{1CH}$ = 154.3 Hz, from coupled HSQC), 98.0 (C-1, ${}^{1}J_{1CH}$ = 169.7 Hz, from coupled HSQC), 82.3 (C-3'), 82.3 (C-3), 80.1 (C-2), 77.7 (C-4), 77.0 (C-5'), 75.9 (Ph*C*H_2), 75.4 (Ph*C*H_2), 74.9 (C-4'), 74.8 (Ph*C*H_2), 74.2 (C-2'), 73.8 (Ph*C*H_2), 73.5 (Ph*C*H_2), 71.7 (Ph*C*H_2), 69.9 (C-5), 68.1 (C-6), 63.4 (C-6'), 55.2 (OCH_3), 26.8 (SiC(*C*H_3)$_3$), 19.4 (Si*C*(CH_3)$_3$).

α-anomer

^{1}H NMR (500 MHz, Chloroform-*d*): δ 3.33 (s, 3H, OCH_3), 1.05 (s, 9H, SiC(C*H*$_3$)$_3$). ^{13}C NMR (126 MHz, Chloroform-*d*): δ 98.2 (C-1'), 97.9 (C-1), 26.9 (SiC(*C*H_3)$_3$).

Methyl (2,3,6-tri-*O*-benzyl-4-*O*-*tert*-butyldimethylsilyl-β-D-mannopyranosyl)-(1→6)-2,3,4-tri-*O*-benzyl-α-D-glucopyranoside 2g

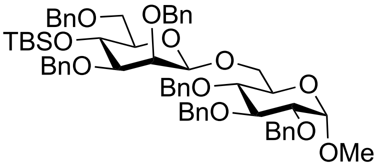

Following the general procedure A, hemiacetal **1g** (56 mg, 0.10 mmol), Ph_3PO (28 mg, 0.10 mmol, 1 eq) and acceptor **3a** (32 mg, 0.069 mmol) were used. The reaction was stirred at 45 °C for 24 h. ^{1}H NMR spectroscopy of the crude reaction mixture gave an α/β = 5:95. Purification by column chromatography (97:3; CH_2Cl_2/Et_2O) gave **2g** as a white solid (31 mg, 44% yield). ESI-HRMS for $C_{61}H_{74}O_{11}SiNa^+$ $(M+Na)^+$ calculated: 1033.4893; found: 1033.4897.

β-anomer

^{1}H NMR (500 MHz, Chloroform-*d*): δ 7.40 – 7.11 (m, 30H, ArCH), 5.00 (d, *J* = 10.9 Hz, 1H, C*H*HPh), 4.89 (d, *J* = 12.4 Hz, 1H, C*H*HPh), 4.82 (d, *J* = 11.0 Hz, 1H, C*H*HPh), 4.81 (d, *J* = 11.2 Hz, 1H, C*H*HPh), 4.78 (d, *J* = 12.1 Hz, 1H, C*H*HPh), 4.65 (d, *J* = 12.1 Hz, 1H, C*H*HPh), 4.64 (d, *J* = 12.0 Hz, 1H, C*H*HPh), 4.64 (d, *J* = 12.3 Hz, 1H, C*H*HPh), 4.57 (d, *J* = 3.5 Hz, 1H, H-1), 4.51 (d, *J* = 11.4 Hz, 1H, C*H*HPh), 4.50 (d, *J* = 12.1 Hz, 1H, C*H*HPh), 4.49 (d, *J* = 11.9 Hz, 1H, C*H*HPh), 4.39 (d, *J* = 11.9 Hz, 1H, C*H*HPh), 4.23 (d, *J* = 0.9 Hz, 1H, H-1'), 4.18 (dd, *J* = 10.5, 2.0 Hz, 1H, H-6a), 4.01 (t, *J* = 9.3 Hz, 1H, H-3), 3.88 (t, *J* = 9.1 Hz, 1H, H-4'), 3.82 (dd, *J* = 10.7, 1.9 Hz, 1H, H-6a'), 3.81 – 3.76 (m, 1H, H-5), 3.71 (d, *J* = 2.8 Hz, 1H, H-2'), 3.62 (dd, *J* = 10.7, 7.4 Hz, 1H, H-6b'), 3.53 – 3.46 (m, 2H, H-2, H-6b), 3.44 (dd, *J* = 10.0, 8.9 Hz, 1H, H-4), 3.35 (ddd, *J* = 9.3, 7.4, 1.9 Hz, 1H, H-5'), 3.31 (s, 3H, OCH_3), 3.20 (dd, *J* = 9.0, 2.8 Hz, 1H, H-3'), 0.79 (s, 9H, SiC(C*H*$_3$)$_3$), -0.028 (s, 3H, $SiCH_3$), -0.035 (s, 3H, $SiCH_3$). ^{13}C NMR (126 MHz, Chloroform-*d*): δ 139.1 (C), 139.0 (C), 138.7 (C), 138.4 (C), 138.23 (C), 138.21 (C), 128.6 (CH), 128.53 (CH), 128.49 (CH), 128.4 (CH), 128.31 (CH), 128.30 (CH), 128.19 (CH), 128.16 (CH), 128.08 (CH), 128.06 (CH), 128.0 (CH), 127.79 (CH), 127.76 (CH), 127.7 (CH), 127.64 (CH), 127.55 (CH), 127.5 (CH), 127.4 (CH), 101.4 (C-1', ${}^{1}J_{1CH}$ = 155.4 Hz, from coupled HSQC), 97.9 (C-1, ${}^{1}J_{1CH}$ = 170.2

Hz, from coupled HSQC), 82.4 (C-3'), 82.3 (C-3), 80.0 (C-2), 77.9 (C-4), 77.7 (C-5'), 75.9 (Ph*C*H_2), 74.9 (Ph*C*H_2), 74.02 (C-2'), 73.98 (Ph*C*H_2), 73.6 (Ph*C*H_2), 73.5 (Ph*C*H_2), 71.2 (Ph*C*H_2), 70.4 (C-6'), 70.0 (C-5), 68.4 (C-4'), 68.3 (C-6), 55.1 (OCH_3), 26.1 (SiC(CH_3)$_3$), 18.2 (Si*C*(CH_3)$_3$), -3.6 ($SiCH_3$), -4.8 ($SiCH_3$).

α-anomer

^{1}H NMR (500 MHz, Chloroform-*d*): δ 3.30 (s, 3H, OCH_3).

Methyl (2,3,4-tri-*O*-benzyl-6-*O*-(benzoyl)-α/β-D-mannopyranosyl)-(1→6)-2,3,4-tri-*O*-benzyl-α-D-glucopyranoside 2h

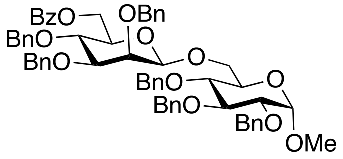

Following the general procedure A, hemiacetal **1h** (55 mg, 0.10 mmol), Ph_3PO (28 mg, 0.10 mmol, 1 eq) and acceptor **3a** (32 mg, 0.069 mmol) were used. The reaction was stirred at 45 °C for 44 h. ^{1}H NMR spectroscopy of the crude reaction mixture gave an α/β = 15:85. Purification by column chromatography (97:3; CH_2Cl_2/Et_2O) gave **2h** as a white solid (23 mg, 37% yield).

β-anomer

^{1}H NMR (500 MHz, Chloroform-*d*): δ 8.05 – 7.98 (m, 2H, ArCH), 7.52 – 7.46 (m, 1H, ArCH), 7.45 – 7.41 (m, 2H, ArCH), 7.39 – 7.11 (m, 30H, ArCH), 4.99 (d, *J* = 10.9 Hz, 1H, C*H*HPh), 4.94 (d, *J* = 12.3 Hz, 1H, C*H*HPh), 4.93 (d, *J* = 10.7 Hz, 1H, C*H*HPh), 4.80 (d, *J* = 10.9 Hz, 1H, C*H*HPh), 4.80 (d, *J* = 11.5 Hz, 1H, C*H*HPh), 4.78 (d, *J* = 12.3 Hz, 1H, C*H*HPh), 4.76 (d, *J* = 12.2 Hz, 1H, C*H*HPh), 4.64 (d, *J* = 12.1 Hz, 1H, C*H*HPh), 4.61 (dd, *J* = 11.7, 2.3 Hz, 1H, H-6a'), 4.59 (d, *J* = 10.7 Hz, 1H, C*H*HPh), 4.59 (d, *J* = 11.9 Hz, 1H, C*H*HPh), 4.55 (d, *J* = 3.6 Hz, 1H, H-1), 4.52 (d, *J* = 11.8 Hz, 1H, C*H*HPh), 4.49 (d, *J* = 11.5 Hz, 1H, C*H*HPh), 4.48 (dd, *J* = 11.7, 5.5 Hz, 1H, H-6b'), 4.19 (s, 1H, H-1'), 4.12 (dd, *J* = 10.5, 2.0 Hz, 1H, H-6a), 3.99 (t, *J* = 9.4 Hz, 1H, H-4'), 3.98 (t, *J* = 9.2 Hz, 1H, H-3), 3.81 – 3.76 (m, H-1, H-5), 3.78 (d, *J* = 3.0 Hz, 1H, H-2'), 3.52 (ddd, *J* = 9.6, 5.5, 2.3 Hz, 1H, H-5'), 3.50 – 3.43 (m, 3H, H-3', H-2, H-6b), 3.39 (dd, *J* = 10.1, 8.8 Hz, 1H, H-4), 3.28 (s, 3H, OCH_3). ^{13}C NMR (126 MHz, Chloroform-*d*): δ 166.5 (C=O), 138.92 (C), 138.85 (C), 138.3 (C), 138.2 (C), 138.13 (C), 138.10 (C), 133.0 (CH), 130.2 (C), 129.9 (CH), 128.60 (CH), 128.57 (CH), 128.56 (CH), 128.54 (CH), 128.51 (CH), 128.4 (CH), 128.34 (CH), 128.31 (CH), 128.29 (CH), 128.14 (CH), 128.10 (CH), 128.08 (CH), 127.88 (CH), 127.86 (CH), 127.79 (CH), 127.76 (CH), 127.5 (CH), 101.9 (C-1'), 97.9 (C-1), 82.2 (C-3 and C-3'), 80.0 (C-2), 77.9 (C-4) , 75.9 (Ph*C*H_2), 75.4 (Ph*C*H_2), 74.9 (Ph*C*H_2), 74.8 (C-4'), 73.9 (Ph*C*H_2, C-2'

and C-5'), 73.5 (PhCH_2), 71.7 (PhCH_2), 69.9 (C-5), 68.6 (C-6), 64.2 (C-6'), 55.1 (OCH_3). NMR data were consistent with literature data.

α-anomer

^{1}H NMR (500 MHz, Chloroform-*d*): δ 3.31 (s, 3H, OCH_3).

Methyl (2,3,4-tri-*O*-*p*-methylbenzyl-6-*O*-(pivaloyl)-β-D-mannopyranosyl)-(1→6)-2,3,4-tri-*O*-benzyl-α-D-glucopyranoside 2i

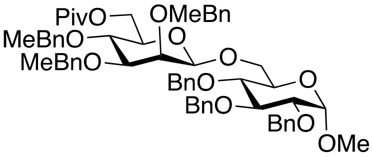

Following general procedure A, hemiacetal **1i** (58 mg, 0.10 mmol), iPr_2NEt (70 µL, 0.4 mmol) and acceptor **3a** (33 mg, 0.07 mmol) were used. The reaction was stirred at 45 °C for 24 h. ^{1}H NMR spectroscopy of the crude reaction mixture showed β-only product. Purification by column chromatography (6:4; pentane/ Et_2O) afforded the desired product **2i** as a colourless syrup (72 mg, quantitative yield). R_f = 0.5 (4:1; cyclohexane/ EtOAc); ^{1}H NMR (600 MHz, Chloroform-*d*) δ 7.39 – 7.08 (m, 27H, Ar-CH), 5.01 (d, *J* = 10.8 Hz, 1H, $PhCH_2$), 4.92 – 4.70 (m, 6H, 4 x $PhCH_2$), 4.67 (d, *J* = 12.1 Hz, 1H, $PhCH_2$), 4.60 – 4.57 (m, 1H, H-1), 4.53 – 4.43 (m, 4H, 4 x $PhCH_2$), 4.41 (d, *J* = 11.8 Hz, 1H, H-6a'), 4.17 (dd, *J* = 11.6, 6.9 Hz, 1H, H-6b'), 4.14 – 4.11 (m, 2H, H-6a, H-1'), 4.00 (t, *J* = 9.3 Hz, 1H, H-3), 3.82 – 3.73 (m, 2H, H-4', H-5'), 3.70 (br s, 1H, H-2'), 3.51 (ddd, *J* = 9.7, 3.5, 1.6 Hz, 1H, H-2), 3.48 – 3.34 (m, 4H, H-6b, H-4, H-3', H-5), 3.30 (s, 3H, OCH_3), 2.35 – 2.31 (m, 9H, CH_3), 1.15 (d, *J* = 1.7 Hz, 9H, CH_3). ^{13}C NMR (126 MHz, Chloroform-*d*) δ 178.2 (C=O), 138.8 (4ºC), 138.3 (4ºC), 138.1 (4ºC), 137.6 (4ºC), 137.4 (4ºC), 137.0 (4ºC), 135.6 (4ºC), 135.1 (4ºC), 135.0 (4ºC), 129.13 (Ar-*C*H), 129.09 (Ar-*C*H), 128.8 (Ar-*C*H), 128.50 (Ar-*C*H), 128.48 (Ar-*C*H), 128.42 (Ar-*C*H), 128.39 (Ar-*C*H), 128.3 (Ar-*C*H), 128.2 (Ar-*C*H), 128.1 (Ar-*C*H), 128.0 (Ar-*C*H), 127.8 (Ar-*C*H), 127.7 (Ar-*C*H), 127.7 (Ar-*C*H), 101.5 (C-1', $^1J_{CH}$ = 156.0 Hz, from coupled HSQC), 97.8 (C-1, $^1J_{CH}$ = 170.5 Hz, from coupled HSQC), 82.2 (C-3), 82.1 (C-3'), 79.9 (C-2), 77.7 (C-4), 75.8 (PhCH_2), 75.2 (PhCH_2), 74.8 (C-4', PhCH_2), 73.9 (C-5), 73.40 (PhCH_2), 73.36 (PhCH_2), 73.1 (C-2'), 71.5 (PhCH_2), 69.7 (C-5'), 68.1 (C-6), 63.7 (C-6'), 55.0 (OCH_3), 38.8 ($C(CH_3)_3$), 27.2 (2 x CH_3), 21.2 (CH_3). ESI-HRMS for $C_{63}H_{74}O_{12}Na^+$ $(M+Na)^+$ calculated: 1045.5072; found: 1045.5074.

Methyl 2,3,4-tri-*O*-benzyl-6-*O*-(2,3,4-tri-*O*-benzyl-β-L-rhamnosyl)-α-D-glucopyranoside 2j

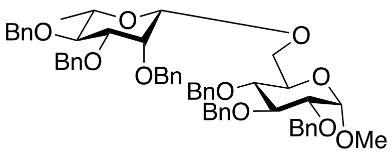

Following general procedure D, hemiacetal **1j** (50 mg, 0.12 mmol), Ph_3PO (33.4 mg, 0.120 mmol), $(COCl)_2$ (12 µL, 0.14 mmol), LiI (64 mg, 0.48 mmol), iPr_2NEt (52 µL, 0.30 mmol) and acceptor **3a** (40.0 mg, 0.084 mmol) were used. The reaction was stirred at 45 °C for 15 h. ^{1}H NMR spectroscopy of the crude reaction mixture showed β-only product. Purification by column chromatography (4:1; cyclohexane/ EtOAc) afforded the desired product **2i** as a colourless syrup (74 mg, quantitative yield). R_f = 0.7 (7:3; cyclohexane/ EtOAc); ^{1}H NMR (600 MHz, Chloroform-*d*) δ 7.43 – 7.19 (m, 30H, Ar-CH), 4.98 – 4.93 (m, 3H, 3 × $PhCH_2$), 4.86 (d, J = 11.6 Hz, 2H, 2 × $PhCH_2$), 4.80 (d, J = 12.2 Hz, 1H, $PhCH_2$), 4.79 – 4.74 (m, 2H, 2 × $PhCH_2$), 4.66 (d, J = 12.2 Hz, 1H, $PhCH_2$), 4.62 (d, J = 10.9 Hz, 1H, $PhCH_2$), 4.60 (d, J = 3.5 Hz, 1H, H-1), 4.53 (d, J = 11.9 Hz, 1H, $PhCH_2$), 4.46 (d, J = 11.9 Hz, 1H, $PhCH_2$), 4.42 (s, 1H, H-1'), 4.28 (dd, J = 11.1, 3.2 Hz, 1H, H-6a), 3.98 (t, J = 9.3 Hz, 1H, H-3), 3.95 (d, J = 3.0 Hz, 1H, H-2'), 3.76 – 3.71 (m, 1H, H-5), 3.65 – 3.60 (m, 2H, H-4, H-6b), 3.58 (t, J = 9.3 Hz, 1H, H-4'), 3.48 (dd, J = 9.6, 3.5 Hz, 1H, H-2), 3.44 (dd, J = 9.4, 3.0 Hz, 1H, H-3'), 3.35 (s, 3H, OCH_3), 3.31 (dq, J = 9.2, 6.1 Hz, 1H, H-5'), 1.34 (d, J = 6.1 Hz, 3H, H-6'). ^{13}C NMR (151 MHz, Chloroform-*d*) δ 138.9 (4ºC), 138.8 (4ºC), 138.6 (4ºC), 138.4 (4ºC), 138.23 (4ºC), 138.21 (4ºC), 128.5 (Ar-*C*H), 128.36 (Ar-*C*H), 128.35 (Ar-*C*H), 128.34 (Ar-*C*H), 128.31 (Ar-*C*H), 128.23 (Ar-*C*H), 128.16 (Ar-*C*H), 128.11 (Ar-*C*H), 128.09 (Ar-*C*H), 128.06 (Ar-*C*H), 127.89 (Ar-*C*H), 127.88 (Ar-*C*H), 127.67 (Ar-*C*H), 127.66 (Ar-*C*H), 127.58 (Ar-*C*H), 127.57 (Ar-*C*H), 127.54 (Ar-*C*H), 127.4 (Ar-*C*H), 101.4 (C-1', $^1J_{1CH}$ = 156.1 Hz, from coupled HSQC), 98.3 (C-1, $^1J_{1CH}$ = 169.2 Hz, from coupled HSQC), 82.0 (C-3'), 81.8 (C-3), 80.2 (C-4), 79.9 (C-2), 77.8 (C-4'), 75.7 (Ph*C*H_2), 75.4 (Ph*C*H_2), 75.2 (Ph*C*H_2), 74.3 (C-2'), 74.0 (Ph*C*H_2), 73.5 (Ph*C*H_2), 72.0 (C-5'), 71.3 (Ph*C*H_2), 70.0 (C-5), 67.2 (C-6), 55.2 (O*C*H_3), 18.0 (C-6'). NMR data were consistent with the literature.[34] ESI-HRMS for $C_{55}H_{64}NO_{10}^+$ $(M+NH_4)^+$ calculated: 898.4525; found: 898.4531.

Methyl 2,3,4-tri-*O*-benzyl-6-*O*-(2,3-di-*O*-benzyl-4-*O*-*p*-methylbenzyl-β-L-rhamnosyl)-α-D-glucopyranoside 2k

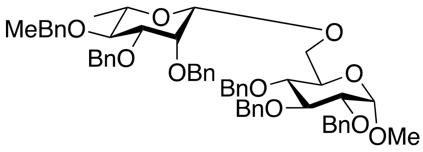

Following general procedure D, hemiacetal **1k** (45 mg, 0.1 mmol), Ph_3PO (28 mg, 0.1 mmol), $(COCl)_2$ (10 μL, 0.12 mmol), LiI (54 mg, 0.4 mmol), iPr_2NEt (44 μL, 0.25 mmol) and acceptor **3a** (33 mg, 0.07 mmol) were used. The reaction was stirred at 30 °C for 20 h. ^{1}H NMR spectroscopy of the crude reaction mixture showed β-only product. Purification by column chromatography (9:1 to 6:4; pentane/ Et_2O) afforded the desired product **2j** as a colourless syrup (64 mg, quantitative yield). R_f = 0.4 (4:1; cyclohexane/EtOAc); Reaction at 45 °C gave desired disaccharide **2j** as an α:β anomeric mixture (α/β 18:82). ^{1}H NMR (600 MHz, Chloroform-*d*) δ 7.45 – 7.40 (m, 2H, Ar-CH), 7.40 – 7.17 (m, 25H, Ar-CH), 7.12 (d, J = 7.8 Hz, 2H, Ar-CH), 4.97 (s, 1H, $PhCH_2$), 4.96 – 4.94 (m, 1H, $PhCH_2$), 4.90 (d, J = 10.6 Hz, 1H, $PhCH_2$), 4.86 (d, J = 11.6 Hz, 2H, 2 × $PhCH_2$), 4.80 (d, J = 12.1 Hz, 1H, $PhCH_2$), 4.78 – 4.76 (m, 2H, 2 × $PhCH_2$), 4.66 (d, J = 12.1 Hz, 1H, $PhCH_2$), 4.60 (d, J = 3.5 Hz, 1H, H-1), 4.57 (d, J = 10.6 Hz, 1H, $PhCH_2$), 4.53 (d, J = 11.9 Hz, 1H, $PhCH_2$), 4.47 (d, J = 11.9 Hz, 1H, $PhCH_2$), 4.42 (s, 1H, H-1'), 4.27 (dd, J = 11.1, 3.2 Hz, 1H, H-6a), 3.98 (t, J = 9.3 Hz, 1H, H-3), 3.94 (d, J = 3.0 Hz, 1H, H-2'), 3.73 (ddd, J = 10.1, 3.2, 1.9 Hz, 1H, H-5), 3.65 – 3.60 (m, 2H, H-4, H-6b), 3.58 (t, J = 9.3 Hz, 1H, H-4'), 3.50 – 3.46 (m, 1H, H-2), 3.43 (dd, J = 9.4, 3.0 Hz, 1H, H-3'), 3.34 (s, 3H, OCH_3), 3.29 (dq, J = 9.2, 6.2 Hz, 1H, H-5'), 2.32 (s, 3H, CH_3), 1.34 (d, J = 6.1 Hz, 3H, H-6'). ^{13}C NMR (151 MHz, Chloroform-*d*) δ 138.9 (4ºC), 138.8 (4ºC), 138.4 (4ºC), 138.3 (4ºC), 138.2 (4ºC), 137.4 (4ºC), 135.5 (4ºC), 129.0 (Ar-*C*H), 128.5 (Ar-*C*H), 128.37 (Ar-*C*H), 128.35 (Ar-*C*H), 128.31 (Ar-*C*H), 128.25 (Ar-*C*H), 128.22 (Ar-*C*H), 128.17 (Ar-*C*H), 128.12 (Ar-*C*H), 128.09 (Ar-*C*H), 127.90 (Ar-*C*H), 127.89 (Ar-*C*H), 127.7 (Ar-*C*H), 127.58 (Ar-*C*H), 127.56 (Ar-*C*H), 127.54 (Ar-*C*H), 127.4 (Ar-*C*H), 101.4 (C-1', $^1J_{CH}$ = 154.0 Hz, from coupled HSQC), 98.3 (C-1, $^1J_{CH}$ = 170.8 Hz, from coupled HSQC), 82.0 (C-3'), 81.9 (C-3), 80.1 (C-4'), 79.9 (C-2), 77.8 (C-4), 75.7 (Ph*C*H_2), 75.3 (Ph*C*H_2), 75.2 (Ph*C*H_2), 74.4 (C-2'), 74.0 (Ph*C*H_2), 73.5 (Ph*C*H_2), 72.0 (C-5'), 71.4 (Ph*C*H_2), 70.0 (C-5), 67.2 (C-6), 55.2 (O*C*H_3), 21.2 (*C*H_3), 18.0 (C-6'). ESI-HRMS $C_{56}H_{66}NO_{10}^+$ $(M+NH_4)^+$ calculated: 912.4681; found: 913.4725.

Methyl 2,3,4-tri-*O*-benzyl-6-*O*-(2,3-di-*O*-benzyl-4-*O*-benzoyl-β-L-rhamnosyl)-α-D-glucopyranoside 2l

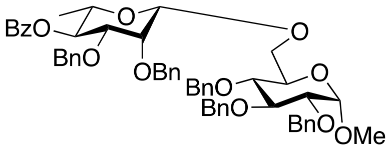

Following general procedure E, hemiacetal **1l** (45 mg, 0.1 mmol), Ph_3PO (14 mg, 0.05 mmol), $(COCl)_2$ (10 µL, 0.12 mmol), LiI (54 mg, 0.4 mmol), iPr_2NEt (70 µL, 0.4 mmol) and acceptor 3a (33 mg, 0.07 mmol) were used. The reaction was stirred at 45 °C for 24 h. ^1H NMR spectroscopy of the crude reaction mixture showed β-only product. Purification by column chromatography (9:1 to 7:3; pentane/ Et_2O) afforded the desired product 2k as a colourless syrup (27 mg, 43% yield). R_f = 0.5 (7:3; cyclohexane/ EtOAc); ^1H NMR (500 MHz, Chloroform-*d*) δ 8.00 (dd, *J* = 8.3, 1.4 Hz, 2H, Ar-CH), 7.61 – 7.55 (m, 1H, Ar-CH), 7.48 – 7.03 (m, 27H, Ar-CH), 5.43 (t, *J* = 9.6 Hz, 1H, H-4’), 4.99 – 4.95 (m, 2H, 2 × $PhCH_2$), 4.91 – 4.86 (m, 2H, 2 × $PhCH_2$), 4.83 – 4.73 (m, 3H, 3 × $PhCH_2$), 4.68 (d, *J* = 12.2 Hz, 1H, $PhCH_2$), 4.62 (d, *J* = 3.6 Hz, 1H, H-1), 4.51 (s, 1H, H-1’), 4.47 (d, *J* = 12.6 Hz, 1H, $PhCH_2$), 4.31 (dd, *J* = 11.1, 3.2 Hz, 1H, H-6a), 4.26 (d, *J* = 12.6 Hz, 1H, $PhCH_2$), 4.02 (d, *J* = 3.1 Hz, 1H, H-2’), 4.01 – 3.95 (m, H-3), 3.79 – 3.72 (m, 1H, H-5), 3.68 – 3.63 (m, 2H, H-4, H-6b), 3.54 – 3.45 (m, 3H, H-3’, H-2, H-5’), 3.36 (s, 3H, OCH_3), 1.26 (d, *J* = 6.2 Hz, 3H, H-6’). ^{13}C NMR (126 MHz, Chloroform-*d*) δ 165.6 (C=O), 138.9 (4ºC), 138.6 (4ºC), 138.4 (4ºC), 138.2 (4ºC), 137.7 (4ºC), 133.1 (4ºC), 130.1 (4ºC), 129.8 (Ar-*C*H), 128.5 (Ar-*C*H), 128.38 (Ar-*C*H), 128.35 (Ar-*C*H), 128.31 (Ar-*C*H), 128.24 (Ar-*C*H), 128.18 (Ar-*C*H), 128.17 (Ar-*C*H), 128.13 (Ar-*C*H), 127.92 (Ar-*C*H), 127.89 (Ar-*C*H), 127.7 (Ar-*C*H), 127.63 (Ar-*C*H), 127.58 (Ar-*C*H), 127.57 (Ar-*C*H), 127.4 (Ar-*C*H), 101.3 (C-1’, $^1J_{1CH}$ = 155.0 Hz, from coupled HSQC), 98.4 (C-1, $^1J_{1CH}$ = 174.0 Hz, from coupled HSQC), 81.9 (C-3), 79.8 (C-2), 78.4 (C-3’), 77.8 (C-4), 75.7 (Ph*C*H_2), 75.2 (Ph*C*H_2), 74.1 (Ph*C*H_2), 73.7 (C-2’), 73.49 (Ph*C*H_2), 73.45 (C-4’), 70.9 (C-5’), 70.7 (Ph*C*H_2), 70.0 (C-5), 67.2 (C-6), 55.2 (O*C*H_3), 17.7 (C-6’). ESI-HRMS for $C_{55}H_{62}NO_{11}^+$ $(M+NH_4)^+$ calculated: 912.4317; found: 912.4318.

Methyl (2,3,4,6-tetra-*O*-benzyl-β-D-mannopyranosyl)-(1→4)-2,3,6-tri-*O*-benzyl-α-D-glucopyranoside 4b

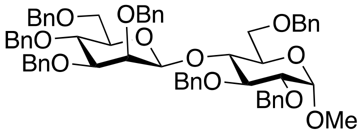

Following the general procedure C, hemiacetal **1a** (54 mg, 0.10 mmol) and acceptor **3b** (32 mg, 0.069 mmol) were used. The reaction was stirred at 45 °C for 24 h. ^{1}H NMR spectroscopy of the crude reaction mixture gave an α/β = 5:95. Purification by column chromatography (97:3; CH_2Cl_2/Et_2O) gave **4b** as a yellow oil (41 mg, 60% yield).

β-anomer

^{1}H NMR (500 MHz, Chloroform-*d*): δ 7.43 – 7.11 (m, 35H, ArCH), 5.14 (d, *J* = 11.3 Hz, 1H, C*H*HPh), 4.85 (d, *J* = 10.9 Hz, 1H, C*H*HPh), 4.84 (d, *J* = 12.1 Hz, 1H, C*H*HPh), 4.81 (d, *J* = 12.1 Hz, 1H, C*H*HPh), 4.77 (d, *J* = 12.2 Hz, 1H, C*H*HPh), 4.75 (d, *J* = 11.3 Hz, 1H, C*H*HPh), 4.60 (d, *J* = 12.1 Hz, 1H, C*H*HPh), 4.58 (d, *J* = 3.6 Hz, 1H, H-1), 4.57 (d, *J* = 12.1 Hz, 1H, C*H*HPh), 4.53 (d, *J* = 10.8 Hz, 1H, C*H*HPh), 4.48 (d, *J* = 11.9 Hz, 1H, C*H*HPh), 4.45 (d, *J* = 11.8 Hz, 1H, C*H*HPh), 4.44 (d, *J* = 12.1 Hz, 1H, C*H*HPh), 4.42 (s, 1H, H-1'), 4.37 (d, *J* = 12.1 Hz, 1H, C*H*HPh), 4.36 (d, *J* = 12.2 Hz, 1H, C*H*HPh), 3.93 – 3.89 (m, 2H, H-3, H-4), 3.87 (t, *J* = 9.5 Hz, 1H, H-4'), 3.76 – 3.69 (m, 1H, H-5), 3.70 (d, *J* = 3.1 Hz, 1H, H-2'), 3.67 (dd, *J* = 11.2, 1.8 Hz, 1H, H-6a'), 3.59 – 3.55 (m, 2H, H-6a, H-6b), 3.54 (dd, *J* = 11.2, 5.4 Hz, 1H, H-6b'), 3.49 – 3.43 (m, 1H, H-2), 3.37 (s, 3H, OCH_3), 3.31 – 3.24 (m, 1H, H-5'), 3.28 (dd, *J* = 9.5, 3.1 Hz, 1H, H-3'). ^{13}C NMR (126 MHz, Chloroform-*d*): δ 139.8 (C), 139.04 (C), 138.98 (C), 138.7 (C), 138.48 (C), 138.46 (C), 137.9 (C), 128.7 (CH), 128.5 (CH), 128.4 (CH), 128.30 (CH), 128.25 (CH), 128.2 (CH), 128.13 (CH), 128.08 (CH), 128.06 (CH), 127.98 (CH), 127.95 (CH), 127.91 (CH), 127.85 (CH), 127.8 (CH), 127.73 (CH), 127.68 (CH), 127.6 (CH), 127.4 (CH), 127.3 (CH), 127.1 (CH), 101.0 (C-1'), 98.5 (C-1), 82.7 (C-3'), 80.5 (C-3), 79.3 (C-2), 77.3 (C-4), 76.3 (C-5'), 75.4 (PhCH_2), 75.2 (C-2'), 75.1 (PhCH_2), 75.0 (C-4'), 74.2 (PhCH_2), 73.8 (PhCH_2), 73.7 (PhCH_2), 73.6 (PhCH_2), 71.8 (PhCH_2), 69.8 (C-5), 69.7 (C-6'), 68.9 (C-6), 55.4 (OCH_3). NMR data were consistent with literature data.[35]

α-anomer

^{1}H NMR (500 MHz, Chloroform-*d*): δ 3.39 (s, 3H, OCH_3).

Methyl (2,3,4,6-tetra-*O*-benzyl-β-D-mannopyranosyl)-(1→3)-2,4,6-tri-*O*-benzyl-α-D-glucopyranoside 4c

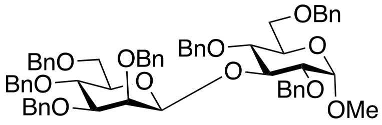

Following the general procedure C, hemiacetal **1a** (54 mg, 0.10 mmol), Ph_3PO (14 mg, 0.050 mmol, 0.5 eq) and acceptor **3c** (32 mg, 0.069 mmol) were used. The reaction was stirred at 45 °C for 24 h. ^{1}H NMR spectroscopy of the crude reaction mixture gave an α/β = 1:99. Purification by column chromatography (97:3; CH_2Cl_2/Et_2O) gave **4c** as a yellow oil (58 mg, 84% yield). ESI-HRMS for $C_{62}H_{66}O_{11}Na^+$ $(M+Na)^+$ calculated: 1009.4497; found: 1009.4496.

β-anomer

^{1}H NMR (500 MHz, Chloroform-*d*): δ 7.49 – 7.44 (m, 2H, ArCH), 7.33 – 7.06 (m, 33H, ArCH), 5.27 (d, *J* = 11.1 Hz, 1H, C*H*HPh), 4.92 (s, 2H, 2 x C*H*HPh), 4.85 (d, *J* = 10.7 Hz, 1H, C*H*HPh), 4.79 (s, 1H, H-1'), 4.68 (d, *J* = 3.4 Hz, 1H, H-1), 4.55 (d, *J* = 12.2 Hz, 1H, C*H*HPh), 4.54 (d, *J* = 10.8 Hz, 1H, C*H*HPh), 4.51 – 4.43 (m, 3H, 3 x C*H*HPh), 4.43 (s, 2H, 2 x C*H*HPh), 4.41 – 4.36 (m, 3H, 3 x C*H*HPh), 4.18 (dd, *J* = 9.6, 8.6 Hz, 1H, H-3), 3.97 (t, *J* = 9.6 Hz, 1H, H-4'), 3.77 (d, *J* = 2.9 Hz, 1H, H-2'), 3.75 – 3.66 (m, 4H, H-5, H-6a', H-6b', H-6a), 3.66 – 3.60 (m, 1H, H-6b), 3.55 (dd, *J* = 9.9, 8.6 Hz, 1H, H-4), 3.46 (dd, *J* = 9.7, 3.4 Hz, 1H, H-2), 3.39 – 3.32 (m, 2H, H-3', H-5'), 3.35 (s, 3H, OCH_3). ^{13}C NMR (126 MHz, Chloroform-*d*): δ 139.3 (C), 139.1 (C), 138.9 (C), 138.64 (C), 138.56 (C), 138.1 (C), 138.0 (C), 128.7 (CH), 128.48 (CH), 128.46 (CH), 128.4 (CH), 128.22 (CH), 128.20 (CH), 128.16 (CH), 128.13 (CH), 128.11 (CH), 128.0 (CH), 127.8 (CH), 127.74 (CH), 127.68 (CH), 127.4 (CH), 127.3 (CH), 102.6 (C-1', $^1J_{1CH}$ = 158.3 Hz, from coupled HSQC), 97.5 (C-1, $^1J_{1CH}$ =170.0 Hz, from coupled HSQC), 83.0 (C-3'), 80.9 (C-2), 80.6 (C-3), 76.3 (C-4), 76.0 (C-5'), 75.3 (Ph*C*H_2), 75.1 (C-4'), 75.0 (C-2'), 74.9 (Ph*C*H_2), 74.0 (Ph*C*H_2), 73.6 (Ph*C*H_2), 73.5 (Ph*C*H_2), 73.1 (Ph*C*H_2), 72.0 (Ph*C*H_2), 70.0 (C-5), 69.7 (C-6'), 68.8 (C-6), 55.2 (OCH_3).

α-anomer

^{1}H NMR (500 MHz, Chloroform-*d*): δ 3.31 (s, 3H, OCH_3).

Methyl (2,3,4,6-tetra-*O*-benzyl-β-D-mannopyranosyl)-(1→3)-2-*O*-benzyl-4,6-*O*-benzylidene-α-D-glucopyranoside 4d

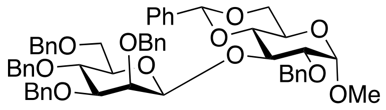

Following the general procedure B, hemiacetal **1a** (54 mg, 0.10 mmol), acceptor **3d** (26 mg, 0.070 mmol) and iPr_2NEt (84 µL, 0.50 mmol) were used. The reaction was stirred at 60 °C for 24 h. ^1H NMR spectroscopy of the crude reaction mixture showed β-only product. Purification by column chromatography (97:3; CH_2Cl_2/Et_2O) gave **4d** as a yellowish oil (33 mg, 52% yield). ^1H NMR (600 MHz, Chloroform-*d*): δ 7.50 – 7.41 (m, 4H, ArCH), 7.32 – 7.12 (m, 26H, ArCH), 5.50 (s, 1H, PhC*H*), 4.93 (d, *J* = 12.2 Hz, 1H, C*H*HPh), 4.87 (d, *J* = 12.2 Hz, 1H, C*H*HPh), 4.82 (d, *J* = 10.7 Hz, 1H, C*H*HPh), 4.70 (s, 1H, H-1'), 4.57 (d, *J* = 11.7 Hz, 1H, C*H*HPh), 4.57 (d, *J* = 3.7 Hz, 1H, H-1), 4.52 (d, *J* = 10.7 Hz, 1H, C*H*HPh), 4.51 (s, 2H, 2 x C*H*HPh), 4.47 (d, *J* = 11.8 Hz, 1H, C*H*HPh), 4.44 (d, *J* = 11.9 Hz, 1H, C*H*HPh), 4.41 (d, *J* = 11.8 Hz, 1H, C*H*HPh), 4.22 (dd, *J* = 10.2, 4.8 Hz, 1H, H-6a), 4.19 (t, *J* = 9.3 Hz, 1H, H-3), 3.93 (t, *J* = 9.6 Hz, 1H, H-4'), 3.85 (d, *J* = 2.9 Hz, 1H, H-2'), 3.80 (td, *J* = 9.9, 4.7 Hz, 1H, H-5), 3.72 (dd, *J* = 11.3, 4.7 Hz, 1H, H-6a'), 3.70 – 3.66 (m, 2H, H-6b', H-6b), 3.63 (t, *J* = 9.4 Hz, 1H, H-4), 3.53 (dd, *J* = 9.3, 3.7 Hz, 1H, H-2), 3.38 (dd, *J* = 9.5, 2.9 Hz, 1H, H-3'), 3.37 (s, 3H, OCH_3), 3.30 (ddd, *J* = 9.7, 4.7, 2.1 Hz, 1H, H-5'). ^{13}C NMR (151 MHz, Chloroform-*d*): δ 139.4 (C), 138.9 (C), 138.6 (C), 138.5 (C), 138.0 (C), 137.5 (C), 128.9 (CH), 128.7 (CH), 128.5 (CH), 128.4 (CH), 128.24 (CH), 128.20 (CH), 128.18 (CH), 128.14 (CH), 128.12 (CH), 128.09 (CH), 127.7 (CH), 127.7 (CH), 127.4 (CH), 127.3 (CH), 126.3 (CH), 102.1 (C-1'), 101.6 (Ph*C*H), 98.7 (C-1), 82.8 (C-3'), 80.33 (C-2), 80.29 (C-4), 77.4 (C-3), 76.2 (C-5'), 75.2 (Ph*C*H_2), 74.92 (C-2'), 74.88 (C-4'), 74.0 (Ph*C*H_2), 73.7 (Ph*C*H_2), 73.5 (Ph*C*H_2), 71.8 (Ph*C*H_2), 69.6 (C-6), 69.1 (C-6'), 62.6 (C-5), 55.5 (OCH_3). NMR data were consistent with literature data.[36]

Phenyl 2,3,4,6-tetra-*O*-benzyl-β-D-mannopyranoside 4e

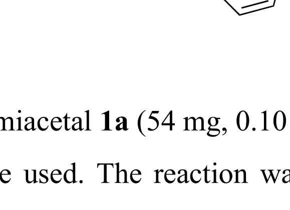

Following the general procedure B, hemiacetal **1a** (54 mg, 0.10 mmol), acceptor **3e** (7 mg, 0.07 mmol) and iPr_2NEt (42 µL, 0.25 mmol) were used. The reaction was stirred at 25 °C for 24 h. ^1H NMR

spectroscopy of the crude reaction mixture gave an α/β = 3:97. Purification by column chromatography (CH_2Cl_2) gave **4e** as a white solid (39 mg, 91% yield).

Reaction at 30 °C gave α/β = 6:94.

β-anomer

^{1}H NMR (500 MHz, Chloroform-*d*): δ 7.58 – 7.43 (m, 2H, ArCH), 7.38 – 7.18 (m, 20H, ArCH), 7.08 – 6.92 (m, 3H, ArCH), 5.09 (d, *J* = 12.3 Hz, 1H, C*H*HPh), 5.00 (d, *J* = 12.4 Hz, 1H, C*H*HPh), 4.98 (d, *J* = 0.8 Hz, 1H, H-1), 4.93 (d, *J* = 10.9 Hz, 1H, C*H*HPh), 4.60 (d, *J* = 11.9 Hz, 1H, C*H*HPh), 4.59 (d, *J* = 11.9 Hz, 1H, C*H*HPh), 4.58 (d, *J* = 10.9 Hz, 1H, C*H*HPh), 4.55 (d, *J* = 11.9 Hz, 1H, C*H*HPh), 4.53 (d, *J* = 11.9 Hz, 1H, C*H*HPh), 4.09 (dd, *J* = 3.0, 0.8 Hz, 1H, H-2), 3.97 (t, *J* = 9.4 Hz, 1H, H-4), 3.87 (dd, *J* = 10.9, 2.0 Hz, 1H, H-6a), 3.77 (dd, *J* = 10.9, 6.2 Hz, 1H, H-6b), 3.64 – 3.58 (m, 1H, H-5), 3.60 (dd, *J* = 9.2, 3.0 Hz, 1H, H-3). ^{13}C NMR (126 MHz, Chloroform-*d*): δ 157.4 (C), 138.7 (C), 138.6 (C), 138.4 (C), 138.2 (C), 129.58 (CH), 128.57 (CH), 128.55 (CH), 128.5 (CH), 128.4 (CH), 128.3 (CH), 128.2 (CH), 127.9 (CH), 127.84 (CH), 127.78 (CH), 127.7 (CH), 127.6 (CH), 122.5 (CH), 116.5 (CH), 99.5 (C-1), 82.3 (C-3), 76.3 (C-5), 75.3 (Ph*C*H_2), 74.9 (C-4), 74.3 (Ph*C*H_2 and C-2), 73.6 (Ph*C*H_2), 71.9 (Ph*C*H_2), 69.7 (C-6). NMR data were consistent with literature data.[37]

α-anomer

^{1}H NMR (500 MHz, Chloroform-*d*): δ 5.59 (d, *J* = 2.0 Hz, 1H, H-1).

1-Naphthyl 2,3,4,6-tetra-*O*-benzyl-β-D-mannopyranoside 4f

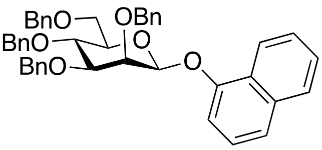

Following the general procedure B, hemiacetal **1a** (54 mg, 0.10 mmol), acceptor **3f** (10 mg, 0.069 mmol) and iPr$_2$NEt (42 μL, 0.25 mmol) were used. The reaction was stirred at 25 °C for 24 h. ^{1}H NMR spectroscopy of the crude reaction mixture gave an α/β = 1:99. Purification by column chromatography (CH_2Cl_2) gave **4f** as a white solid (38 mg, 83% yield). ESI-HRMS for $C_{44}H_{42}O_6Na^+$ $(M+Na)^+$ calculated: 689.2874; found: 689.2880.

β-anomer

^{1}H NMR (500 MHz, Chloroform-*d*): δ 8.13 (dd, *J* = 8.4, 1.3 Hz, 1H, ArCH), 7.82 – 7.77 (m, 1H, ArCH), 7.60 – 7.56 (m, 2H, ArCH), 7.51 (d, *J* = 8.2 Hz, 1H, ArCH), 7.47 (ddd, *J* = 8.2, 6.8, 1.4 Hz, 1H, ArCH), 7.42 (ddd, *J* = 8.2, 6.8, 1.4 Hz, 1H, ArCH), 7.37 – 7.20 (m, 19H, ArCH), 7.15 (dd, *J* = 7.8, 1.0 Hz, 1H, ArCH), 5.24 (d, *J* = 12.1 Hz, 1H, C*H*HPh), 5.17 (d, *J* = 0.9 Hz, 1H, H-1), 5.09 (d, *J* = 12.2 Hz, 1H, C*H*HPh), 4.93 (d, *J* = 10.9 Hz, 1H, C*H*HPh), 4.65 (d, *J* = 11.8 Hz, 1H, C*H*HPh), 4.61 (d, *J* = 10.9 Hz,

1H, C*H*HPh), 4.59 (d, *J* = 11.9 Hz, 1H, C*H*HPh), 4.59 (d, *J* = 11.9 Hz, 1H, C*H*HPh), 4.54 (d, *J* = 11.9 Hz, 1H, C*H*HPh), 4.27 (dd, *J* = 2.9, 0.8 Hz, 1H, H-2), 4.03 (t, *J* = 9.2 Hz, 1H, H-4), 3.90 (dd, *J* = 10.8, 2.1 Hz, 1H, H-6a), 3.79 (dd, *J* = 10.9, 6.3 Hz, 1H, H-6b), 3.74 – 3.65 (m, 2H, H-3, H-5). ^{13}C NMR (126 MHz, Chloroform-*d*): δ 153.5 (C), 138.8 (C), 138.6 (C), 138.4 (C), 138.2 (C), 134.6 (C), 128.6 (CH), 128.51 (CH), 128.45 (CH), 128.43 (CH), 128.38 (CH), 128.2 (CH), 127.9 (CH), 127.8 (CH), 127.74 (CH), 127.69 (CH), 127.5 (CH), 126.4 (CH), 126.1 (CH), 126.0 (CH), 125.6 (CH), 122.3 (CH), 122.2 (CH), 109.2 (CH), 100.0 (C-1, $^1J_{1CH}$ = 155.5 Hz, from coupled HSQC), 82.3 (C-3), 76.4 (C-5), 75.2 (Ph*C*H$_2$ and C-2), 75.0 (C-4), 74.7 (Ph*C*H$_2$), 73.6 (Ph*C*H$_2$), 72.1 (Ph*C*H$_2$), 69.7 (C-6).

α-anomer

^{1}H NMR (500 MHz, Chloroform-*d*): δ 5.76 (d, *J* = 1.9 Hz, 1H, H-1).

***p*-Nitrophenyl 2,3,4,6-tetra-*O*-benzyl-β-D-mannopyranoside 4g**

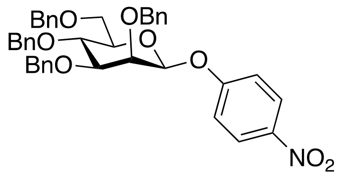

Following the general procedure B, hemiacetal **1a** (54 mg, 0.10 mmol), acceptor **3g** (9.7 mg, 0.070 mmol) and iPr$_2$NEt (42 μL, 0.25 mmol) were used. The reaction was stirred at 45 °C for 24 h. ^{1}H NMR spectroscopy of the crude reaction mixture gave an α/β = 1:99. Purification by column chromatography (CH$_2$Cl$_2$) gave **4g** as a white solid (34 mg, 74% yield). ESI-HRMS for $C_{40}H_{39}NO_8Na^+$ (M+Na)$^+$ calculated: 684.2568; found: 684.2566.

β-anomer

^{1}H NMR (500 MHz, Chloroform-*d*): δ 8.16 – 8.09 (m, 2H, ArCH), 7.52 – 7.46 (m, 2H, ArCH), 7.40 – 7.17 (m, 18H, ArCH), 7.08 – 7.01 (m, 2H, ArCH), 5.06 (d, *J* = 1.0 Hz, 1H, H-1), 5.02 (d, *J* = 12.2 Hz, 1H, C*H*HPh), 4.97 (d, *J* = 12.3 Hz, 1H, C*H*HPh), 4.92 (d, *J* = 10.9 Hz, 1H, C*H*HPh), 4.63 (d, *J* = 11.9 Hz, 1H, C*H*HPh), 4.59 (d, *J* = 11.7 Hz, 1H, C*H*HPh), 4.58 (d, *J* = 10.9 Hz, 1H, C*H*HPh), 4.56 (d, *J* = 11.8 Hz, 1H, C*H*HPh), 4.50 (d, *J* = 11.8 Hz, 1H, C*H*HPh), 4.11 (dd, *J* = 2.9, 1.0 Hz, 1H, H-2), 3.96 (t, *J* = 9.2 Hz, 1H, H-4), 3.85 (dd, *J* = 10.6, 1.9 Hz, 1H, H-6a), 3.71 (dd, *J* = 10.6, 6.7 Hz, 1H, H-6b), 3.69 – 3.62 (m, 1H, H-5), 3.64 (dd, *J* = 9.1, 2.8 Hz, 1H, H-3). ^{13}C NMR (126 MHz, Chloroform-*d*): δ 162.0 (C), 142.8 (C), 138.4 (C), 138.3 (C), 138.2 (C), 138.0 (C), 128.63 (CH), 128.56 (CH), 128.51 (CH), 128.47 (CH), 128.4 (CH), 128.2 (CH), 127.98 (CH), 127.97 (CH), 127.9 (CH), 127.8 (CH), 125.9 (CH), 116.5 (CH), 98.7 (C-1, $^1J_{1CH}$ = 159.0 Hz, from coupled HSQC), 82.0 (C-3), 76.4 (C-5), 75.3 (Ph*C*H$_2$), 74.7 (C-4), 74.6 (Ph*C*H$_2$), 74.3 (C-2), 73.6 (Ph*C*H$_2$), 72.3 (Ph*C*H$_2$), 69.4 (C-6).

α-anomer

^{1}H NMR (500 MHz, Chloroform-*d*): δ 5.64 (d, *J* = 2.2 Hz, 1H, H-1).

***p*-Methoxyphenyl (2,3,4,6-tetra-*O*-benzyl-β-D-mannopyranosyl)-(1→4)-2-azido-3,6-di-*O*-benzyl-β-D-glucopyranoside 4h**

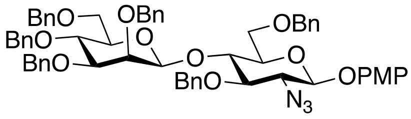

Following the general procedure B, hemiacetal **1a** (108 mg, 0.200 mmol), Ph_3PO (28 mg, 0.10 mmol), oxalyl chloride (20 μL, 0.24 mmol), LiI (107 mg, 0.800 mmol), acceptor **3h** (49 mg, 0.10 mmol) and iPr_2NEt (0.14 mL, 0.80 mmol) were used. The reaction was stirred at 45 °C for 22 h. ^{1}H NMR spectroscopy of the crude reaction mixture showed β-only product. Purification by column chromatography (90:10 to 75:25; cyclohexane/Et_2O) gave **4h** as a colourless syrup (45 mg, 45% yield). ESI-HRMS for $C_{62}H_{69}N_4O_{11}^+$ $(M+NH_4)^+$ calculated: 1045.4957; found: 1045.4957.

^{1}H NMR (600 MHz, Chloroform-*d*): δ 7.41 – 7.38 (m, 2H, ArCH), 7.37 – 7.33 (m, 2H, ArCH), 7.33 – 7.13 (m, 26H, ArCH), 7.05 – 6.99 (m, 2H, ArCH), 6.83 – 6.77 (m, 2H, ArCH), 5.18 (d, *J* = 11.3 Hz, 1H, C*H*HPh), 4.86 (d, *J* = 12.0 Hz, 1H, C*H*HPh), 4.86 (d, *J* = 10.9 Hz, 1H, C*H*HPh), 4.83 (d, *J* = 12.0 Hz, 1H, C*H*HPh), 4.70 (d, *J* = 11.3 Hz, 1H, C*H*HPh), 4.67 (d, *J* = 8.2 Hz, 1H, H-1), 4.57 (d, *J* = 12.1 Hz, 1H, C*H*HPh), 4.53 (d, *J* = 10.9 Hz, 1H, C*H*HPh), 4.50 (s, 1H, H-1'), 4.50 (d, *J* = 11.9 Hz, 1H, C*H*HPh), 4.48 (d, *J* = 11.9 Hz, 1H, C*H*HPh), 4.45 (d, *J* = 12.1 Hz, 1H, C*H*HPh), 4.40 (d, *J* = 12.1 Hz, 1H, C*H*HPh), 4.37 (d, *J* = 12.1 Hz, 1H, C*H*HPh), 4.00 (dd, *J* = 9.8, 8.8 Hz, 1H, H-4), 3.88 (t, *J* = 9.5 Hz, 1H, H-4'), 3.79 – 3.75 (m, 1H, H-2'), 3.77 (s, 3H, OCH_3), 3.73 – 3.67 (m, 2H, H-6a, H-6a'), 3.61 (dd, *J* = 11.1, 4.4 Hz, 1H, H-6b), 3.59 (dd, *J* = 9.9, 8.2 Hz, 1H, H-2), 3.53 (dd, *J* = 11.0, 5.6 Hz, 1H, H-6b'), 3.49 (ddd, *J* = 9.8, 4.3, 2.4 Hz, 1H, H-5), 3.44 (dd, *J* = 9.8, 8.8 Hz, 1H, H-3), 3.36 (dd, *J* = 9.4, 2.9 Hz, 1H, H-3'), 3.32 (ddd, *J* = 9.7, 5.6, 1.8 Hz, 1H, H-5'). ^{13}C NMR (151 MHz, Chloroform-*d*): δ 155.7 (C), 151.3 (C), 138.9 (C), 138.81 (C), 138.76 (C), 138.5 (C), 138.4 (C), 137.9 (C), 128.62 (CH), 128.53 (CH), 128.44 (CH), 128.35 (CH), 128.28 (CH), 128.2 (CH), 128.104 (CH), 128.096 (CH), 128.0 (CH), 127.9 (CH), 127.82 (CH), 127.78 (CH), 127.75 (CH), 127.65 (CH), 127.64 (CH), 127.55 (CH), 127.47 (CH), 127.4 (CH), 118.9 (CH), 114.7 (CH), 101.7 (C-1, $^1J_{CH}$ = 163.4 Hz, from coupled HSQC), 101.0 (C-1', $^1J_{CH}$ = 155.2 Hz, from coupled HSQC), 82.8 (C-3'), 81.2 (C-3), 77.0 (C-4), 76.2 (C-5'), 75.18 (C-5, C-2', Ph*C*H_2), 75.1 (Ph*C*H_2), 74.9 (C-4'), 74.4 (Ph*C*H_2), 73.7 (Ph*C*H_2), 73.5 (Ph*C*H_2), 71.9 (Ph*C*H_2), 69.7 (C-6'), 68.8 (C-6), 65.9 (C-2), 55.8 (OCH_3).

Methyl 2,3,6-tri-*O*-benzyl-4-*O*-(2,3,4-tri-*O*-benzyl-β-D-rhamnosyl)-α-D-glucopyranoside 5b

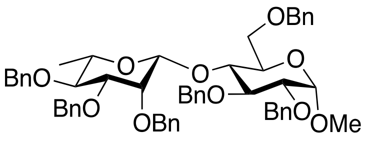

Following general procedure E, hemiacetal **1j** (44 mg, 0.1 mmol), Ph_3PO (14 mg, 0.05 mmol), $(COCl)_2$ (10 µL, 0.12 mmol), LiI (54 mg, 0.4 mmol), iPr_2NEt (70 µL, 0.4 mmol) and acceptor **3b** (33 mg, 0.07 mmol) were used. The reaction was stirred at 45 °C for 24 h. ^{1}H NMR spectroscopy of the crude reaction mixture showed β-only product. Purification by column chromatography (9:1 to 6:4; pentane/ Et_2O) afforded the desired product **5b** as a colourless syrup (48.2 mg, 78% yield). R_f = 0.6 (4:1; cyclohexane/ EtOAc); ^{1}H NMR (500 MHz, Chloroform-*d*) δ 7.44 – 7.39 (m, 2H, Ar-CH), 7.37 – 7.16 (m, 26H, Ar-CH), 7.16 – 7.12 (m, 2H, Ar-CH), 4.96 (d, *J* = 11.6 Hz, 1H, $PhCH_2$), 4.88 (d, *J* = 10.7 Hz, 1H, $PhCH_2$), 4.81 (s, 2H, 2 × $PhCH_2$), 4.73 (d, *J* = 12.1 Hz, 1H, $PhCH_2$), 4.66 (d, *J* = 3.5 Hz, 1H, H-1), 4.65 – 4.61 (m, 2H, 2 × $PhCH_2$), 4.61 (s, 1H, H-1'), 4.57 (d, *J* = 10.8 Hz, 1H, $PhCH_2$), 4.53 (d, *J* = 12.0 Hz, 1H, $PhCH_2$), 4.30 (d, *J* = 11.6 Hz, 1H, $PhCH_2$), 4.22 (d, *J* = 11.7 Hz, 1H, $PhCH_2$), 4.18 (d, *J* = 11.7 Hz, 1H, $PhCH_2$), 3.90 (dd, *J* = 10.6, 1.9 Hz, 1H, H-6a), 3.83 (t, *J* = 9.3 Hz, 1H, H-3), 3.80 – 3.70 (m, 2H, H-5, H-6b), 3.70 – 3.63 (m, 1H, H-4), 3.61 (d, *J* = 2.9 Hz, 1H, H-2'), 3.54 – 3.47 (m, 2H, H-2, H-4'), 3.44 (s, 3H, OCH_3), 3.23 – 3.14 (m, 1H, H-5'), 3.13 (dd, *J* = 9.5, 2.9 Hz, 1H, H-3'), 1.25 (d, *J* = 6.1 Hz, 3H, H-6'). ^{13}C NMR (126 MHz, Chloroform-*d*) δ 138.84 (4ºC), 138.79 (4ºC), 138.6 (4ºC), 138.42 (4ºC), 138.35 (4ºC), 138.0 (4ºC), 128.49 (Ar-*C*H), 128.48 (Ar-*C*H), 128.44 (Ar-*C*H), 128.38 (Ar-*C*H), 128.30 (Ar-*C*H), 128.21 (Ar-*C*H), 128.17 (Ar-*C*H), 128.14 (Ar-*C*H), 128.13 (Ar-*C*H), 128.0 (Ar-*C*H), 127.7 (Ar-*C*H), 127.6 (Ar-*C*H), 127.53 (Ar-*C*H), 127.51 (Ar-*C*H), 127.50 (Ar-*C*H), 127.4 (Ar-*C*H), 127.3 (Ar-*C*H), 127.1 (Ar-*C*H), 102.4 (C-1', $^1J_{1CH}$ = 159.0 Hz, from coupled HSQC), 97.9 (C-1, $^1J_{1CH}$ = 170.0 Hz, from coupled HSQC), 82.8 (C-3'), 82.1 (C-3), 80.0 (C-4'), 79.8 (C-2), 76.9 (C-4), 75.5 (Ph*C*H_2), 75.4 (Ph*C*H_2), 73.8 (C-2'), 73.7 (Ph*C*H_2), 73.4 (Ph*C*H_2), 73.1 (Ph*C*H_2), 71.8 (C-5'), 71.5 (Ph*C*H_2), 69.8 (C-5), 69.1 (C-6), 55.4 (O*C*H_3), 17.9 (C-6'). ESI-HRMS for $C_{55}H_{64}NO_{10}^+$ $(M+NH_4)^+$ calculated: 898.4525; found: 898.4567.

Methyl 2,4,6-tri-*O*-benzyl-3-*O*-(2,3,4-tri-*O*-benzyl-β-L-rhamnosyl)-α-D-glucopyranoside 5c

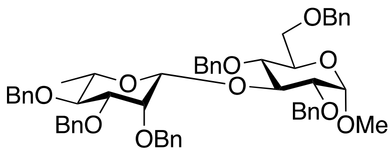

Following general procedure E, hemiacetal **1j** (44 mg, 0.1 mmol), Ph_3PO (28 mg, 0.1 mmol), $(COCl)_2$ (10 μL, 0.12 mmol), LiI (54 mg, 0.4 mmol), iPr_2NEt (70 μL, 0.4 mmol) and acceptor **3c** (33 mg, 0.07 mmol) were used. The reaction was stirred at 45 °C for 20 h. ^{1}H NMR spectroscopy of the crude reaction mixture showed β-only product. Purification by column chromatography (9:1 to 6:4; pentane/ Et_2O) afforded the desired product **5c** as a colourless syrup (54 mg, 88% yield). R_f = 0.4 (4:1; cyclohexane/ EtOAc); ^{1}H NMR (500 MHz, Chloroform-*d*) δ 7.45 – 7.40 (m, 2H, Ar-CH), 7.40 – 7.17 (m, 26H, Ar-CH), 7.04 – 7.01 (m, 2H, Ar-CH), 4.99 (d, *J* = 12.6 Hz, 1H, $PhCH_2$), 4.93 – 4.84 (m, 3H, 3 × $PhCH_2$), 4.77 (d, *J* = 12.6 Hz, 1H, $PhCH_2$), 4.65 – 4.64 (m, 2H, H-1', $PhCH_2$), 4.63 – 4.58 (m, 2H, H-1, $PhCH_2$), 4.48 (d, *J* = 12.0 Hz, 1H, $PhCH_2$), 4.44 (d, *J* = 11.8 Hz, 1H, $PhCH_2$), 4.30 (d, *J* = 11.8 Hz, 1H, $PhCH_2$), 4.26 – 4.19 (m, 2H, 2 × $PhCH_2$), 4.09 (t, *J* = 9.3 Hz, 1H, H-3), 3.74 – 3.61 (m, 4H, H-2', H-5, H-6a, H-6b), 3.60 – 3.44 (m, 3H, H-4', H-4, H-2), 3.36 (s, 3H, OCH_3), 3.25 (dd, *J* = 9.3, 6.1 Hz, 1H, H-5'), 3.20 (dd, *J* = 9.4, 2.9 Hz, 1H, H-3'), 1.35 (d, *J* = 6.1 Hz, 3H, H-6'). ^{13}C NMR (126 MHz, Chloroform-*d*) δ 139.1 (4ºC), 139.0 (4ºC), 138.5 (4ºC), 138.4 (4ºC), 138.3 (4ºC), 137.8 (4ºC), 128.5 (Ar-*C*H), 128.40 (Ar-*C*H), 128.36 (Ar-*C*H), 128.3 (Ar-*C*H), 128.23 (Ar-*C*H), 128.18 (Ar-*C*H), 128.15 (Ar-*C*H), 128.14 (Ar-*C*H), 128.11 (Ar-*C*H), 128.06 (Ar-*C*H), 127.8 (Ar-*C*H), 127.7 (Ar-*C*H), 127.50 (Ar-*C*H), 127.49 (Ar-*C*H), 127.46 (Ar-*C*H), 127.40 (Ar-*C*H), 127.35 (Ar-*C*H), 126.5 (Ar-*C*H), 102.6 (C-1', $^1J_{CH}$ = 156.0 Hz, from coupled HSQC), 99.2 (C-1, $^1J_{CH}$ = 171.0 Hz, from coupled HSQC), 83.0 (C-3'), 82.4 (C-3), 80.3 (C-4'), 78.6 (C-4), 77.1 (C-2), 75.5 (Ph*C*H_2), 74.6 (C-2'), 74.1 (Ph*C*H_2), 73.9 (Ph*C*H_2), 73.7 (Ph*C*H_2), 73.6 (Ph*C*H_2), 71.8 (C-5'), 71.5 (Ph*C*H_2), 69.6 (C-5), 68.5 (C-6), 55.2 (O*C*H_3), 18.1 (C-6'). ESI-HRMS for $C_{55}H_{64}NO_{10}^+$ $(M+NH_4)^+$ calculated: 898.4525; found: 898.4529.

Methyl 2-*O*-benzyl-4,6-benzylidene-3-*O*-(2,3,4-tri-*O*-benzyl-β-L-rhamnosyl)-α-D-glucopyranoside 5d

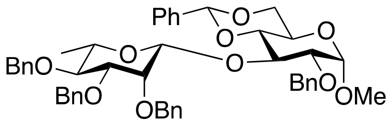

Following general procedure E, hemiacetal **1j** (44 mg, 0.1 mmol), Ph_3PO (28 mg, 0.1 mmol), $(COCl)_2$ (10 μL, 0.12 mmol), LiI (54 mg, 0.4 mmol), iPr_2NEt (70 μL, 0.4 mmol) and acceptor **3d** (26 mg, 0.07 mmol) were used. The reaction was stirred at 45 °C for 24 h. ^{1}H NMR spectroscopy of the crude reaction mixture showed β-only product. Purification by column chromatography (9:1 to 6:4; pentane/ Et_2O) afforded the desired product 5d as a colourless syrup (24.3 mg, 44% yield). R_f = 0.6 (4:1; cyclohexane/ EtOAc); Reaction gave similar results when 50 mol% Ph_3PO was used. ^{1}H NMR (500 MHz, Chloroform-*d*) δ 7.43 – 7.19 (m, 25H, Ar-CH), 5.26 (s, 1H, PhC*H*), 5.04 (d, *J* = 12.2 Hz, 1H, $PhCH_2$), 4.89 (d, *J* = 10.8 Hz, 1H, $PhCH_2$), 4.87 (s, 2H, 2 × $PhCH_2$), 4.77 (d, *J* = 12.3 Hz, 1H, $PhCH_2$), 4.63 (s, 1H, H-1'), 4.60 (d, *J* = 10.8 Hz, 1H, $PhCH_2$), 4.57 (d, *J* = 3.8 Hz, 1H, H-1), 4.29 – 4.13 (m, 4H, 2 × $PhCH_2$, H-6a, H-3), 3.95 (d, *J* = 2.6 Hz, 1H, H-2'), 3.78 (td, *J* = 10.0, 4.8 Hz, 1H, H-5), 3.65 – 3.56 (m, 2H, H-4', H-6b), 3.53 (dd, *J* = 9.2, 3.8 Hz, 1H, H-2), 3.38 (s, 3H, OCH_3), 3.33 – 3.25 (m, 3H, H-3', H-4, H-5'), 1.37 (d, *J* = 6.2 Hz, 3H, H-6'). ^{13}C NMR (126 MHz, Chloroform-*d*) δ 139.2 (4ºC), 138.7 (4ºC), 138.5 (4ºC), 138.3 (4ºC), 137.17 (4ºC), 129.22 (Ar-*C*H), 128.4 (Ar-*C*H), 128.33 (Ar-*C*H), 128.30 (Ar-*C*H), 128.10 (Ar-*C*H), 128.09 (Ar-*C*H), 128.0 (Ar-*C*H), 127.69 (Ar-*C*H), 127.64 (Ar-*C*H), 127.59 (Ar-*C*H), 127.48 (Ar-*C*H), 127.46 (Ar-*C*H), 127.34 (Ar-*C*H), 126.0 (Ar-*C*H), 103.2 (C-1', $^1J_{1CH}$ = 157.0 Hz, from coupled HSQC), 101.66 (PhC*H*), 99.78 (C-1, $^1J_{1CH}$ = 171.0 Hz, from coupled HSQC), 83.0 (C-3'), 81.7 (C-4), 79.9 (C-4', C-3), 77.8 (C-2), 75.4 (Ph*C*H_2), 74.18 (Ph*C*H_2), 74.15 (C-2'), 73.8 (Ph*C*H_2), 72.1 (C-5'), 71.4 (Ph*C*H_2), 69.1 (C-6), 61.9 (C-5), 55.41 (O*C*H_3), 18.1 (C-6'). ESI-HRMS for $C_{48}H_{56}NO_{10}^+$ $(M+NH_4)^+$ calculated: 806.3899; found: 806.3920.

Phenyl 2,3,4-tri-*O*-benzyl-β-L-rhamnoside 5e

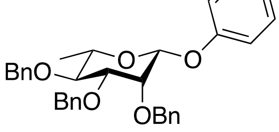

Following general procedure E, hemiacetal **1j** (44 mg, 0.1 mmol), Ph_3PO (28 mg, 0.1 mmol), $(COCl)_2$ (10 μL, 0.12 mmol), LiI (54 mg, 0.4 mmol), iPr_2NEt (44 μL, 0.25 mmol) and acceptor **3e** (0.007 mg,

0.07 mmol) were used. The reaction was stirred at rt for 20 h. ^{1}H NMR spectroscopy of the crude reaction mixture gave an α/β = 2:98. Purification by column chromatography (9:1; pentane/ Et_2O) afforded the desired product 5e as a white solid (35.7 mg, quantitative yield). R_f = 0.6 (4:1; cyclohexane/ EtOAc); Reaction gave anomeric mixture (α/β 17:83) when performed at 45 °C. ^{1}H NMR (500 MHz, Chloroform-*d*) δ 7.55 – 7.51 (m, 2H, Ar-CH), 7.38 – 7.20 (m, 15H, Ar-CH), 7.03 – 6.99 (m, 1H, Ar-CH), 6.99 – 6.96 (m, 2H, Ar-CH), 5.08 (d, *J* = 12.4 Hz, 1H, $PhCH_2$), 5.03 – 4.95 (m, 3H, 2 × $PhCH_2$, H-1), 4.70 – 4.66 (m, 1H, $PhCH_2$), 4.60 – 4.49 (m, 2H, 2 × $PhCH_2$), 4.07 (d, *J* = 2.8 Hz, 1H, H-2), 3.71 (t, *J* = 9.3 Hz, 1H, H-4), 3.55 (dd, *J* = 9.4, 3.0 Hz, 1H, H-3), 3.51 – 3.43 (m, 1H, H-5), 1.42 (d, *J* = 6.2 Hz, 3H, H-6). ^{13}C NMR (126 MHz, Chloroform-*d*) δ 157.2 (4ºC), 138.6 (4ºC), 138.4 (4ºC), 138.1 (4ºC), 129.5 (Ar-*C*H), 128.5 (Ar-*C*H), 128.4 (Ar-*C*H), 128.2 (Ar-*C*H), 128.1 (Ar-*C*H), 127.8 (Ar-*C*H), 127.7 (Ar-*C*H), 127.64 (Ar-*C*H), 127.59 (Ar-*C*H), 122.4 (Ar-*C*H), 116.2 (Ar-*C*H), 99.1 (C-1, $^1J_{1CH}$ = 156.0 Hz, from coupled HSQC), 82.0 (C-3), 79.9 (C-4), 75.5 (Ph*C*H_2), 74.34 (C-2), 74.29 (Ph*C*H_2), 72.1 (C-5), 71.7 (Ph*C*H_2), 18.1 (C-6). ESI-HRMS for $C_{33}H_{38}NO_5^+$ $(M+NH_4)^+$ calculated: 528.2744; found: 528.2745.

Naphthyl 2,3,4-tri-*O*-benzyl-β-L-rhamnoside 5f

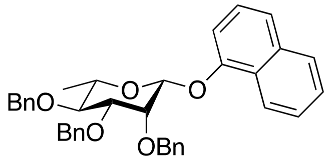

Following general procedure E, hemiacetal **1j** (44 mg, 0.1 mmol), Ph_3PO (28 mg, 0.1 mmol), $(COCl)_2$ (10 μL, 0.12 mmol), LiI (54 mg, 0.4 mmol), iPr_2NEt (44 μL, 0.25 mmol) and acceptor **3f** (10 mg, 0.07 mmol) were used. The reaction was stirred at rt for 20 h. ^{1}H NMR spectroscopy of the crude reaction mixture showed β-only product. Purification by column chromatography (4:1; pentane/ Et_2O) afforded the desired product 5f as a brown solid (40 mg, quantitative yield). ^{1}H NMR (600 MHz, Chloroform-*d*) δ 8.12 (d, *J* = 8.3 Hz, 1H, Ar-CH), 7.79 (d, *J* = 8.0 Hz, 1H, Ar-CH), 7.59 (d, *J* = 7.0 Hz, 1H, Ar-CH), 7.53 – 7.41 (m, 3H, Ar-CH), 7.39 – 7.24 (m, 15H, Ar-CH), 7.03 (d, *J* = 7.5 Hz, 1H, Ar-CH), 5.24 (d, *J* = 12.2 Hz, 1H, $PhCH_2$), 5.13 (s, 1H, H-1), 5.11 (d, *J* = 12.2 Hz, 1H, $PhCH_2$), 5.00 (d, *J* = 10.8 Hz, 1H, $PhCH_2$), 4.70 (d, *J* = 10.8 Hz, 1H, $PhCH_2$), 4.65 – 4.56 (m, 2H, 2 × $PhCH_2$), 4.25 (d, *J* = 2.7 Hz, 1H, H-2), 3.77 (t, *J* = 9.3 Hz, 1H, H-4), 3.62 (dd, *J* = 9.4, 2.9 Hz, 1H, H-3), 3.55 (dq, *J* = 9.2, 6.2 Hz, 1H, H-5), 1.46 (d, *J* = 6.2 Hz, 3H, H-6). ^{13}C NMR (151 MHz, Chloroform-*d*) δ 153.3 (4ºC), 138.7 (4ºC), 138.4 (4ºC), 138.1 (4ºC), 134.5 (4ºC), 128.5 (Ar-*C*H), 128.43 (Ar-*C*H), 128.41 (Ar-*C*H), 128.3 (Ar-*C*H), 128.1 (Ar-*C*H), 127.8 (Ar-*C*H), 127.72 (Ar-*C*H), 127.65 (Ar-*C*H), 127.57 (Ar-*C*H), 126.3 (Ar-*C*H), 125.84 (Ar-*C*H), 125.80 (Ar-*C*H), 125.5 (Ar-*C*H), 122.2 (Ar-*C*H), 122.1 (Ar-*C*H), 108.6 (Ar-*C*H), 99.7 (C-1, $^1J_{1CH}$ = 156.0 Hz, from coupled HSQC), 82.3 (C-3), 78.0 (C-4), 75.5 (Ph*C*H_2), 75.3 (C-2), 74.7 (Ph*C*H_2),

72.2 (C-5), 71.9 (Ph*C*H_2), 18.2 (C-6). ESI-HRMS for $C_{37}H_{40}NO_5^+$ $(M+NH_4)^+$ calculated: 578.2901; found: 578.2907.

***p*-Nitrophenyl 2,3,4-tri-*O*-benzyl-β-L-rhamnoside 5g**

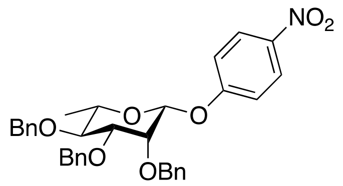

Following general procedure E, hemiacetal **1j** (44 mg, 0.1 mmol), Ph_3PO (28 mg, 0.1 mmol), $(COCl)_2$ (10 µL, 0.12 mmol), LiI (54 mg, 0.4 mmol), iPr_2NEt (44 µL, 0.25 mmol) and acceptor **3g** (9.7 mg, 0.07 mmol) were used. The reaction was stirred at 45 °C for 24 h. ^{1}H NMR spectroscopy of the crude reaction mixture gave an α/β = 4:96. Purification by column chromatography (4:1; pentane/ Et_2O) afforded the desired product 5g as a white solid (34 mg, 87% yield). R_f = 0.8 (4:1; cyclohexane/ EtOAc); ^{1}H NMR (500 MHz, Chloroform-*d*) δ 8.18 (d, J = 9.3 Hz, 2H, Ar-CH), 7.53 – 7.49 (m, 2H, Ar-CH), 7.39 – 7.26 (m, 13H, Ar-CH), 6.99 (d, J = 9.3 Hz, 2H, Ar-CH), 5.06 – 5.05 (m, 1H, H-1), 5.04 – 4.97 (m, 3H, 3 × $PhCH_2$), 4.71 – 4.67 (m, 1H, $PhCH_2$), 4.65 – 4.56 (m, 2H, 2 × $PhCH_2$), 4.09 (d, J = 2.8 Hz, 1H, H-2), 3.72 (t, J = 9.2 Hz, 1H, H-4), 3.59 (dd, J = 9.3, 2.8 Hz, 1H, H-3), 3.52 (dq, J = 9.1, 6.2 Hz, 1H, H-5), 1.42 (d, J = 6.2 Hz, 3H, H-6). ^{13}C NMR (126 MHz, Chloroform-*d*) δ 161.8 (4ºC), 142.6 (4ºC), 138.24 (4ºC), 138.21 (4ºC), 137.9 (4ºC), 128.49 (Ar-*C*H), 128.46 (Ar-*C*H), 128.45 (Ar-*C*H), 128.3 (Ar-*C*H), 128.1 (Ar-*C*H), 127.9 (Ar-*C*H), 127.83 (Ar-*C*H), 127.79 (Ar-*C*H), 127.70 (Ar-*C*H), 125.8 (Ar-*C*H), 116.1 (Ar-*C*H), 98.3 (C-1, $^1J_{1CH}$ = 156.0 Hz, from coupled HSQC), 81.8 (C-3), 79.6 (C-4), 75.6 (Ph*C*H_2), 74.6 (Ph*C*H_2), 74.3 (C-2), 72.4 (C-5), 72.1 (Ph*C*H_2), 18.0 (C-6). ESI-HRMS for $C_{33}H_{33}NO_7Na^+$ $(M+Na)^+$ calculated: 578.2149; found: 578.2149.

***p*-Methoxyphenyl 2-azido-3,6-di-*O*-benzyl-4-*O*-(2,3,4-tri-*O*-benzyl-β-L-rhamnosyl)-β-D-glucopyranoside 5h**

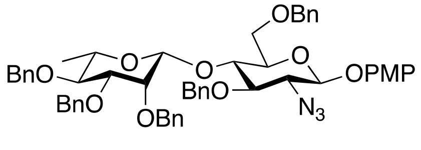

Following general procedure E, hemiacetal **1j** (44 mg, 0.1 mmol), Ph_3PO (14 mg, 0.05 mmol), $(COCl)_2$ (10 µL, 0.12 mmol), LiI (54 mg, 0.4 mmol), iPr_2NEt (70 µL, 0.4 mmol) and acceptor **3h** (24.5 mg, 0.05

mmol) were used. The reaction was stirred at 45 °C for 15 h. ^{1}H NMR spectroscopy of the crude reaction mixture showed β-only product. Purification by column chromatography (9:1; cyclohexane/Et_2O) afforded the desired product **5h** as a colourless syrup (27 mg, 60% yield). R_f = 0.5 (4:1; cyclohexane/ EtOAc); ^{1}H NMR (500 MHz, Chloroform-*d*) δ 7.40 (dd, *J* = 7.6, 1.6 Hz, 2H, Ar-CH), 7.35 – 7.21 (m, 21H, Ar-CH), 7.18 (d, *J* = 6.8 Hz, 2H, Ar-CH), 7.12 – 7.09 (m, 2H, Ar-CH), 6.82 – 6.78 (m, 2H, Ar-CH), 4.89 (d, *J* = 11.0 Hz, 2H, 2 × $PhCH_2$), 4.79 – 4.77 (m, 2H, 2 × $PhCH_2$), 4.76 (d, *J* = 8.1 Hz, 1H, H-1), 4.62 – 4.54 (m, 3H, 3 × $PhCH_2$), 4.53 (s, 1H, H-1'), 4.31 (s, 2H, 2 × $PhCH_2$), 4.26 (d, *J* = 11.5 Hz, 1H, $PhCH_2$), 4.16 – 4.10 (m, 1H, H-6a), 3.77 (s, 3H, OCH_3), 3.68 – 3.61 (m, 4H, H-2, H-4, H-5, H-6b), 3.58 (d, *J* = 2.7 Hz, 1H, H-2'), 3.52 (t, *J* = 9.4 Hz, 1H, H-4'), 3.30 – 3.24 (m, 1H, H-3), 3.23 – 3.14 (m, 2H, H-3', H-5'), 1.29 (d, *J* = 6.1 Hz, 3H, H-6'). ^{13}C NMR (126 MHz, Chloroform-*d*) δ 155.5 (4ºC), 151.3 (4ºC), 138.7 (4ºC), 138.6 (4ºC), 138.4 (4ºC), 138.3 (4ºC), 138.0 (4ºC), 128.6 (Ar-*C*H), 128.5 (Ar-*C*H), 128.4 (Ar-*C*H), 128.3 (Ar-*C*H), 128.2 (Ar-*C*H), 127.9 (Ar-*C*H), 127.8 (Ar-*C*H), 127.7 (Ar-*C*H), 127.6 (Ar-*C*H), 127.5 (Ar-*C*H), 127.4 (Ar-*C*H), 127.2 (Ar-*C*H), 101.9 (C-1', $^{1}J_{1CH}$ = 157.0 Hz, from coupled HSQC), 101.5 (C-1, $^{1}J_{1CH}$ = 167.0 Hz, from coupled HSQC), 83.4 (C-3), 82.7 (C-3'), 79.9 (C-4'), 76.3 (C-4), 75.5 (Ph*C*H_2), 75.4 (Ph*C*H_2), 75.0 (C-5), 74.0 (C-2'), 73.9 (Ph*C*H_2), 73.4 (Ph*C*H_2), 71.9 (C-5'), 71.8 (Ph*C*H_2), 69.7 (C-6), 66.0 (C-2) 55.7 (O*C*H_3), 17.9 (C-6'). ESI-HRMS for $C_{55}H_{63}N_4O_{10}$ $(M+Na)^+$ calculated: 944.4093; found: 944.2120.

Ethyl 1-thiol 2,3,4-tri-*O*-benzyl-6-*O*-(2,3,4-tri-*O*-*p*-methylbenzyl-β-L-rhamnosyl)-α-D-mannopyranoside 5i

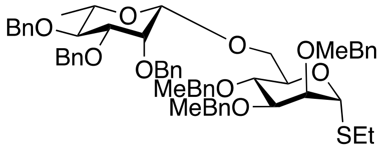

Following general procedure E, hemiacetal **1j** (44 mg, 0.1 mmol), Ph_3PO (14 mg, 0.05 mmol), $(COCl)_2$ (10 μL, 0.12 mmol), LiI (54 mg, 0.4 mmol), iPr_2NEt (44 μL, 0.25 mmol) and acceptor **3i** (38 mg, 0.07 mmol) were used. The reaction was stirred at 45 °C for 15 h. ^{1}H NMR spectroscopy of the crude reaction mixture showed β-only product. Purification by column chromatography (9:1 to 7:3; cyclohexane/Et_2O) afforded the desired product **5i** as a colourless syrup (65.6 mg, 98% yield). R_f = 0.55 (4:1; cyclohexane/ EtOAc); ^{1}H NMR (500 MHz, Chloroform-*d*) δ 7.46 – 7.42 (m, 2H, Ar-CH), 7.35 – 7.17 (m, 19H, Ar-CH), 7.14 (d, *J* =7.7 Hz, 2H, Ar-CH), 7.10 (d, *J* =7.7 Hz, 2H, Ar-CH), 7.03 (d, *J* = 7.7 Hz, 2H, Ar-CH), 5.33 (d, *J* = 1.6 Hz, 1H, H-1), 4.99 (d, *J* = 12.3 Hz, 1H, $PhCH_2$), 4.95 (d, *J* = 10.9 Hz, 1H, $PhCH_2$), 4.83 (d, *J* = 12.3 Hz, 1H, $PhCH_2$), 4.78 (d, *J* = 10.1 Hz, 1H, $PhCH_2$), 4.67 (d, *J* = 10.1 Hz, 1H, $PhCH_2$), 4.65 – 4.53 (m, 5H, 5 × $PhCH_2$), 4.52 (s, 1H, H-1'), 4.42 (d, *J* = 11.9 Hz, 1H, $PhCH_2$), 4.38 – 4.29 (m, 2H,

PhCH$_2$, H-6a), 4.14 (t, J = 9.5 Hz, 1H, H-4), 4.05 (ddd, J = 9.7, 3.6, 1.7 Hz, 1H, H-5), 3.99 (d, J = 3.0 Hz, 1H, H-2'), 3.82 (dd, J = 3.1, 1.6 Hz, 1H, H-2), 3.79 (dd, J = 9.3, 3.1 Hz, 1H, H-3), 3.68 (dd, J = 11.4, 1.8 Hz, 1H, H-6b), 3.58 (t, J = 9.3 Hz, 1H, H-4'), 3.42 (dd, J = 9.4, 3.1 Hz, 1H, H-3'), 3.32 (dq, J = 9.1, 6.1 Hz, 1H, H-5'), 2.63 – 2.44 (m, 2H, SCH_2CH$_3$), 2.35 (s, 3H, CH$_3$), 2.32 (s, 3H, CH$_3$), 2.25 (s, 3H, CH$_3$), 1.37 (d, J = 6.2 Hz, 3H, H-6'), 1.19 (t, J = 7.4 Hz, 3H, SCH$_2$CH_3). ^{13}C NMR (126 MHz, Chloroform-*d*) δ 139.0 (4ºC), 138.7 (4ºC), 138.4 (4ºC), 137.30 (4ºC), 137.28 (4ºC), 137.2 (4ºC), 135.7 (4ºC), 135.5 (4ºC), 135.2 (4ºC), 129.00 (Ar-*C*H), 128.97 (Ar-*C*H), 128.5 (Ar-*C*H), 128.40 (Ar-*C*H), 128.35 (Ar-*C*H), 128.3 (Ar-*C*H), 128.1 (Ar-*C*H), 128.0 (Ar-*C*H), 127.8 (Ar-*C*H), 127.62 (Ar-*C*H), 127.59 (Ar-*C*H), 127.50 (Ar-*C*H), 127.2 (Ar-*C*H), 101.5 (C-1', $^1J_{1CH}$ = 155.6 Hz, from coupled HSQC), 82.5 (C-1, $^1J_{1CH}$ = 164.0 Hz, from coupled HSQC), 81.9 (C-3'), 80.2 (C-4'), 80.1 (C-3), 76.6 (C-2), 75.4 (Ph*C*H$_2$), 75.2 (Ph*C*H$_2$), 74.7 (C-4), 74.13 (Ph*C*H$_2$), 74.10 (C-2'), 72.3 (Ph*C*H$_2$), 72.1 (Ph*C*H$_2$, C-5), 71.9 (C-5'), 70.9 (Ph*C*H$_2$), 67.5 (C-6), 25.4 (S*C*H$_2$CH$_3$), 21.3 (*C*H$_3$), 21.23 (*C*H$_3$), 21.16 (*C*H$_3$), 18.11 (C-6'), 15.05 (SCH$_2$*C*H$_3$). ESI-HRMS for $C_{59}H_{72}NO_9S^+$ $(M+NH_4)^+$ calculated: 970.4922; found: 970.4917.

Methyl 3-*O*-benzyl-4,6-benzylidene-2-*O*-(2,3,4-tri-*O*-benzyl-β-L-rhamnosyl)-α-D-galactopyranoside 5j

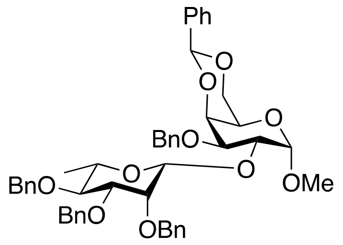

Following general procedure E, hemiacetal **1j** (44 mg, 0.1 mmol), Ph$_3$PO (14 mg, 0.05 mmol), (COCl)$_2$ (10 µL, 0.12 mmol), LiI (54 mg, 0.4 mmol), iPr$_2$NEt (70 µL, 0.4 mmol) and **3j** (26 mg, 0.07 mmol) were used. The reaction was stirred at 45 °C for 24 h. ^{1}H NMR spectroscopy of the crude reaction mixture showed β-only product. Purification by column chromatography (4:1 to 1:1; pentane/ Et$_2$O) afforded the desired product **5j** as a colourless syrup (24.1 mg, 44% yield). R_f = 0.3 (7:3; cyclohexane/ EtOAc); ^{1}H NMR (500 MHz, Chloroform-*d*) δ 7.56 – 7.51 (m, 2H, Ar-CH), 7.45 – 7.19 (m, 23H, Ar-CH), 5.45 (s, 1H, PhCH), 4.99 (d, J = 12.3 Hz, 1H, PhCH$_2$), 4.94 – 4.91 (m, 2H, H-1, PhCH$_2$), 4.90 – 4.84 (m, 2H, 2 × PhCH$_2$), 4.71 (d, J = 12.3 Hz, 1H, PhCH$_2$), 4.64 – 4.60 (m, 2H, PhCH$_2$, H-1), 4.53 (d, J = 11.8 Hz, 1H, PhCH$_2$), 4.47 (d, J = 11.8 Hz, 1H, PhCH$_2$), 4.42 (dd, J = 10.1, 3.5 Hz, 1H, H-2), 4.23 (dd, J = 12.5, 1.6 Hz, 1H, H-6a), 4.14 (dd, J = 3.7, 1.2 Hz, 1H, H-4), 4.01 (dd, J = 12.5, 1.8 Hz, 1H, H-6b), 3.97 (dd, J = 10.1, 3.6 Hz, 1H, H-3), 3.92 (d, J = 3.0 Hz, 1H, H-2'), 3.63 – 3.58 (m, 2H, H-5, H-4'), 3.46 (dd, J = 9.4, 2.9 Hz, 1H, H-3'), 3.39 (s, 3H, OCH$_3$), 3.27 (dq, J = 9.2, 6.1 Hz, 1H, H-5'), 1.35 (d, J = 6.1 Hz, 3H,

H-6’). ^{13}C NMR (126 MHz, Chloroform-*d*) δ 139.1 (4ºC), 139.0 (4ºC), 138.6 (4ºC), 138.4 (4ºC), 137.8 (4ºC), 128.9 (Ar-*C*H), 128.33 (Ar-*C*H), 128.32 (Ar-*C*H), 128.2 (Ar-*C*H), 128.11 (Ar-*C*H), 128.08 (Ar-*C*H), 128.07 (Ar-*C*H), 128.06 (Ar-*C*H), 127.7 (Ar-*C*H), 127.6 (Ar-*C*H), 127.51 (Ar-*C*H), 127.45 (Ar-*C*H), 127.2 (Ar-*C*H), 126.3 (Ar-*C*H), 101.0 (Ph*C*H), 99.5 (C-1’, $^{1}J_{1CH}$ = 154.0 Hz, from coupled HSQC), 98.7 (C-1, $^{1}J_{1CH}$ = 170.0 Hz, from coupled HSQC), 82.3 (C-3’), 80.1 (C-4’), 75.4 (Ph*C*H$_2$), 75.0 (C-3), 74.9 (C-4), 74.8 (C-2’), 74.1 (Ph*C*H$_2$), 73.0 (C-2), 72.2 (C-5’), 72.1 (Ph*C*H$_2$), 71.4 (Ph*C*H$_2$), 69.3 (C-6), 62.6 (C-5), 55.4 (O*C*H$_3$), 18.0 (C-6’). ESI-HRMS for $C_{48}H_{56}NO_{10}^+$ (M+NH$_4$)$^+$ calculated: 806.3899; found: 806.3900.

Boc-L-tyrosine methyl ester 2,3,4-tri-*O*-benzyl-β-L-rhamnoside 5k

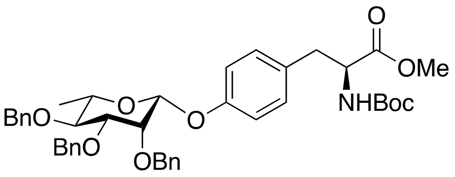

Following general procedure E**,** hemiacetal **1j** (44 mg, 0.1 mmol), Ph$_3$PO (28 mg, 0.1 mmol), (COCl)$_2$ (10 μL, 0.12 mmol), LiI (54 mg, 0.4 mmol), iPr$_2$NEt (70 μL, 0.4 mmol) and acceptor **3k** (24 mg, 0.07 mmol) were used. The reaction was stirred at 45 °C for 24 h. ^{1}H NMR spectroscopy of the crude reaction mixture showed β-only product. Purification by column chromatography (9:1 to 7:3; pentane/ Et$_2$O) afforded the desired product **5k** as a white solid (30 mg, 60% yield) along with hemiacetal **1j** (15%). R_f = 0.6 (4:1; cyclohexane/ EtOAc); ^{1}H NMR (500 MHz, Chloroform-*d*) δ 7.52 (d, *J* = 6.9 Hz, 2H, Ar-CH), 7.39 – 7.26 (m, 13H, Ar-CH), 7.02 (d, *J* = 8.5 Hz, 2H, Ar-CH), 6.89 (d, *J* = 8.6 Hz, 2H, Ar-CH), 5.06 (d, *J* = 12.4 Hz, 1H, PhCH$_2$), 4.98 (d, *J* = 11.2 Hz, 2H, 2 × PhCH$_2$), 4.93 (s, 1H, H-1), 4.67 (d, *J* = 10.8 Hz, 1H, PhCH$_2$), 4.61 – 4.49 (m, 3H, CH, 2 × PhCH$_2$), 4.05 (d, *J* = 2.7 Hz, 1H, H-2), 3.68 (br s, 4H, H-4, OCH$_3$), 3.54 (dd, *J* = 9.4, 2.9 Hz, 1H, H-3), 3.52 – 3.42 (m, 1H, H-5), 3.09 – 2.96 (m, 2H, PhCH$_2$), 1.44 – 1.38 (m, 12H, H-6, C(CH$_3$)$_3$). ^{13}C NMR (101 MHz, Chloroform-*d*) δ 172.3 (C=O), 156.3 (4ºC), 138.5 (4ºC), 138.4 (4ºC), 138.0 (4ºC), 130.3 (Ar-*C*H), 129.53 (4ºC), 128.46 (Ar-*C*H), 128.4 (Ar-*C*H), 128.3 (Ar-*C*H), 128.2 (Ar-*C*H), 128.1 (Ar-*C*H), 127.6 (Ar-*C*H), 116.3 (Ar-*C*H), 99.1 (C-1, $^{1}J_{1CH}$ = 154.9 Hz, from coupled HSQC), 82.0 (C-3), 80.0 (*C*(CH$_3$)$_3$), 79.9 (C-4), 75.5 (PhCH$_2$), 74.32 (C-2), 74.26 (PhCH$_2$), 72.1 (C-5), 71.7 (PhCH$_2$), 54.5 (*C*H), 52.2 (O*C*H$_3$), 37.5 (*C*H$_2$), 28.29 (C(*C*H$_3$)$_3$)), 18.06 (C-6). ESI-HRMS for $C_{42}H_{53}N_2O_9^+$ (M+NH$_4$)$^+$ calculated: 729.3746; found: 729.3749.

Cholesteryl 2,3,4-tri-*O*-benzyl-α/β-L-rhamnoside S50

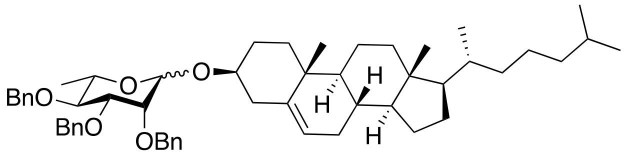

Following general procedure E, hemiacetal **1j** (44 mg, 0.1 mmol), Ph_3PO (28 mg, 0.1 mmol), $(COCl)_2$ (10 µL, 0.12 mmol), LiI (54 mg, 0.4 mmol), iPr_2NEt (44 µL, 0.25 mmol) and cholesterol (27 mg, 0.07 mmol) were used. The reaction was stirred at rt for 24 h. ^{1}H NMR spectroscopy of the crude reaction mixture gave an α/β = 1:1. Purification by column chromatography (9:1; pentane/ Et_2O) afforded the desired product S50 as a white solid (54 mg, quantitative yield). R_f = 0.8 (4:1; cyclohexane/ EtOAc); Reaction gave same anomeric mixture when performed at rt and when general procedure B was used. Signals observed for both anomers:

^{1}H NMR (500 MHz, Chloroform-*d*) δ 7.50 – 7.46 (m, 2H, Ar-CH), 7.40 – 7.24 (m, 28H, Ar-CH), 5.01 – 4.87 (m, 3H, 3 × $PhCH_2$), 4.78 (d, *J* = 12.3 Hz, 1H, $PhCH_2$), 4.73 – 4.69 (m, 1H, $PhCH_2$), 4.67 – 4.61 (m, 5H, 5 × $PhCH_2$), 4.53 – 4.40 (m, 2H, 2 × $PhCH_2$), 3.61 (td, *J* = 9.3, 2.0 Hz, 2H, H-4α, H-4β), 3.54 (dddd, *J* = 15.8, 11.2, 8.3, 3.4 Hz, 1H), 3.39 (ddd, *J* = 15.4, 7.7, 3.2 Hz, 1H), 2.53 – 2.43 (m, 1H), 2.42 – 2.32 (m, 1H), 2.20 (ddd, *J* = 13.1, 4.7, 1.9 Hz, 2H), 2.13 – 1.91 (m, 7H), 1.90 – 1.77 (m, 7H), 1.63 – 1.42 (m, 22H), 1.40 – 1.29 (m, 5H), 1.29 – 1.20 (m, 4H), 1.19 – 1.04 (m, 17H), 1.03 (s, 3H), 1.00 (dd, *J* = 7.1, 3.6 Hz, 4H), 0.97 (s, 3H), 0.93 – 0.89 (m, 8H), 0.88 – 0.84 (m, 11H), 0.69 – 0.66 (m, 5H). ^{13}C NMR (126 MHz, Chloroform-*d*) δ 140.9 (4°C), 140.5 (4°C), 138.9 (4°C), 138.7 (4°C), 138.63 (4°C), 138.57 (4°C), 138.4 (4°C), 138.3 (4°C), 128.6 (Ar-*C*H), 128.37 (Ar-*C*H), 128.35 (Ar-*C*H), 128.33 (Ar-*C*H), 128.31 (Ar-*C*H), 128.12 (Ar-*C*H), 128.09 (Ar-*C*H), 128.0 (Ar-*C*H), 127.95 (Ar-*C*H), 127.64 (Ar-*C*H), 127.56 (Ar-*C*H), 127.54 (Ar-*C*H), 127.50 (Ar-*C*H), 127.47 (Ar-*C*H), 127.3 (Ar-*C*H), 78.3, 76.4, 75.4 ($PhCH_2$), 73.8 ($PhCH_2$), 72.8 ($PhCH_2$), 72.1 ($PhCH_2$), 71.3 ($PhCH_2$), 56.8, 56.7, 56.16, 56.15, 50.2, 50.1, 42.34, 42.32, 40.2, 39.81, 39.77, 39.5, 38.4, 37.3, 37.1, 36.74, 36.72, 36.2, 35.8, 31.95, 31.90, 31.89, 29.4, 28.2, 28.1, 28.0, 24.3, 23.8, 22.8, 22.6, 21.10, 21.05, 19.42, 19.36, 18.74, 18.73, 11.9. ESI-HRMS for $C_{54}H_{75}O_5^+$ $(M+H)^+$ calculated: 803.5609; found: 803.5616.

α-anomer:

^{1}H NMR (500 MHz, Chloroform-*d*) δ 5.33 – 5.29 (m, 1H, C=C*H*), 4.88 (d, *J* = 1.6 Hz, 1H, H-1), 3.88 (dd, *J* = 9.4, 3.1 Hz, 1H, H-3), 3.79 – 3.72 (m, 2H, H-5, H-2), 1.31 (d, *J* = 6.2 Hz, 3H, H-6). ^{13}C NMR (126 MHz, Chloroform-*d*) δ 121.8 (C=*C*H), 96.0 (C-1, $^1J_{1CH}$ = 169.0 Hz, from coupled HSQC), 80.8 (C-4), 80.3 (C-3), 75.5 (C-2), 68.0 (C-5), 18.0 (C-6).

β-anomer:

^{1}H NMR (500 MHz, Chloroform-*d*) δ 5.39 – 5.33 (m, 1H, C=C*H*), 4.47 (s, 1H, H-1), 3.84 (d, *J* = 3.0 Hz, 1H, H-2), 3.44 (dd, *J* = 9.4, 3.1 Hz, 1H, H-3), 3.29 (dq, *J* = 9.2, 6.1 Hz, 1H, H-5), 1.36 (d, *J* = 6.1

Hz, 3H, H-6). ^{13}C NMR (126 MHz, Chloroform-*d*) δ 121.8 (C=*C*H), 99.3 (C-1, $^{1}J_{1CH}$ = 152.0 Hz, from coupled HSQC), 82.4 (C-3), 80.2 (C-4), 74.3 (C-2), 71.8 (C-5), 18.1 (C-6).

Donor and Acceptor Limitations

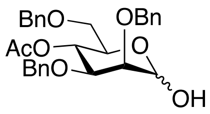

S22

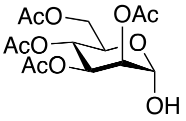

S24

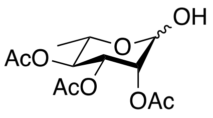

S35

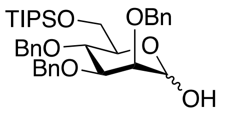

S23

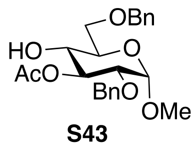

S43

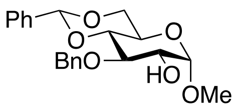

S40

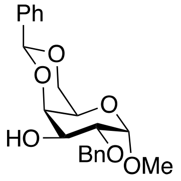

S46

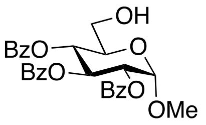

S49

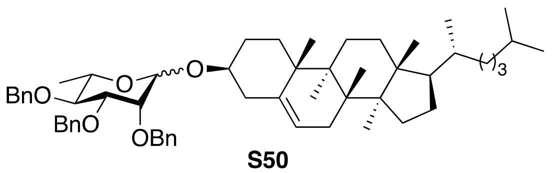

S50
quant
α/β 1:1

Peracetylated/4-OAc donors **S22**, **S24** and **S35**, were disarmed and no glycosylation was observed. 6-OTIPS donor **S23** led to the anhydro sugar. Benzylidene acceptors **S40** and **S46** were poor nucleophiles and no desired reaction was observed. Benzoylated acceptor **S49** led to complex mixtures due to ester migration. We confirmed that ester migration occurred on benzoylated acceptor **S49** with iPr_2NEt in $CHCl_3$ at 45 °C in the absence of other reagents. Acceptor **S43** gave a complex mixture in reactions with **1a**; there were trace amounts of the desired β-product (as evidenced by HSQC), and the major product was the donor elimination product. We suspect there was also acyl migration but it was difficult to be sure because of the complex mixture generated. **S43** is a poor nucleophile and so observation of elimination is not that surprising. Reaction with cholesterol gave the product **S50** in a very high yield but with no selectivity. When **S35** used as donor in the glycosylation reaction with acceptor **3a**, transesterification was observed giving product **S51** (see below).[38]

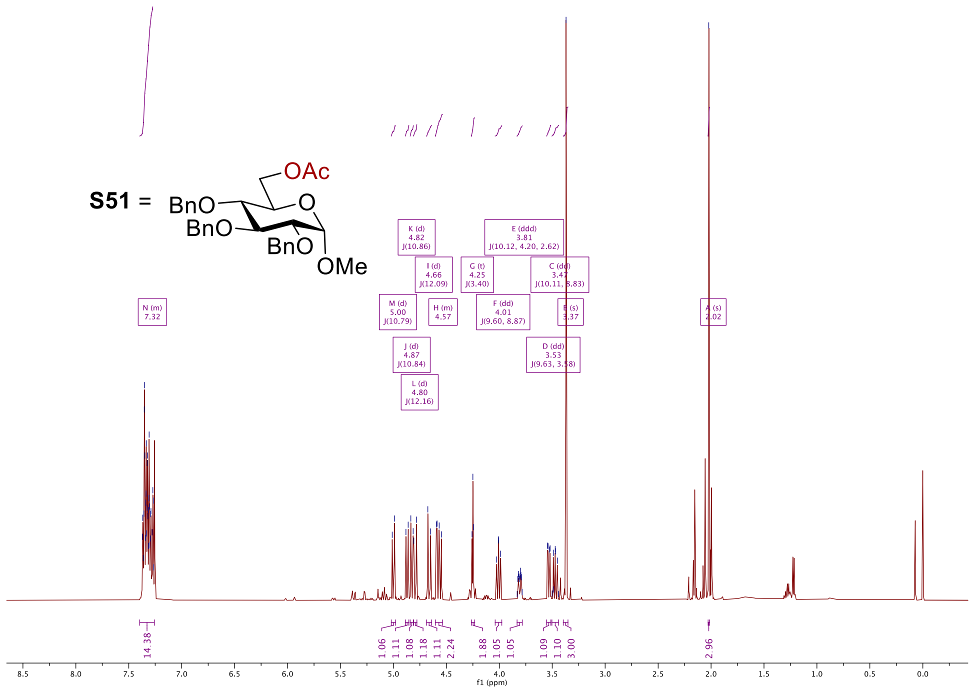

Mechanistic Investigations

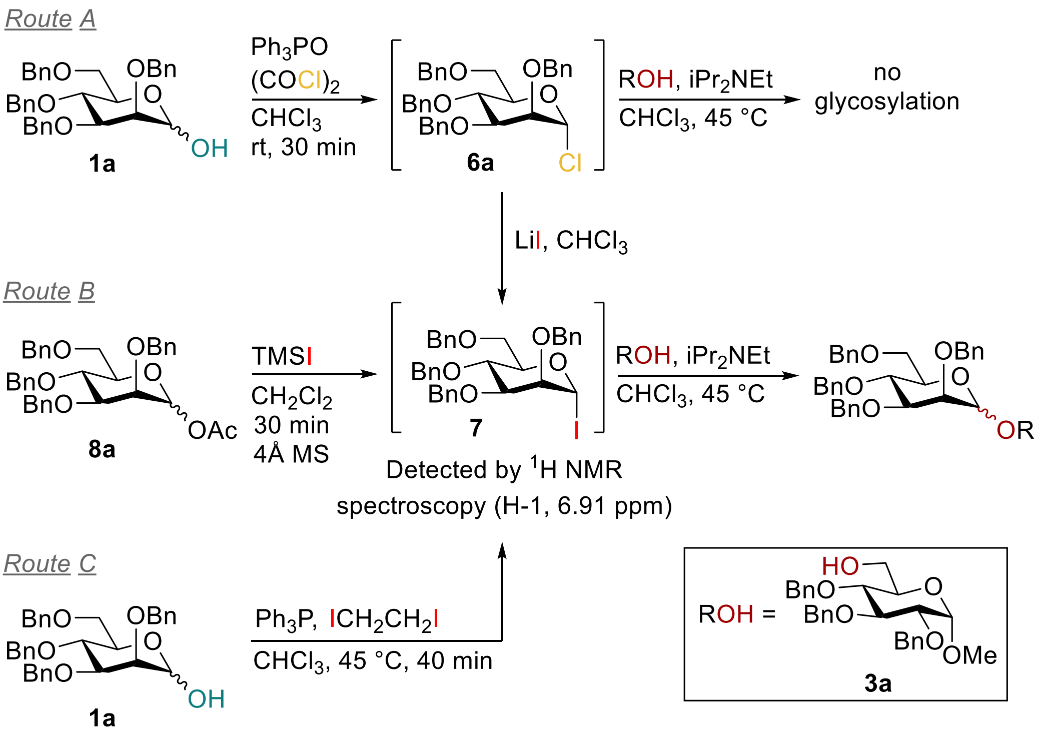

Entry	Route	Deviation from standard procedure	β/α[a]
1	A	No Ph_3PO[b]	≥20:1
2	A	0.5 eq Ph_3PO	≥20:1
3	A	No LiI	No glycosylation
4	A	NaI *in lieu* of LiI	1:1
5	A	Filtration of insoluble LiCl/LiI salts	1:1
6	A	Filtration of insoluble LiCl/LiI salts; no Ph_3PO[b]	≥20:1
7	A	2 eq LiI in lieu of 4 eq LiI	2:3
8	A	2 eq Ph_3PO	1:1
9	A	2 eq Ph_3PO, 8 eq LiI	10:1
10	A	No Ph_3PO,[b] 4Å MS	≥20:1
11	B	0.5 eq Ph_3PO	2:3
12	B	4 eq LiI	3:1
13	B	4 eq LiCl	2:3
14	C	-	2:3
15	C	4 eq LiI	≥20:1

[a] Determined by ^{1}H NMR spectroscopy of the reaction mixture.[b] After synthesis of **6a**, the Ph_3PO was removed by chromatography.

Route A:

Following the general procedure A, hemiacetal **1a** (378 mg, 0.700 mmol), Ph_3PO (195 mg, 0.500 mmol) and oxalyl chloride (64 µL, 0.77 mmol) were used. After 30 minutes, the solvent and excess oxalyl chloride were removed by applying vacuum. (For removal of Ph_3PO: The crude mannosyl chloride was subjected to flash column chromatography (R_f = 0.9, 96:4; CH_2Cl_2/Et_2O) to obtain the isolated mannosyl chloride **6a** as a yellowish syrup. A stock solution of **6a** in anhydrous $CHCl_3$ (0.4 M) was prepared).

Entry 1: Acceptor (0.7 eq), powdered LiI (4 eq) and iPr_2NEt (2.5 eq) were used (reaction was also performed for 5 h).

Entry 2: Ph_3PO (0.5 eq), acceptor (0.7 eq), powdered LiI (4 eq) and iPr_2NEt (2.5 eq) were used.

Entry 3: Ph_3PO (1 eq), acceptor (0.7 eq) and iPr_2NEt (2.5 eq) were used.

Entry 4: Ph_3PO (1 eq), acceptor (0.7 eq), NaI (4 eq) and iPr_2NEt (2.5 eq) were used (reaction gave same glycosylation outcome in both $CHCl_3$ and MeCN).

Entry 5: Ph_3PO (1 eq), powdered LiI (4 eq) and iPr_2NEt (2.5 eq) were used and the reaction left to stir for 3 h (glycosyl iodide formation); syringe filtration was carried out and then acceptor (0.7 eq) added.

Entry 6: Isolated mannosyl chloride **6a** (1 eq), powdered LiI (4 eq) and iPr_2NEt (2.5 eq) were used and the reaction left to stir for 3 h (glycosyl iodide formation); syringe filtration was carried out and then acceptor (0.7 eq) added.

Entry 7: Acceptor (0.7 eq), powdered LiI (2 eq), iPr_2NEt (2.5 eq) and were used (reaction was carried out with and without Ph_3PO, leading to similar results in both cases).
Entry 8: Ph_3PO (2 eq), acceptor (0.7 eq), LiI (4 eq) and iPr_2NEt (2.5 eq) were used.
Entry 9: Ph_3PO (2 eq), acceptor (0.7 eq), LiI (8 eq) and iPr_2NEt (2.5 eq) were used.
Entry 10: Acceptor (0.7 eq), powdered LiI (4 eq), iPr_2NEt (2.5 eq) and 4Å MS (30 mg) were used.
Route B:
In a solution of glycosyl acetate **8a** (57 mg, 0.1 mmol) in anhydrous CH_2Cl_2 (500 µL), freshly activated 4Å MS (59 mg) were added followed by the addition of TMSI (17 µL, 0.12 mmol). After 30 minutes, the solvent and excess reagent were removed by applying vacuum. The residue was redissolved in anhydrous $CHCl_3$ (250 µL).
Entry 11: Ph_3PO (0.5 eq), acceptor (0.7 eq) and iPr_2NEt (2.5 eq) were used.
Entry 12: Acceptor (0.7 eq), powdered LiI (4 eq) and iPr_2NEt (2.5 eq) were used.
Entry 13: Acceptor (0.7 eq), powdered LiCl (4 eq) and iPr_2NEt (2.5 eq) were used.
Route C:
Based on a modified literature procedure,[39] hemiacetal **1a** (54 mg, 0.10 mmol), Ph_3P (26 mg, 0.10 mmol) and 1,2-diiodoethane (28 mg, 0.10 mmol) were dissolved in anhydrous $CHCl_3$ (300 µL) and stirred at 45 °C for 40 minutes. Solvent was removed by applying vacuum and the residue was redissolved in anhydrous $CHCl_3$ (250 µL).
Entry 14: Acceptor (0.7 eq) and iPr_2NEt (2.5 eq) were used.
Entry 15: Acceptor (0.7 eq), powdered LiI (4 eq) and iPr_2NEt (2.5 eq) were used.

The reactions were stirred at 45 °C for 24 h. The α/β ratio was determined by ^1H NMR spectroscopy of the crude reaction mixture.

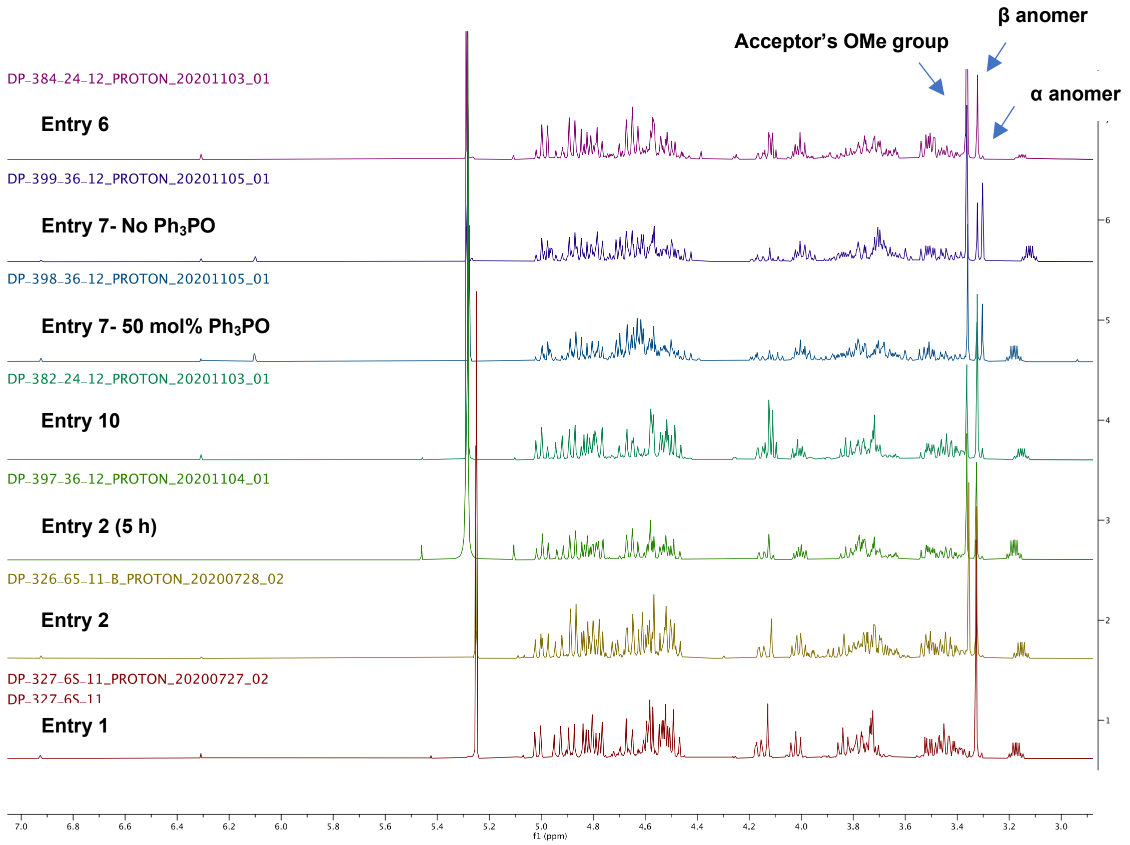

Figure S1 Stacked ^{1}H NMR spectra ($CDCl_3$, 500 MHz) of experiments to probe mechanism.

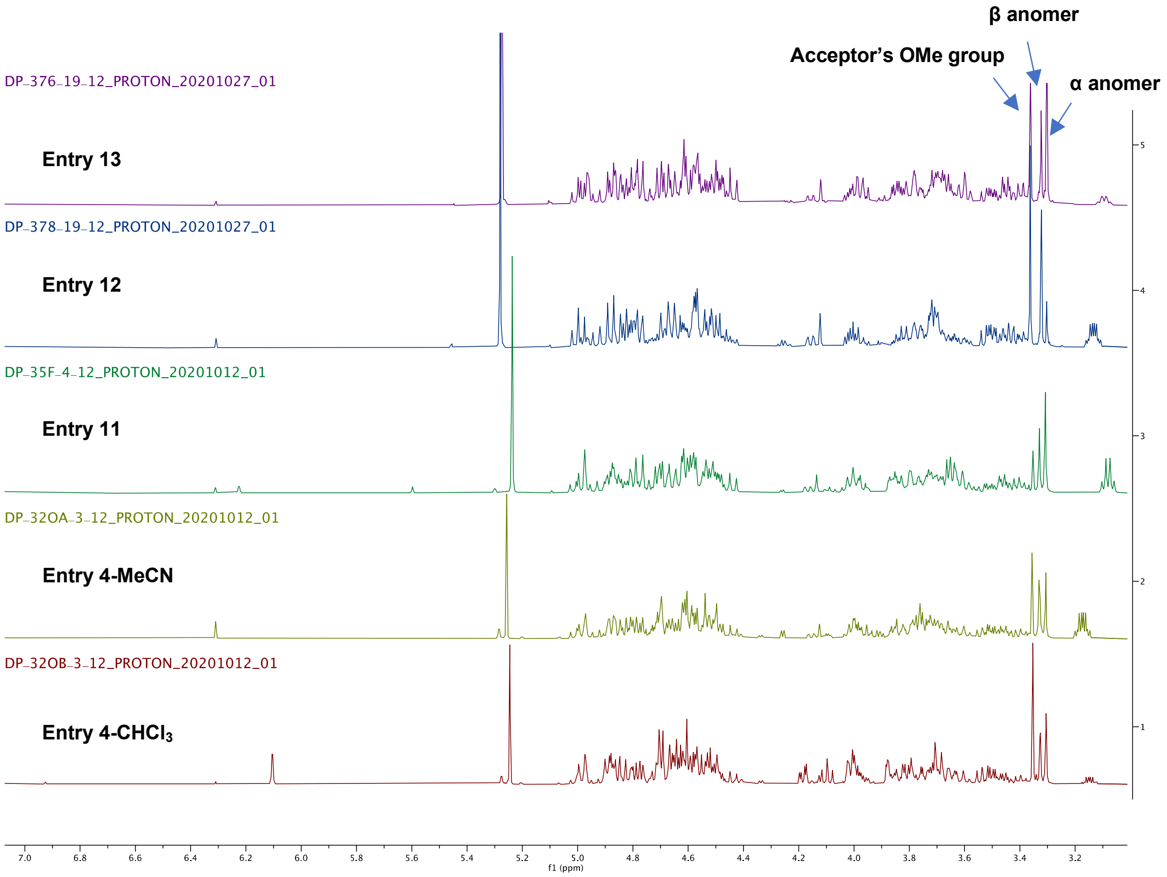

Figure S2 Stacked ^{1}H NMR spectra ($CDCl_3$, 500 MHz) of experiments to probe mechanism.

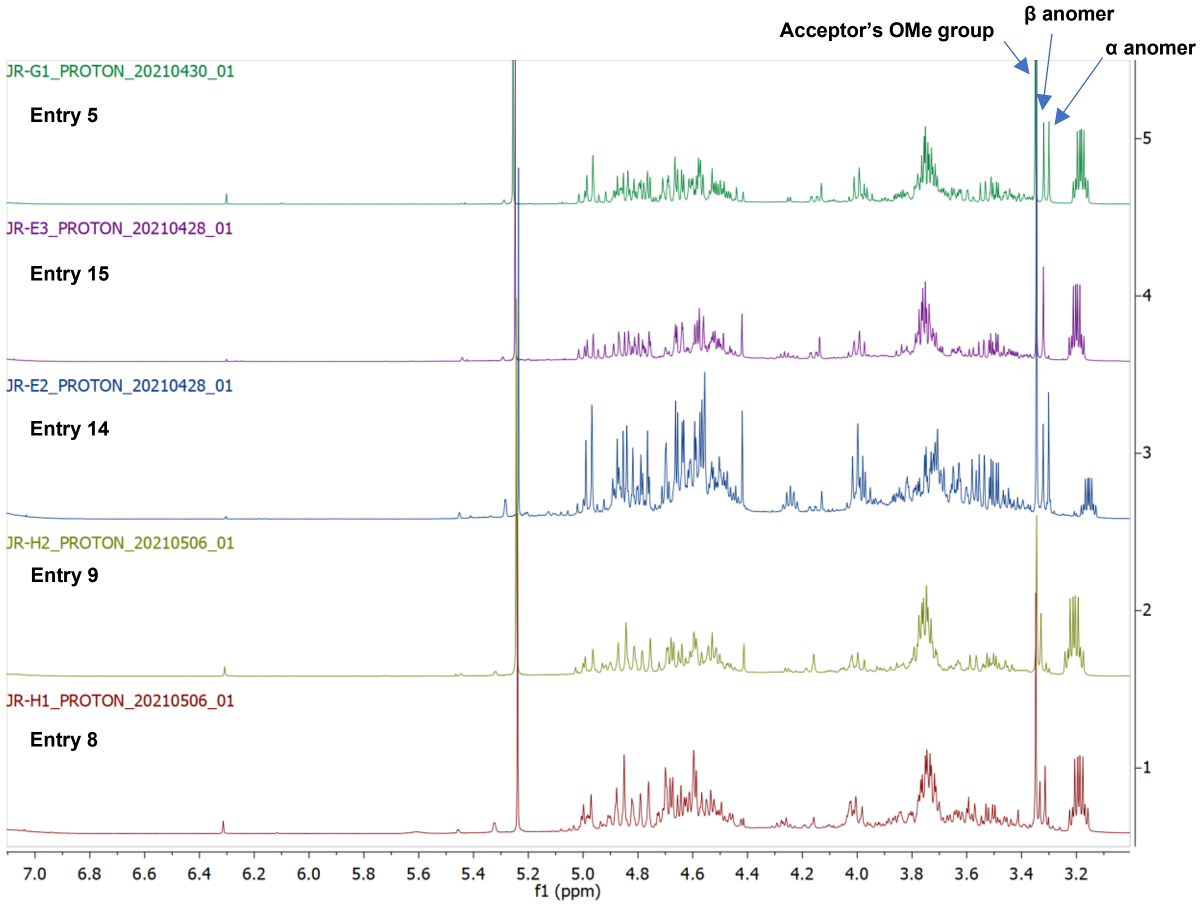

Figure S3 Stacked ^{1}H NMR spectra ($CDCl_3$, 500 MHz) of experiments to probe mechanism.

References

[1] A. B. Pangborn, M. A. Giardello, R. H. Grubbs, R. K. Rosen, F. J. Timmers, *Organometallics* **1996**, *15*, 1518–1520.

[2] M. A. Hollas, S. J. Webb, S. L. Flitsch, A. J. Fielding, *Angew. Chem. Int. Ed.* **2017**, *56*, 9449–9453.

[3] O. Norberg, B. Wu, N. Thota, J. T. Ge, G. Fauquet, A. K. Saur, T. Aastrup, H. Dong, M. Yan, O. Ramström, *Carbohydr. Res.* **2017**, *452*, 35–42.

[4] P. J. Garegg, I. Kvarnström, A. Niklasson, S. C. T. Svensson, *J. Carbohydr. Chem.* **1993**, *12*, 933–953.

[5] L. Van Huy, C. Tanaka, T. Imai, S. Yamasaki, T. Miyamoto, *ACS Med. Chem. Lett.* **2019**, *10*, 44–49.

[6] M. P. DeNinno, J. B. Etienne, K. C. Duplantier, *Tetrahedron Lett.* **1995**, *36*, 669–672.

[7] Y. Asahina, T. Kawakami, H. Hojo, *Eur. J. Org. Chem.* **2019**, 1915–1920.

[8] E. I. Balmond, D. M. Coe, M. C. Galan, E. M. McGarrigle, *Angew. Chem. Int. Ed.* **2012**, *51*, 9152–9155.

[9] G. Lemanski, T. Ziegler, *Helv. Chim. Acta* **2000**, *83*, 2655–2675.

[10] T. Shirahata, A. Kojima, S. Teruya, J. I. Matsuo, M. Yokoyama, S. Unagiike, T. Sunazuka, K. Makino, E. Kaji, S. Omura, Y. Kobayashi, *Tetrahedron* **2011**, *67*, 6482–6496.

[11] J. López-Prados, F. Cuevas, N. C. Reichardt, J. L. De Paz, E. Q. Morales, M. Martín-Lomas, *Org. Biomol. Chem.* **2005**, *3*, 764–786.

[12] J. Y. Baek, B. Y. Lee, M. G. Jo, K. S. Kim, *J. Am. Chem. Soc.* **2009**, *131*, 17705–17713.

[13] S. Koto, N. Morishima, K. Takenaka, K. Kanemitsu, N. Shimoura, M. Kase, S. Kojiro, T. Nakamura, T. Kawase, S. Zeu, *Bull. Chem. Soc. Jpn.* **1989**, *62*, 3549–3566.

[14] G. Wei, L. Zhang, C. Cai, S. Cheng, Y. Du, *Tetrahedron Lett.* **2008**, *49*, 5488–5491.

[15] Q. Wang, G. Liu, S. Gong, Q. Sun, *Adv. Mater. Res.* **2014**, *830*, 155–158.

[16] A. Subratti, N. K. Jalsa, *Tetrahedron Lett.* **2018**, *59*, 2082–2085.

[17] M. Seepersaud, S. Seecharan, L. J. Lalgee, N. K. Jalsa, *Synth. Commun.* **2017**, *47*, 853–871.

[18] E. Lee, N. Gavin, P. Mcardle, D. Cunningham, *Carbohydr. Res.* **1990**, *197*, 270–275.

[19] J. Bourke, C. F. Brereton, S. V. Gordon, E. C. Lavelle, E. M. Scanlan, *Org. Biomol. Chem.* **2014**, *12*, 1114–1123.

[20] D. A. Leigh, J. P. Smart, A. M. Truscello, *Carbohydr. Res.* **1995**, *276*, 417–424.

[21] S. Dasgupta, B. Roy, B. Mukhopadhyay, *Carbohydr. Res.* **2006**, *341*, 2708–2713.

[22] Y. Zhu, Z. Shen, W. Li, B. Yu, *Org. Biomol. Chem.* **2016**, *14*, 1536–1539.

[23] Y. Liu, T. Song, W. Meng, Y. Xu, P. G. Wang, W. Zhao, *Tetrahedron Lett.* **2016**, *57*, 2758–2762.

[24] S. Boonyarattanakalin, X. Liu, M. Michieletti, B. Lepenies, P. H. Seeberger, *J. Am. Chem.*

Soc. **2008**, *130*, 16791–16799.

[25] M. Matwiejuk, J. Thiem, *Chem. Commun.* **2011**, *47*, 8379–8381.

[26] H. Paulsen, J. P. Lorentzen, *Carbohydr. Res.* **1985**, *140*, 155–162.

[27] A. Français, D. Urban, J.-M. Beau, *Angew. Chem. Int. Ed.* **2007**, *46*, 8662–8665.

[28] C. C. Wang, J. C. Lee, S. Y. Luo, S. S. Kulkarni, Y. W. Huang, C. C. Lee, K. L. Chang, S. C. Hung, *Nature* **2007**, *446*, 896–899.

[29] S. C. Ranade, S. Kaeothip, A. V. Demchenko, *Org. Lett.* **2010**, *12*, 5628–5631.

[30] E. Durantie, C. Bucher, R. Gilmour, *Chem. Eur. J.* **2012**, *18*, 8208–8215.

[31] Z. Wang, *Comprehensive Organic Name Reactions and Reagents*, John Wiley & Sons, Ltd, Hoboken, **2010**.

[32] F. Dasgupta, P. J. Garegg, *Synthesis* **1994**, 1121–1123.

[33] J. Dinkelaar, A. R. De Jong, R. Van Meer, M. Somers, G. Lodder, H. S. Overkleeft, J. D. C. Codée, G. A. Van Der Marel, *J. Org. Chem.* **2009**, *74*, 4982–4991.

[34] L. Meng, P. Wu, J. Fang, Y. Xiao, X. Xiao, G. Tu, X. Ma, S. Teng, J. Zeng, Q. Wan, *J. Am. Chem. Soc.* **2019**, *141*, 11775–11780.

[35] P. Wen, D. Crich, *Org. Lett.* **2017**, *19*, 2402–2405.

[36] F. Dasgupta, P. J. Garegg, *Carbohydr. Res.* **1990**, *202*, 225–238.

[37] V. Dimakos, J. J. W. Liu, Z. Ge, M. S. Taylor, *Org. Biomol. Chem.* **2019**, *17*, 5671–5674.

[38] A. Gurawa, M. Kumar, D. S. Rao, S. Kashyap, *New J. Chem.* **2020**, *44*, 16702–16707.

[39] J. Chen, J. H. Lin, J. C. Xiao, *Org. Lett.* **2018**, *20*, 3061–3064.
