# Supplementary material for: Stereoselective β-mannosylations and β-rhamnosylations from glycosyl hemiacetals mediated by lithium iodide
Source: Chem Sci. 2021 Jul 5;12(29):10070–5. doi: 10.1039/d1sc01300a (PMC8317664; doi:10.1039/d1sc01300a)

Electronic Supplementary Material (ESI) for Chemical Science.
This journal is © The Royal Society of Chemistry 2021

Stereoselective β-Mannosylations and β-Rhamnosylations from Glycosyl Hemiacetals Mediated by Lithium Iodide

Imlirenla Pongener, Dionissia A. Pepe, Joseph J. Ruddy and Eoghan M. McGarrigle*

Centre for Synthesis & Chemical Biology, UCD School of Chemistry, University College Dublin, Belfield, Dublin 4, Ireland.

Supporting Information – Part 2
NMR Spectra

NMR Spectra

^{1}H NMR (500 MHz, Chloroform-*d*) S1

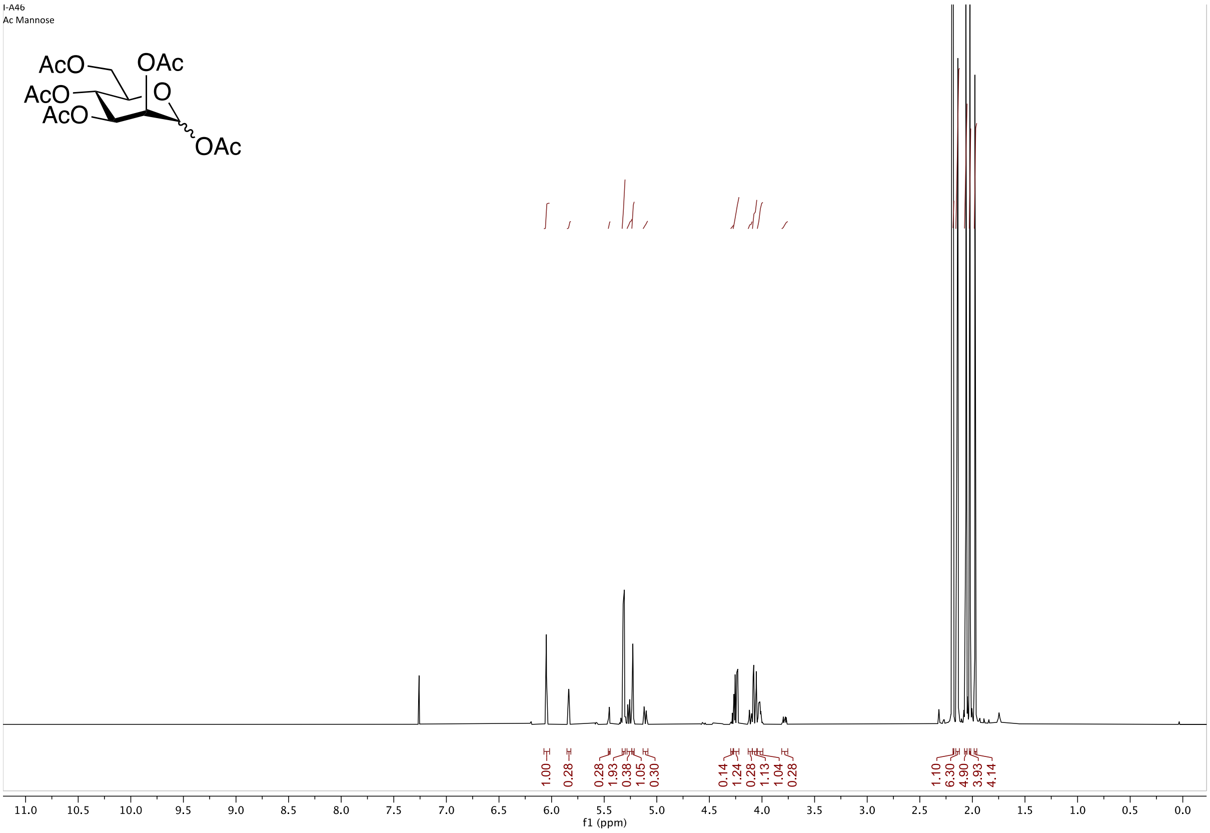

^{1}H NMR (500 MHz, Chloroform-*d*) S2

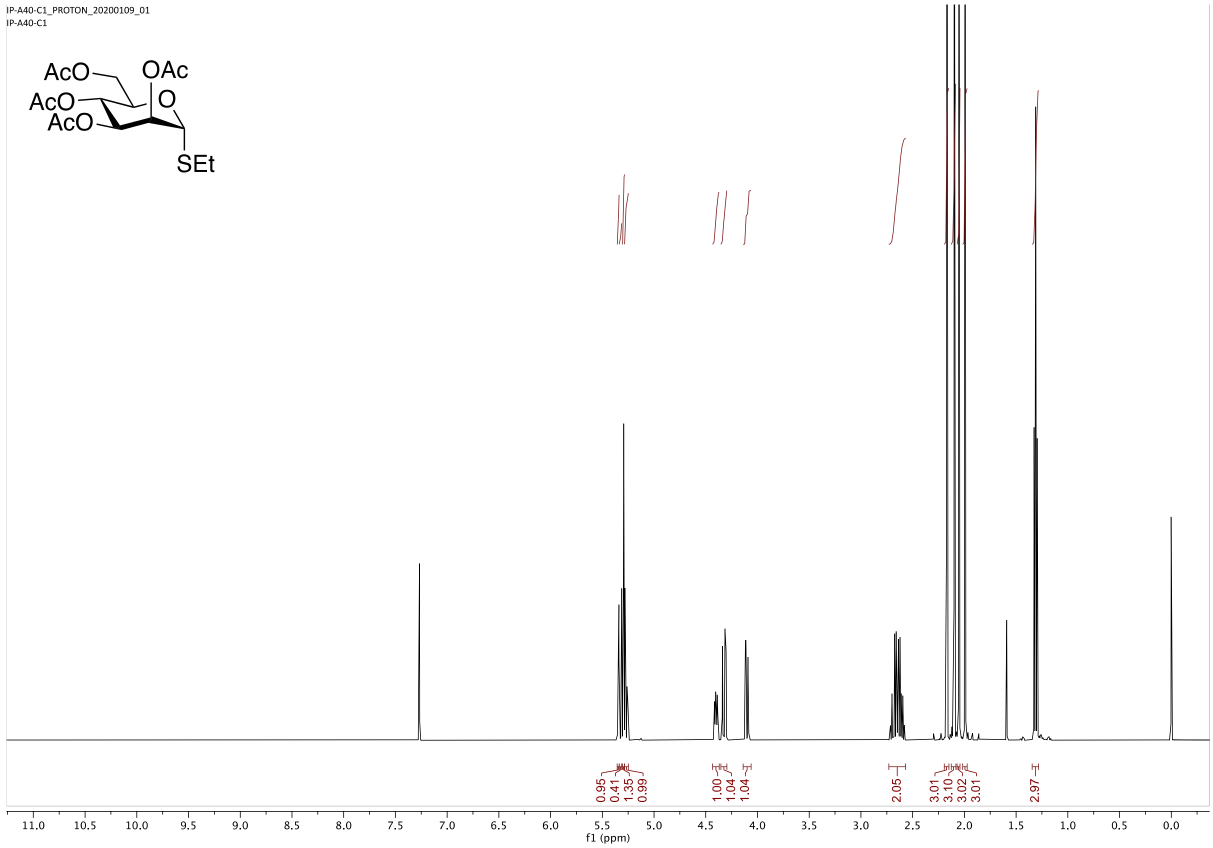
IP-A40-C1_PROTON_20200109_01
IP-A40-C1
AcO
OAc
AcO
O
AcO
SEt
0.95
0.41
1.35
0.99
1.00
1.04
1.04
2.05
3.01
3.10
3.02
3.01
2.97
11.0
10.5
10.0
9.5
9.0
8.5
8.0
7.5
7.0
6.5
6.0
5.5
5.0
4.5
4.0
3.5
3.0
2.5
2.0
1.5
1.0
0.5
0.0
f1 (ppm)

^{13}C NMR (126 MHz, Chloroform-*d*) S2

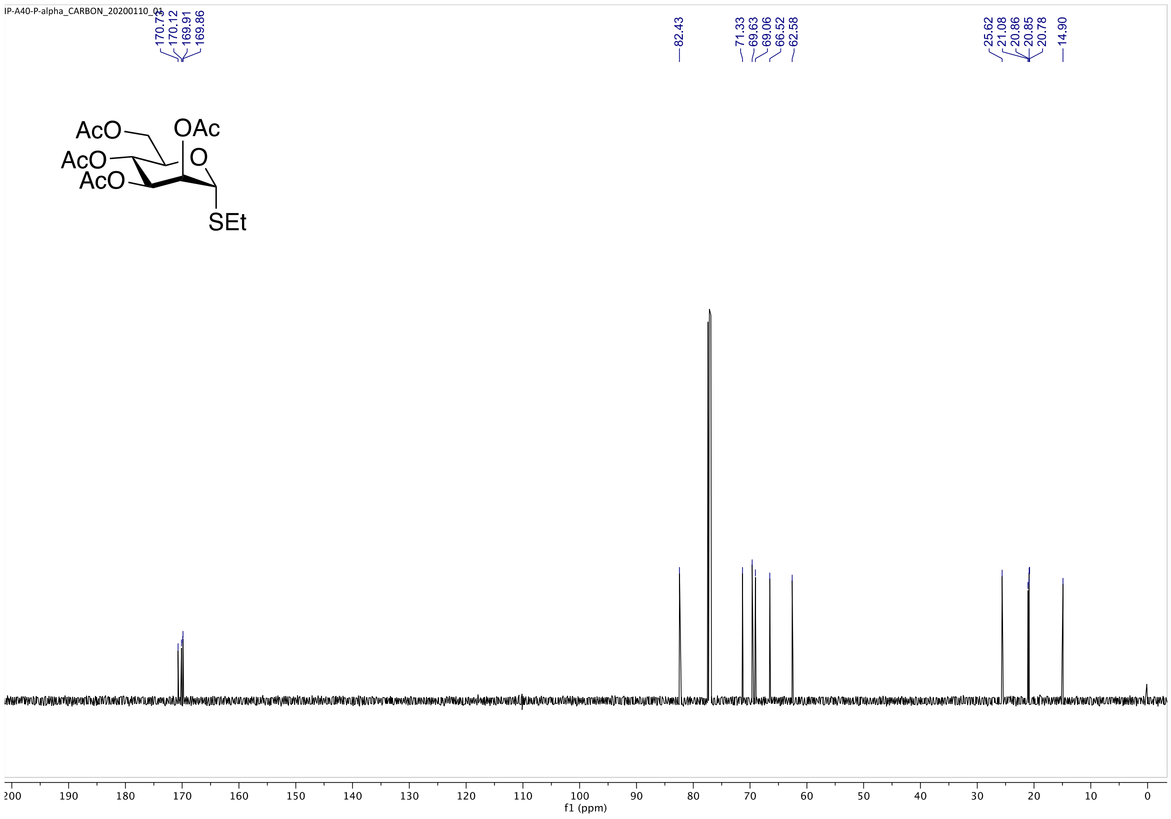

^{1}H NMR (500 MHz, Chloroform-*d*) S3

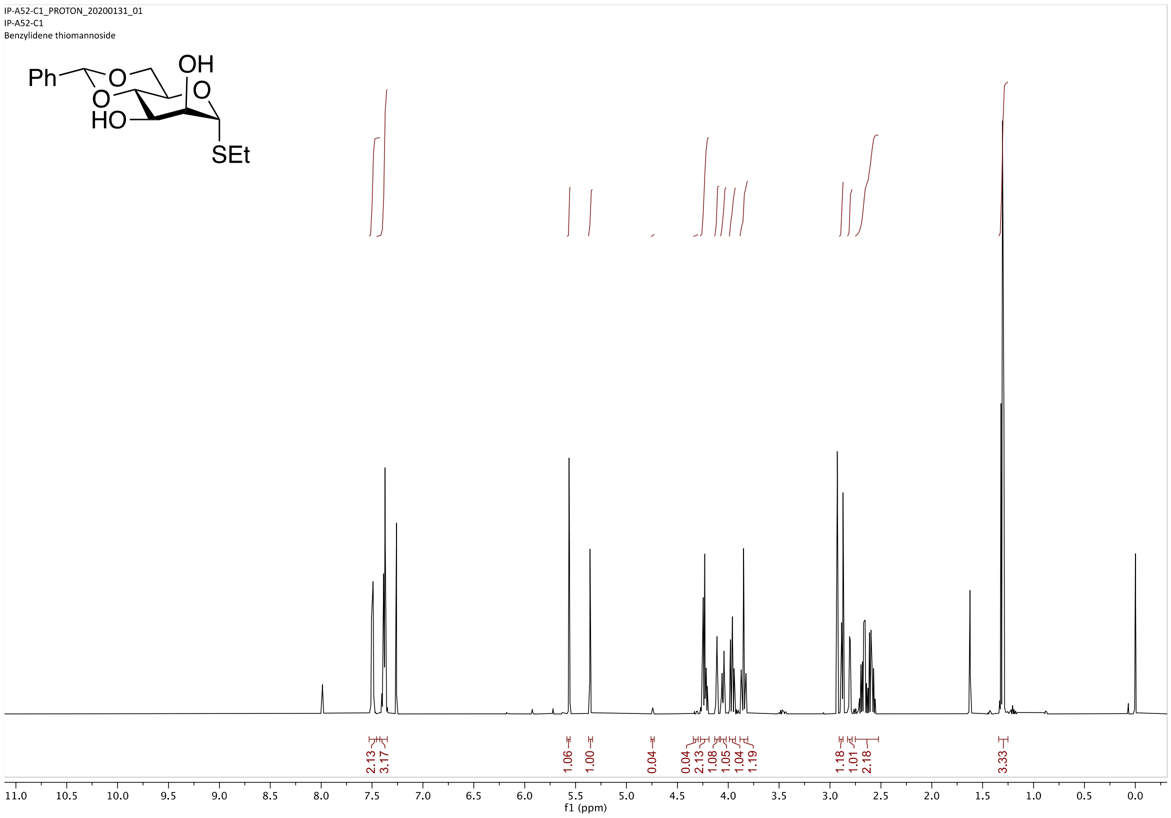

^{1}H NMR (500 MHz, Chloroform-*d*) S4

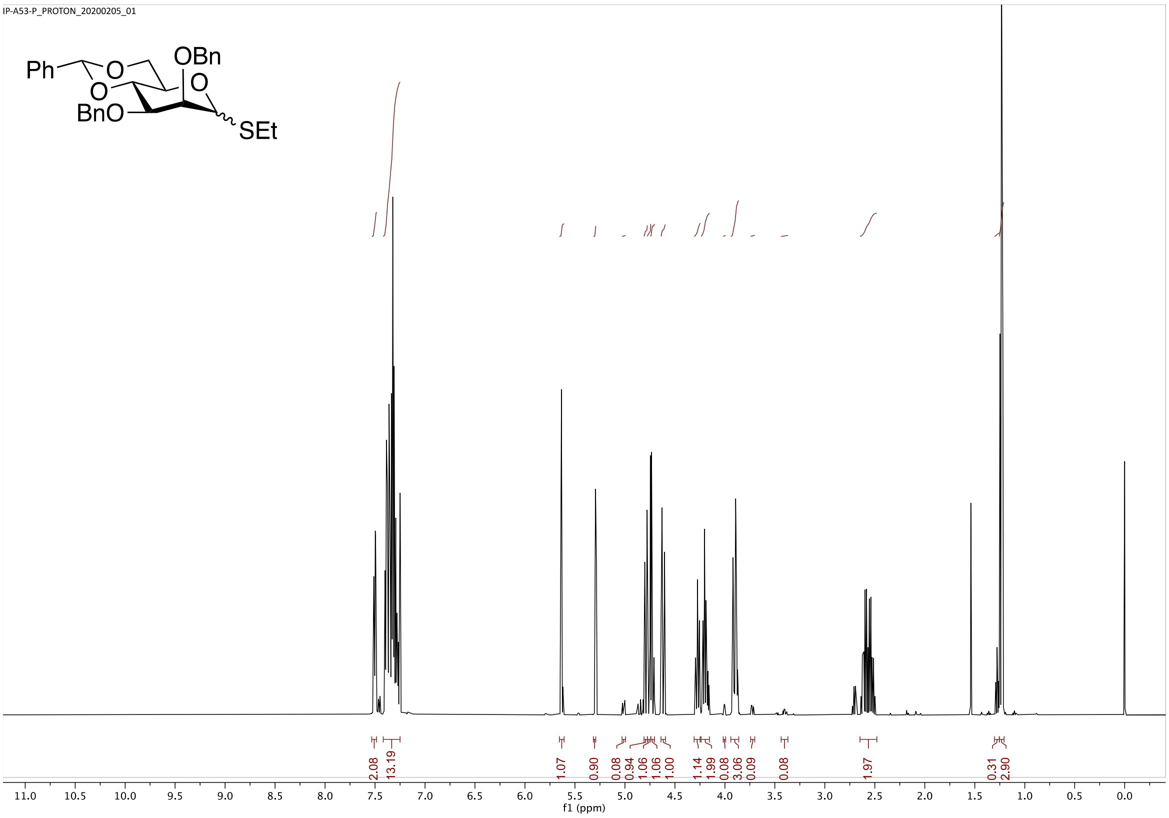

^{13}C NMR (126 MHz, Chloroform-*d*) S4

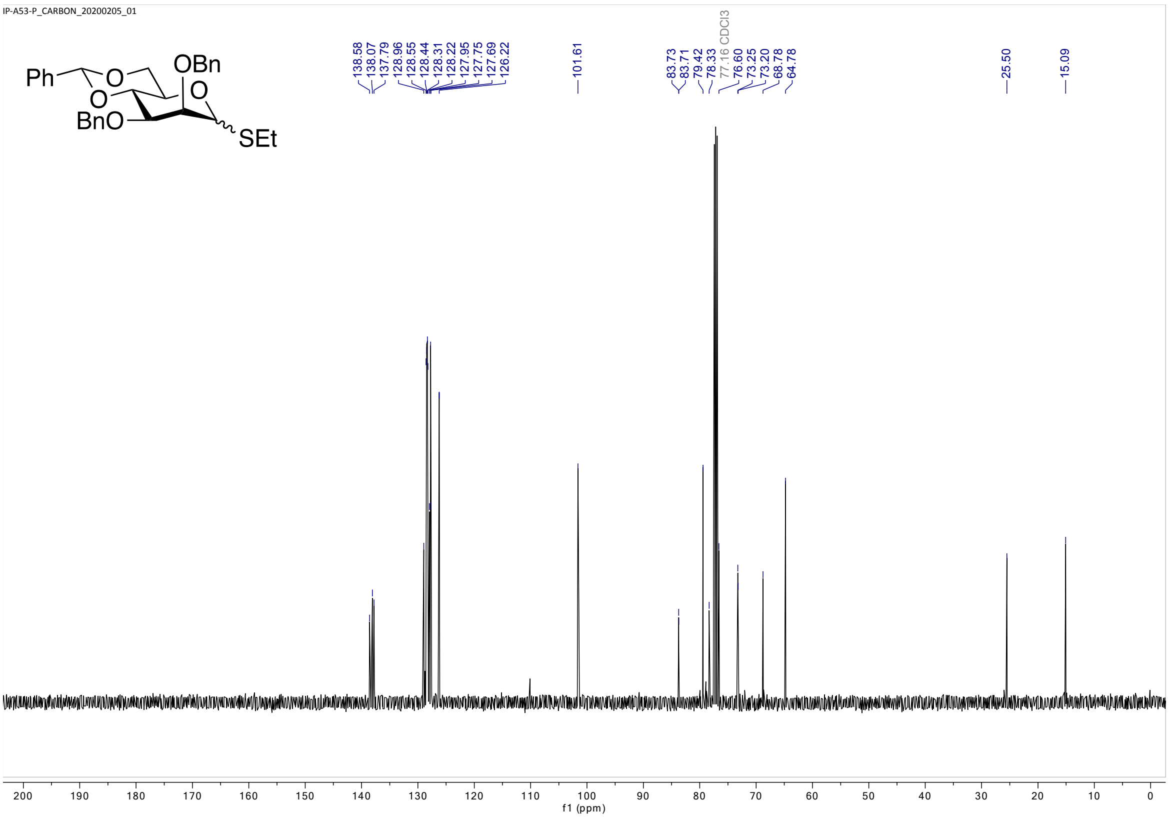

^{1}H NMR (500 MHz, Chloroform-*d*) S5

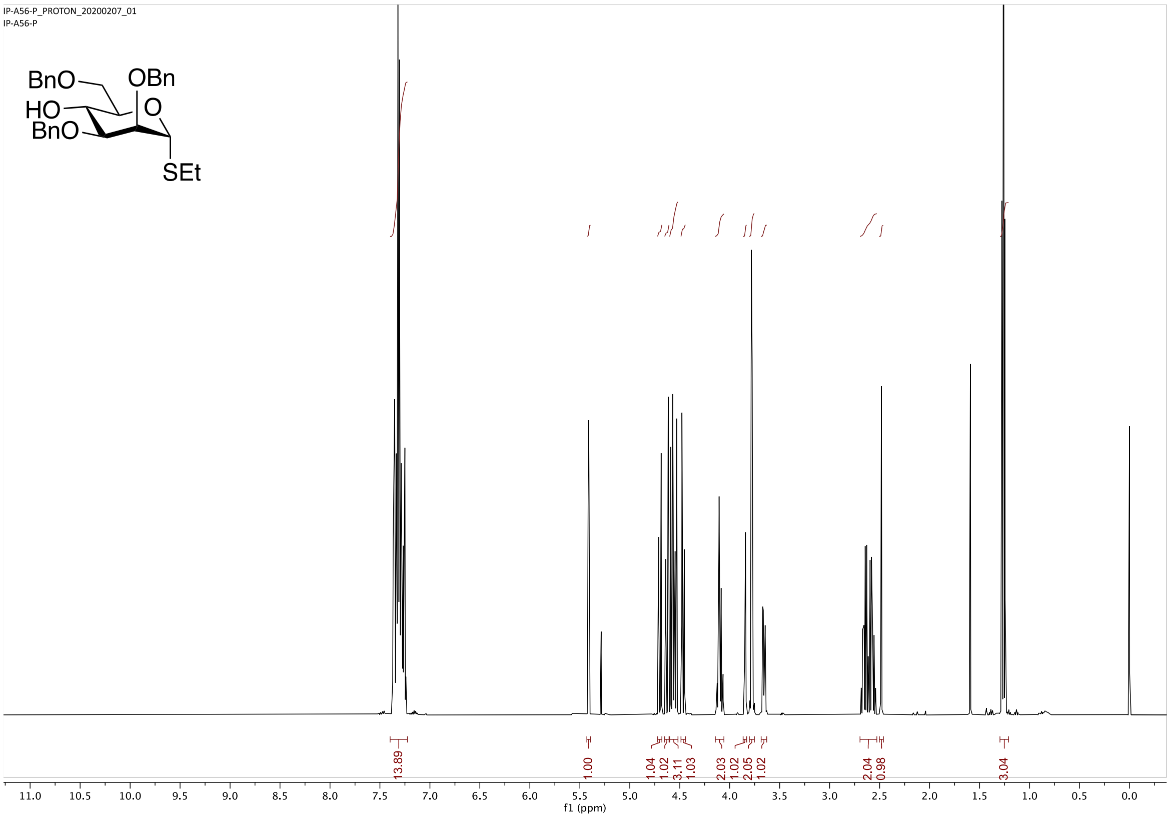

^{13}C NMR (126 MHz, Chloroform-*d*) S5

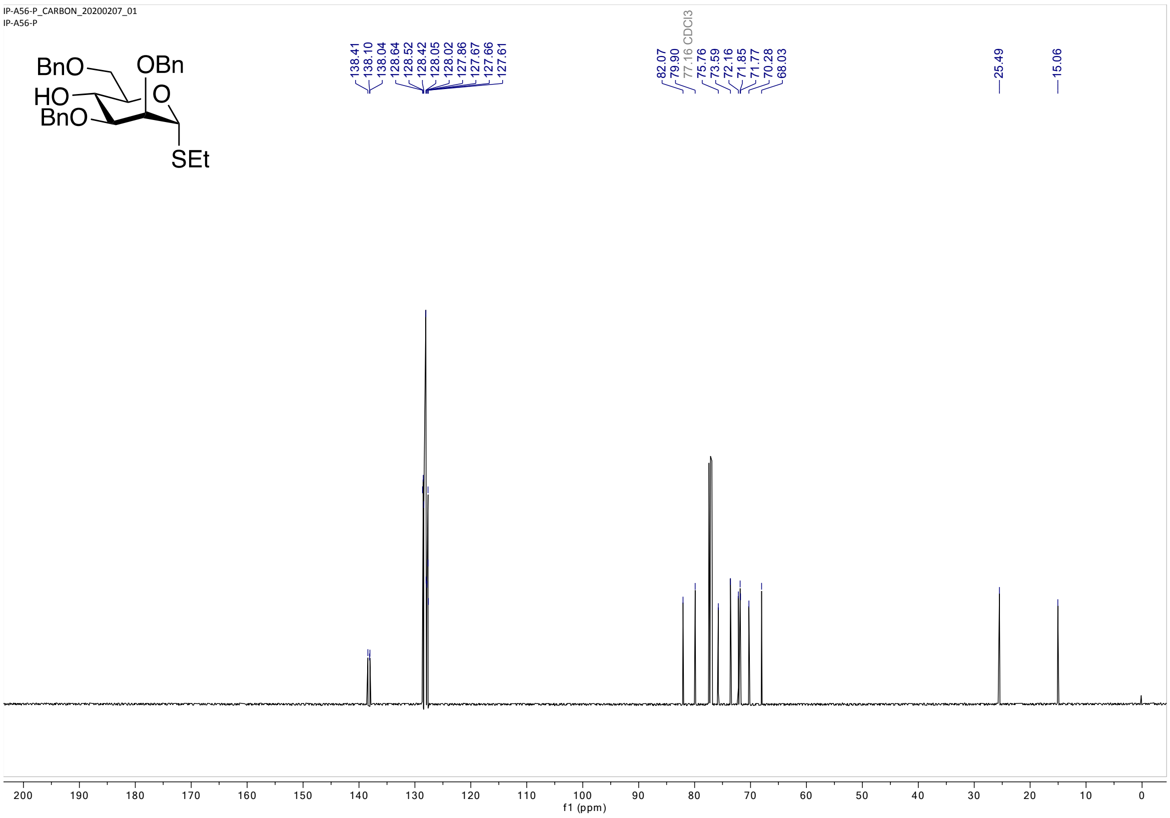

^{1}H NMR (600 MHz, Chloroform-*d*) S6

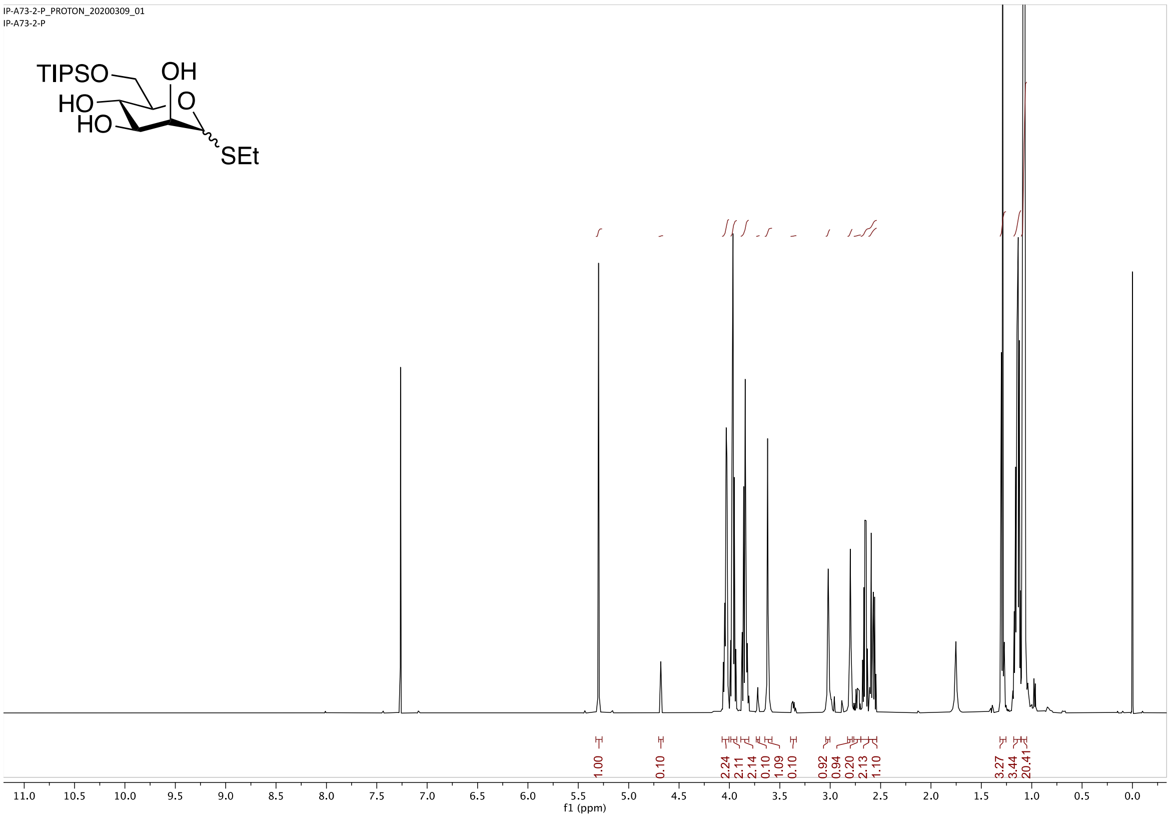

^{13}C NMR (151 MHz, Chloroform-*d*) S6

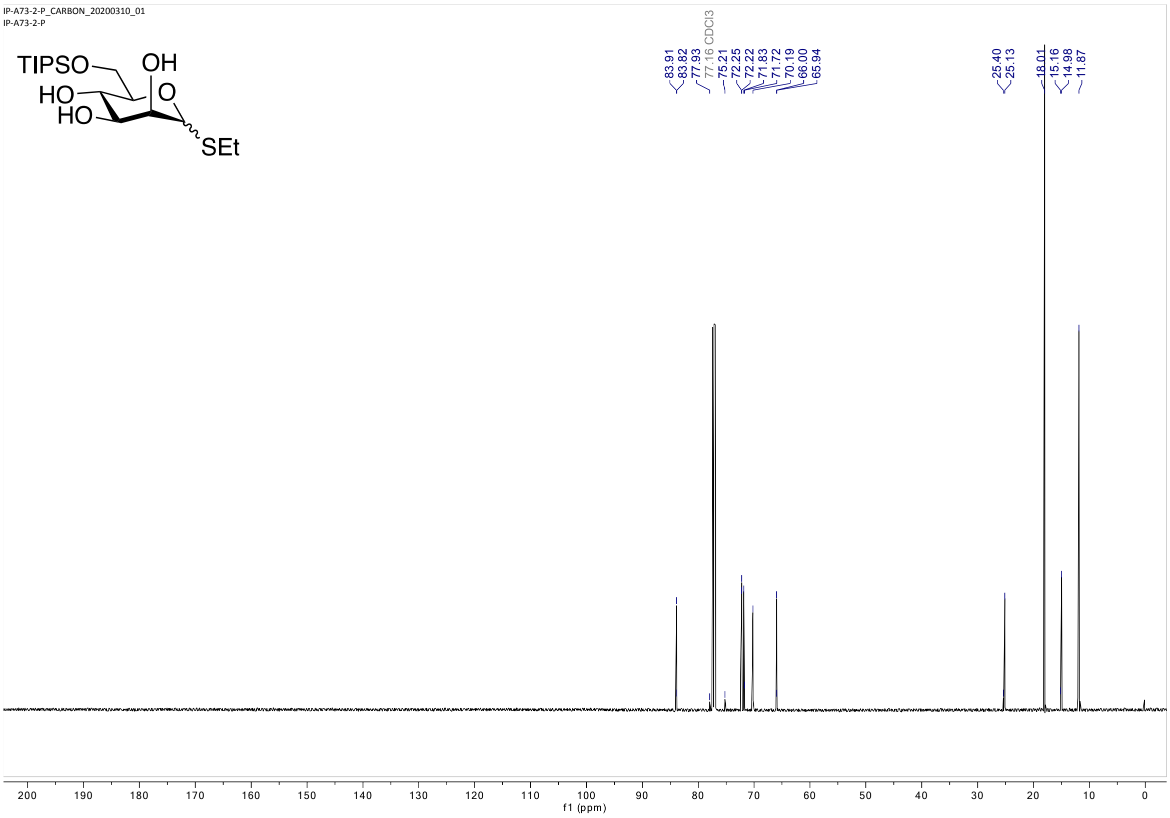

COSY NMR (600 MHz, Chloroform-*d*) S6

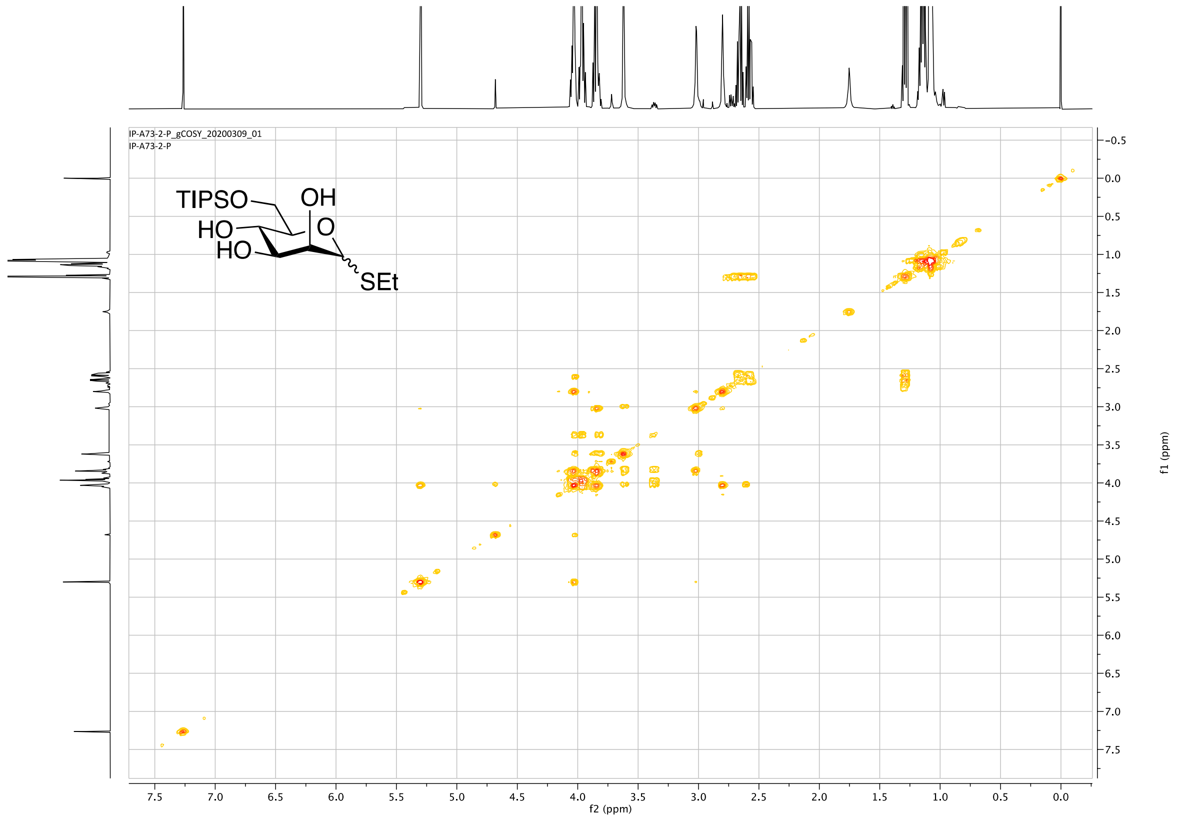

HSQC NMR (600 MHz x 151 MHz, Chloroform-*d*) S6

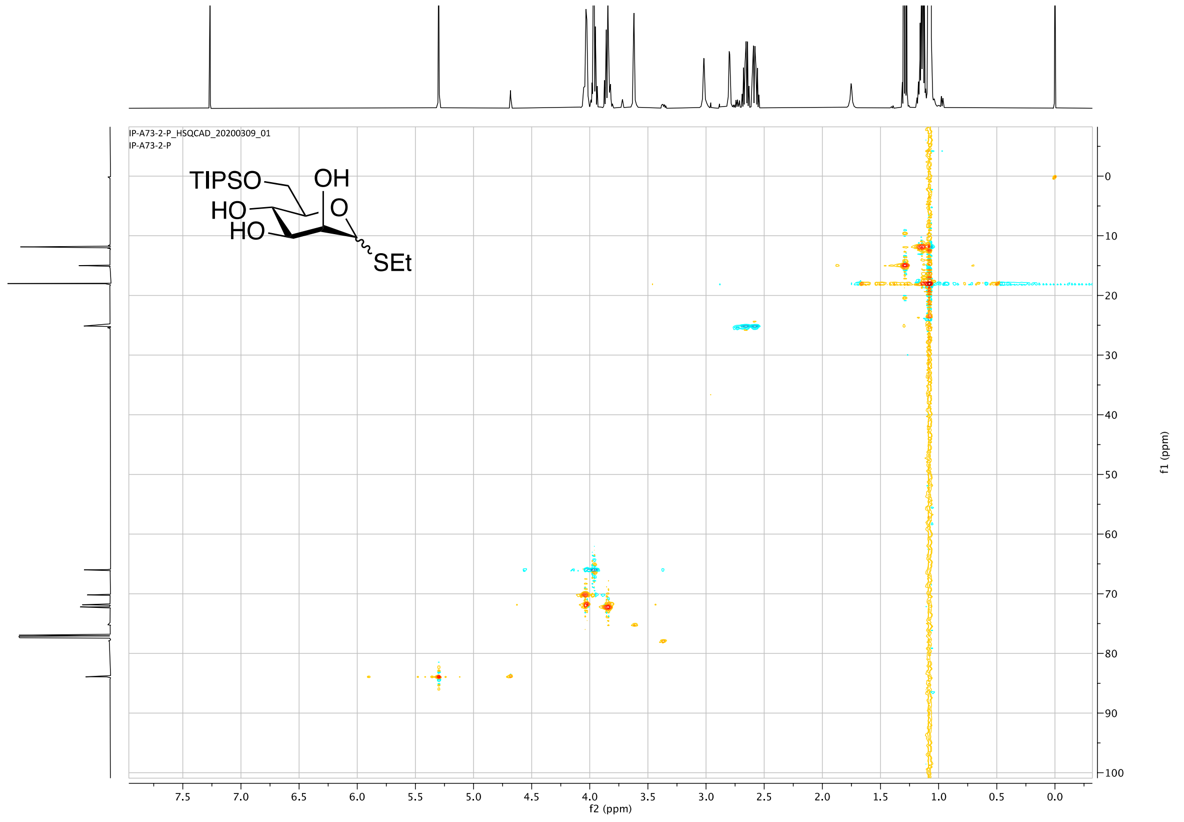

HMBC NMR (600 MHz x 151 MHz, Chloroform-*d*) S6

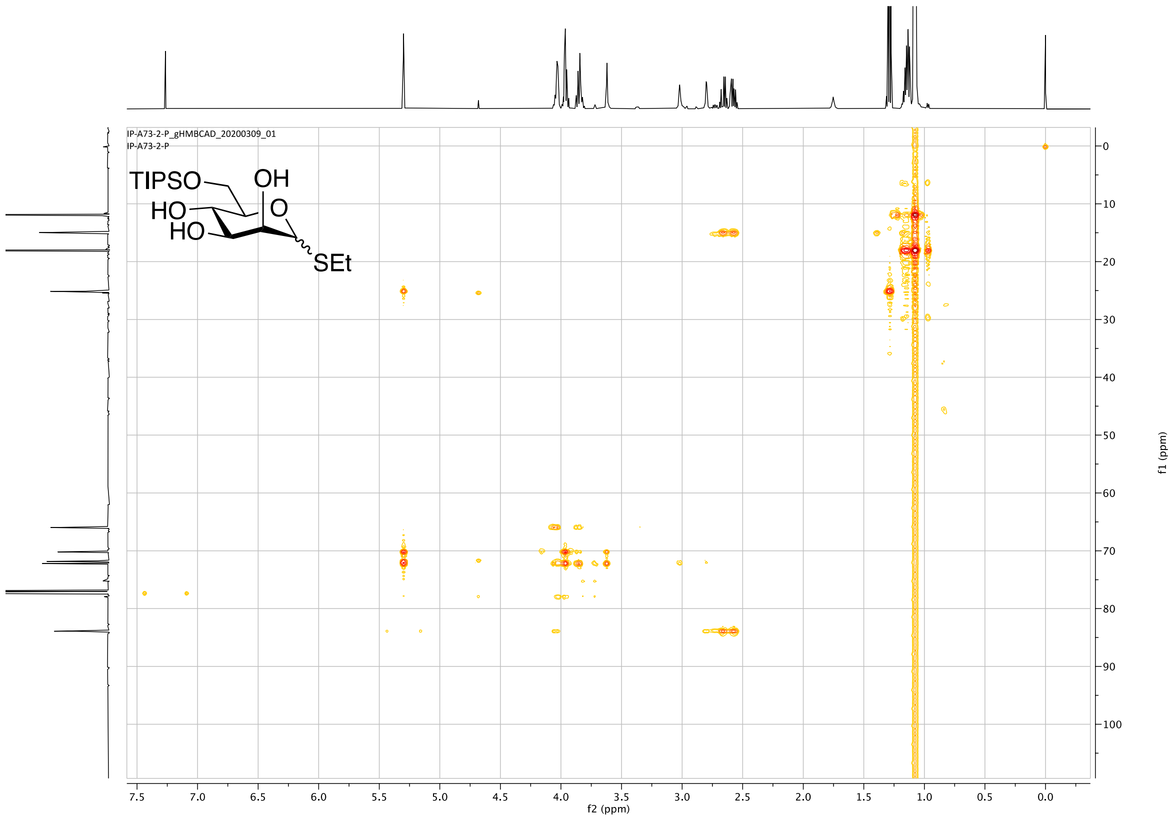

^{1}H NMR (600 MHz, Chloroform-*d*) S7

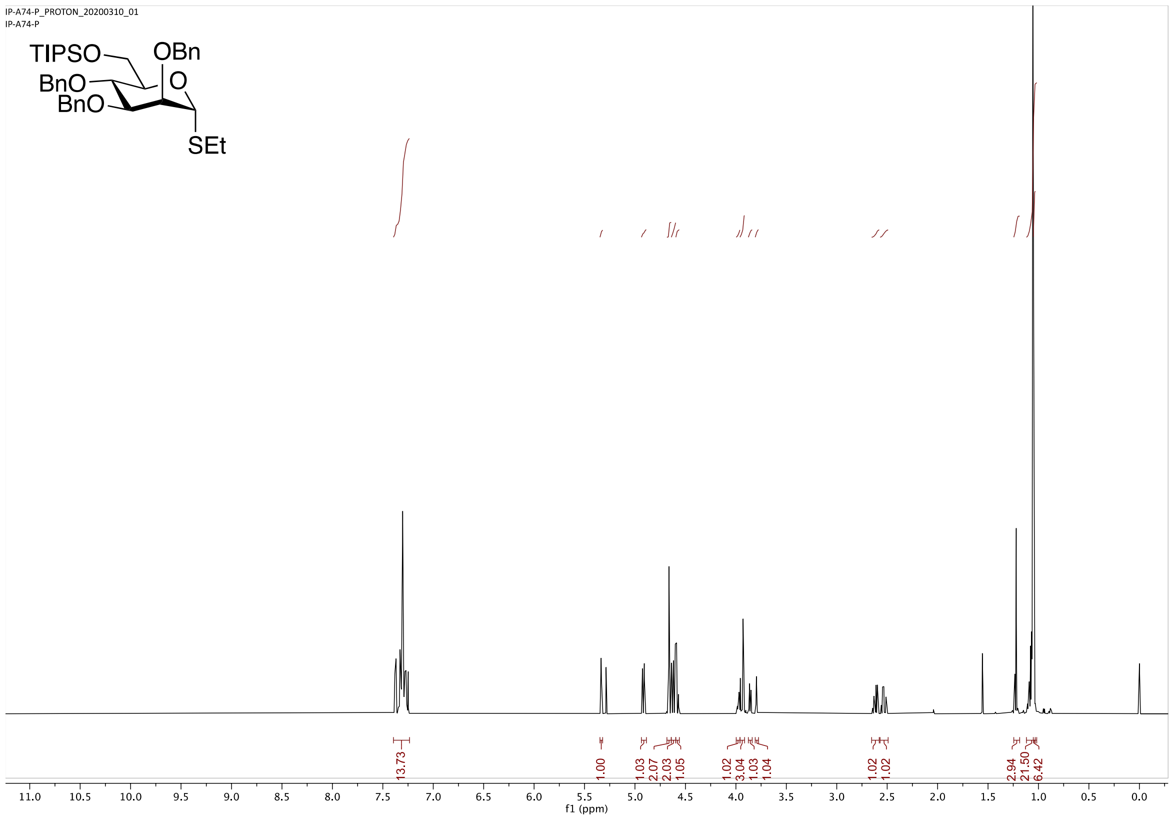

^{13}C NMR (151 MHz, Chloroform-*d*) S7

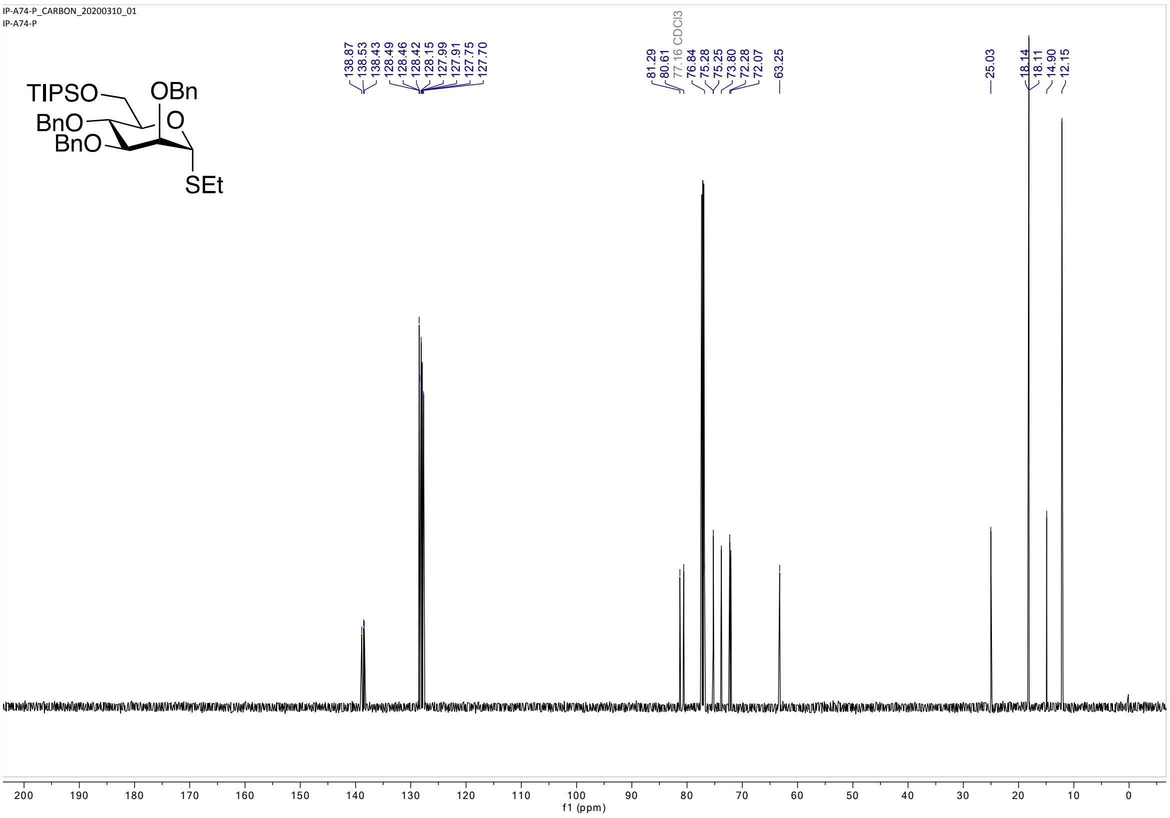

COSY NMR (600 MHz, Chloroform-*d*) S7

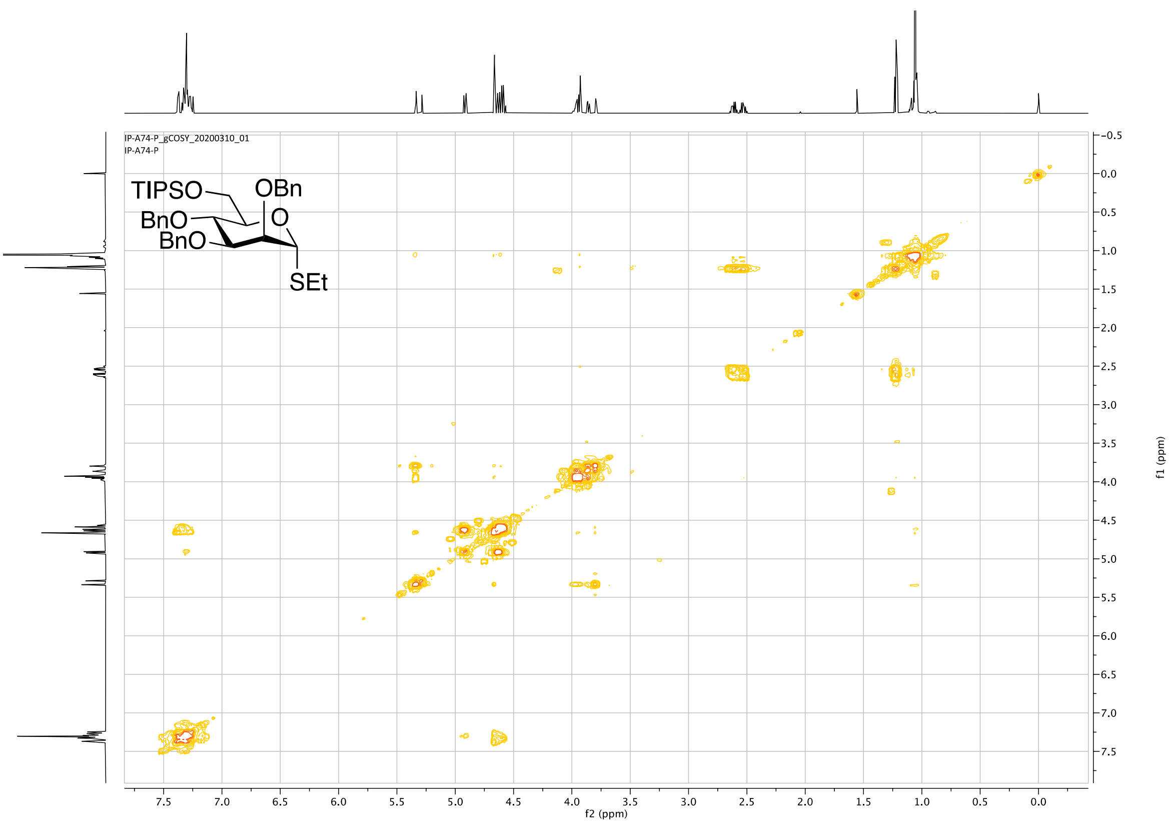

DEPT NMR (151 MHz, Chloroform-*d*) S7

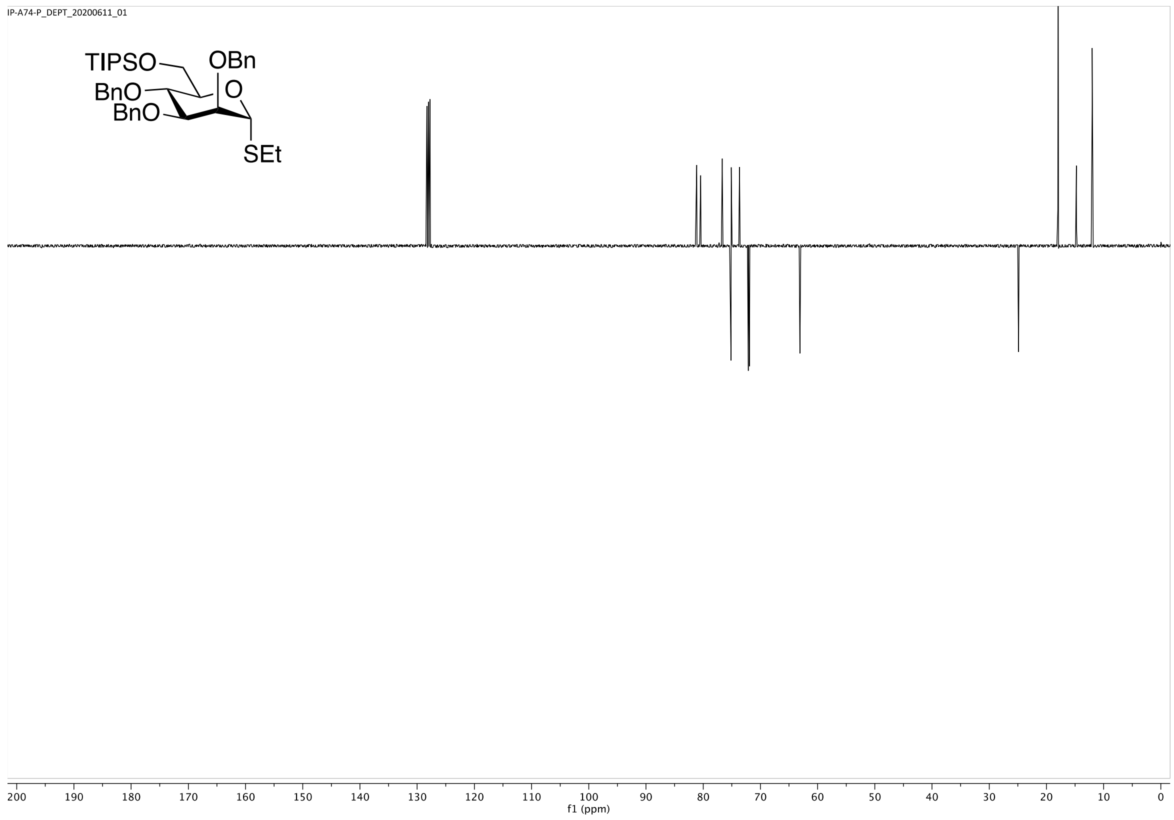

HSQC NMR (600 MHz x 151 MHz, Chloroform-*d*) S7

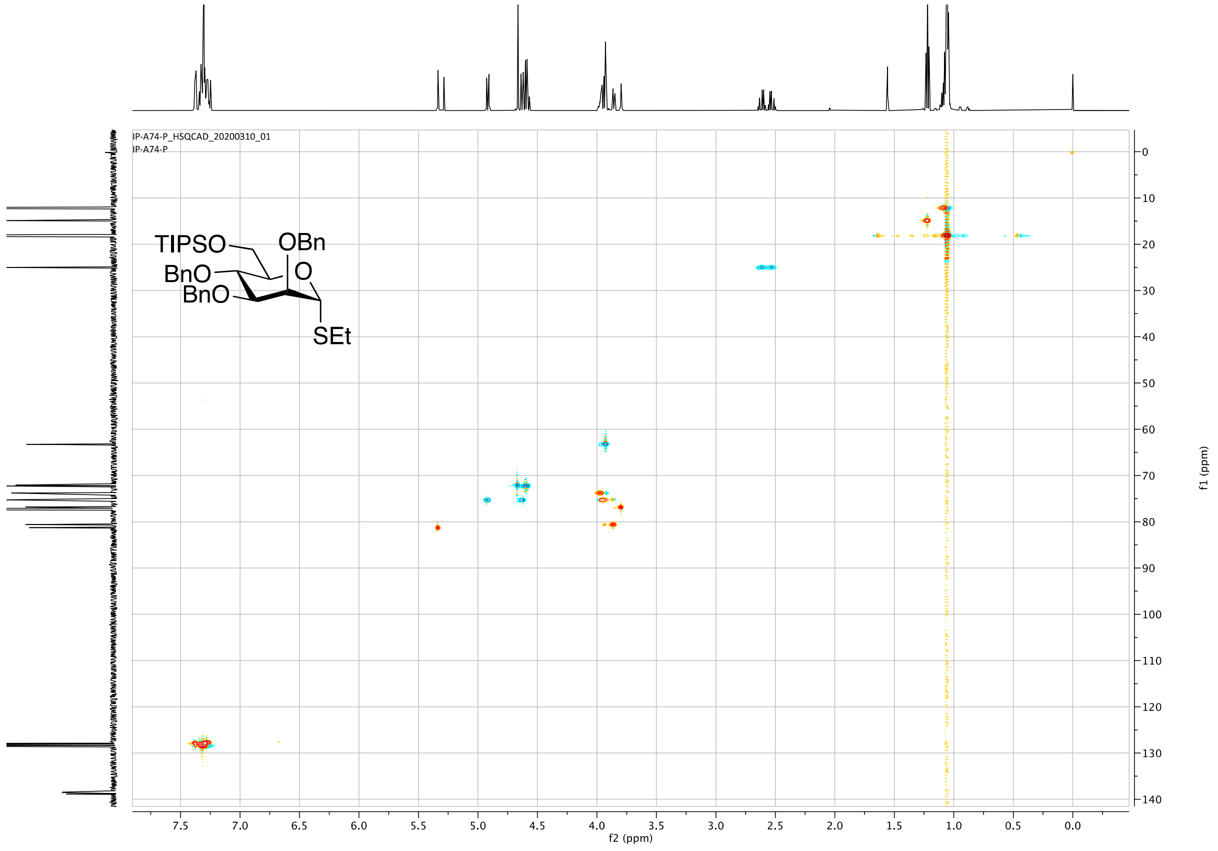

HMBC NMR (600 MHz x 151 MHz, Chloroform-*d*) S7

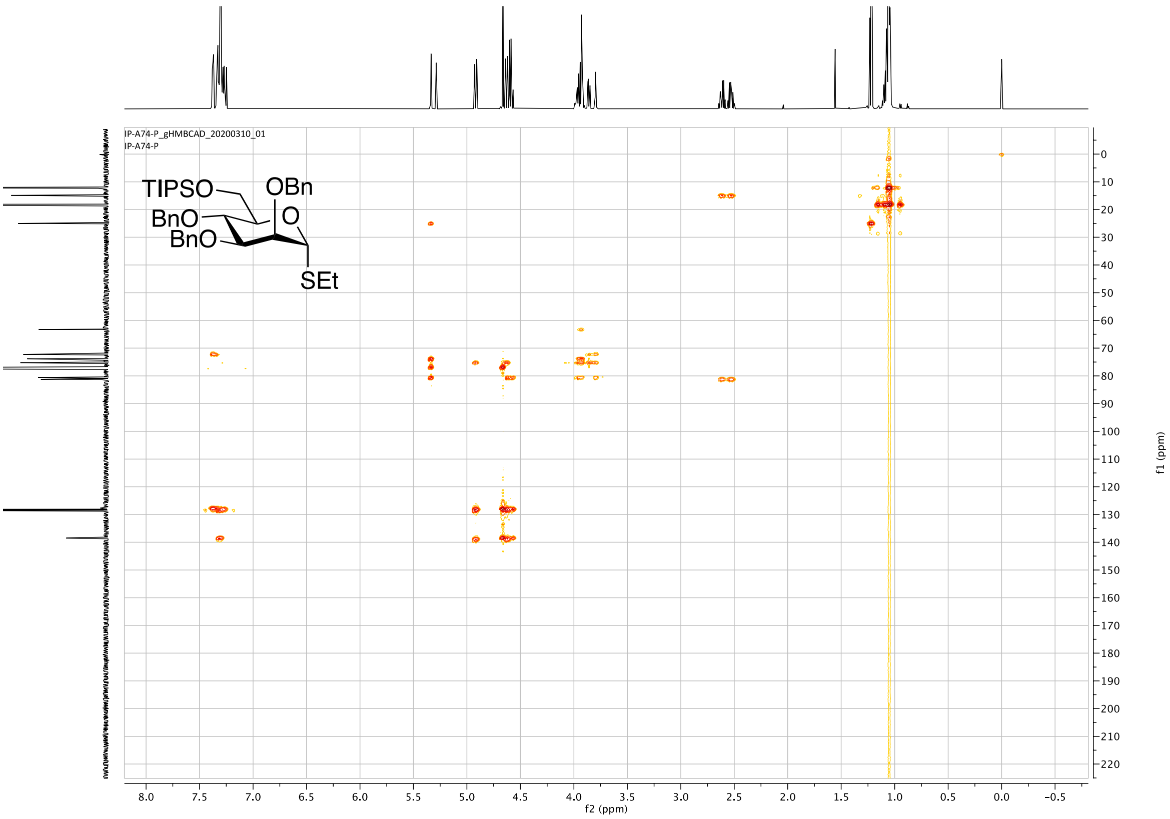

^{1}H NMR (600 MHz, Chloroform-*d*) S8

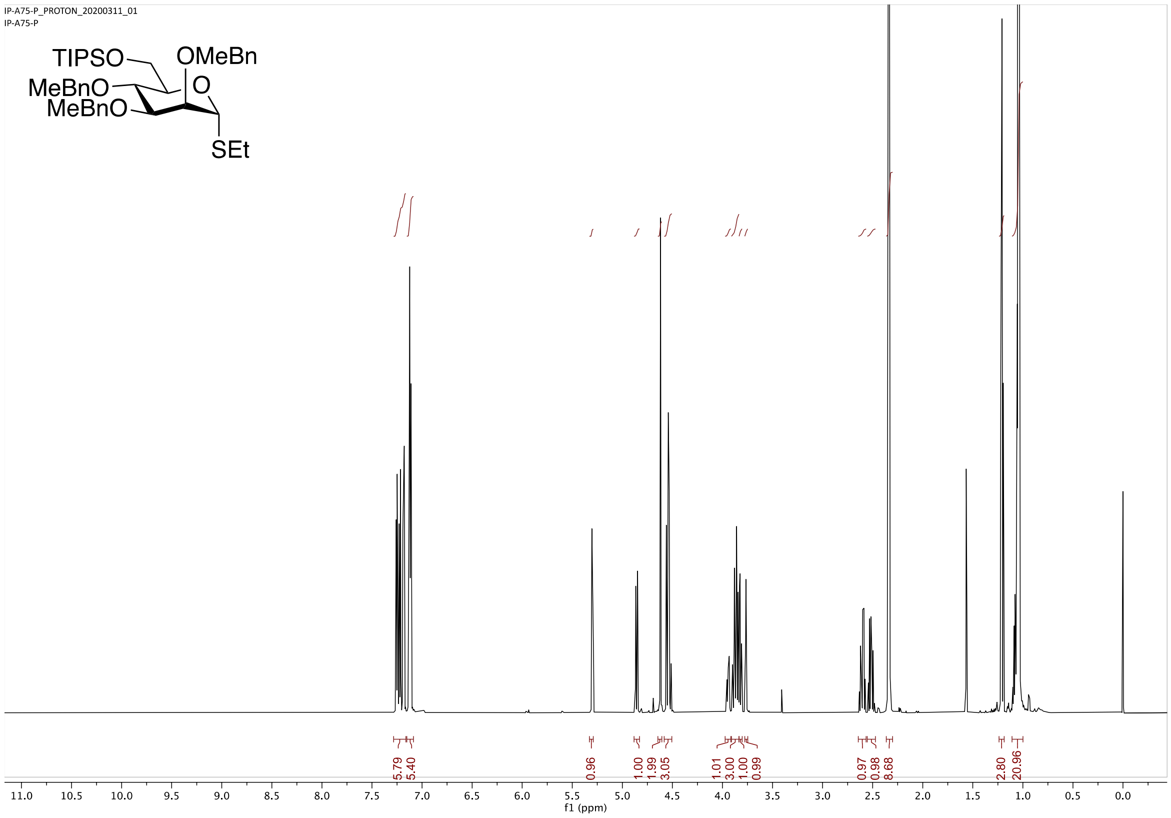

^{13}C NMR (151 MHz, Chloroform-*d*) S8

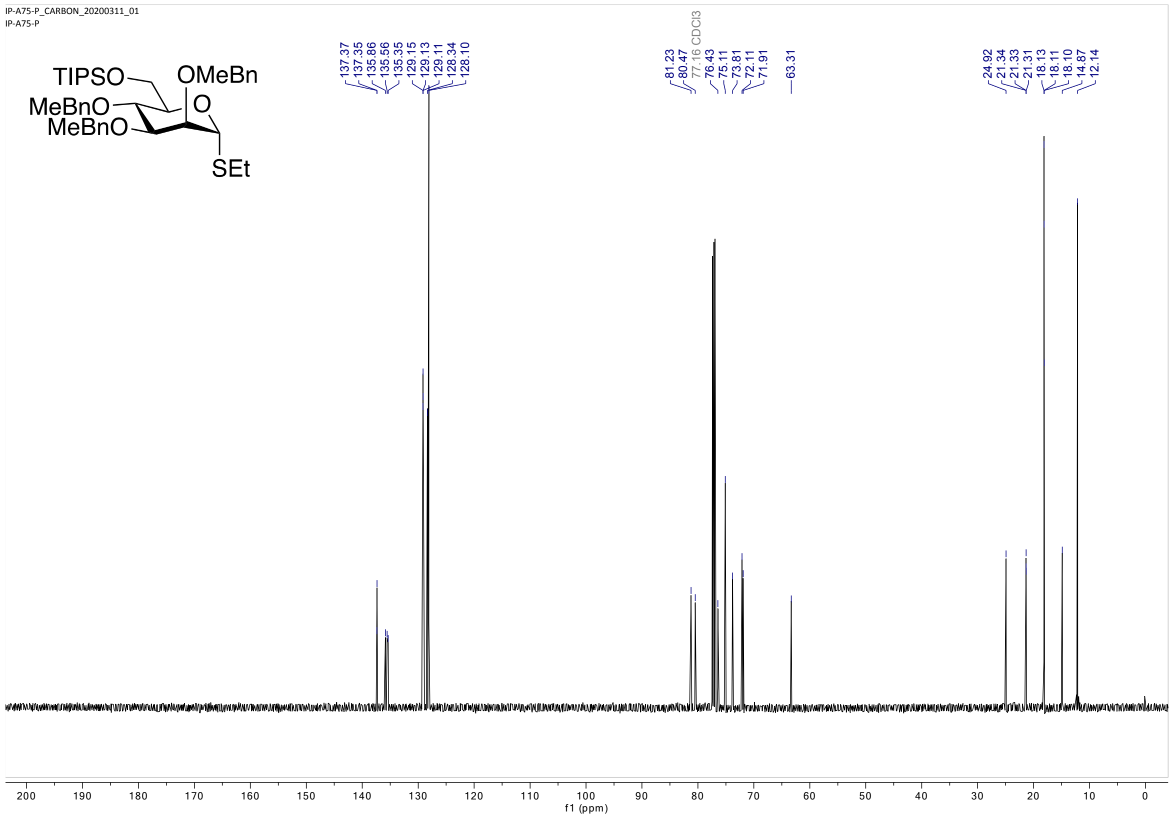

COSY NMR (600 MHz, Chloroform-*d*) S8

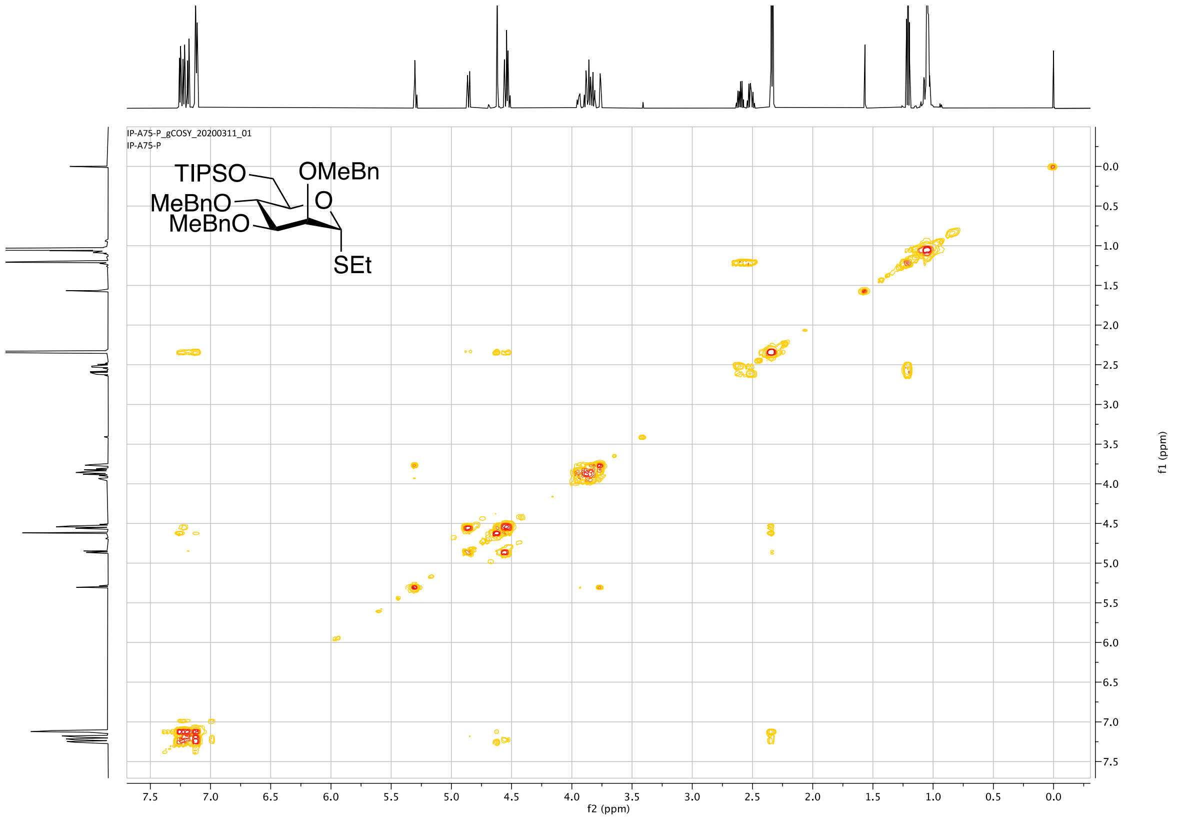

HSQC NMR (600 MHz x 151 MHz, Chloroform-*d*) S8

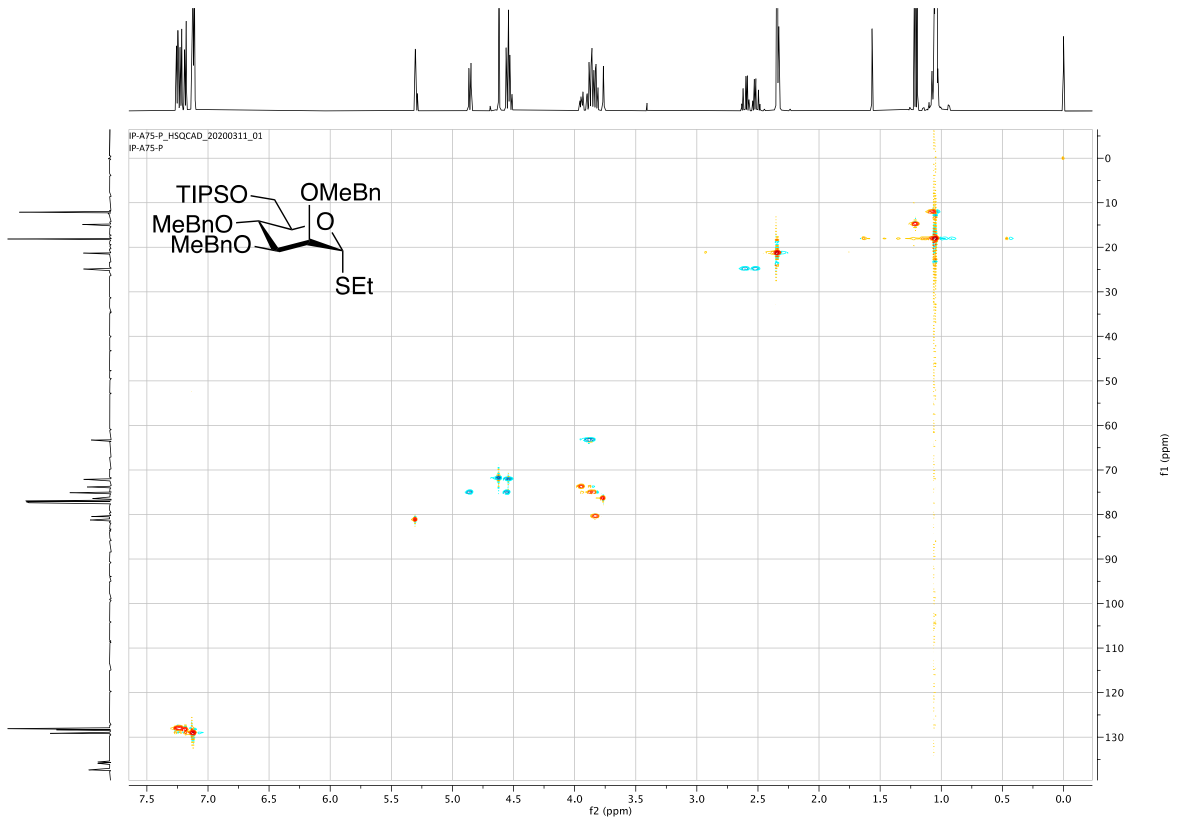

HMBC NMR (600 MHz x 151 MHz, Chloroform-*d*) S8

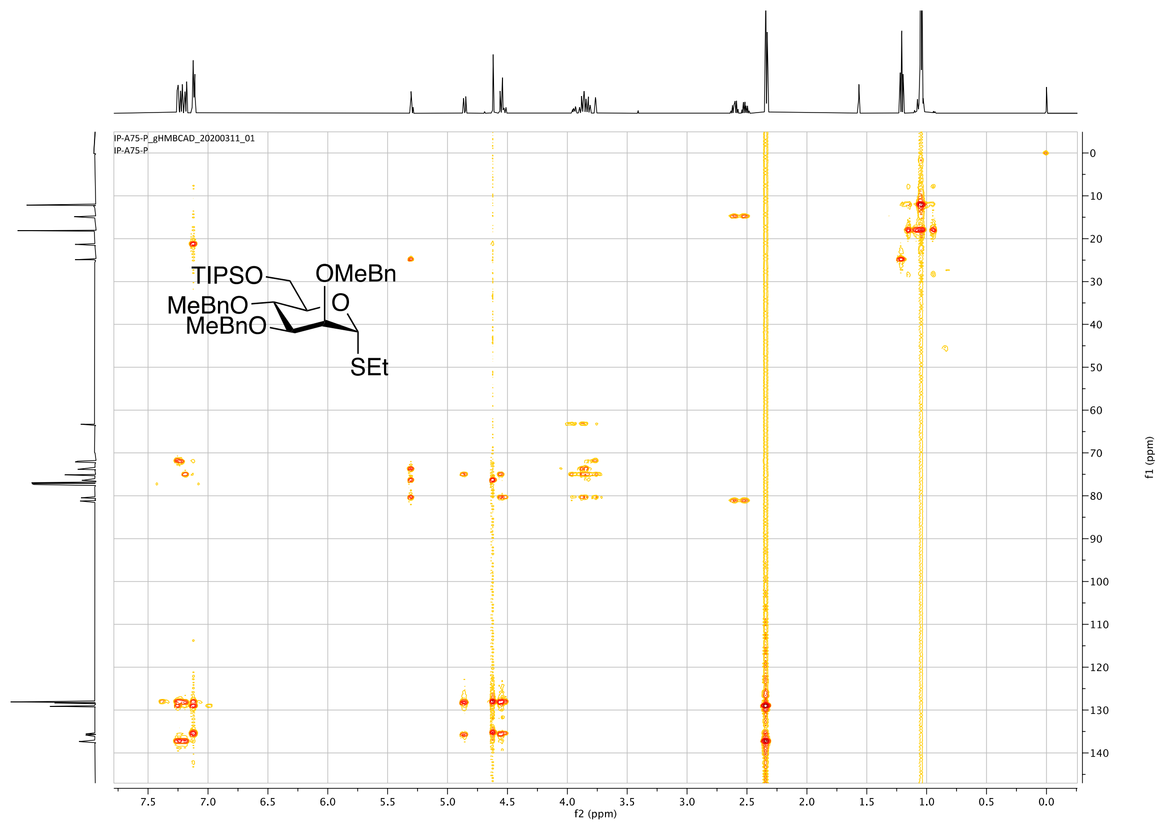

^{1}H NMR (500 MHz, Chloroform-*d*) S9

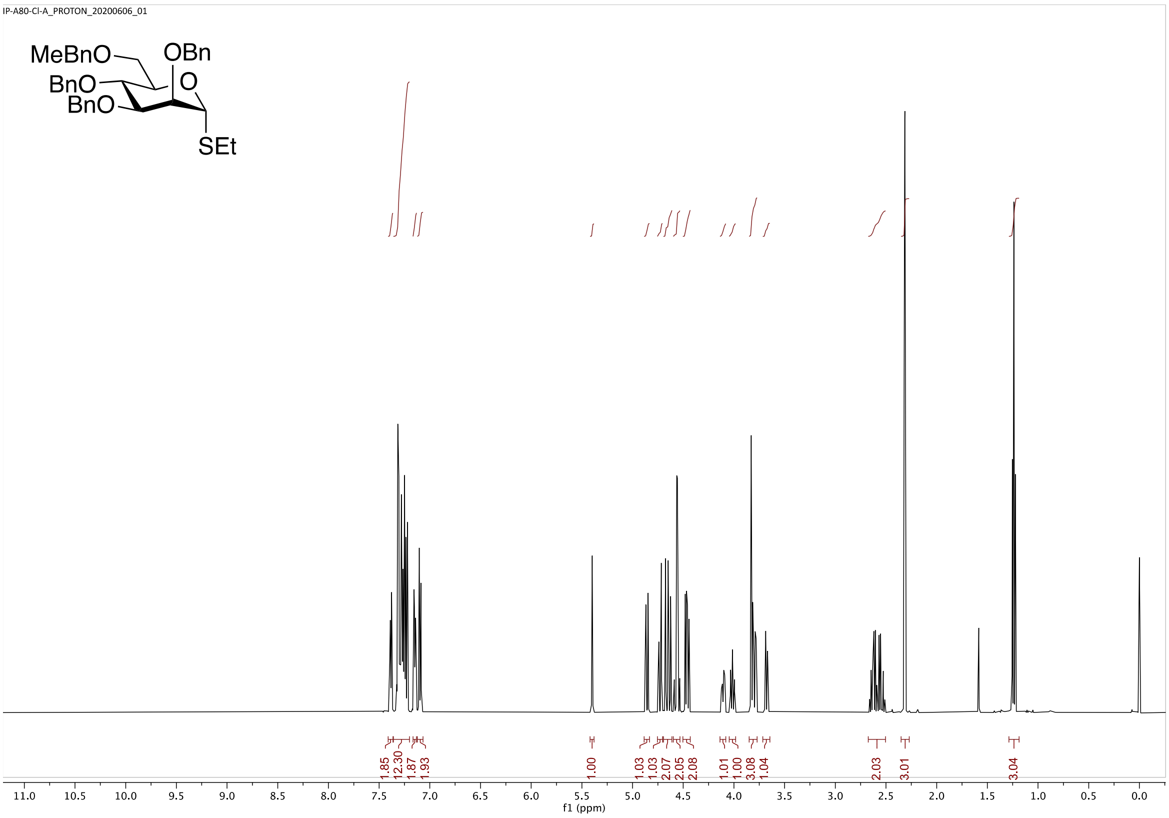

^{13}C NMR (126 MHz, Chloroform-*d*) S9

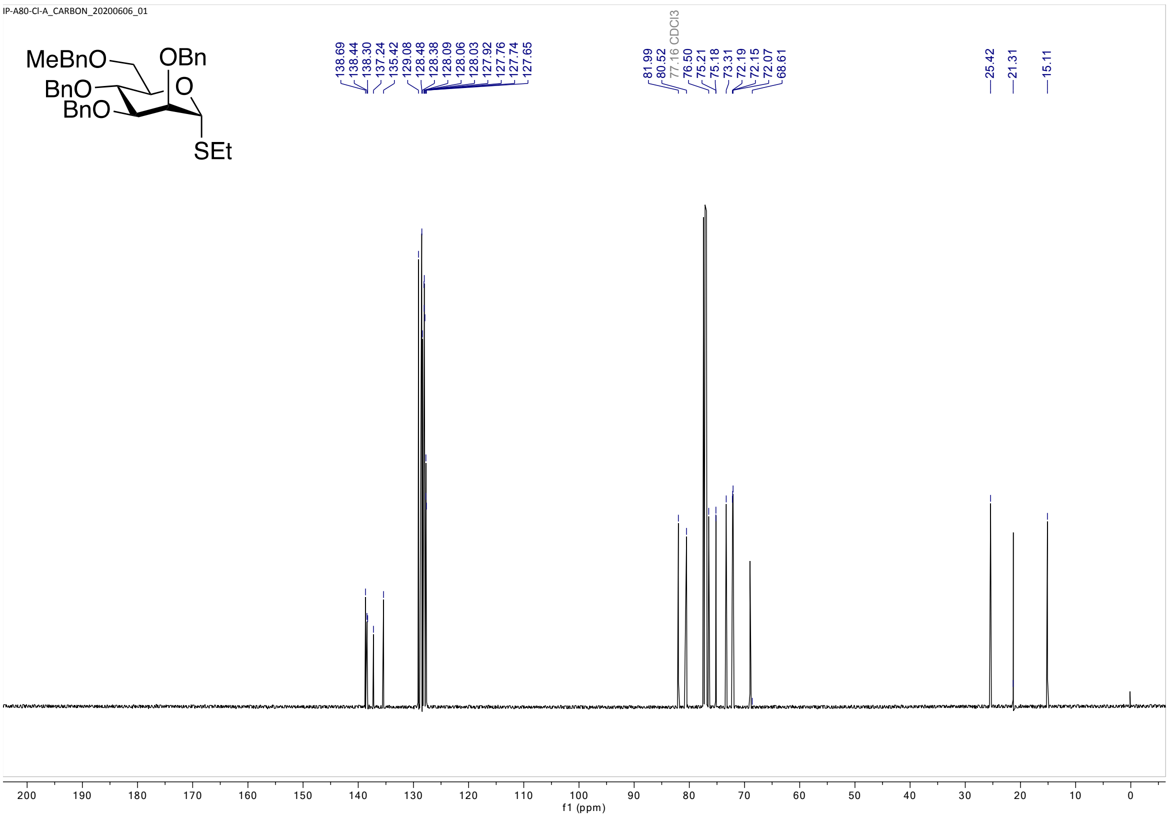

COSY NMR (500 MHz, Chloroform-*d*) S9

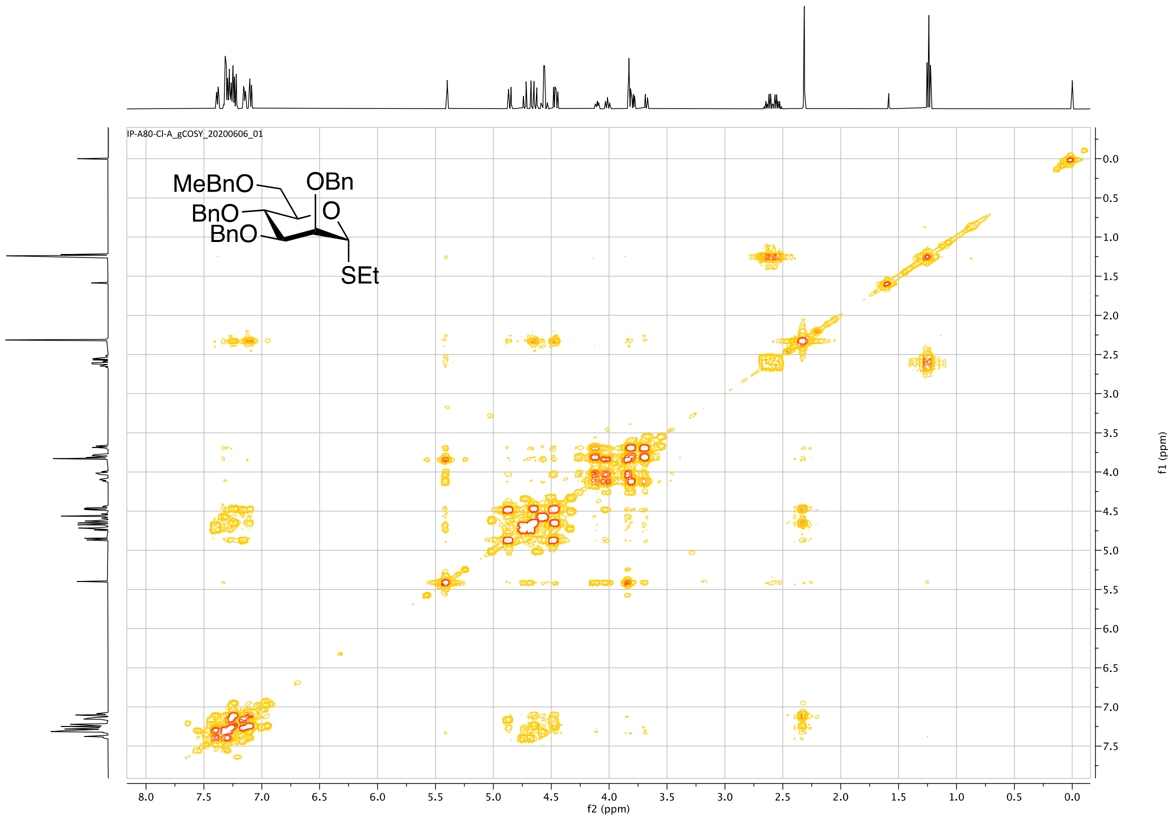

HSQC NMR (500 MHz x 126 MHz, Chloroform-*d*) S9

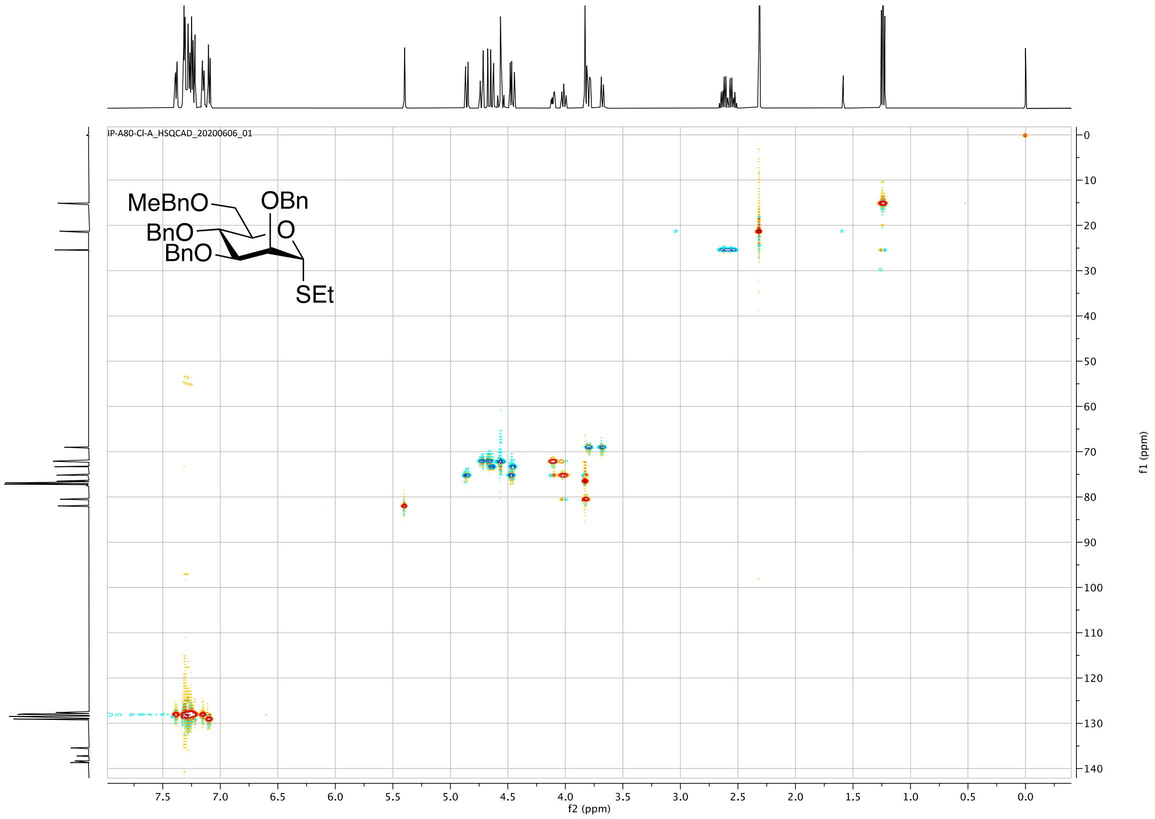

HMBC NMR (500 MHz x 126 MHz, Chloroform-*d*) S9

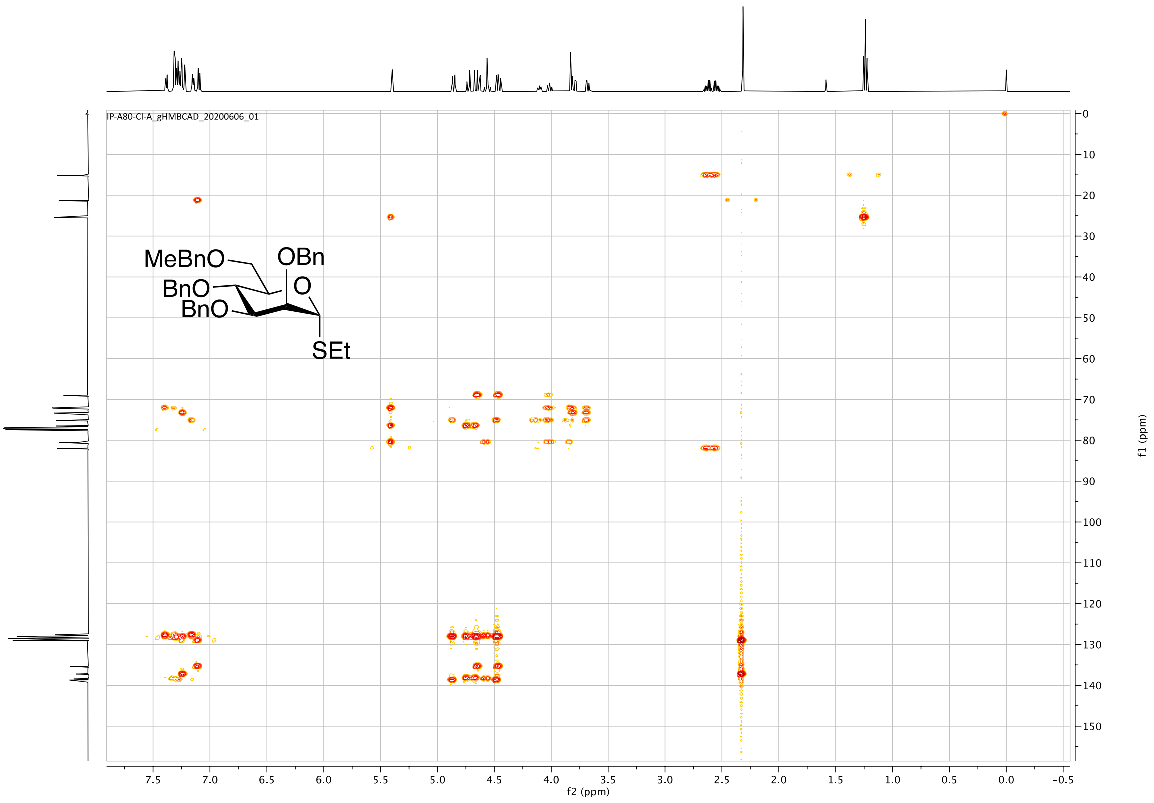

^{1}H NMR (500 MHz, Chloroform-*d*) S10

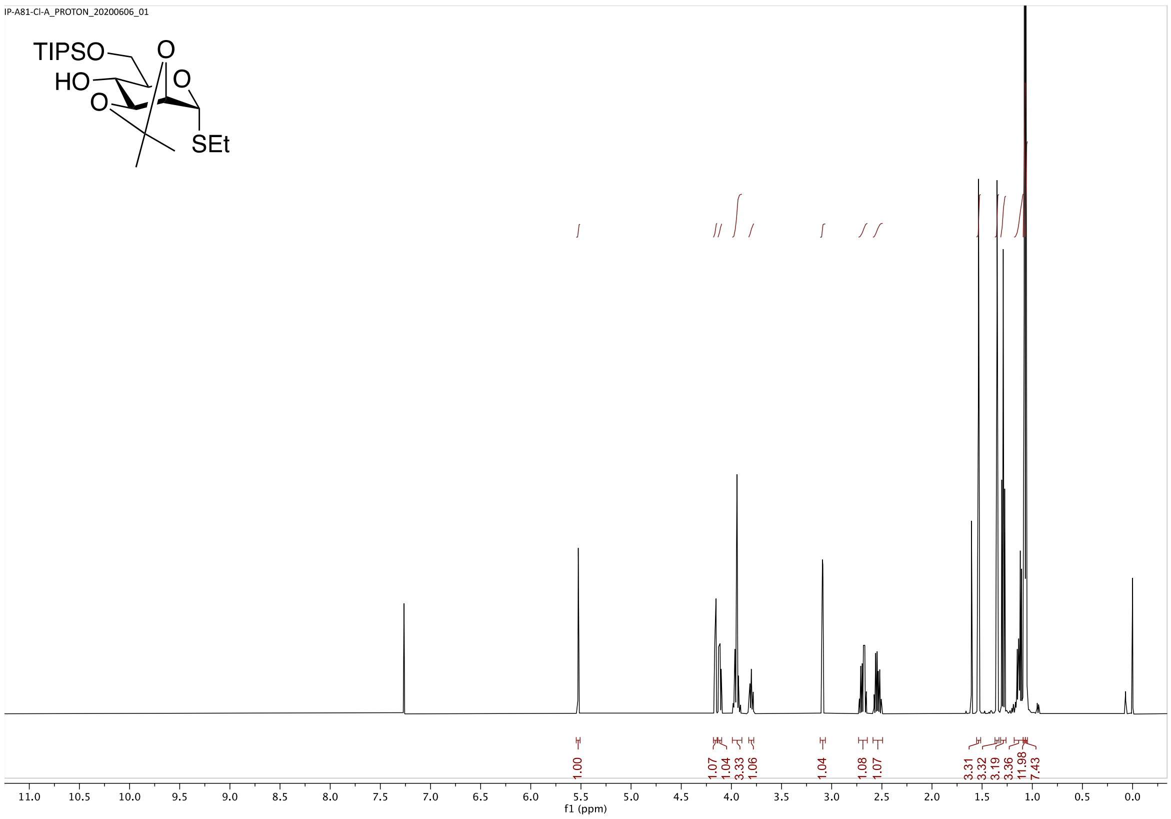

^{13}C NMR (126 MHz, Chloroform-*d*) S10

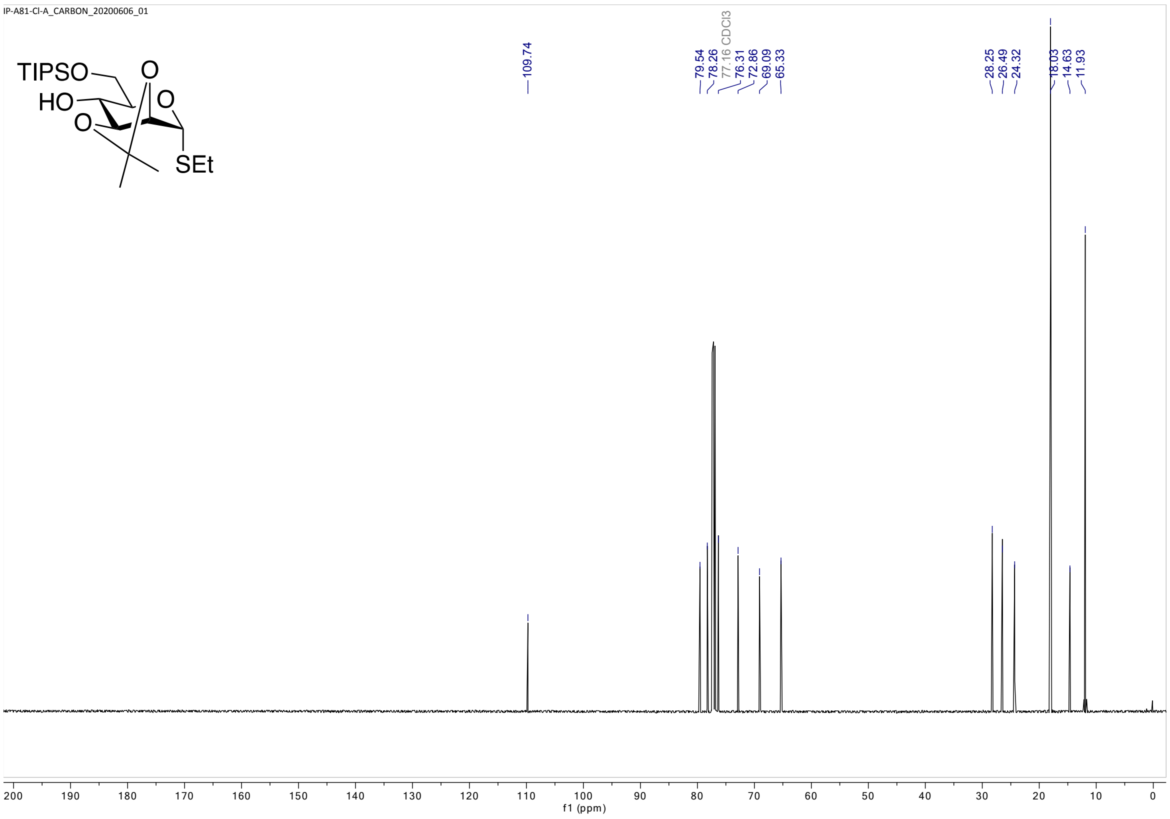

COSY NMR (500 MHz, Chloroform-*d*) S10

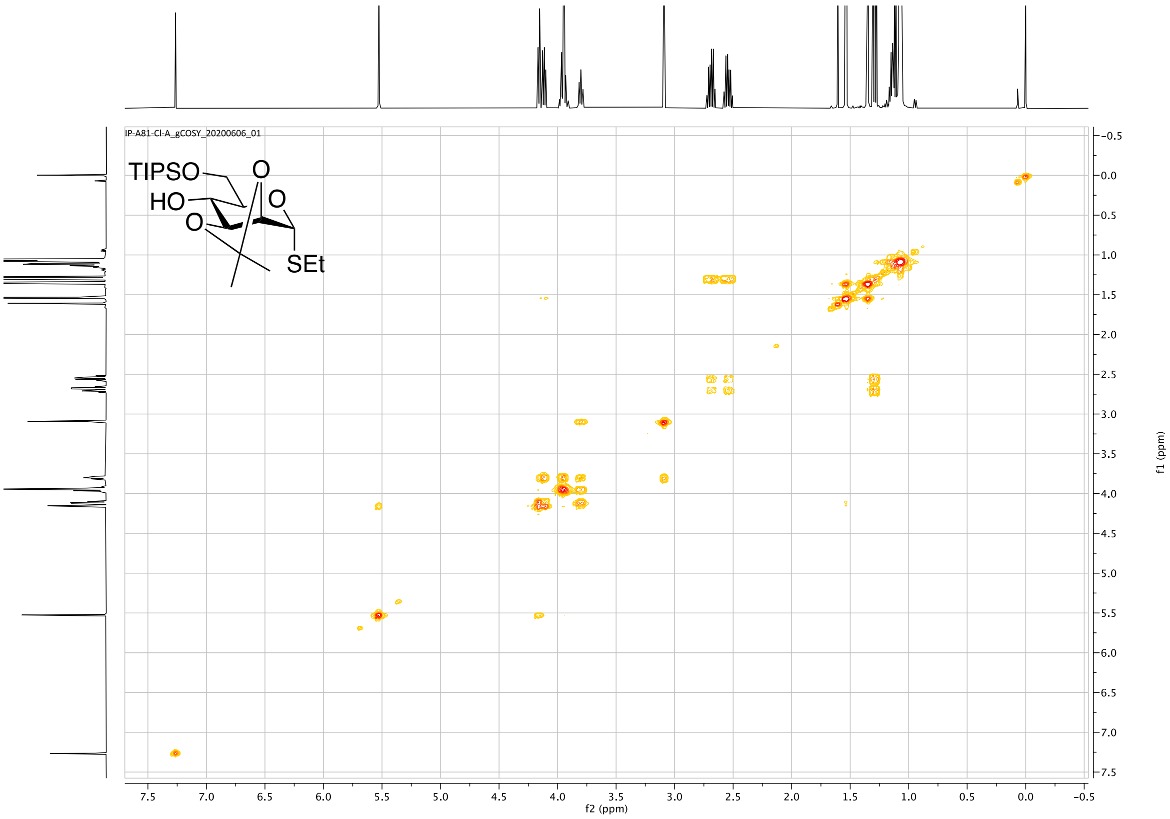

HSQC NMR (500 MHz x 126 MHz, Chloroform-*d*) S10

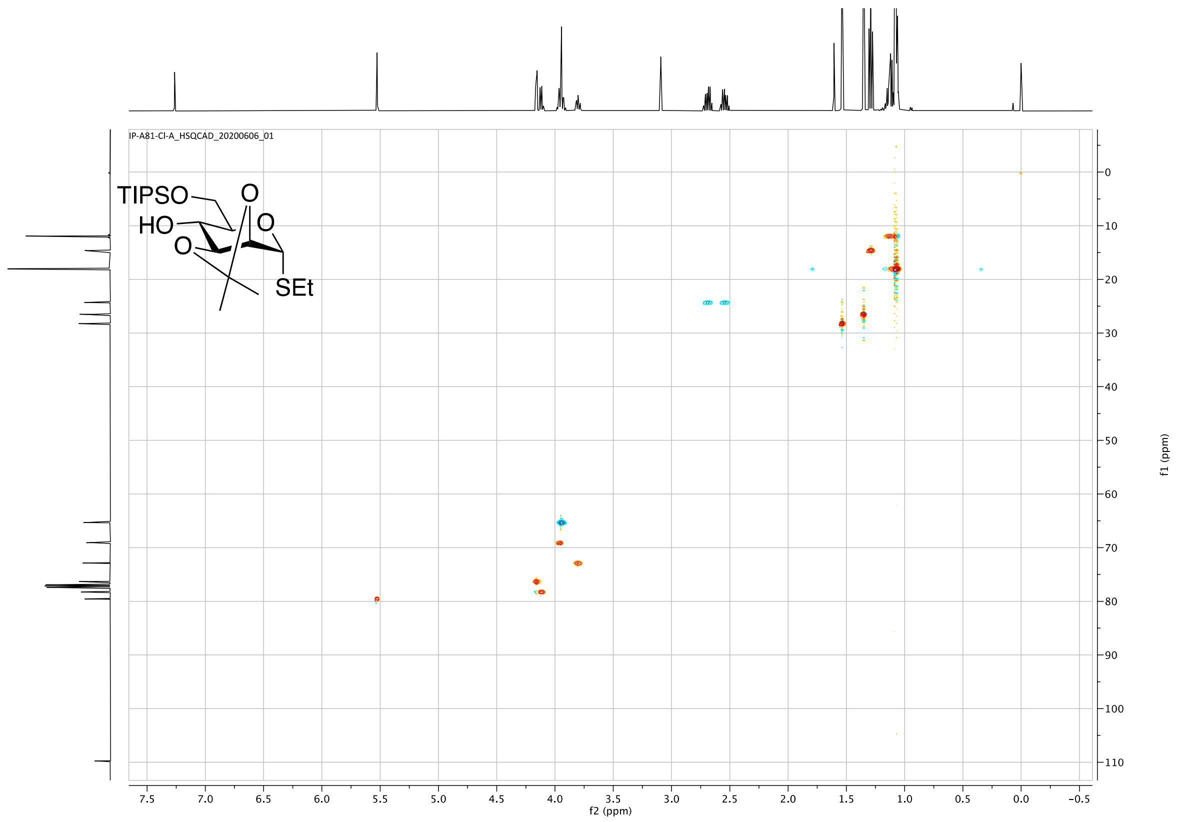

HMBC NMR (500 MHz x 126 MHz, Chloroform-*d*) S10

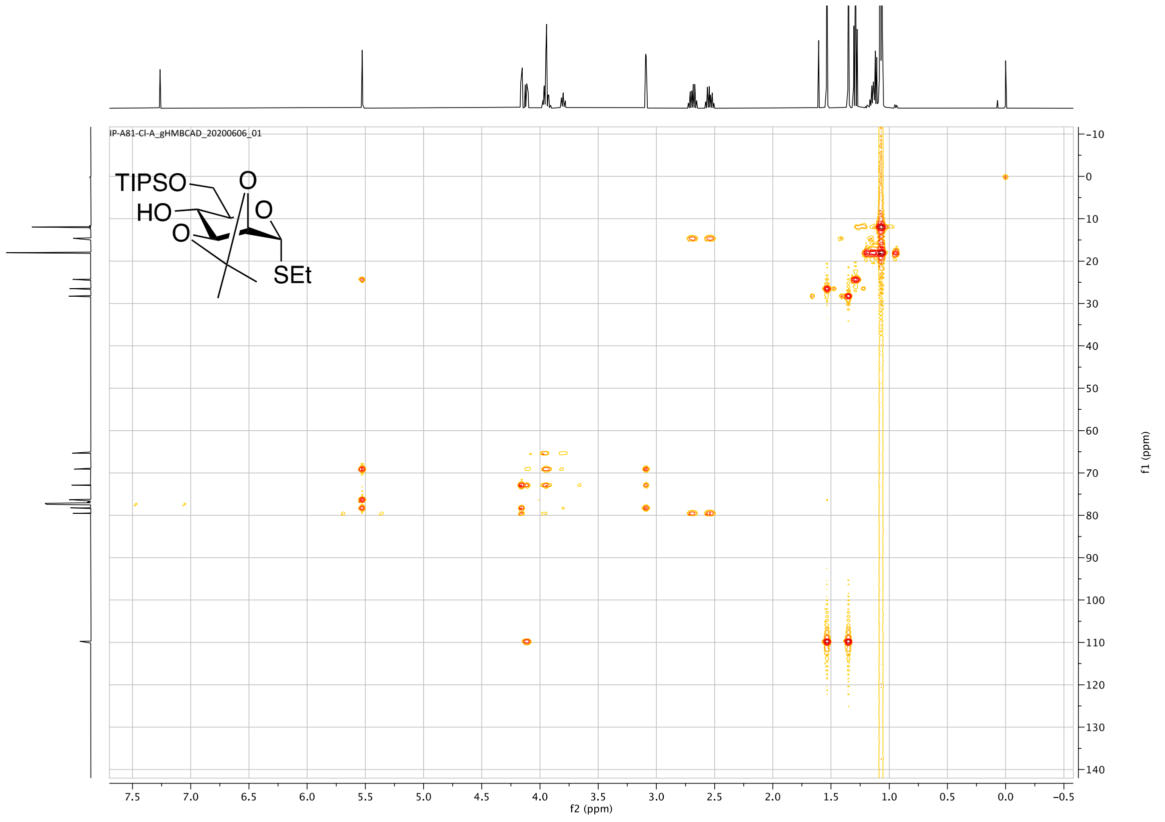

^{1}H NMR (500 MHz, Chloroform-*d*) S11

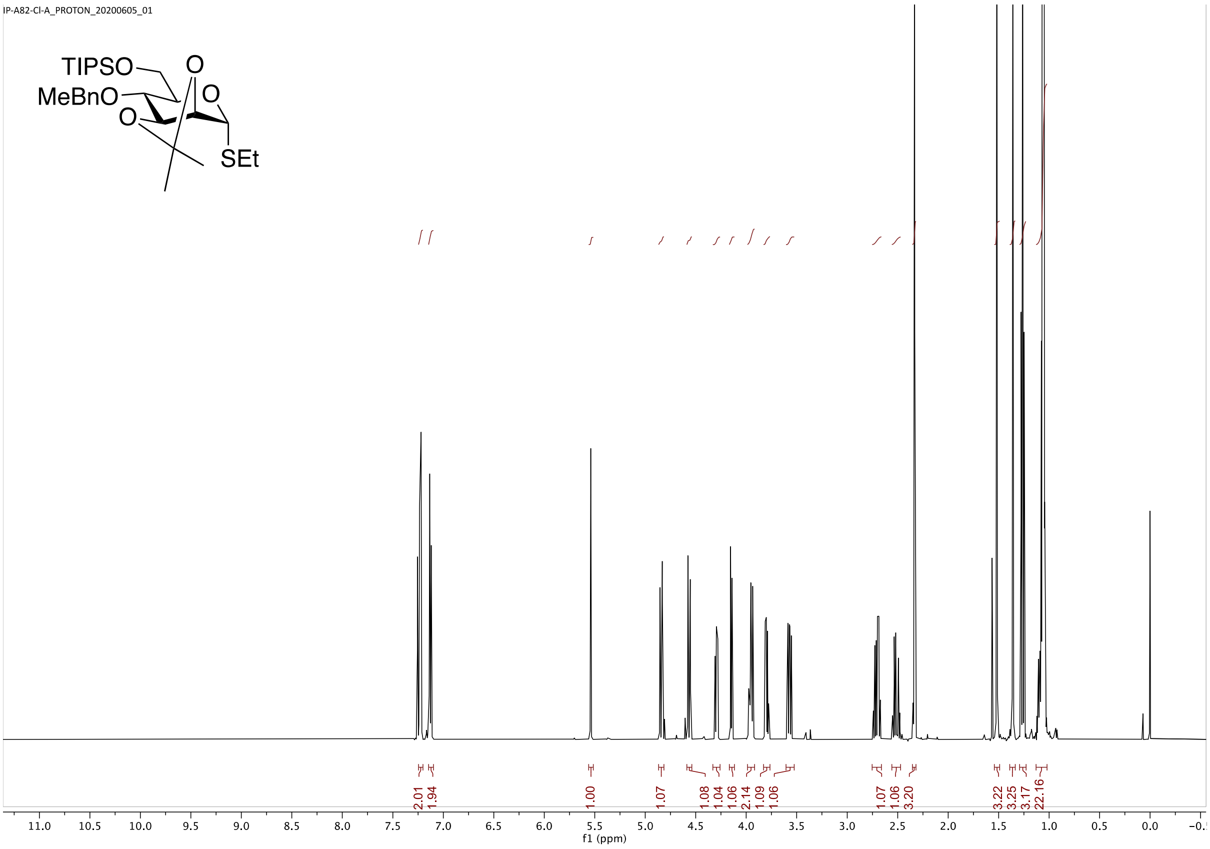

^{13}C NMR (126 MHz, Chloroform-*d*) S11

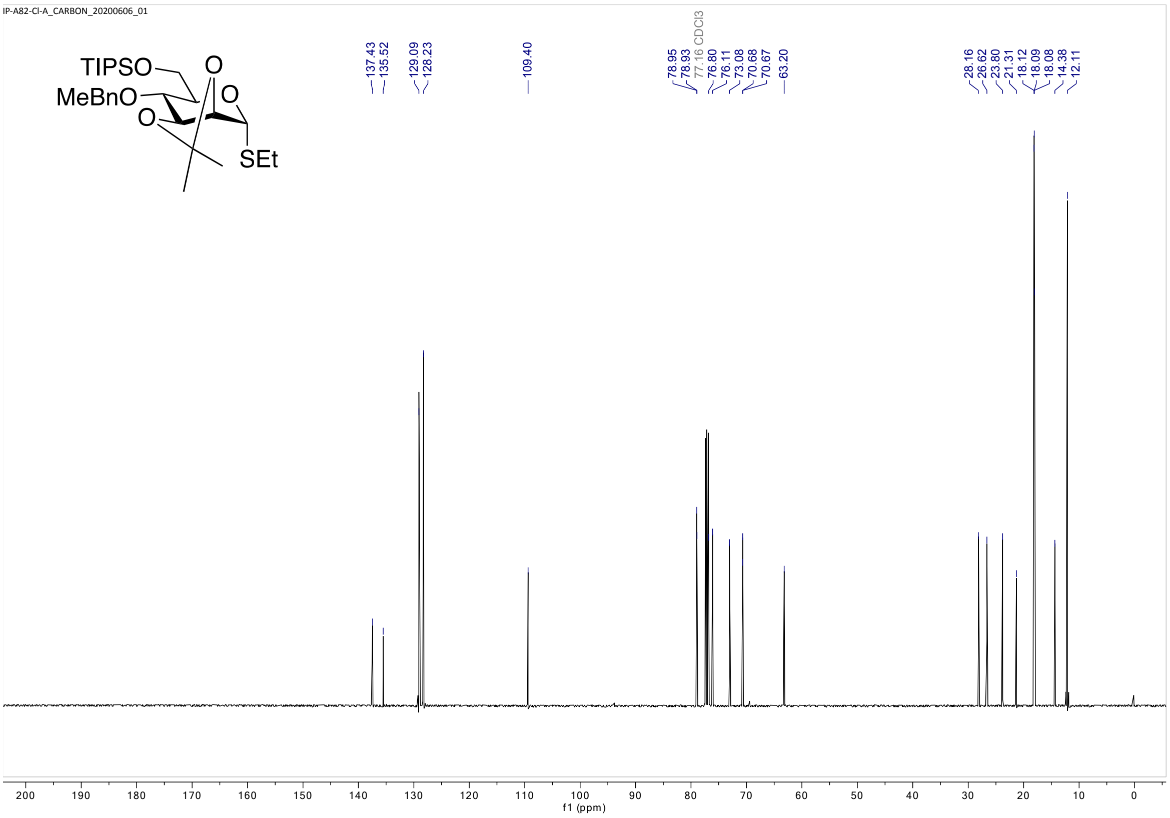

COSY NMR (500 MHz, Chloroform-*d*) S11

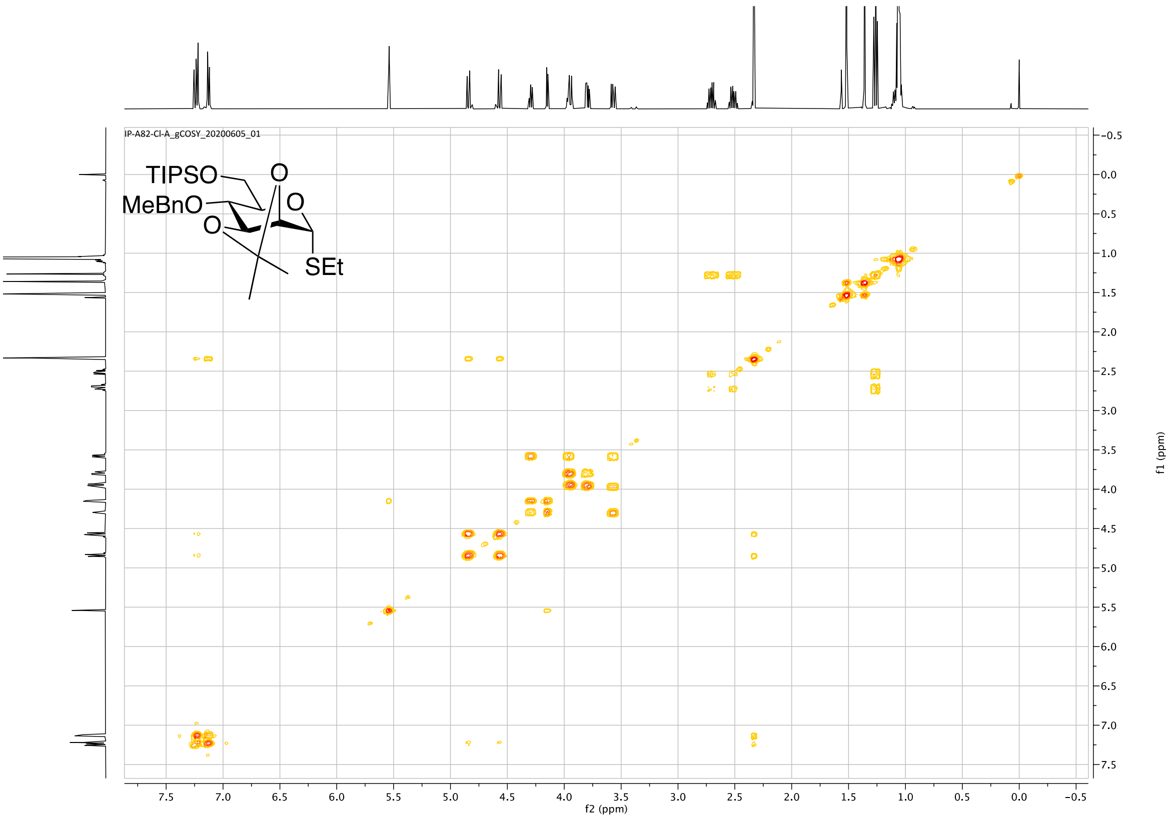

HSQC NMR (500 MHz x 126 MHz, Chloroform-*d*) S11

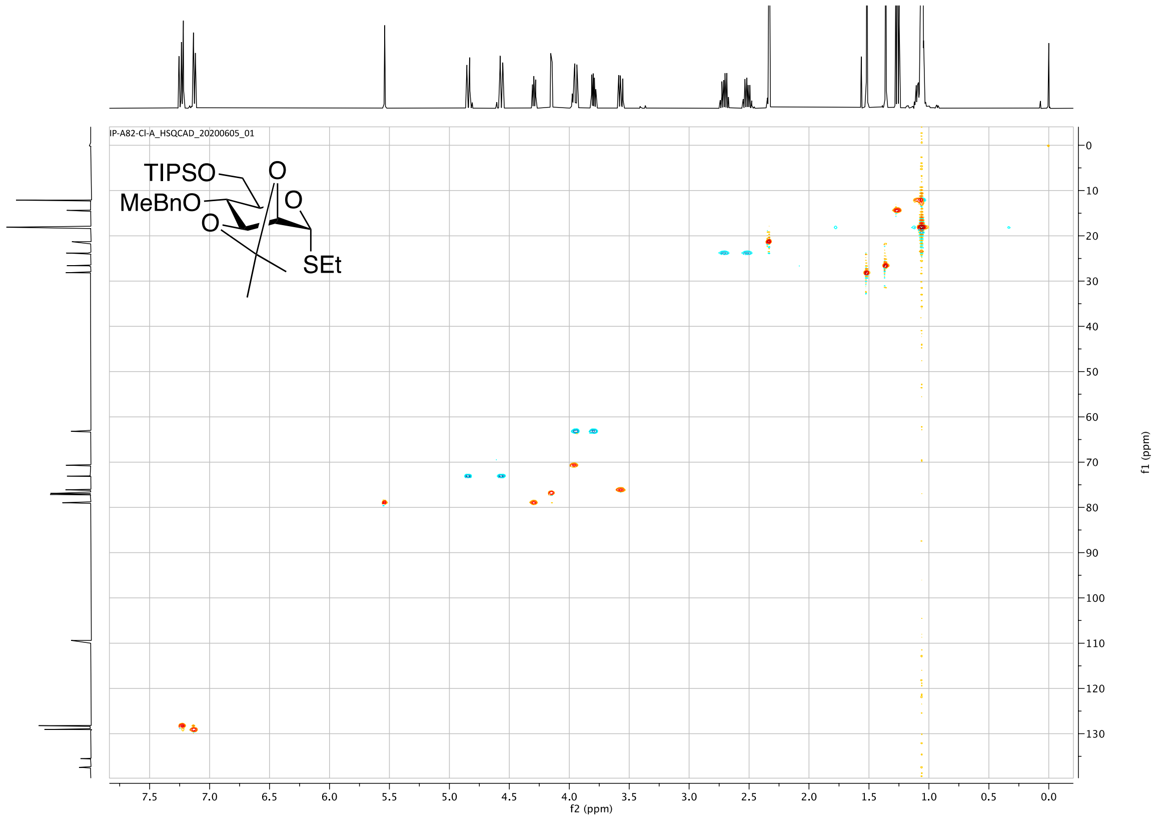

HMBC NMR (500 MHz x 126 MHz, Chloroform-*d*) S11

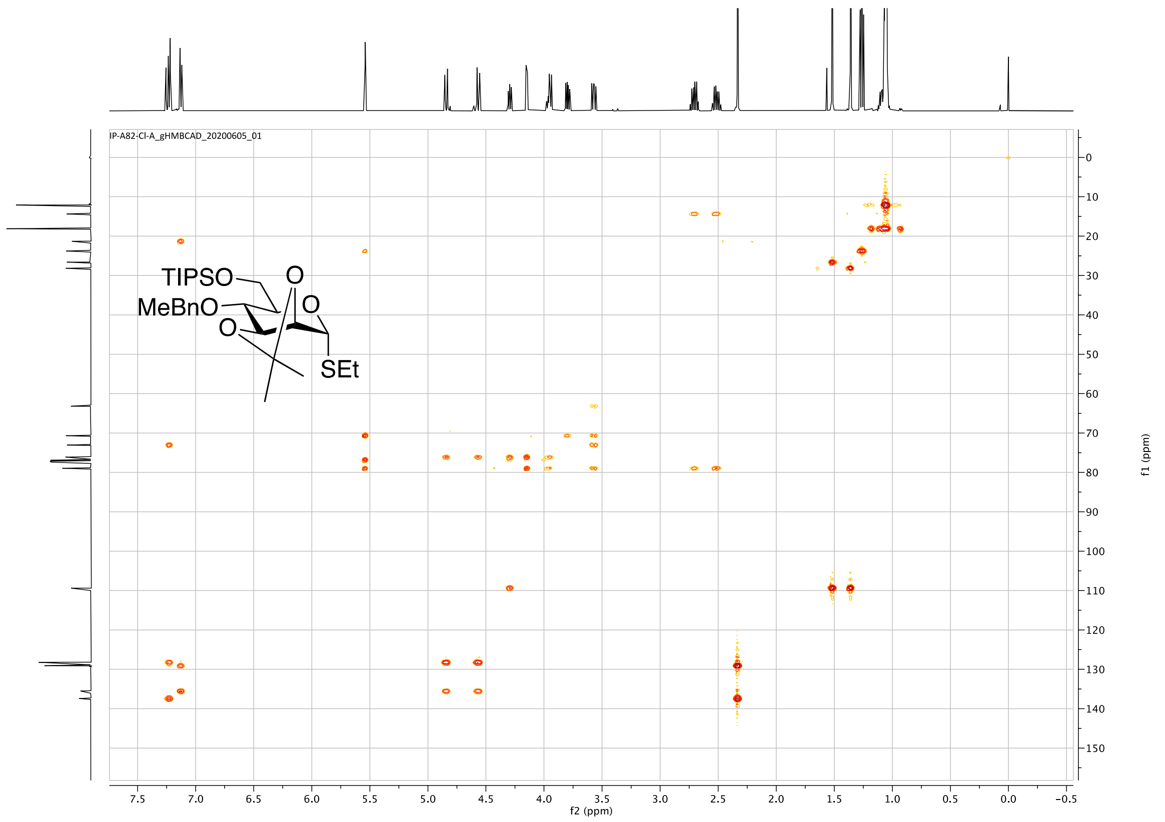

^{1}H NMR (400 MHz, Chloroform-*d*) S12

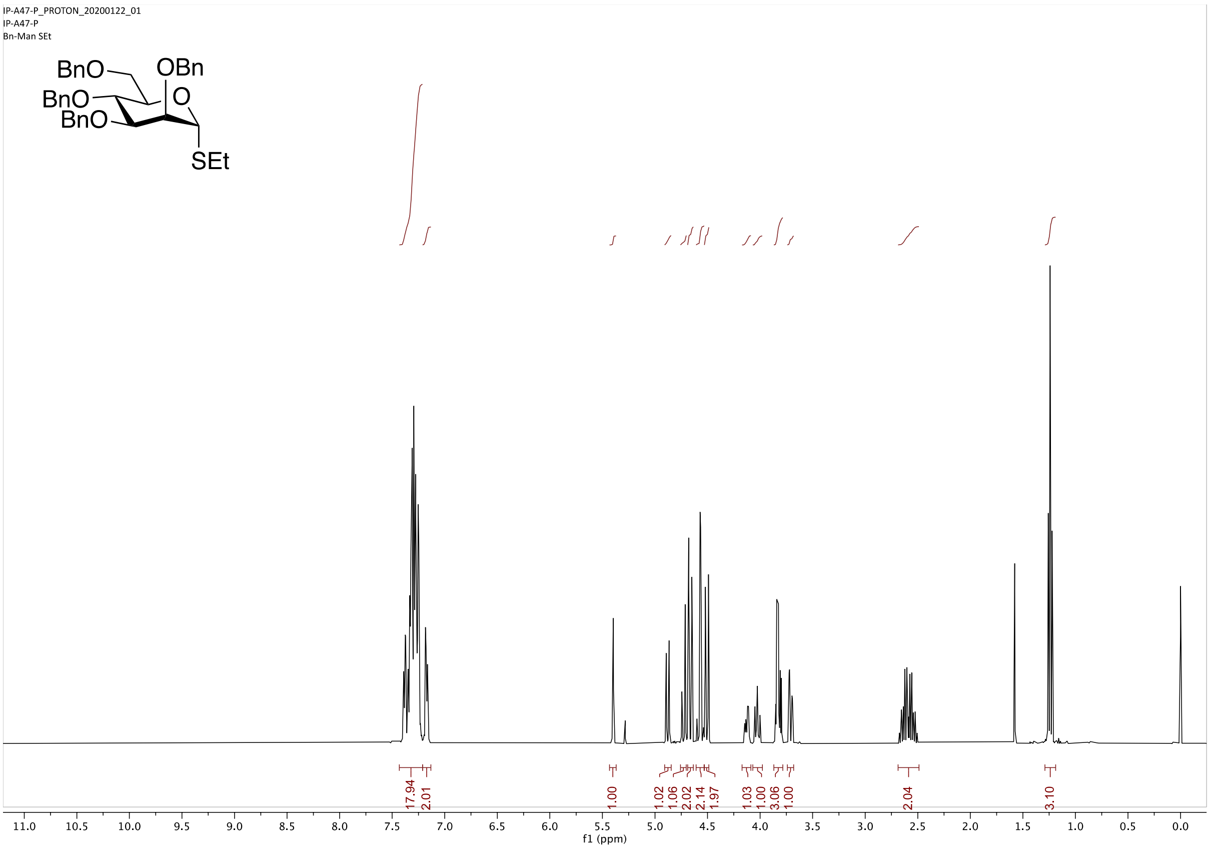

^{13}C NMR (126 MHz, Chloroform-*d*) S12

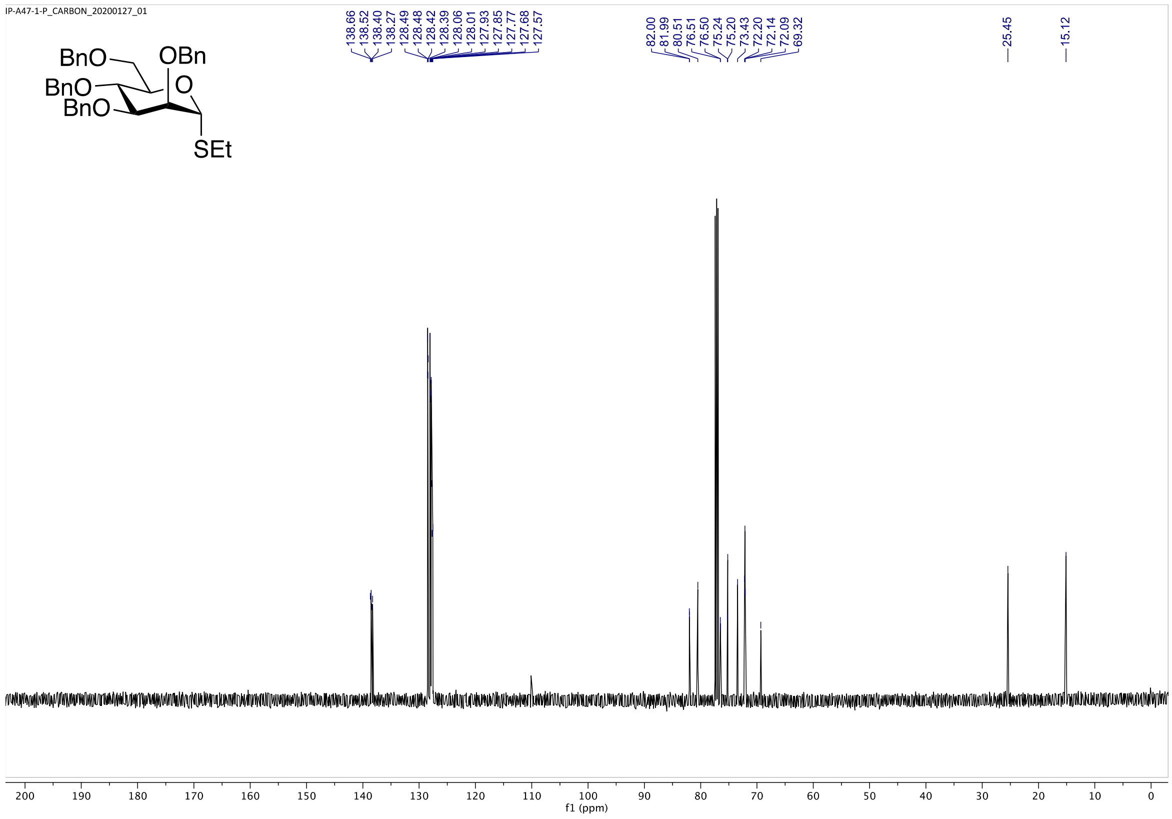

^{1}H NMR (500 MHz, Chloroform-*d*) S19

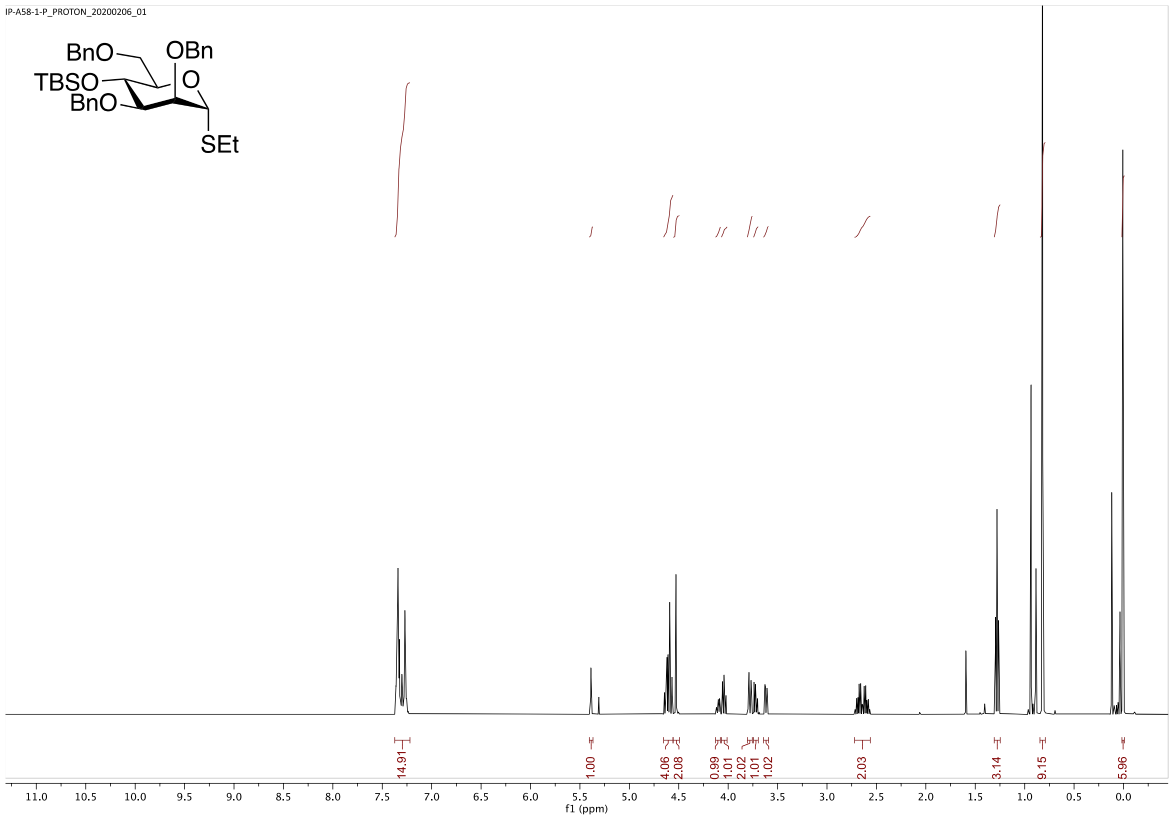

^{13}C NMR (126 MHz, Chloroform-*d*) S19

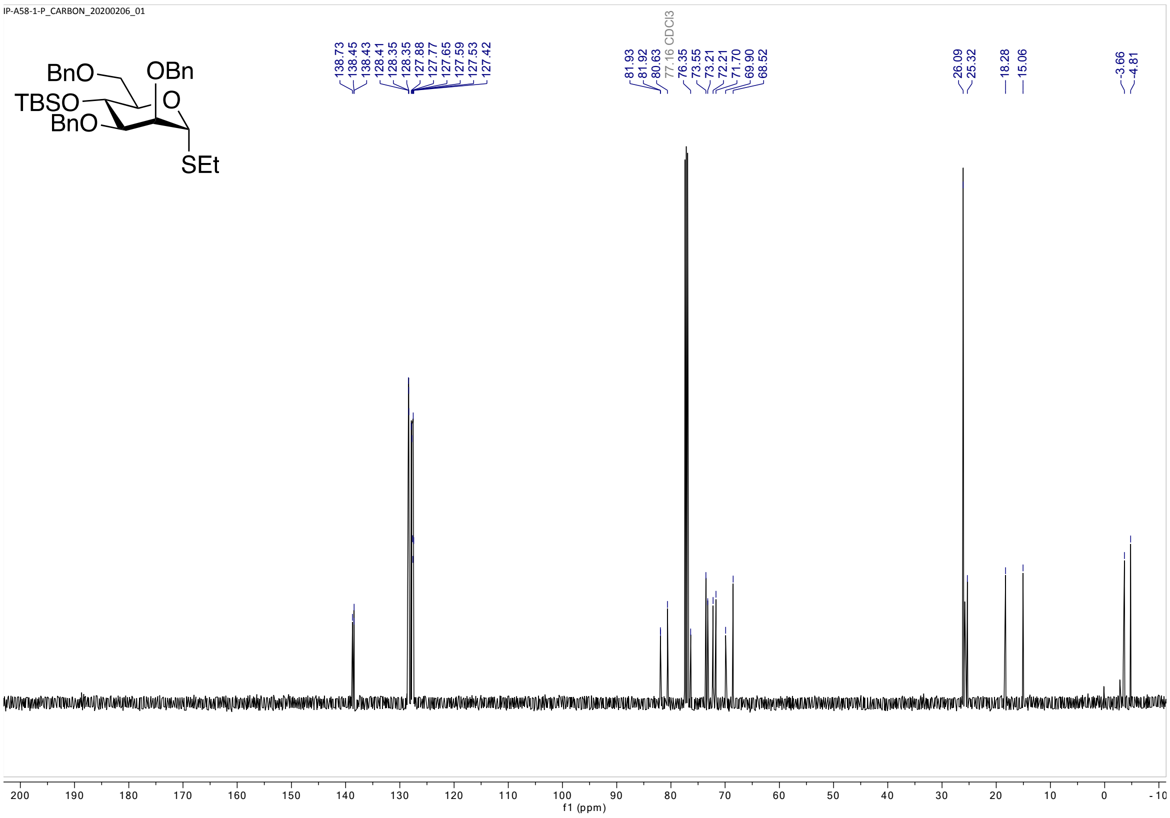

COSY NMR (500 MHz, Chloroform-*d*) S19

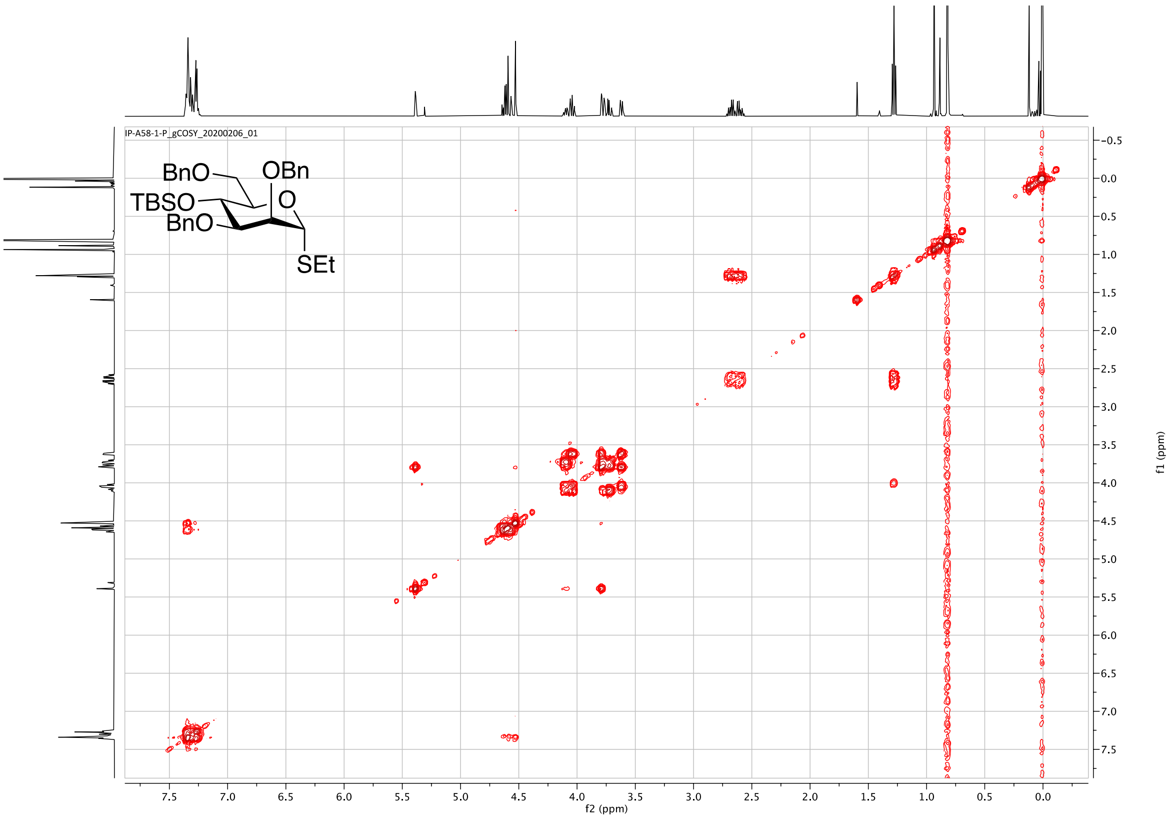

HSQC NMR (500 MHz x 126 MHz, Chloroform-*d*) S19

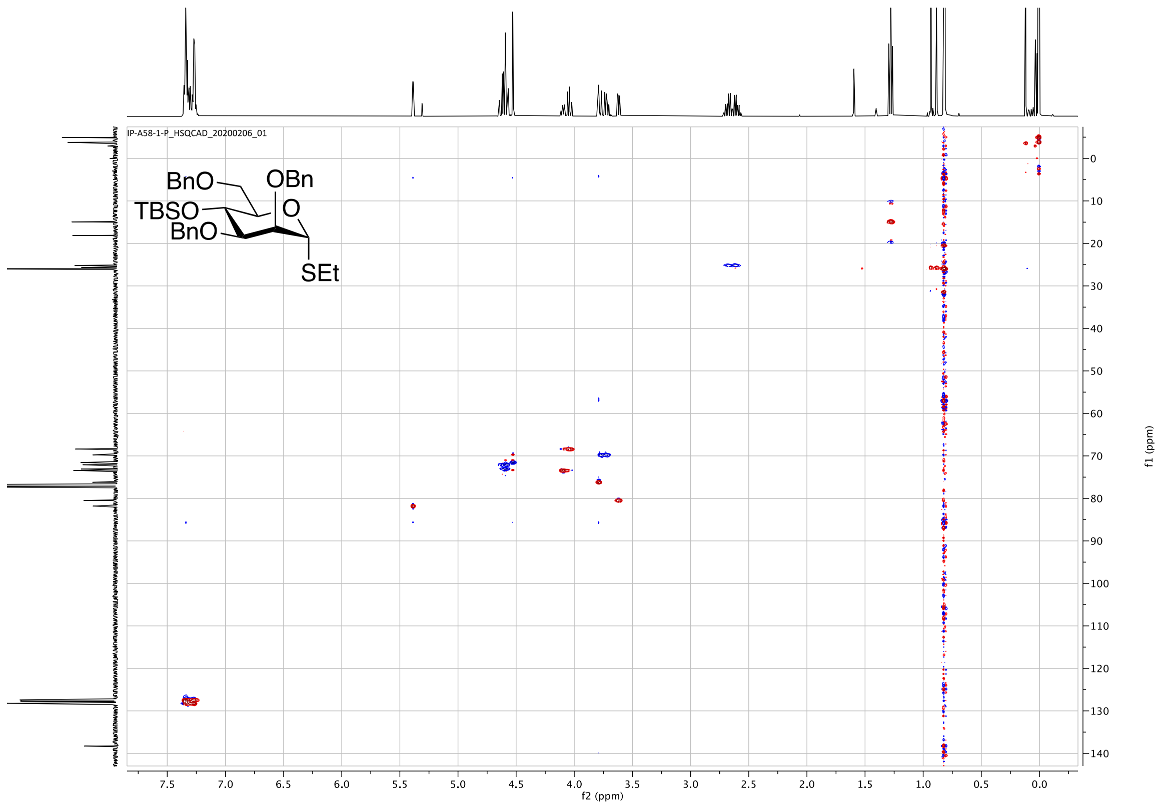

HMBC NMR (500 MHz x 126 MHz, Chloroform-*d*) S19

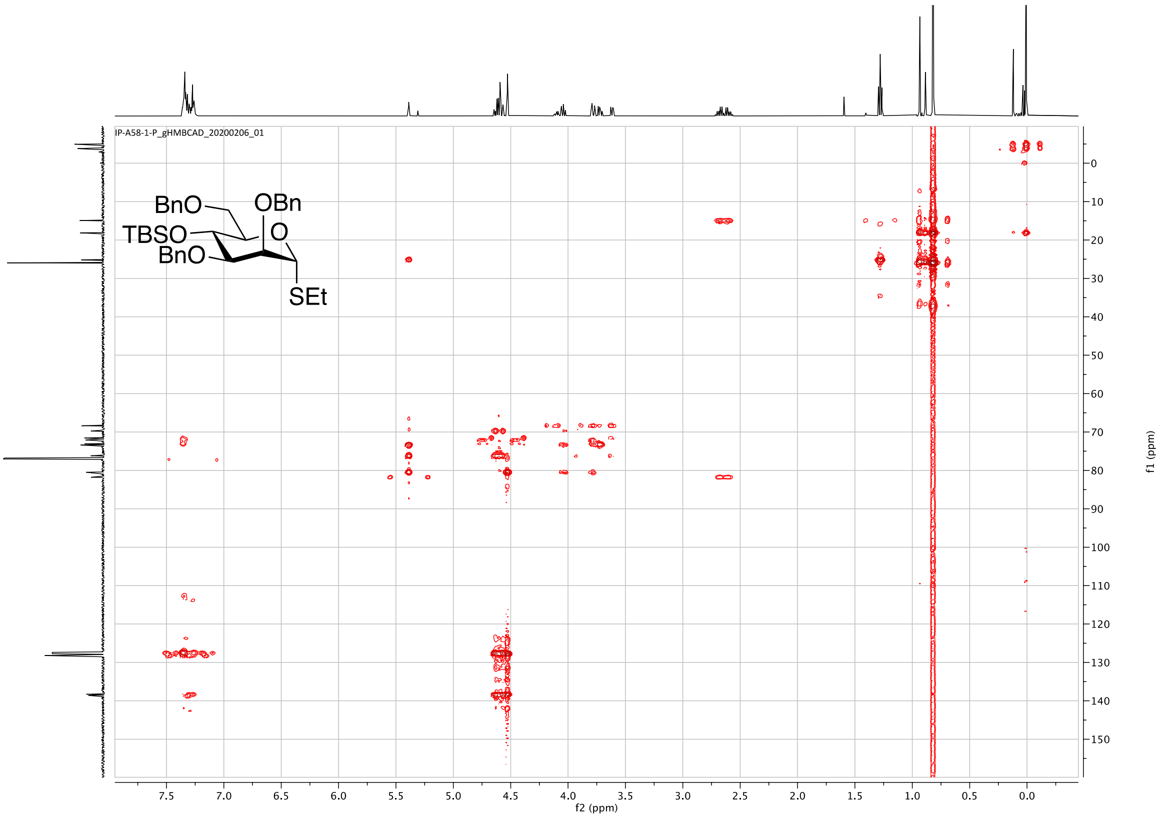

^{1}H NMR (500 MHz, Chloroform-*d*) S21

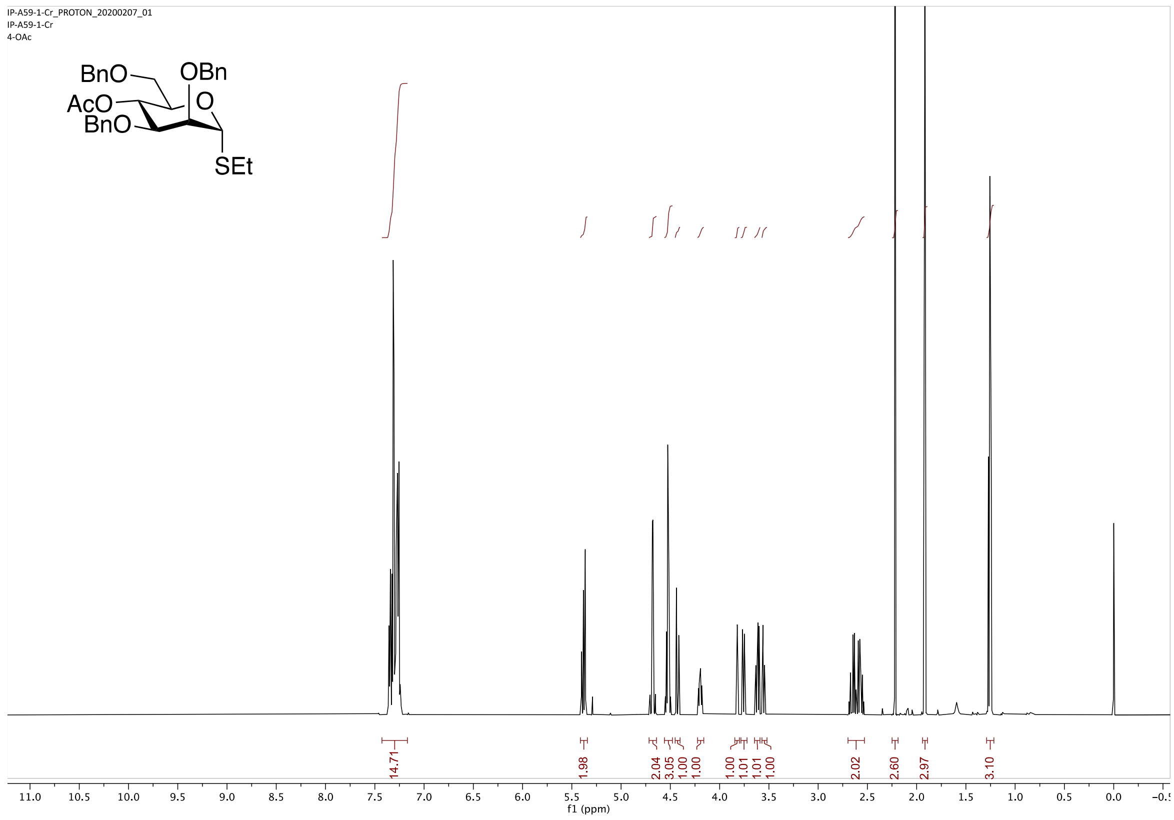

^{13}C NMR (126 MHz, Chloroform-*d*) S21

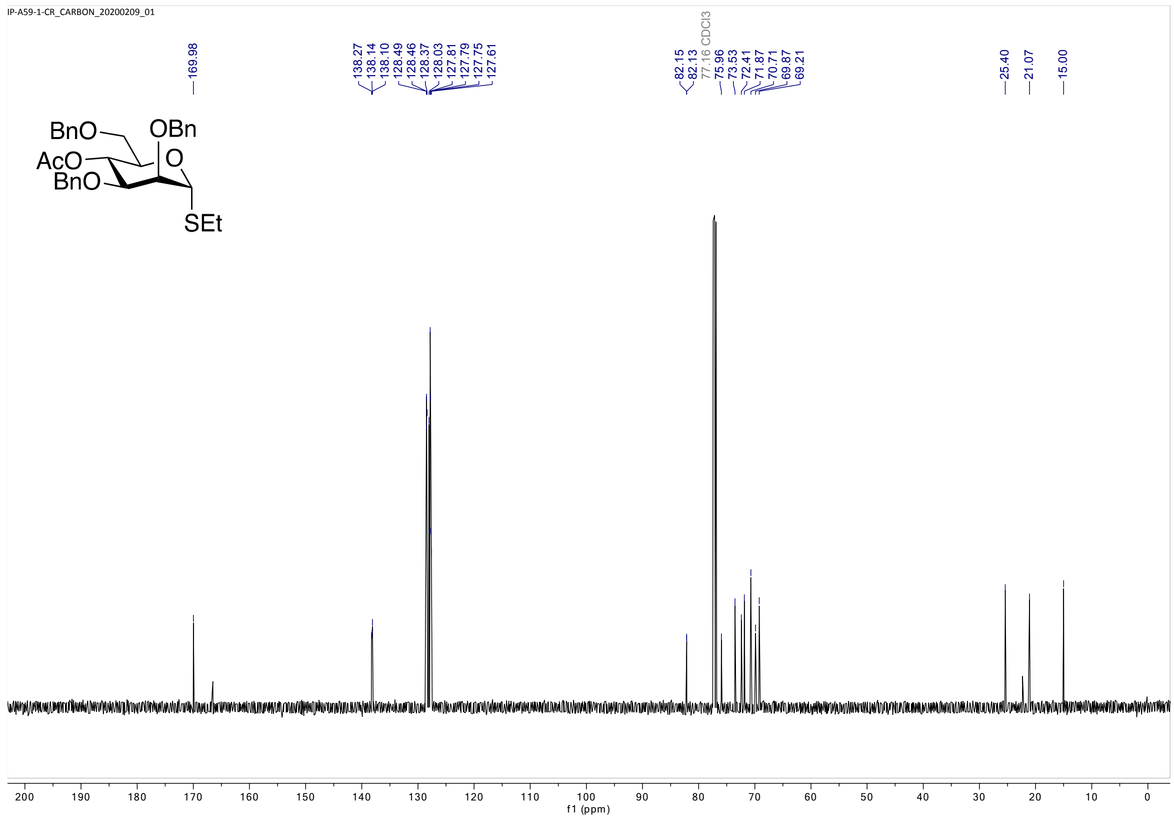

^{1}H NMR (500 MHz, Chloroform-*d*) 1a

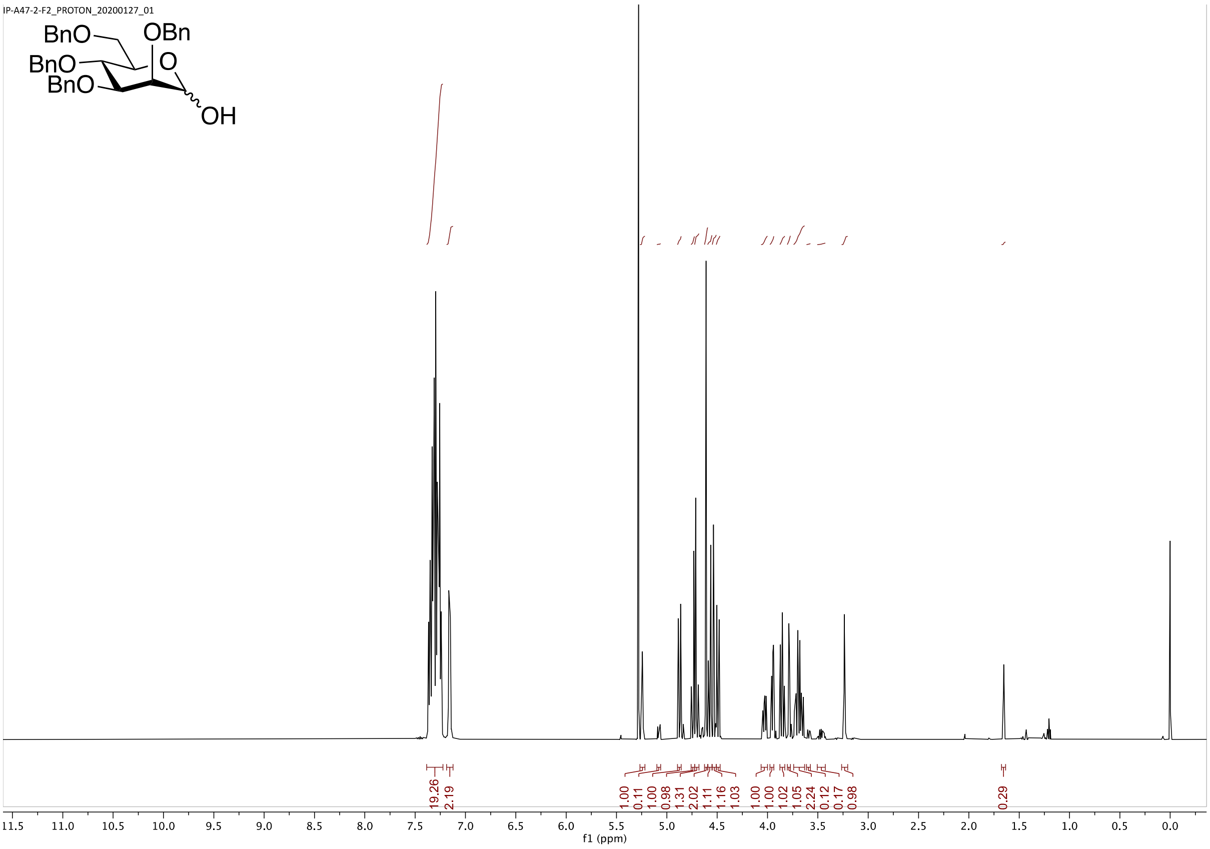

^{1}H NMR (500 MHz, Chloroform-*d*) 8a

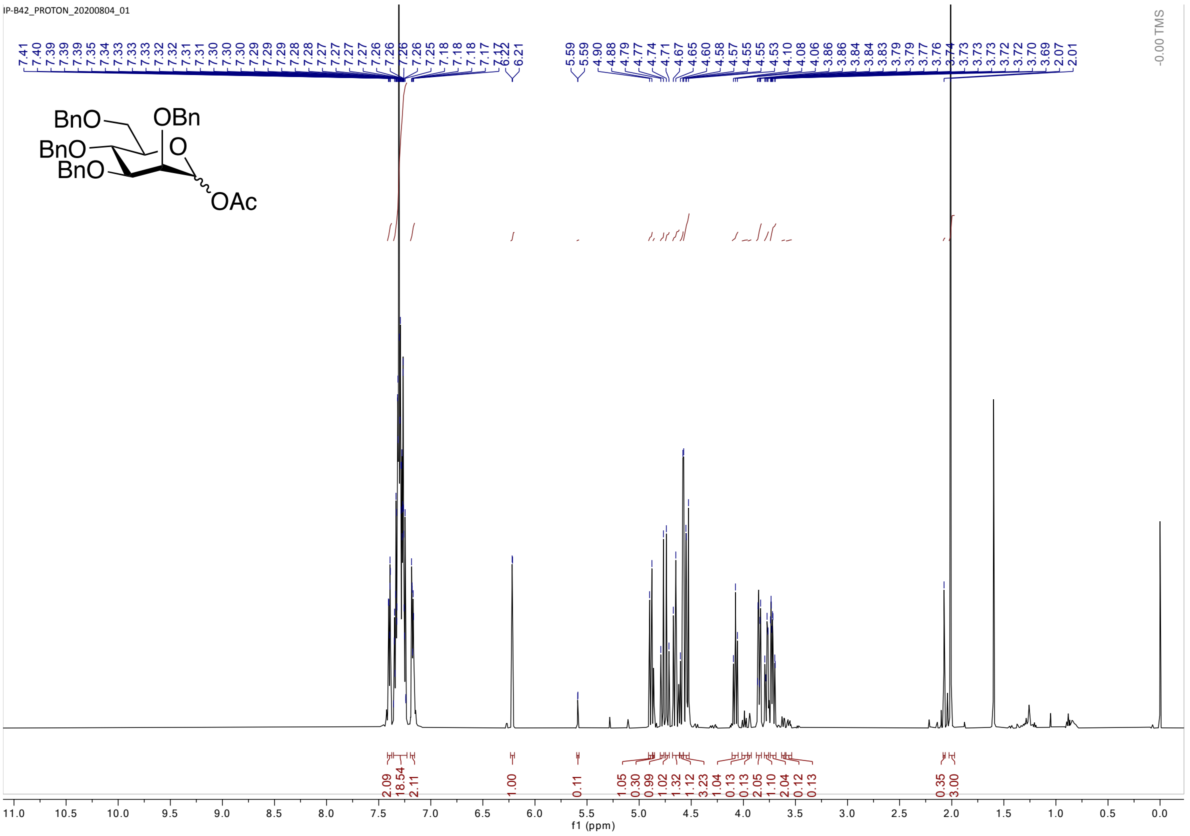

^{13}C NMR (126 MHz, Chloroform-*d*) 8a

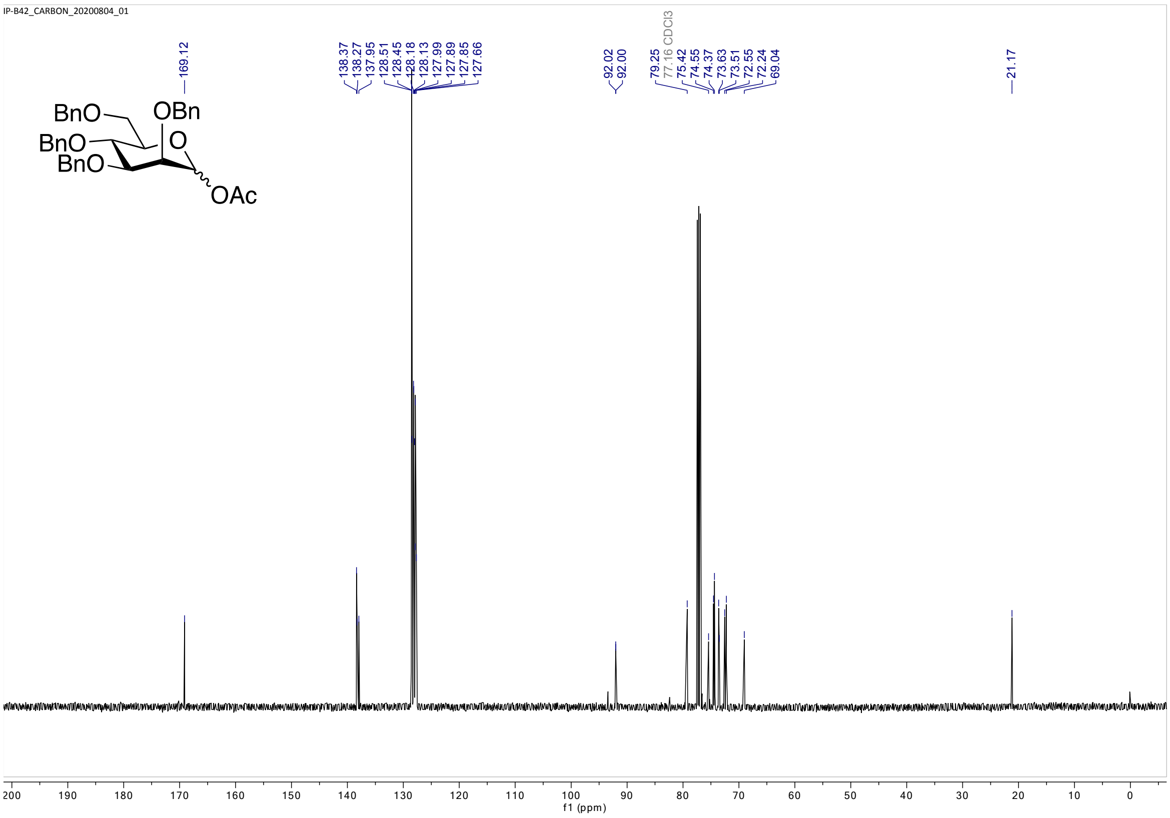

^{1}H NMR (600 MHz, Chloroform-*d*) 1b

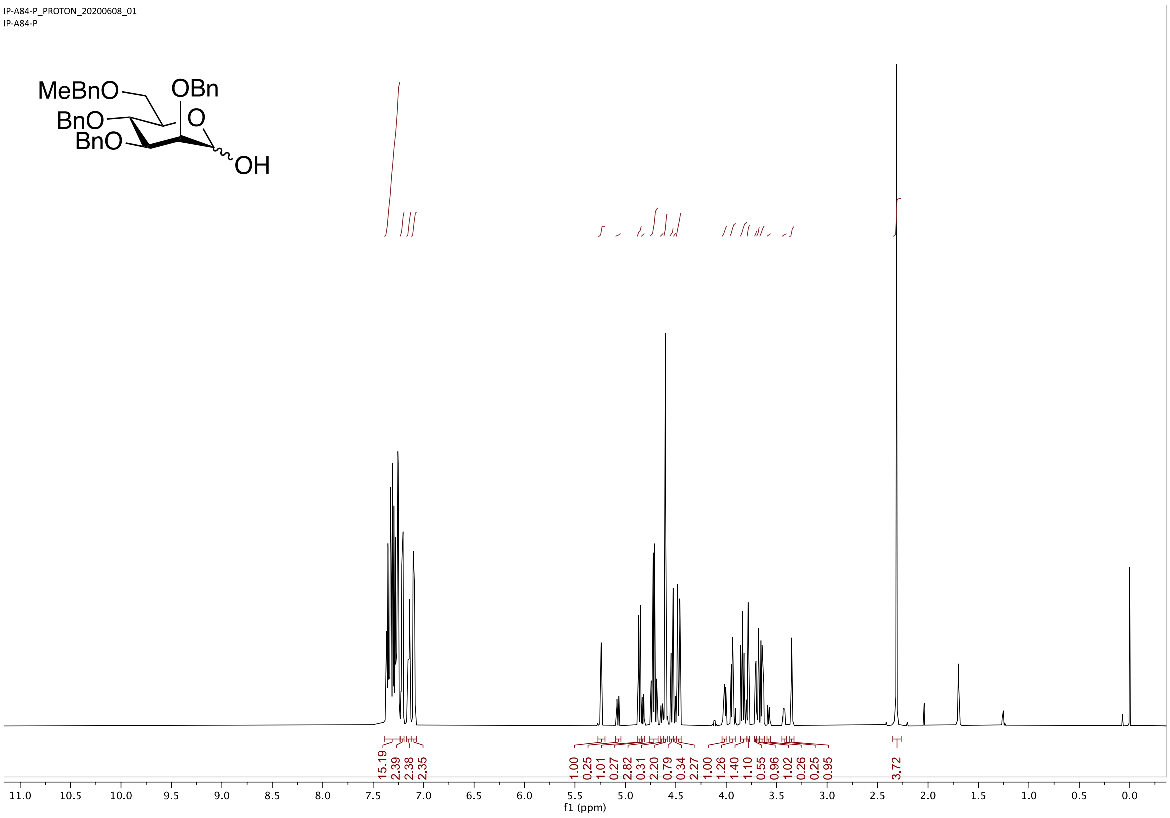

^{13}C NMR (151 MHz, Chloroform-*d*) 1b

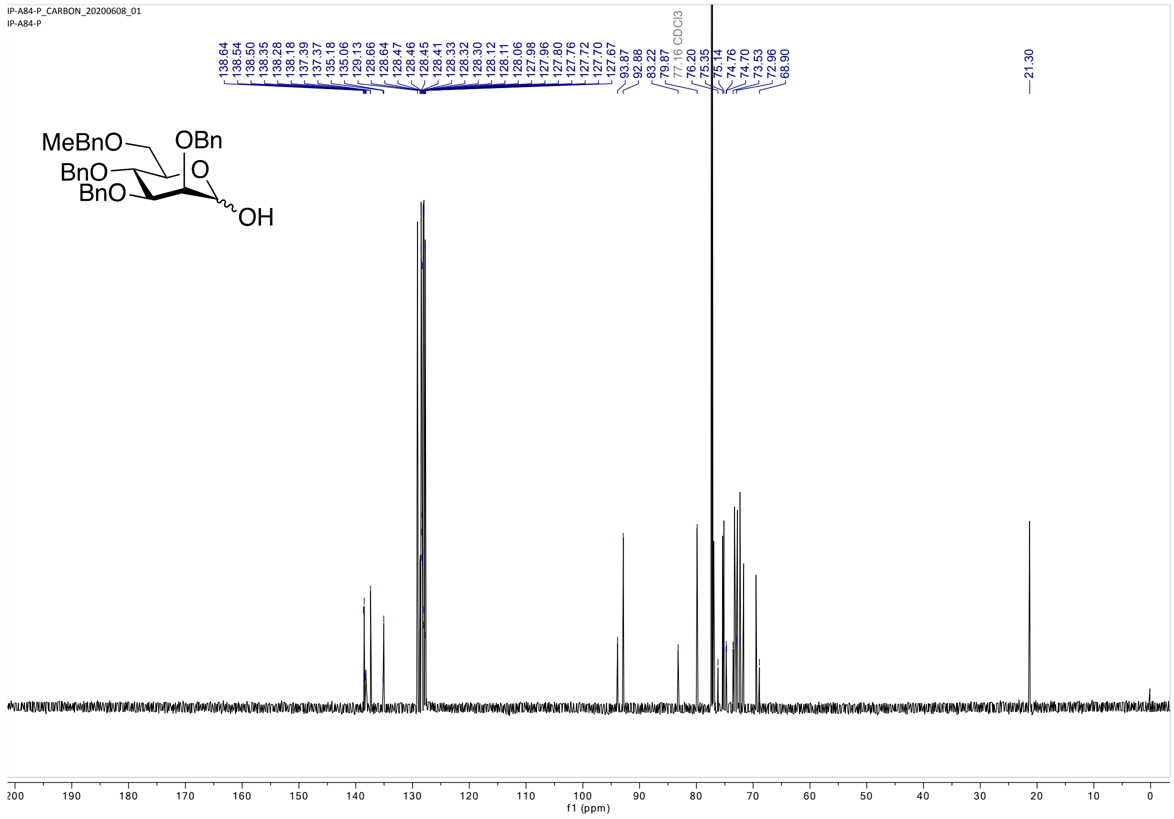

COSY NMR (600 MHz, Chloroform-*d*) 1b

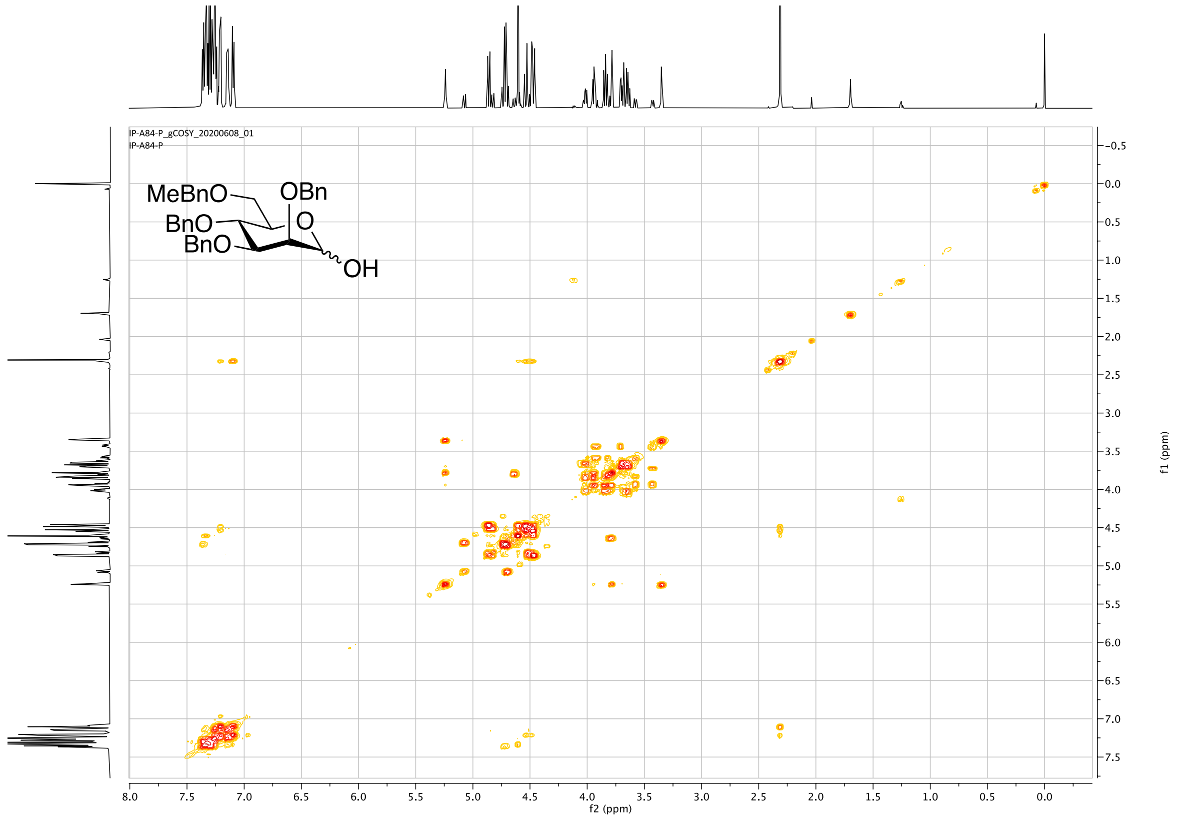

HSQC NMR (600 MHz x 151 MHz, Chloroform-*d*) 1b

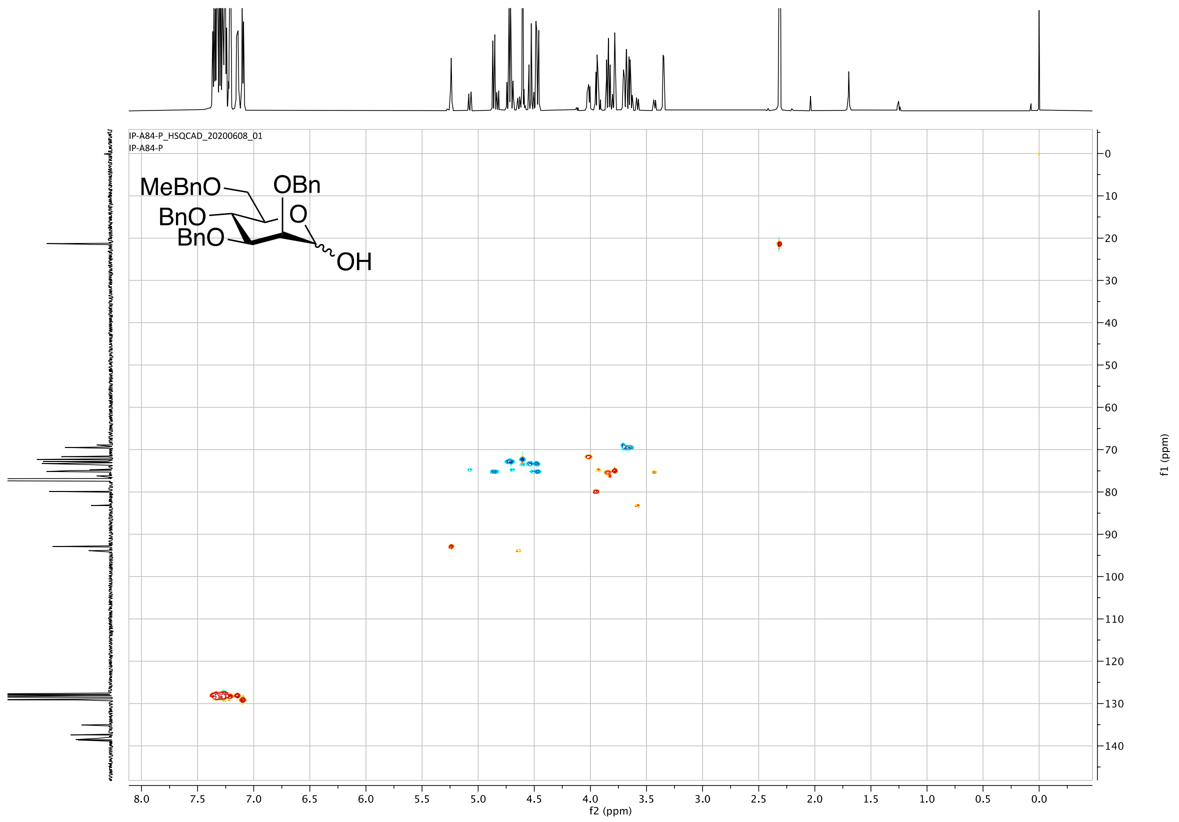

HMBC NMR (600 MHz x 151 MHz, Chloroform-*d*) 1b

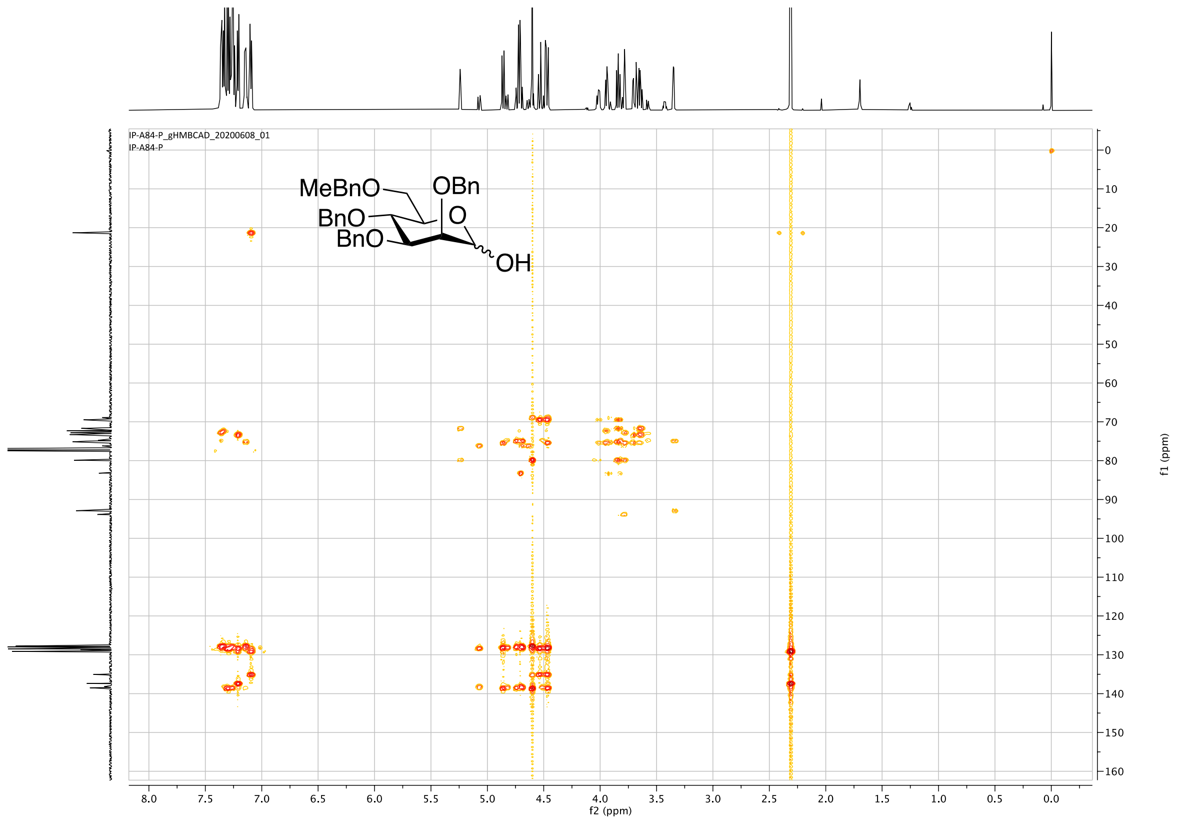

^{1}H NMR (500 MHz, Chloroform-*d*) 1c

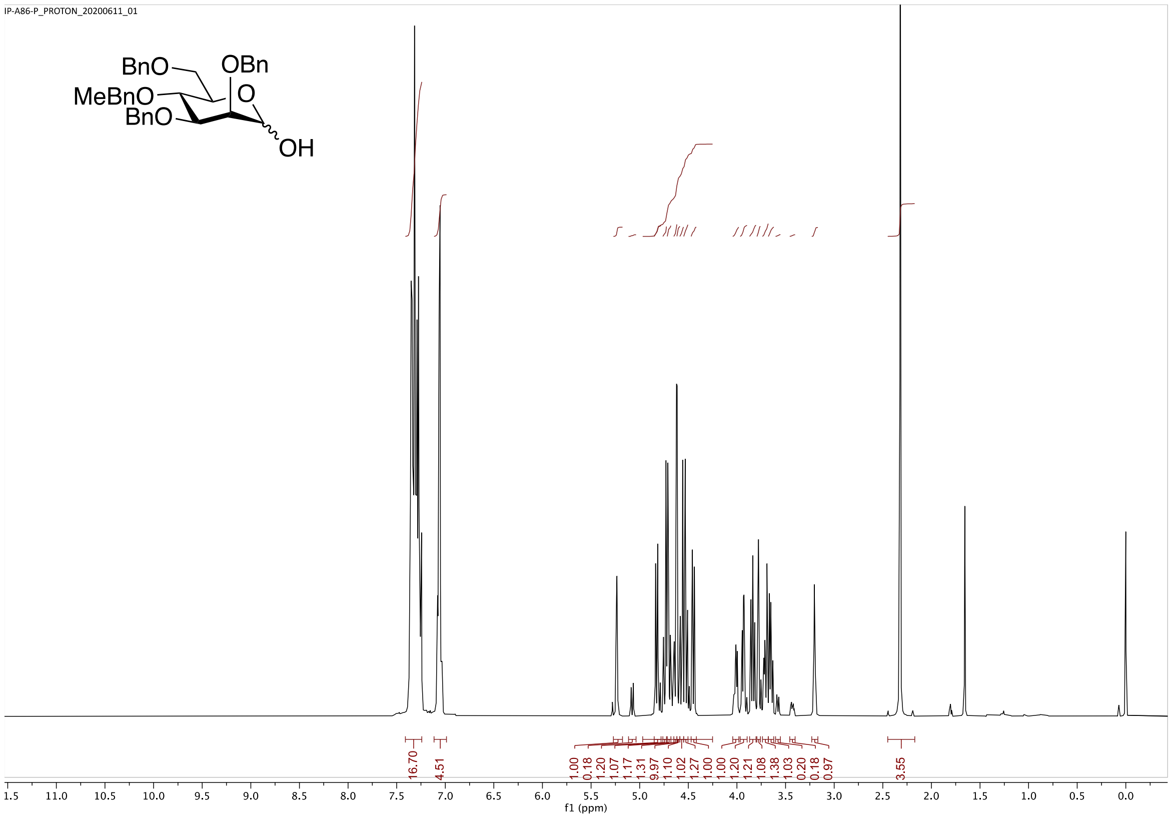

^{13}C NMR (126 MHz, Chloroform-*d*) 1c

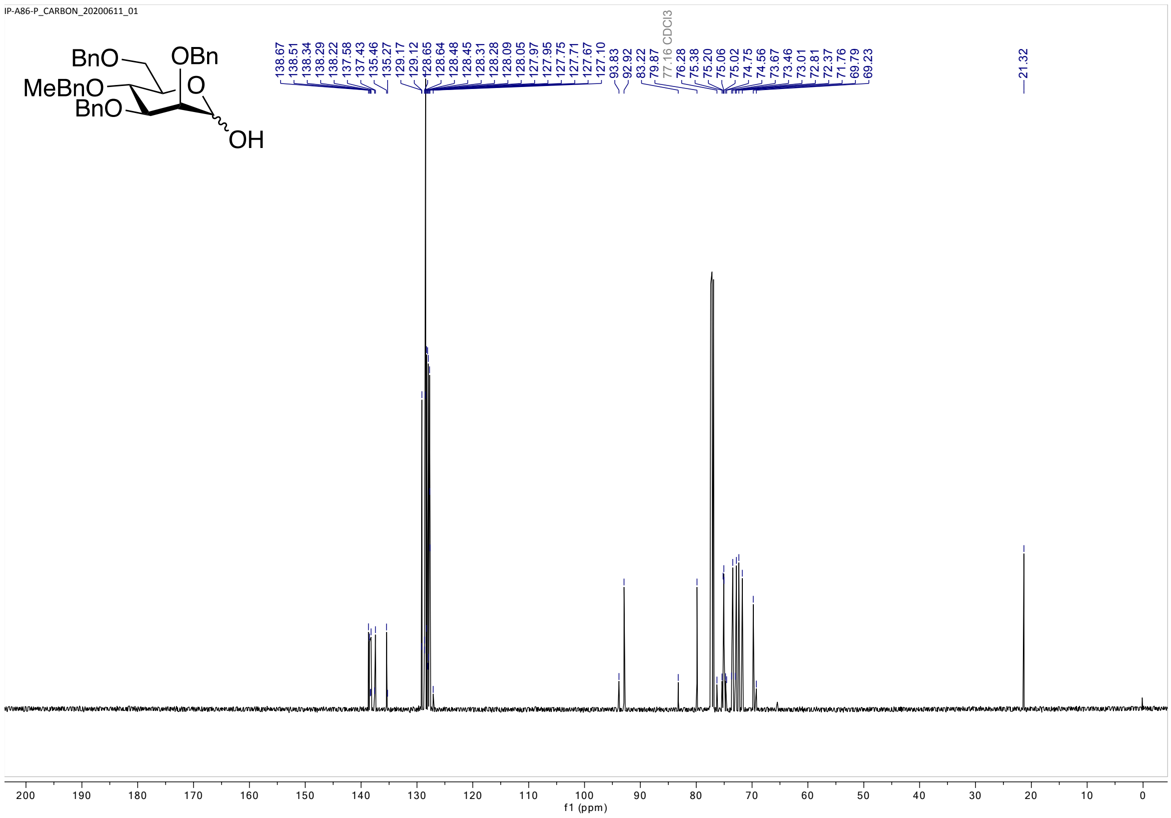

COSY NMR (500 MHz, Chloroform-*d*) 1c

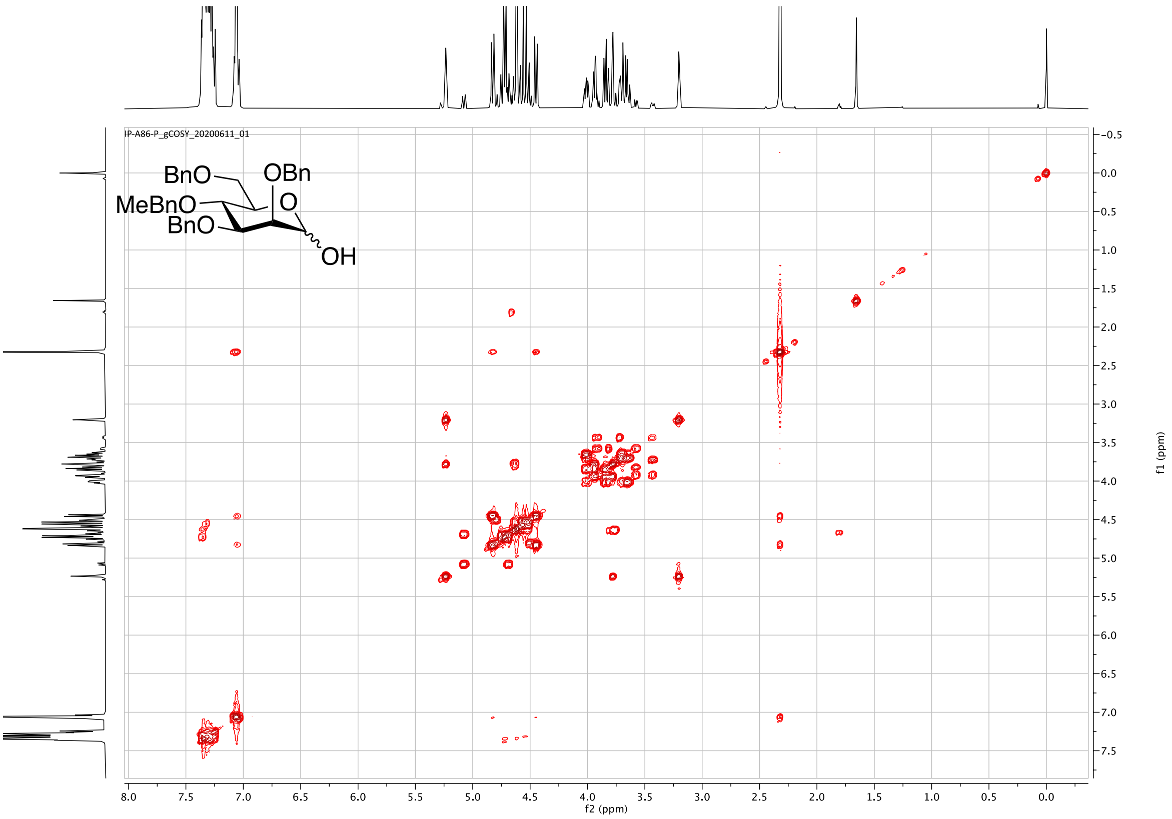

HSQC NMR (500 MHz x 126 MHz, Chloroform-*d*) 1c

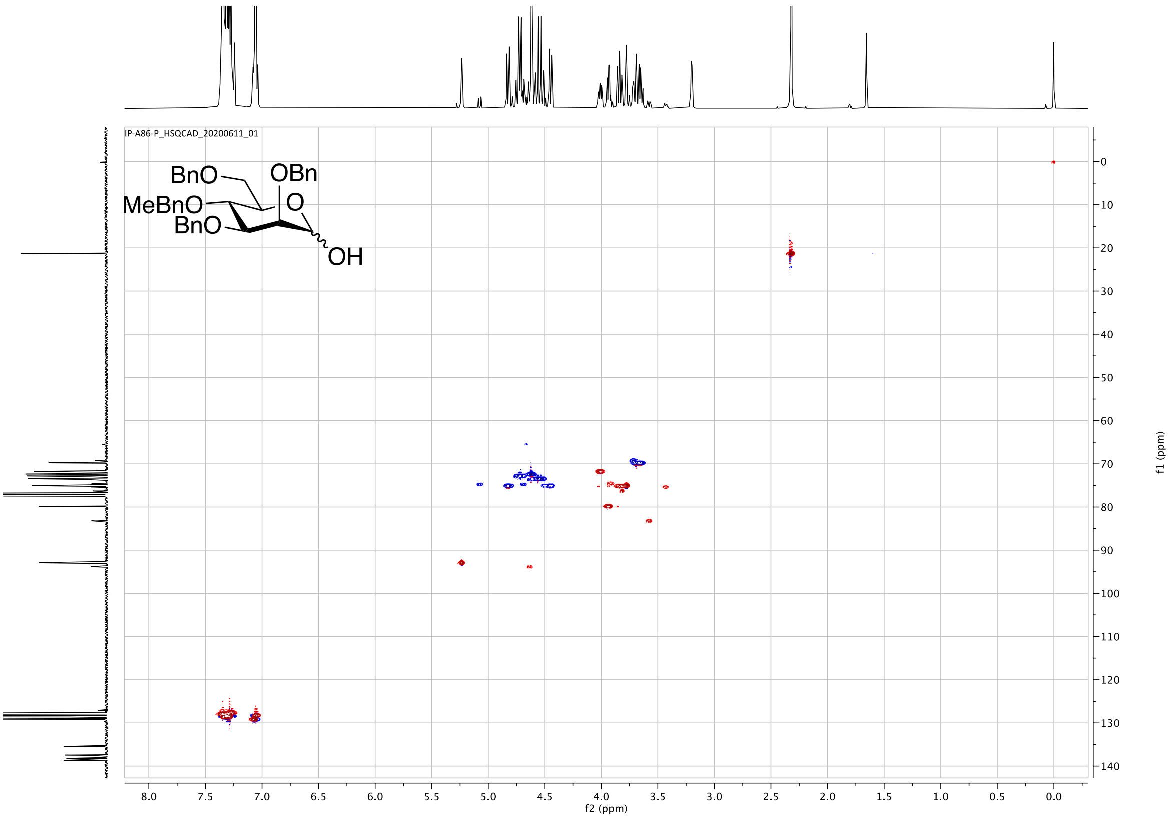

^{1}H NMR (600 MHz, Chloroform-*d*) 1d

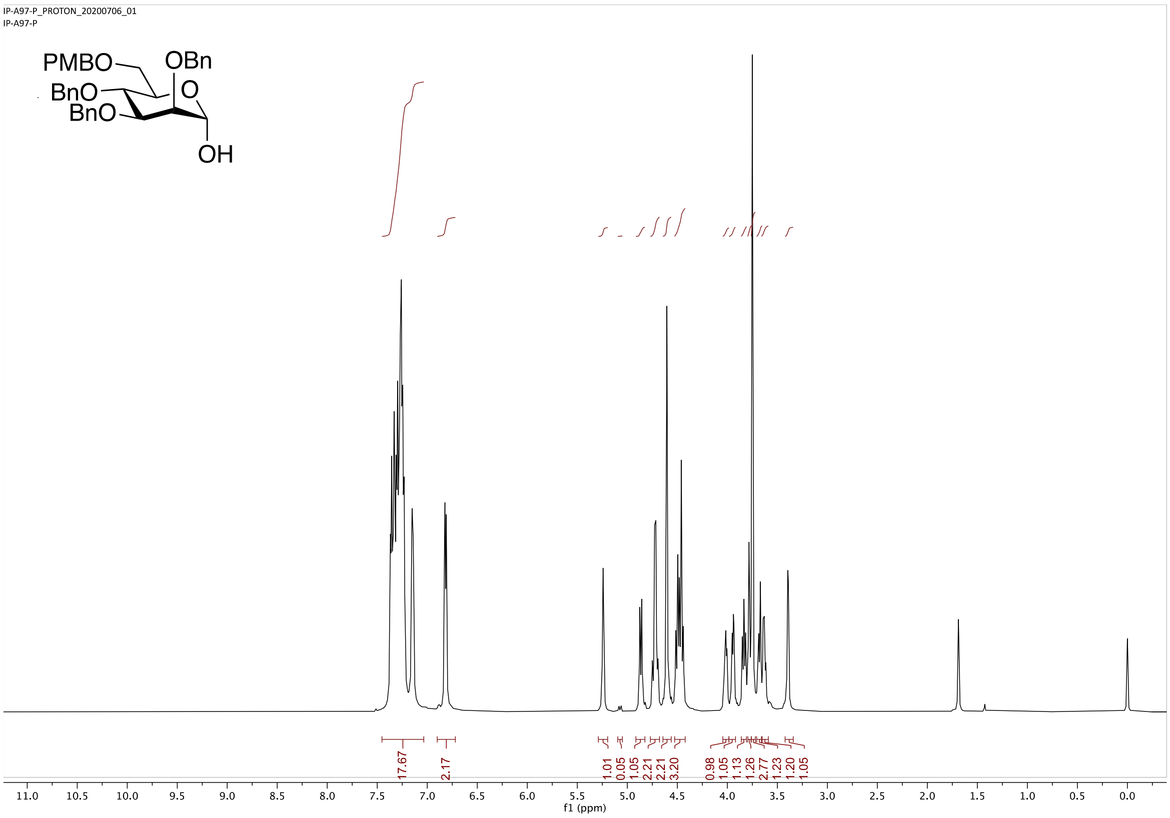

^{13}C NMR (151 MHz, Chloroform-*d*) 1d

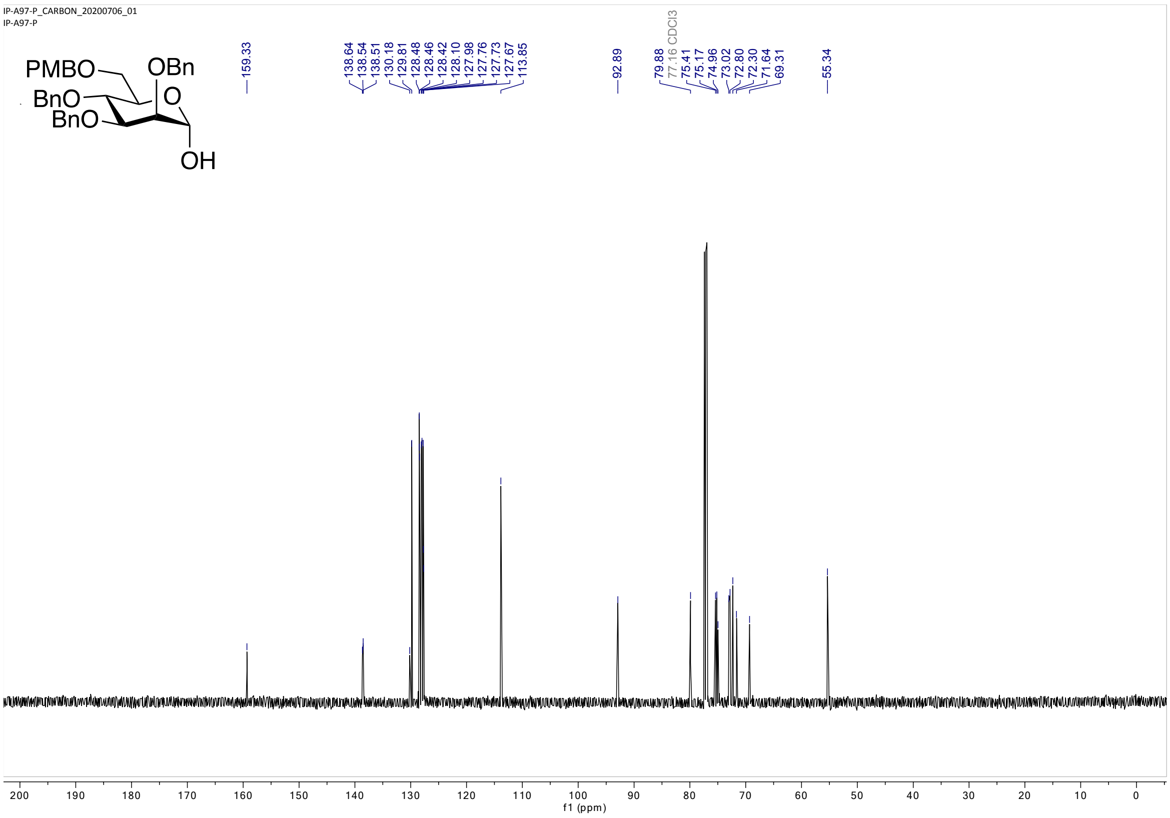

^{1}H NMR (600 MHz, Chloroform-*d*) 1e

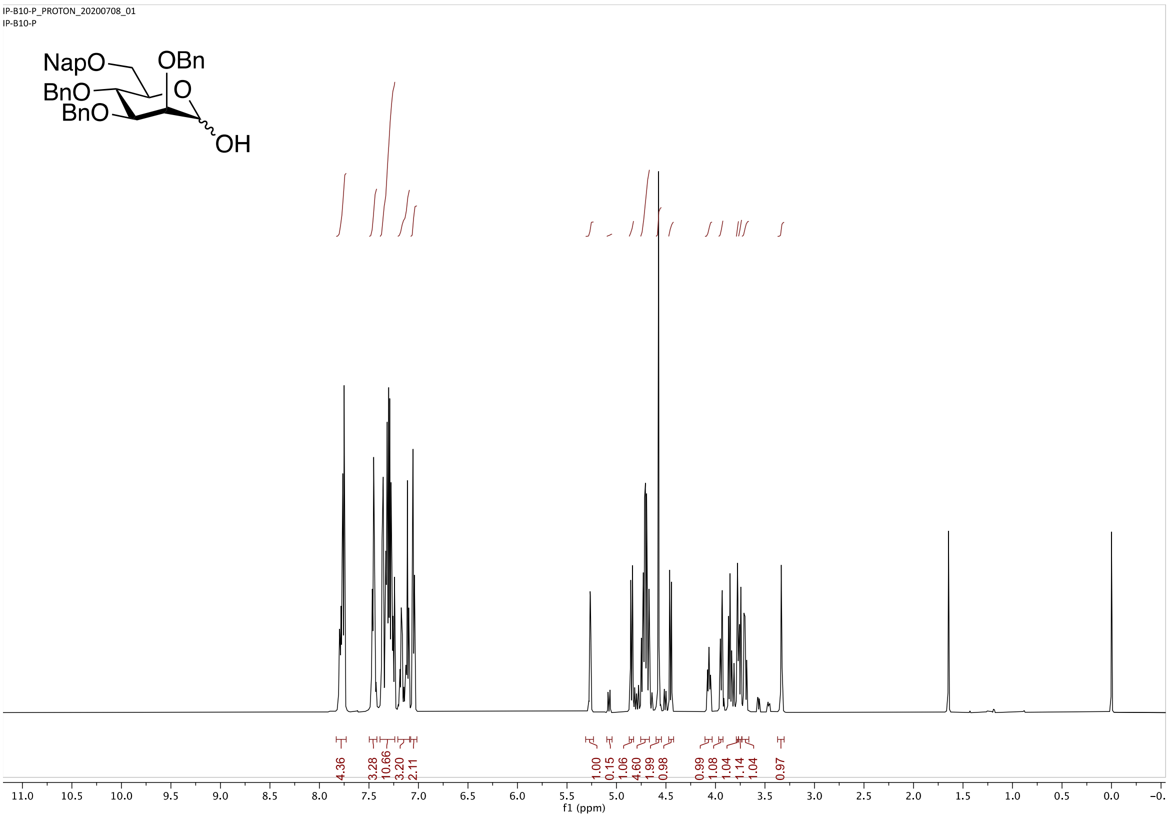

^{13}C NMR (151 MHz, Chloroform-*d*) 1e

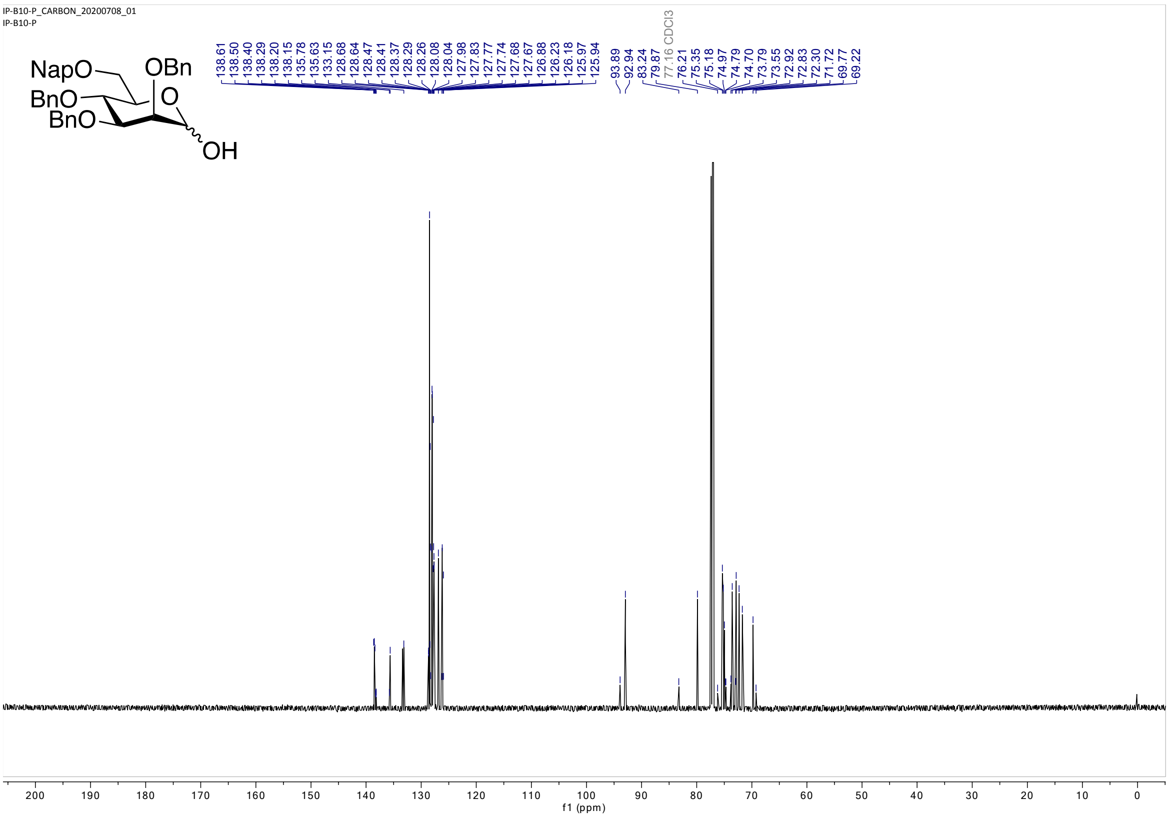

COSY NMR (600 MHz, Chloroform-*d*) 1e

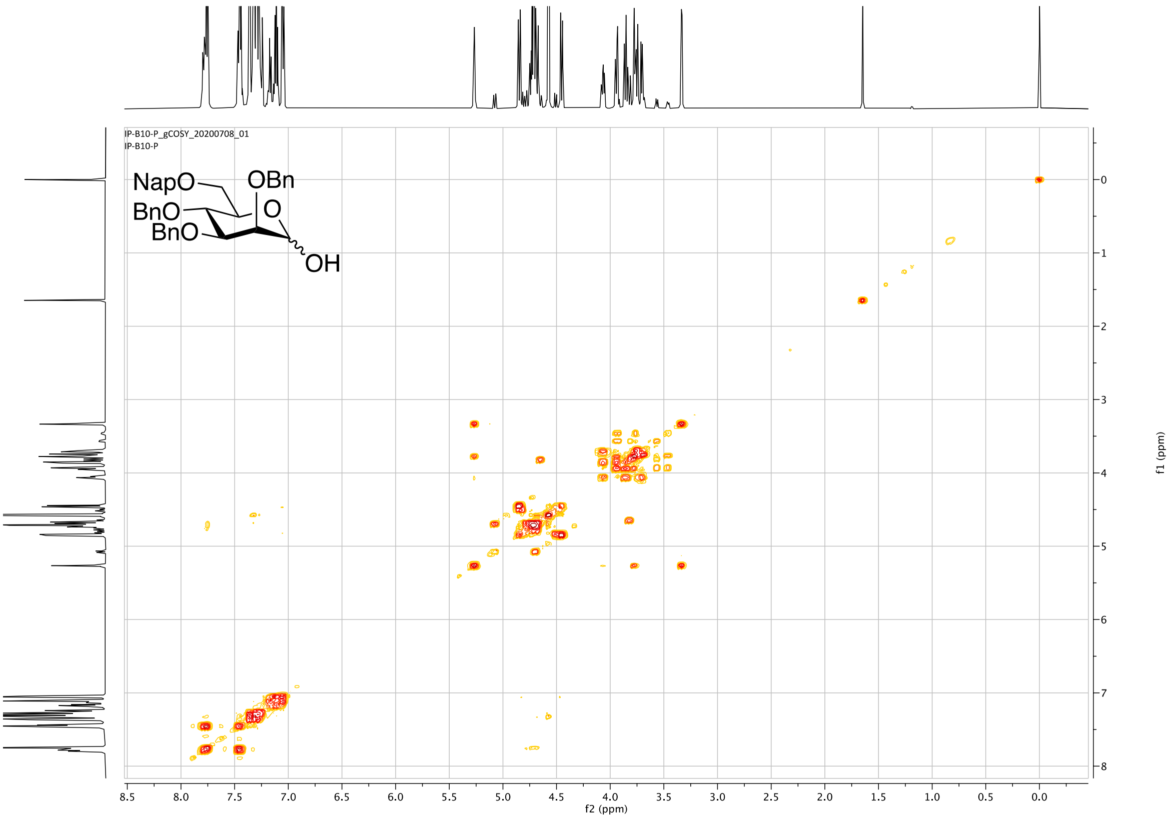

HSQC NMR (600 MHz x 151 MHz, Chloroform-*d*) 1e

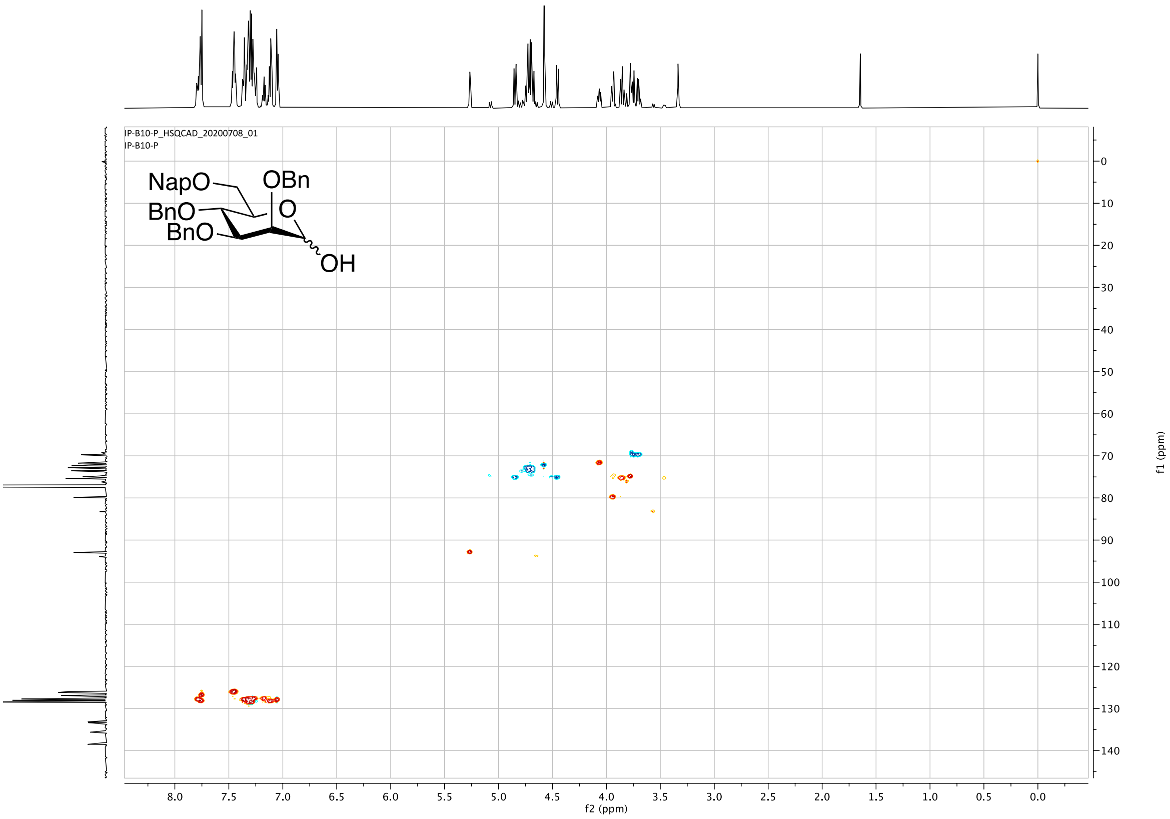

HMBC NMR (600 MHz x 151 MHz, Chloroform-*d*) 1e

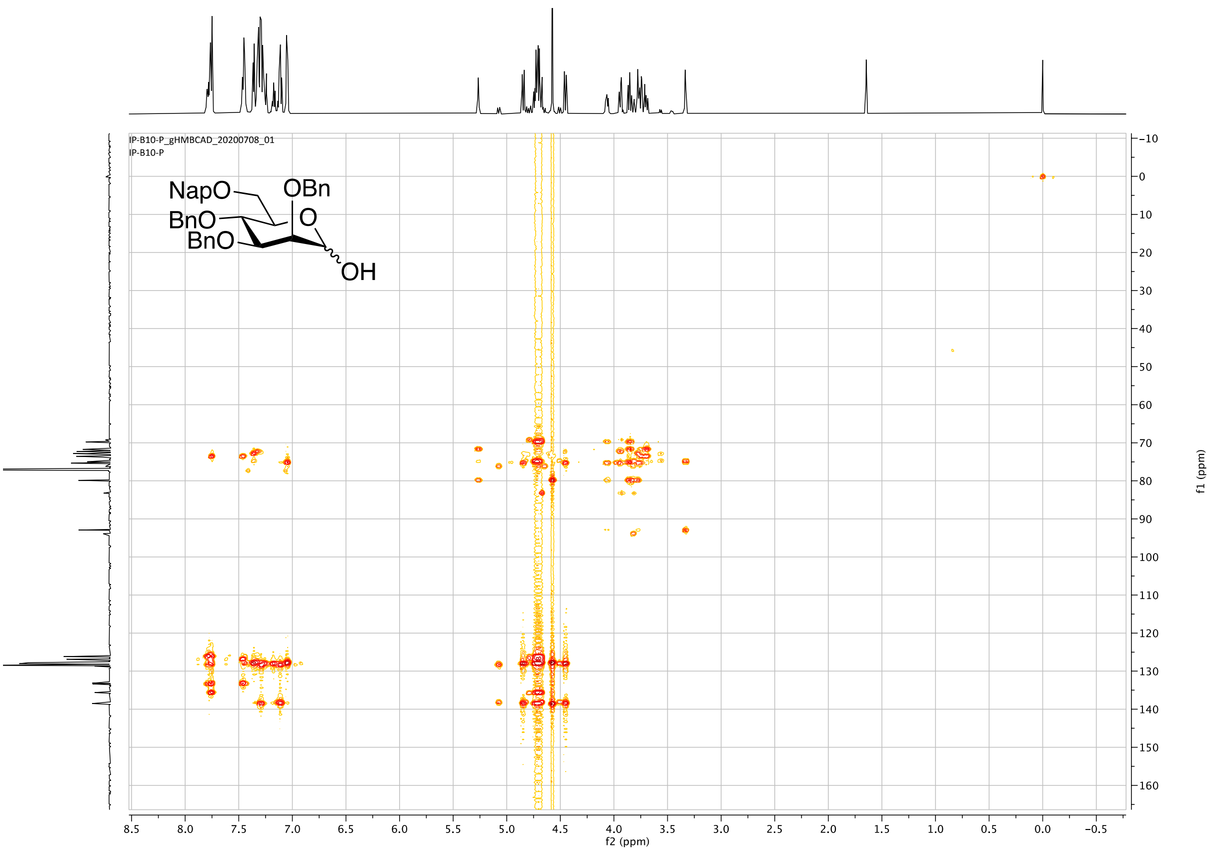

^{1}H NMR (500 MHz, Chloroform-*d*) 1f

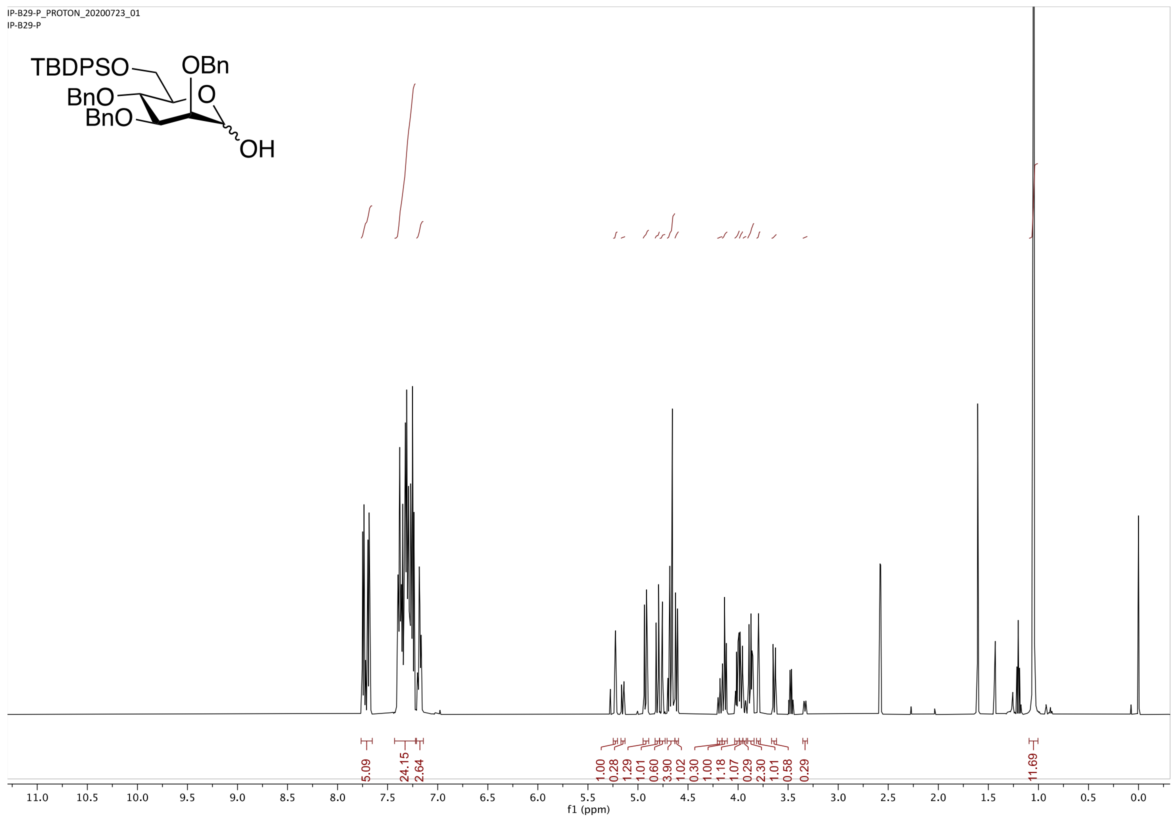

^{13}C NMR (126 MHz, Chloroform-*d*) 1f

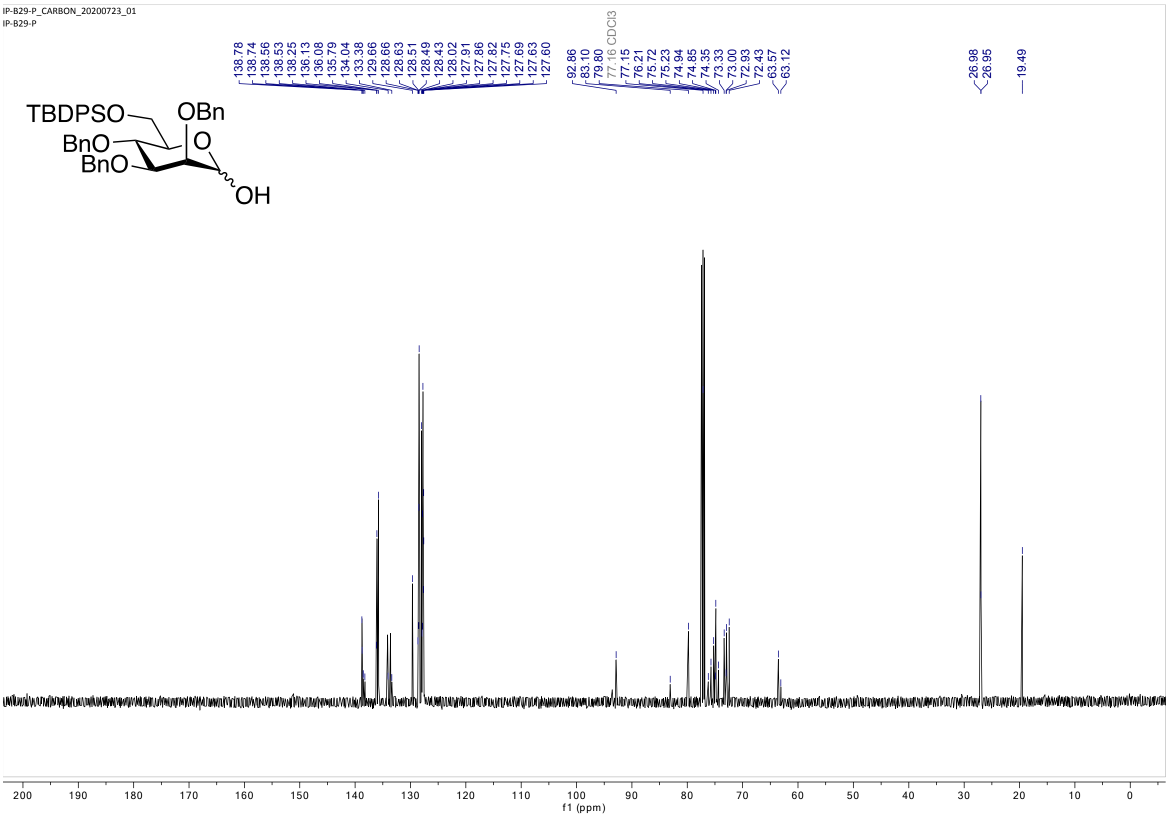

^{1}H NMR (500 MHz, Chloroform-*d*) 1g

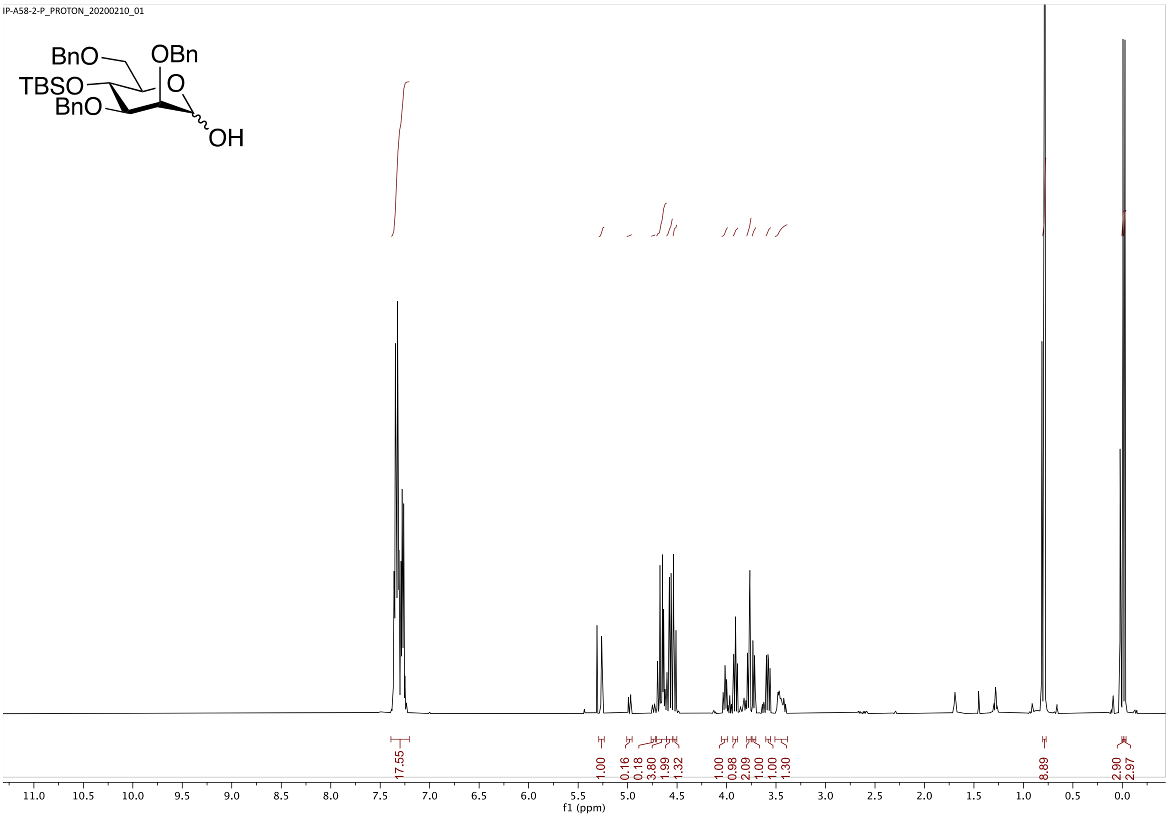

^{13}C NMR (126 MHz, Chloroform-*d*) 1g

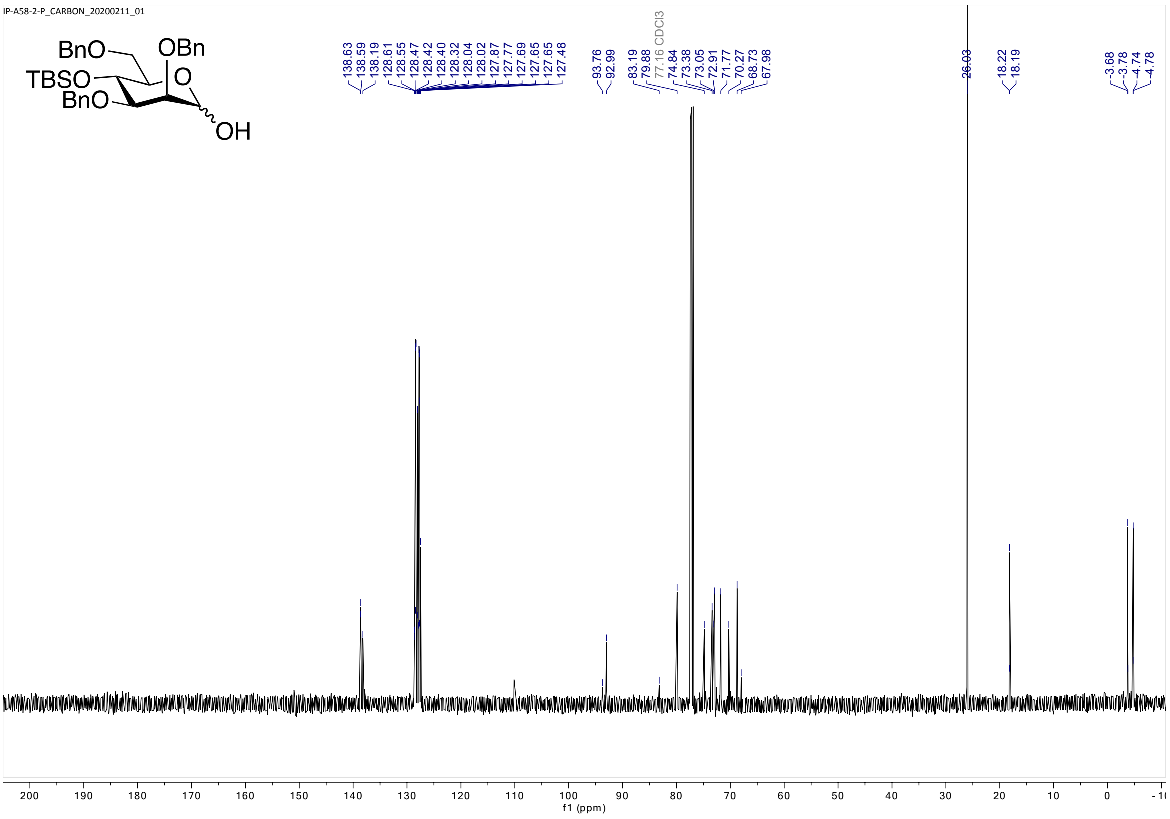

COSY NMR (500 MHz, Chloroform-*d*) 1g

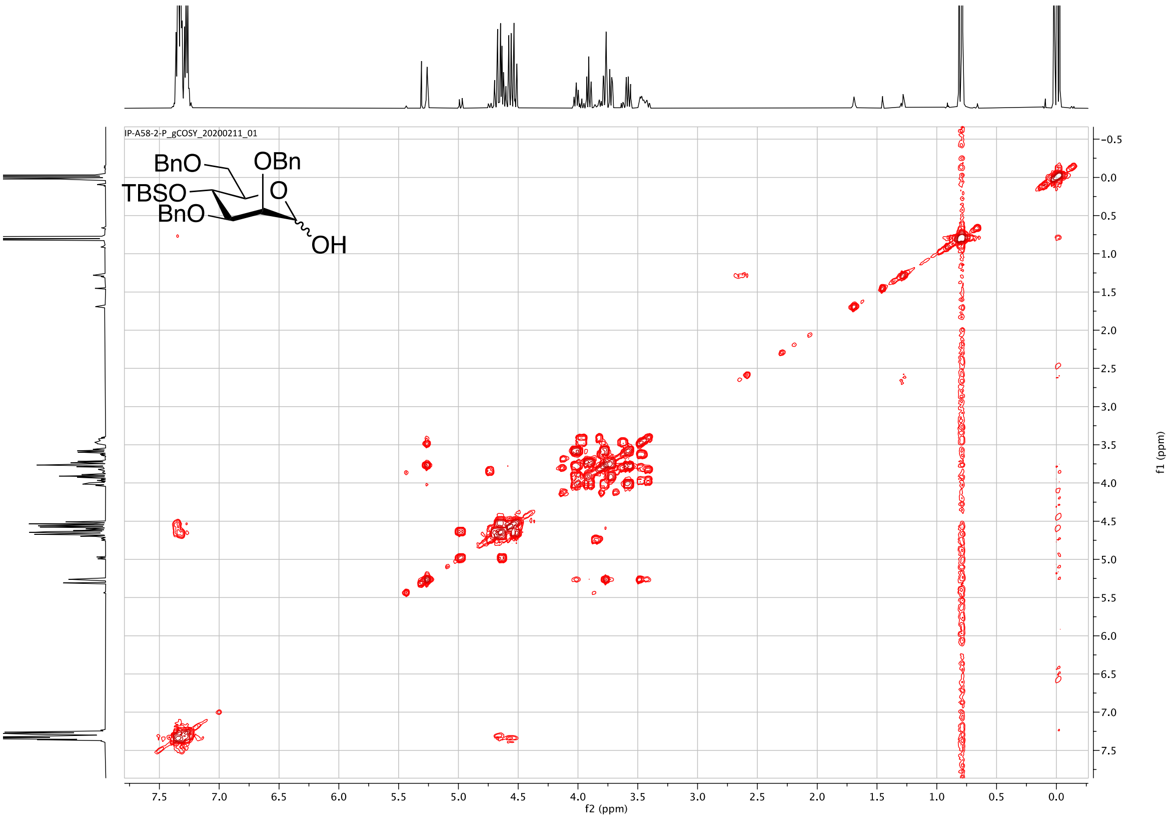

HSQC NMR (500 MHz x 126 MHz, Chloroform-*d*) 1g

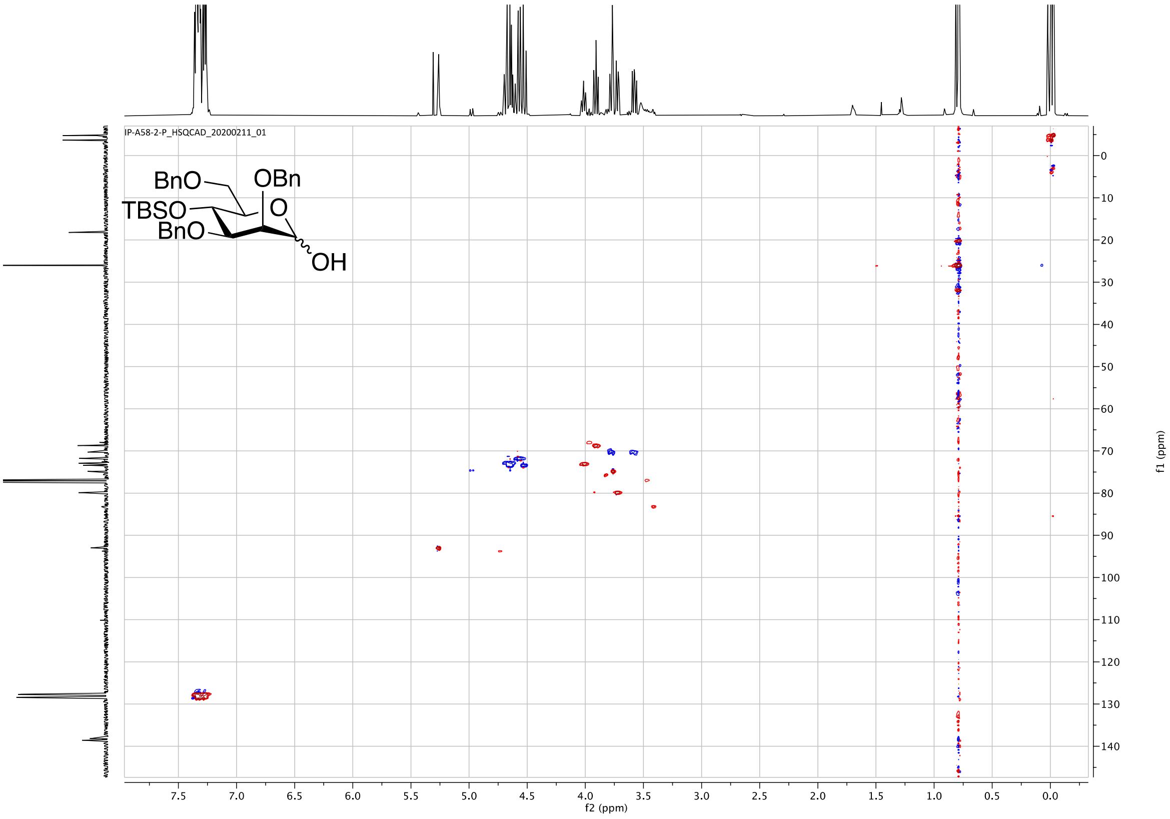

HMBC NMR (500 MHz x 126 MHz, Chloroform-*d*) 1g

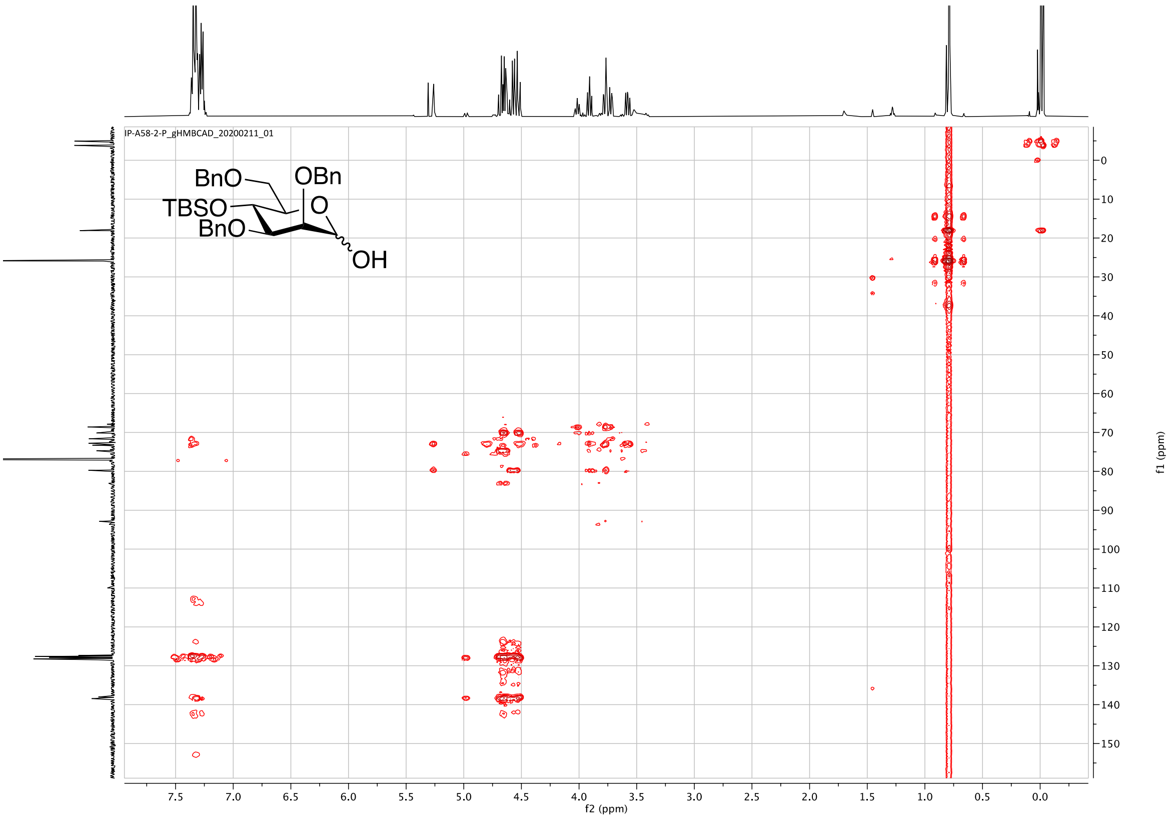

^{1}H NMR (500 MHz, Chloroform-*d*) 1h

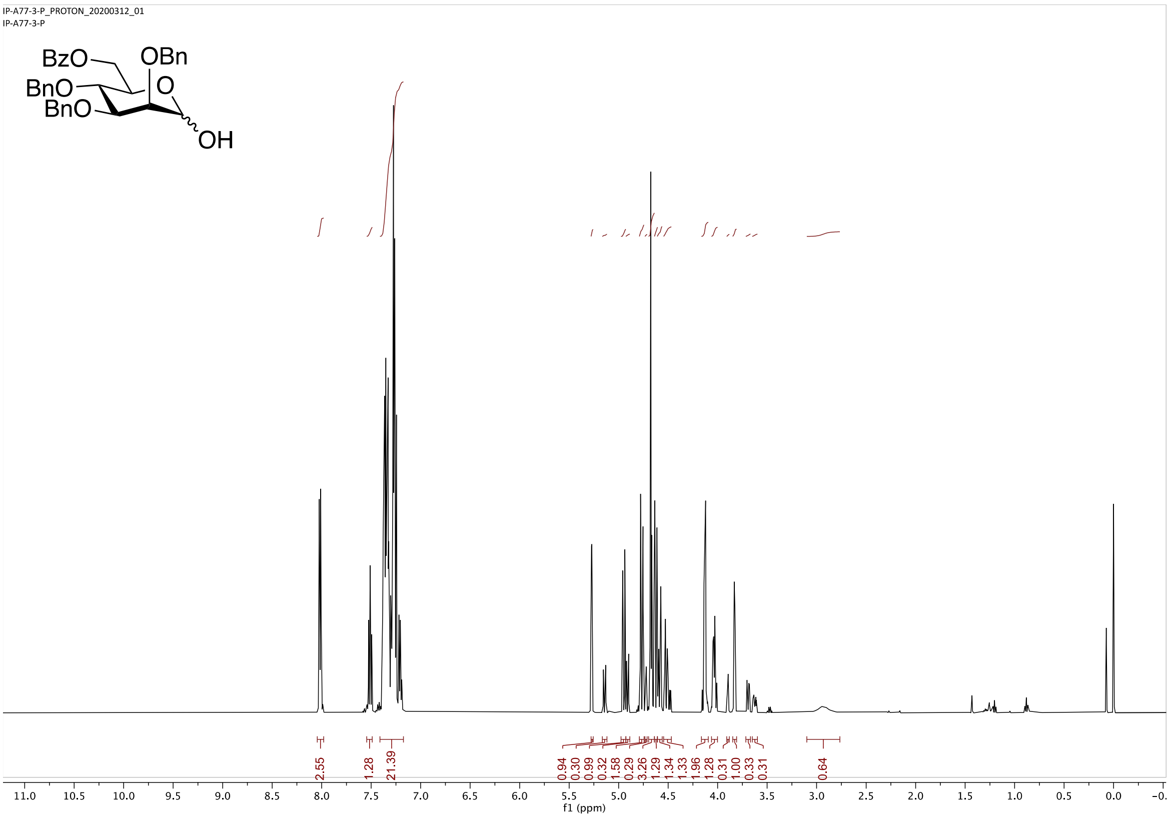

^{13}C NMR (126 MHz, Chloroform-*d*) 1h

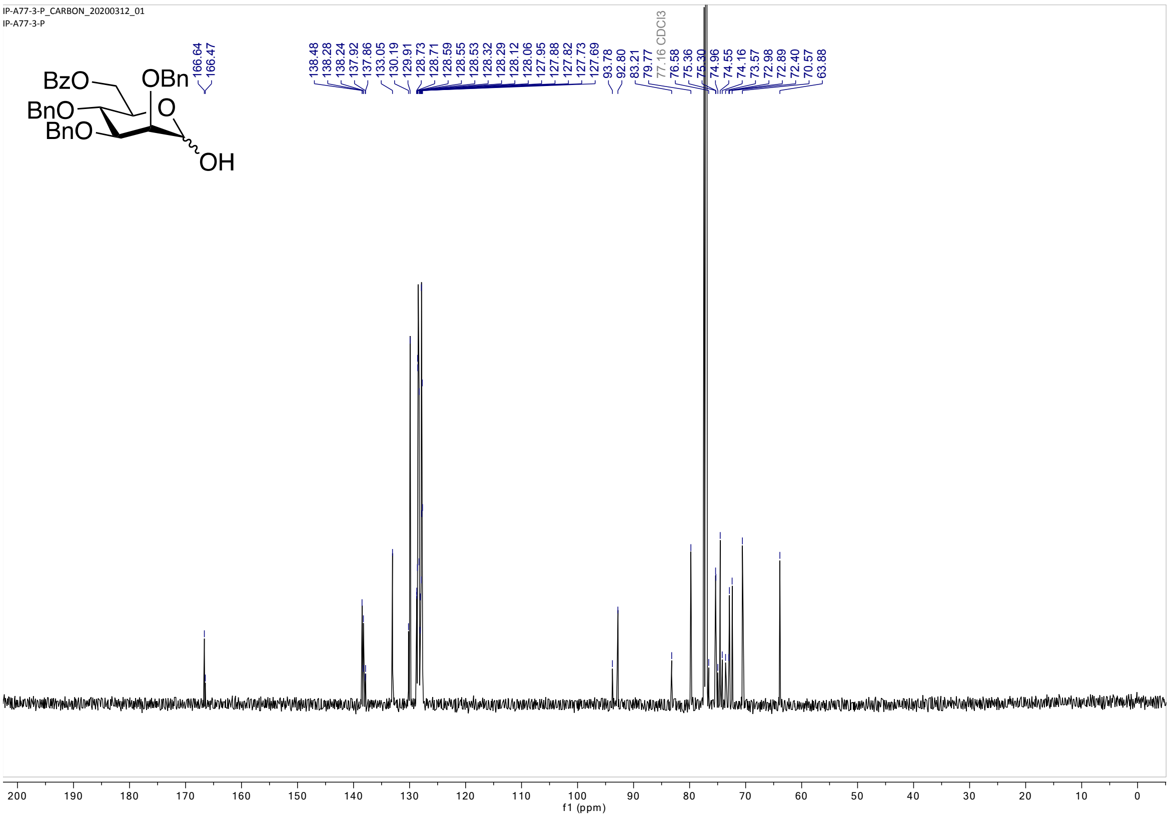

^{1}H NMR (500 MHz, Chloroform-*d*) S22

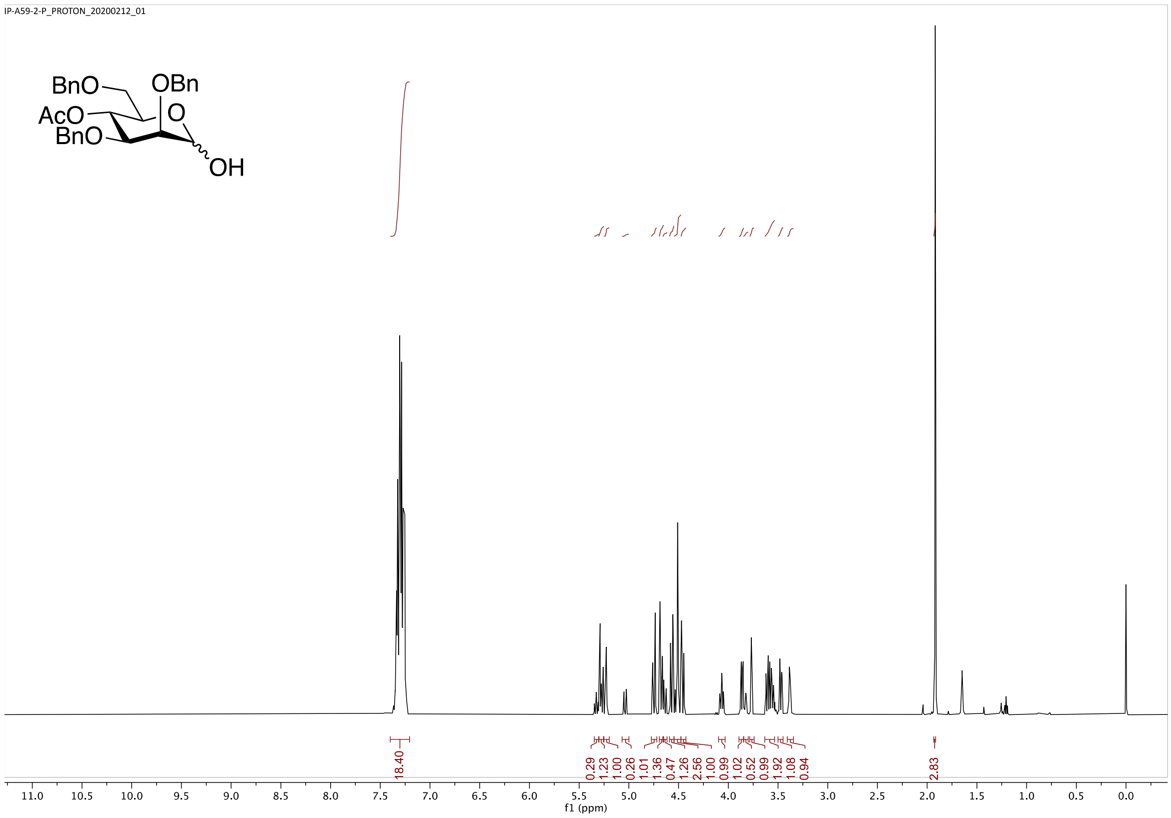

^{13}C NMR (126 MHz, Chloroform-*d*) S22

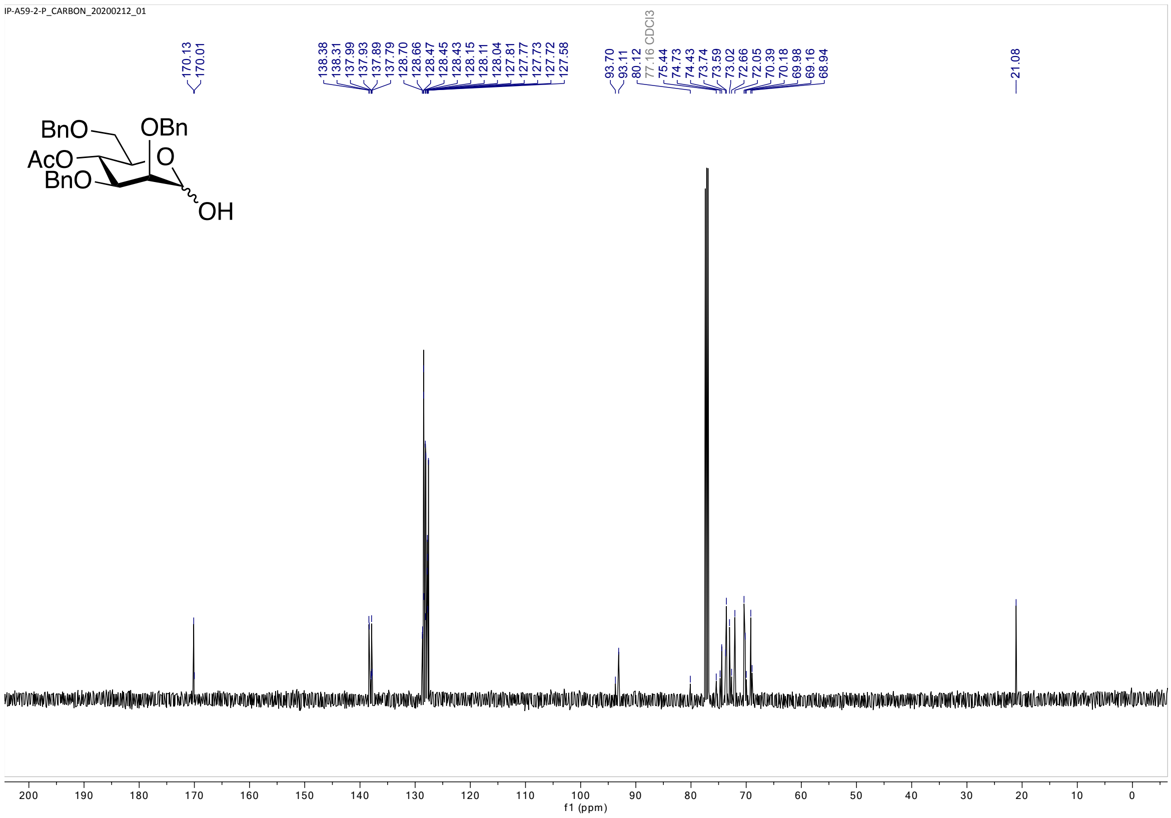

^{1}H NMR (600 MHz, Chloroform-*d*) S23

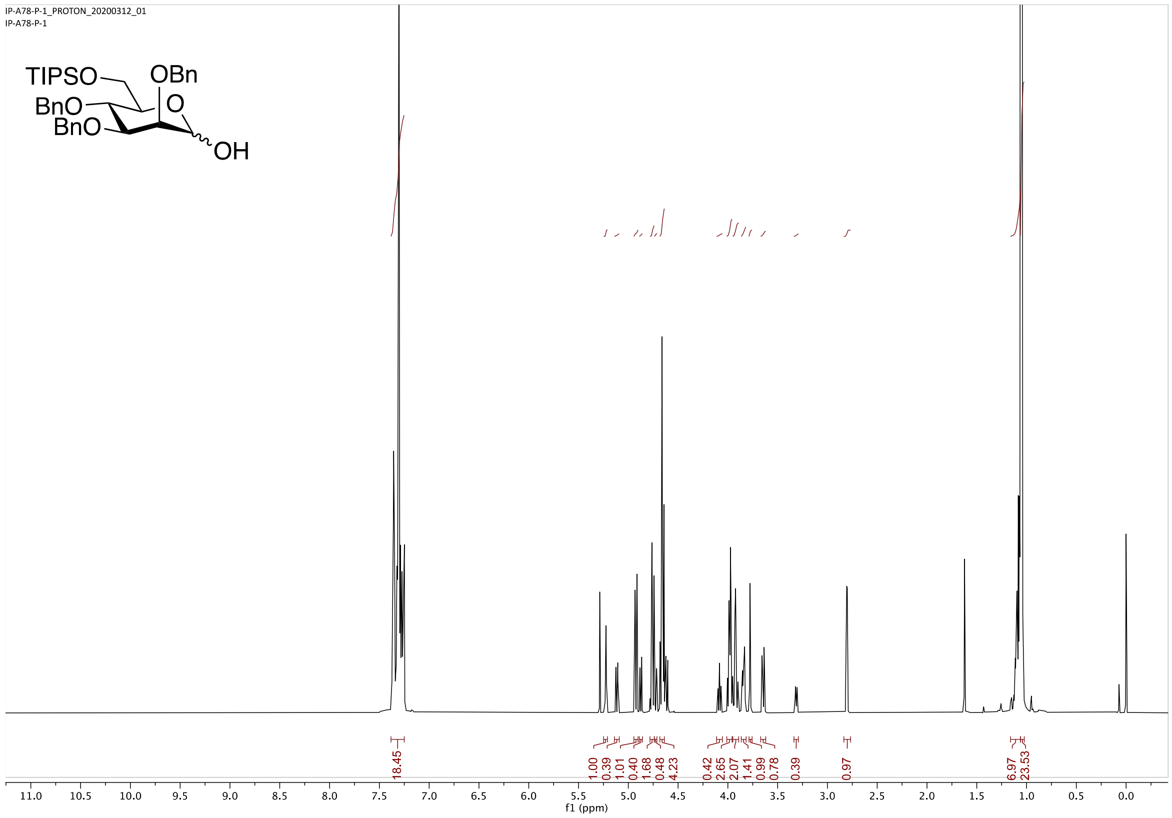

^{13}C NMR (151 MHz, Chloroform-*d*) S23

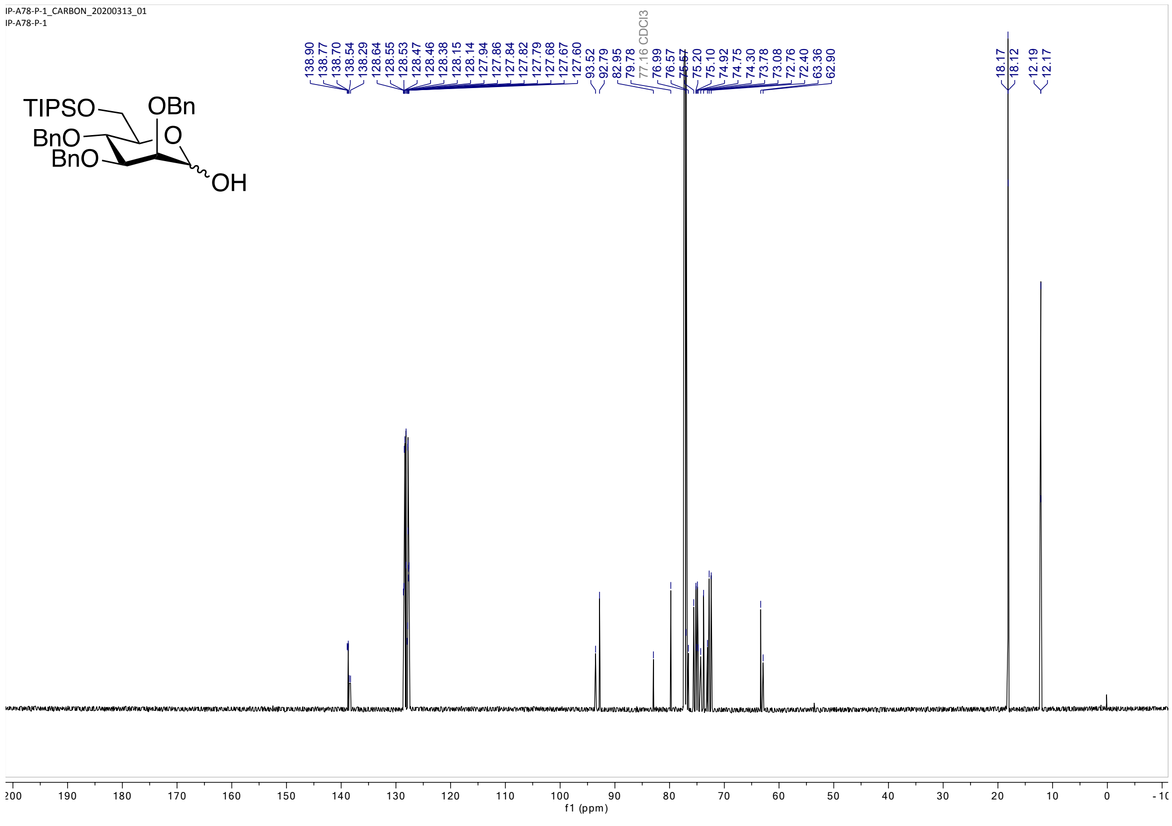

COSY NMR (600 MHz, Chloroform-*d*) S23

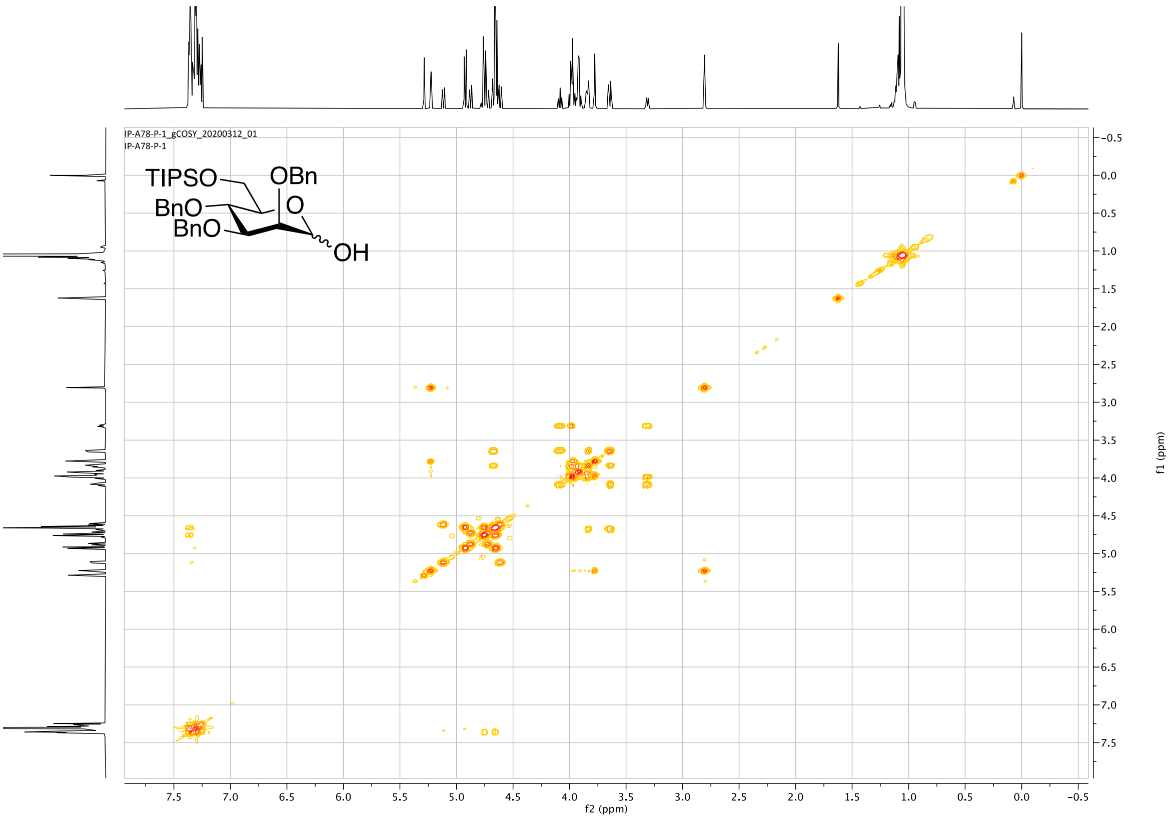

HSQC NMR (600 MHz x 151 MHz, Chloroform-*d*) S23

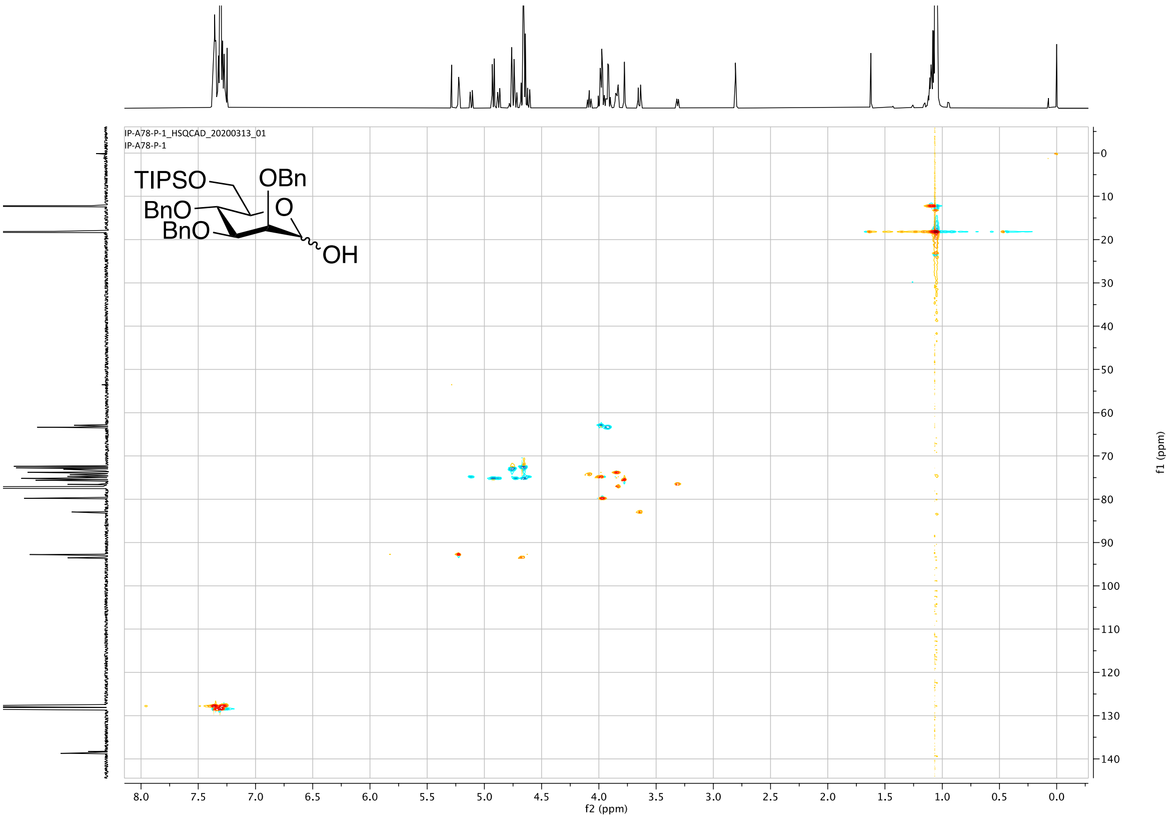

HMBC NMR (600 MHz x 151 MHz, Chloroform-*d*) S23

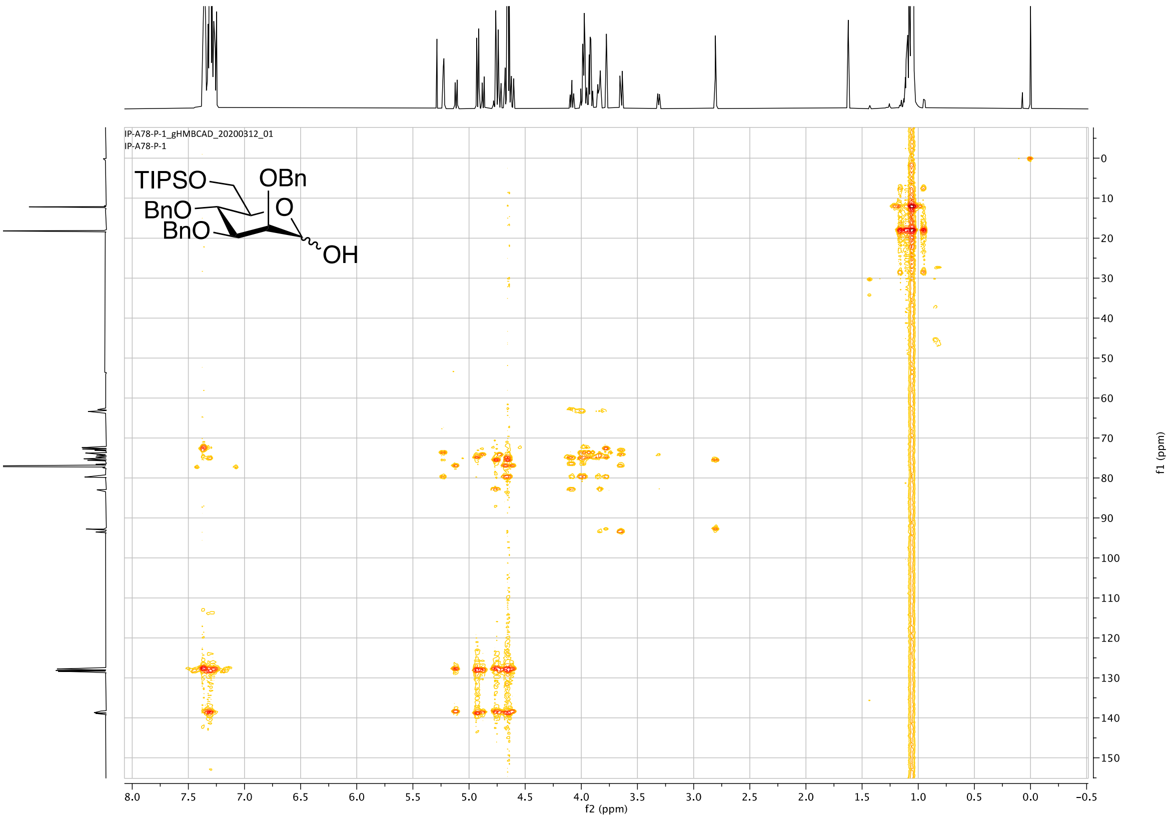

^{1}H NMR (500 MHz, Chloroform-*d*) S24

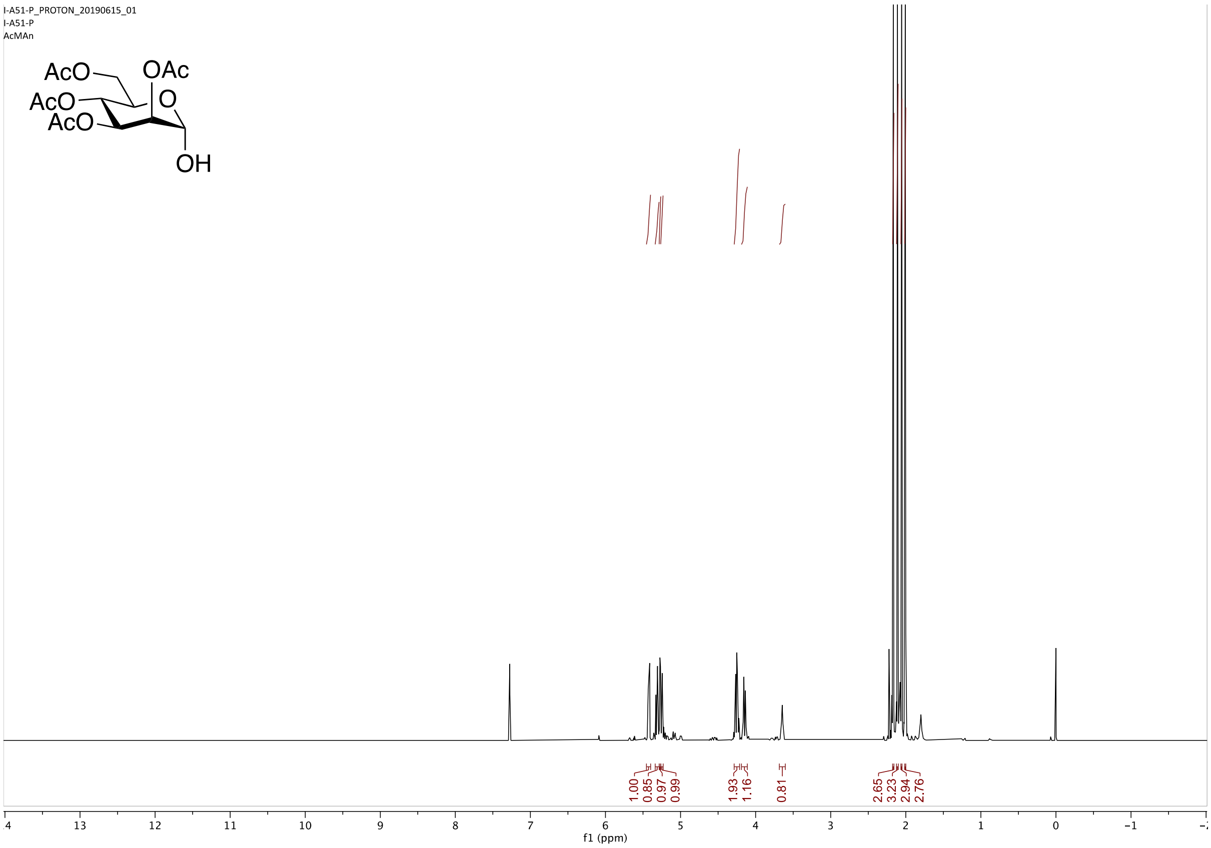

^{1}H NMR (500 MHz, Chloroform-*d*) 1i

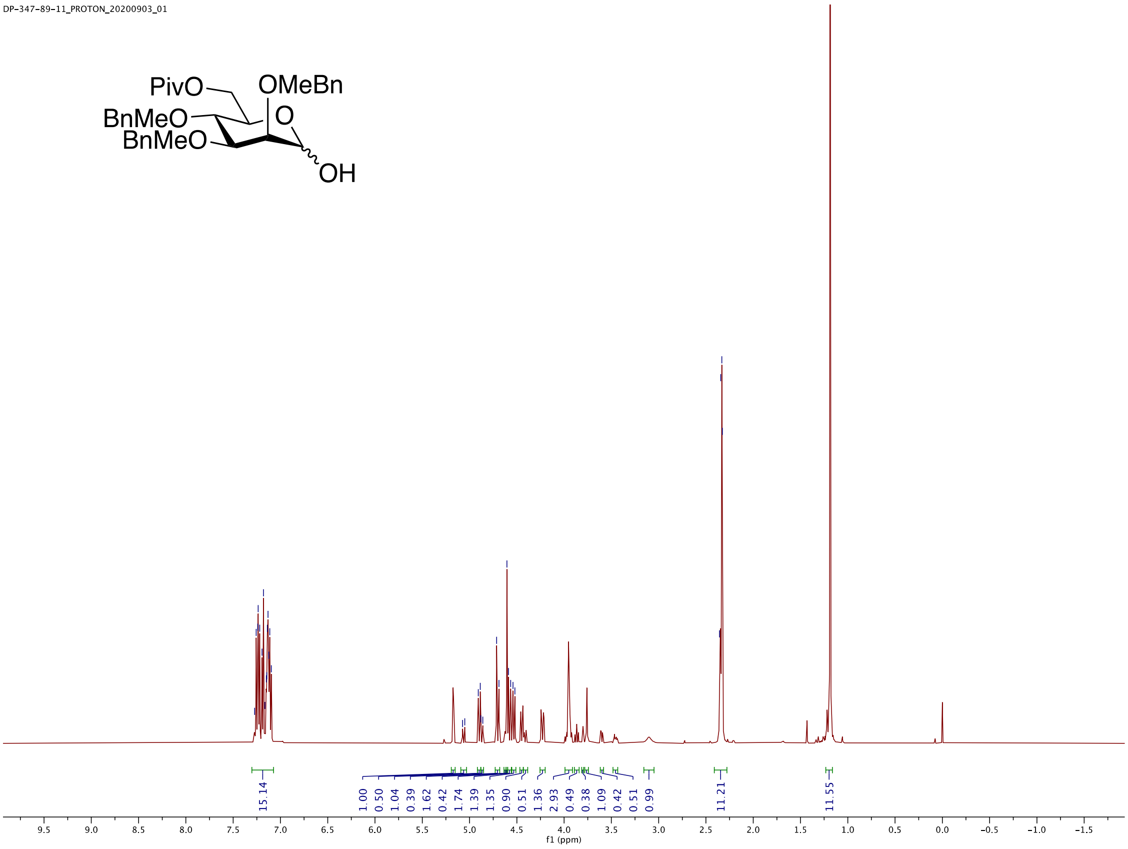

^{13}C NMR (126 MHz, Chloroform-*d*) 1i

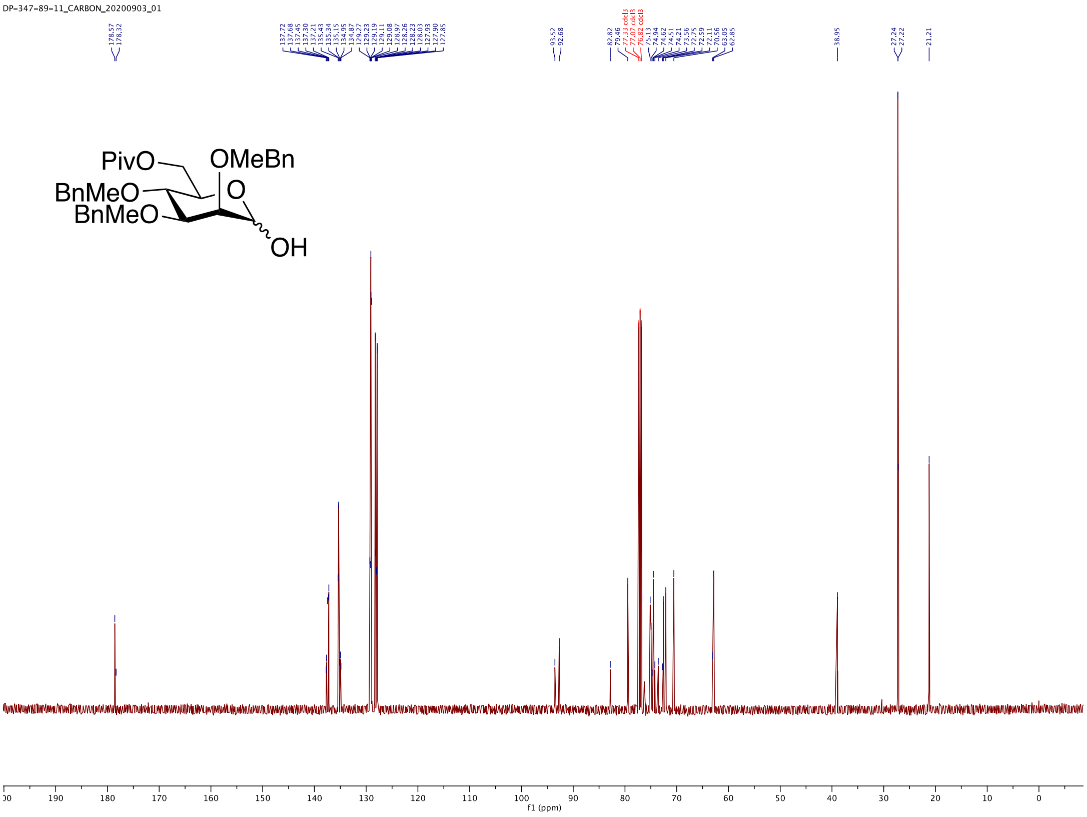

COSY NMR (500 MHz, Chloroform-*d*) 1i

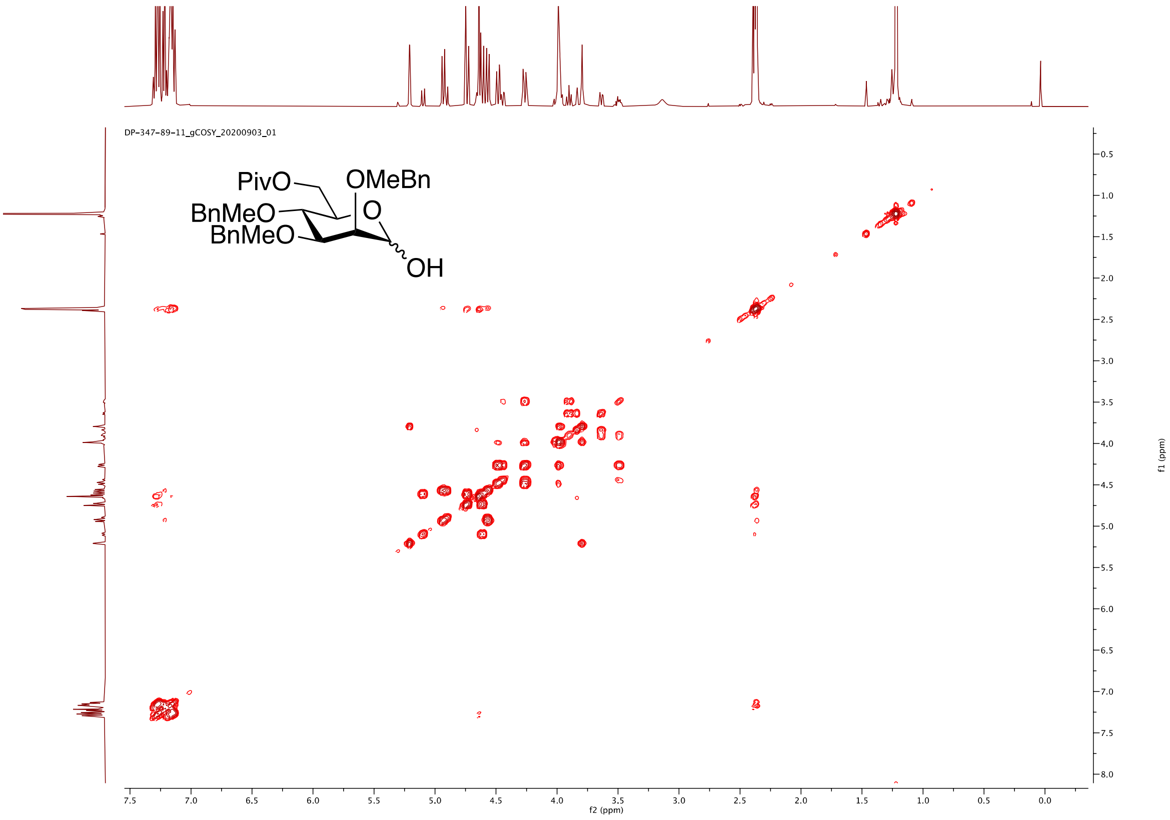

HSQC NMR (500 MHz x 126 MHz, Chloroform-*d*) 1i

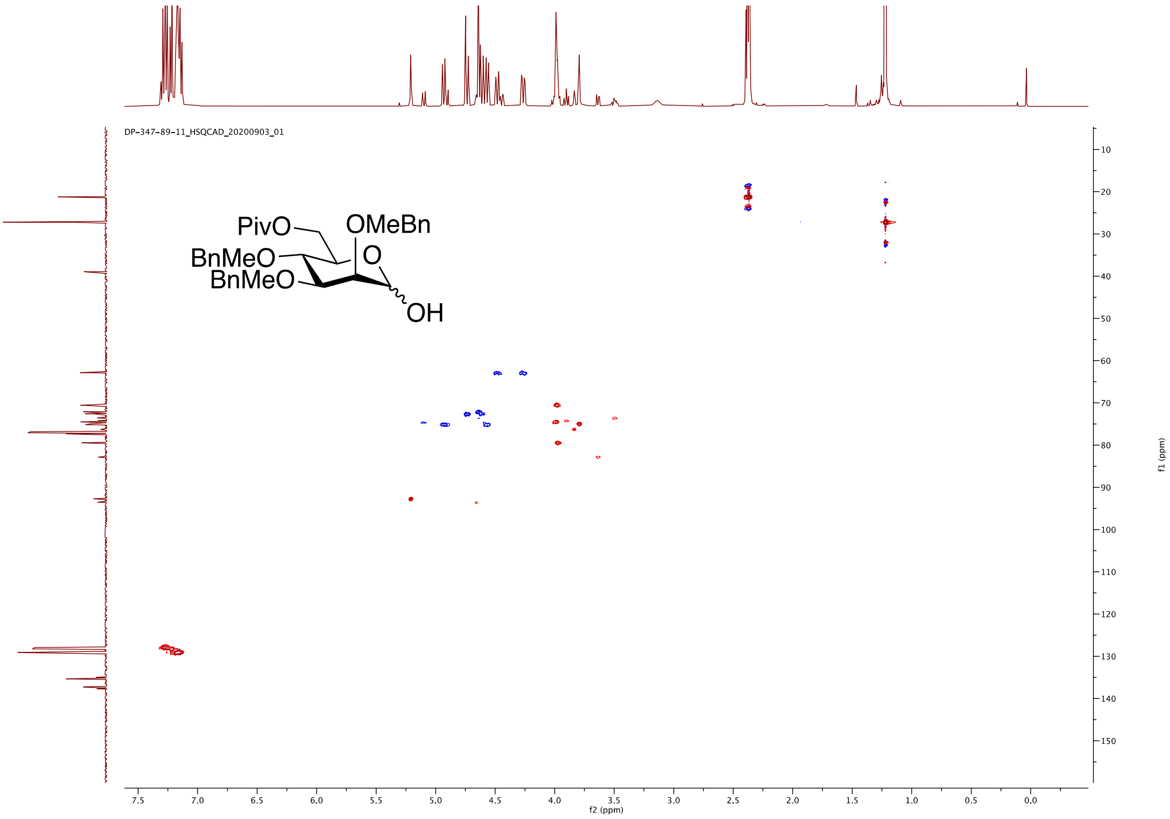

HMBC NMR (500 MHz x 126 MHz, Chloroform-*d*) 1i

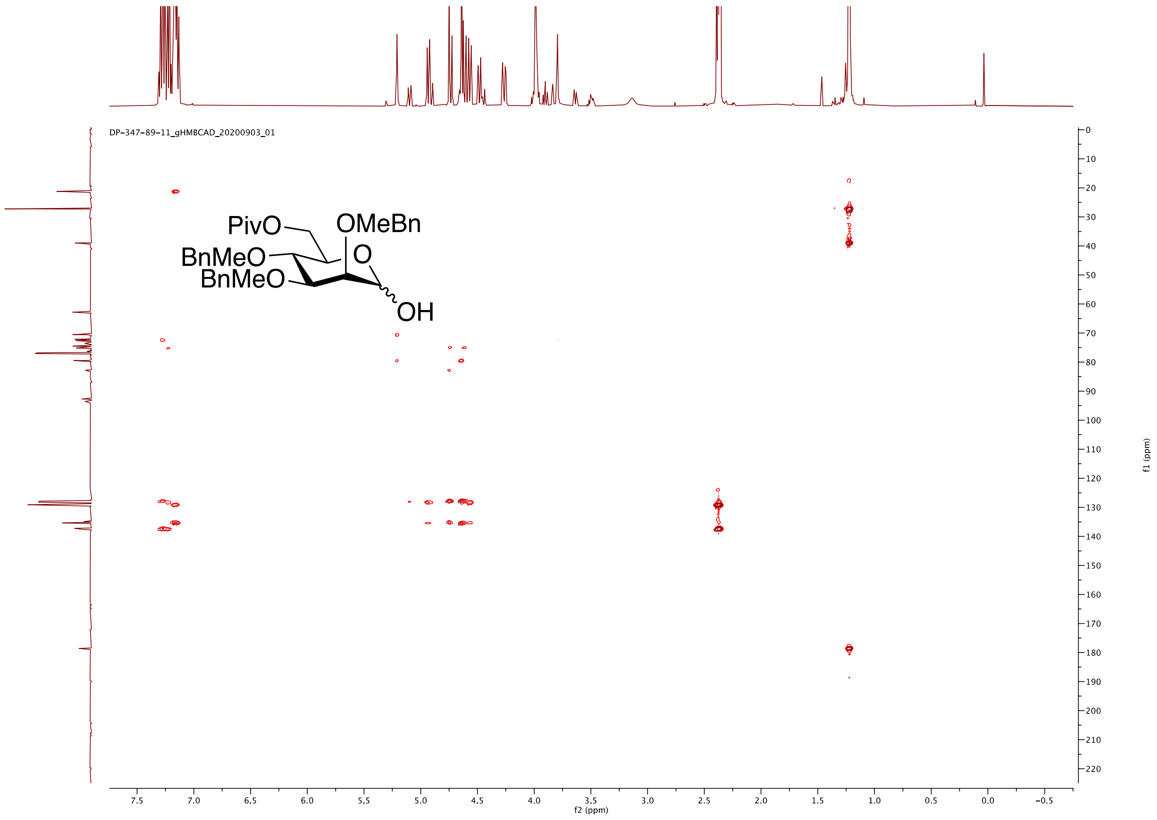

^{1}H NMR (500 MHz, Chloroform-*d*) 1j

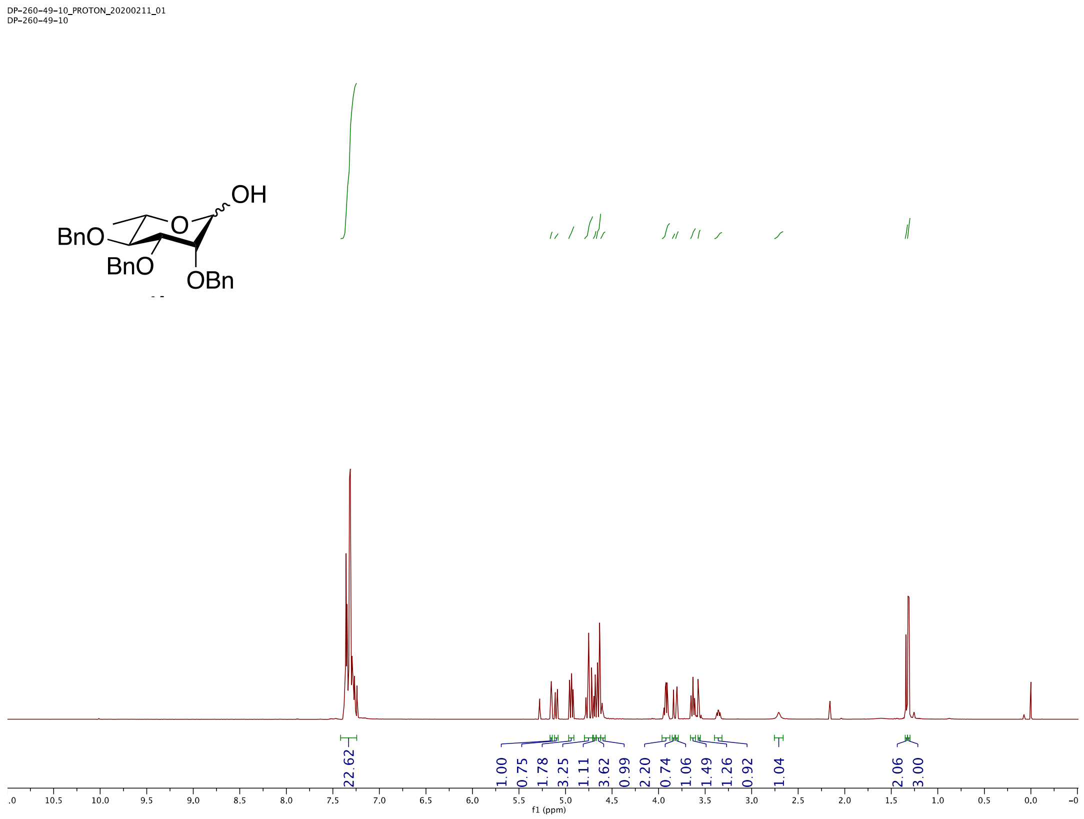

^{13}C NMR (126 MHz, Chloroform-*d*) 1j

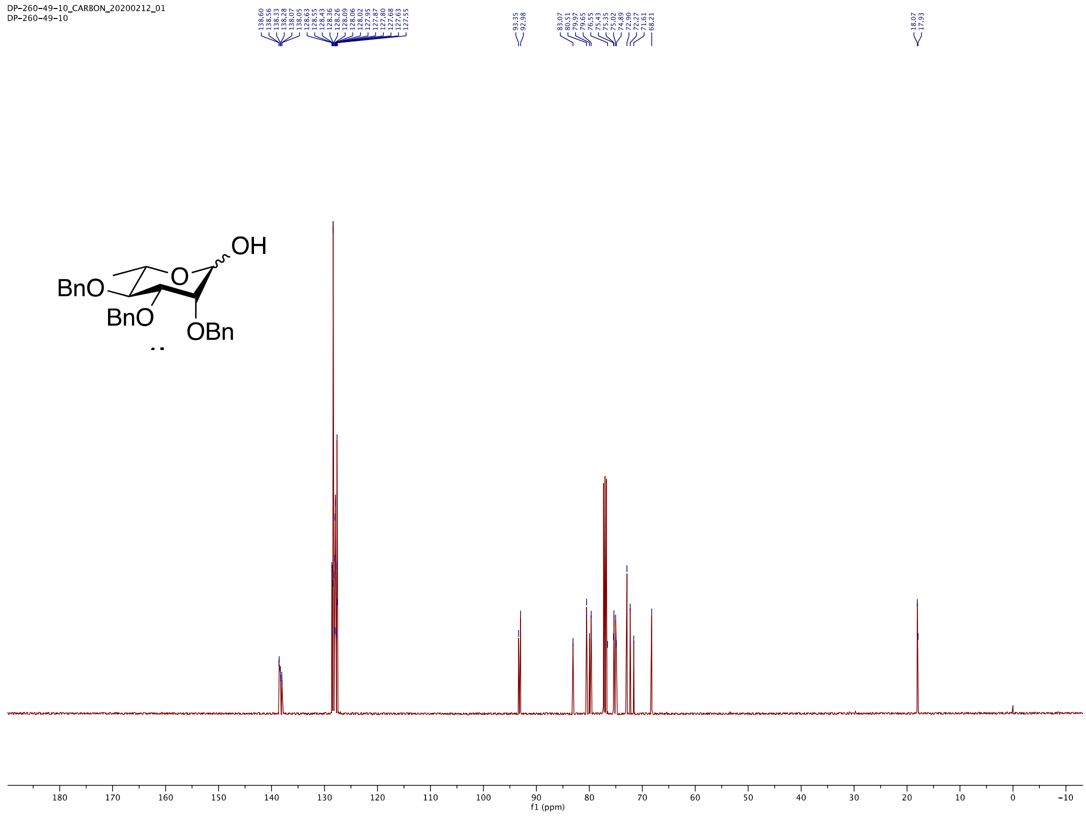

^{13}C - ^{1}H decoupled HSQC (500 x 126 MHz, Chloroform-*d*) 1j

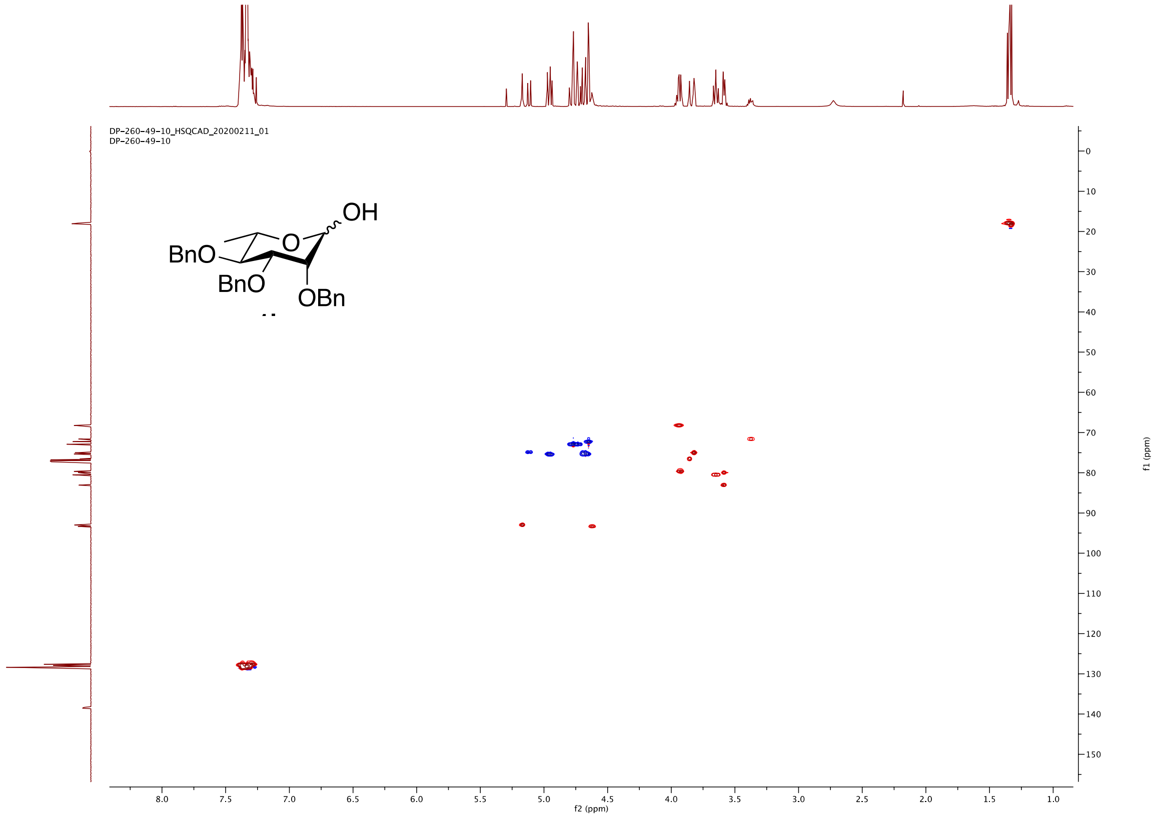

COSY (500 x 500 MHz, Chloroform-*d*) 1j

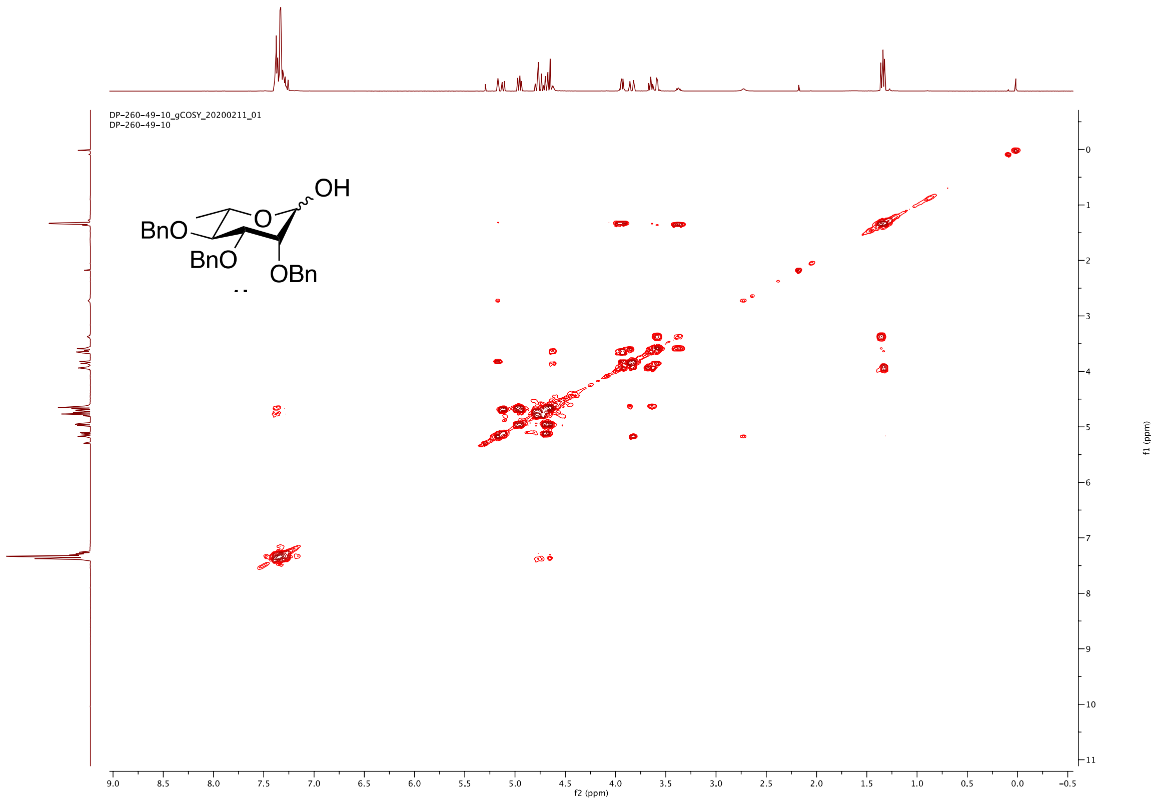

HMBC (500 x 126 MHz, Chloroform-*d*) 1j

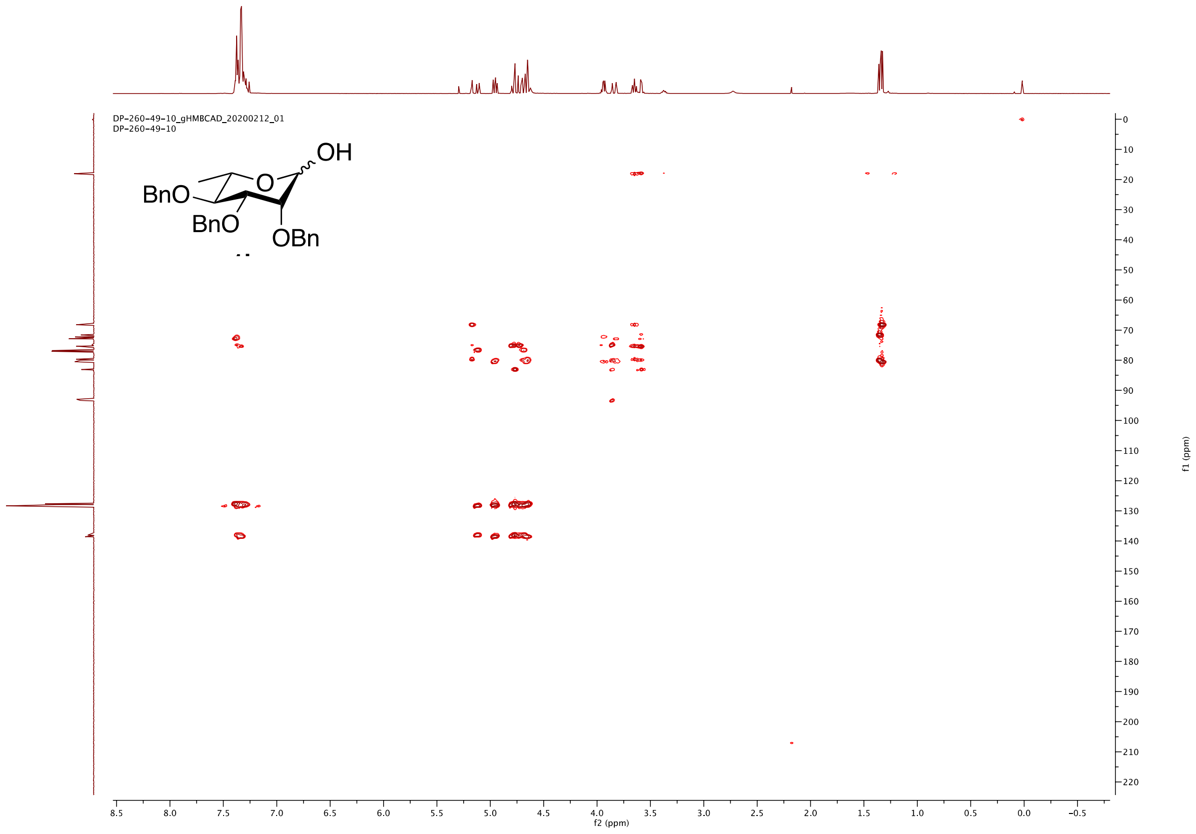

^{1}H NMR (500 MHz, Chloroform-*d*) 1k

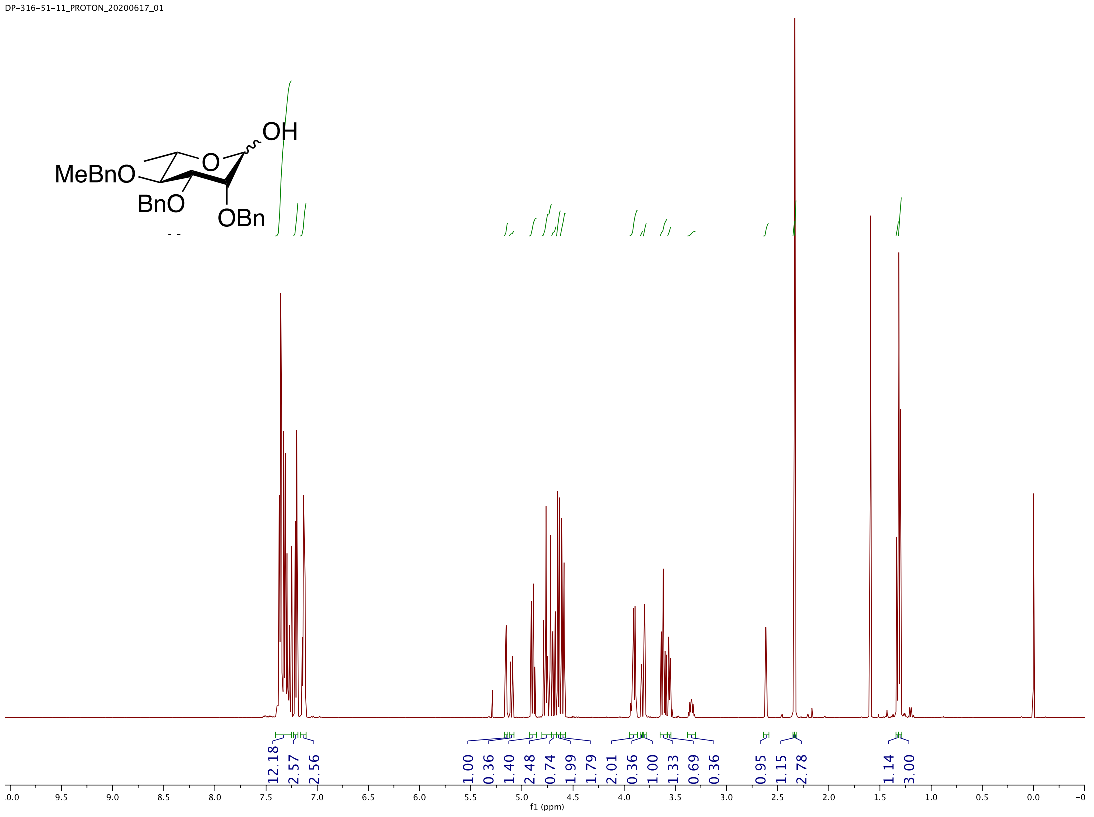

^{13}C NMR (126 MHz, Chloroform-*d*) 1k

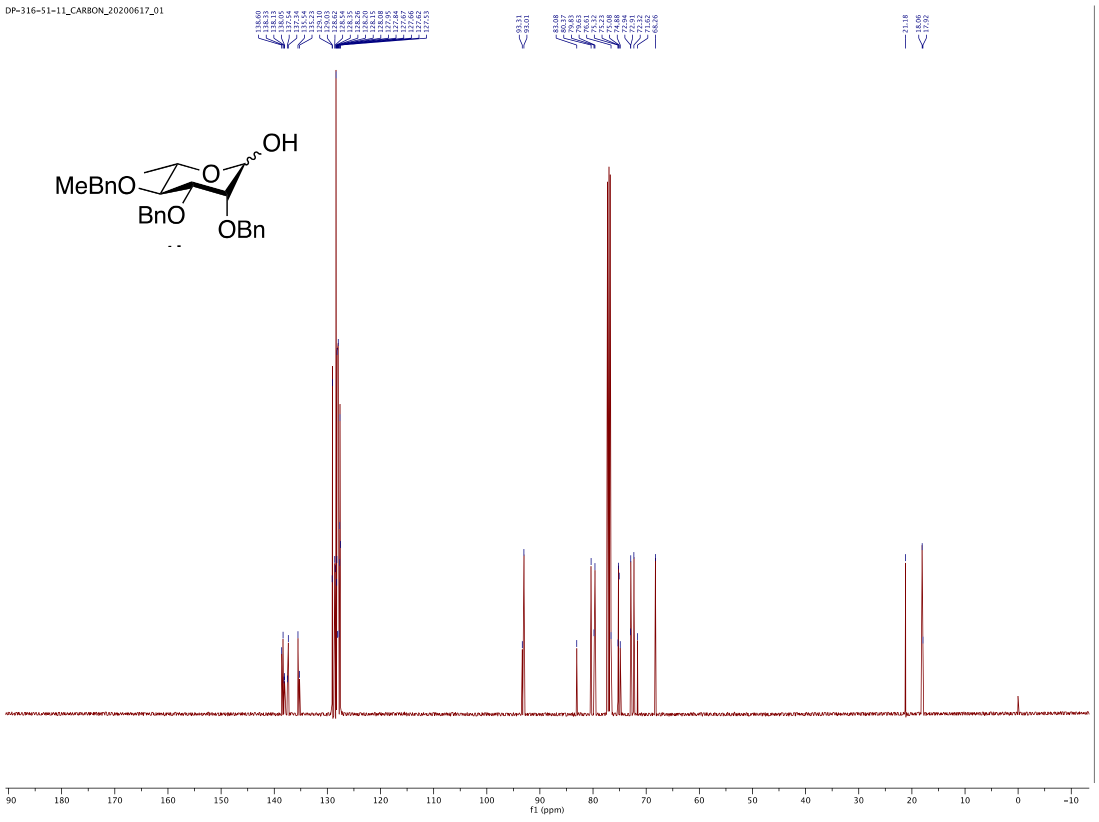

^{13}C - ^{1}H decoupled HSQC (500 x 126 MHz, Chloroform-*d*) 1k

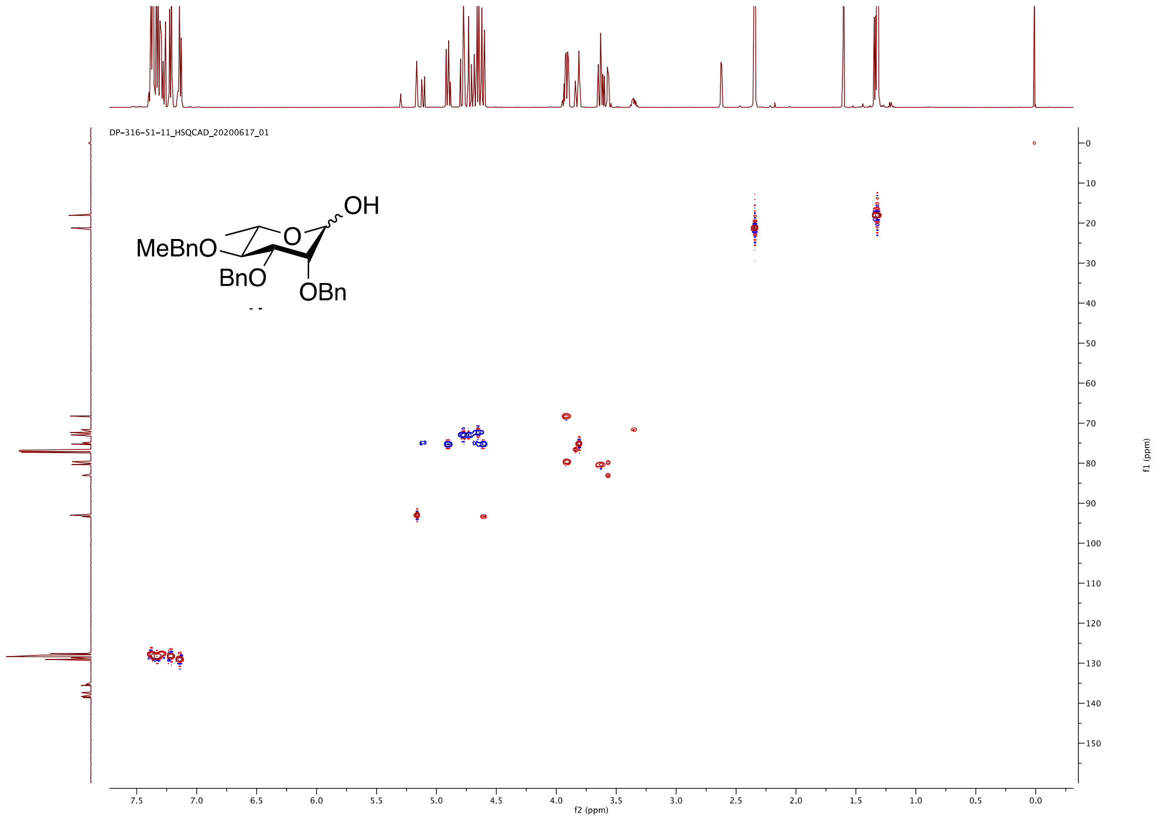

COSY NMR (650 MHz, Chloroform-*d*) 1k

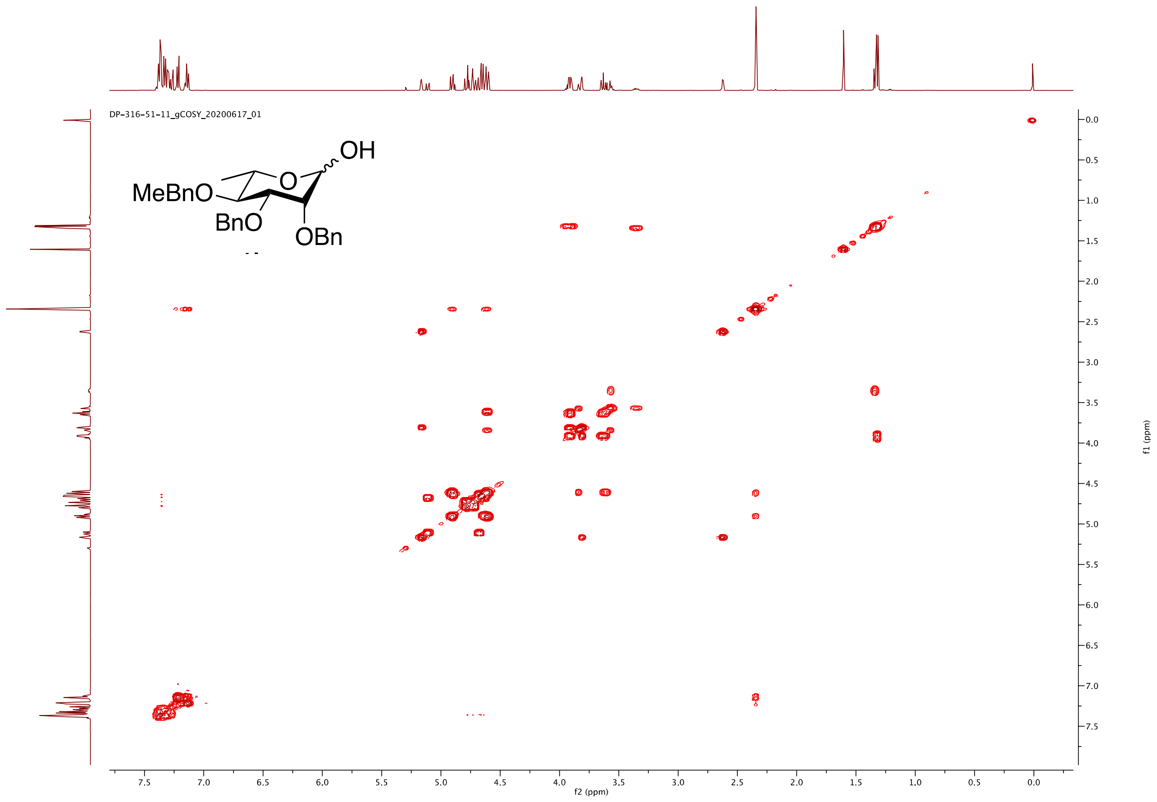

HMBC (500 x 126 MHz, Chloroform-*d*) 1k

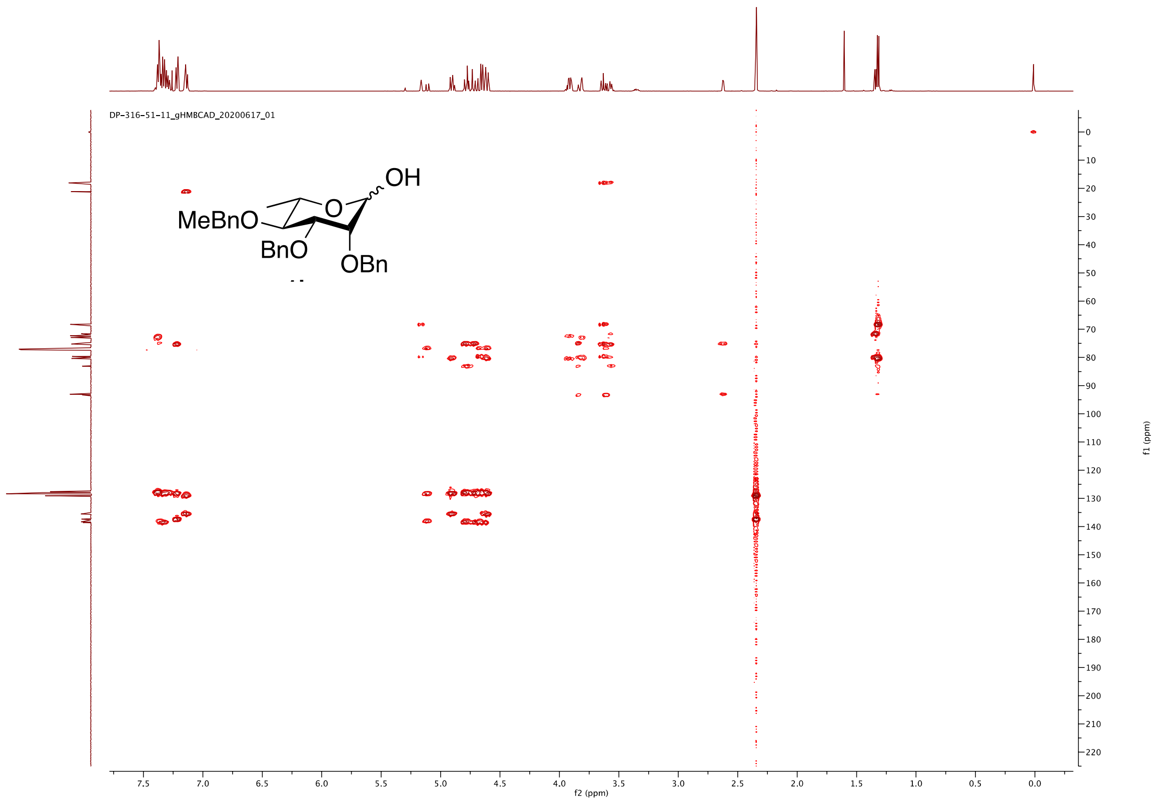

^{1}H NMR (500 MHz, Chloroform-*d*) S35

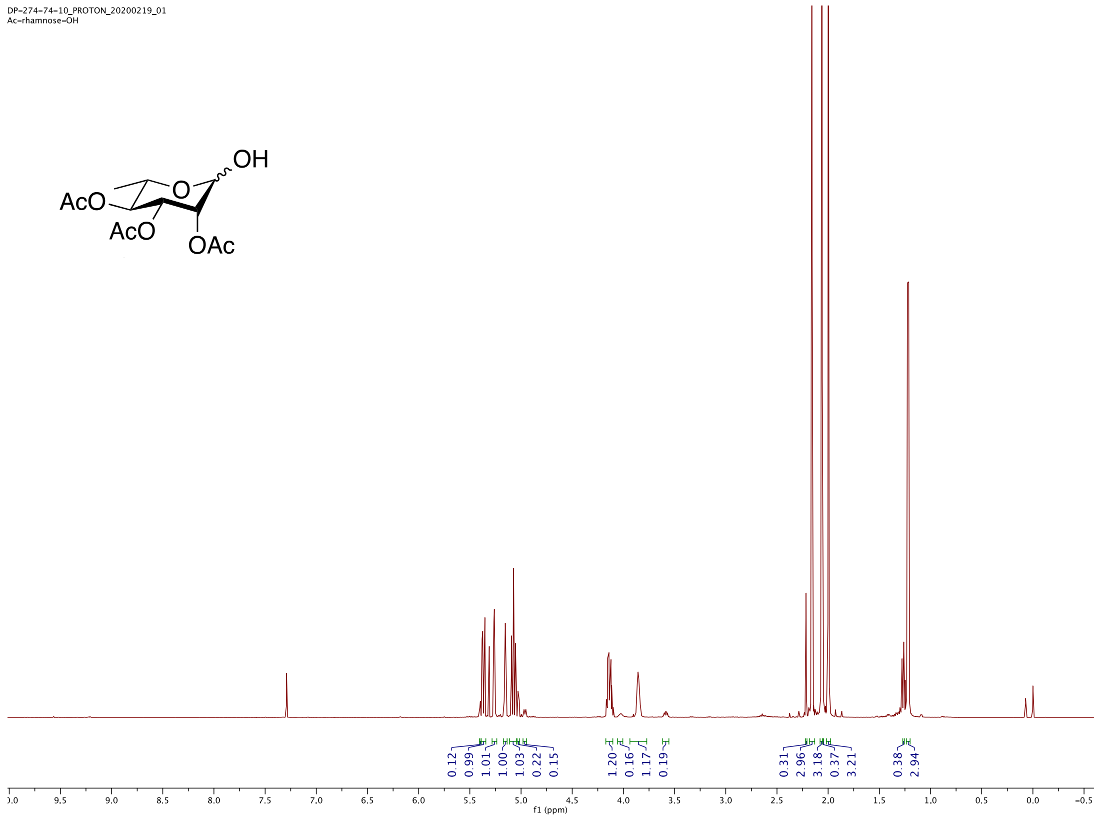

^{13}C NMR (126 MHz, Chloroform-*d*) S35

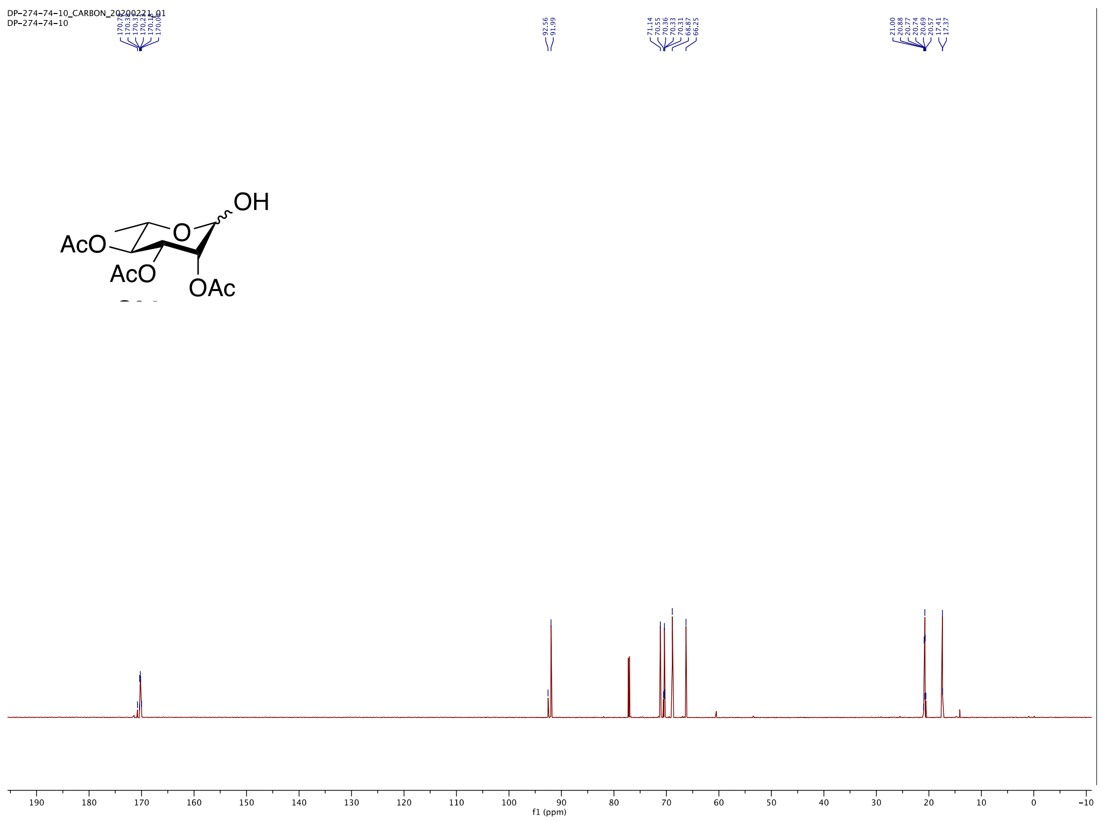

^{13}C - ^{1}H decoupled HSQC (500 x 126 MHz, Chloroform-*d*) S35

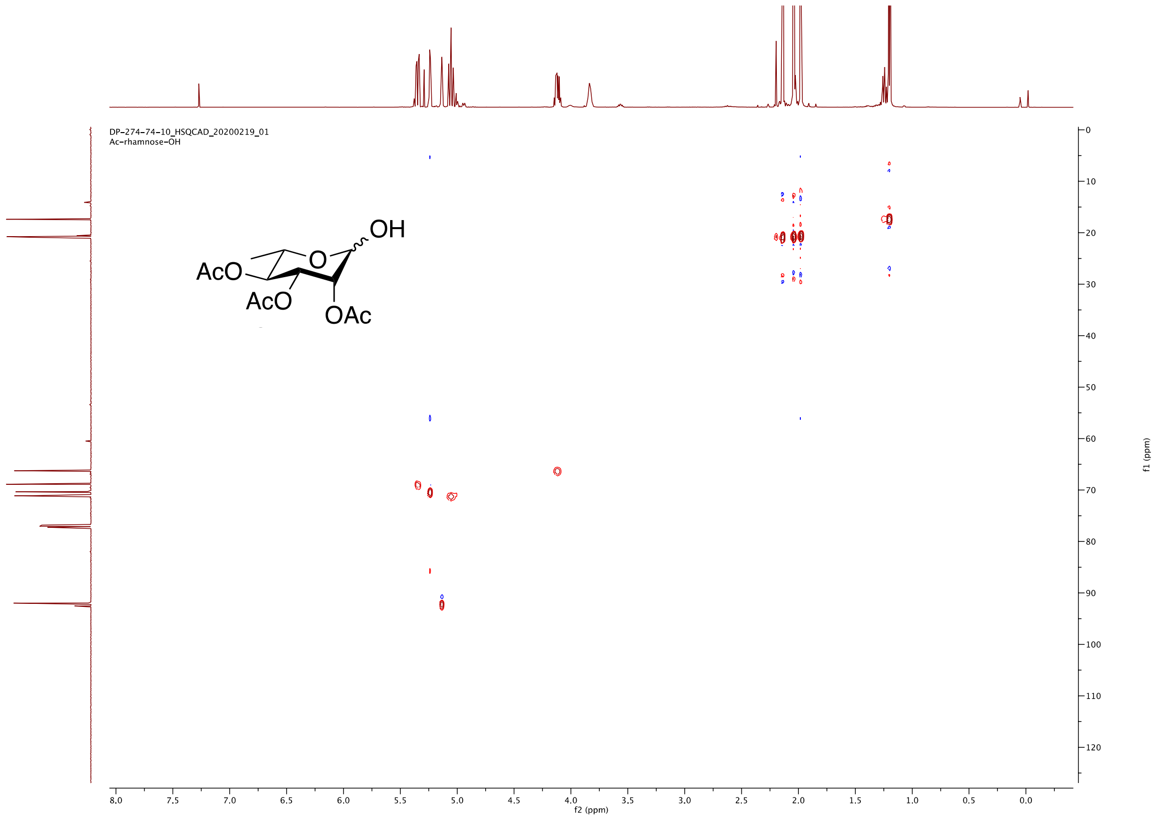

COSY (500 x 500 MHz, Chloroform-*d*) S35

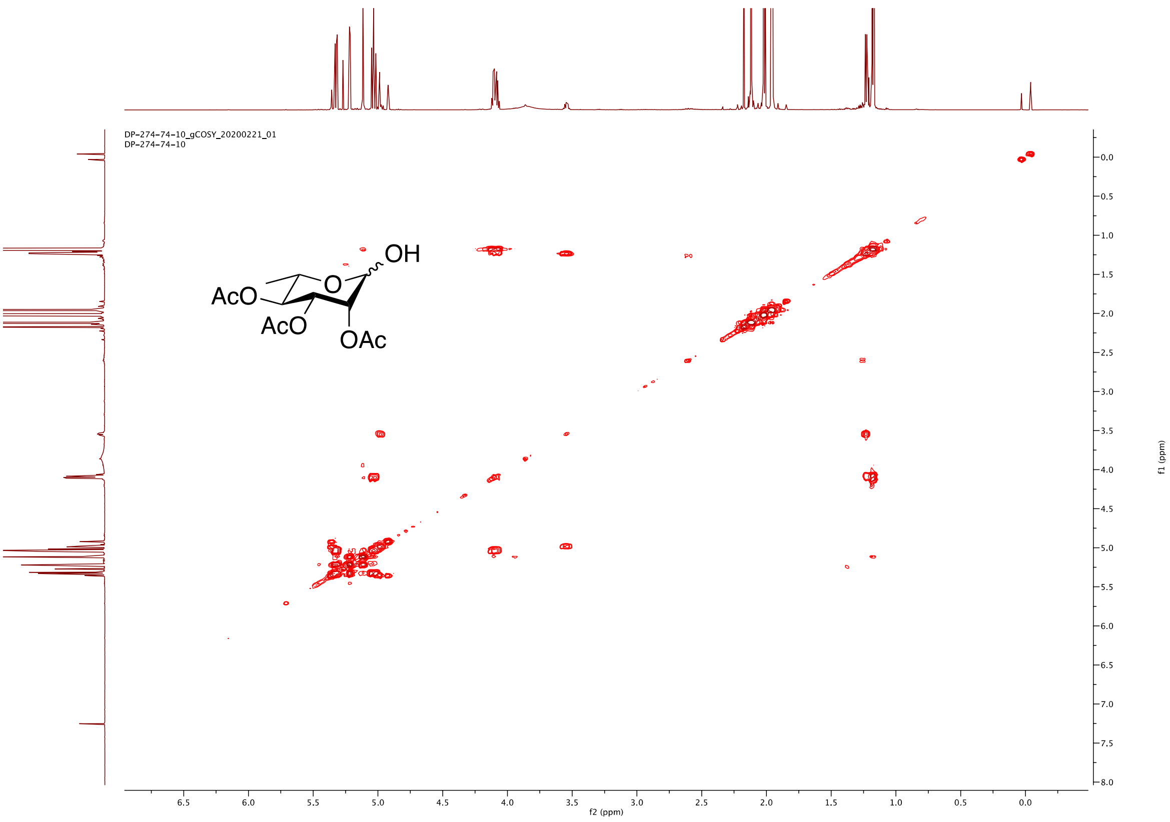

^{1}H NMR (500 MHz, Chloroform-*d*) 3a

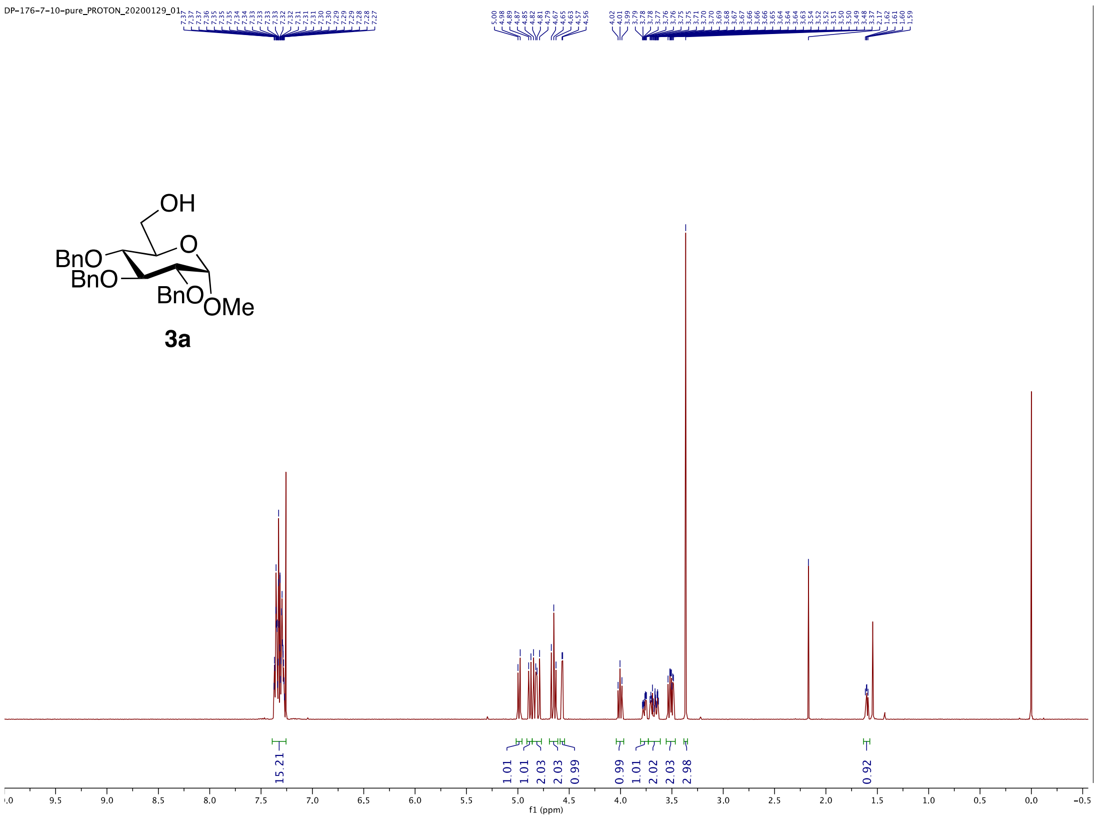

^{1}H NMR (500 MHz, Chloroform-*d*) S38

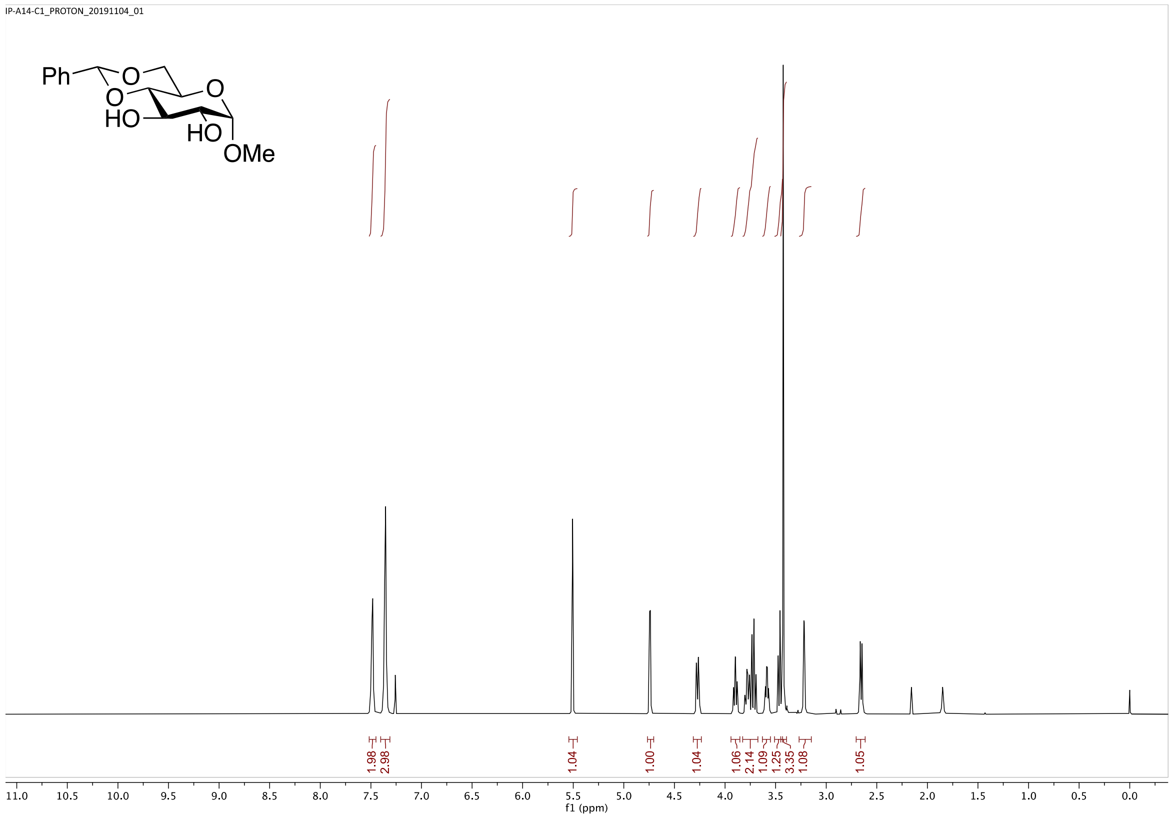

^{13}C NMR (126 MHz, Chloroform-*d*) S38

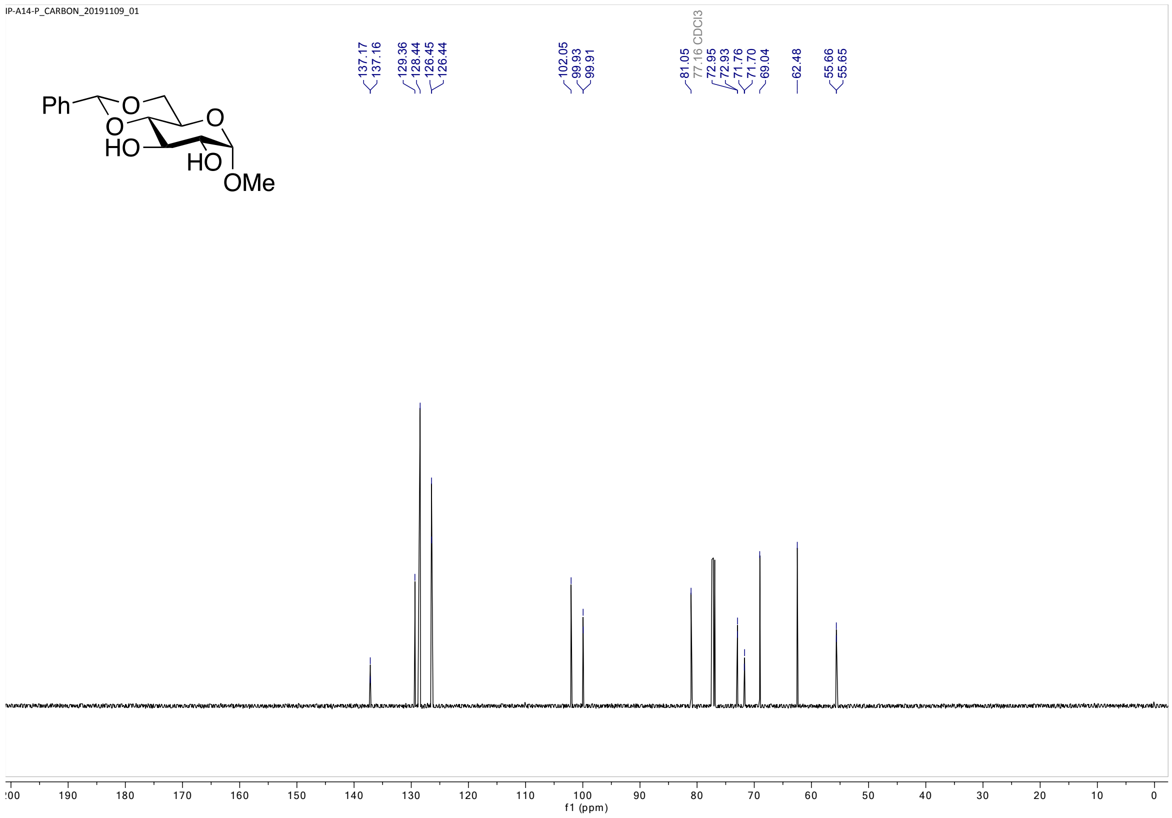

^{1}H NMR (500 MHz, Chloroform-*d*) S39

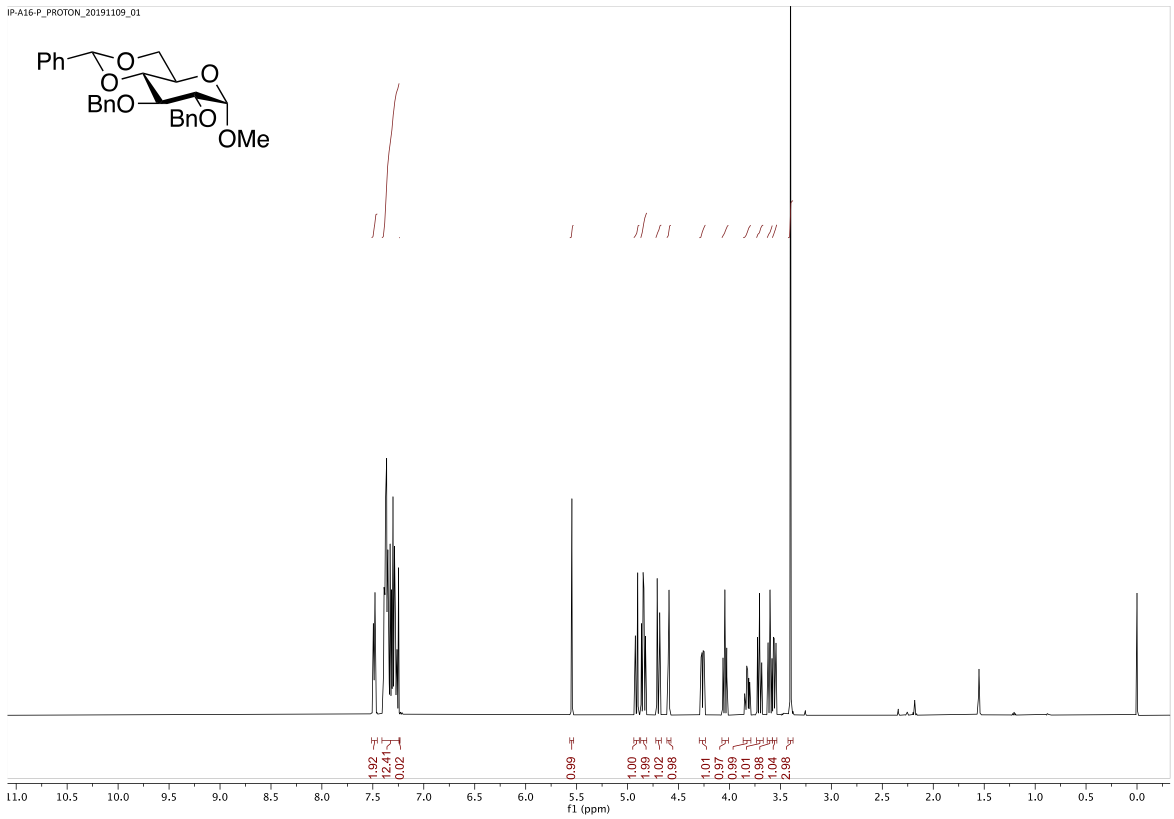

^{13}C NMR (126 MHz, Chloroform-*d*) S39

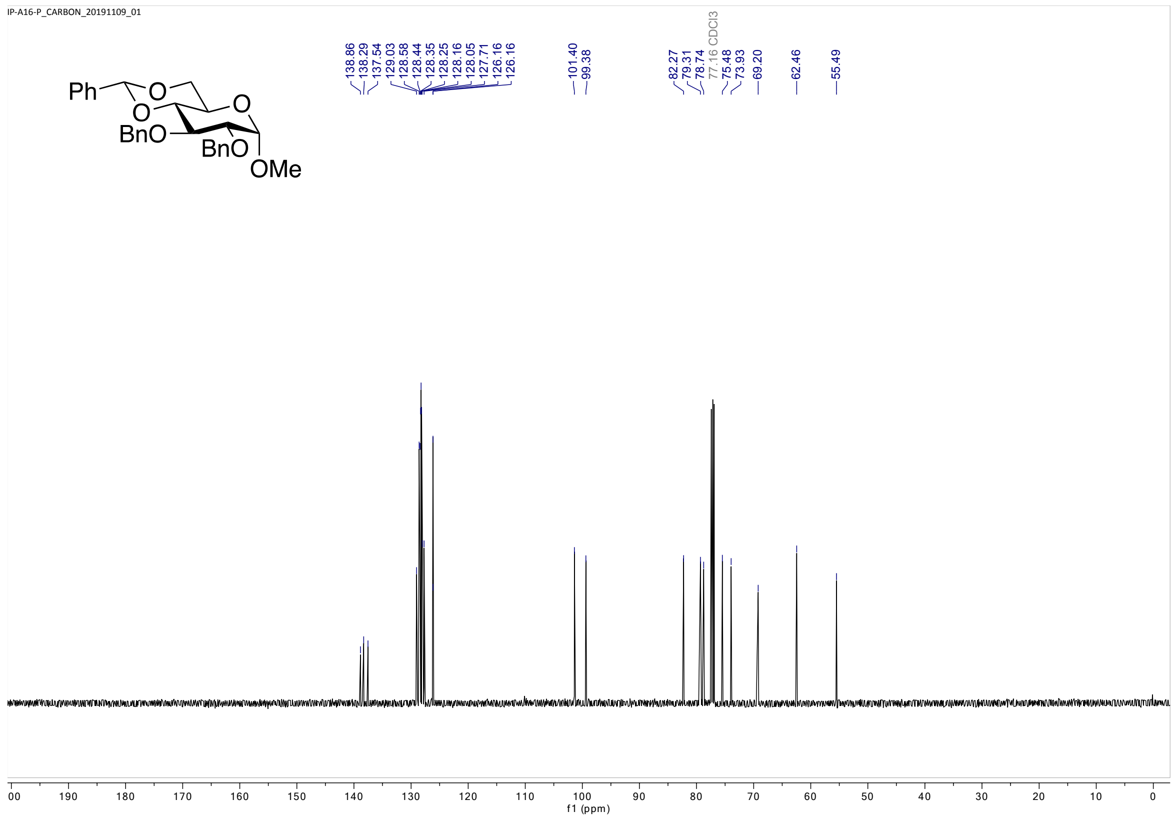

^{1}H NMR (500 MHz, Chloroform-*d*) 3b

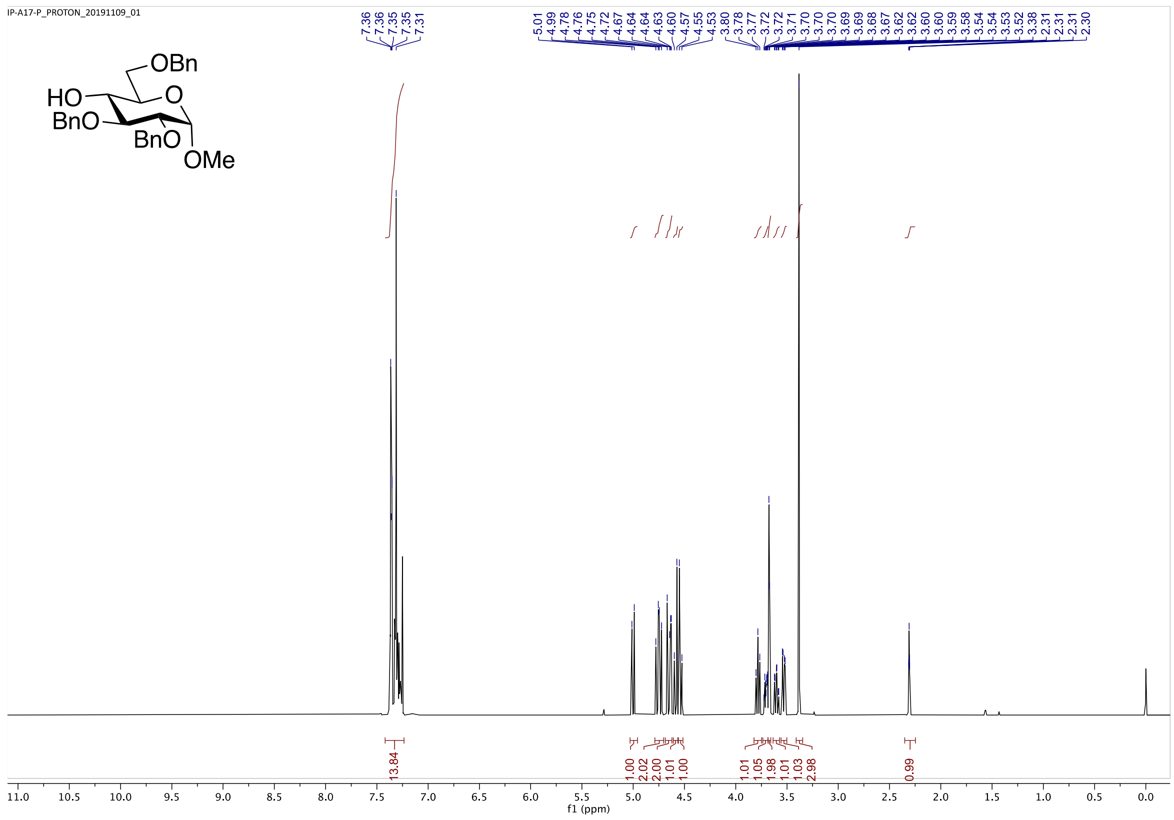

^{13}C NMR (126 MHz, Chloroform-*d*) 3b

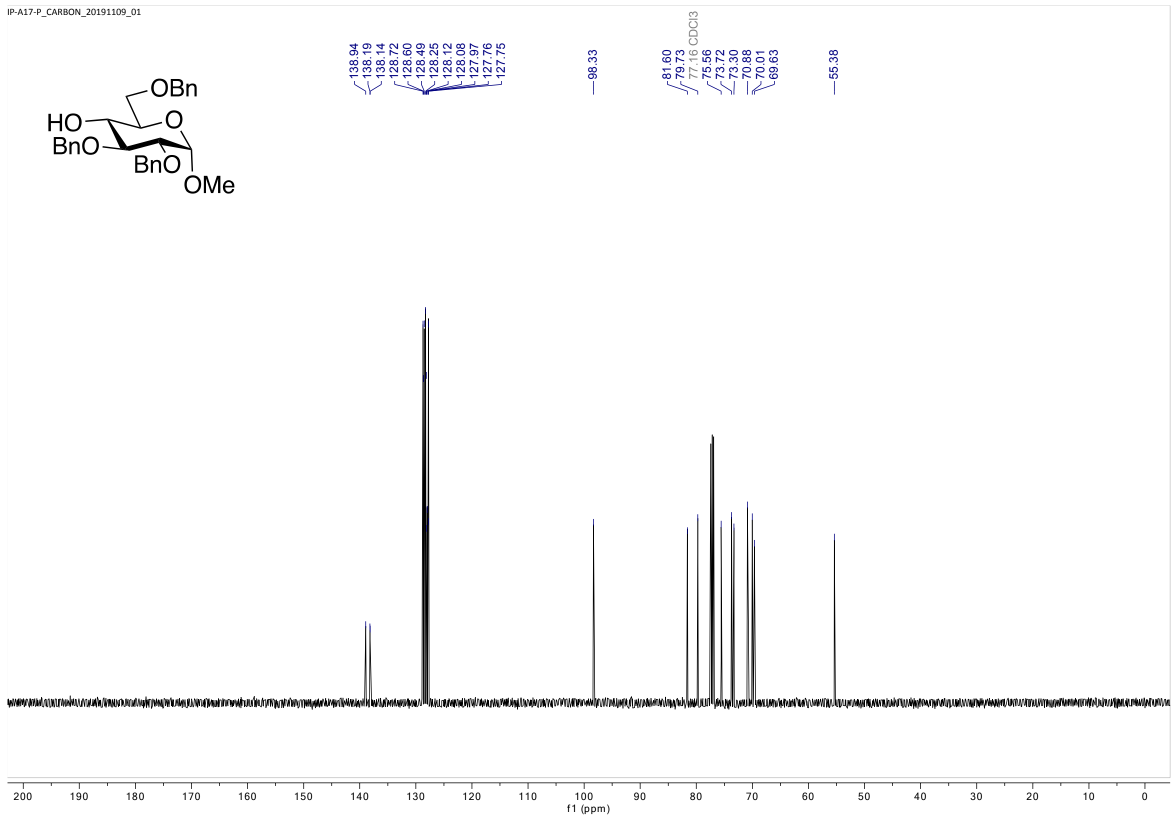

^{1}H NMR (500 MHz, Chloroform-*d*) S40

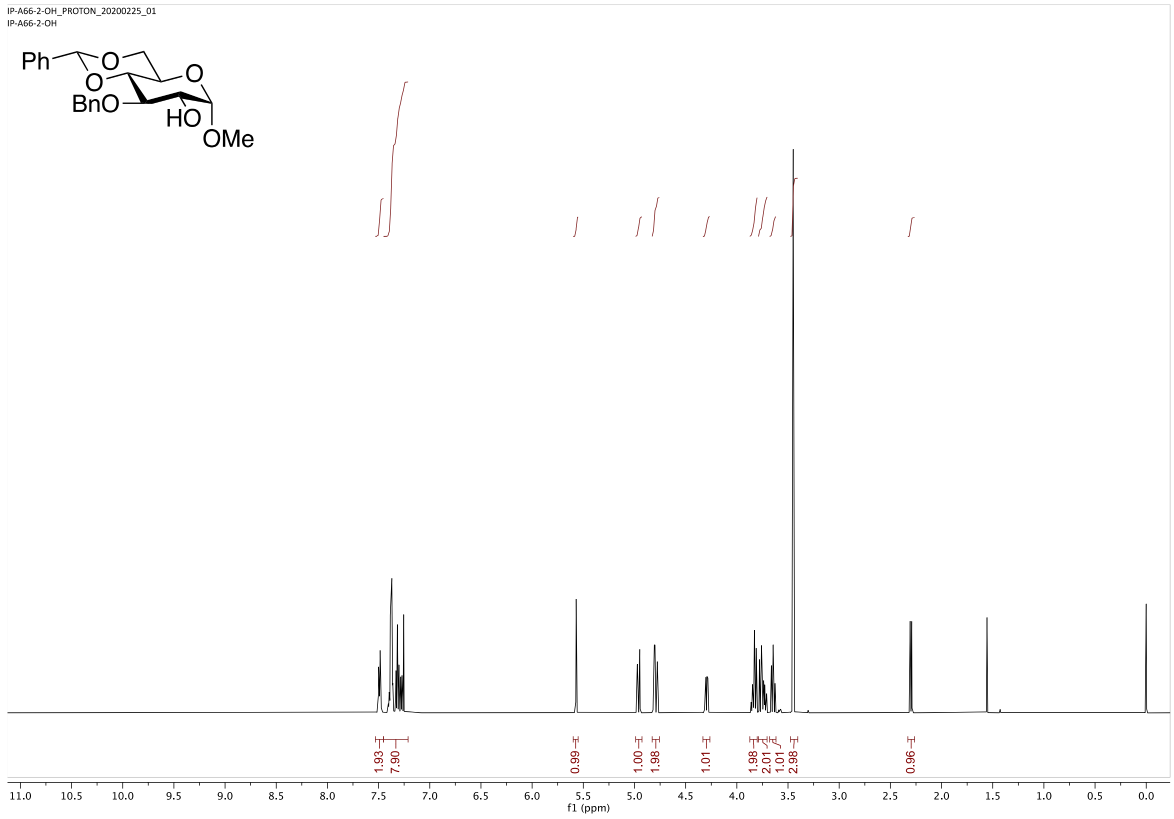

^{13}C NMR (126 MHz, Chloroform-*d*) S40

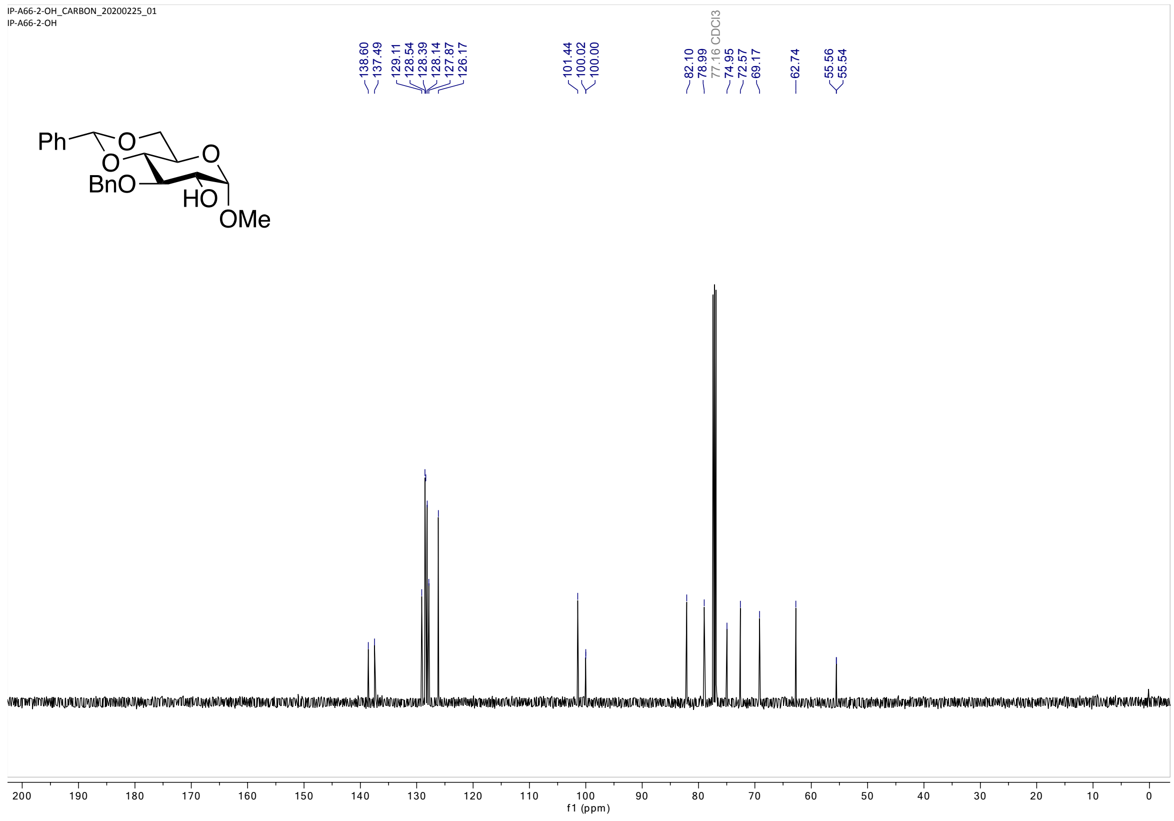

^{1}H NMR (400 MHz, Chloroform-*d*) 3d

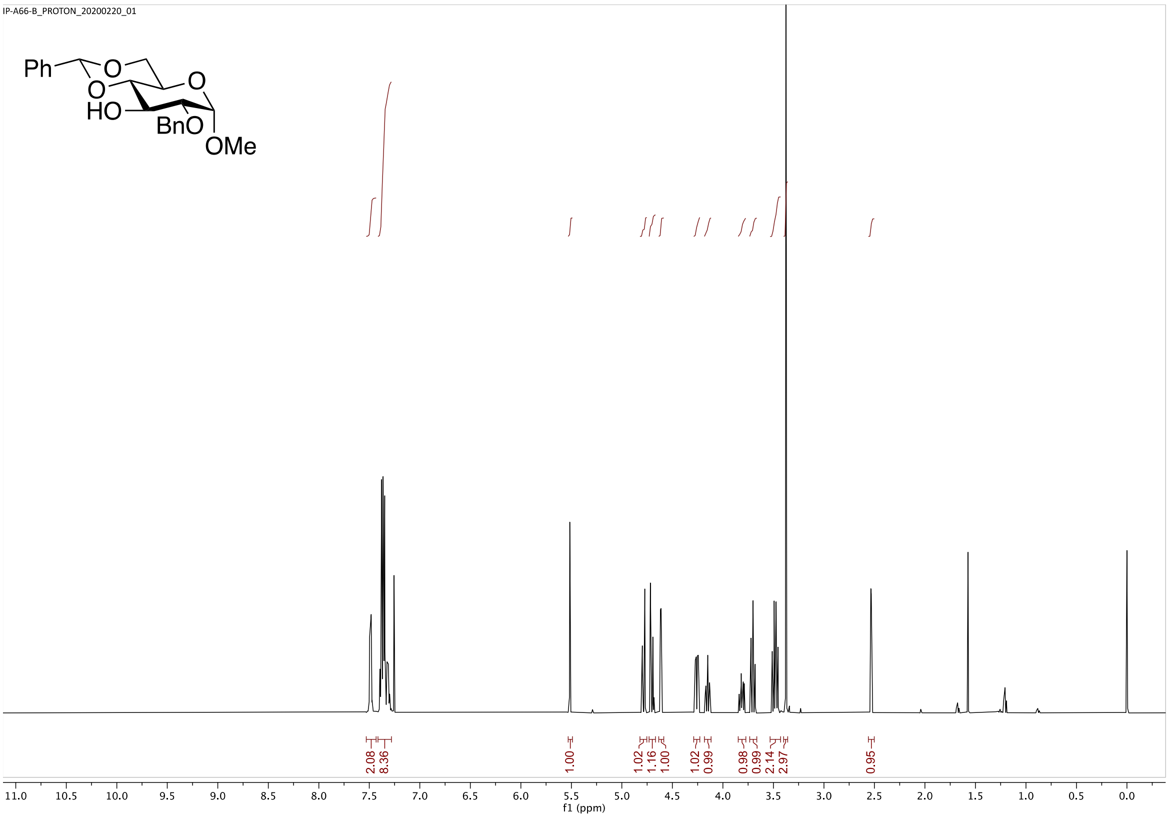

^{13}C NMR (101 MHz, Chloroform-*d*) 3d

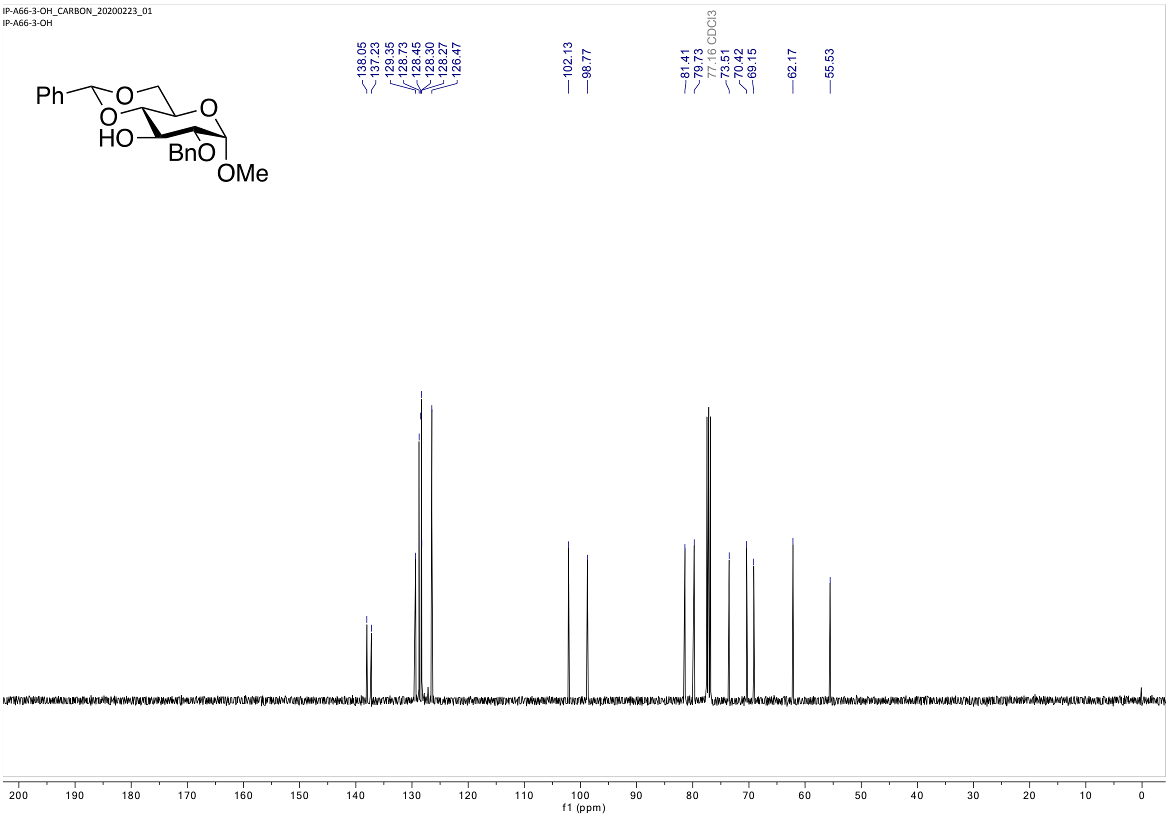

^{1}H NMR (600 MHz, Chloroform-*d*) S41 and S42

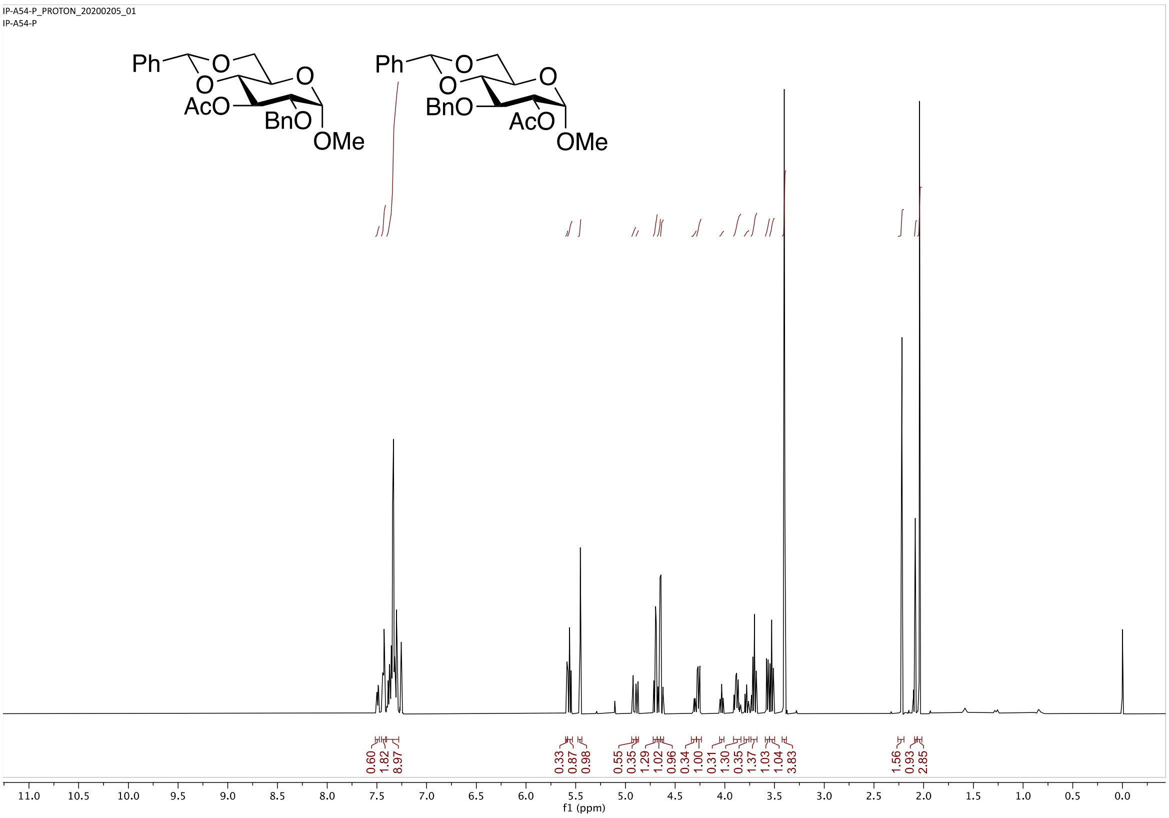

^{13}C NMR (151 MHz, Chloroform-*d*) S41 and S42

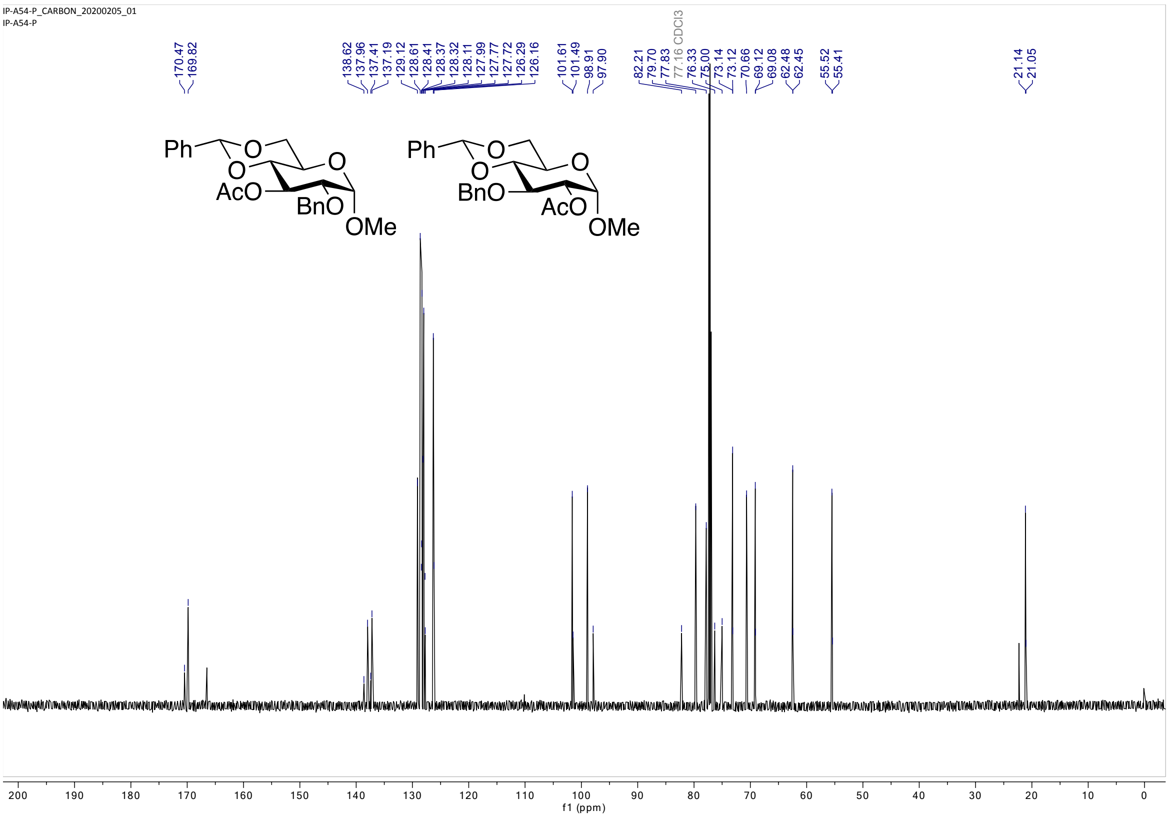

^{1}H NMR (500 MHz, Chloroform-*d*) S43

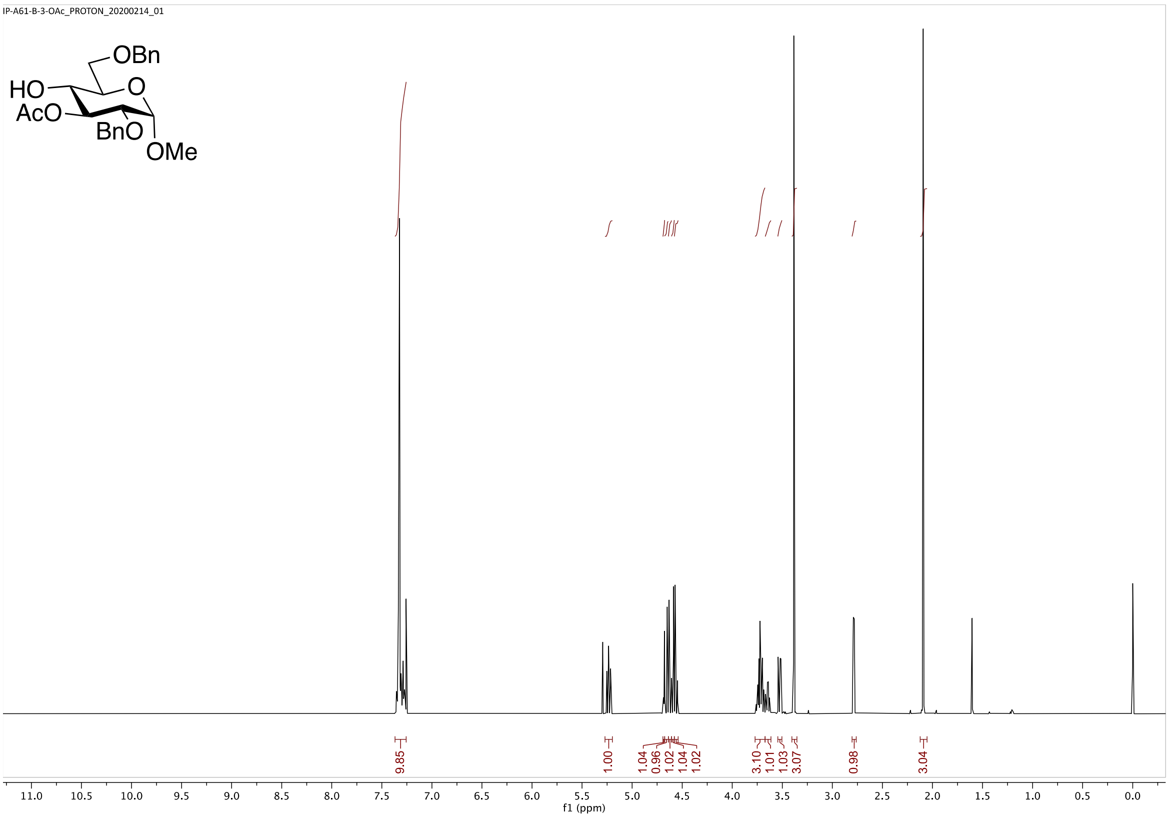

^{13}C NMR (126 MHz, Chloroform-*d*) S43

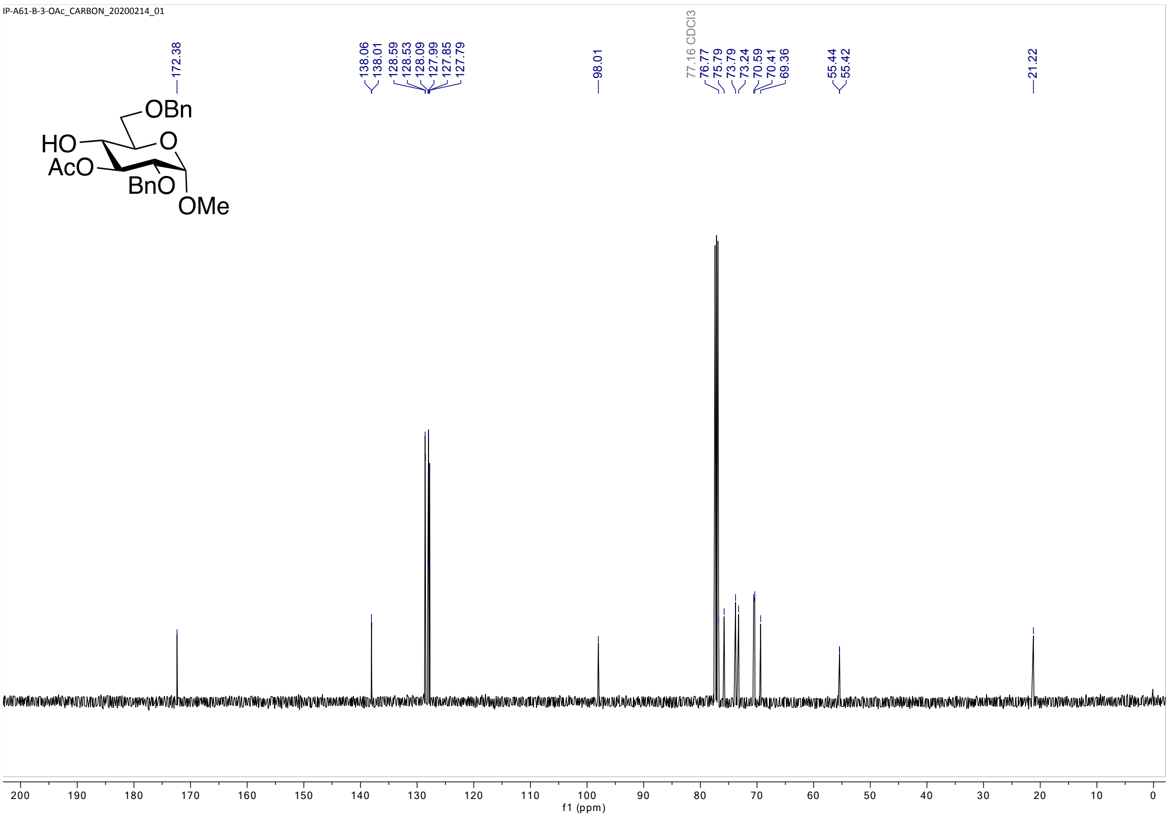

COSY NMR (500 MHz, Chloroform-*d*) S43

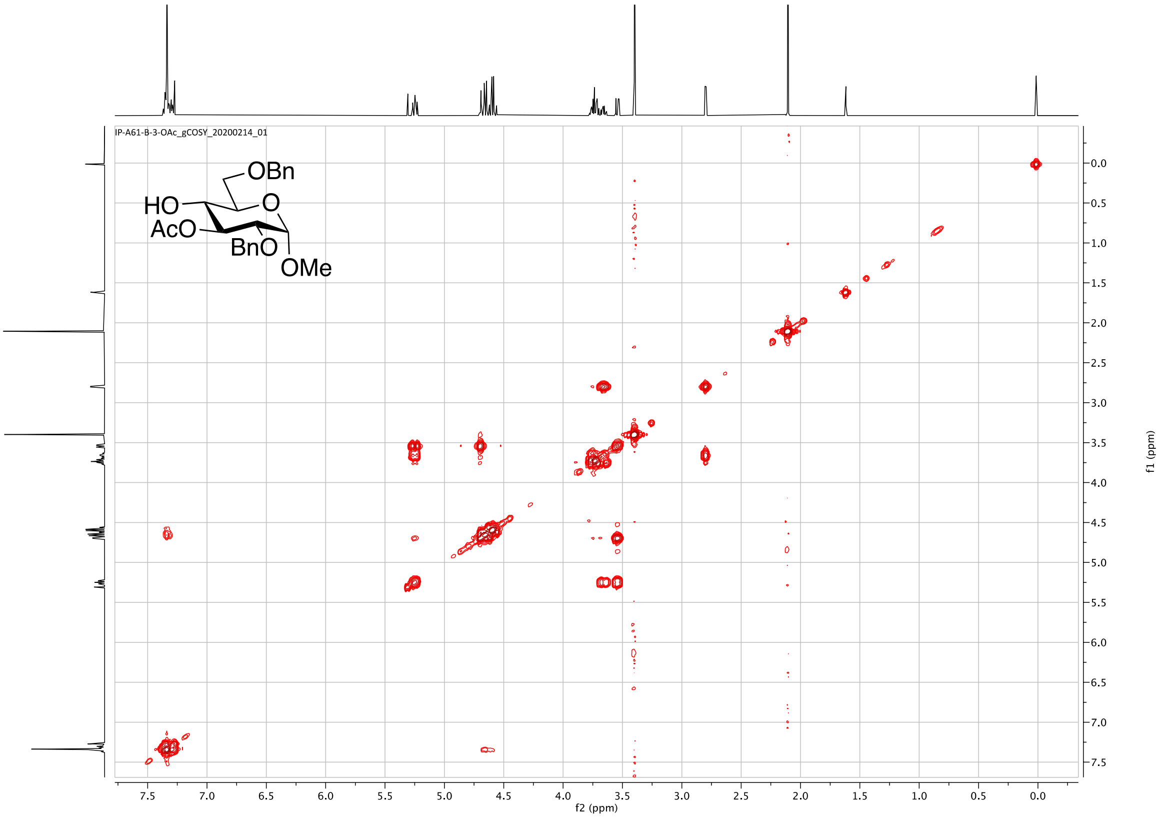

HSQC NMR (500 MHz x 126 MHz, Chloroform-*d*) S43

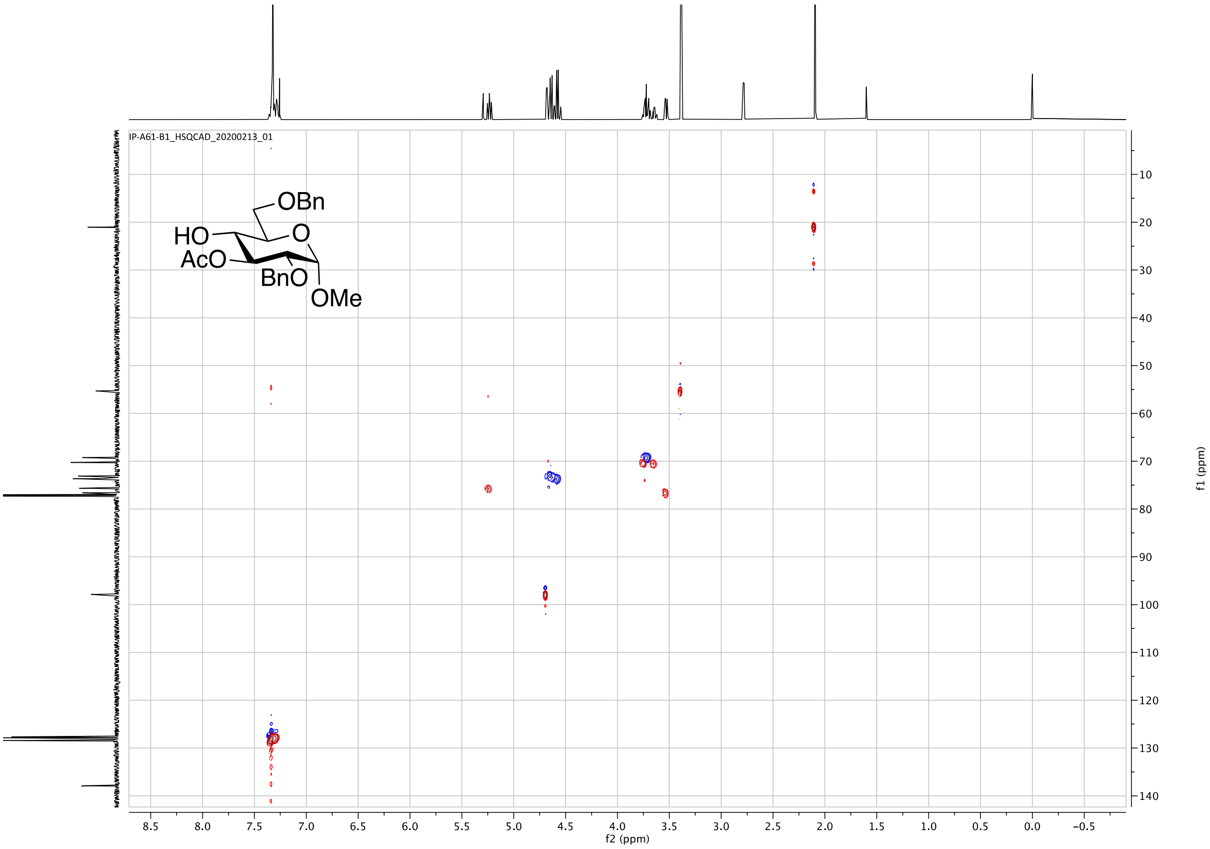

HMBC NMR (500 MHz x 126 MHz, Chloroform-*d*) S43

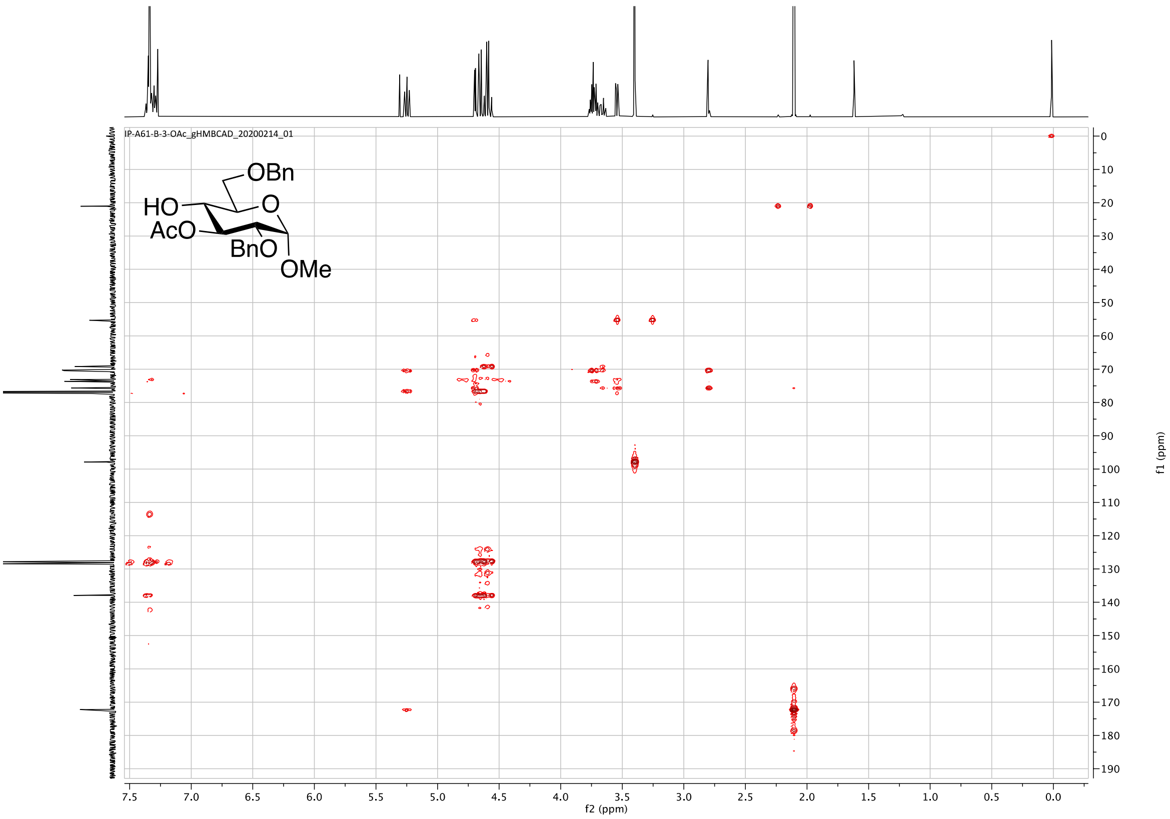

^{1}H NMR (500 MHz, Chloroform-*d*) S44

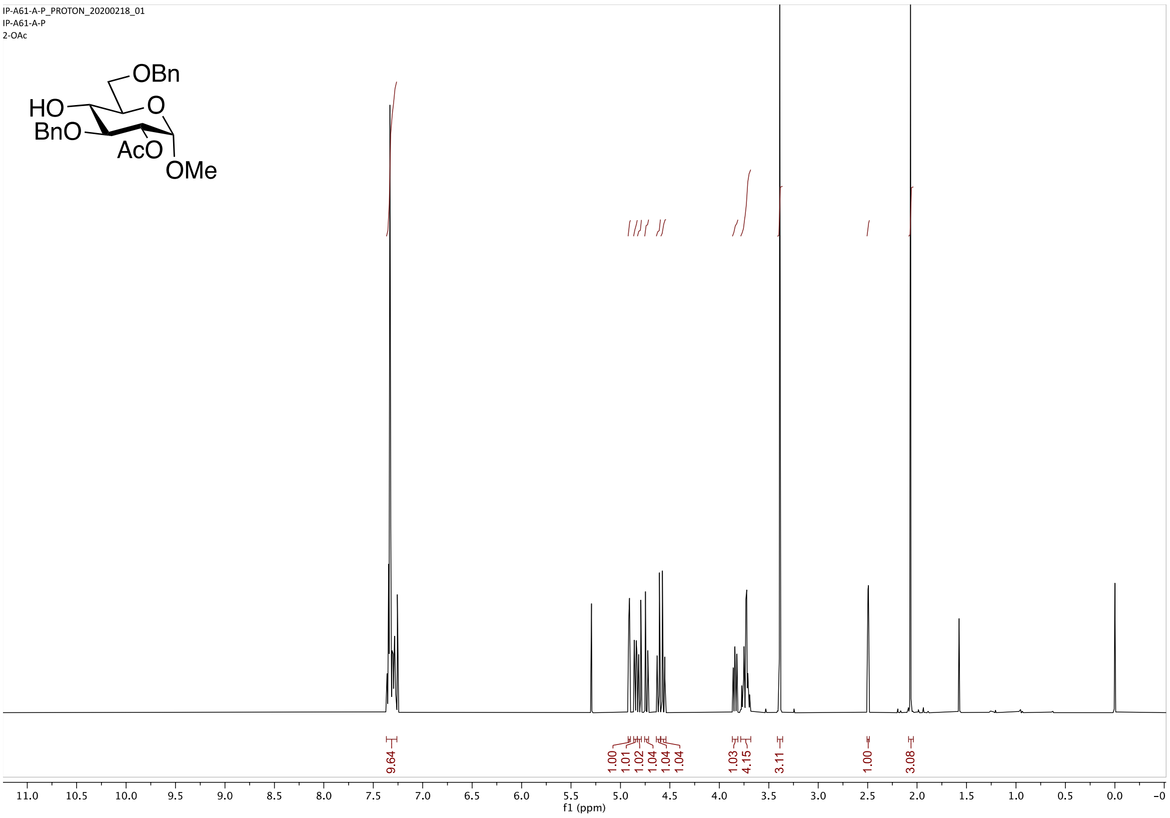

^{13}C NMR (126 MHz, Chloroform-*d*) S44

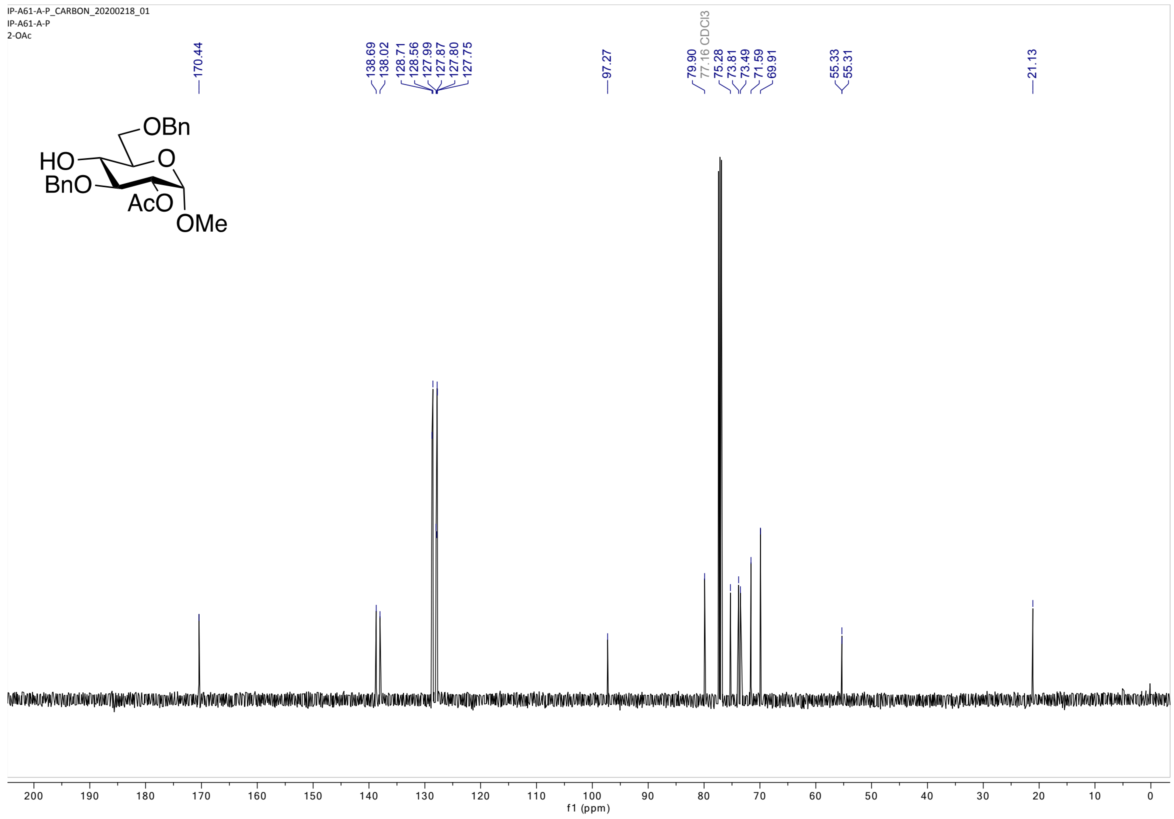

^{1}H NMR (500 MHz, Chloroform-*d*) 3c

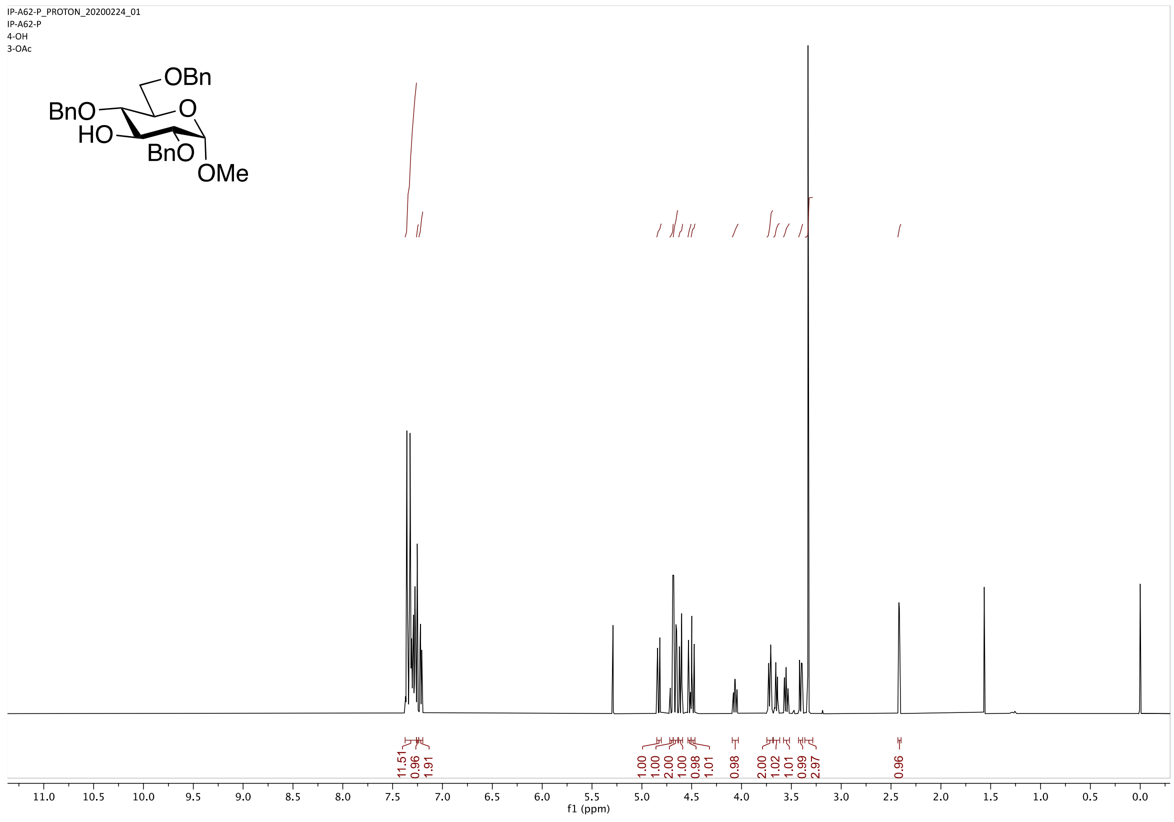

^{13}C NMR (126 MHz, Chloroform-*d*) 3c

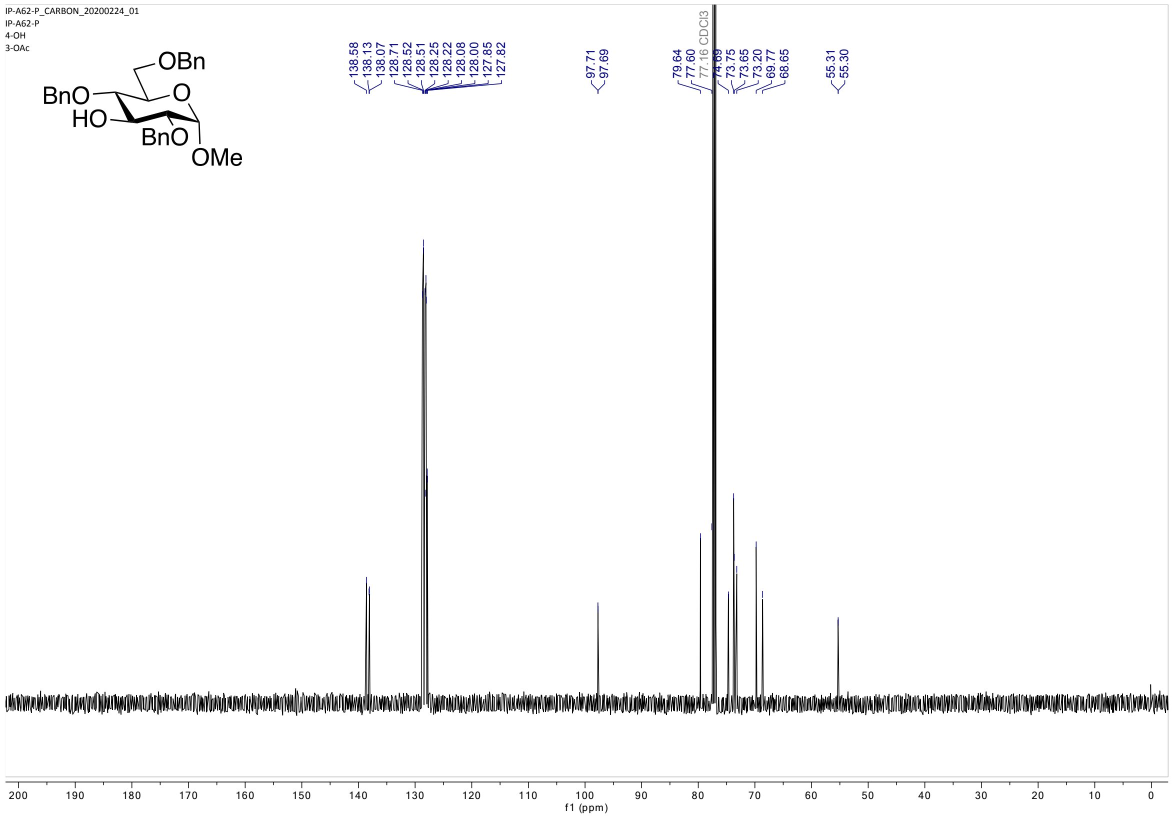

^{1}H NMR (500 MHz, Chloroform-*d*) 3i

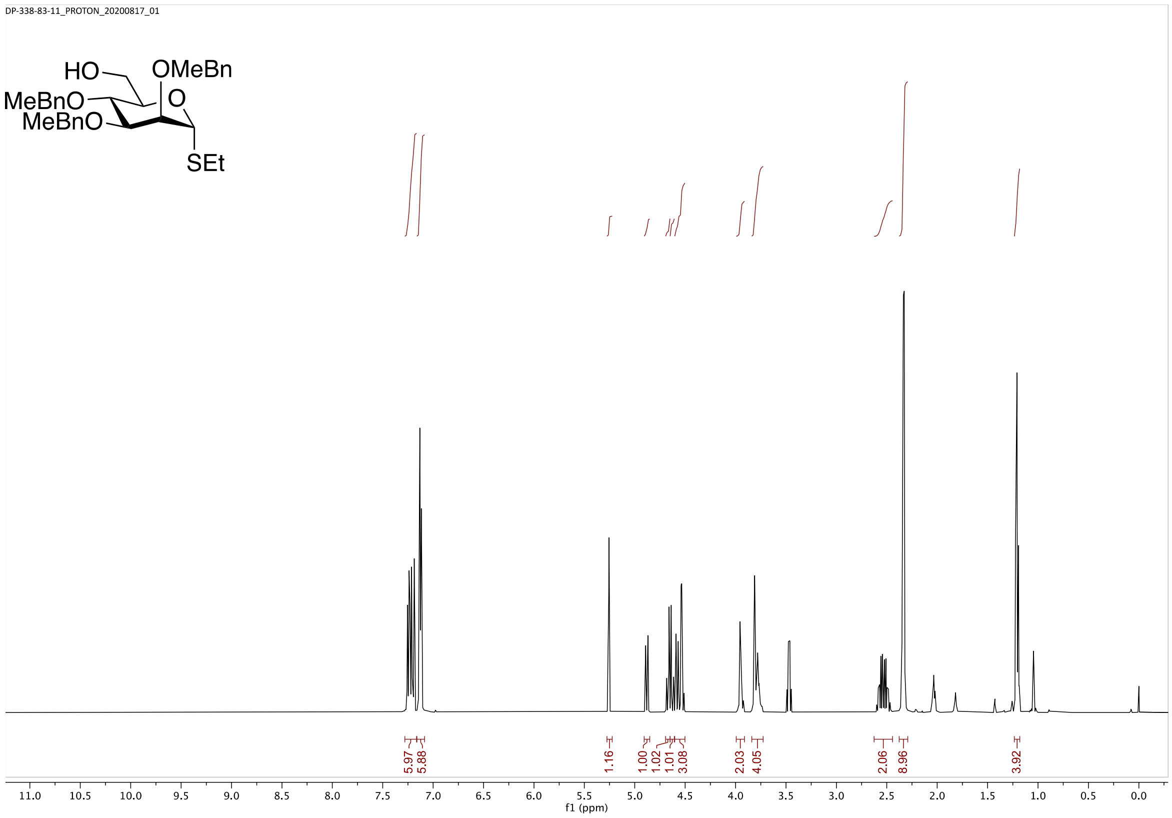

^{13}C NMR (126 MHz, Chloroform-*d*) 3i

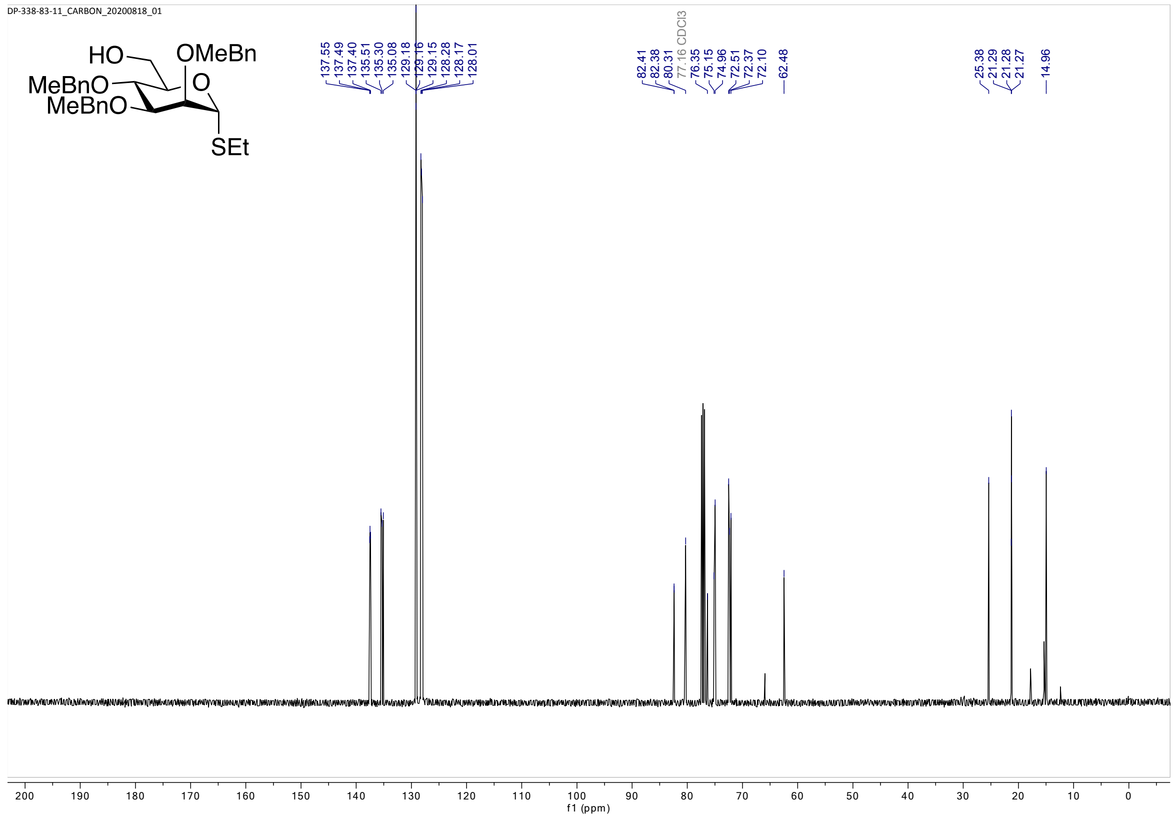

COSY NMR (500 MHz, Chloroform-*d*) 3i

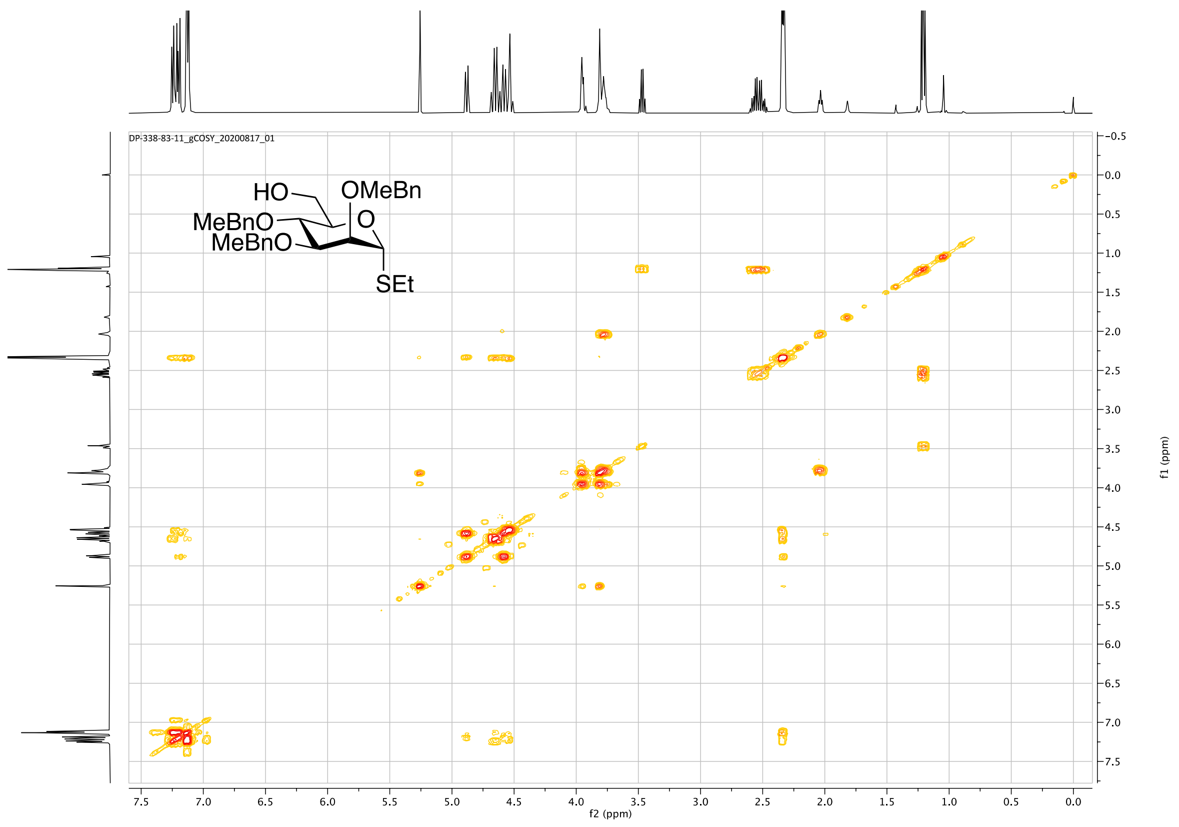

HSQC NMR (500 MHz x 126 MHz, Chloroform-*d*) 3i

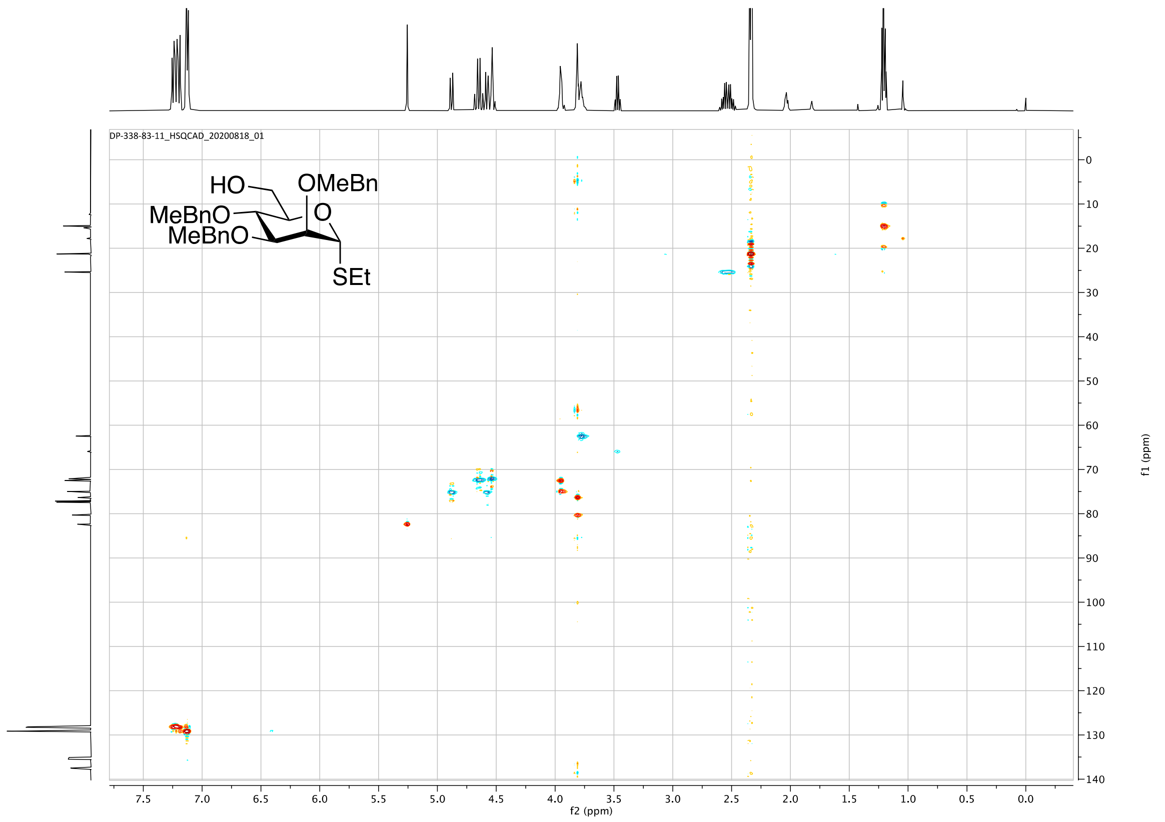

HMBC NMR (500 MHz x 126 MHz, Chloroform-*d*) 3i

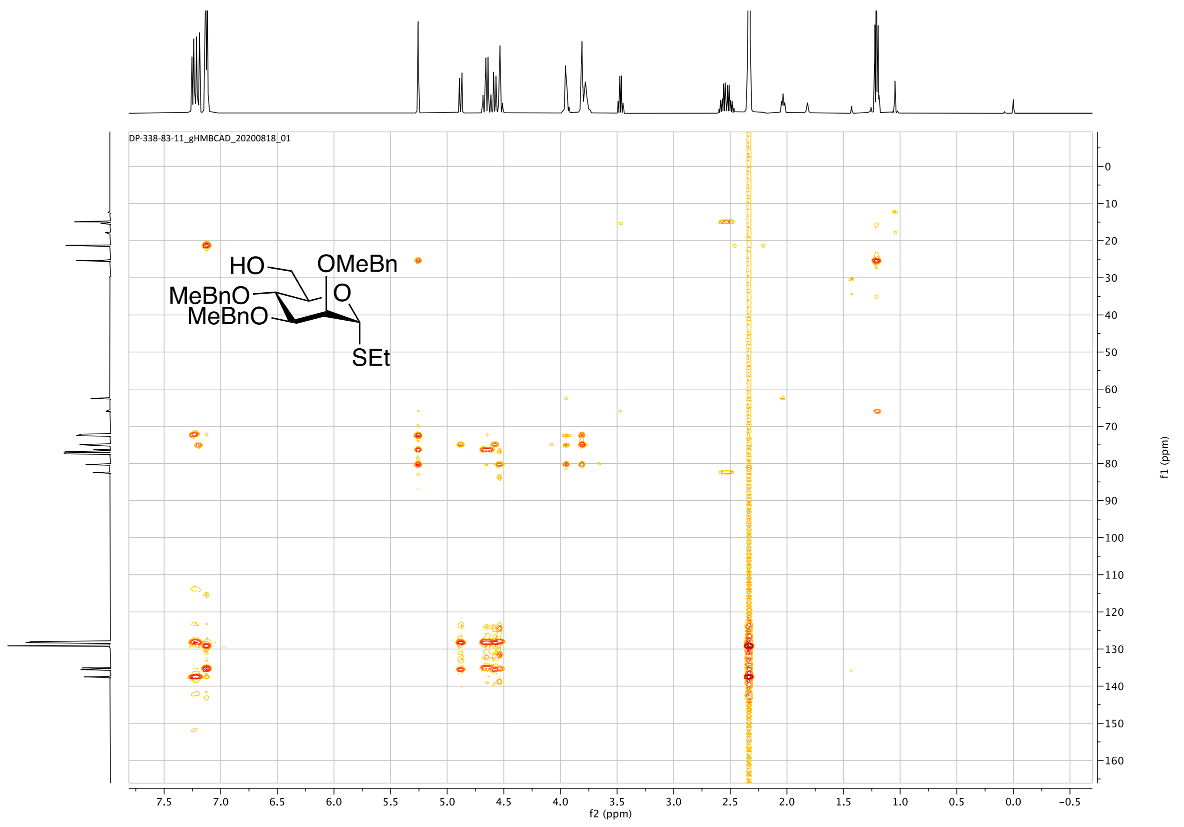

^{1}H NMR (500 MHz, Chloroform-*d*) 3j

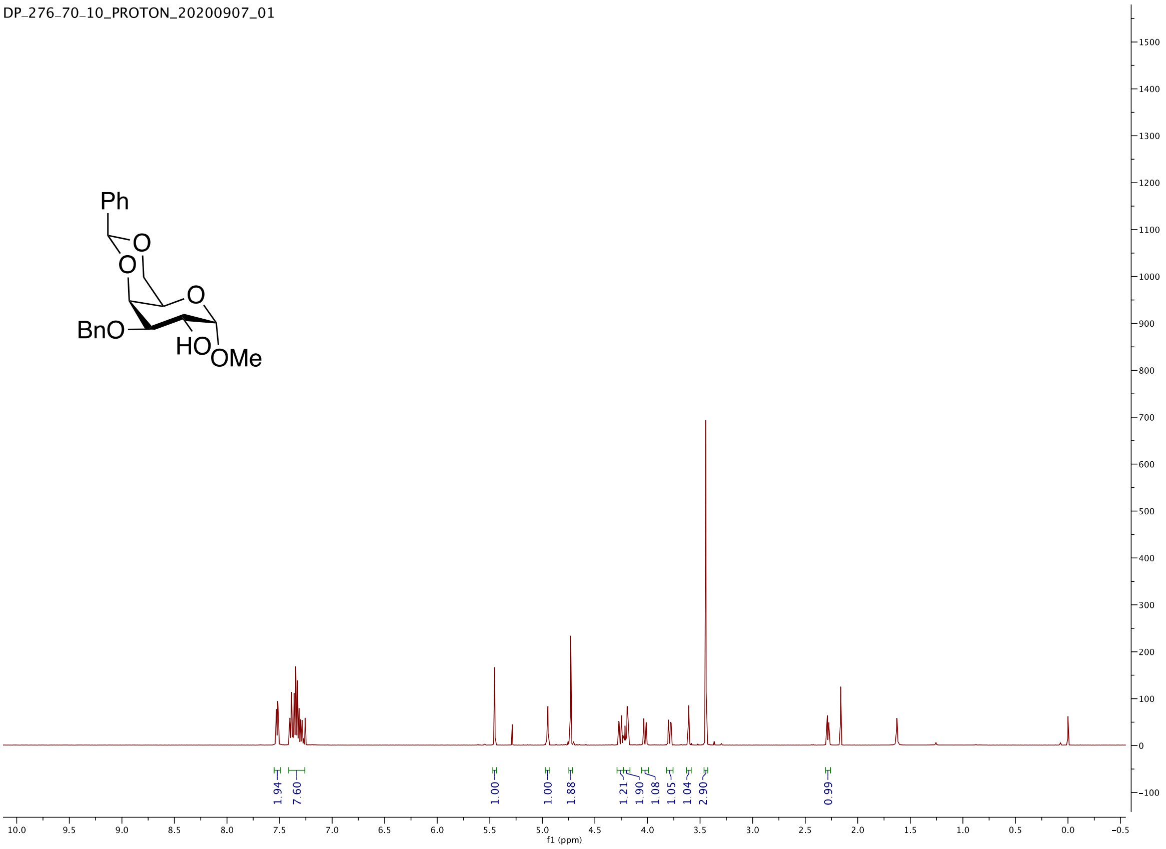

^{13}C NMR (126 MHz, Chloroform-*d*) 3j

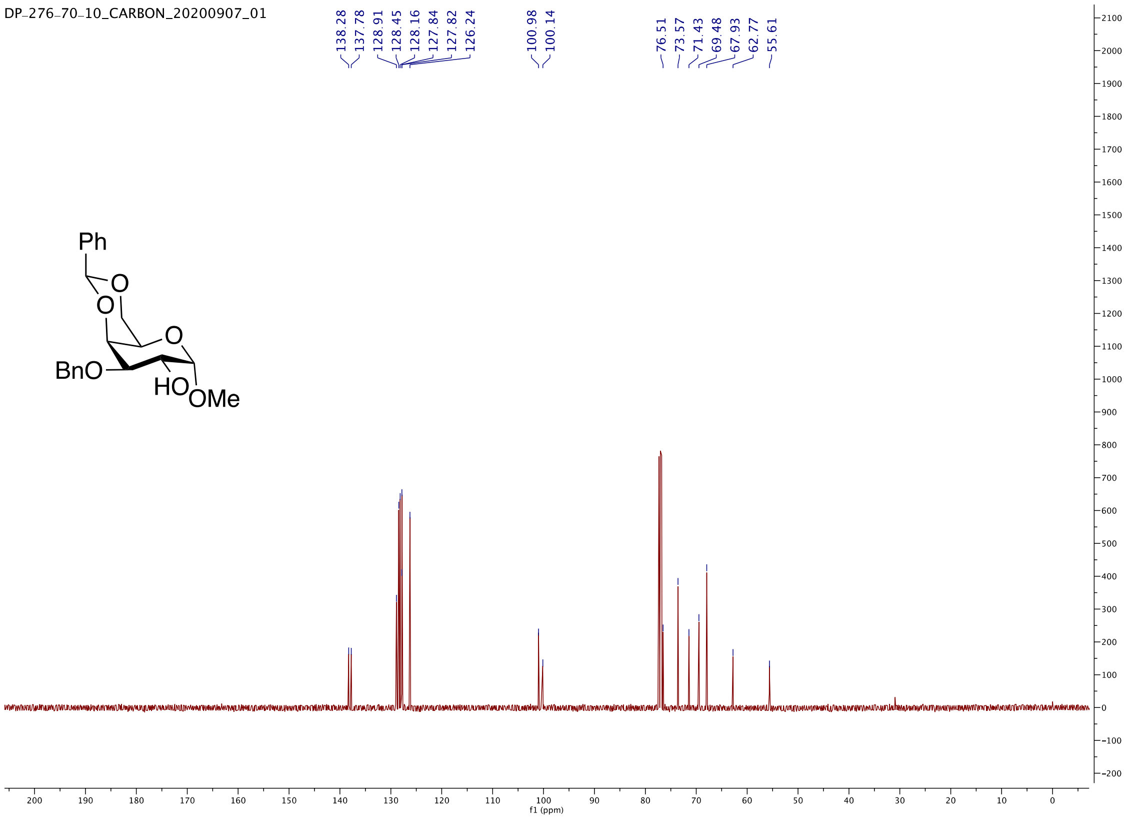

^{1}H NMR (500 MHz, Chloroform-*d*) S46

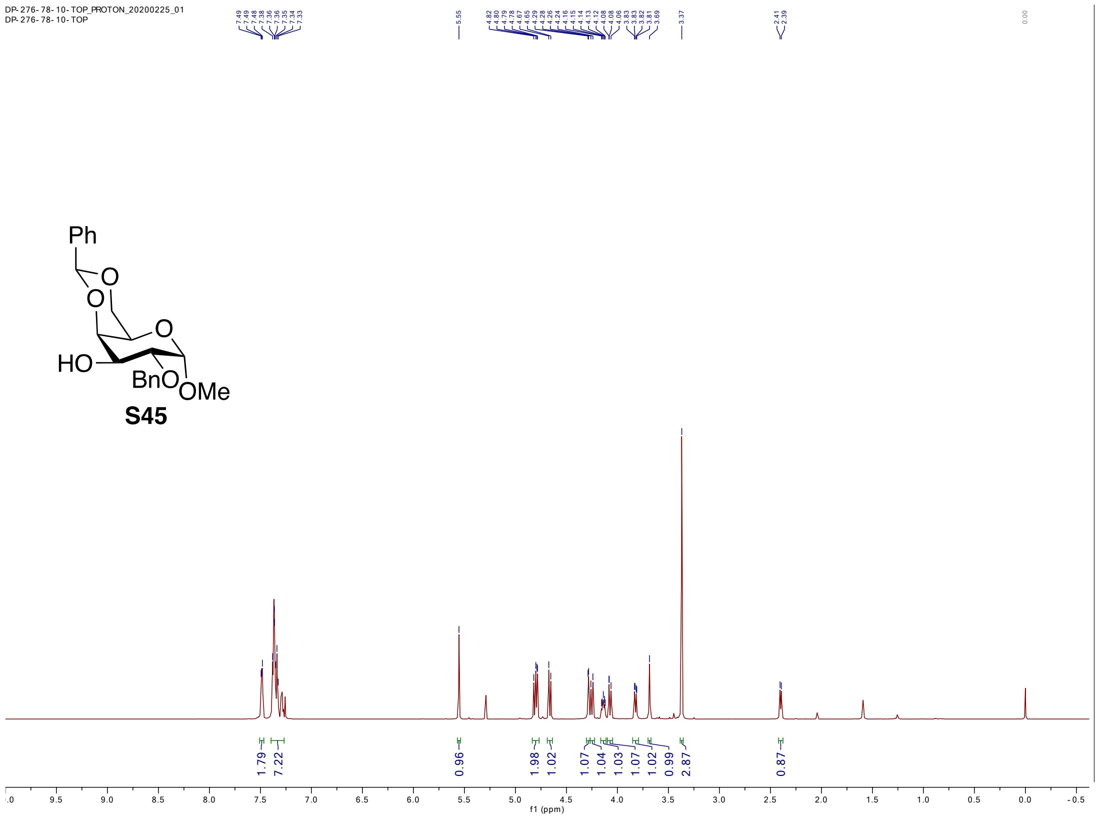

^{13}C NMR (126 MHz, Chloroform-*d*) S46

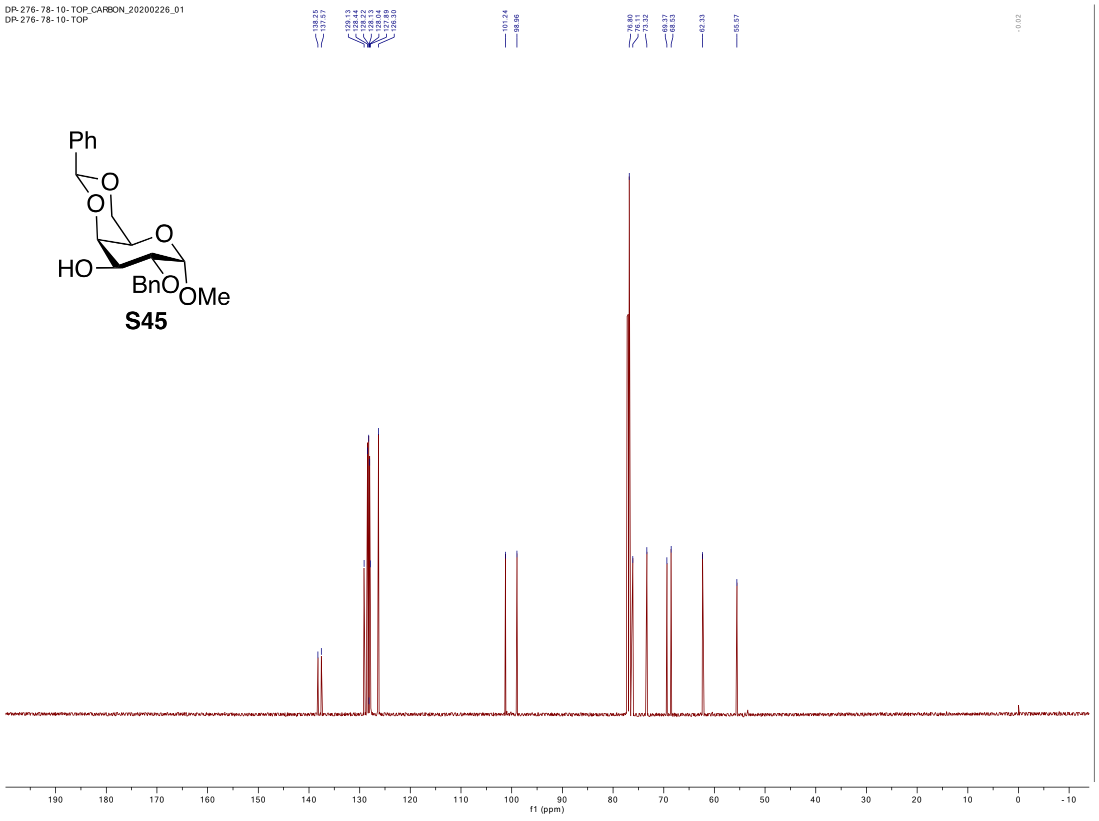

^{1}H NMR (500 MHz, Chloroform-*d*) 2a

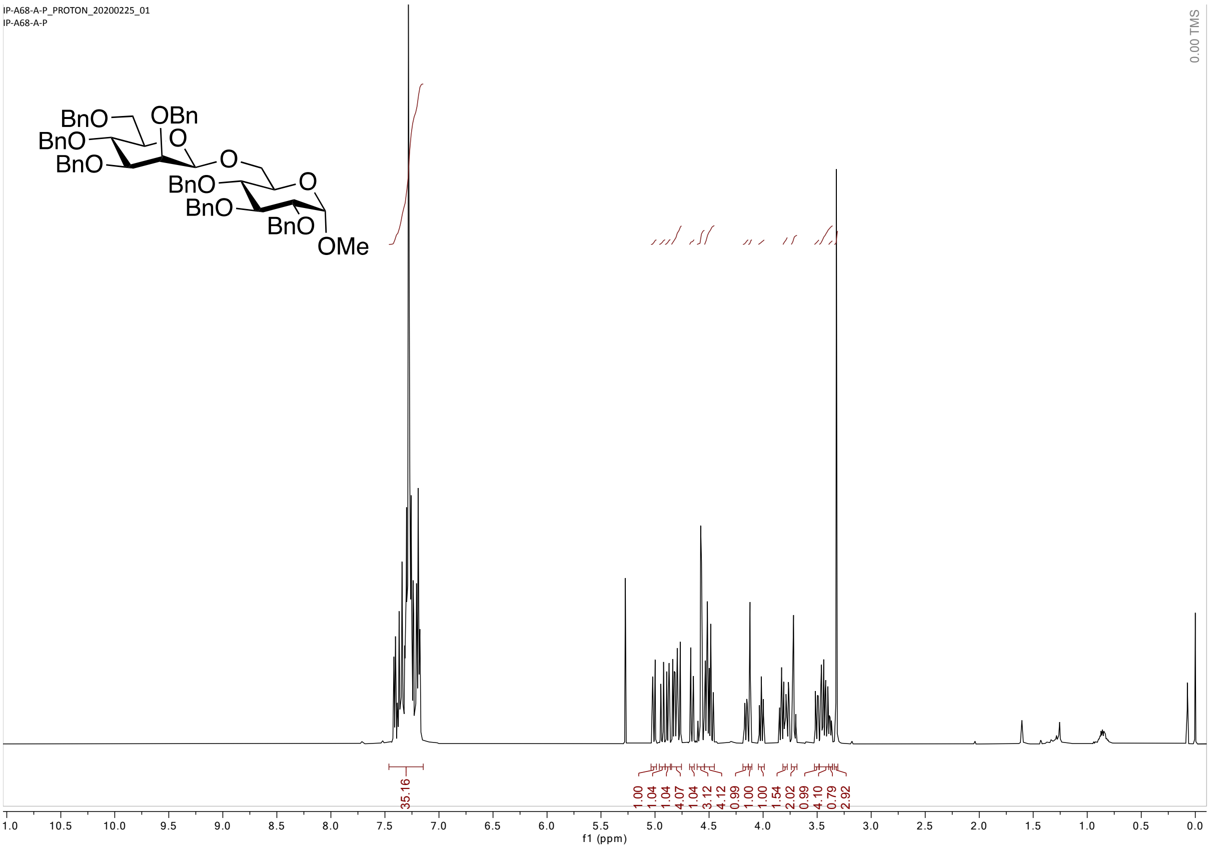

^{13}C NMR (126 MHz, Chloroform-*d*) 2a

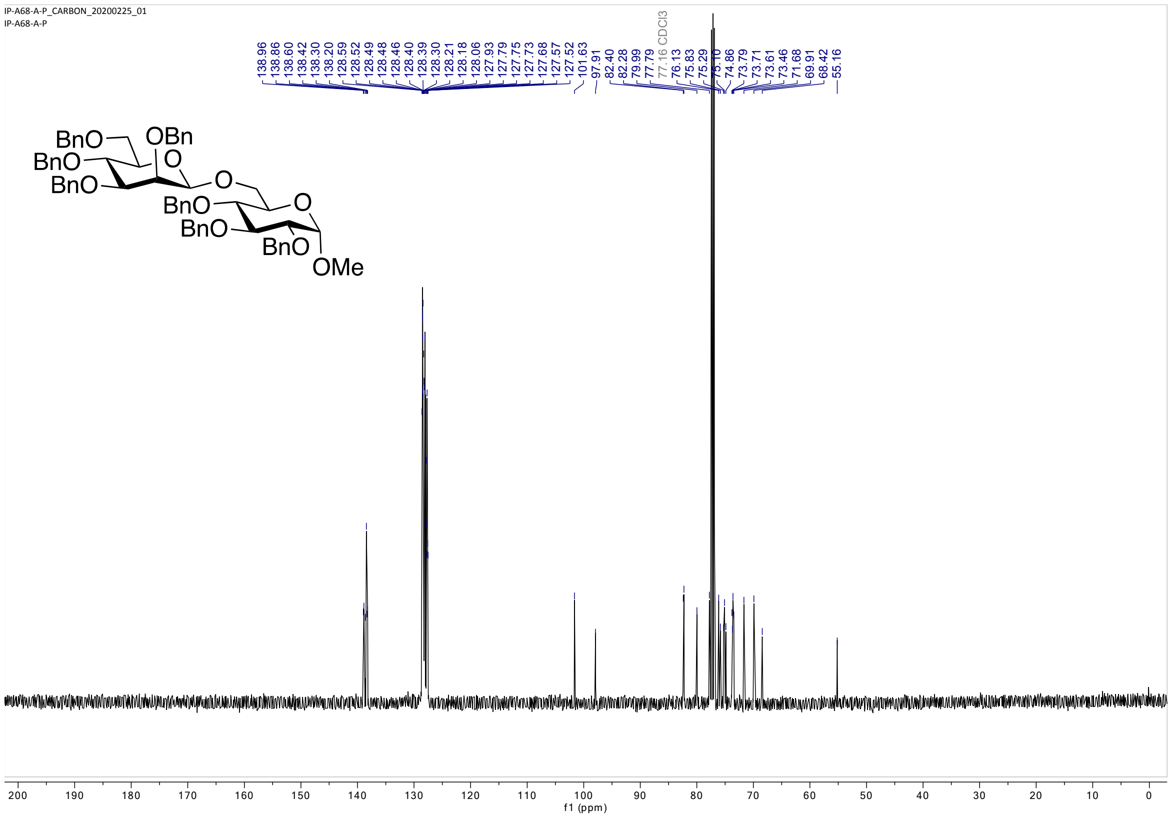

^{1}H NMR (600 MHz, Chloroform-*d*) 2b

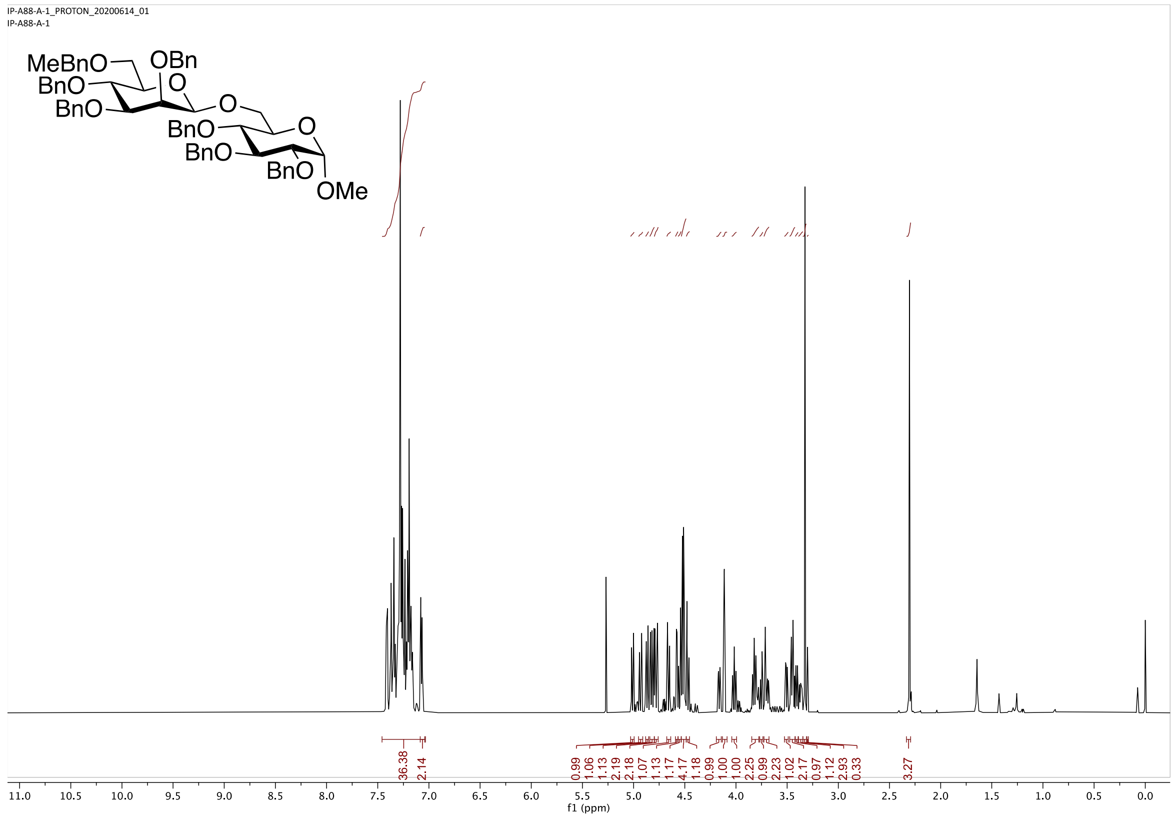

^{13}C NMR (151 MHz, Chloroform-*d*) 2b

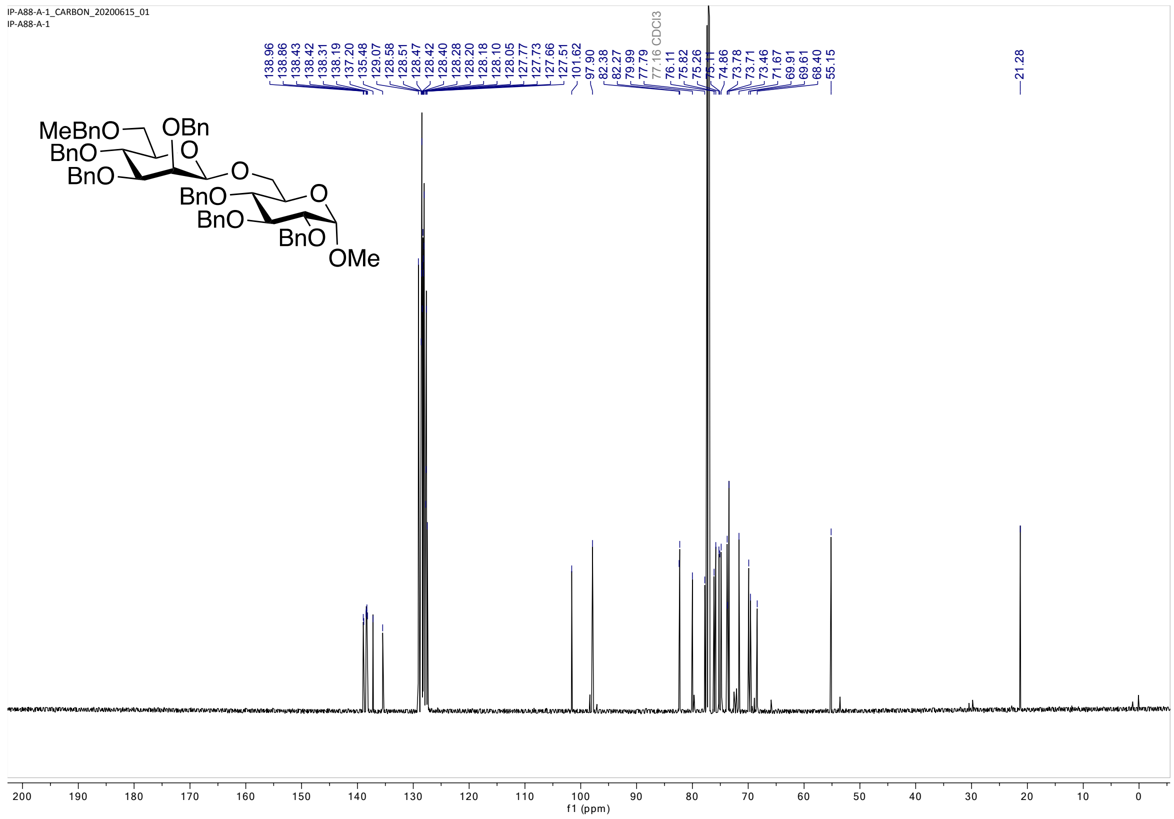

COSY NMR (600 MHz, Chloroform-*d*) 2b

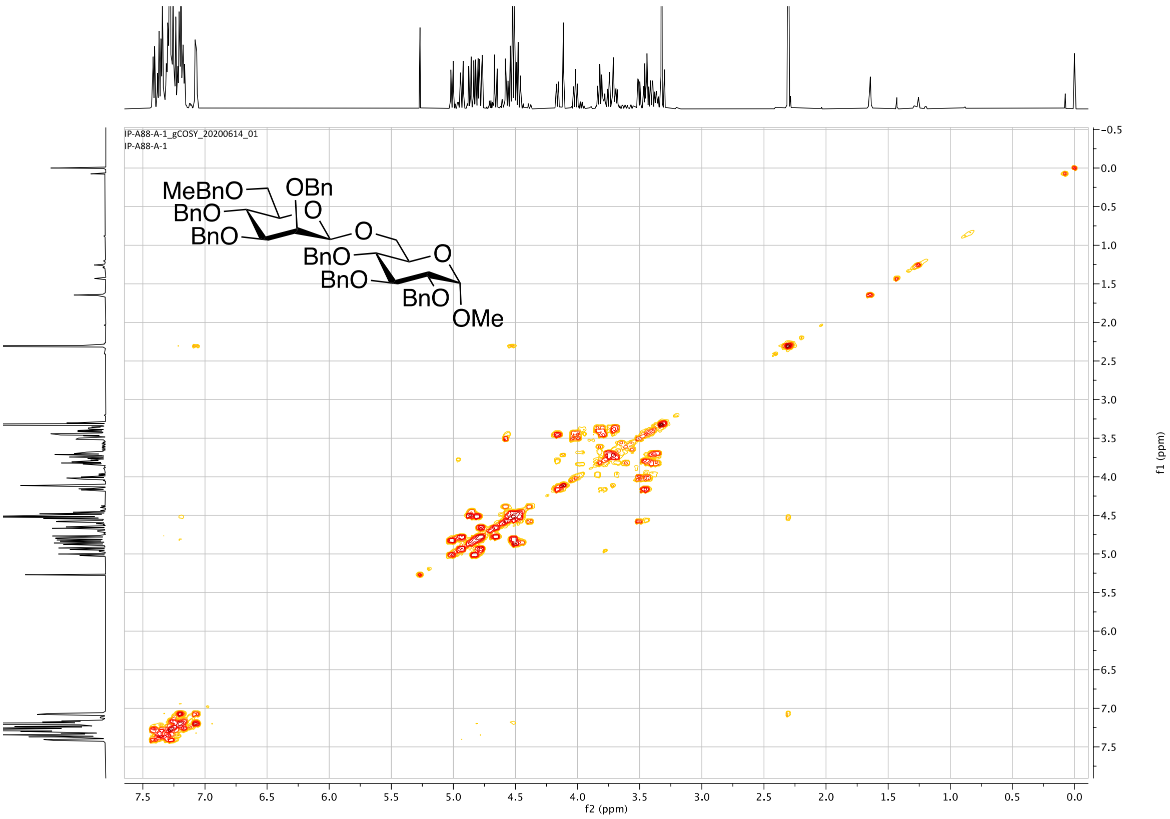

HSQC NMR (600 MHz x 151 MHz, Chloroform-*d*) 2b

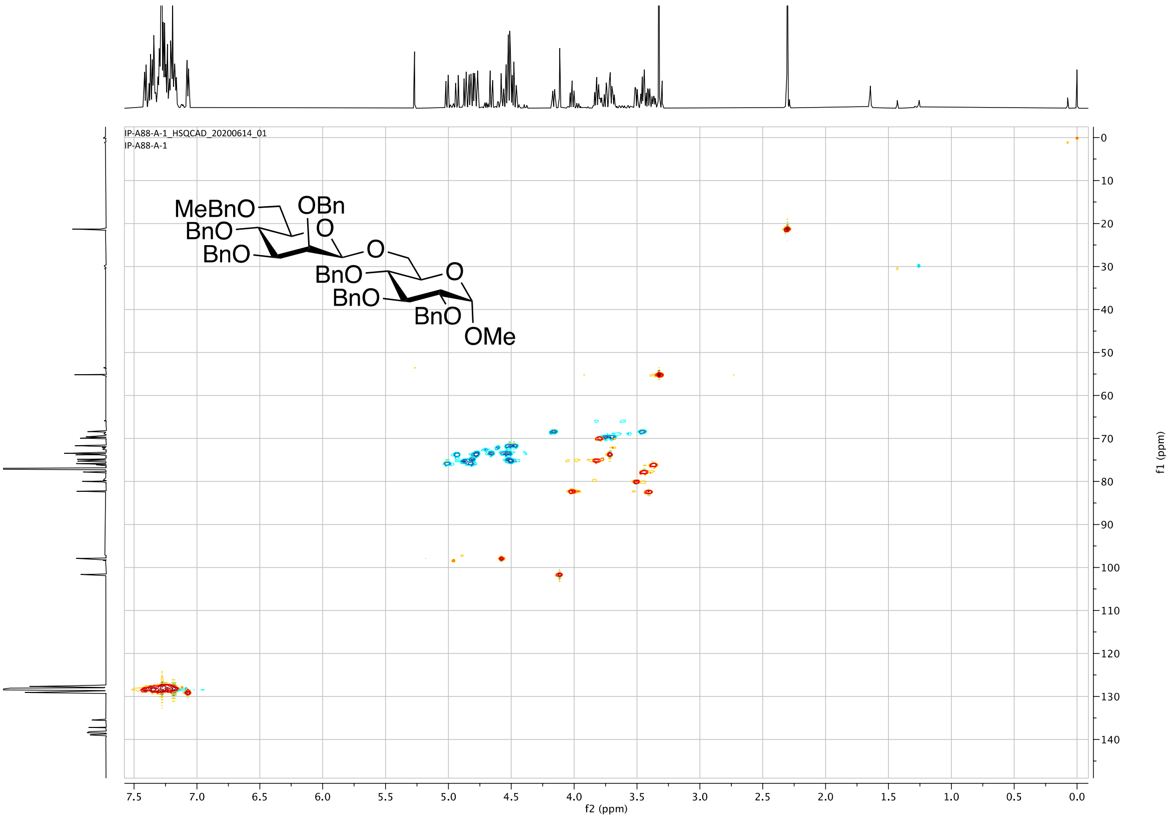

^{13}C- ^{1}H coupled HSQC NMR (600 MHz x 151 MHz, Chloroform-*d*) 2b

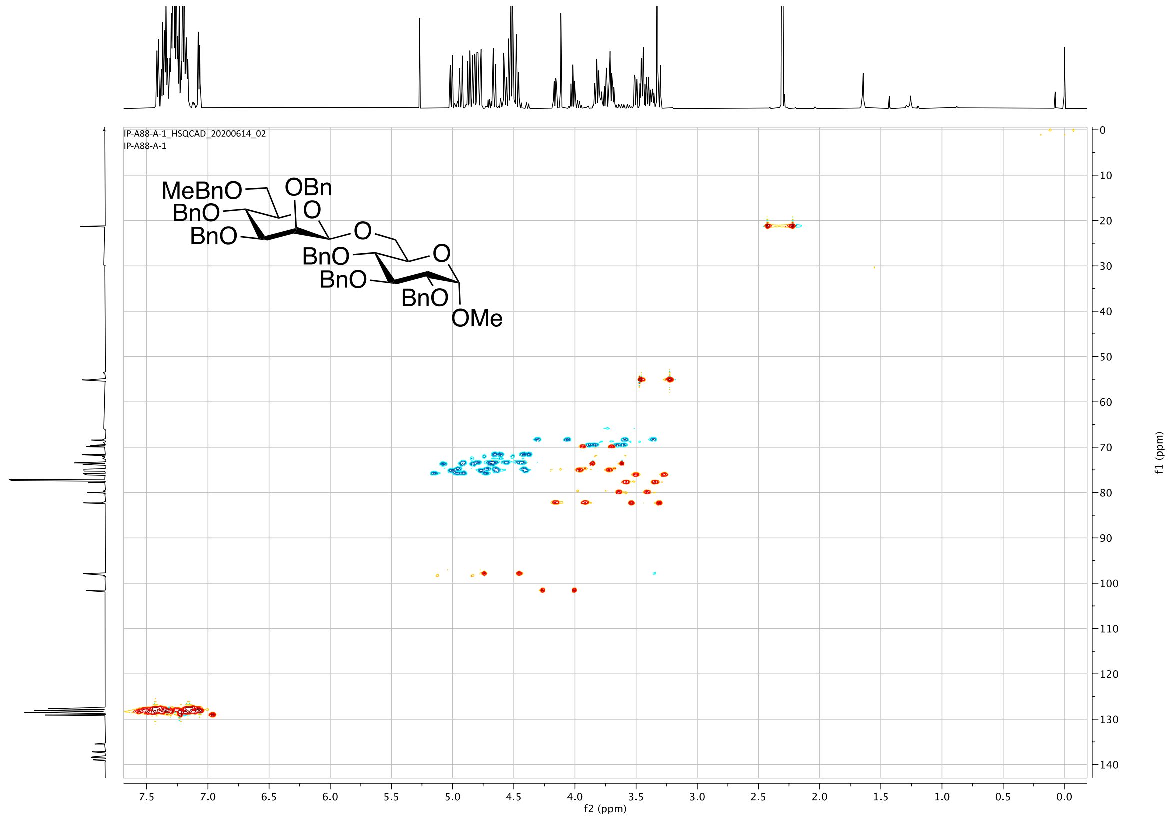

HMBC NMR (600 MHz x 151 MHz, Chloroform-*d*) 2b

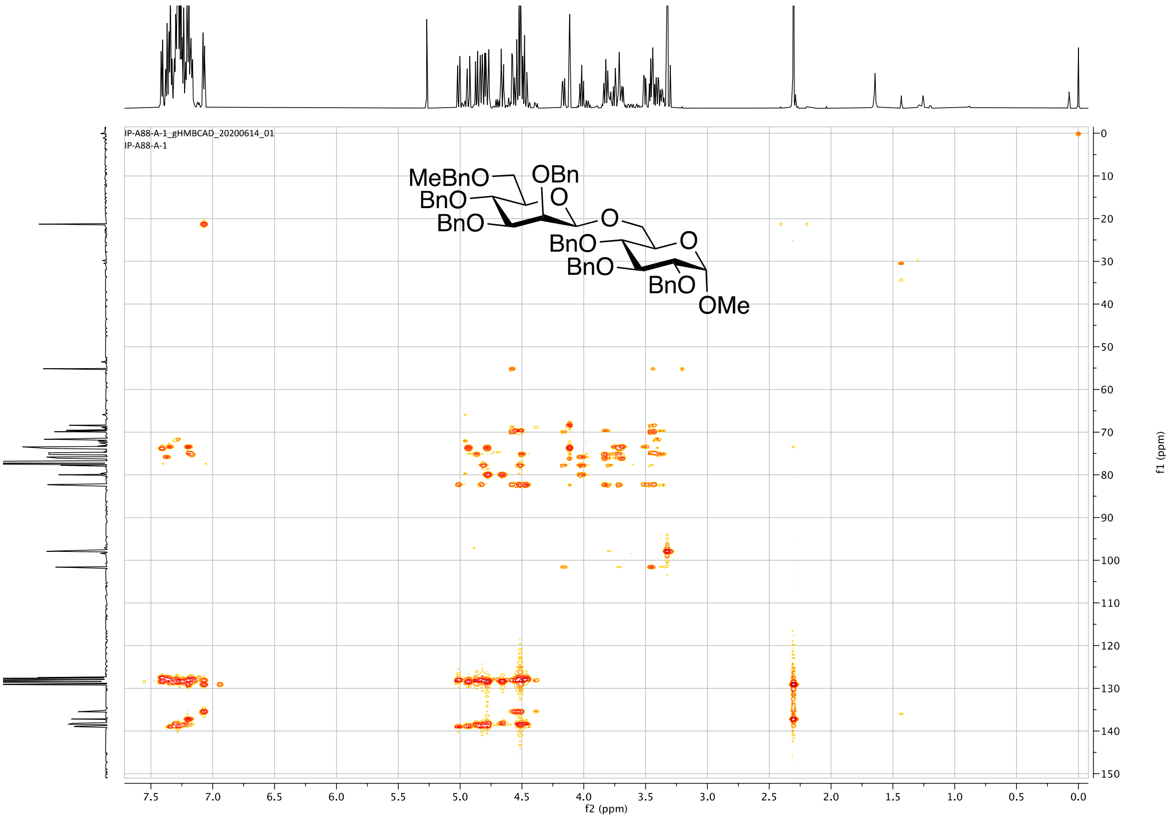

^{1}H NMR (500 MHz, Chloroform-*d*) 2c

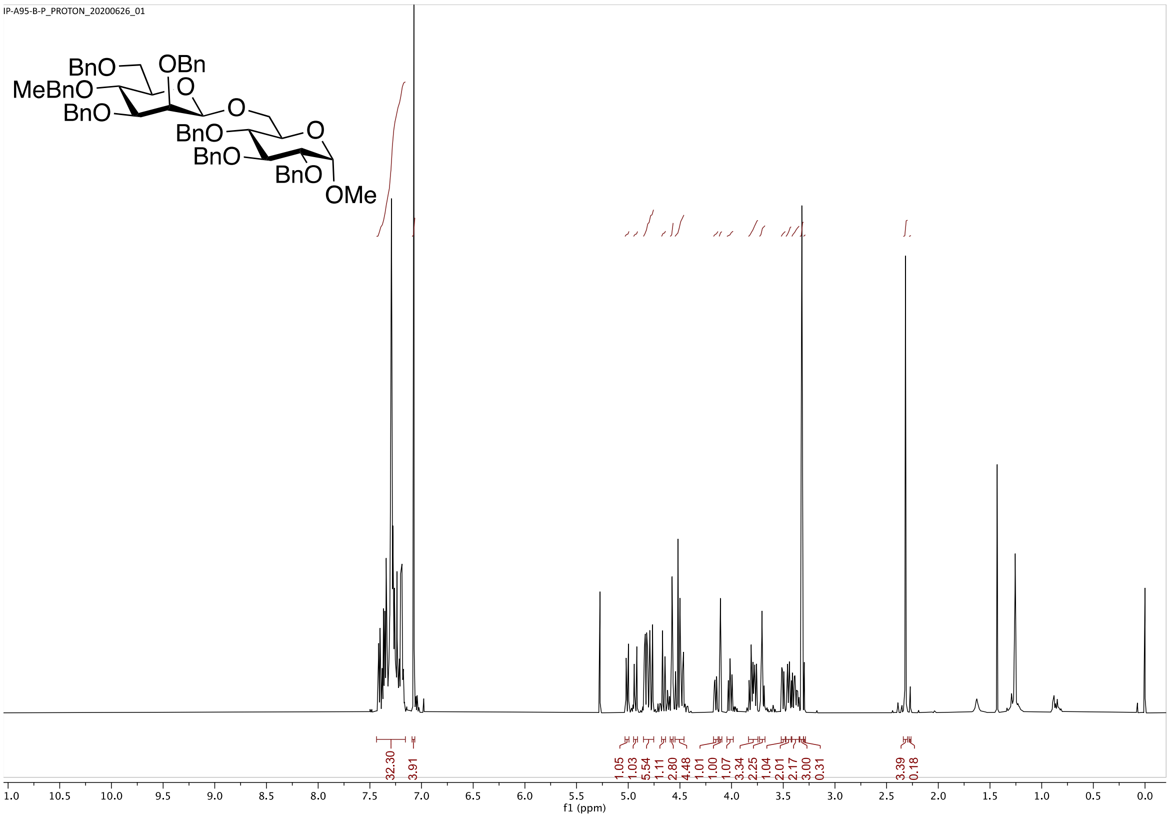

^{13}C NMR (126 MHz, Chloroform-*d*) 2c

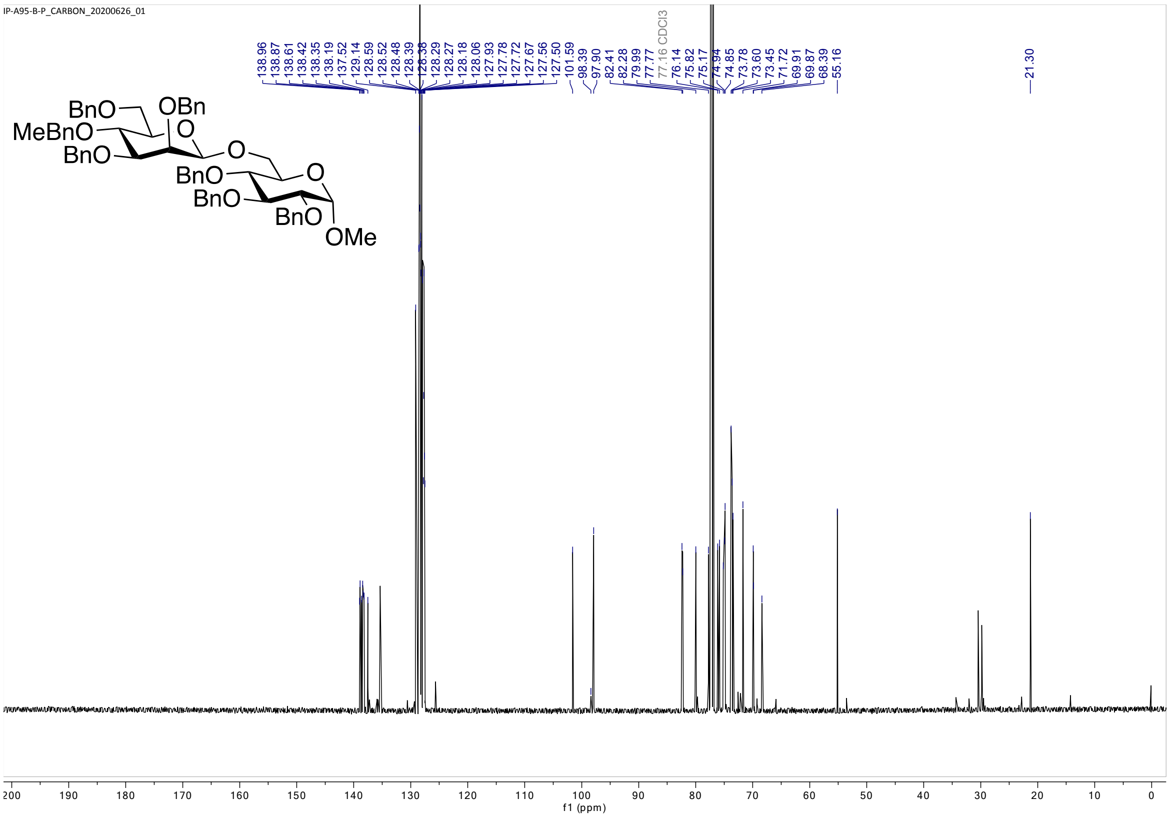

COSY NMR (500 MHz, Chloroform-*d*) 2c

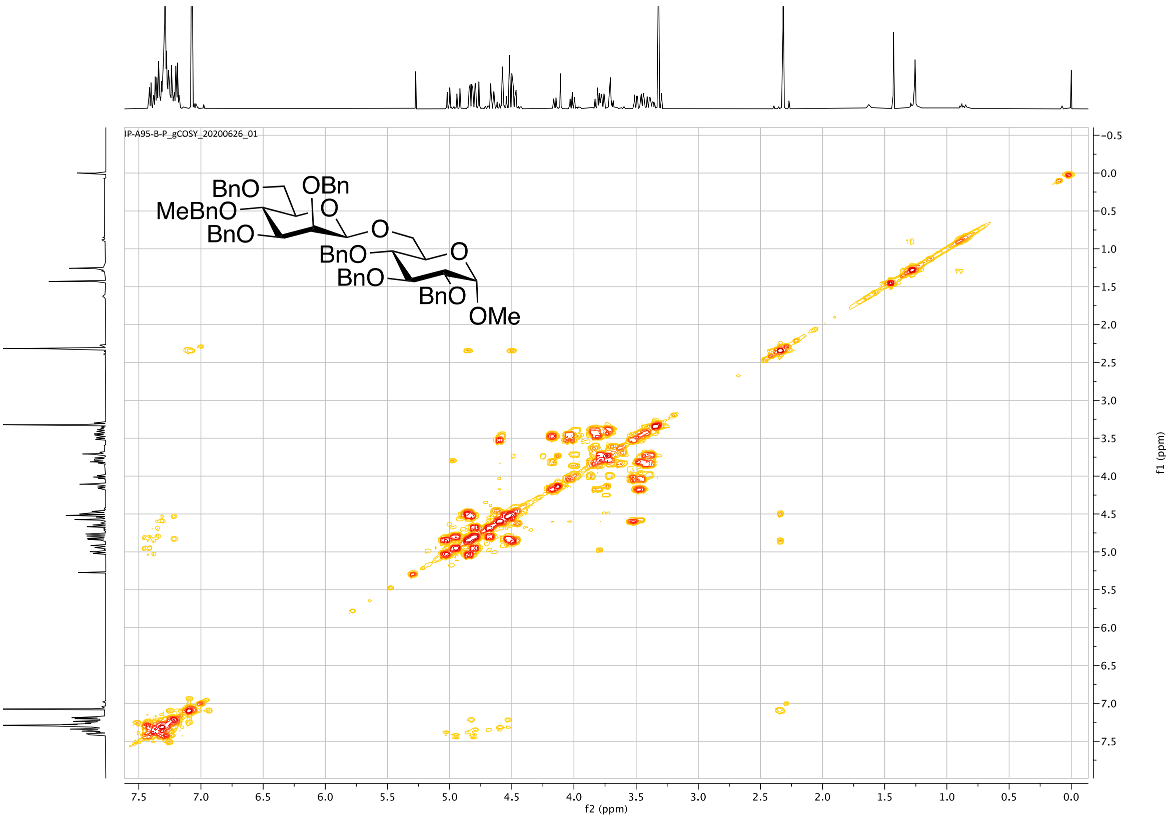

HSQC NMR (500 MHz x 126 MHz, Chloroform-*d*) 2c

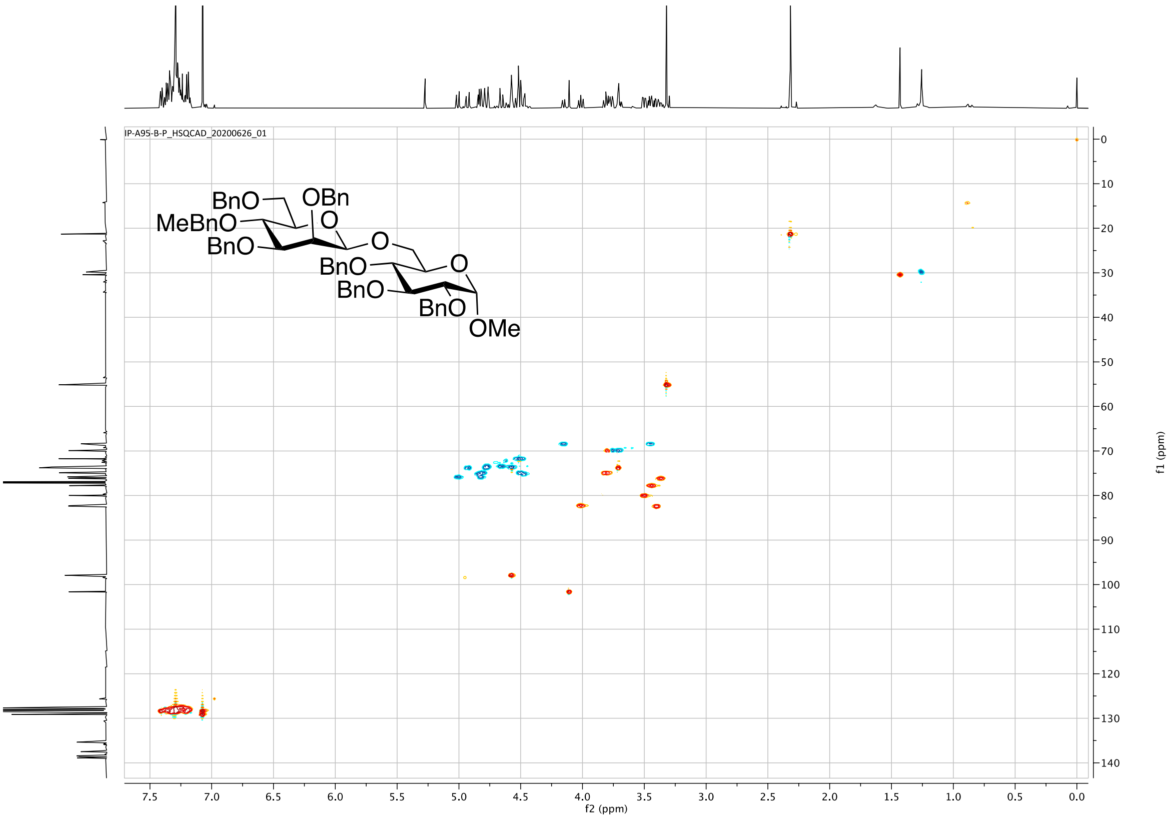

^{13}C- ^{1}H coupled HSQC NMR (500 MHz x 126 MHz, Chloroform-*d*) 2c

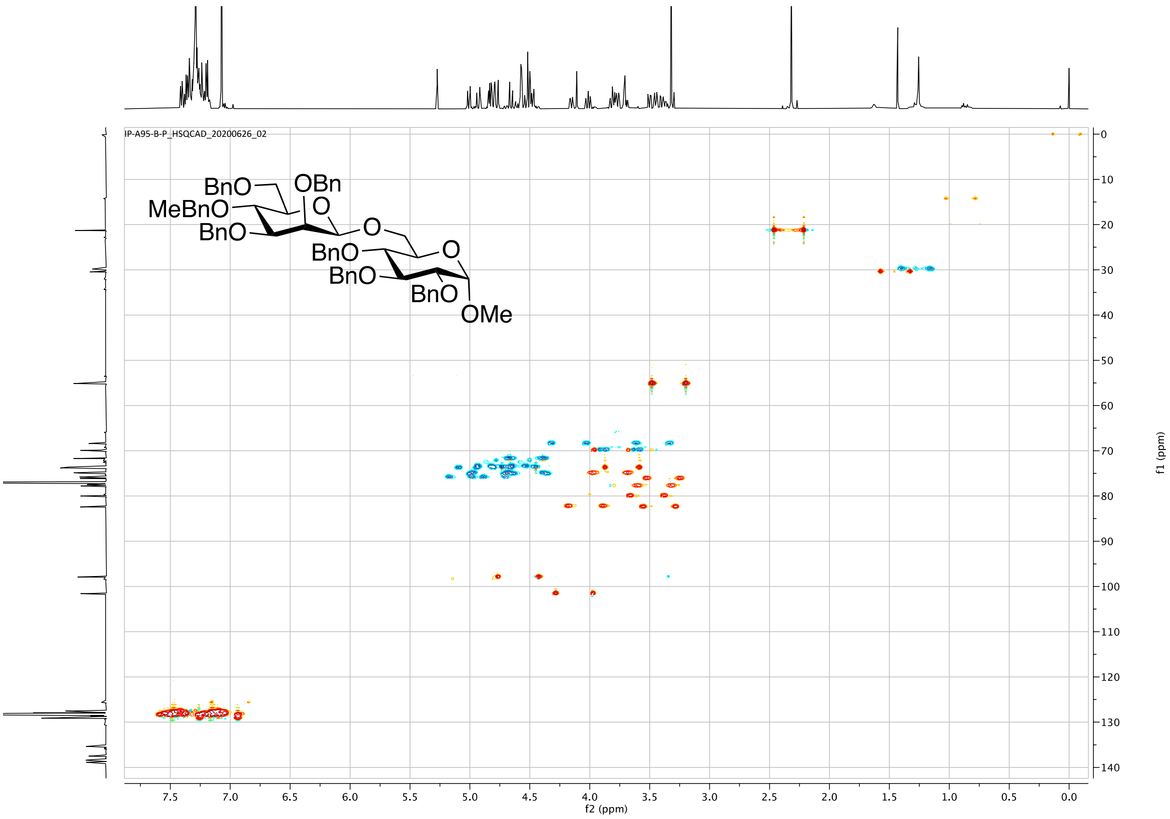

HMBC NMR (500 MHz x 126 MHz, Chloroform-*d*) 2c

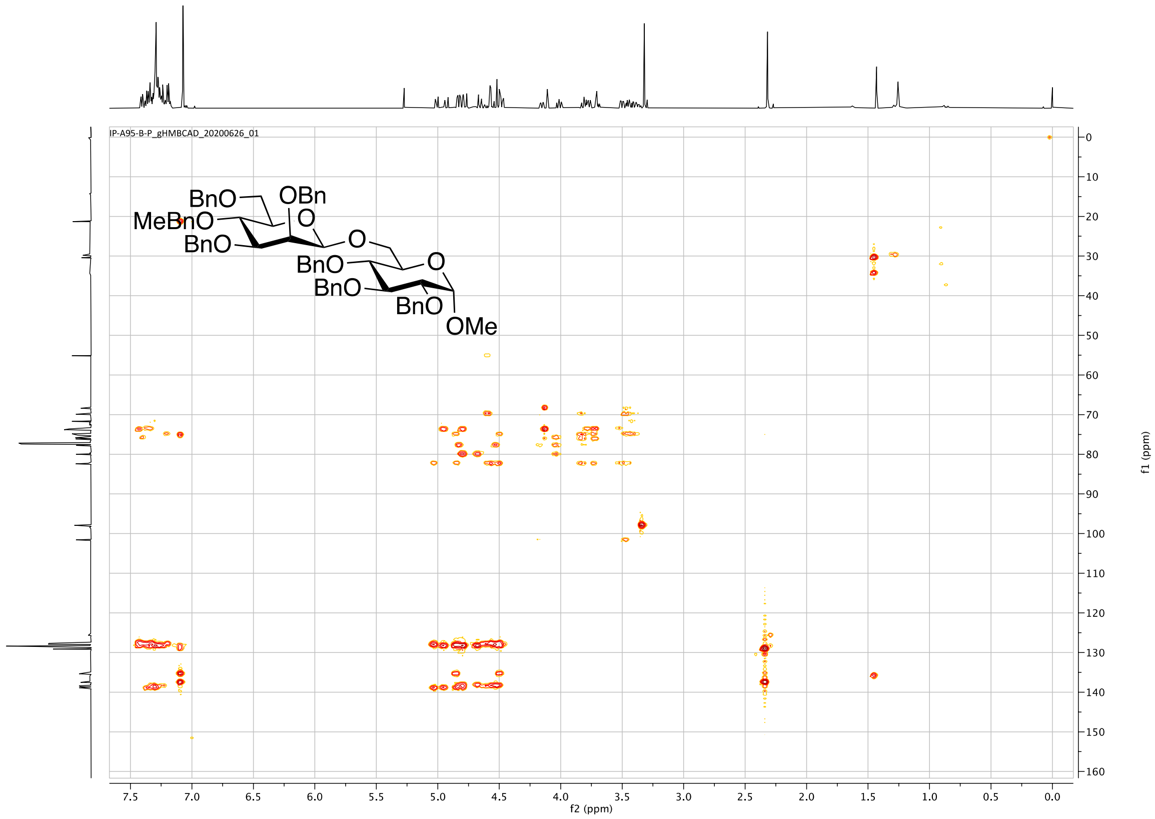

^{1}H NMR (600 MHz, Chloroform-*d*) 2d

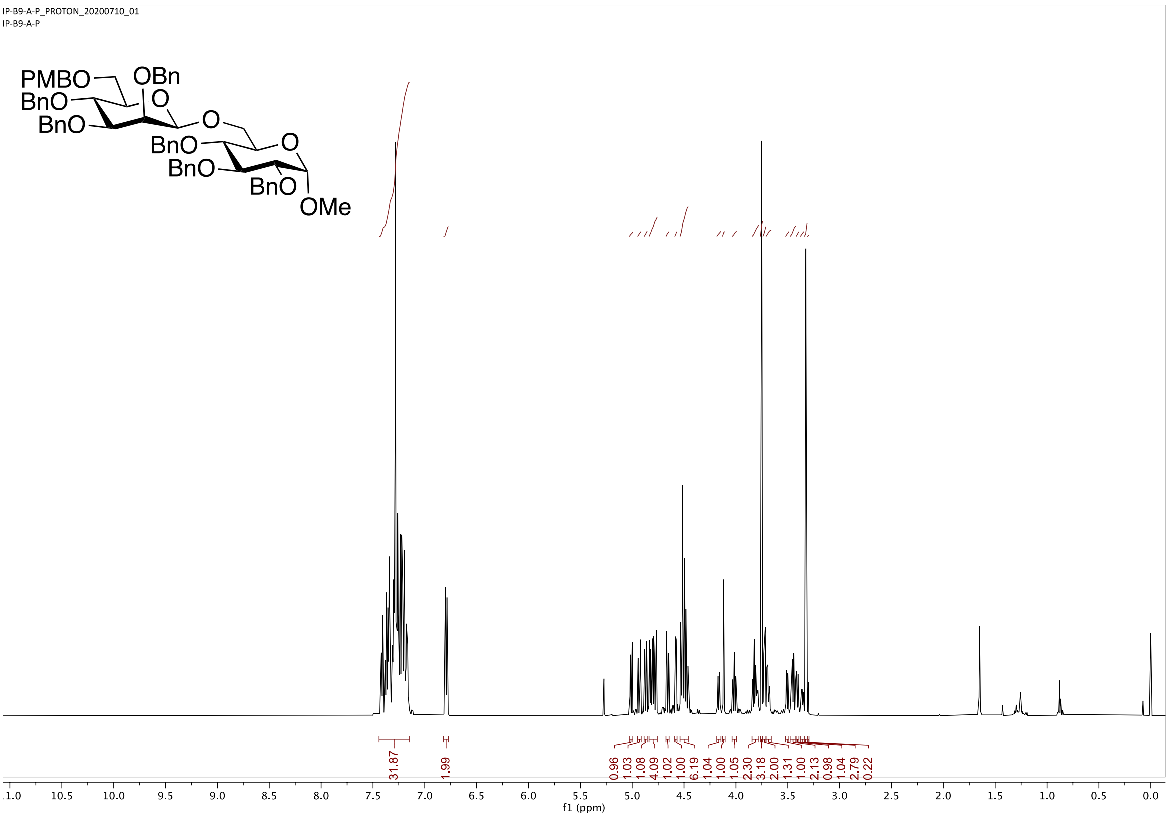

^{13}C NMR (151 MHz, Chloroform-*d*) 2d

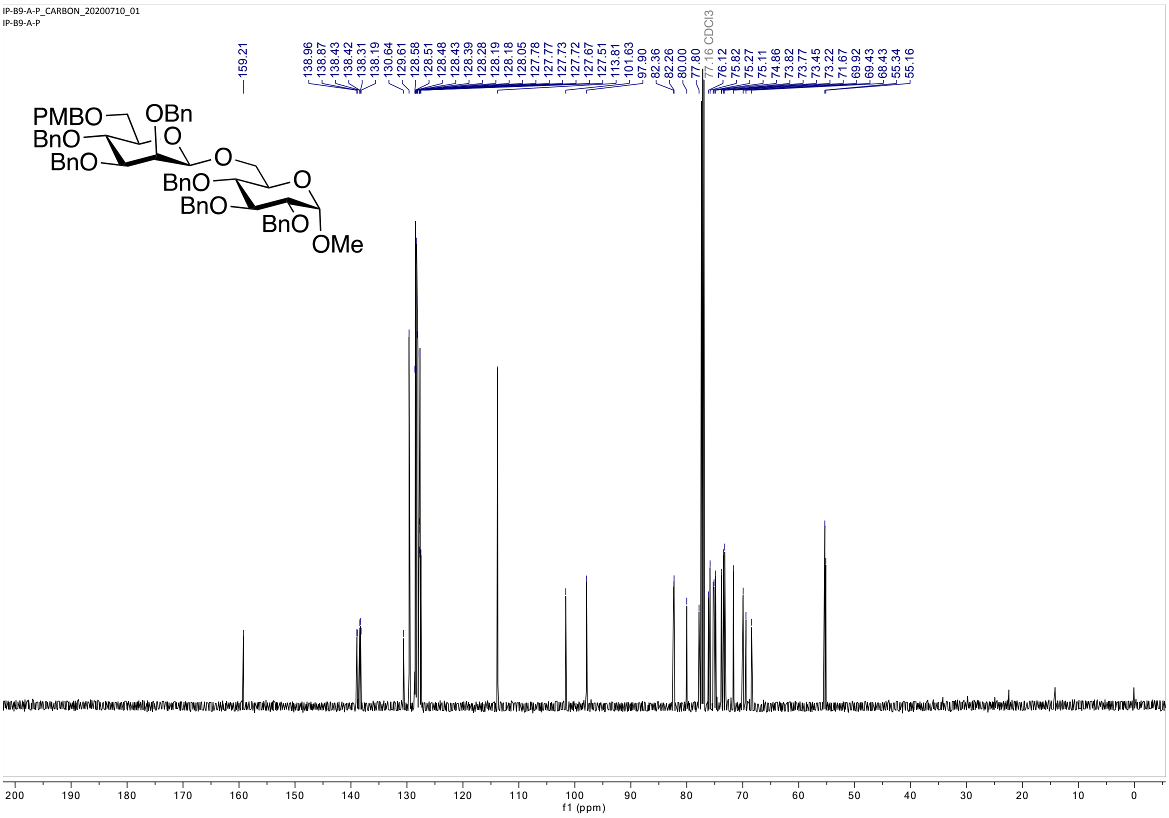

COSY NMR (600 MHz, Chloroform-*d*) 2d

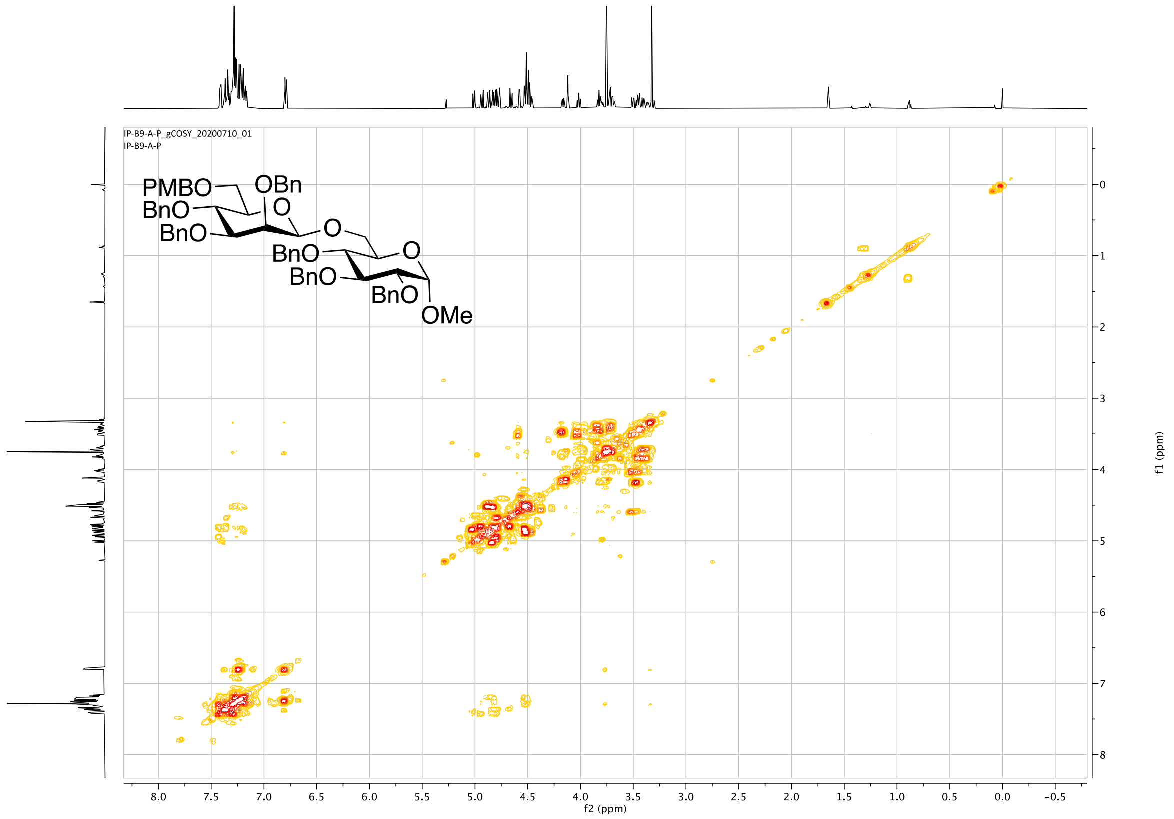

HSQC NMR (600 MHz x 151 MHz, Chloroform-*d*) 2d

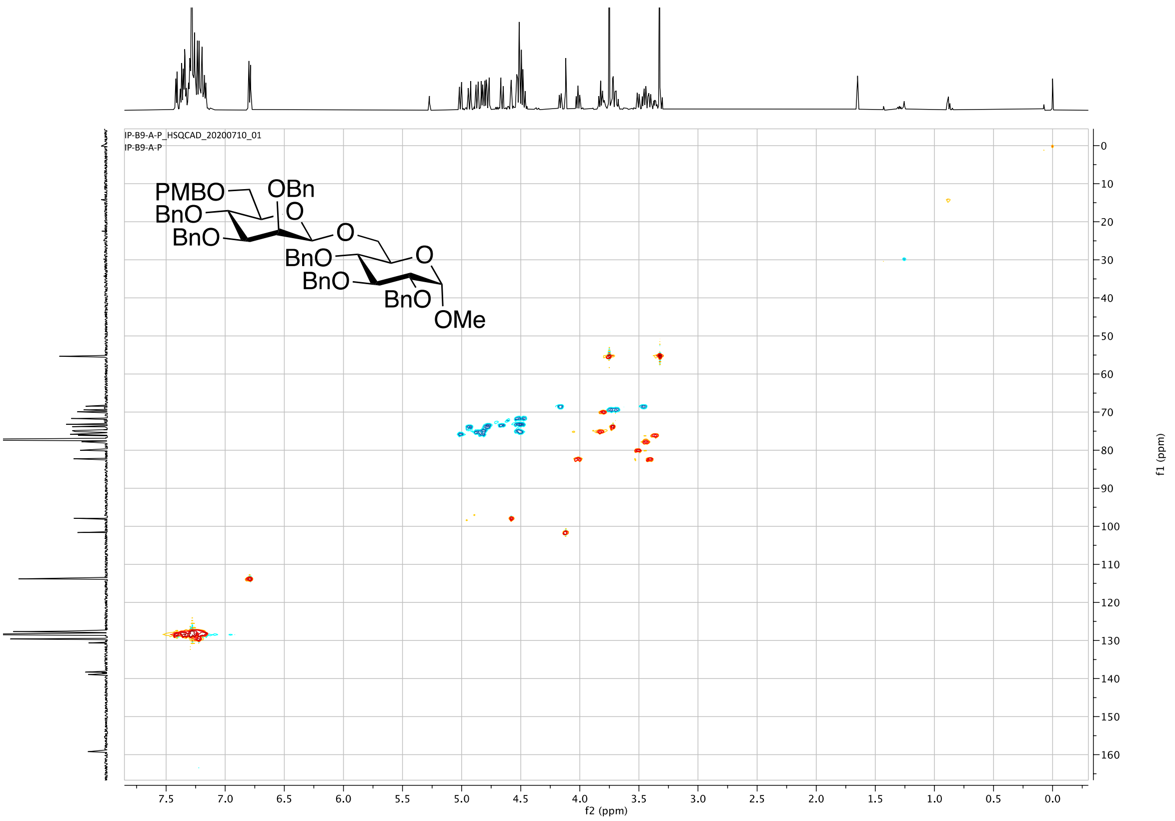

^{13}C- ^{1}H coupled HSQC NMR (600 MHz x 151 MHz, Chloroform-*d*) 2d

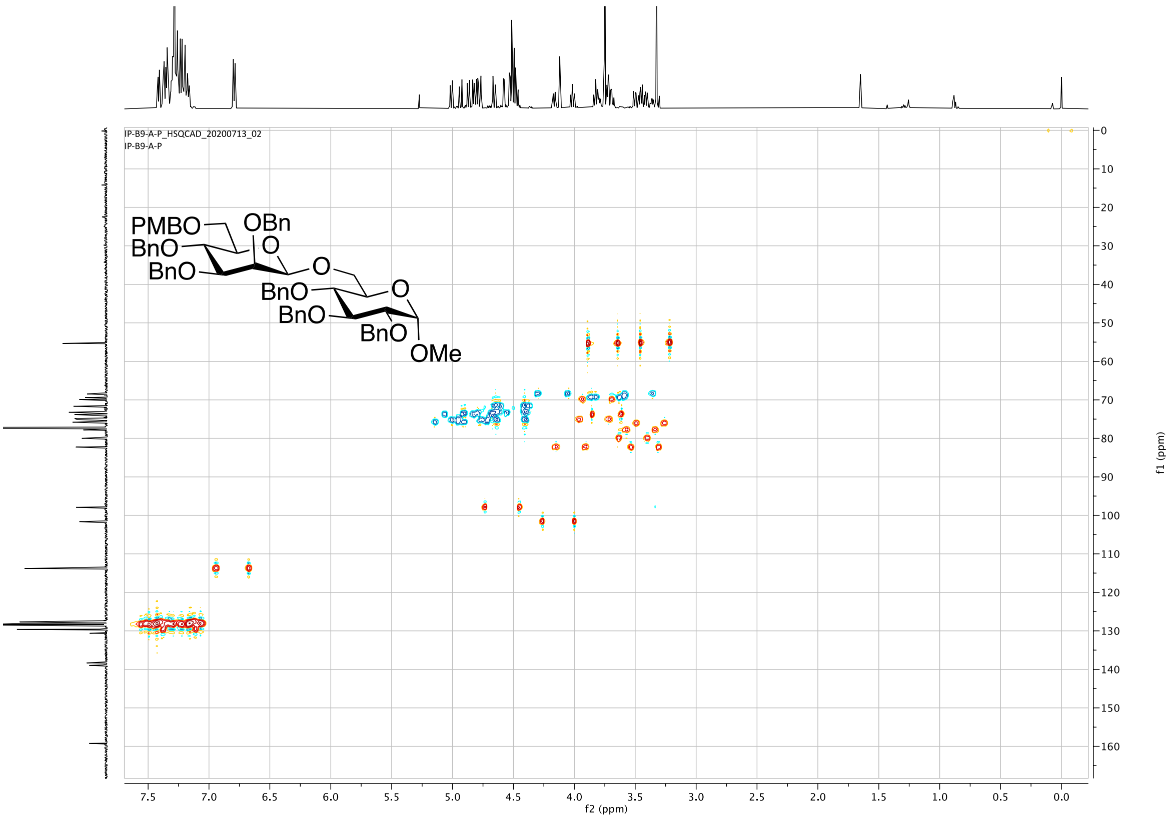

HMBC NMR (600 MHz x 151 MHz, Chloroform-*d*) 2d

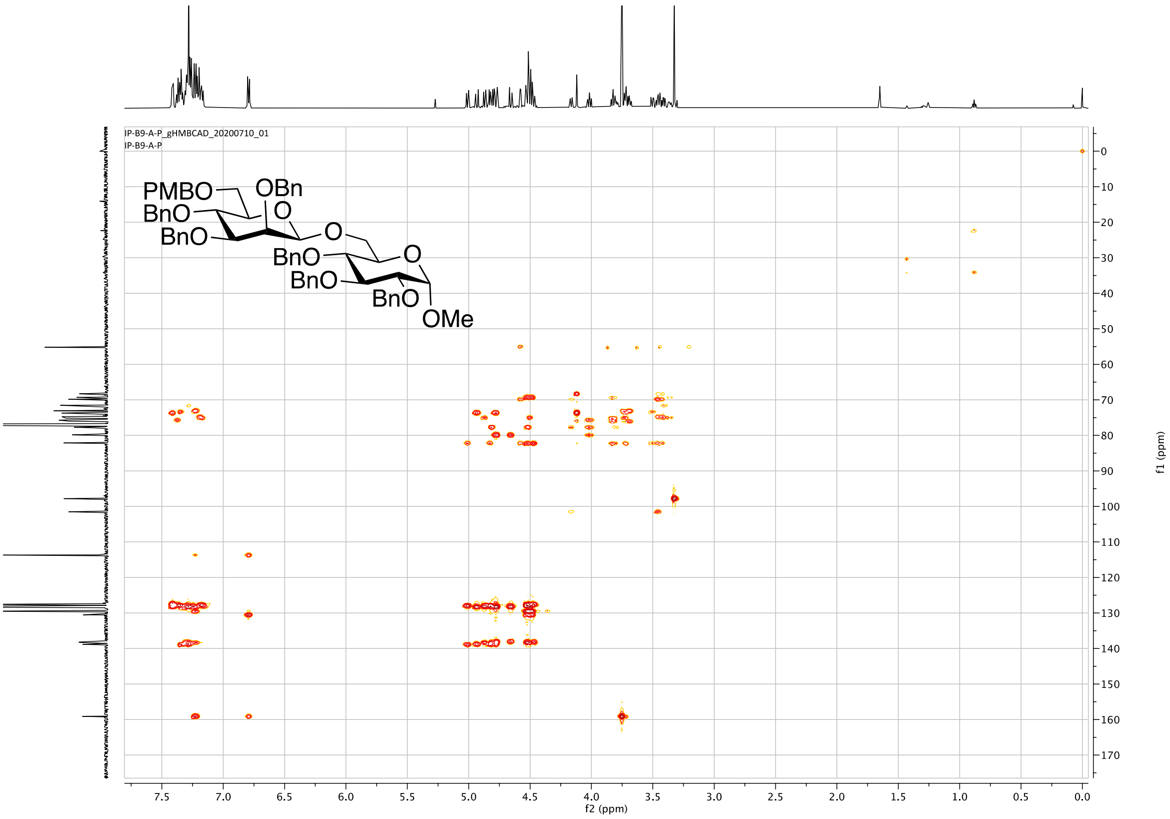

^{1}H NMR (500 MHz, Chloroform-*d*) 2e

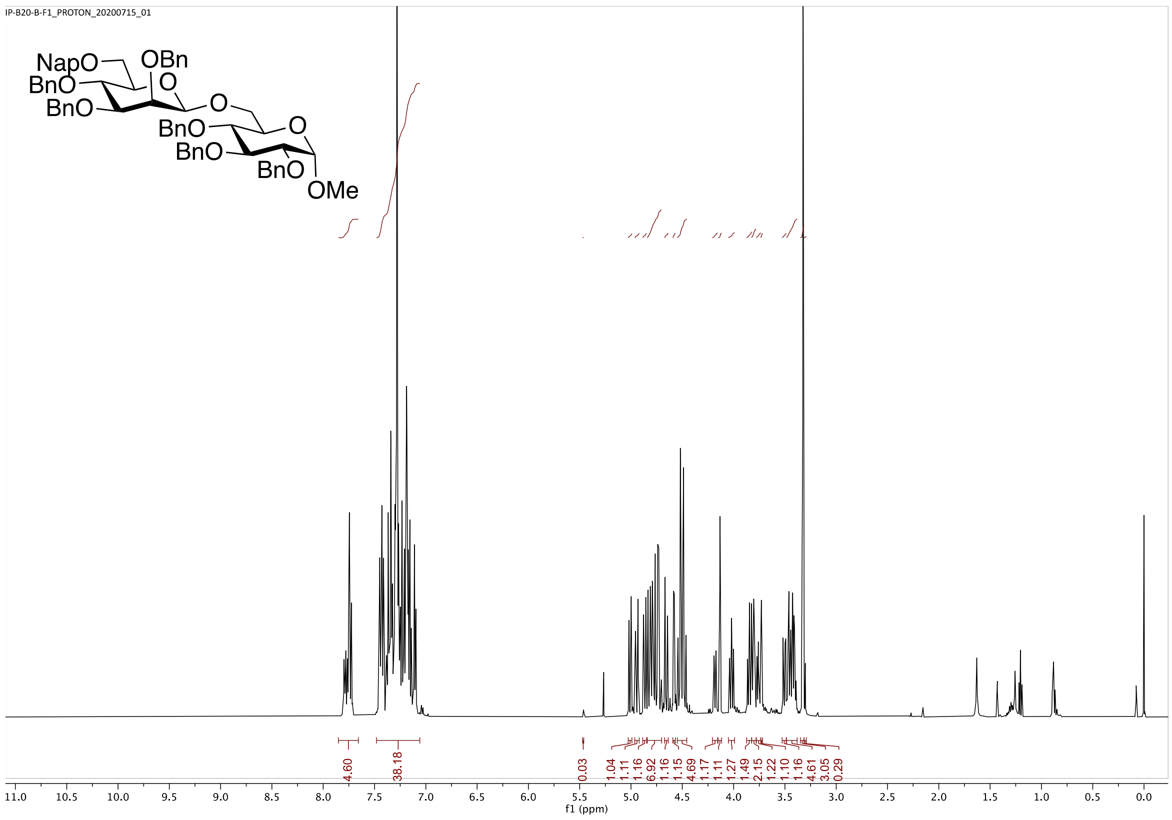

^{13}C NMR (126 MHz, Chloroform-*d*) 2e

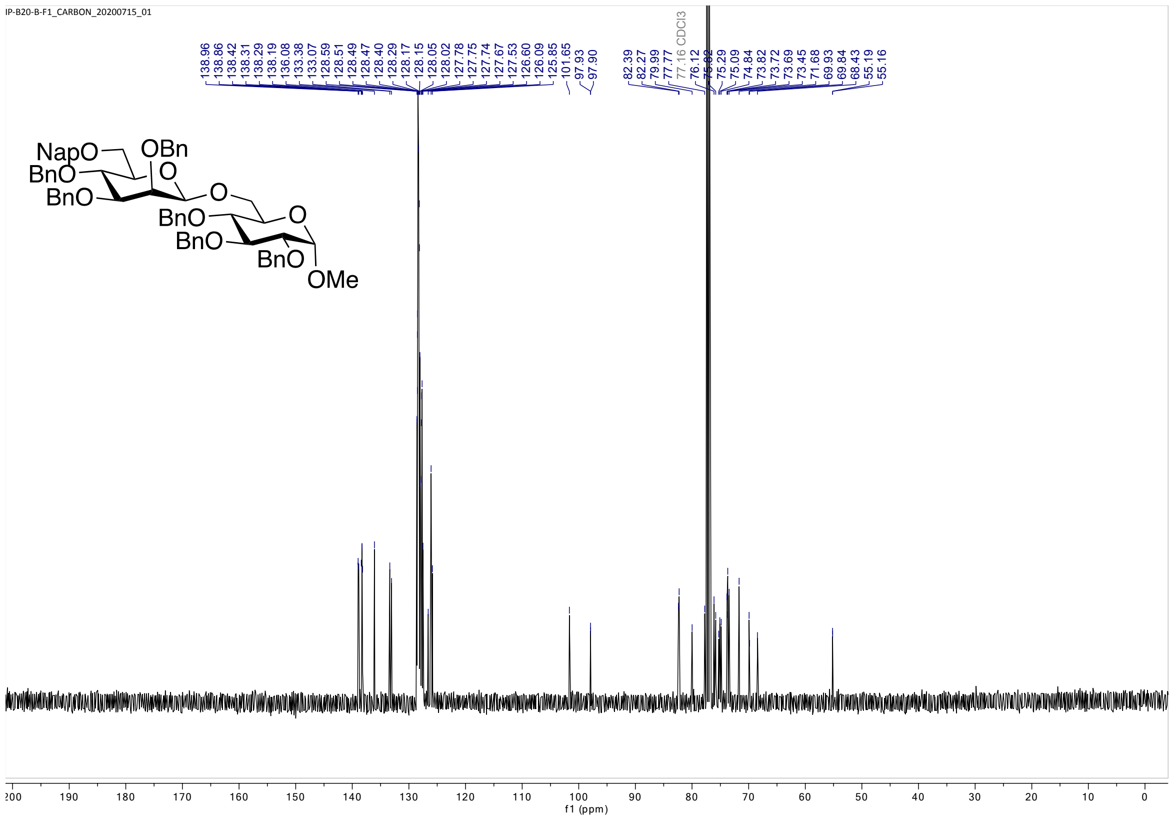

COSY NMR (500 MHz, Chloroform-*d*) 2e

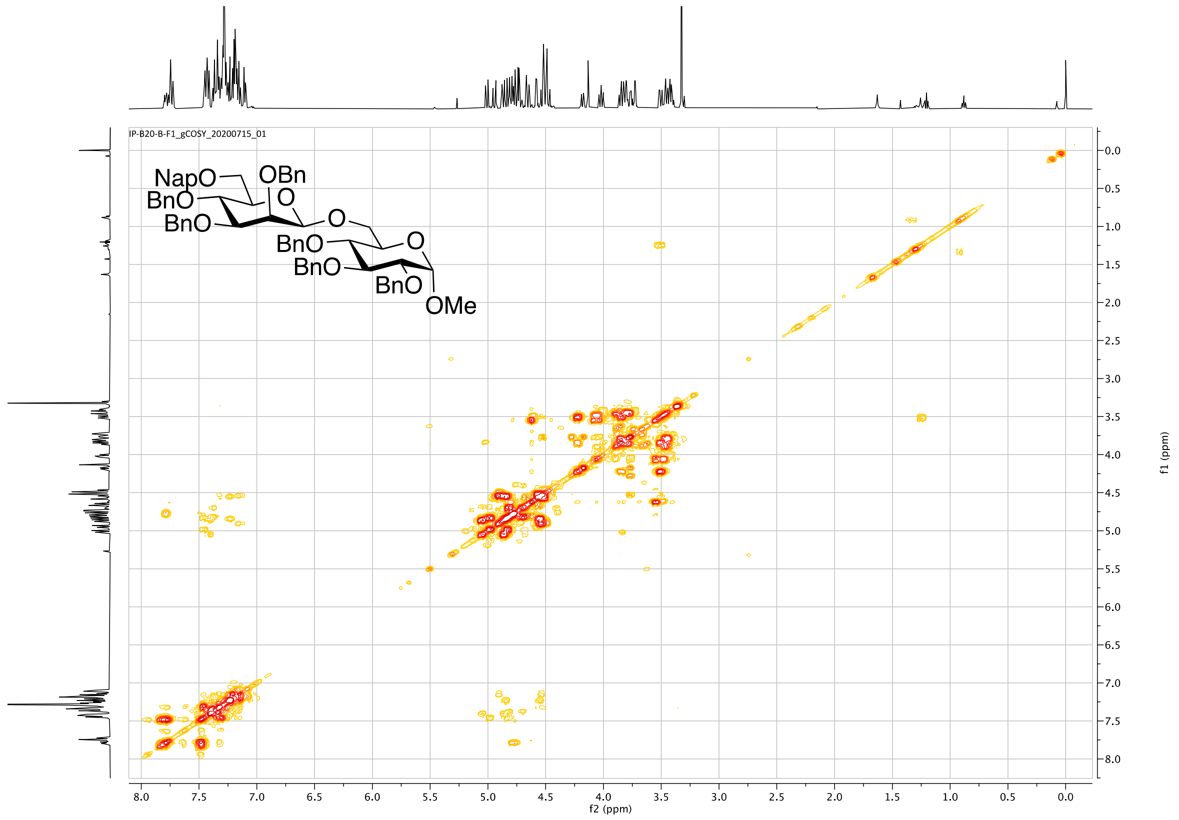

HSQC NMR (500 MHz x 126 MHz, Chloroform-*d*) 2e

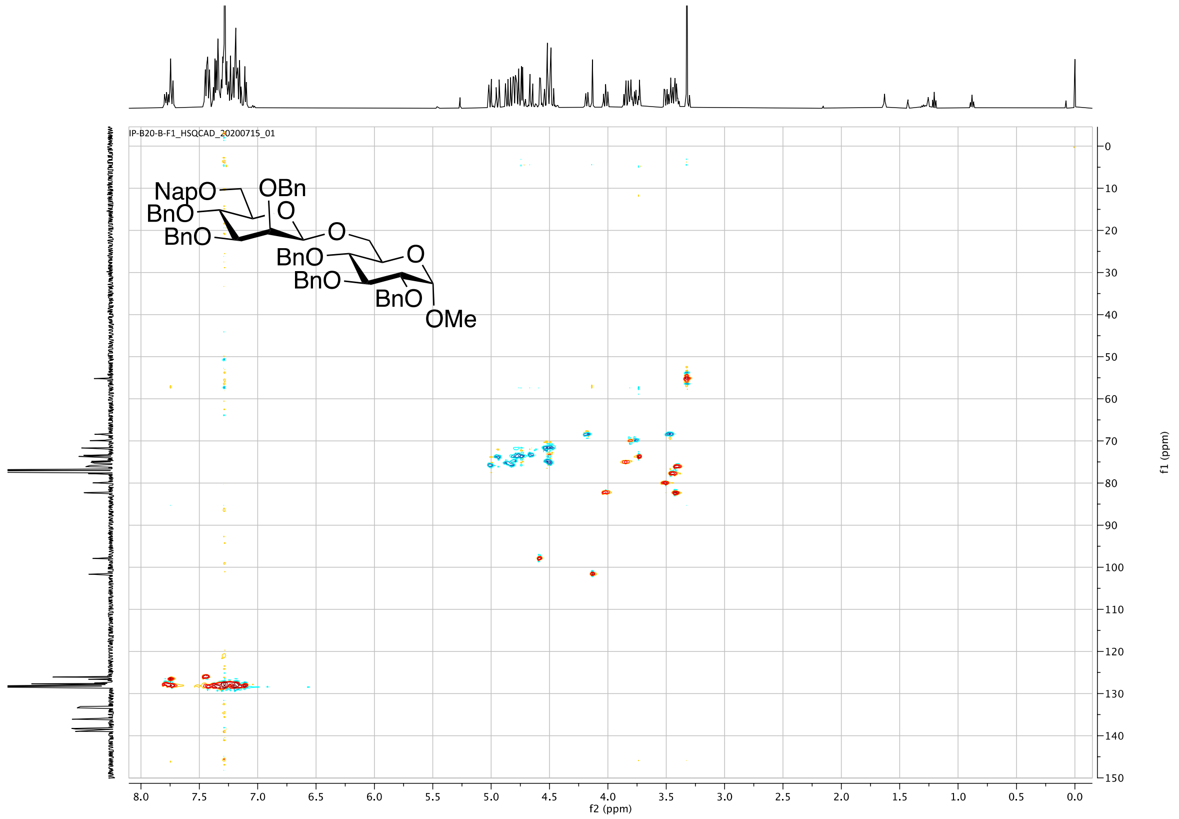

^{13}C- ^{1}H coupled HSQC NMR (500 MHz x 126 MHz, Chloroform-*d*) 2e

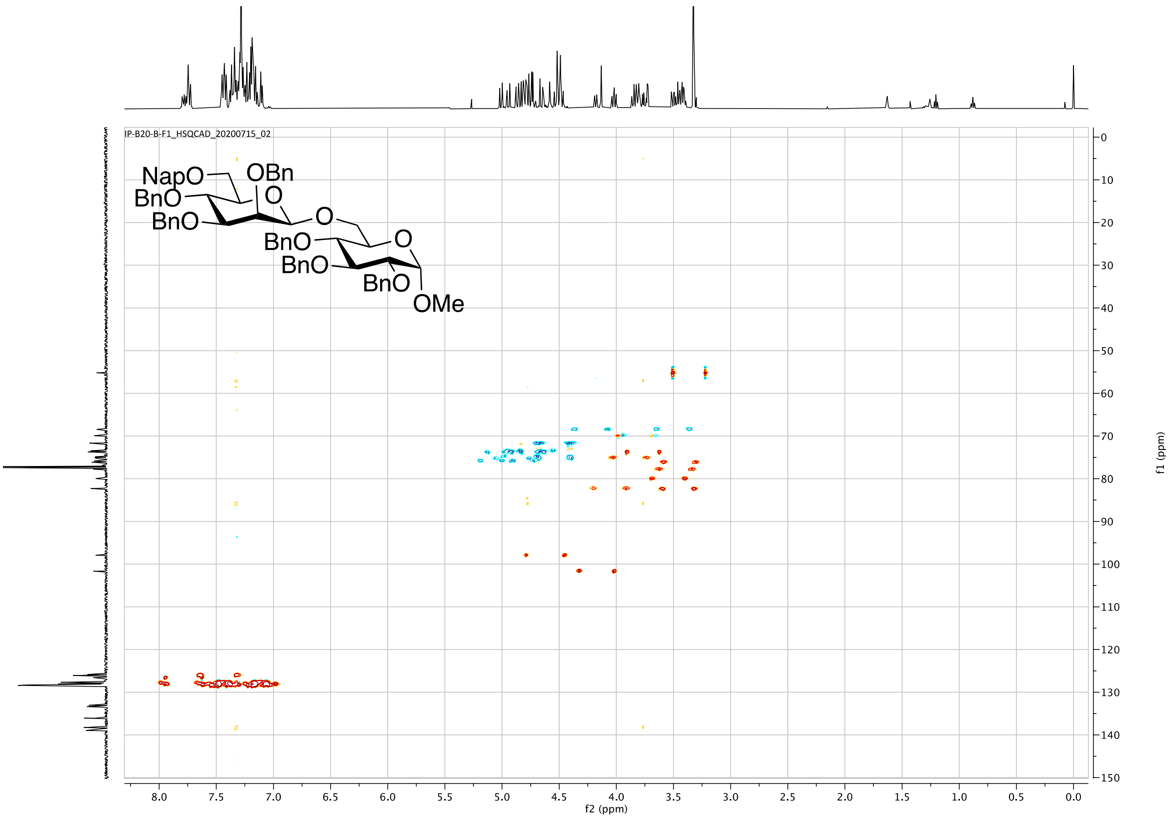

HMBC NMR (500 MHz x 126 MHz, Chloroform-*d*) 2e

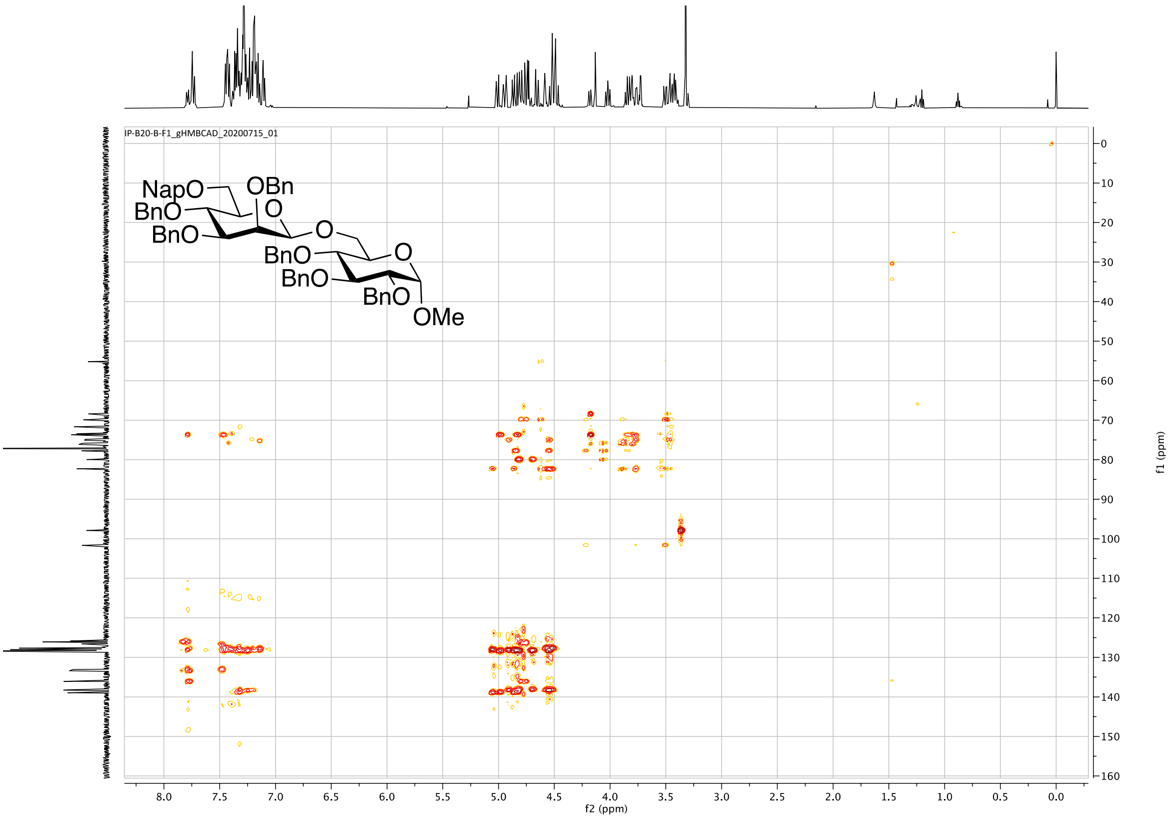

^{1}H NMR (500 MHz, Chloroform-*d*) 2f

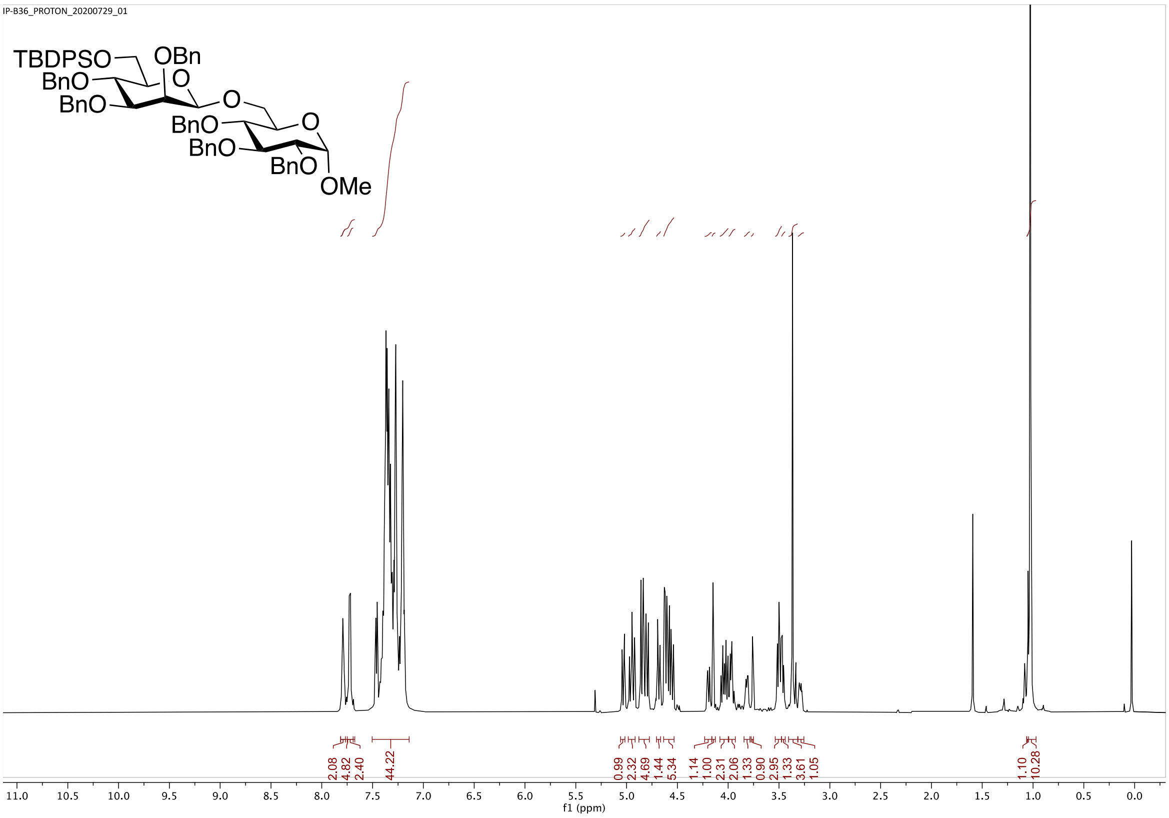

^{13}C NMR (126 MHz, Chloroform-*d*) 2f

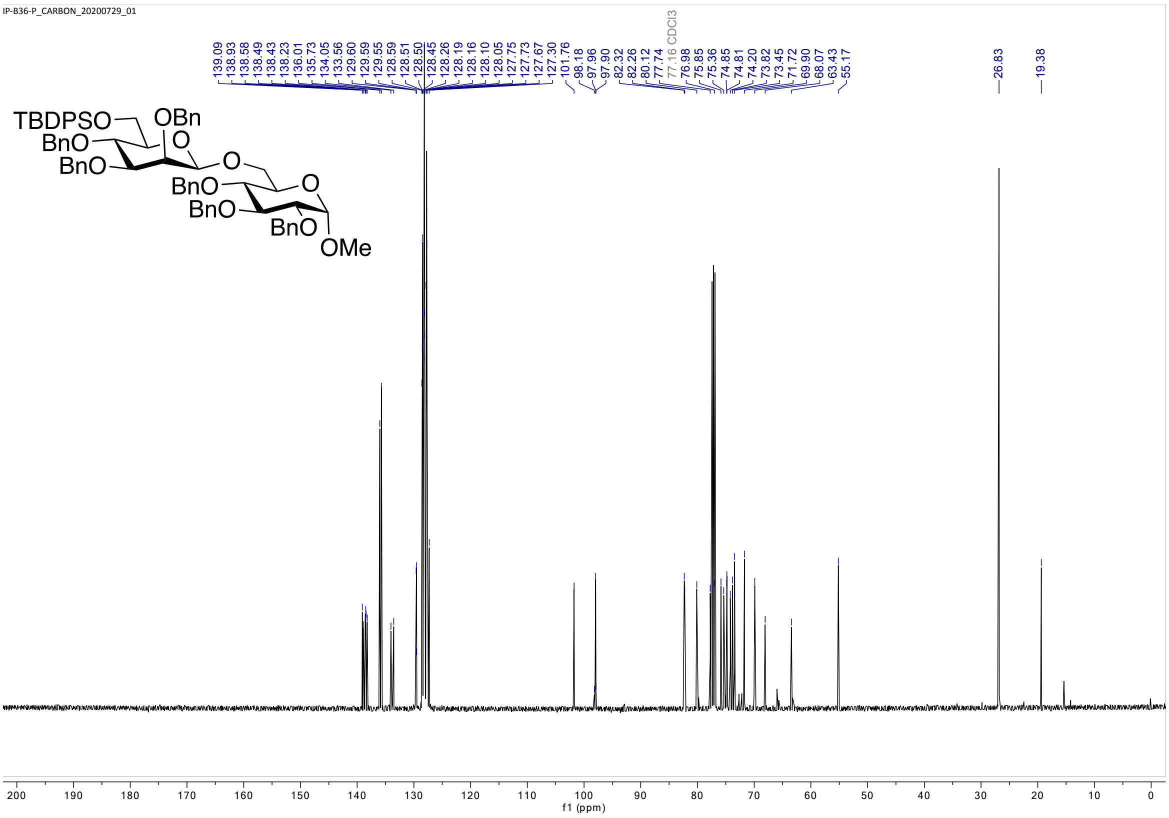

COSY NMR (500 MHz, Chloroform-*d*) 2f

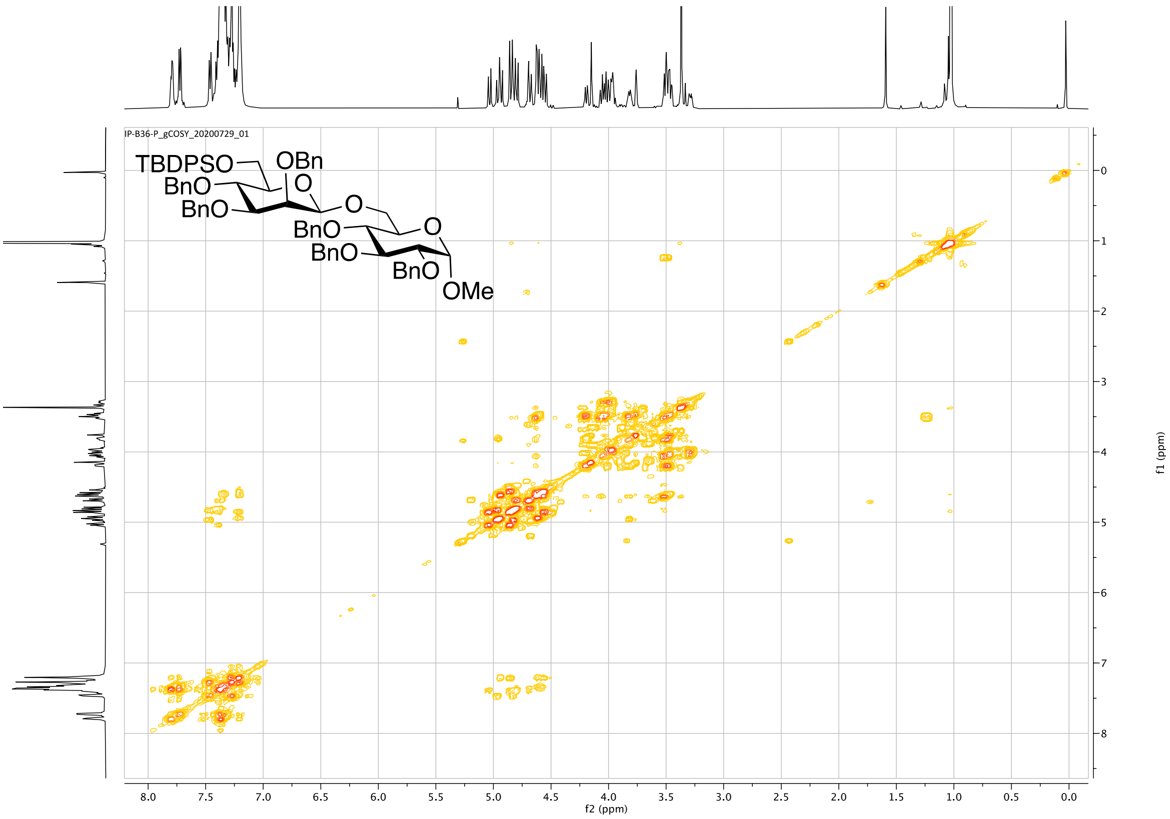

HSQC NMR (500 MHz x 126 MHz, Chloroform-*d*) 2f

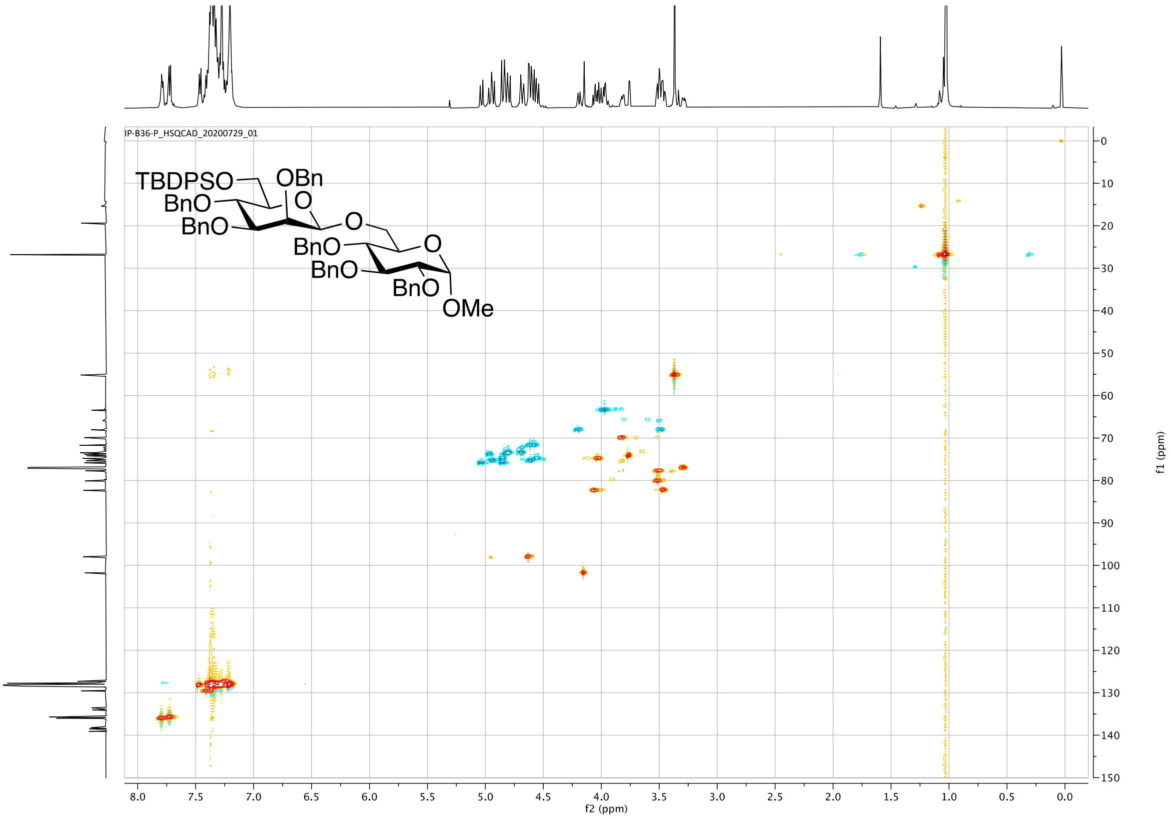

^{13}C- ^{1}H coupled HSQC NMR (500 MHz x 126 MHz, Chloroform-*d*) 2f

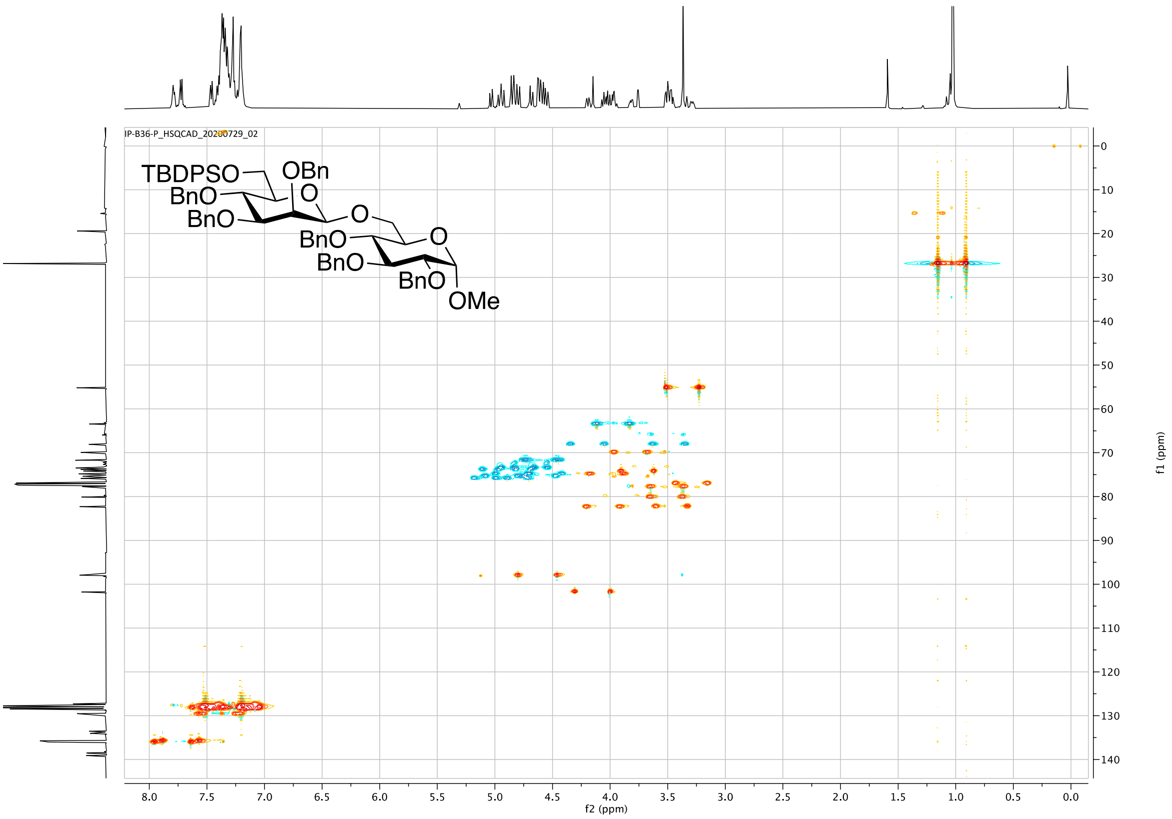

HMBC NMR (500 MHz x 126 MHz, Chloroform-*d*) 2f

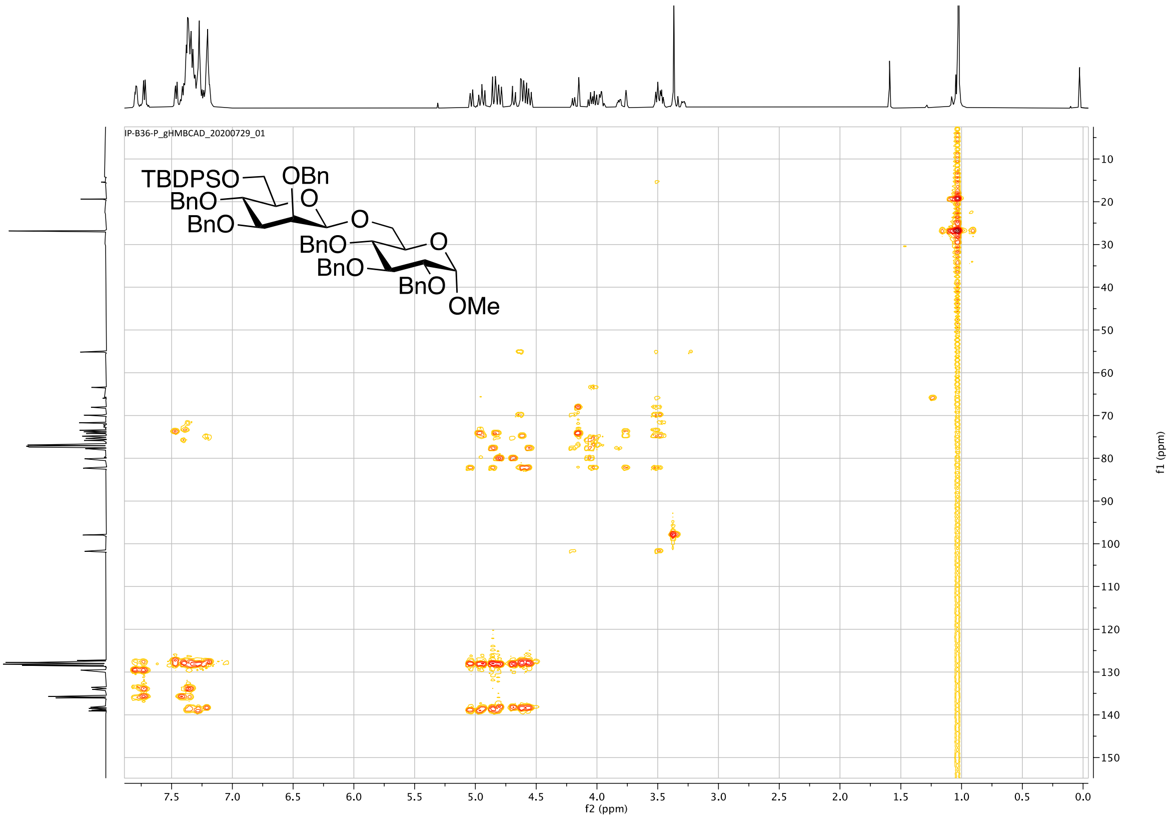

^{1}H NMR (500 MHz, Chloroform-*d*) 2g

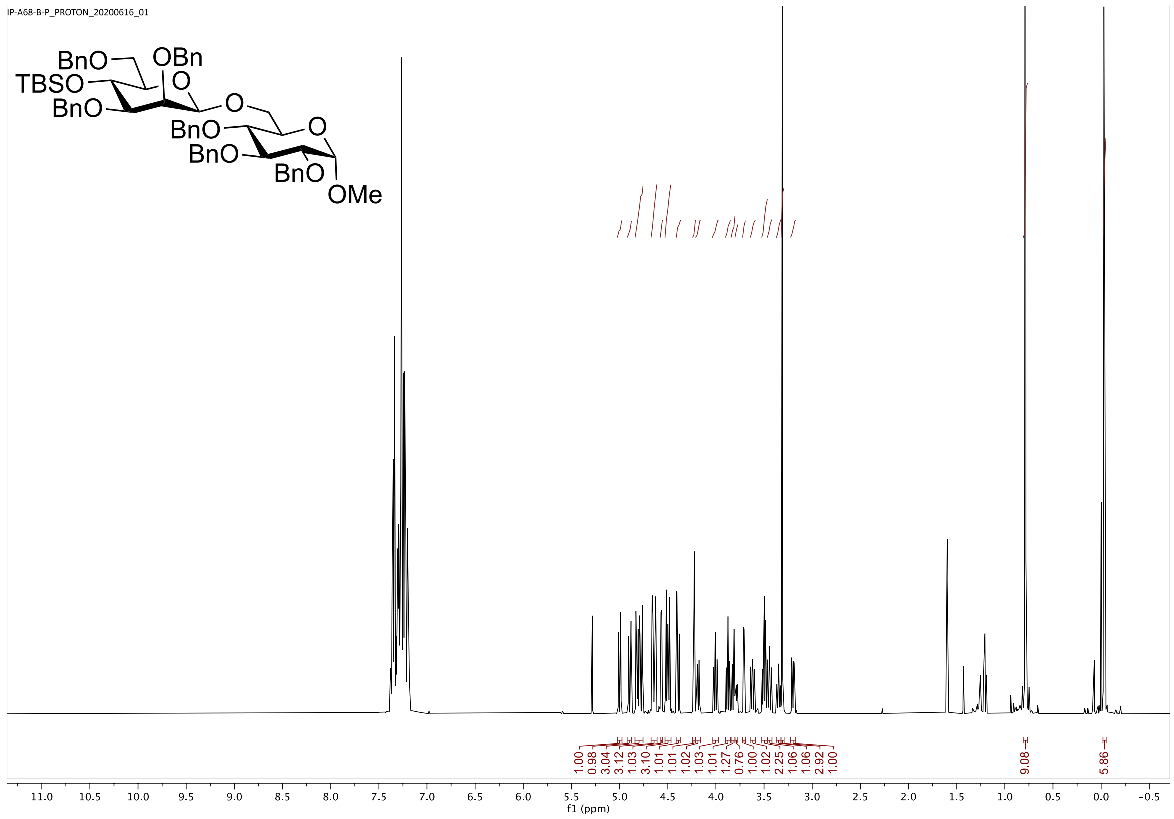

^{13}C NMR (126 MHz, Chloroform-*d*) 2g

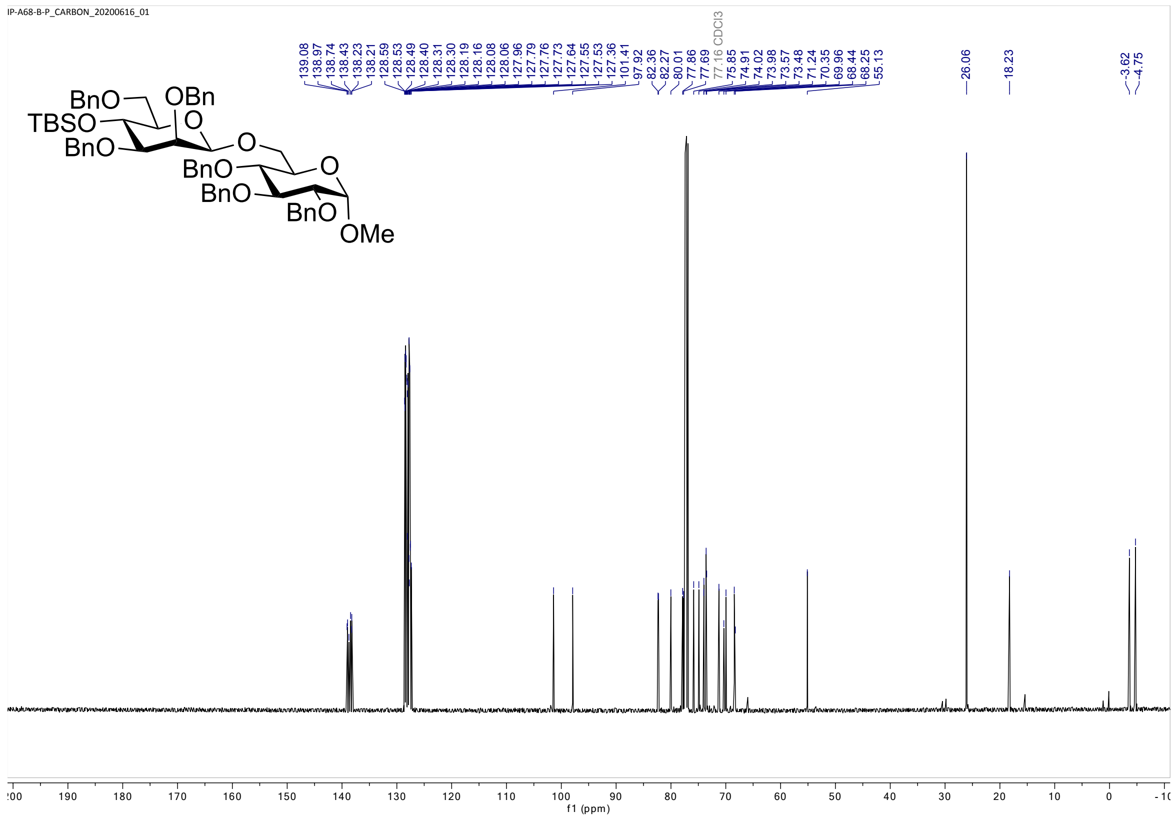

COSY NMR (500 MHz, Chloroform-*d*) 2g

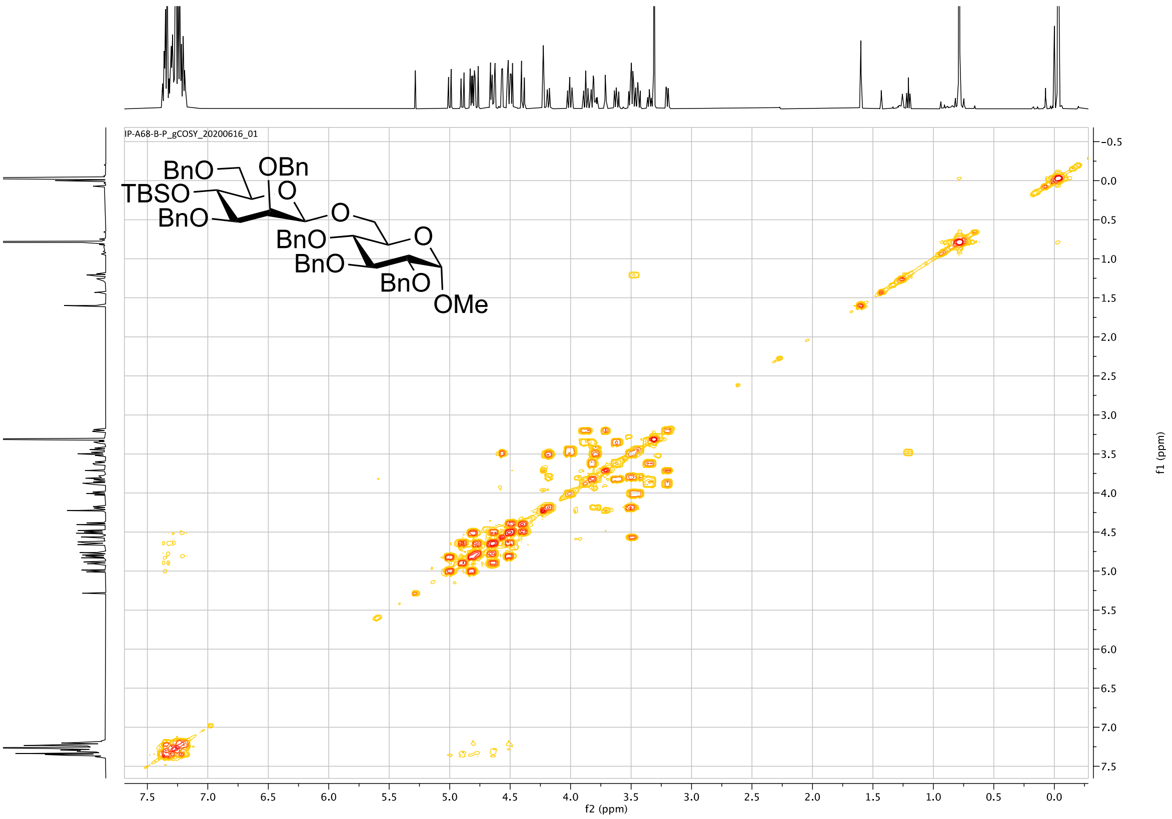

HSQC NMR (500 MHz x 126 MHz, Chloroform-*d*) 2g

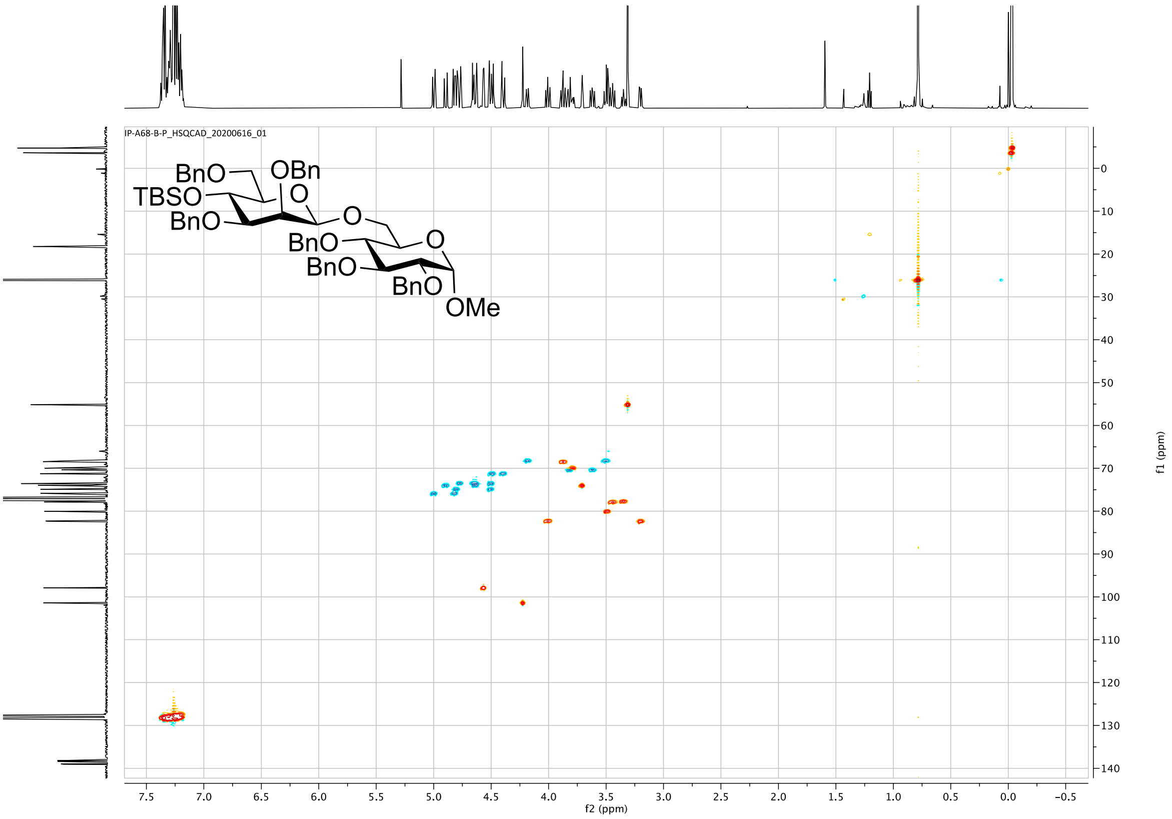

^{13}C- ^{1}H coupled HSQC NMR (500 MHz x 126 MHz, Chloroform-*d*) 2g

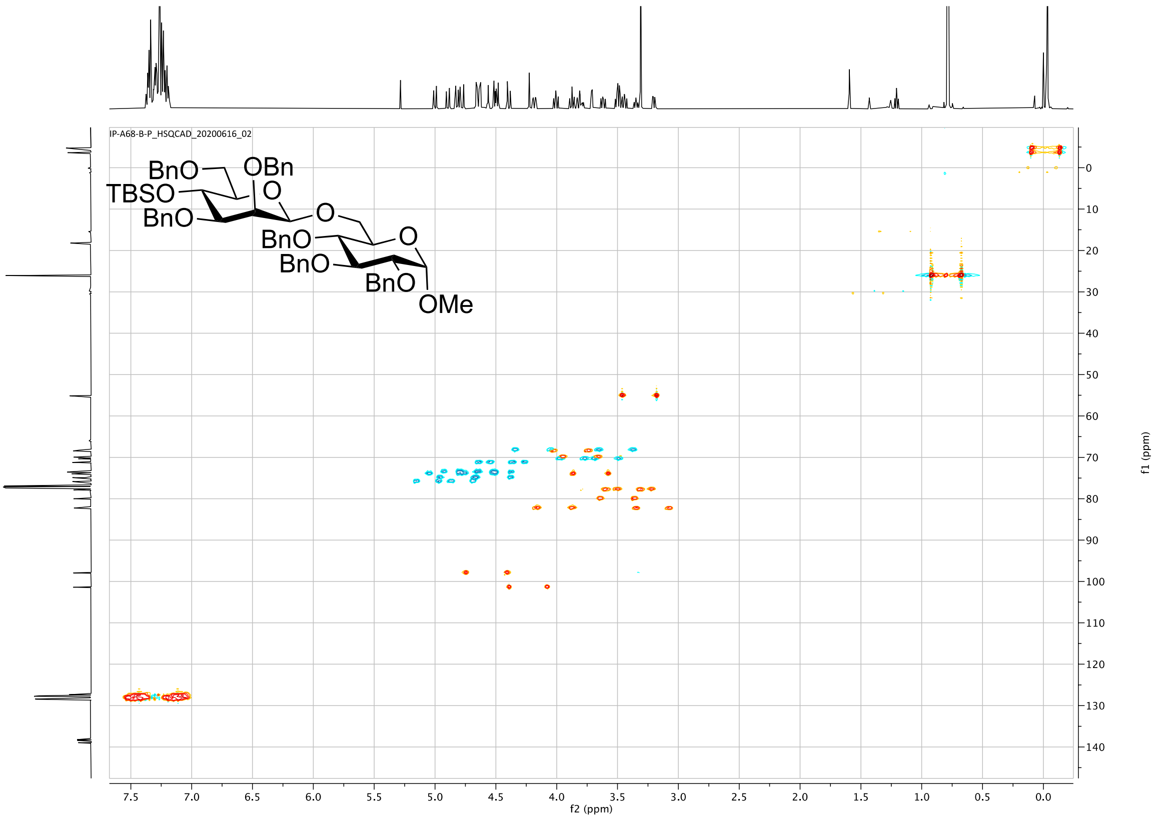

HMBC NMR (500 MHz x 126 MHz, Chloroform-*d*) 2g

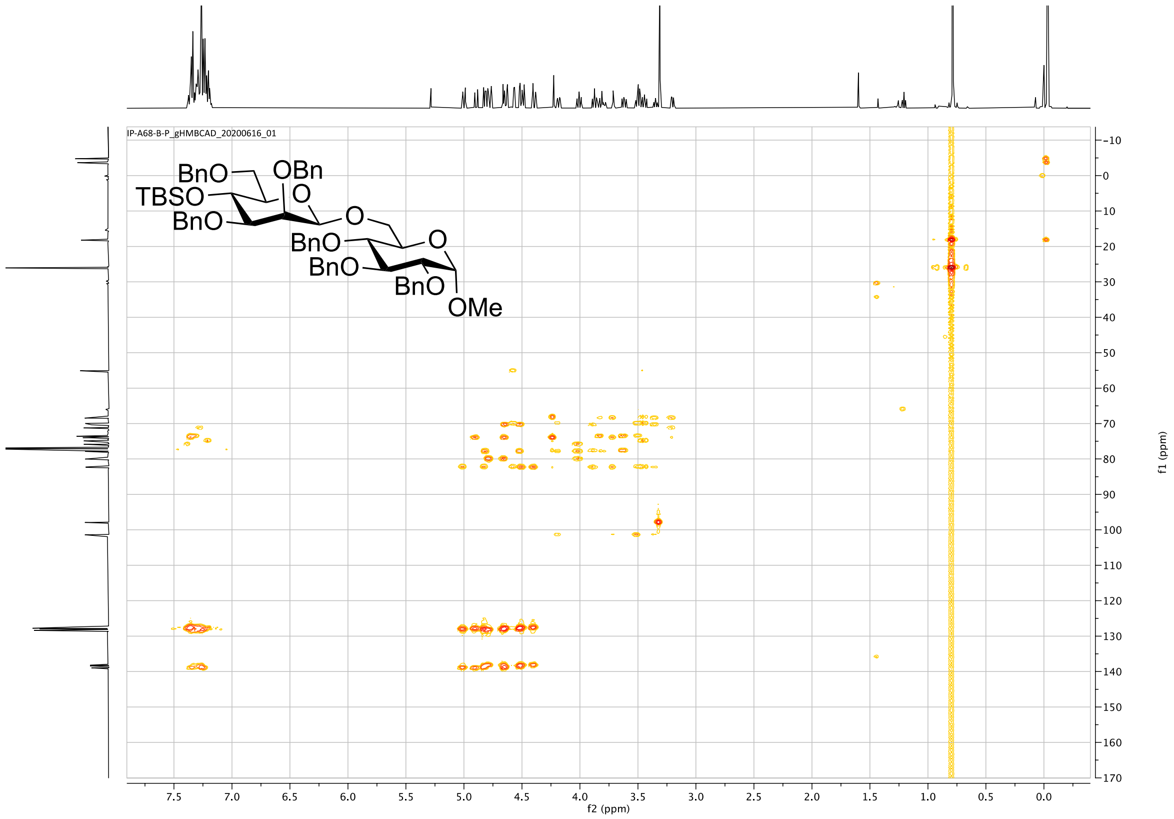

^{1}H NMR (500 MHz, Chloroform-*d*) 2h

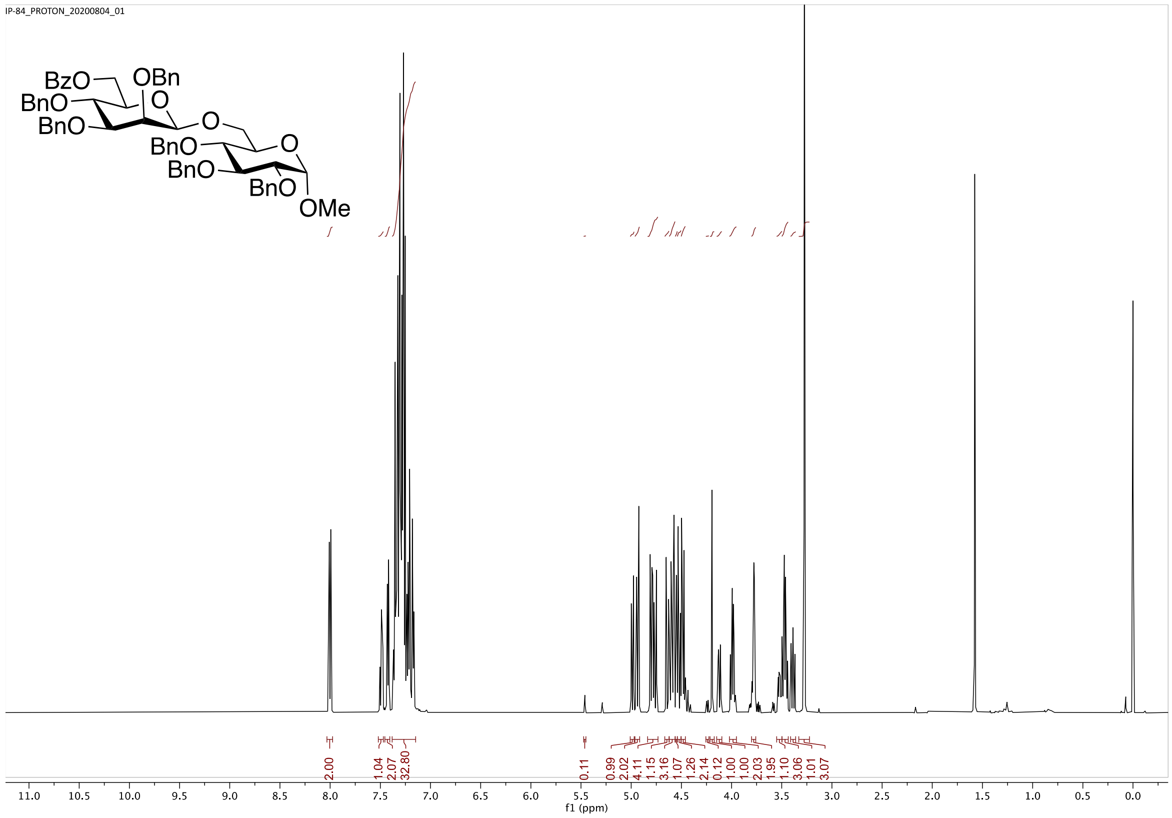

^{13}C NMR (126 MHz, Chloroform-*d*) 2h

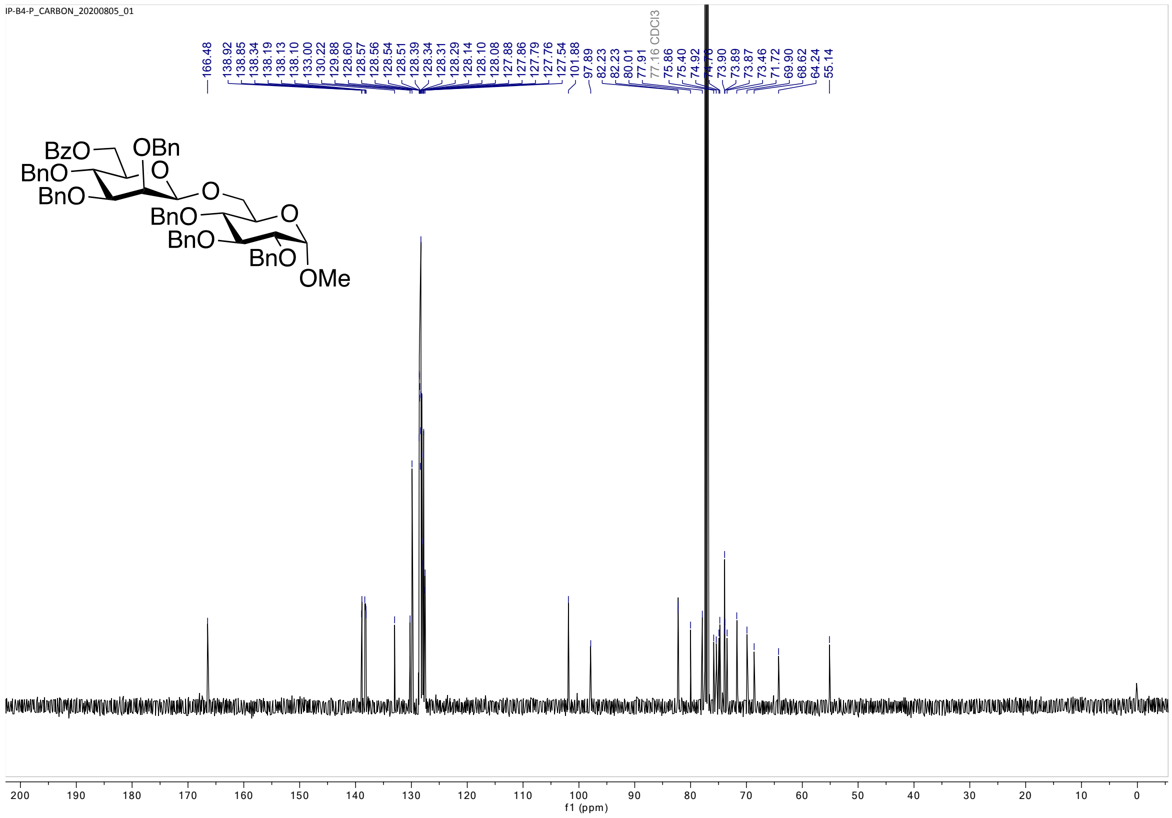

^{1}H NMR (500 MHz, Chloroform-*d*) 2i

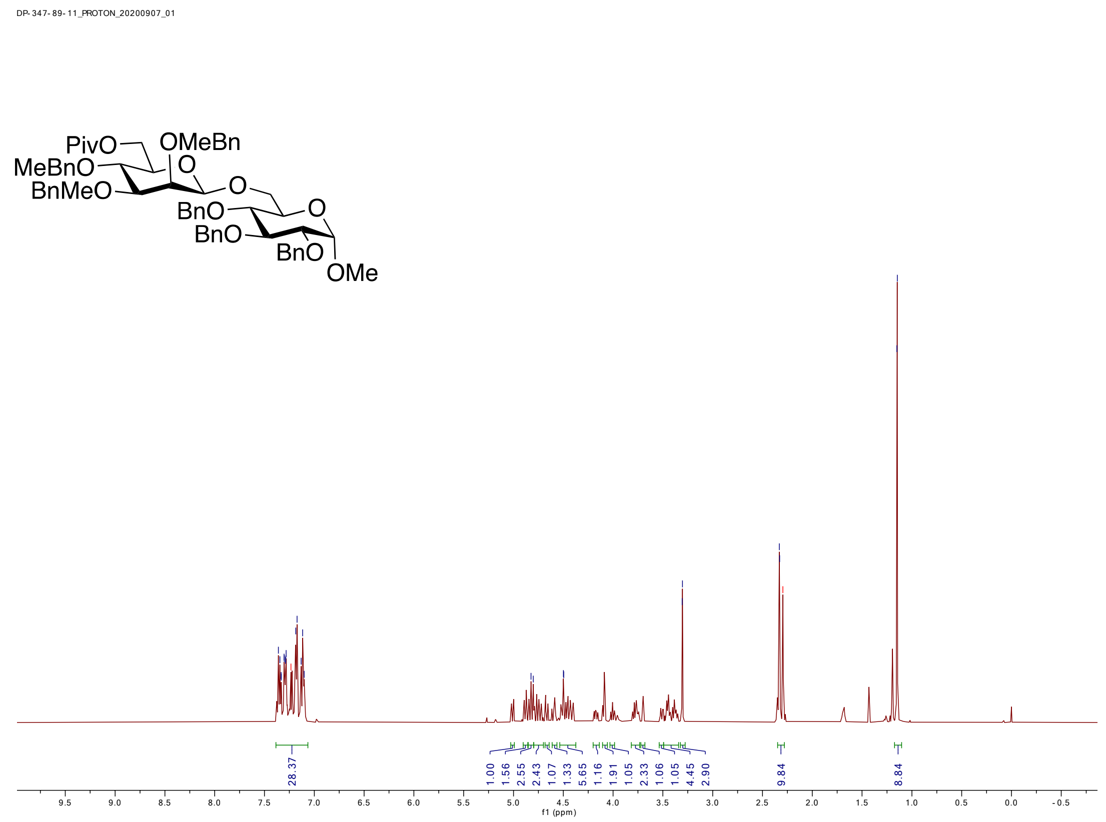

^{13}C NMR (126 MHz, Chloroform-*d*) 2i

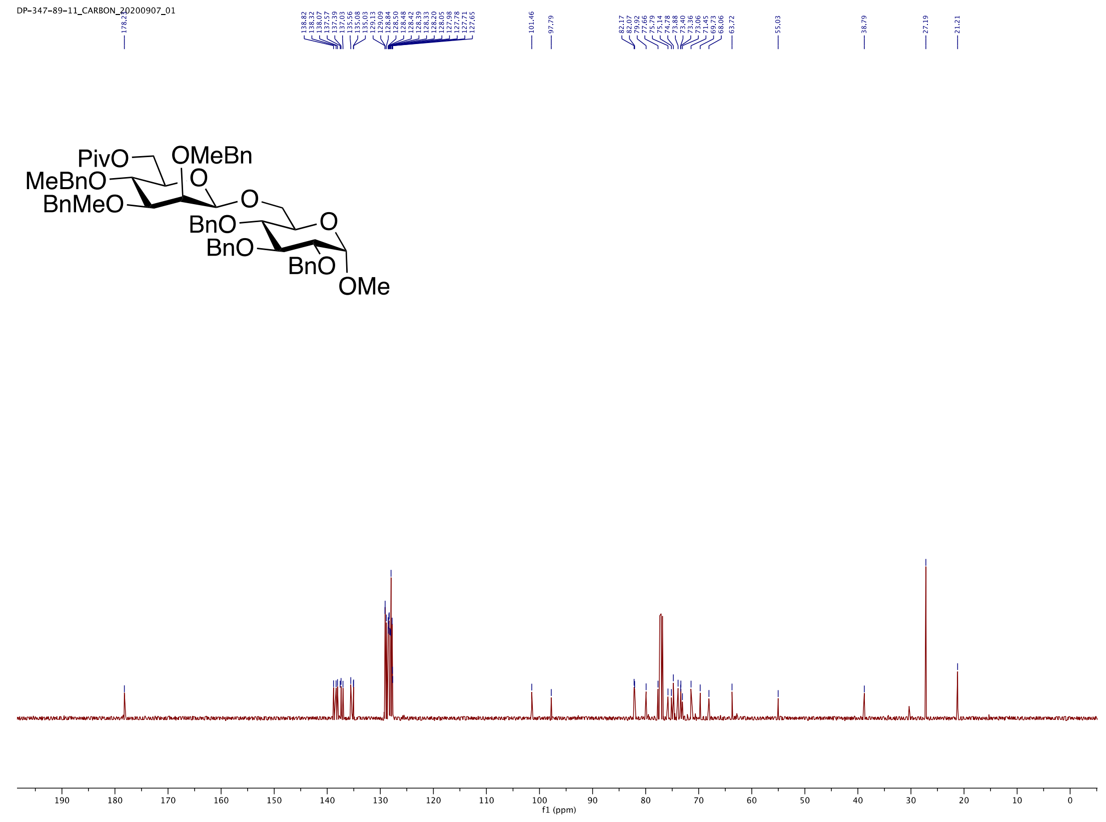

COSY NMR (500 MHz, Chloroform-*d*) 2i

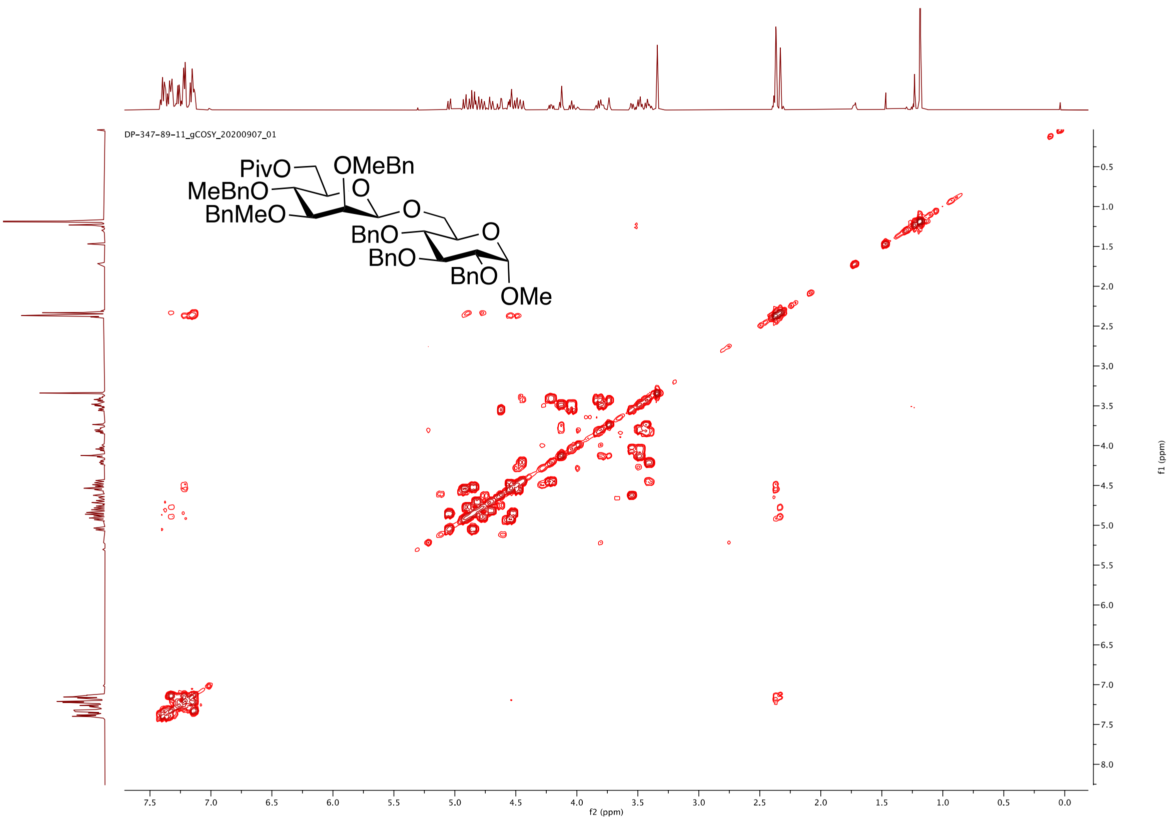

HSQC NMR (500 MHz x 126 MHz, Chloroform-*d*) 2i

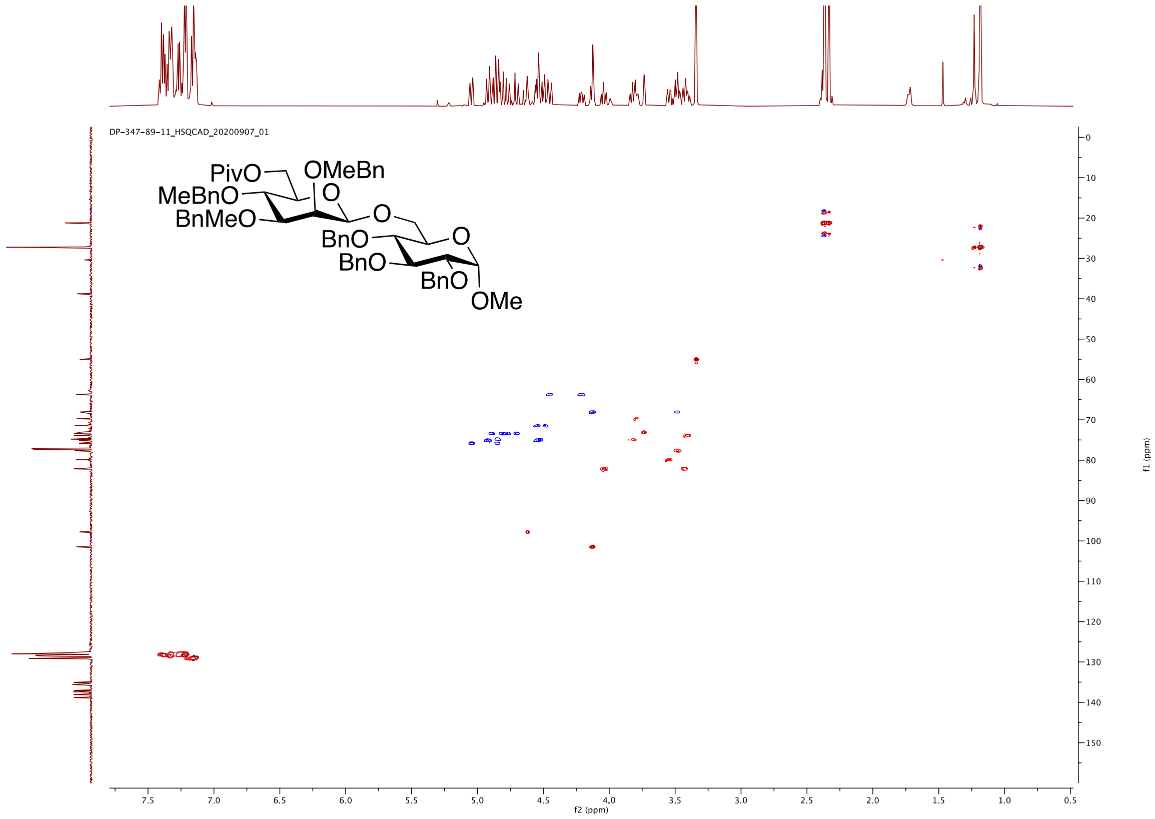

^{13}C- ^{1}H coupled HSQC NMR (500 MHz x 126 MHz, Chloroform-*d*) 2i

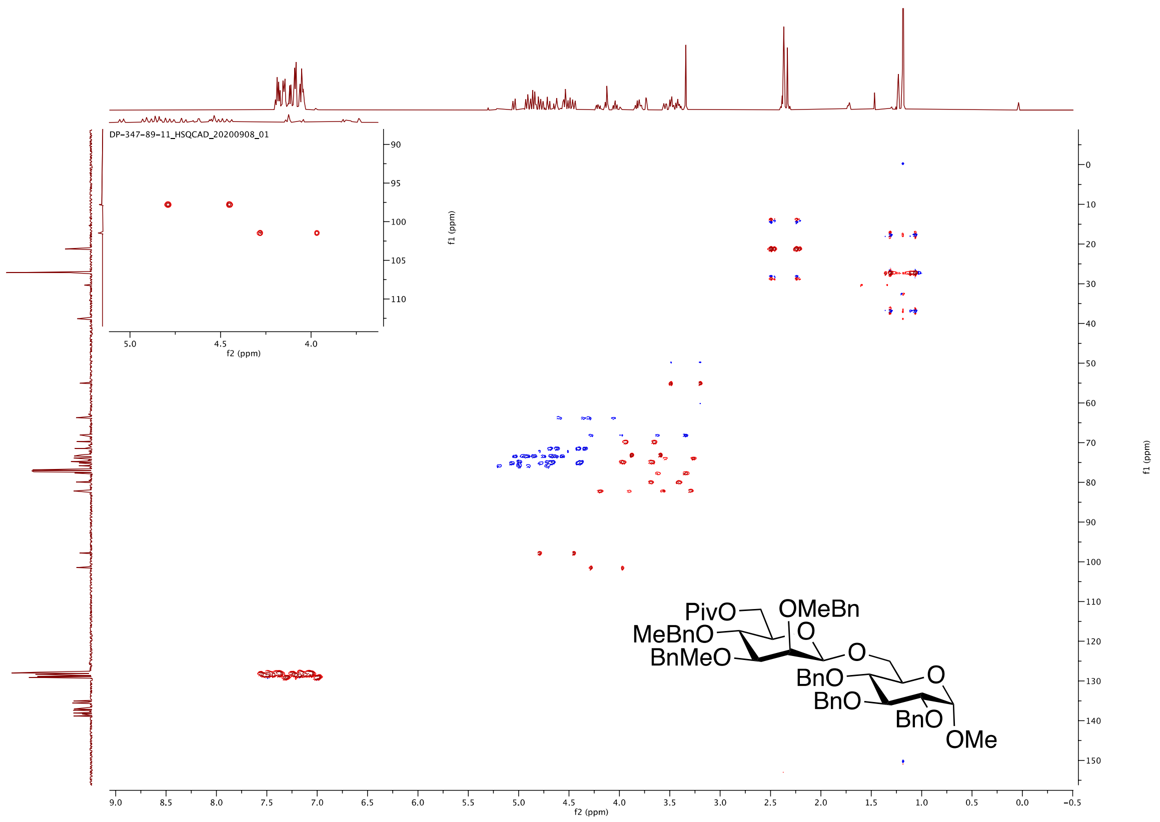

HMBC NMR (500 MHz x 126 MHz, Chloroform-*d*) 2i

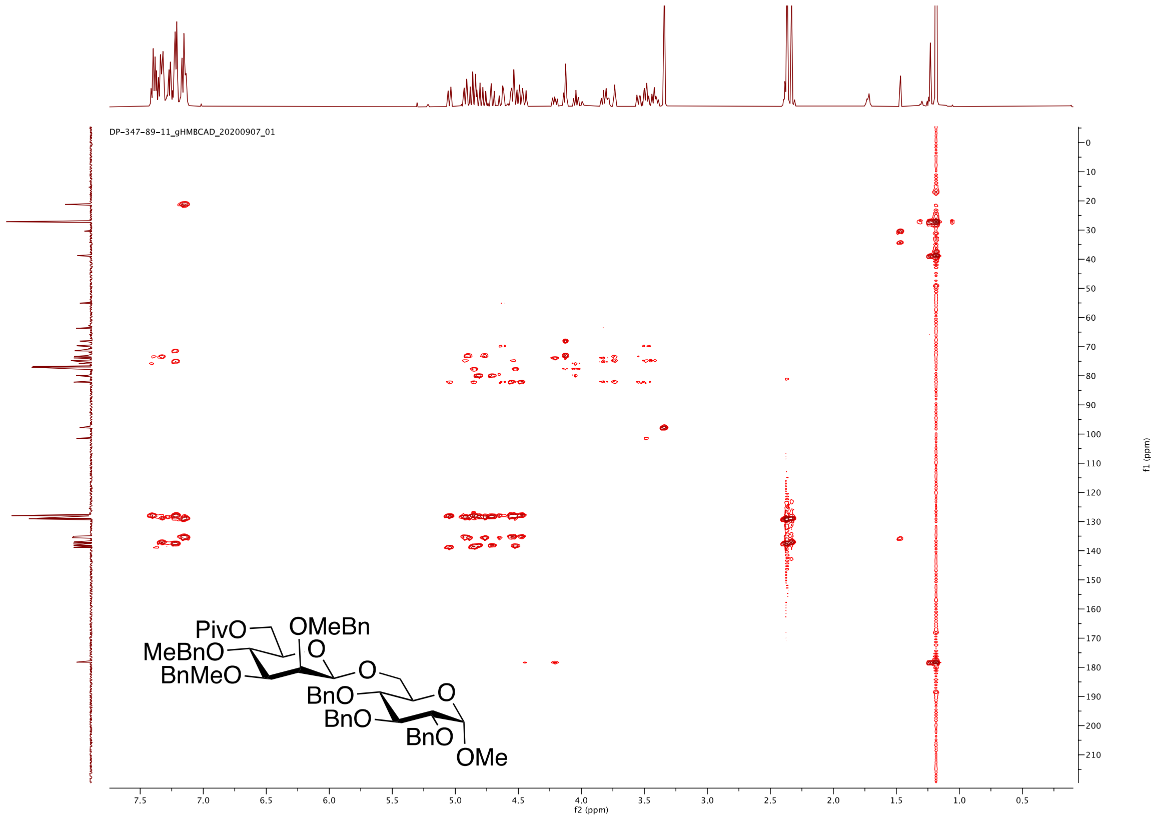

^{1}H NMR (600 MHz, Chloroform-*d*) 2j

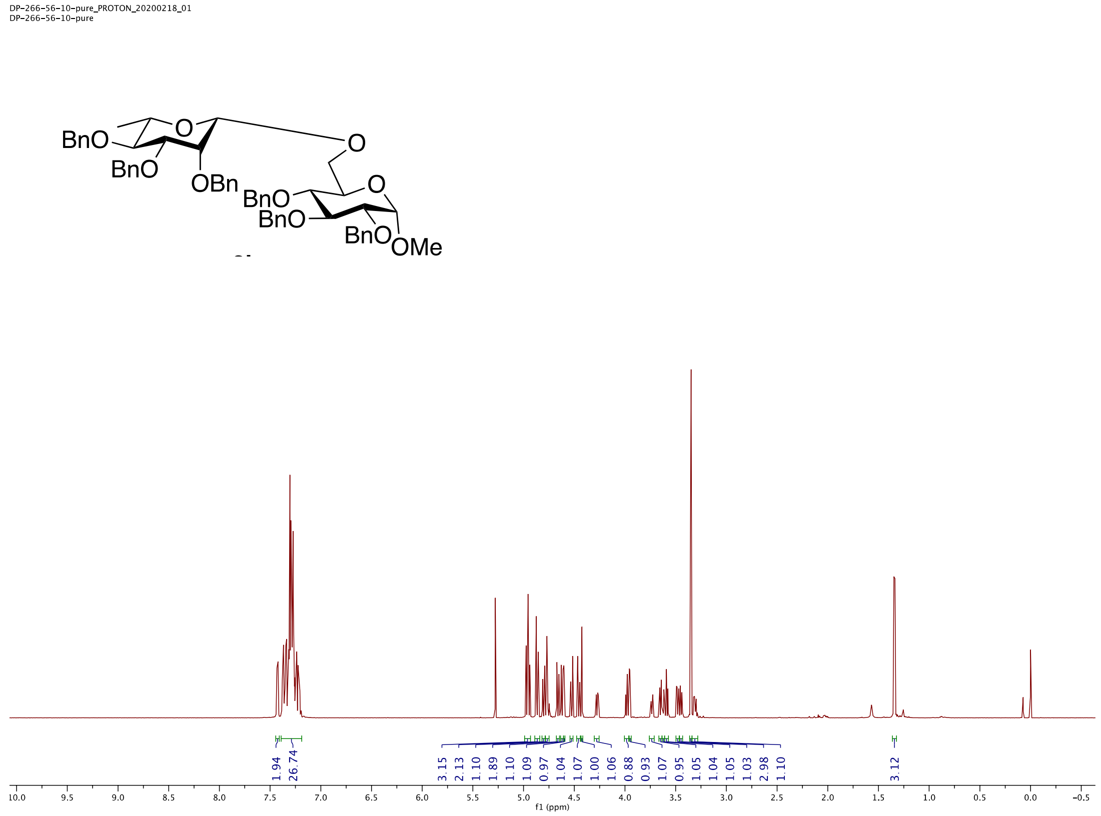

^{13}C NMR (151 MHz, Chloroform-*d*) 2j

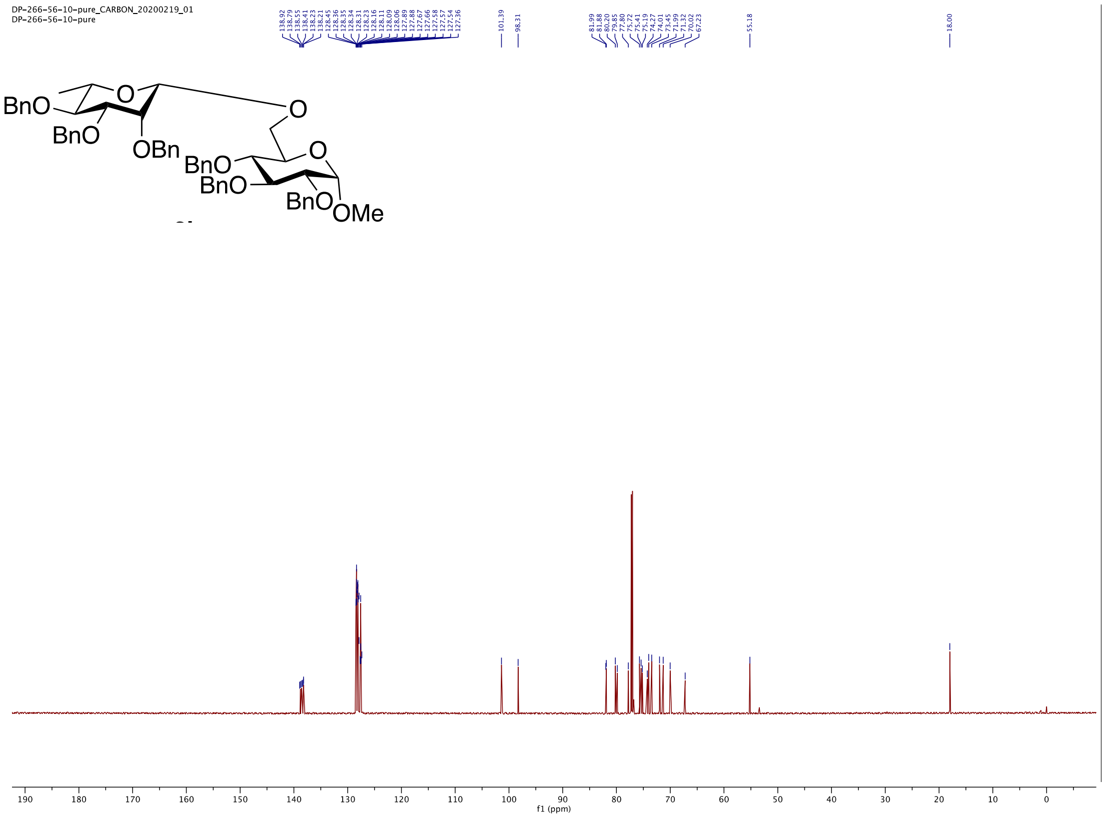

^{13}C - ^{1}H decoupled HSQC (600 x 151 MHz, Chloroform-*d*) 2j

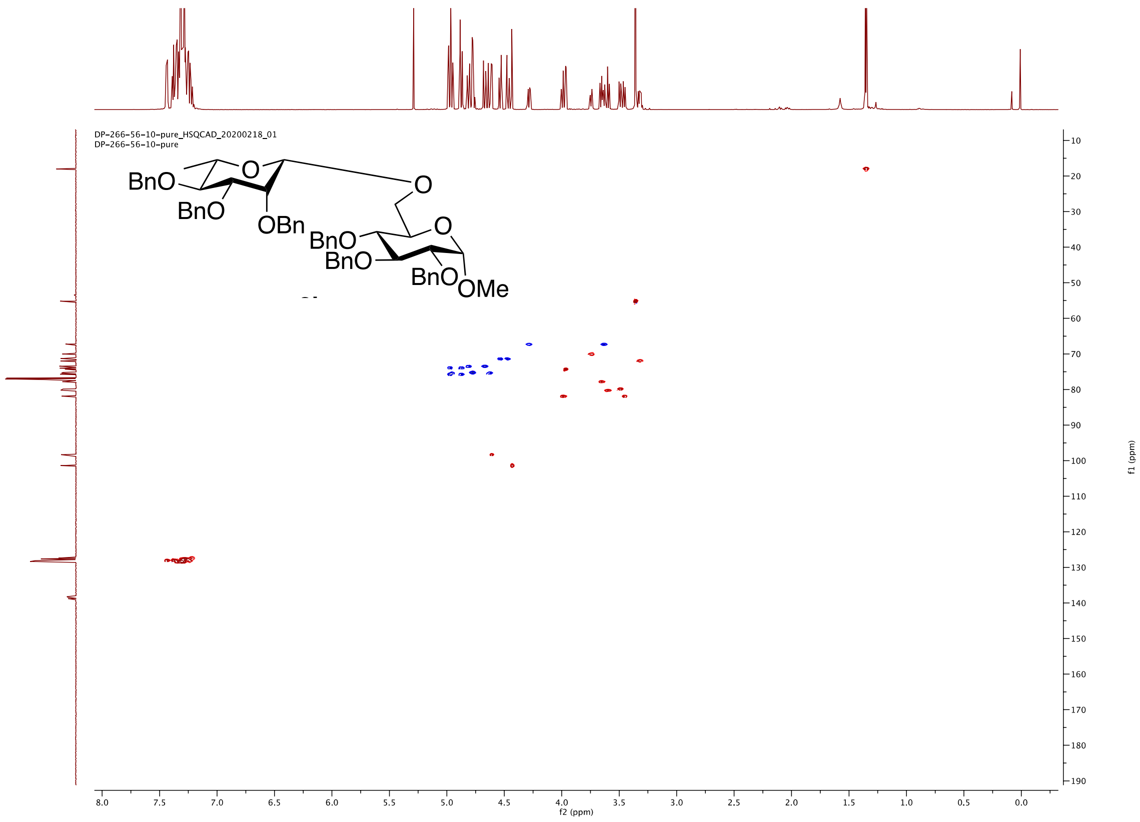

COSY (600 x 600 MHz, Chloroform-*d*) 2j

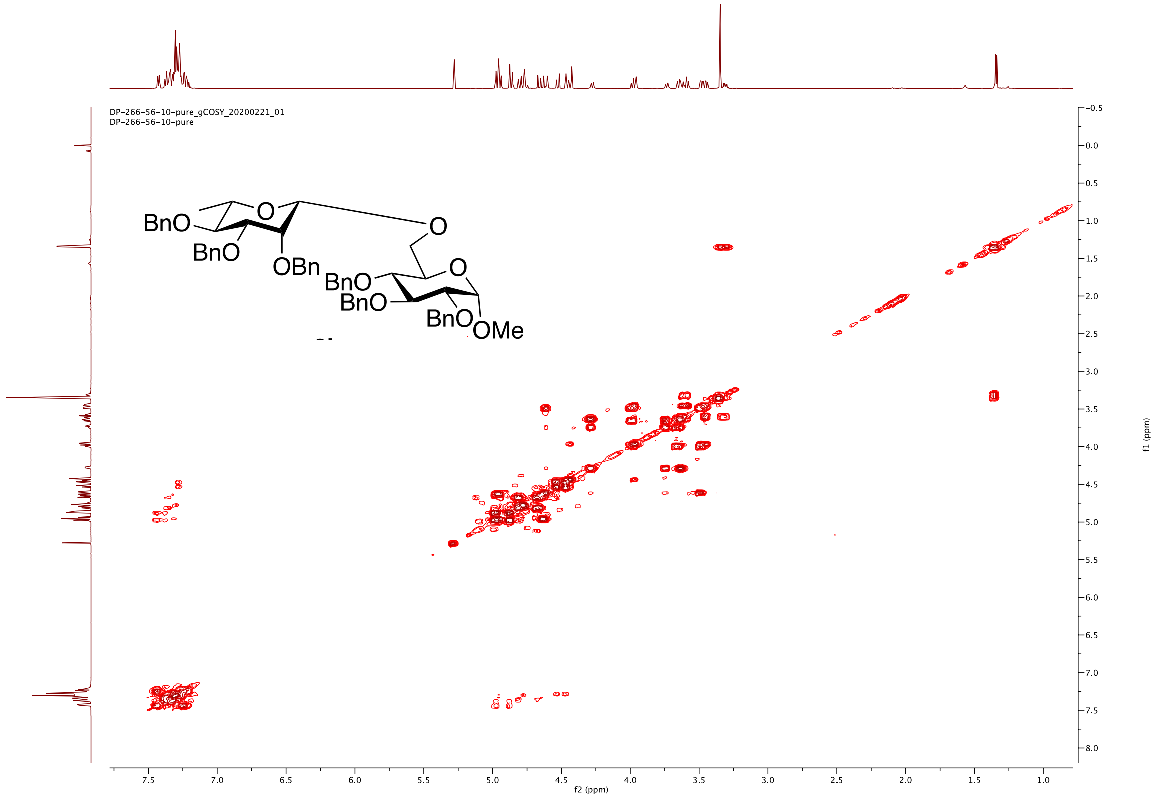

HMBC (600 x 151 MHz, Chloroform-*d*) 2j

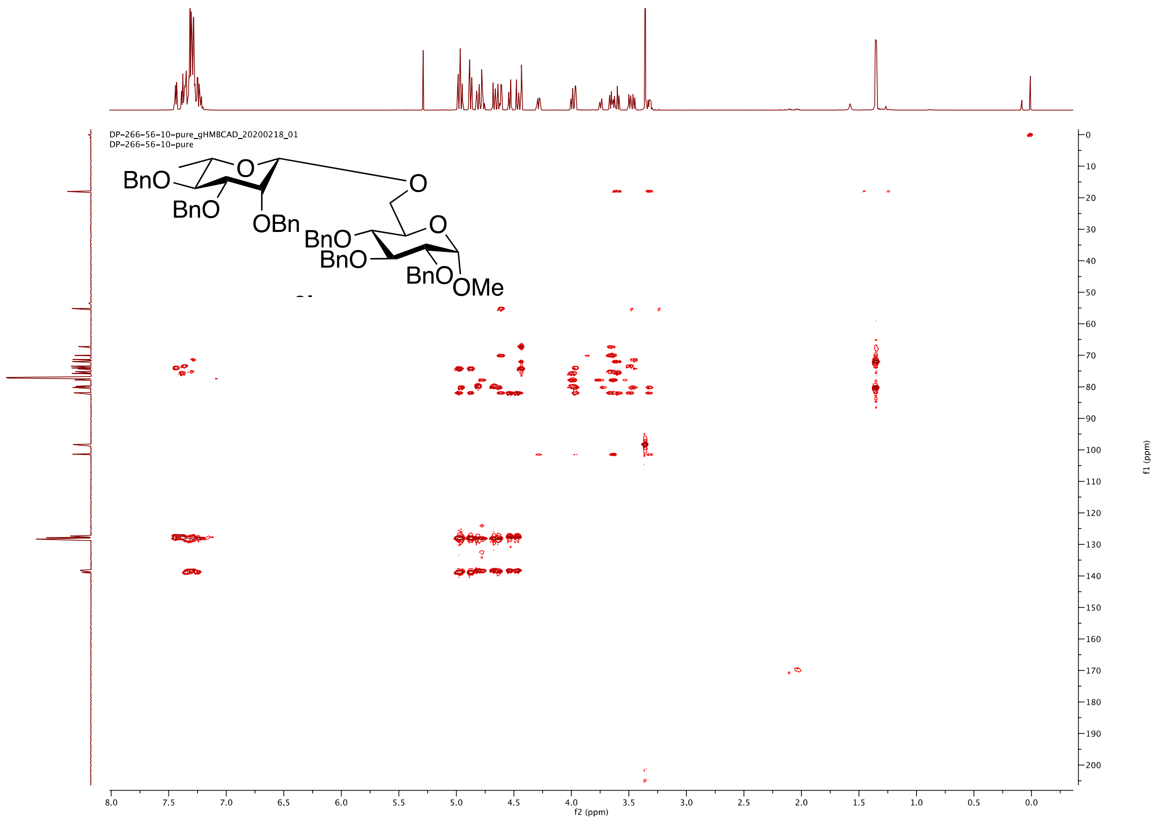

^{1}H NMR (600 MHz, Chloroform-*d*) 2k

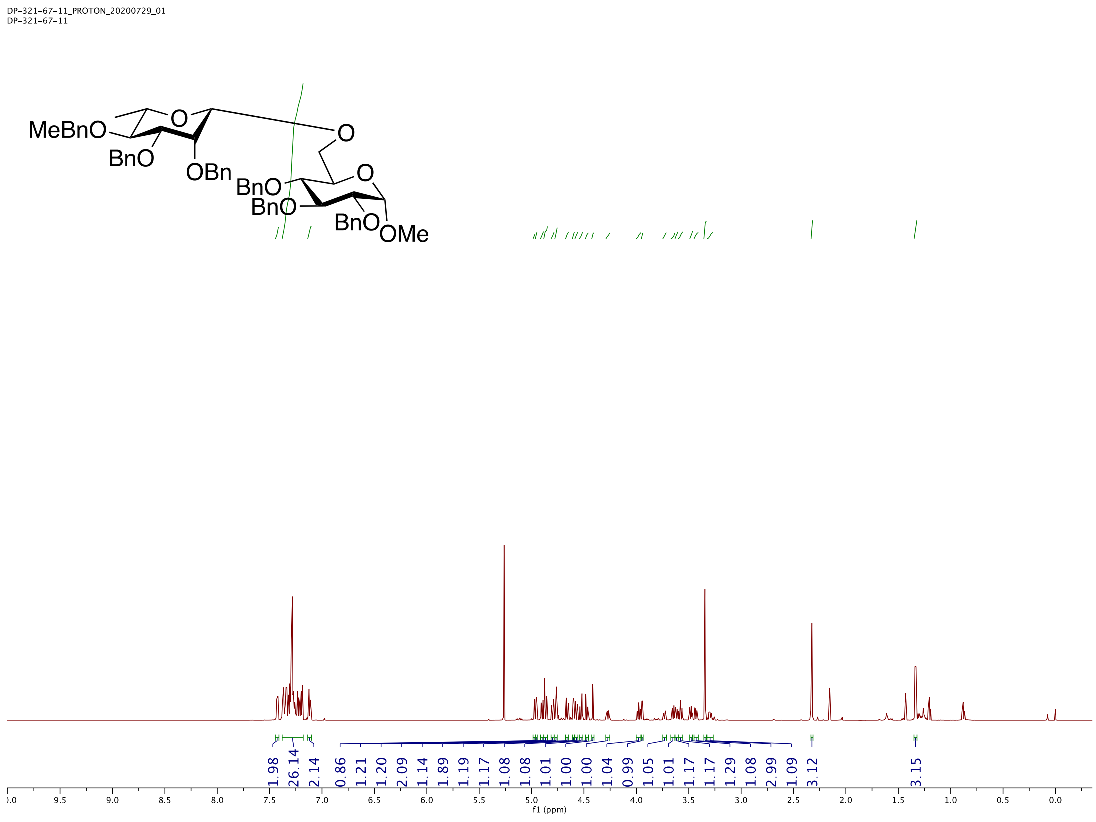

^{13}C NMR (151 MHz, Chloroform-*d*) 2k

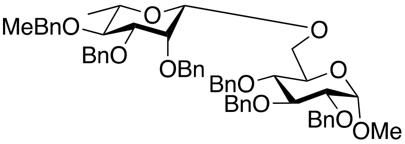

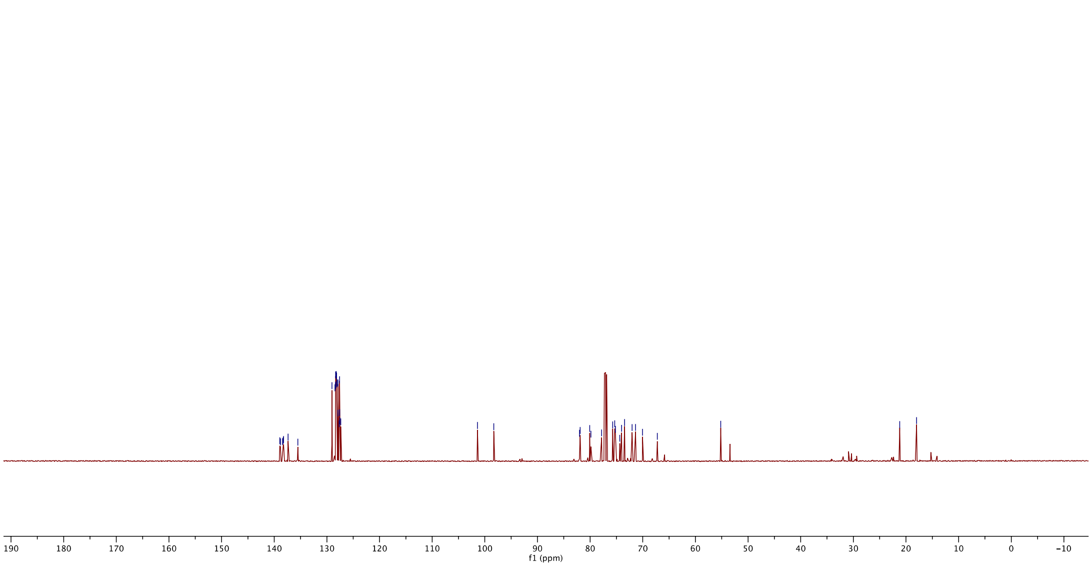

^{13}C - ^{1}H decoupled HSQC (600 x 151 MHz, Chloroform-*d*) 2k

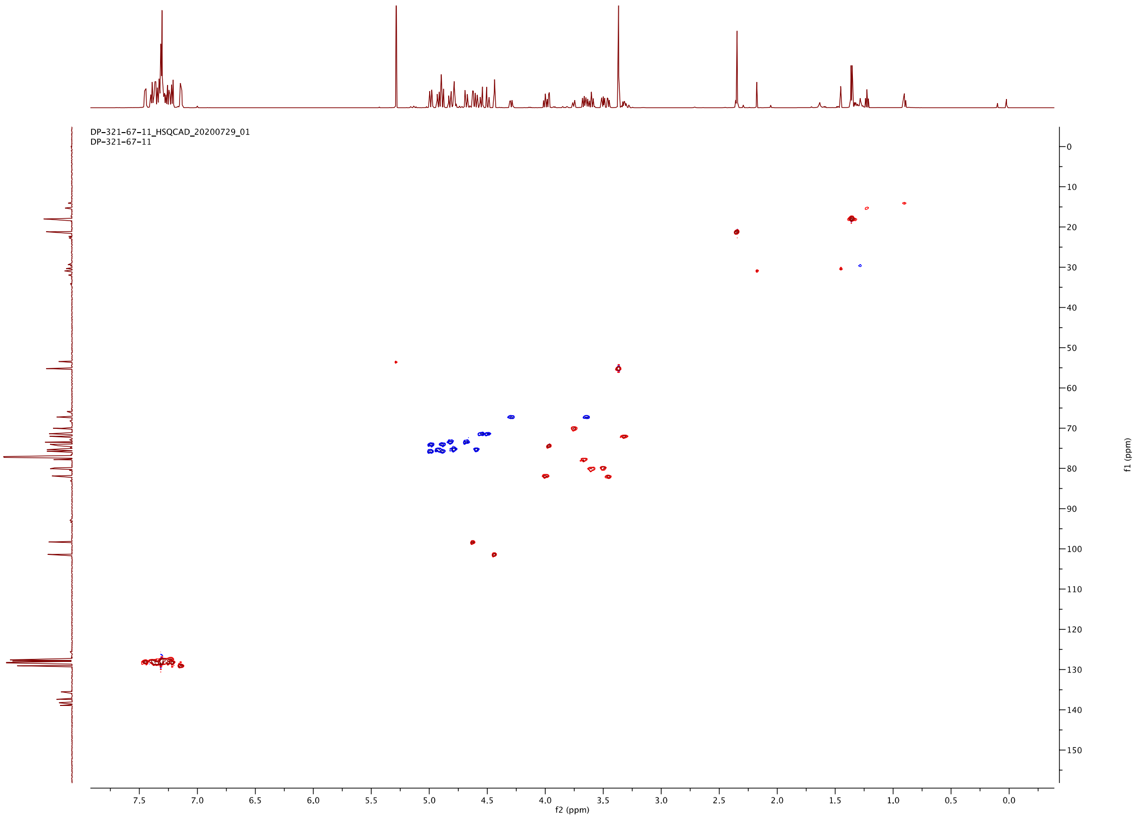

^{13}C - ^{1}H coupled HSQC (600 x 151 MHz, Chloroform-*d*) 2k

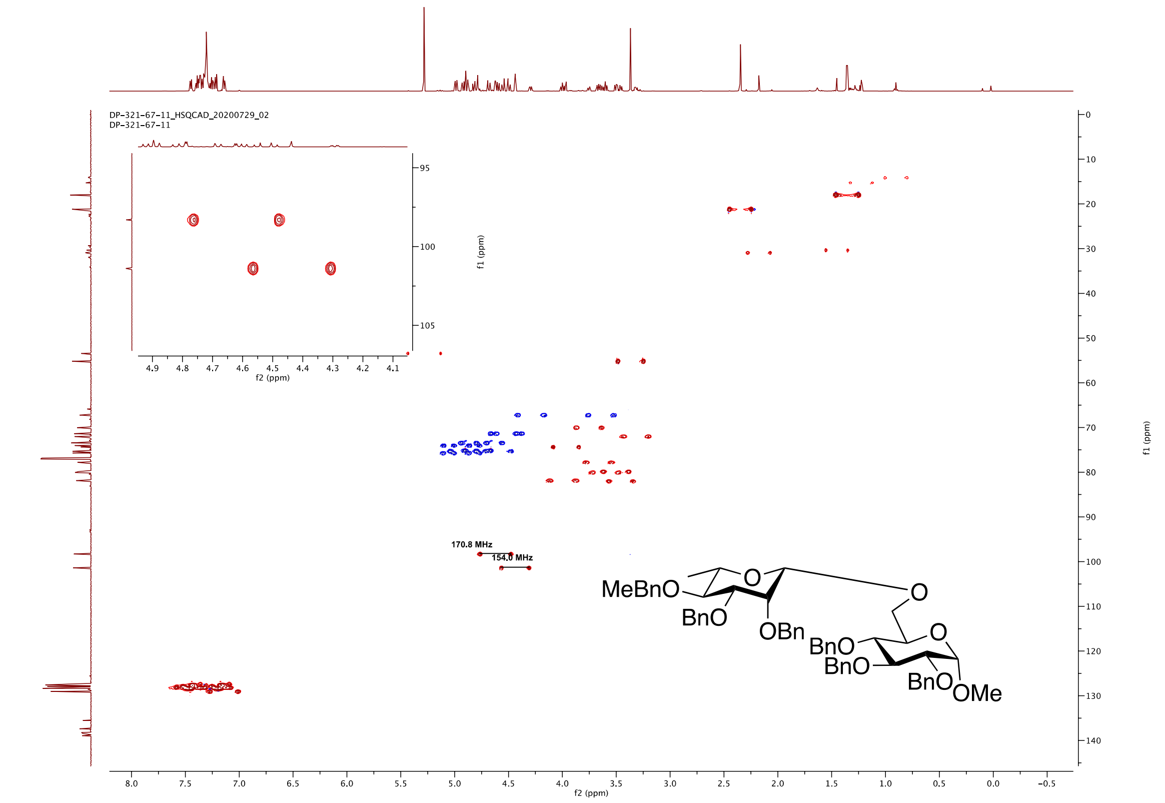

COSY (600 x 600 MHz, Chloroform-*d*) 2k

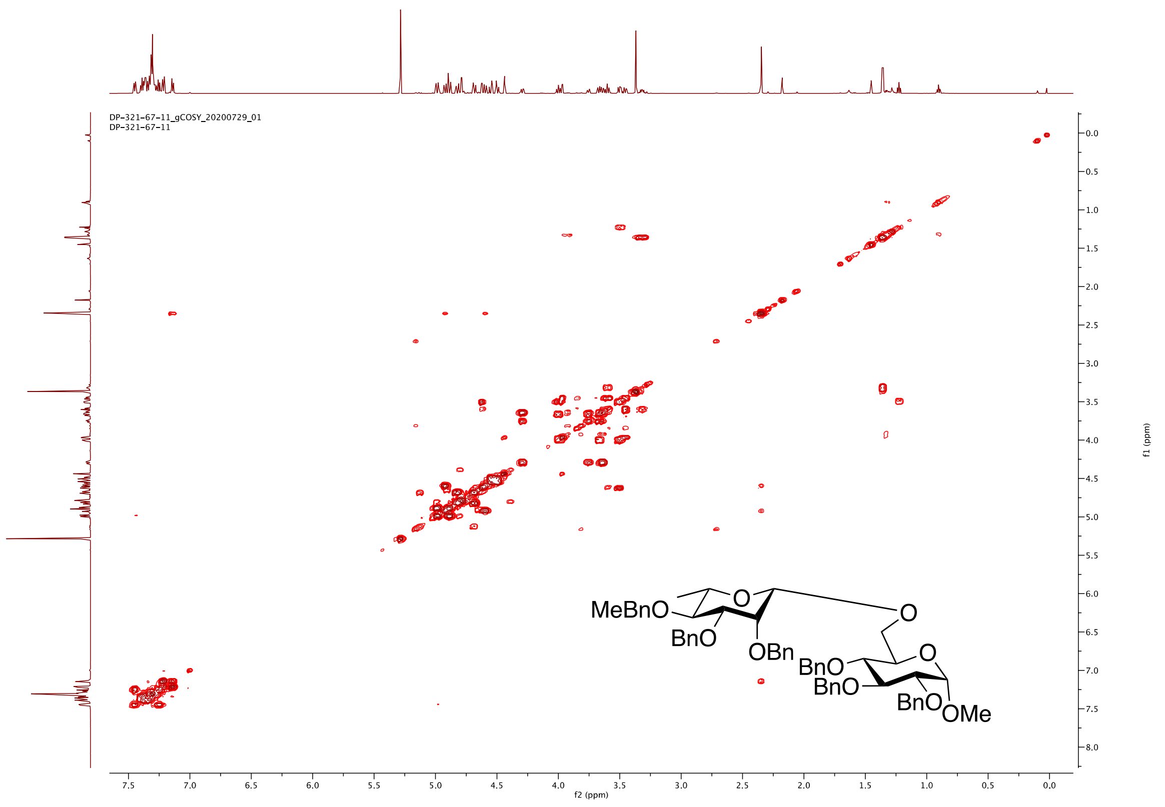

HMBC (600 x 151 MHz, Chloroform-*d*) 2k

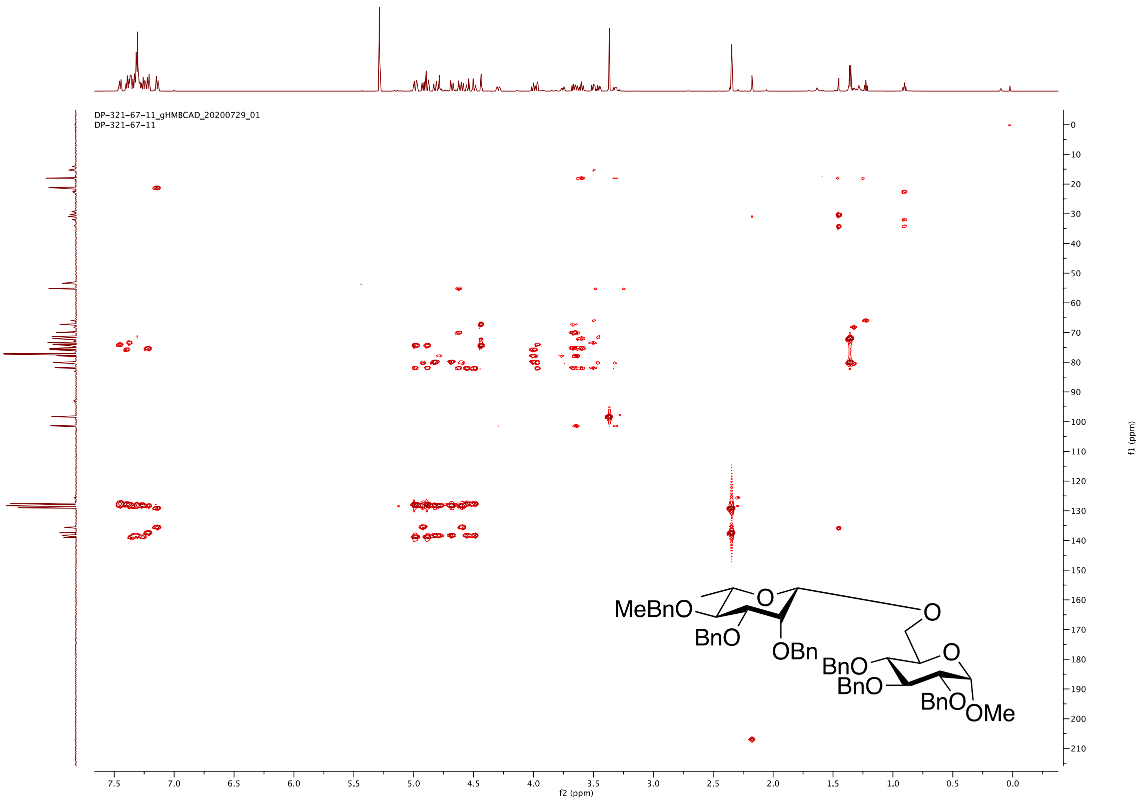

^{1}H NMR (500 MHz, Chloroform-*d*) 2l

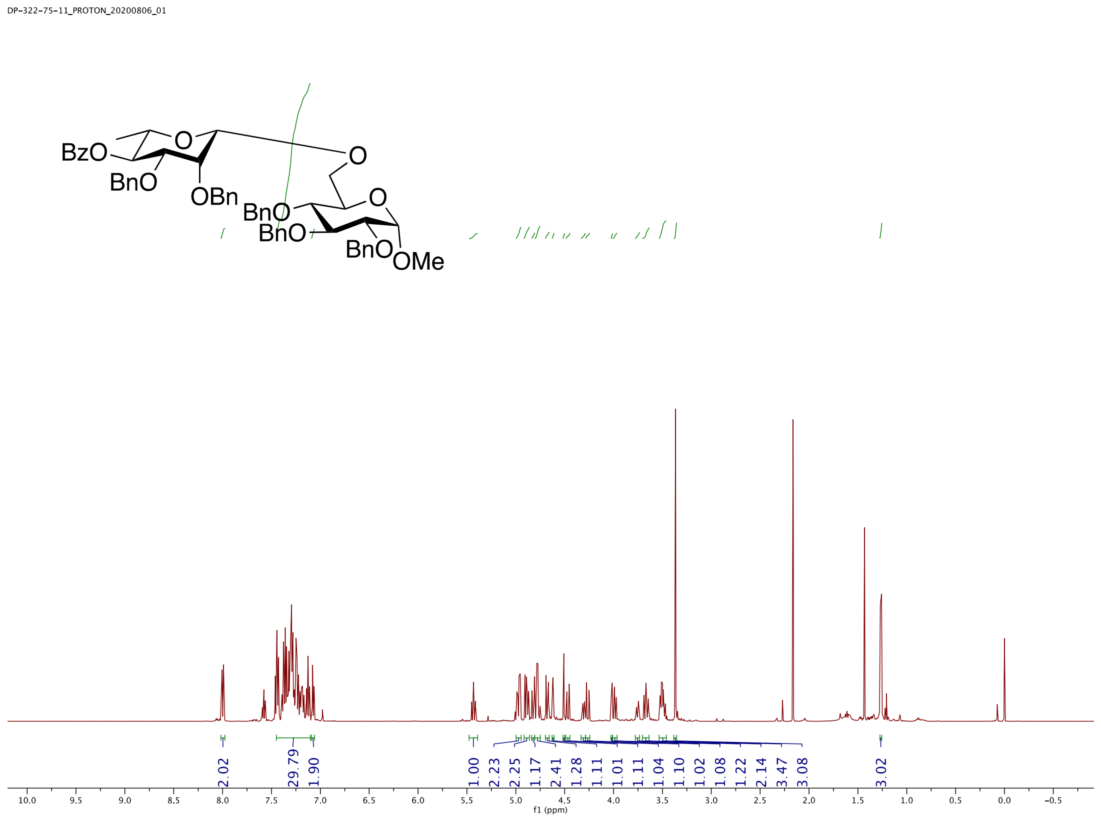

^{13}C NMR (126 MHz, Chloroform-*d*) 2l

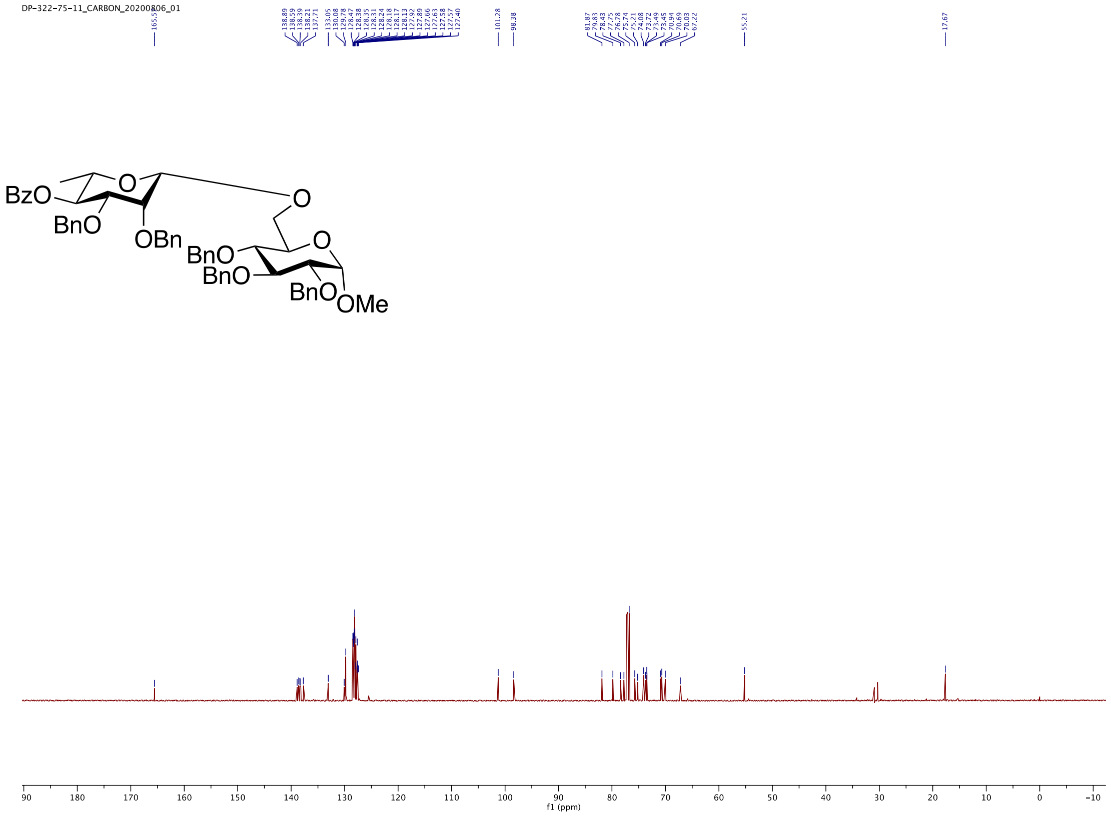

^{13}C - ^{1}H decoupled HSQC (500 x 126 MHz, Chloroform-*d*) 2l

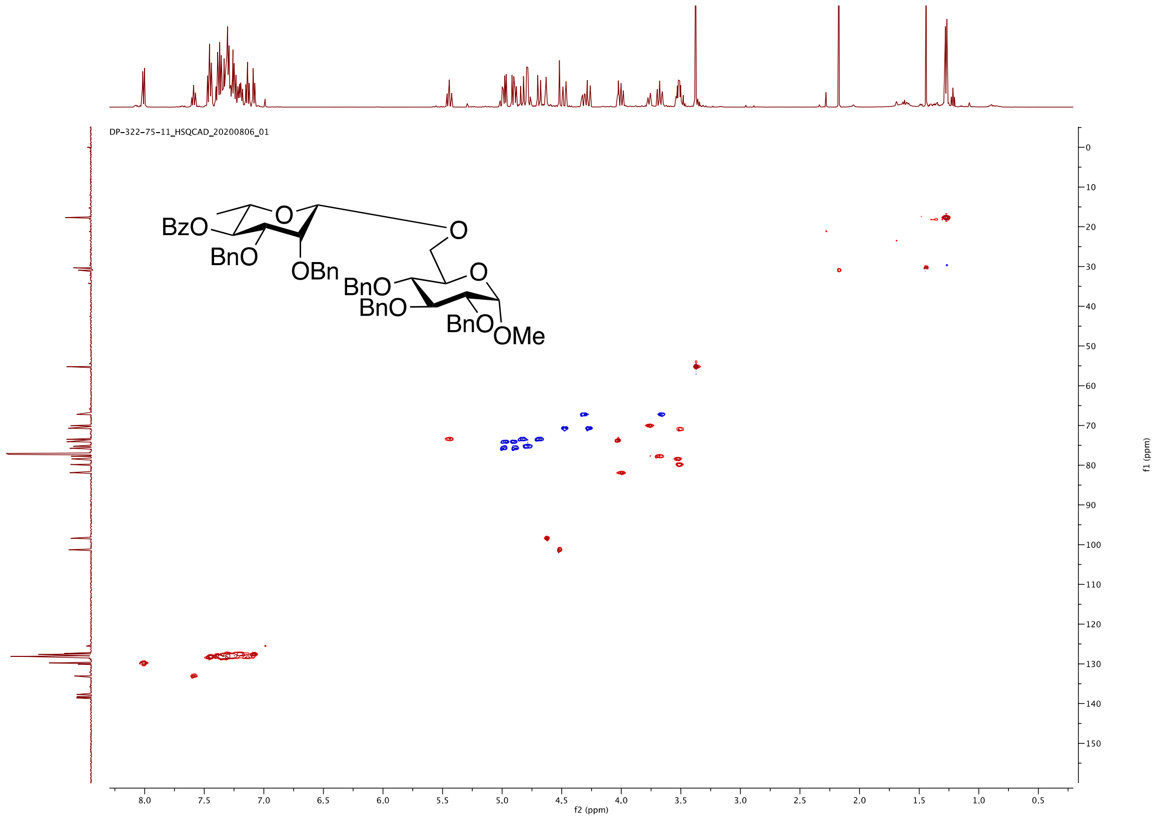

^{13}C - ^{1}H coupled HSQC (500 x 126 MHz, Chloroform-*d*) 2l

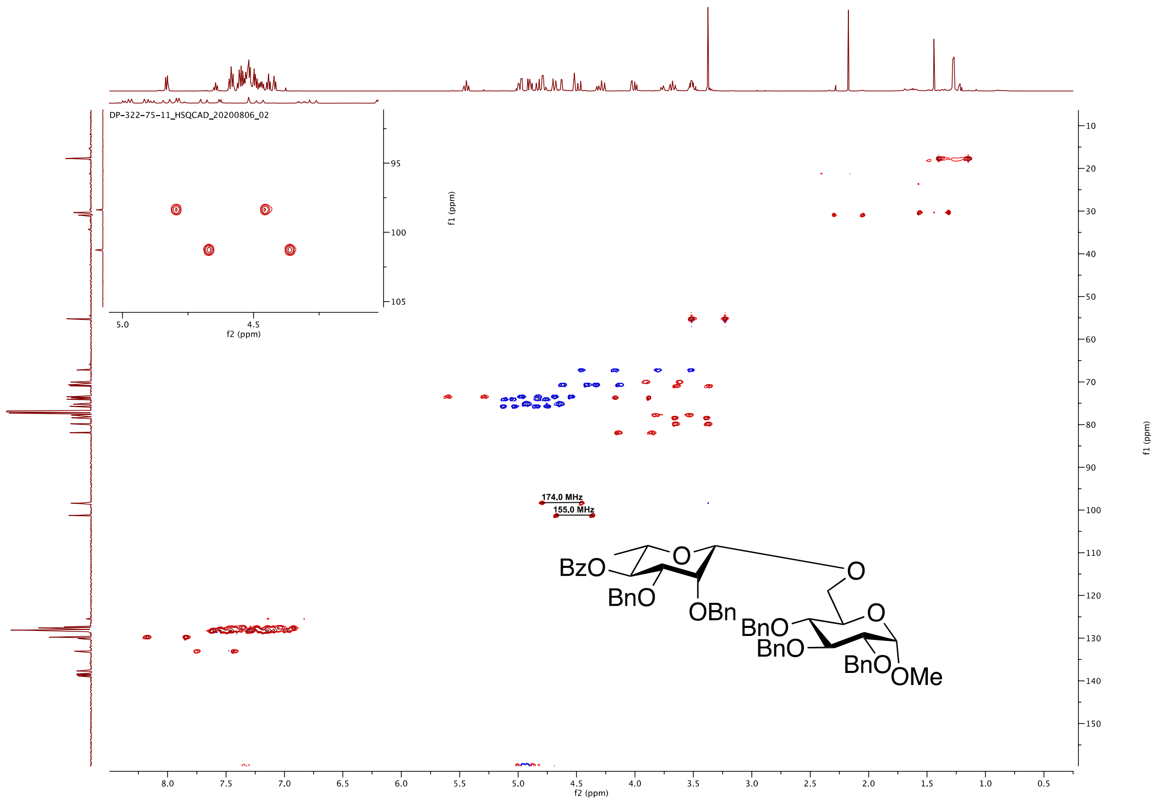

COSY (500 x 500 MHz, Chloroform-*d*) 2l

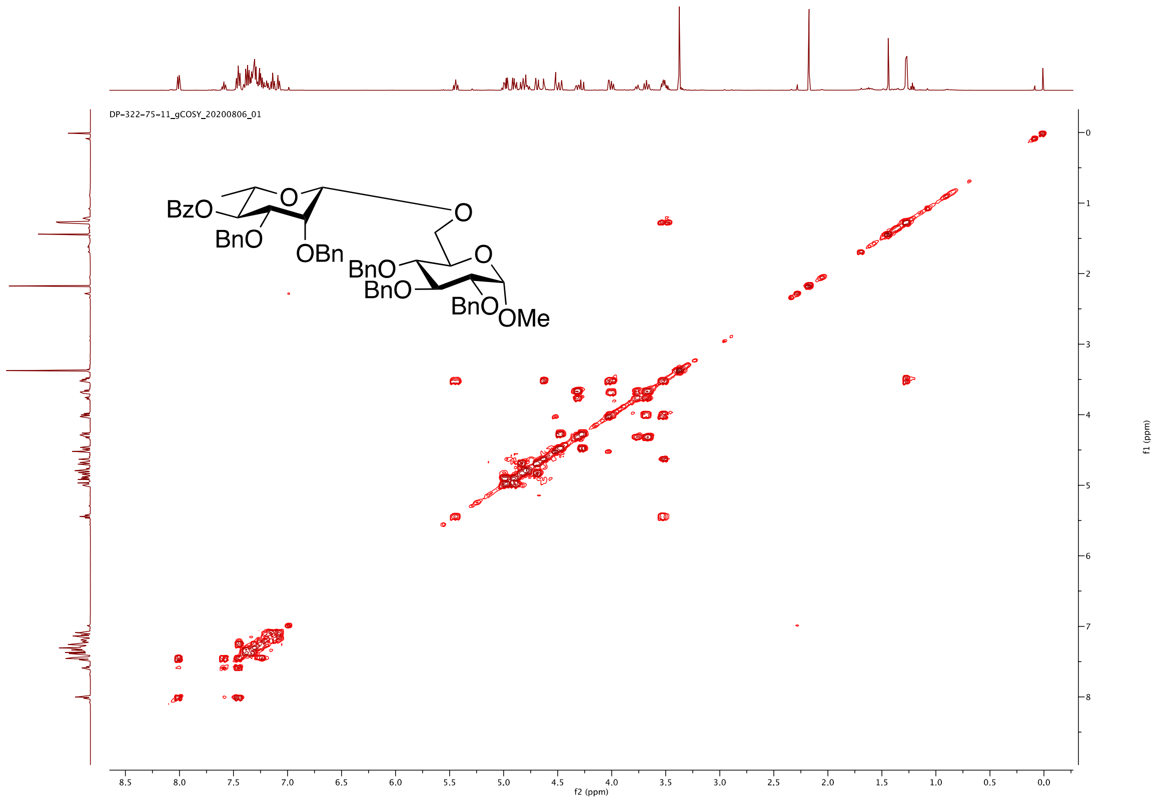

HMBC (500 x 126 MHz, Chloroform-*d*) 2l

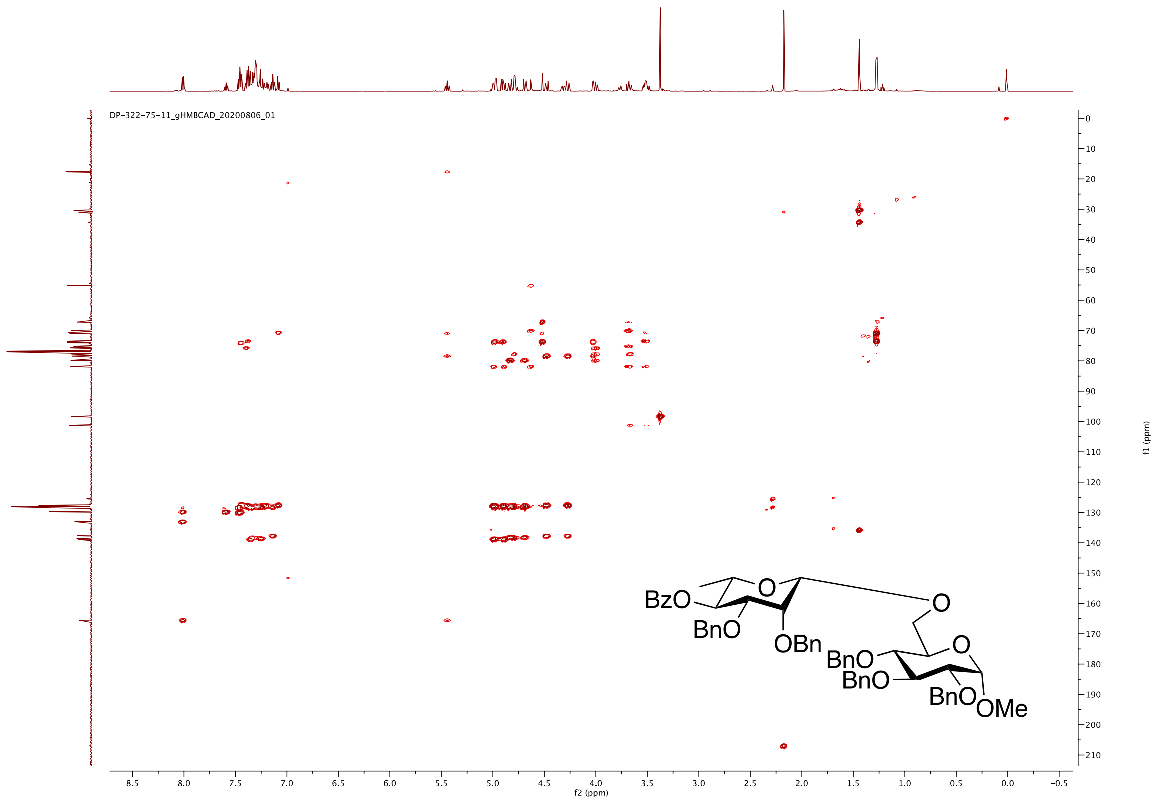

^{1}H NMR (500 MHz, Chloroform-*d*) 4b

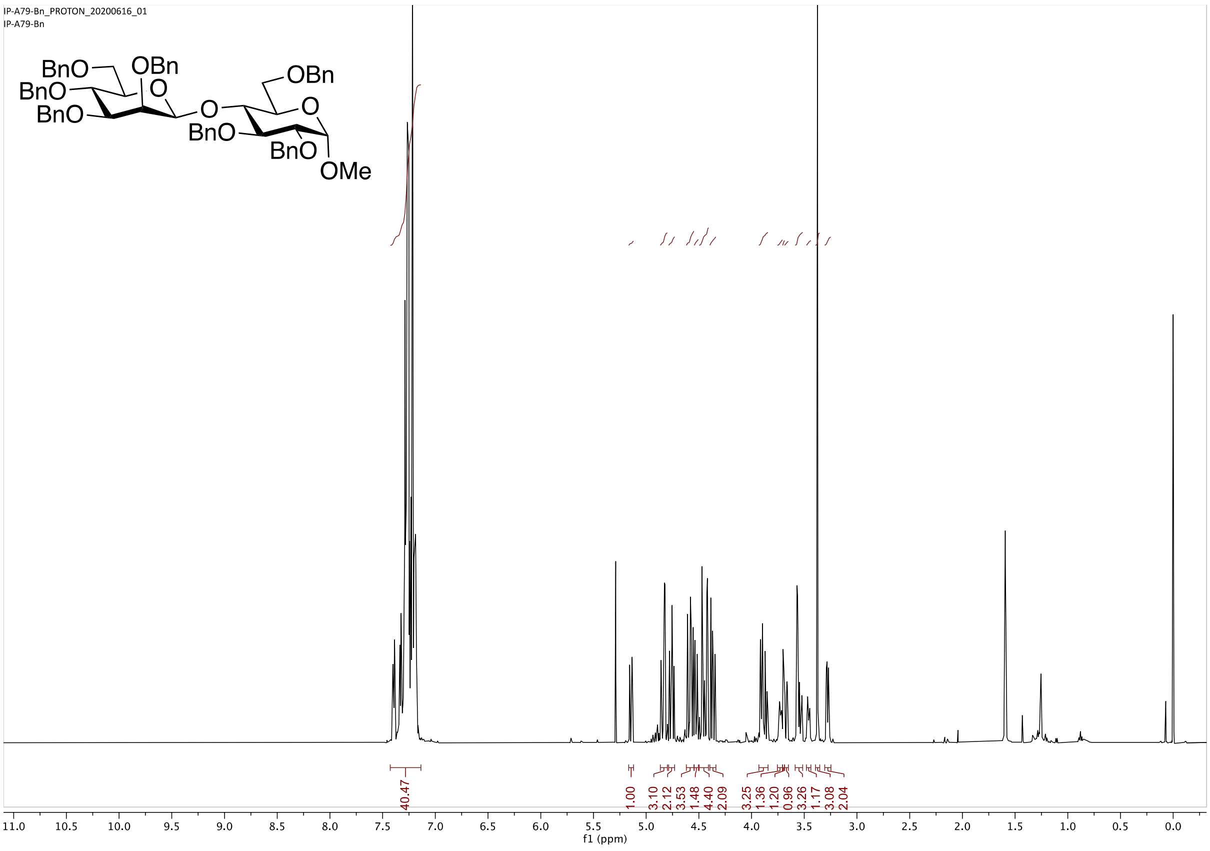

^{13}C NMR (126 MHz, Chloroform-*d*) 4b

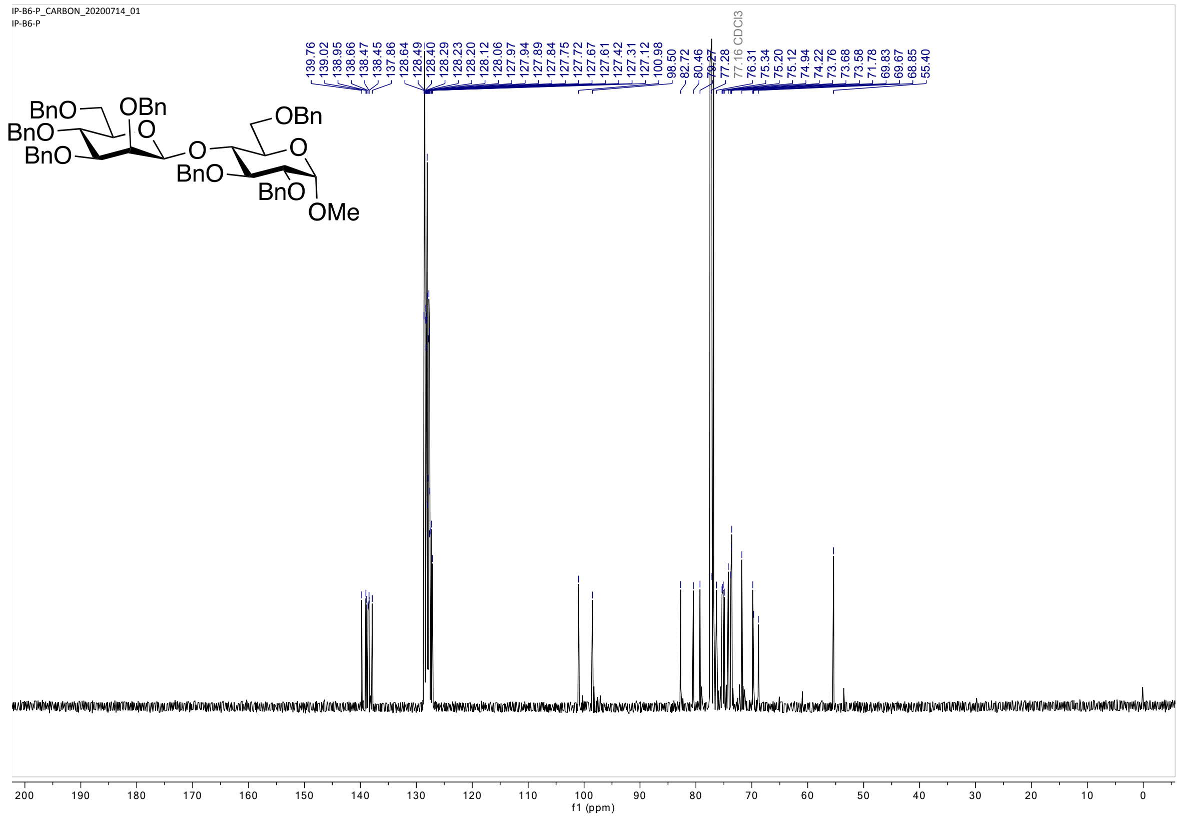

COSY NMR (500 MHz, Chloroform-*d*) 4b

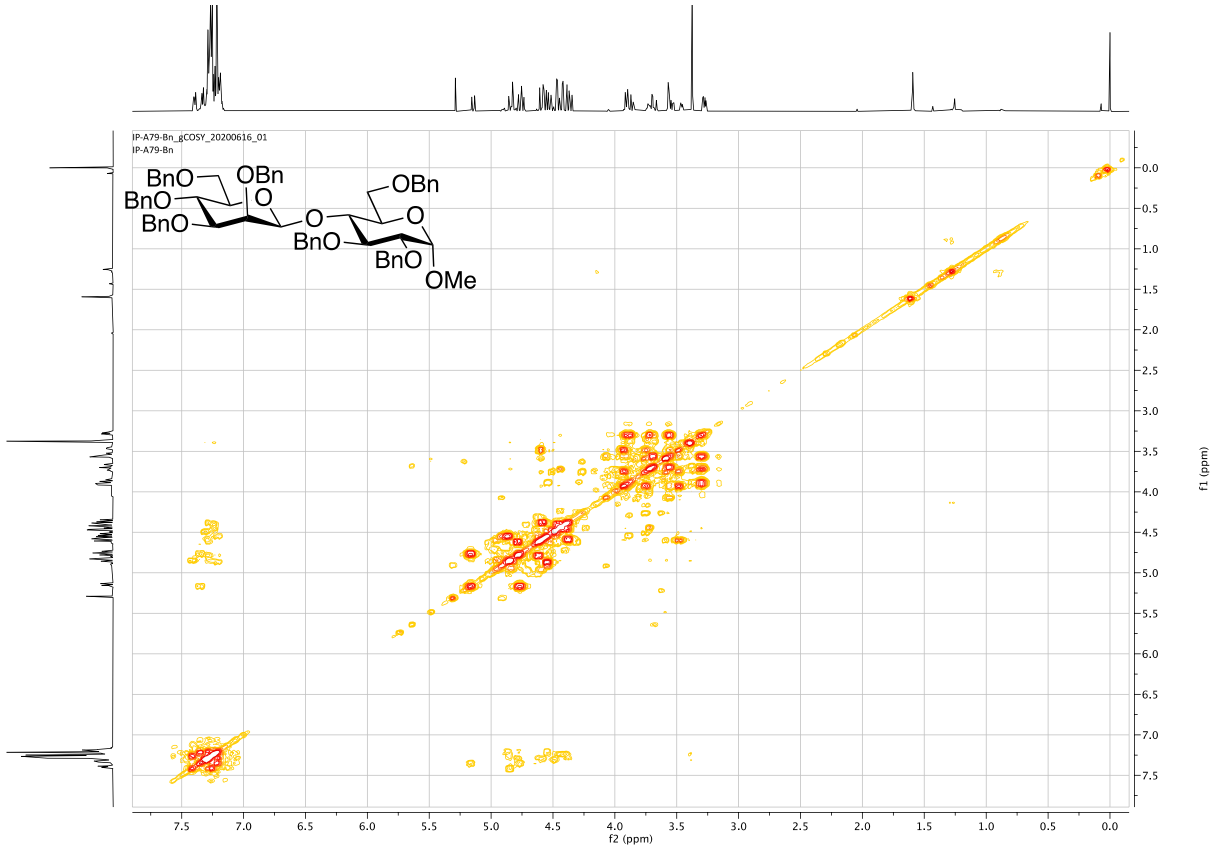

HSQC NMR (500 MHz x 126 MHz, Chloroform-*d*) 4b

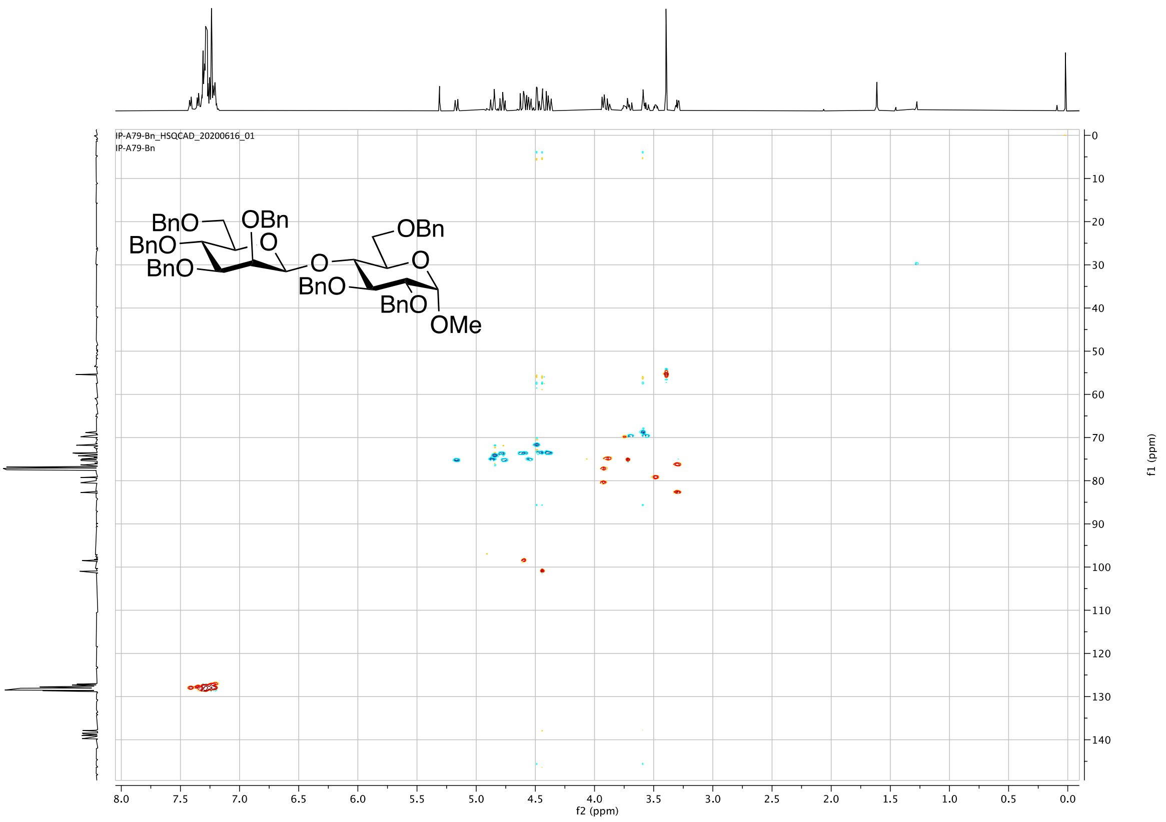

HMBC NMR (500 MHz x 126 MHz, Chloroform-*d*) 4b

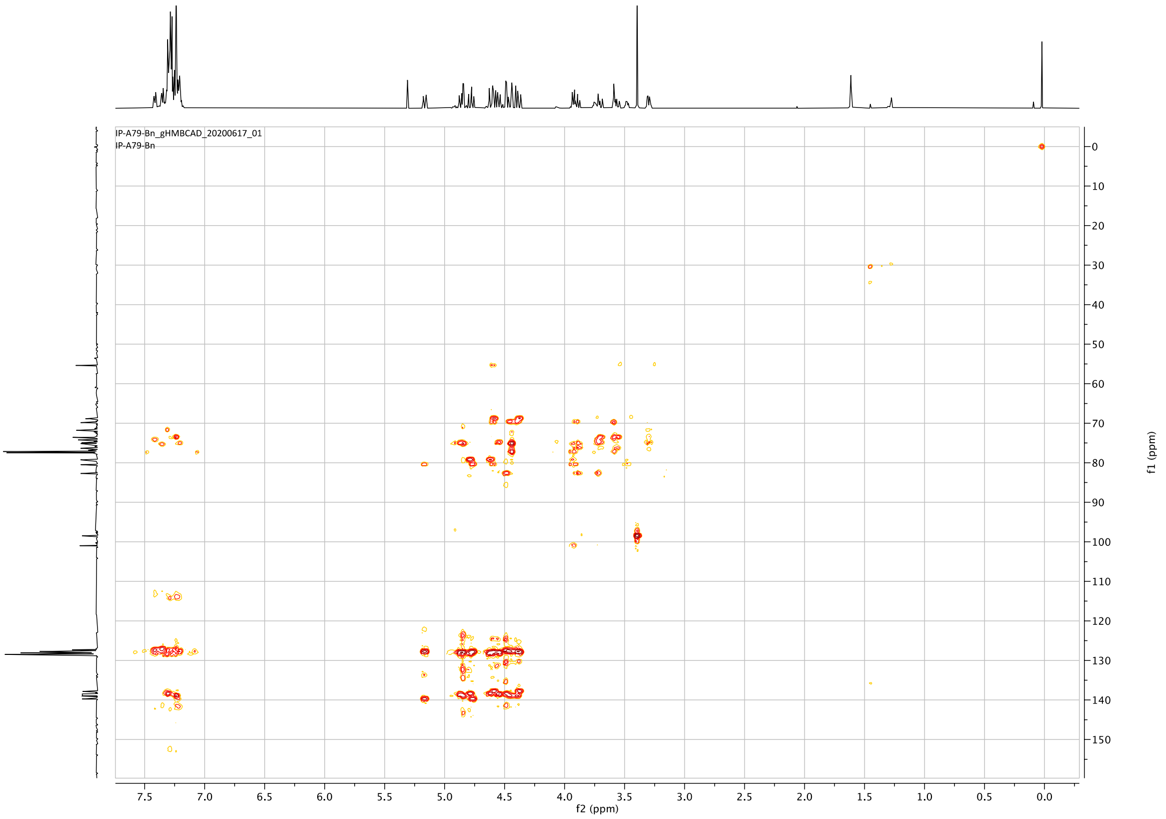

^{1}H NMR (500 MHz, Chloroform-*d*) 4c

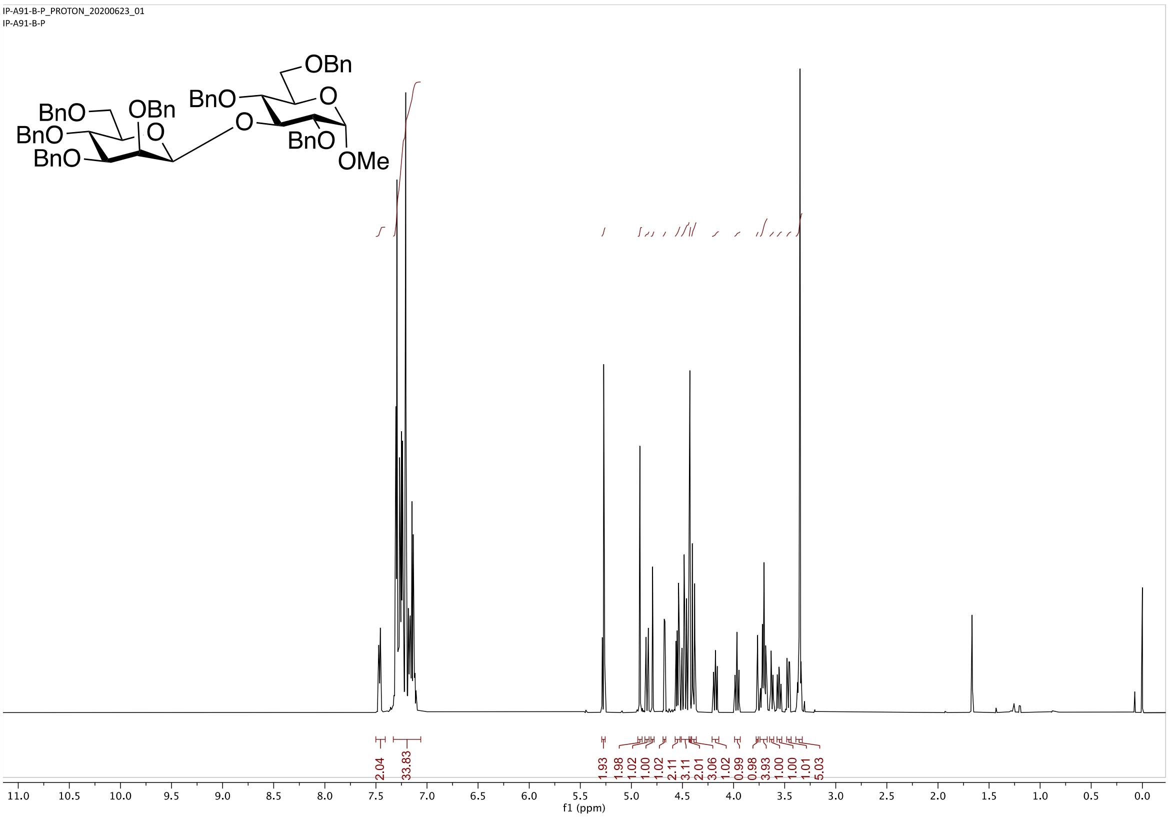

^{13}C NMR (126 MHz, Chloroform-*d*) 4c

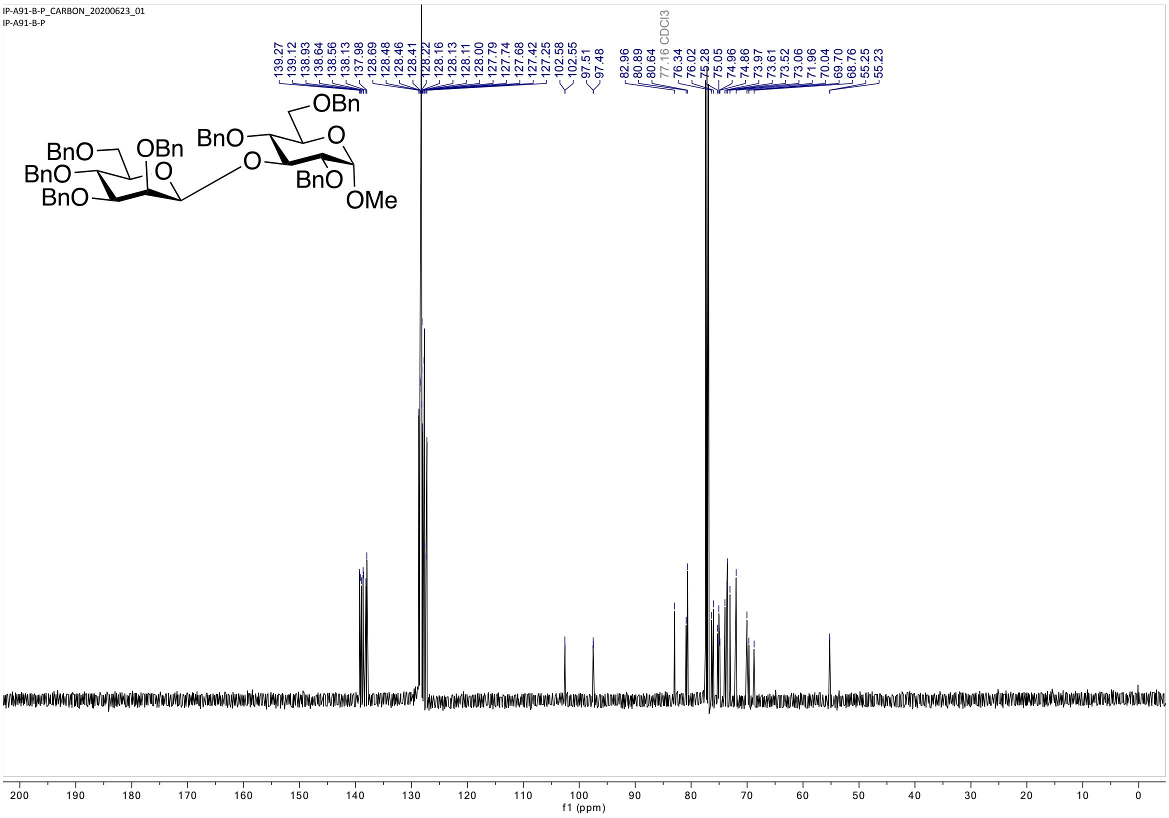

COSY NMR (500 MHz, Chloroform-*d*) 4c

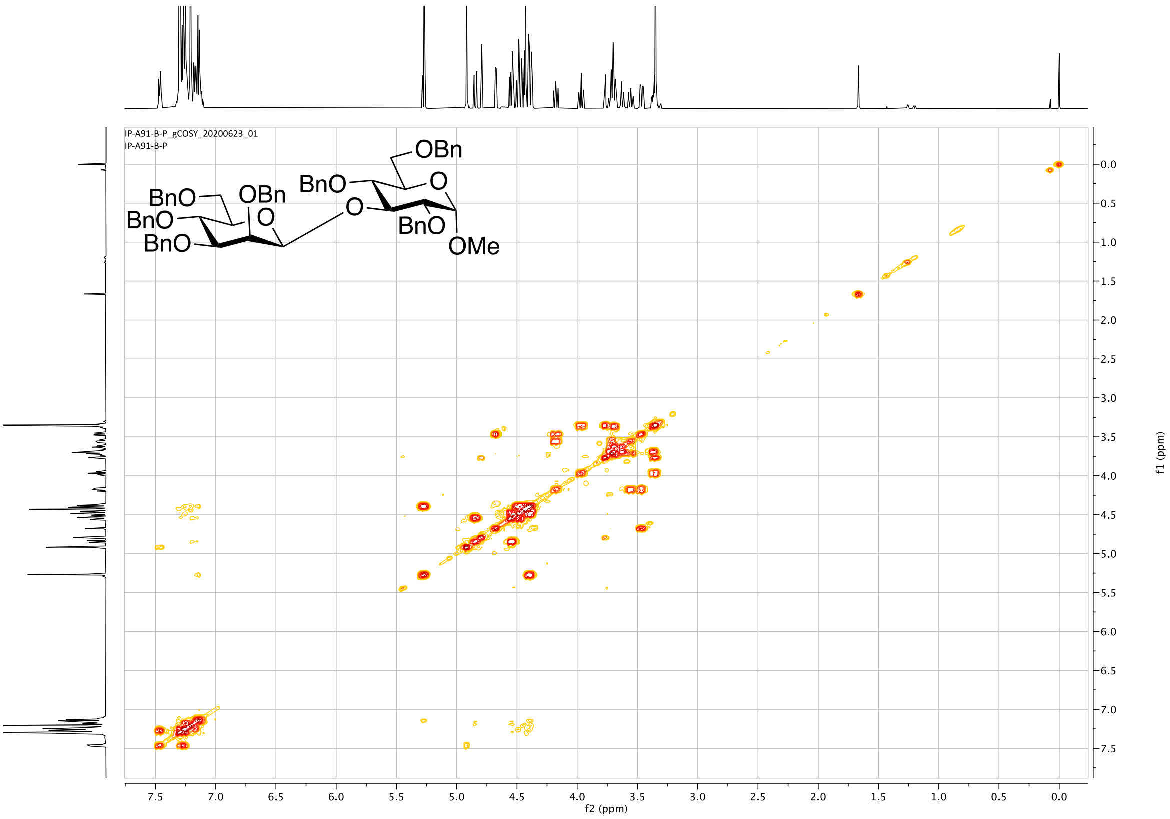

HSQC NMR (500 MHz x 126 MHz, Chloroform-*d*) 4c

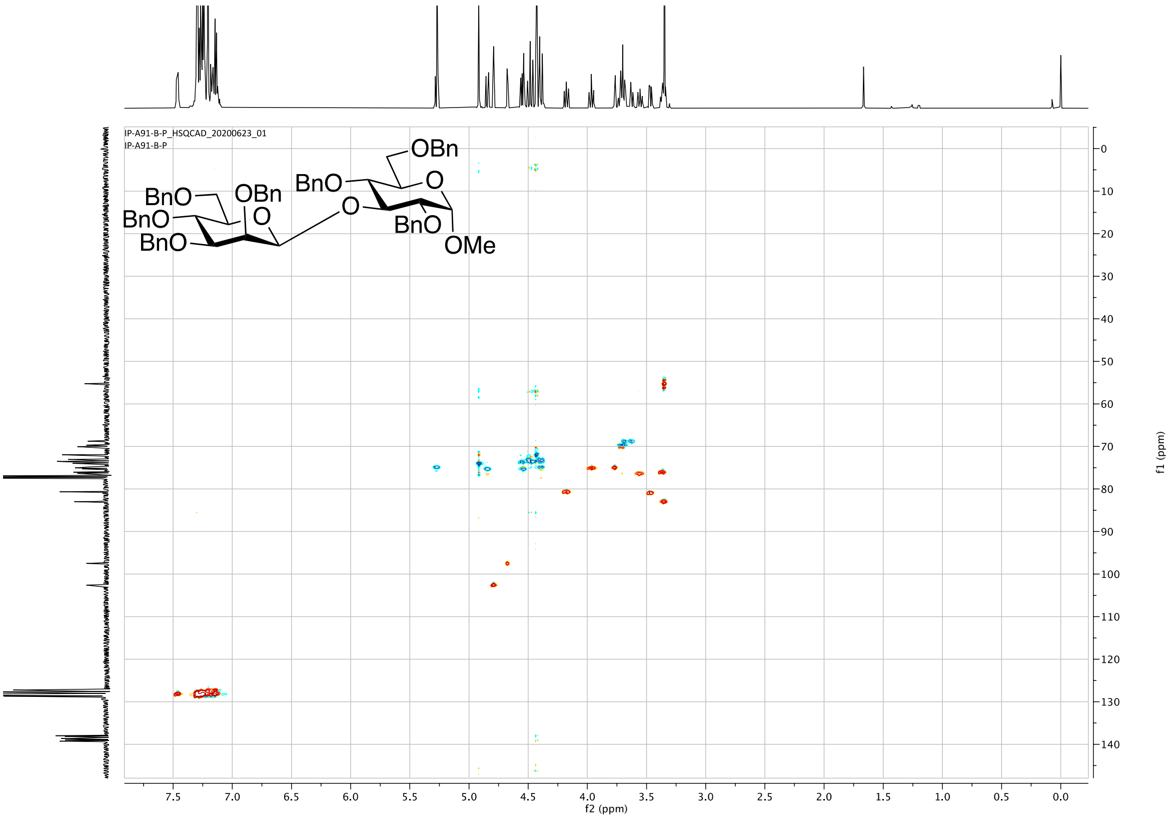

^{13}C- ^{1}H coupled HSQC NMR (500 MHz x 126 MHz, Chloroform-*d*) 4c

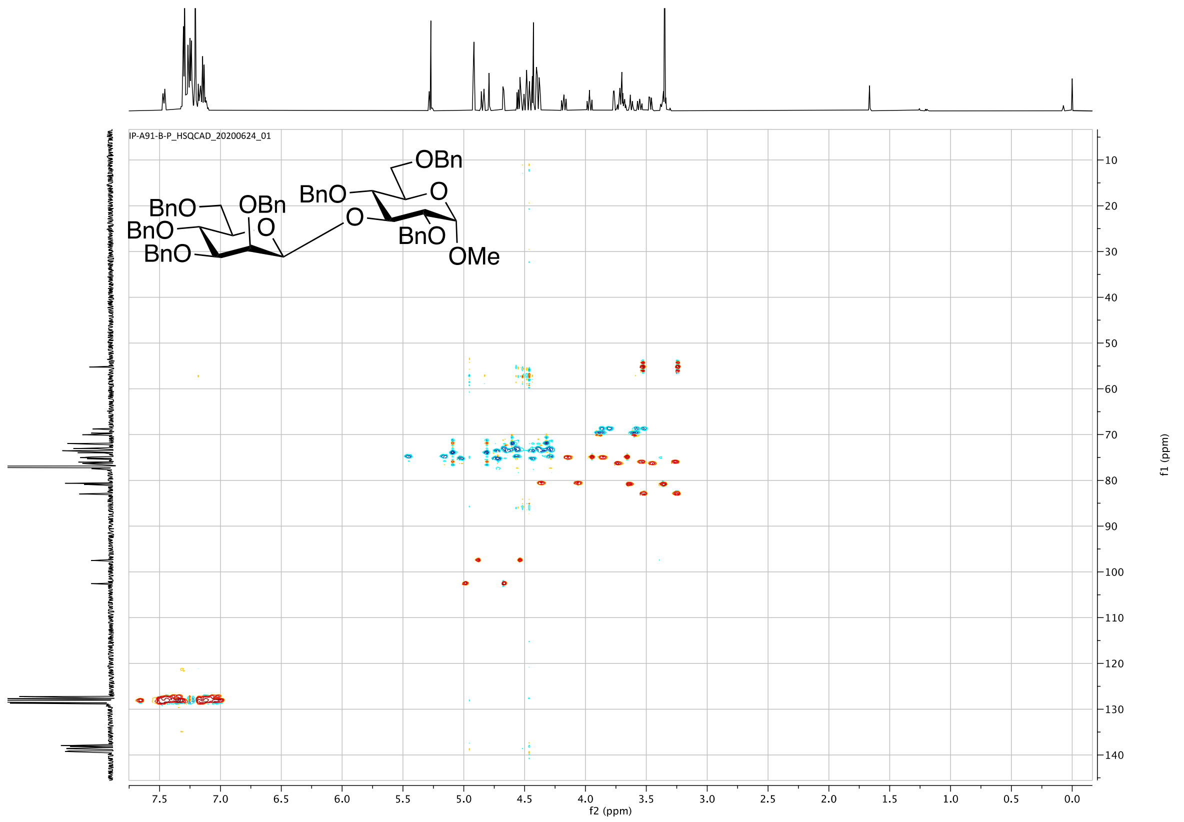

HMBC NMR (500 MHz x 126 MHz, Chloroform-*d*) 4c

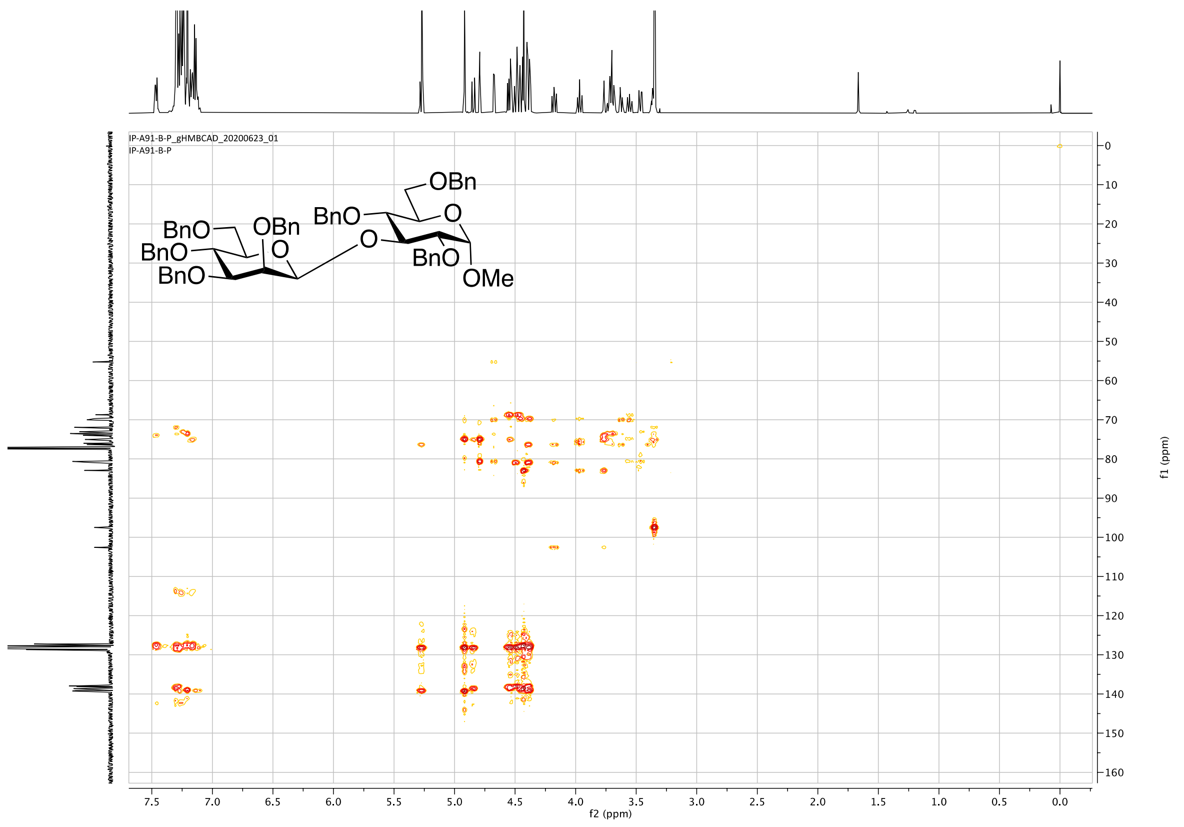

^{1}H NMR (600 MHz, Chloroform-*d*) 4d

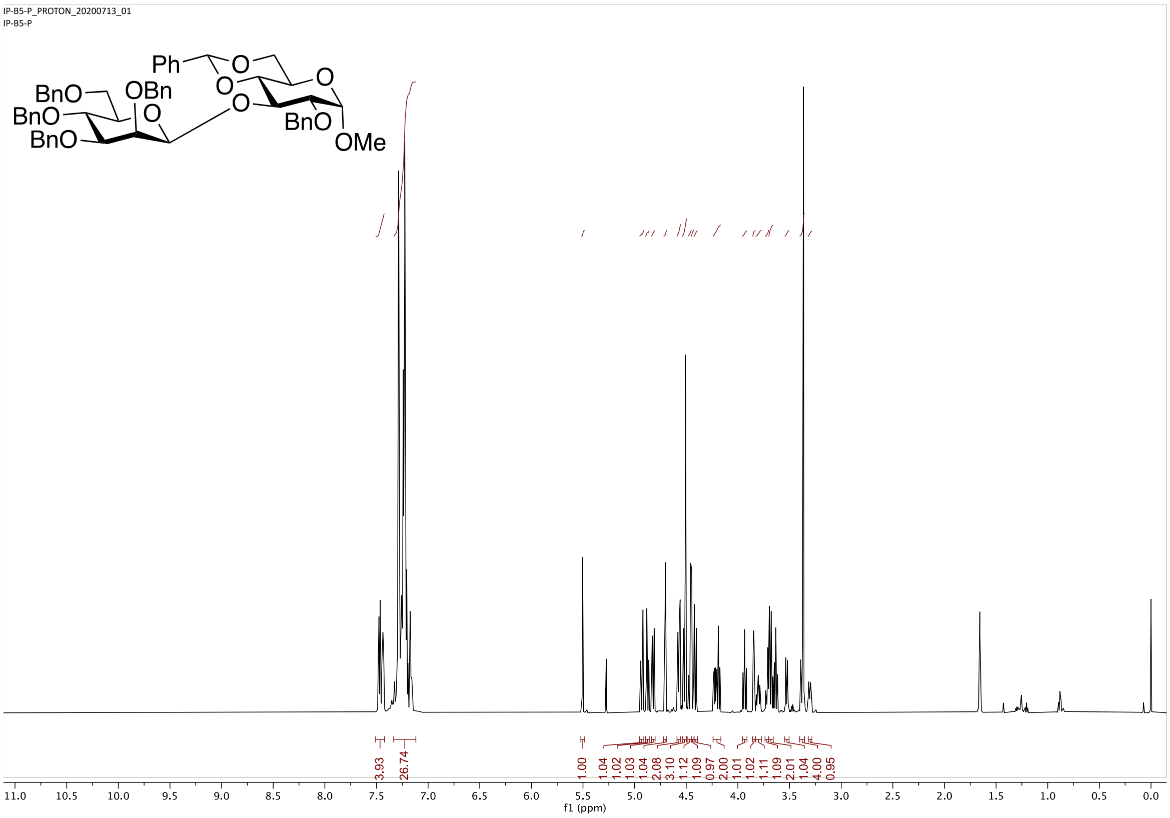

^{13}C NMR (151 MHz, Chloroform-*d*) 4d

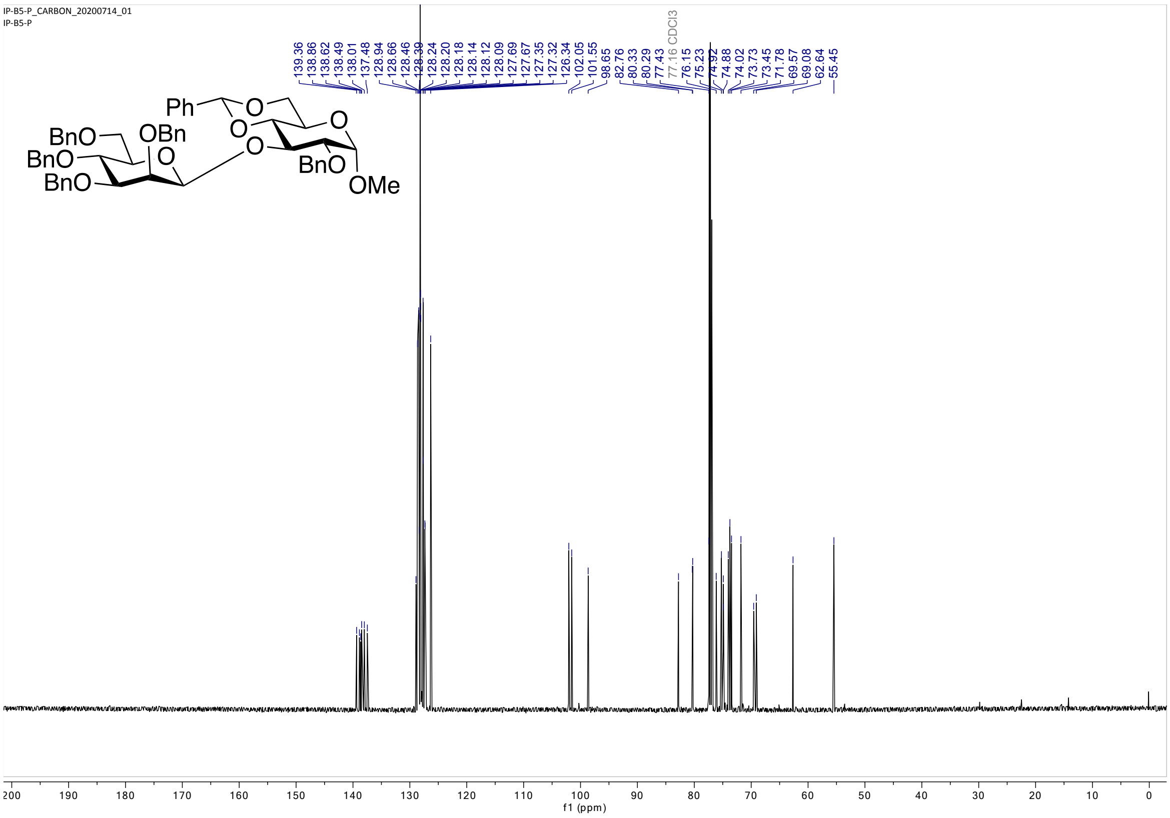

COSY NMR (600 MHz, Chloroform-*d*) 4d

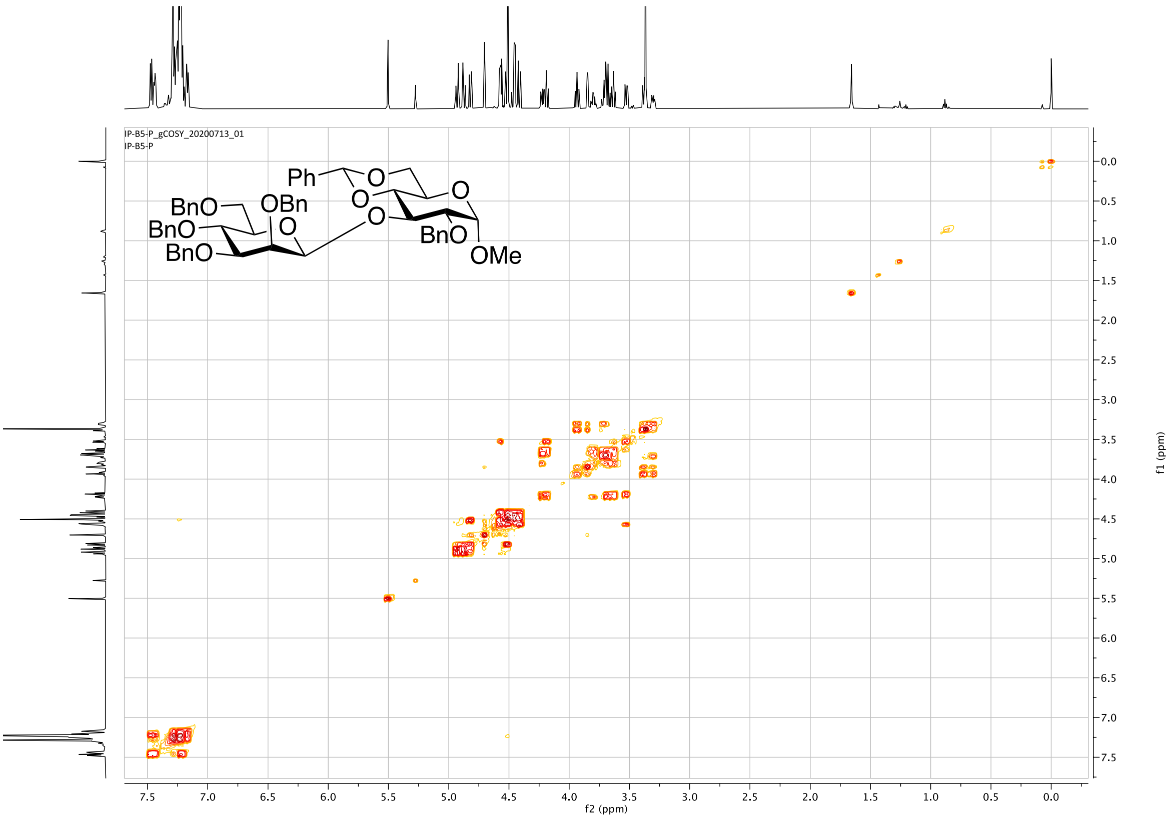

HSQC NMR (600 MHz x 151 MHz, Chloroform-*d*) 4d

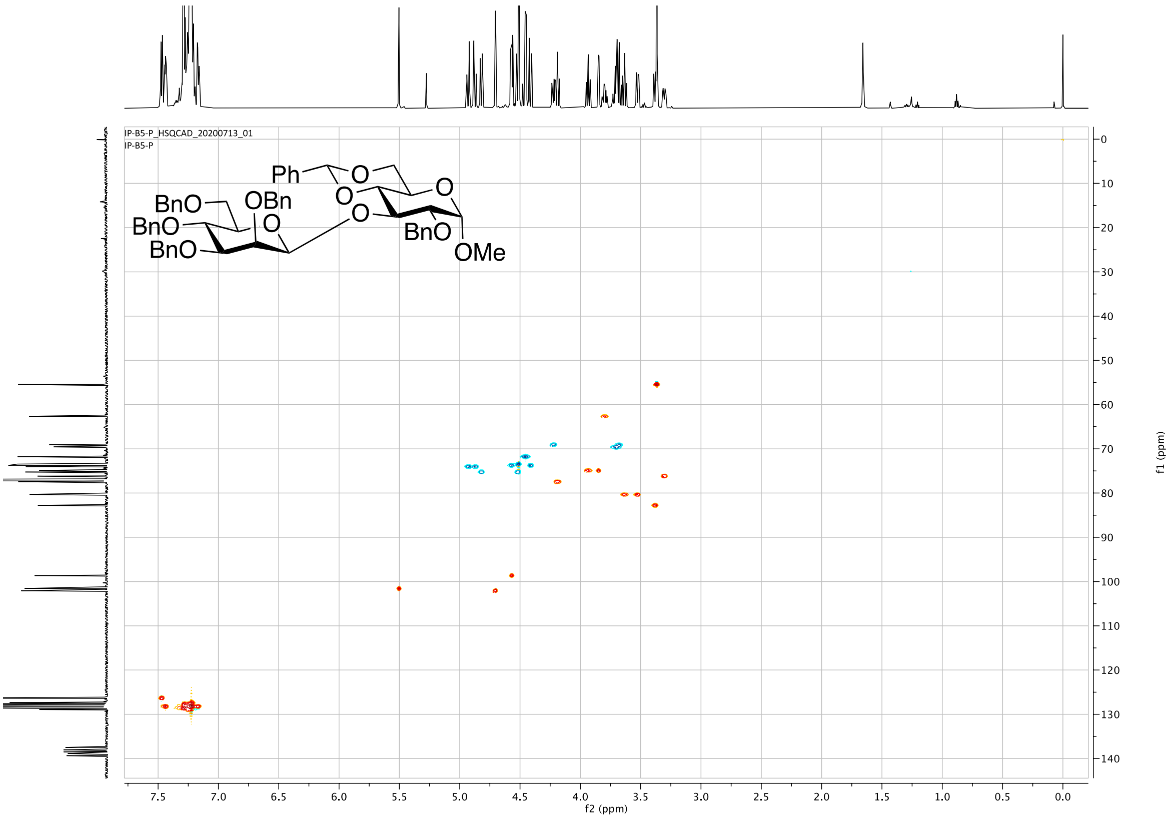

HMBC NMR (600 MHz x 151 MHz, Chloroform-*d*) 4d

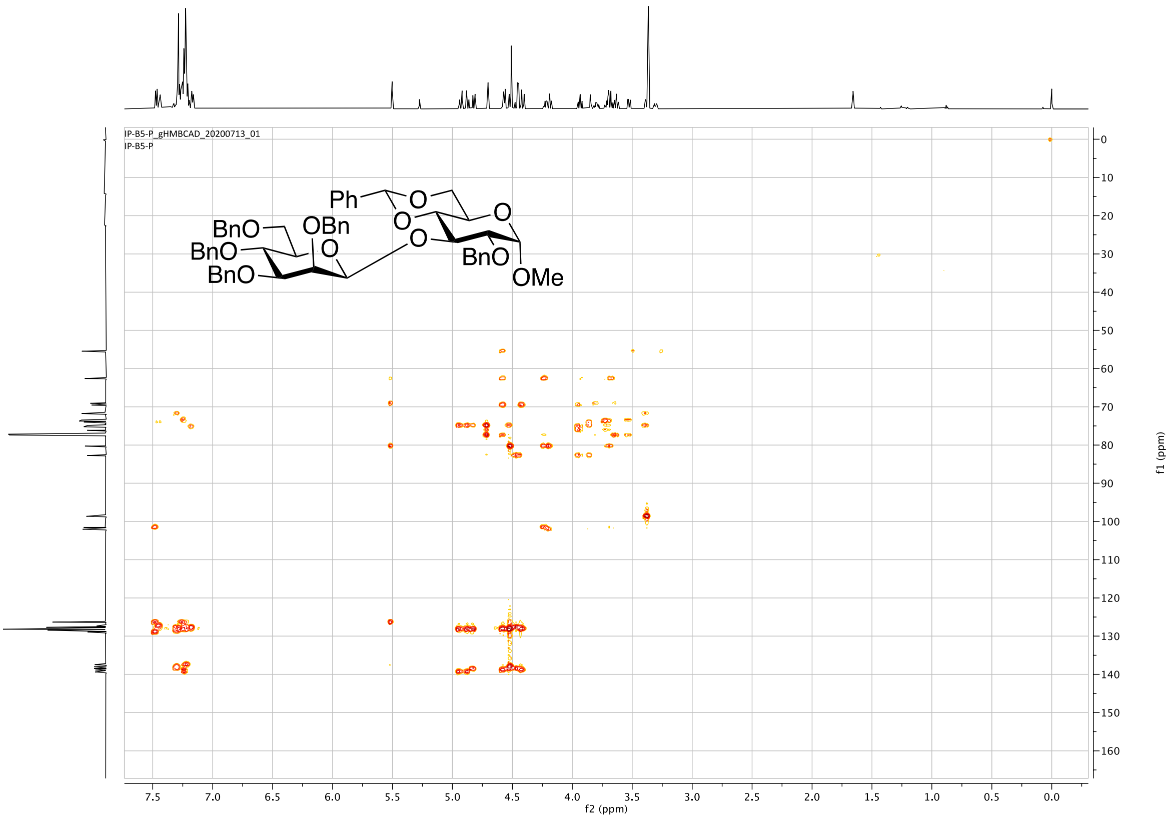

^{1}H NMR (500 MHz, Chloroform-*d*) 4e

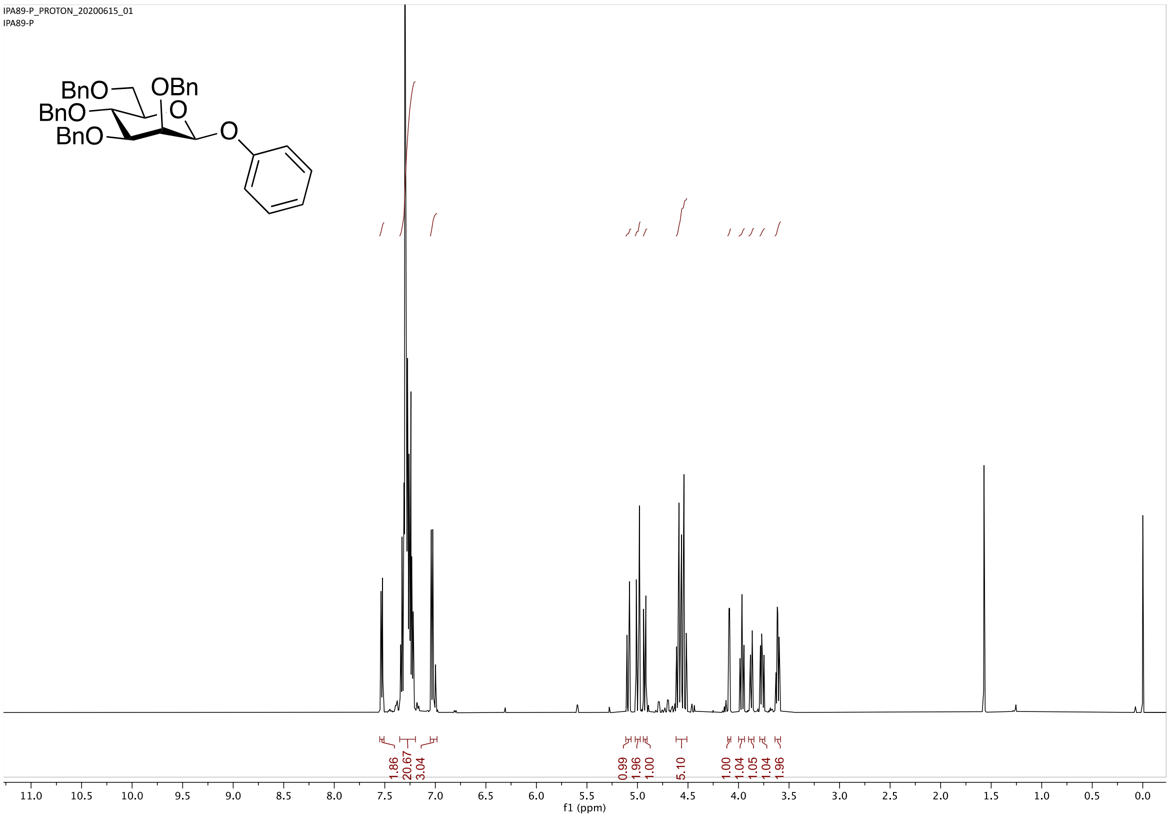

^{13}C NMR (126 MHz, Chloroform-*d*) 4e

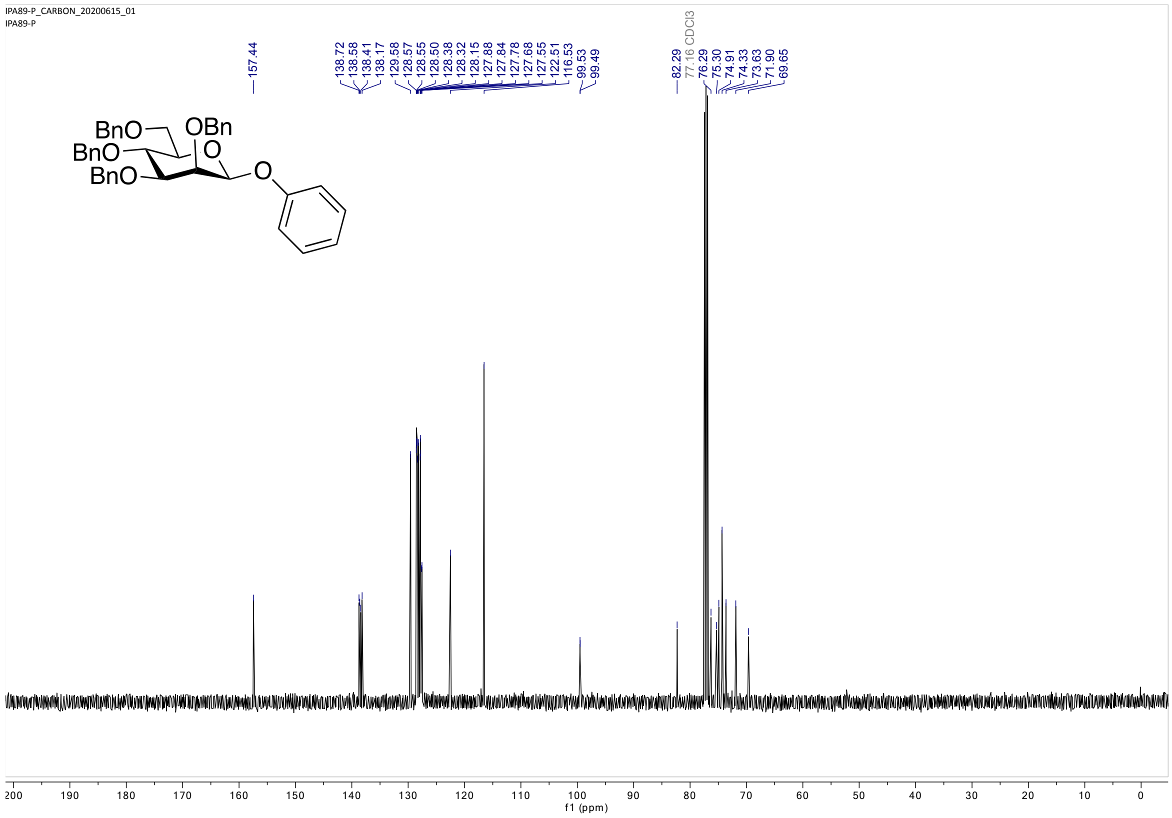

COSY NMR (500 MHz, Chloroform-*d*) 4e

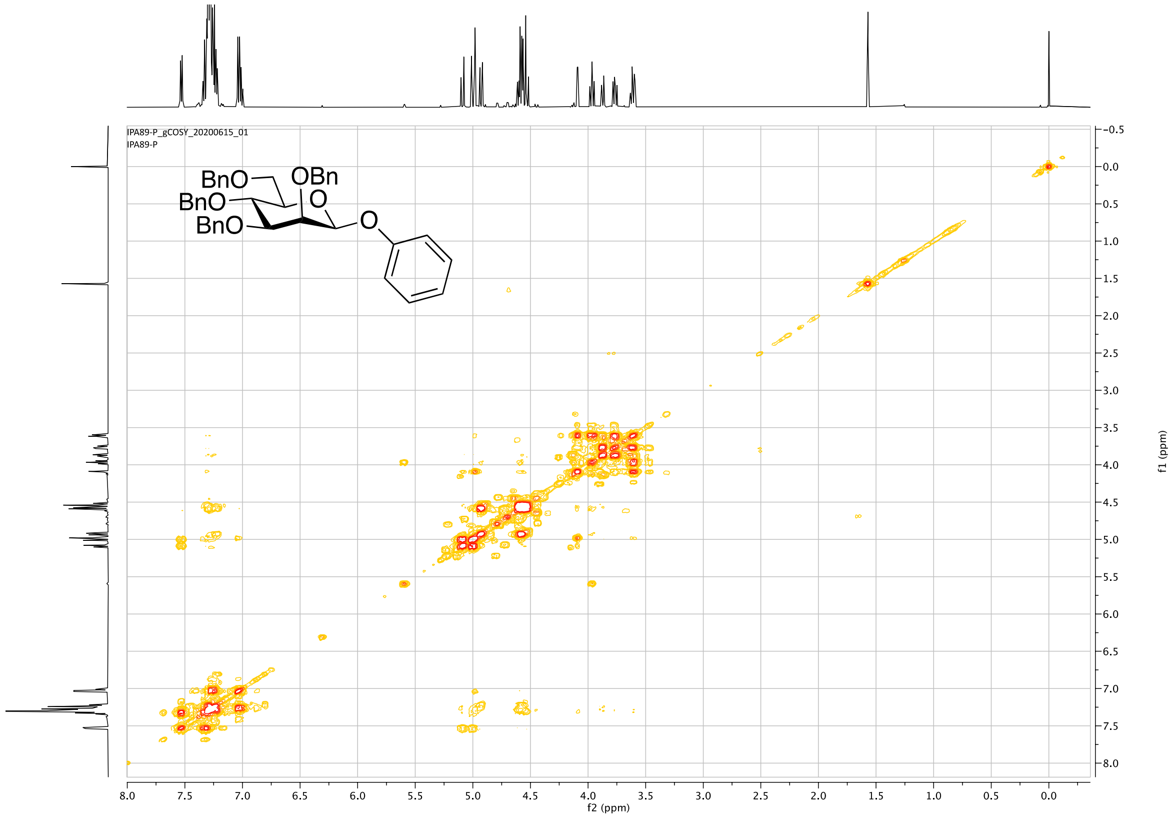

HSQC NMR (500 MHz x 126 MHz, Chloroform-*d*) 4e

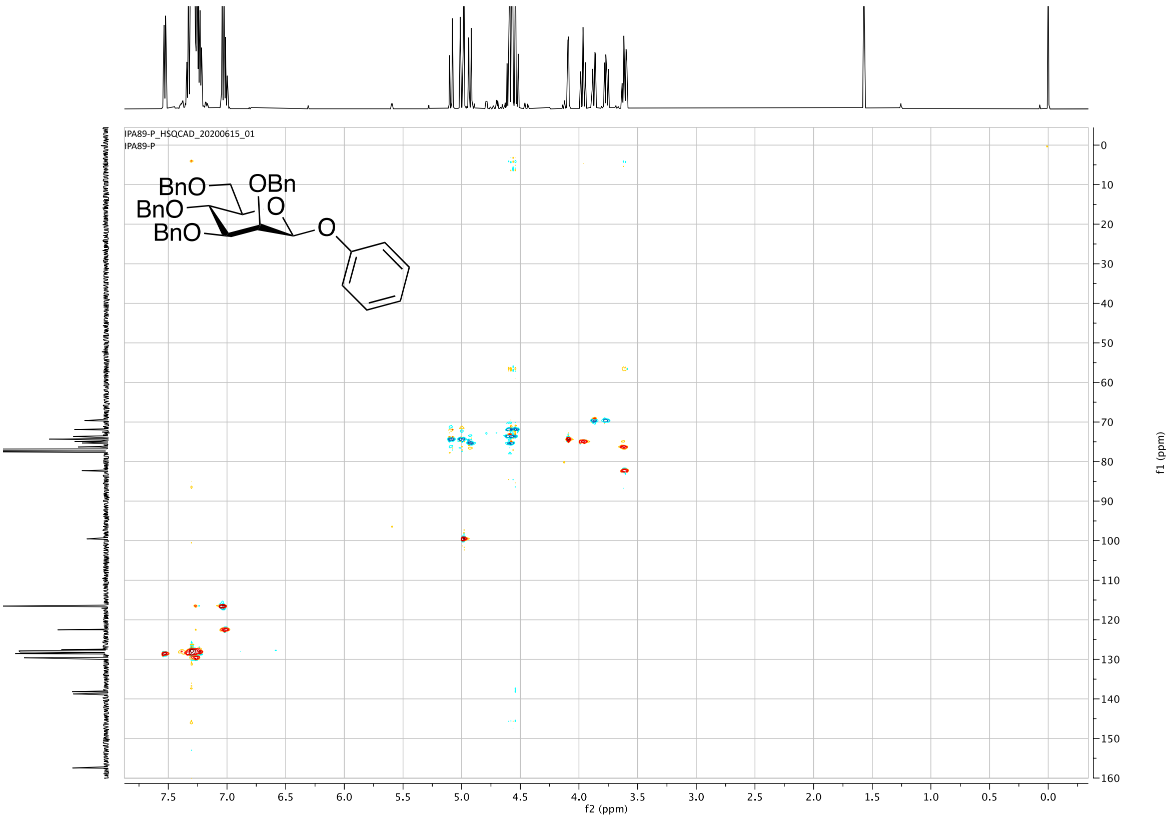

HMBC NMR (500 MHz x 126 MHz, Chloroform-*d*) 4e

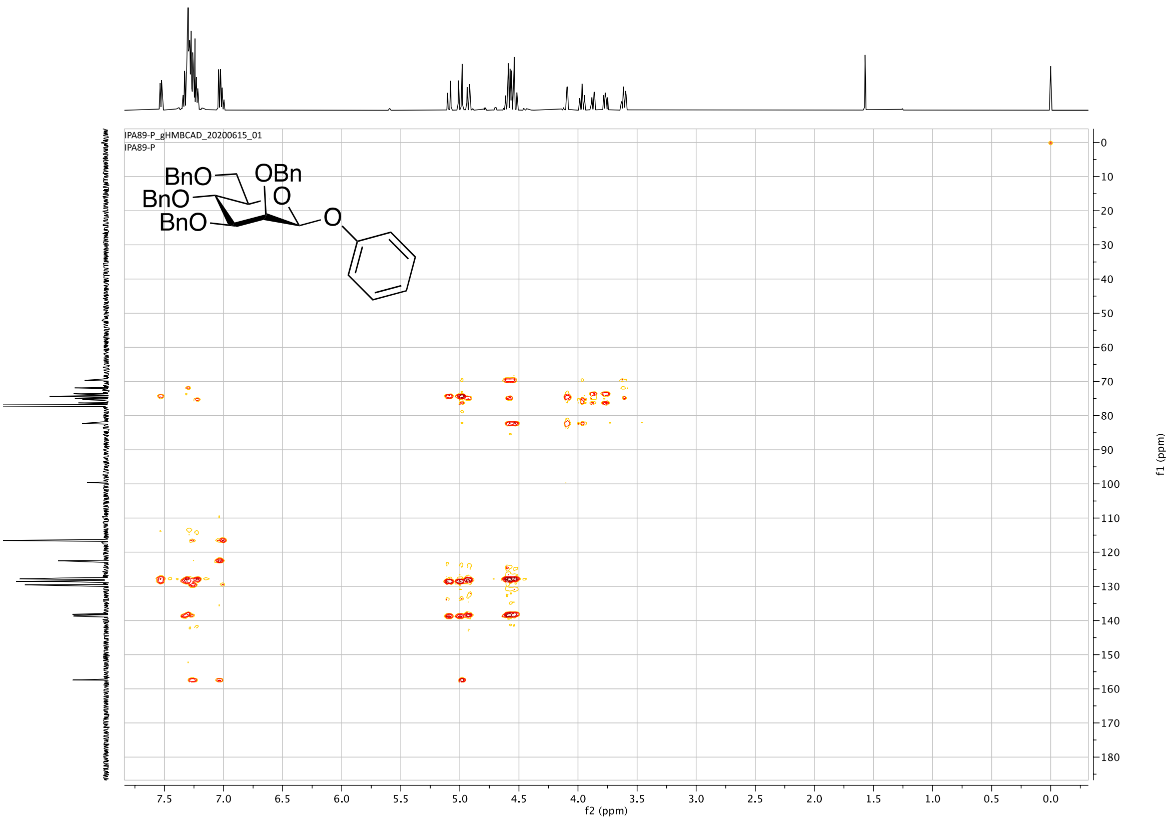

^{1}H NMR (500 MHz, Chloroform-*d*) 4f

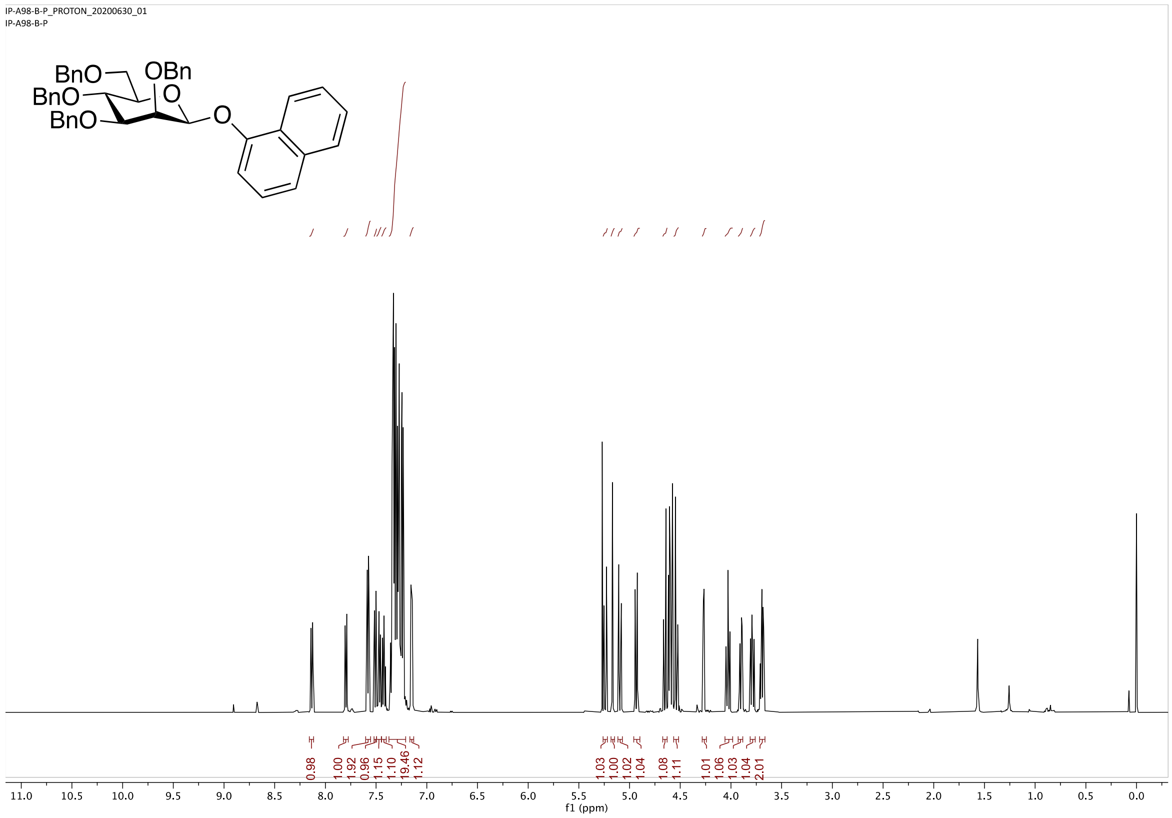

^{13}C NMR (126 MHz, Chloroform-*d*) 4f

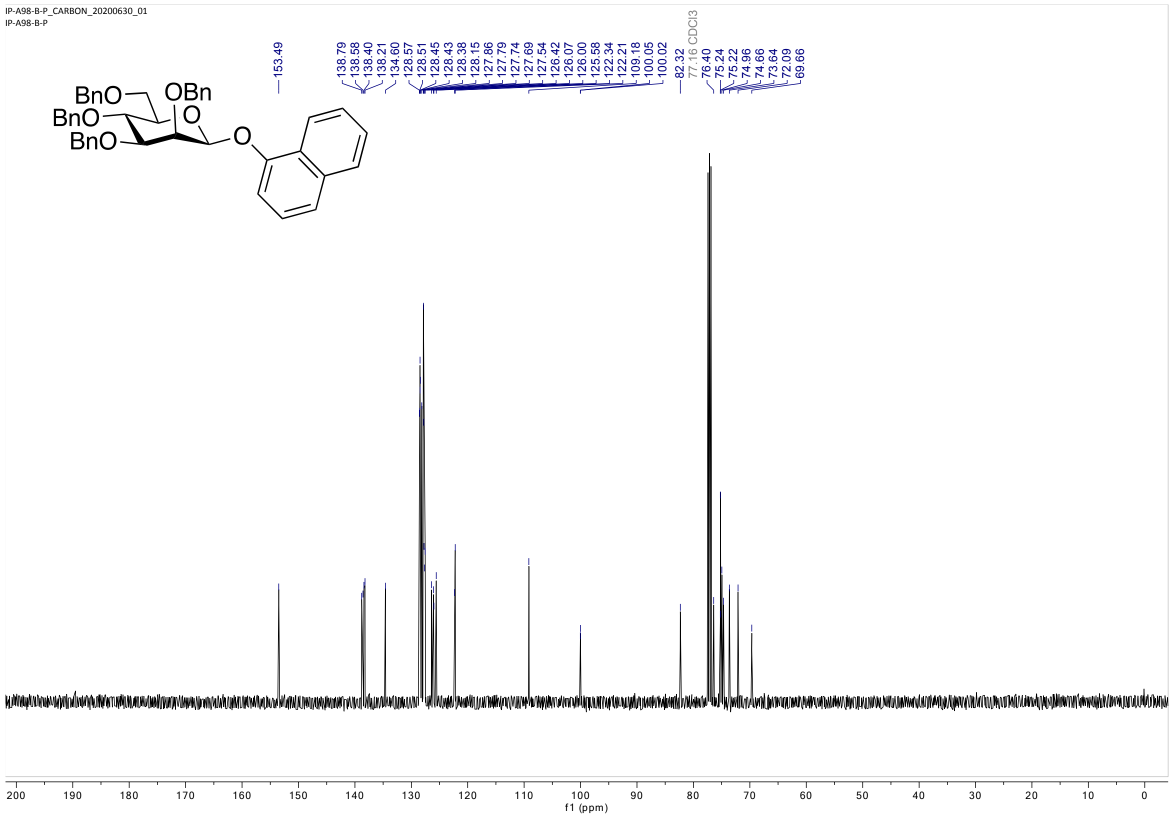

COSY NMR (500 MHz, Chloroform-*d*) 4f

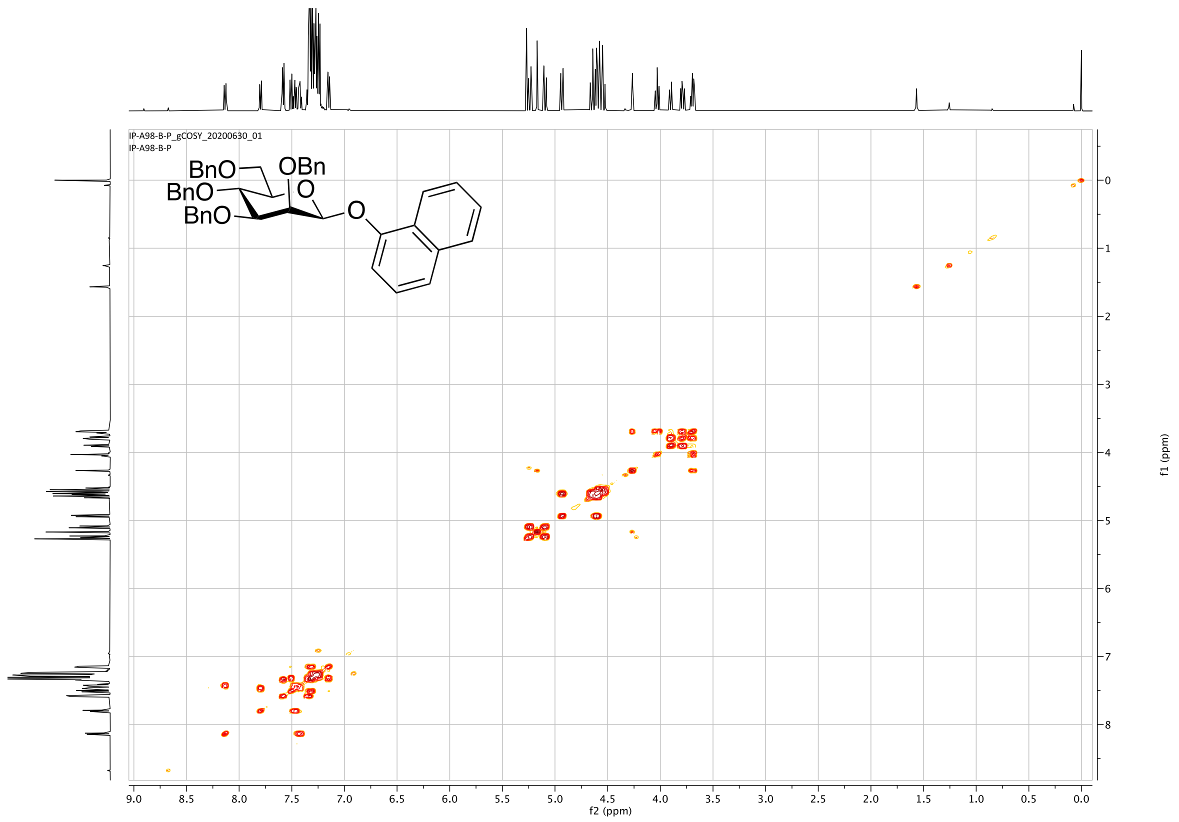

HSQC NMR (500 MHz x 126 MHz, Chloroform-*d*) 4f

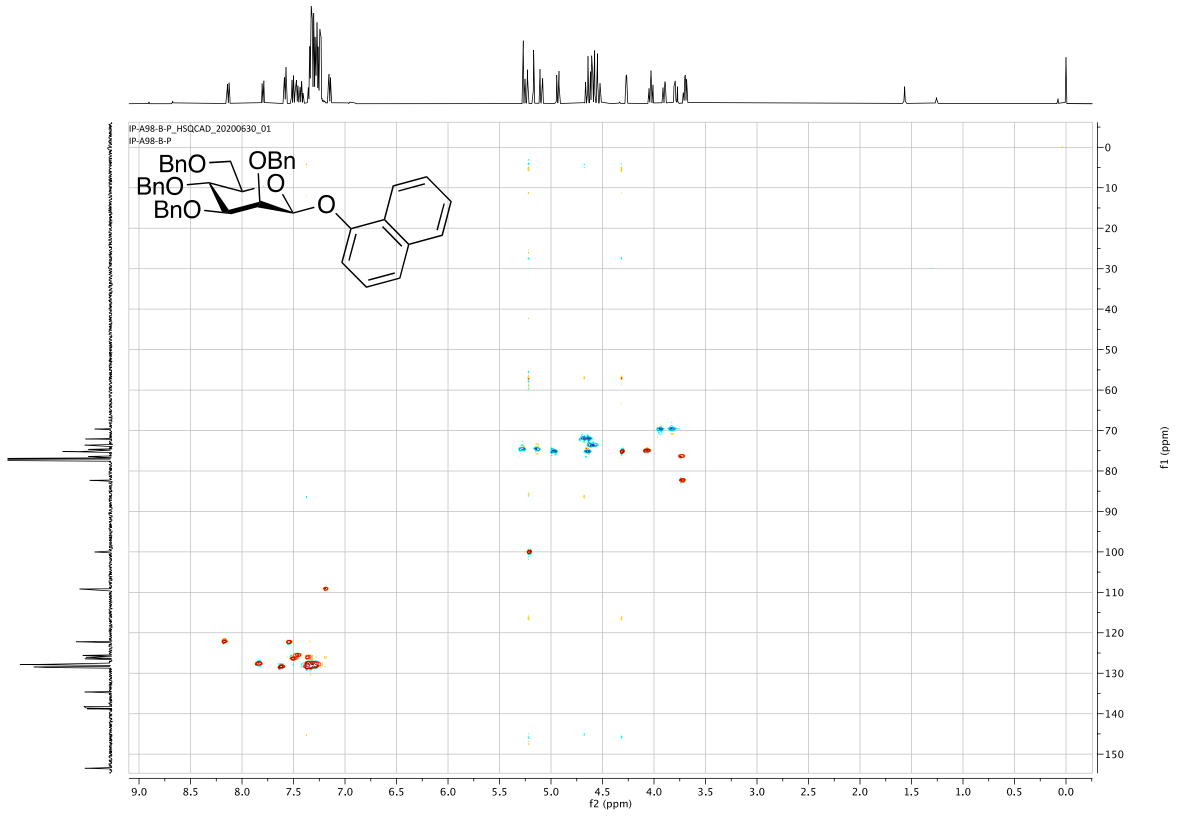

^{13}C- ^{1}H coupled HSQC NMR (500 MHz x 126 MHz, Chloroform-*d*) 4f

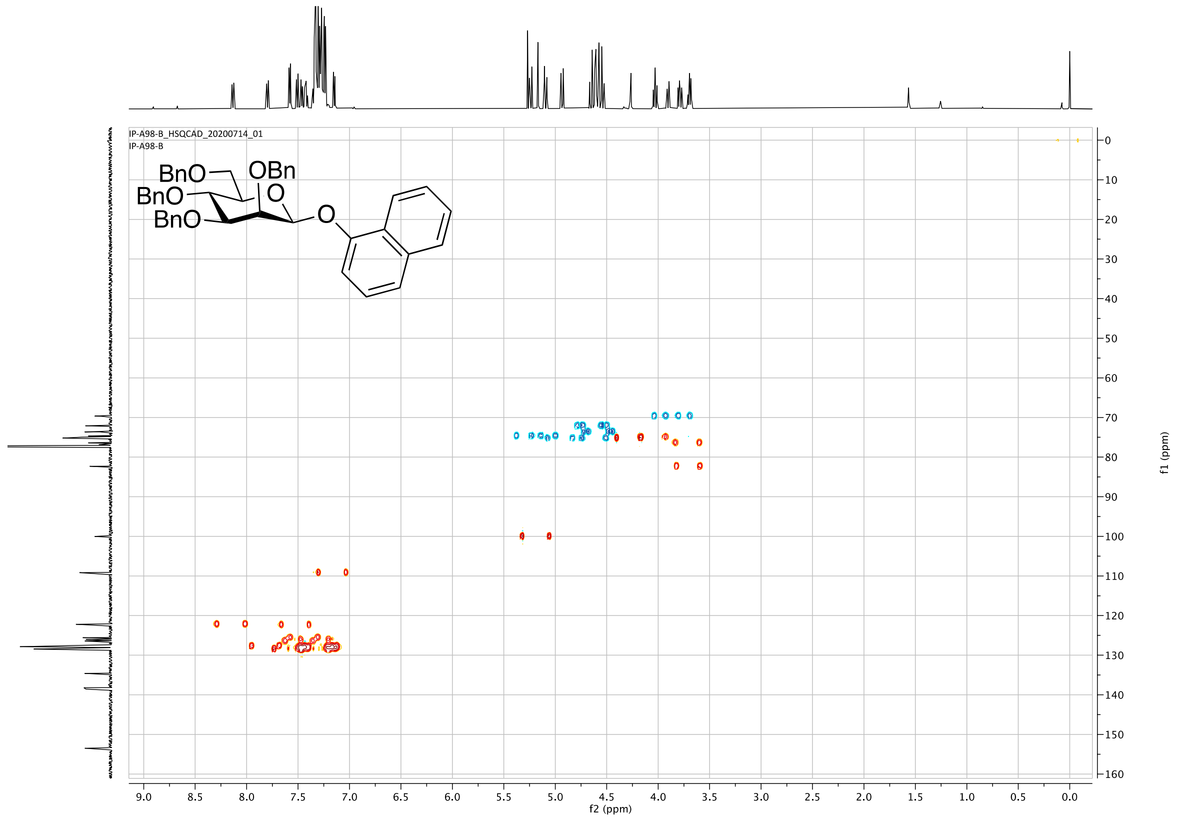

HMBC NMR (500 MHz x 126 MHz, Chloroform-*d*) 4f

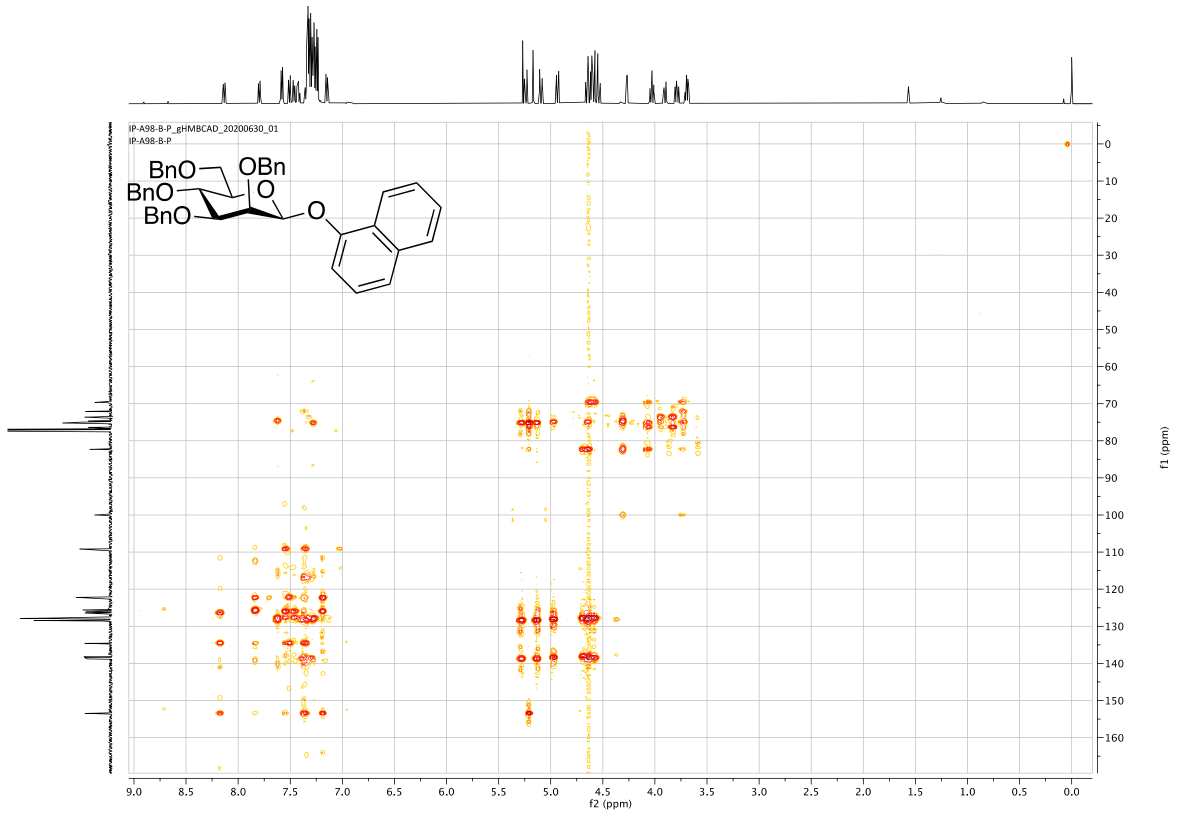

^{1}H NMR (500 MHz, Chloroform-*d*) 4g

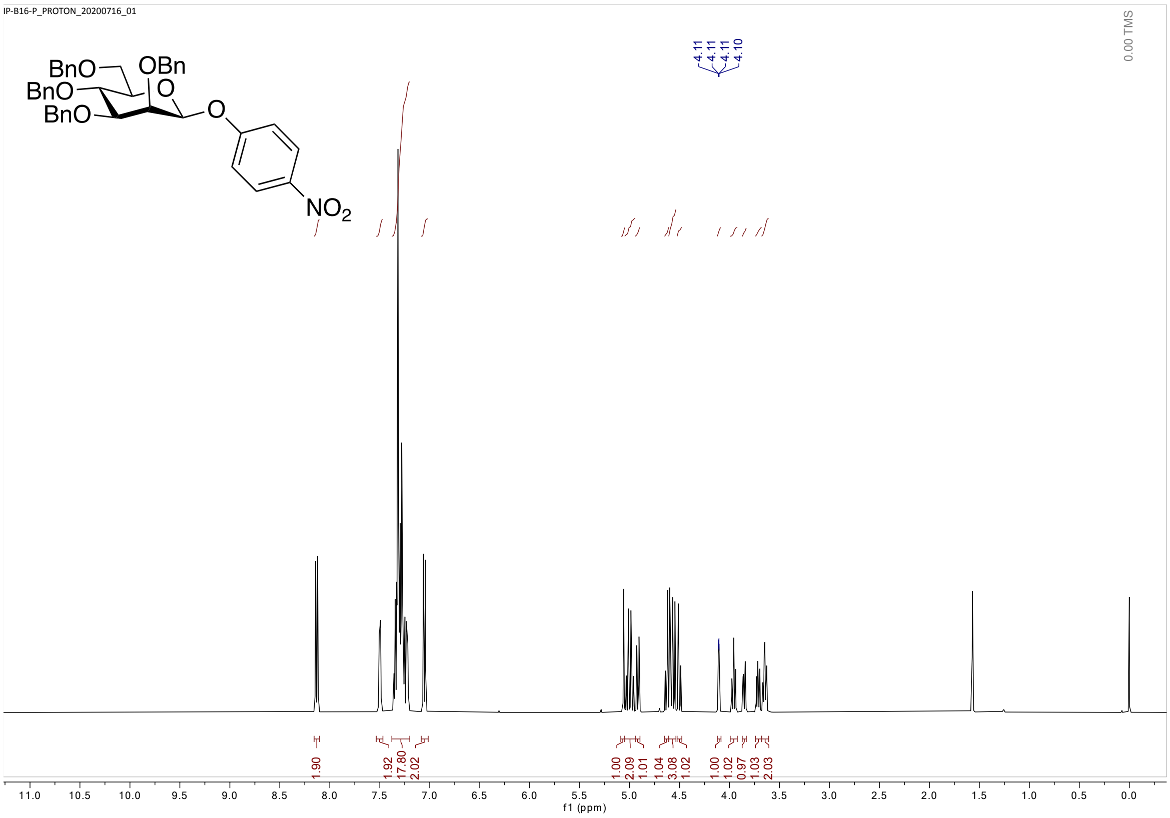

^{13}C NMR (126 MHz, Chloroform-*d*) 4g

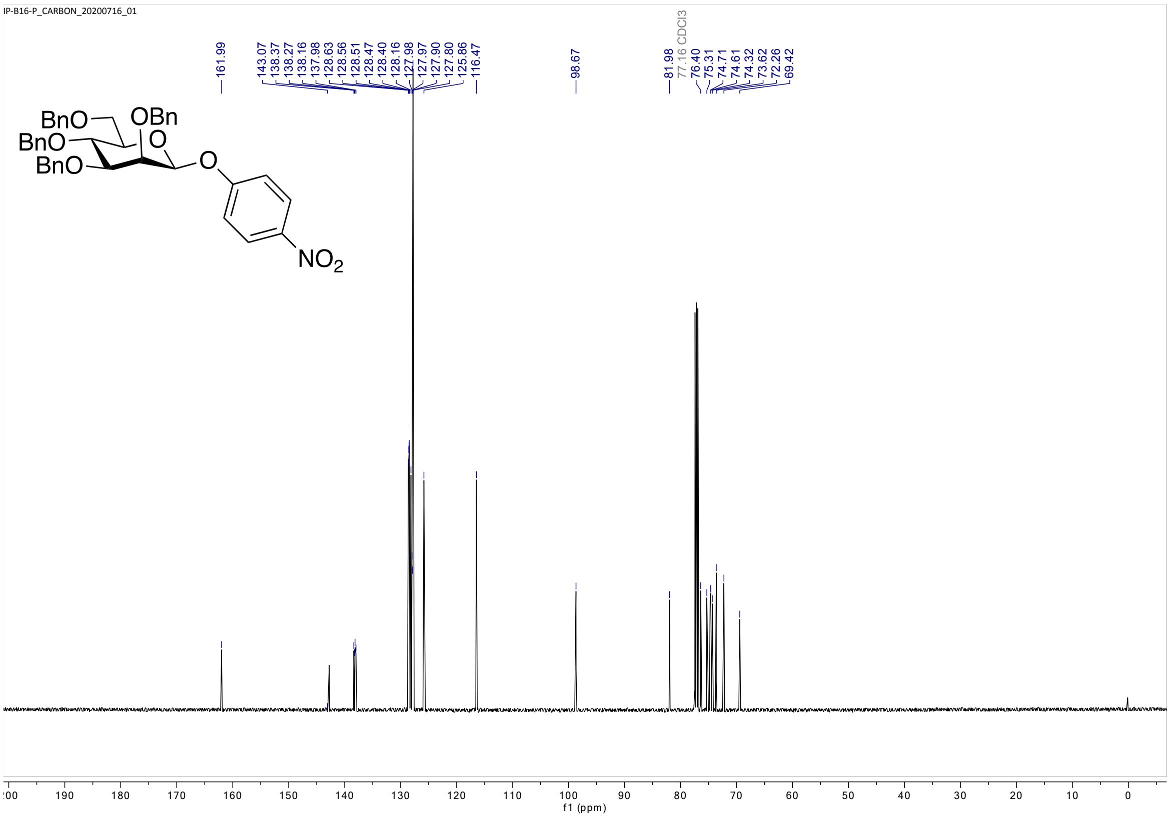

COSY NMR (500 MHz, Chloroform-*d*) 4g

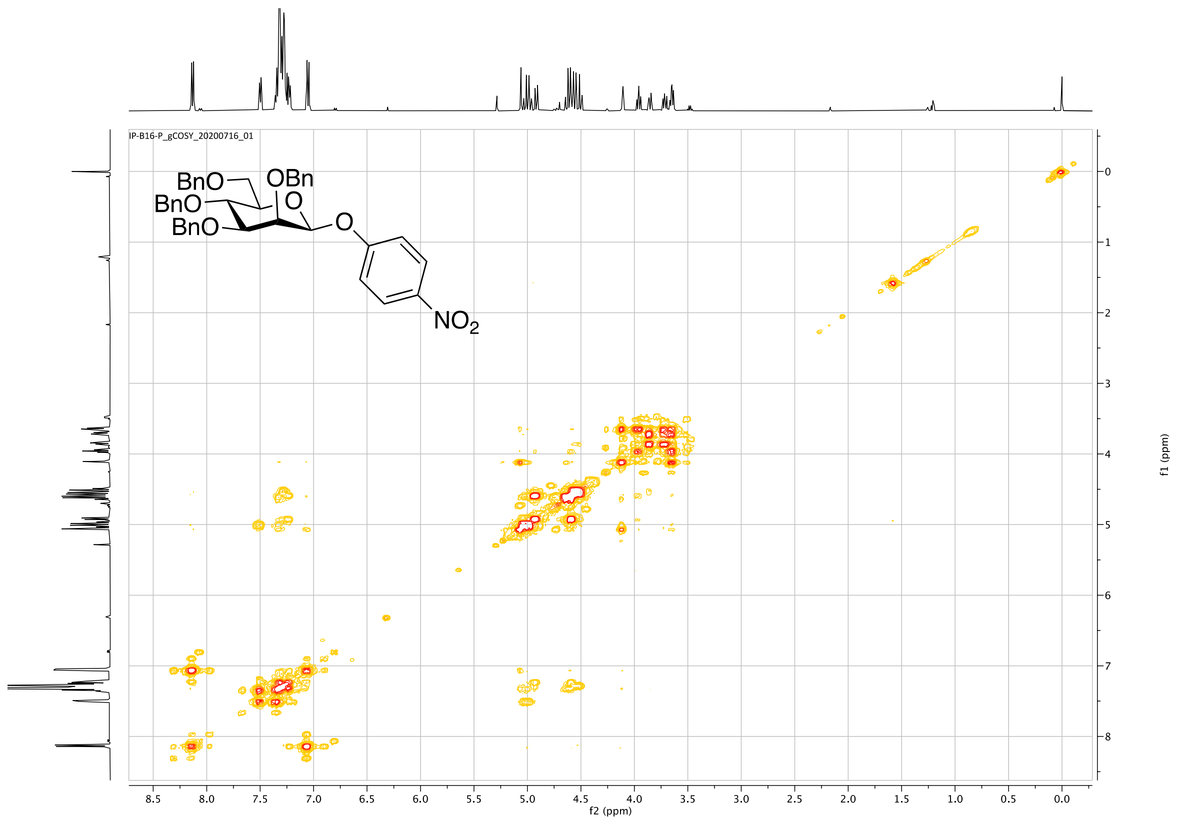

HSQC NMR (500 MHz x 126 MHz, Chloroform-*d*) 4g

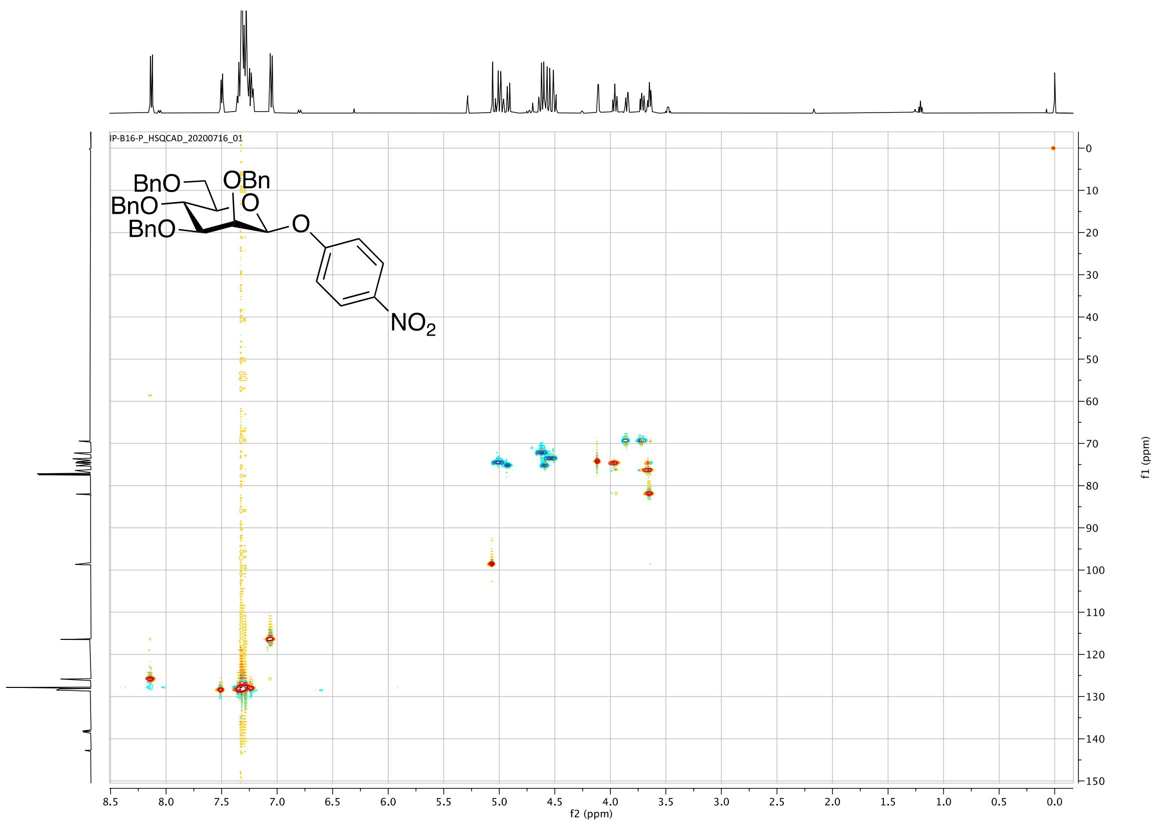

^{13}C- ^{1}H coupled HSQC NMR (500 MHz x 126 MHz, Chloroform-*d*) 4g

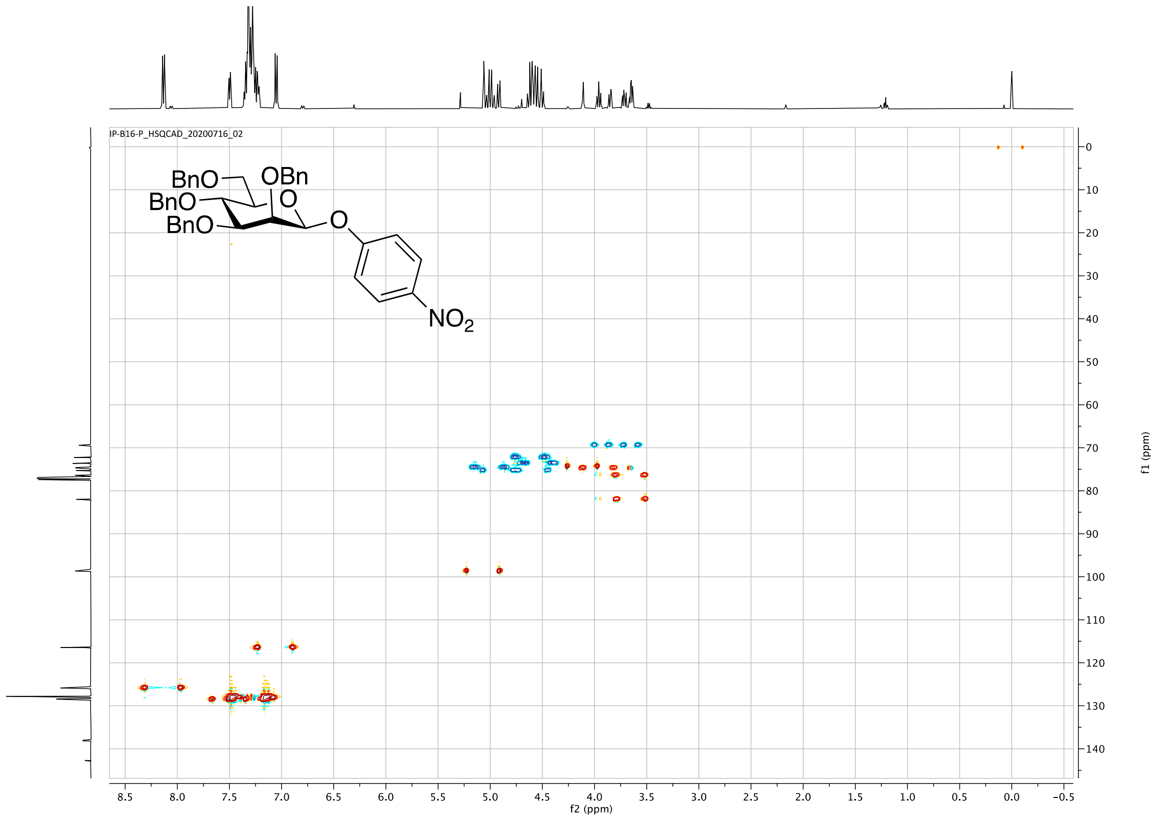

HMBC NMR (500 MHz x 126 MHz, Chloroform-*d*) 4g

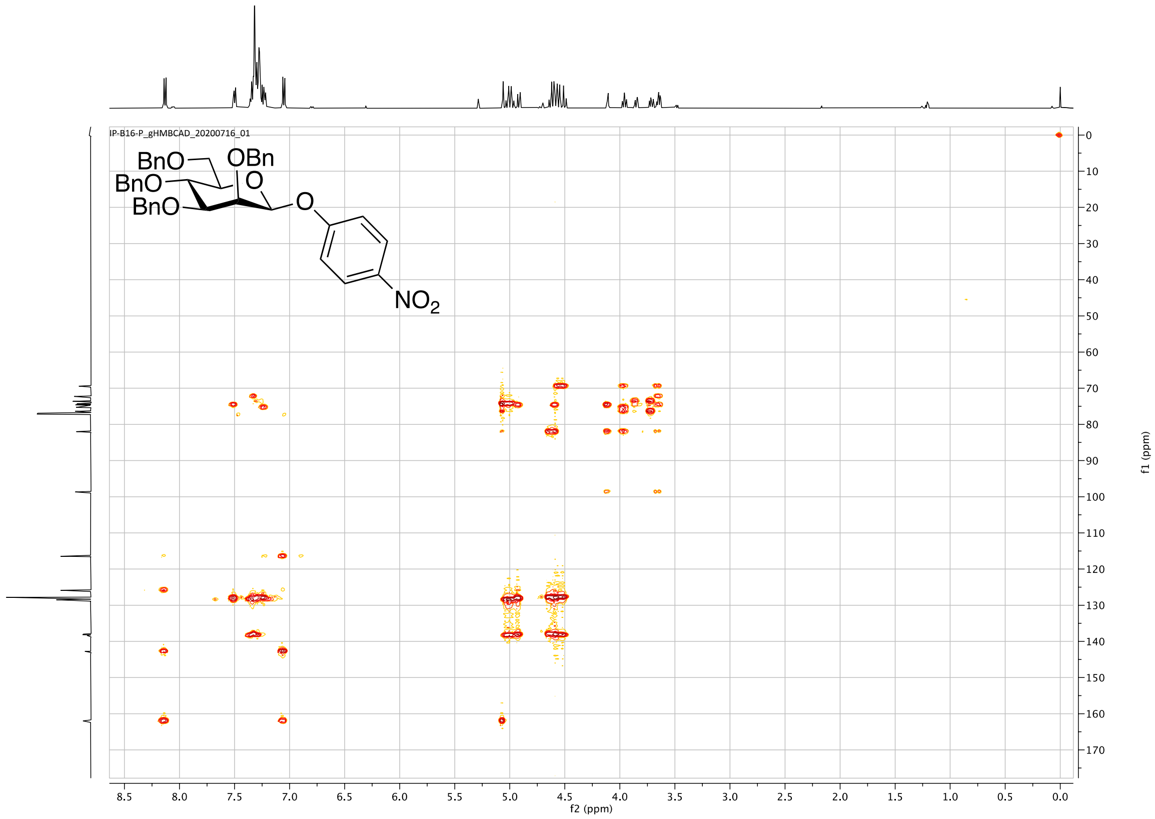

^{1}H NMR (600 MHz, Chloroform-*d*) 4h

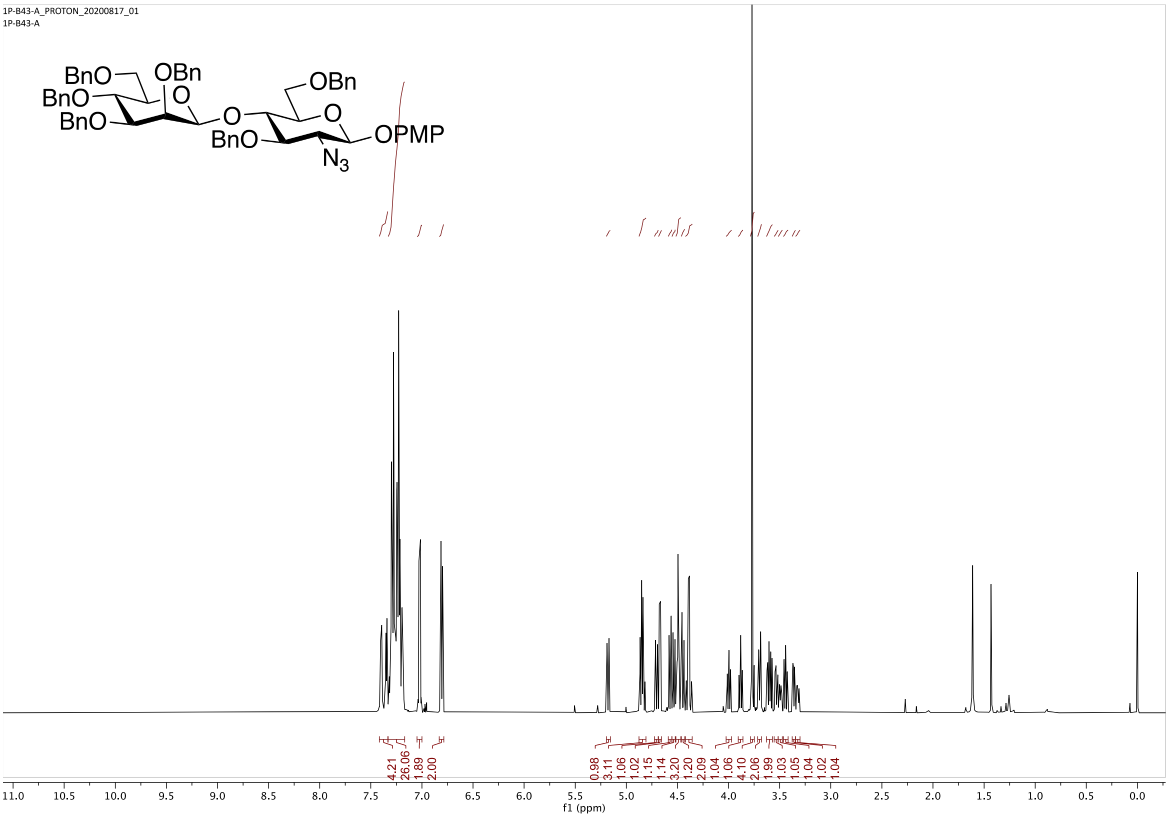

^{13}C NMR (151 MHz, Chloroform-*d*) 4h

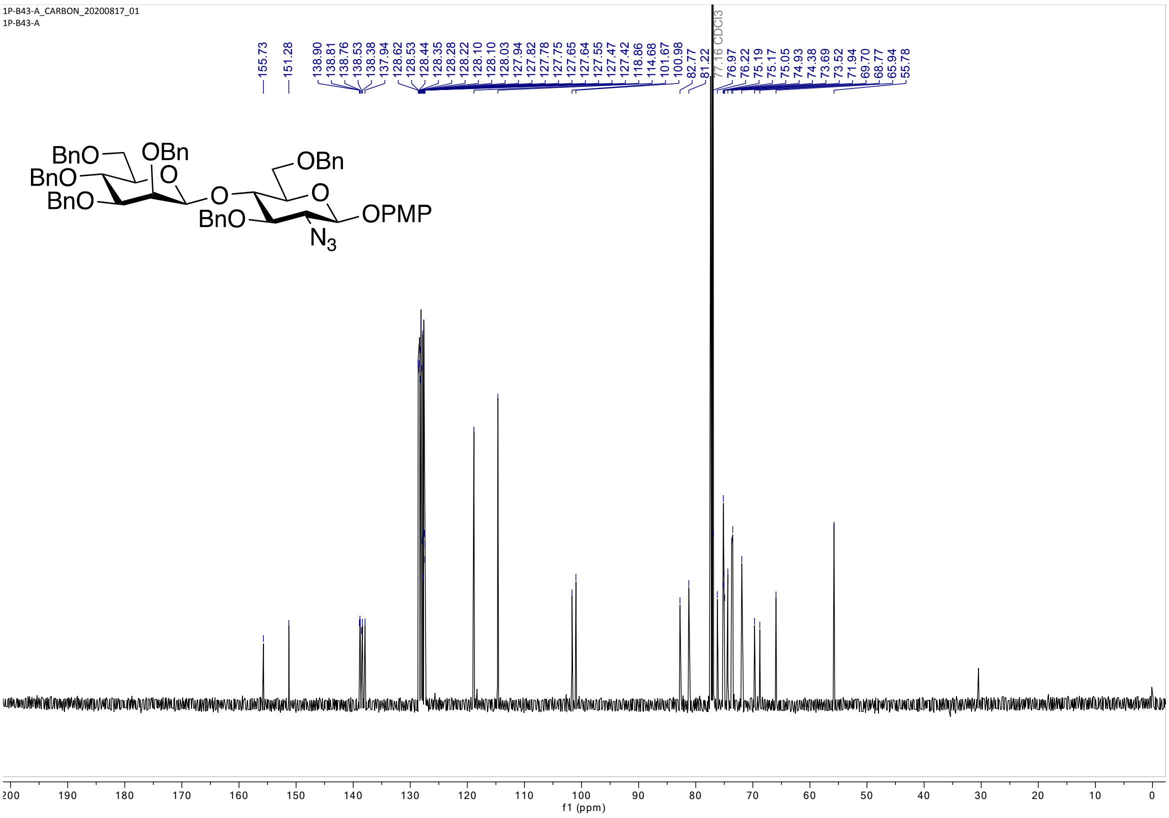

COSY NMR (600 MHz, Chloroform-*d*) 4h

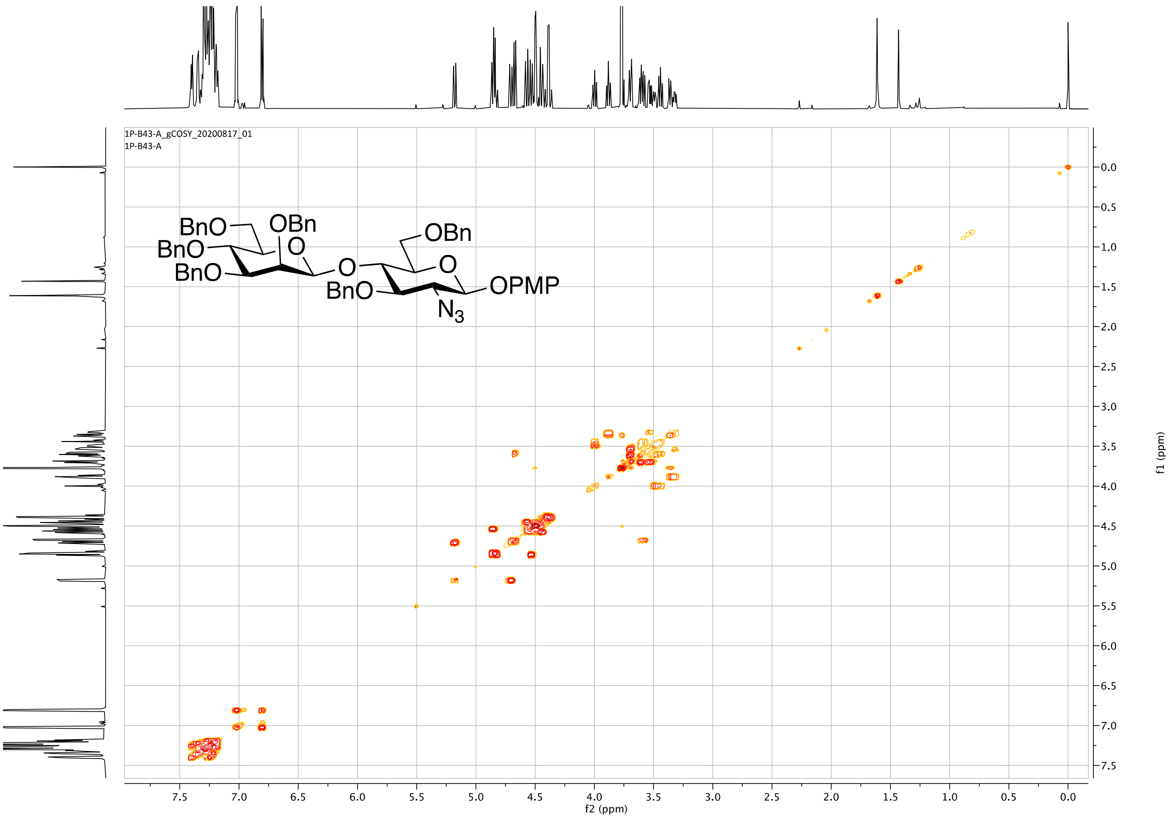

HSQC NMR (600 MHz x 151 MHz, Chloroform-*d*) 4h

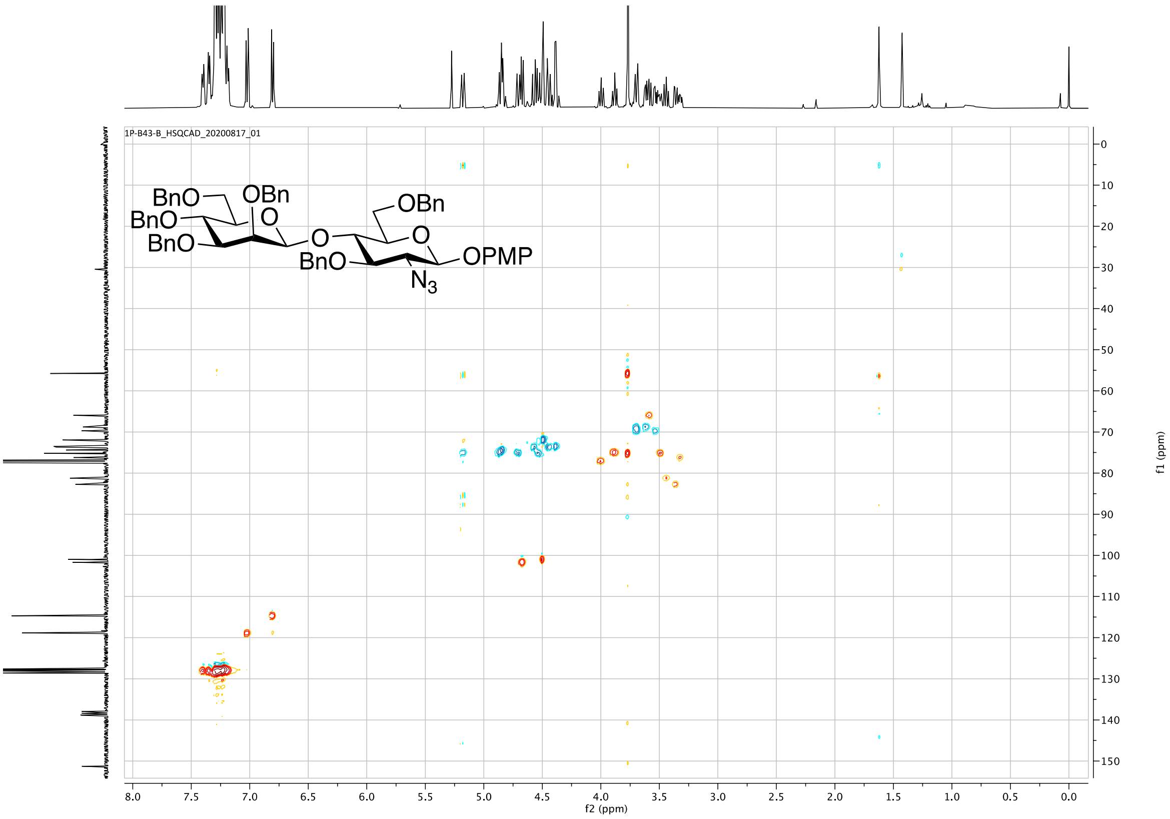

^{13}C- ^{1}H coupled HSQC NMR (600 MHz x 151 MHz, Chloroform-*d*) 4h

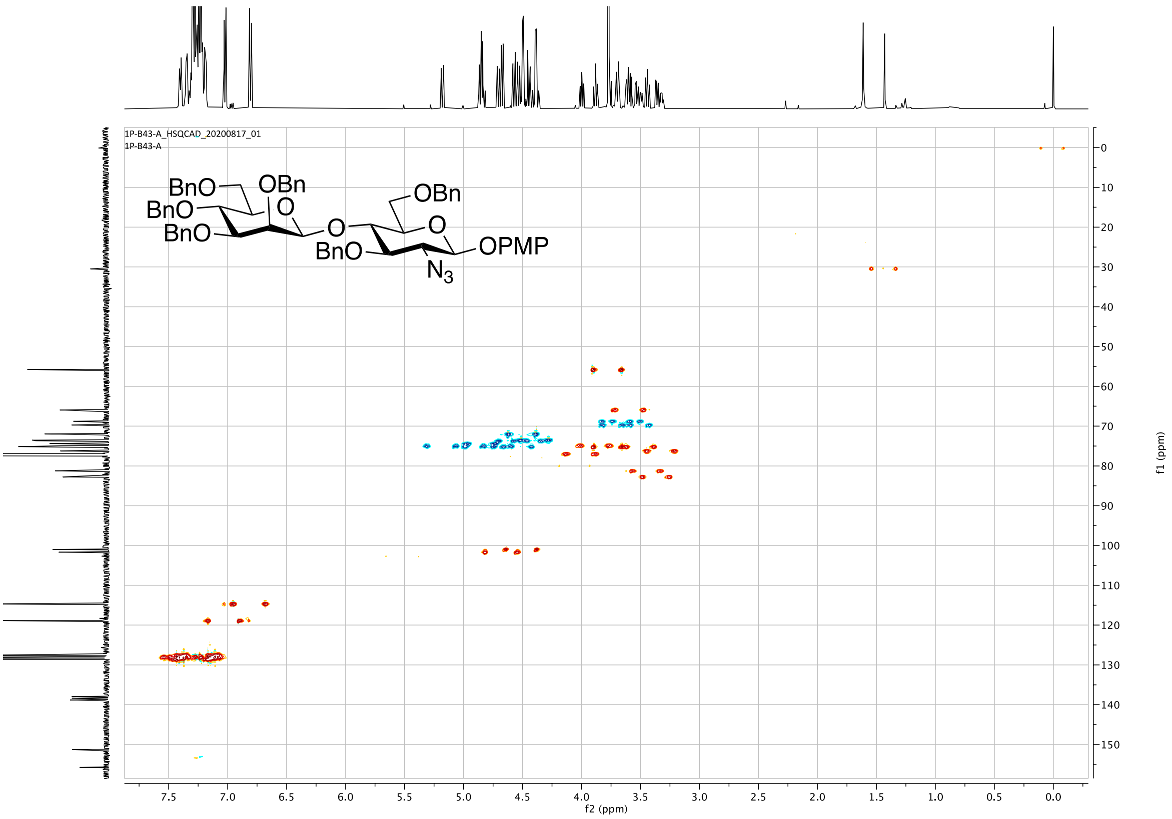

HMBC NMR (600 MHz x 151 MHz, Chloroform-*d*) 4h

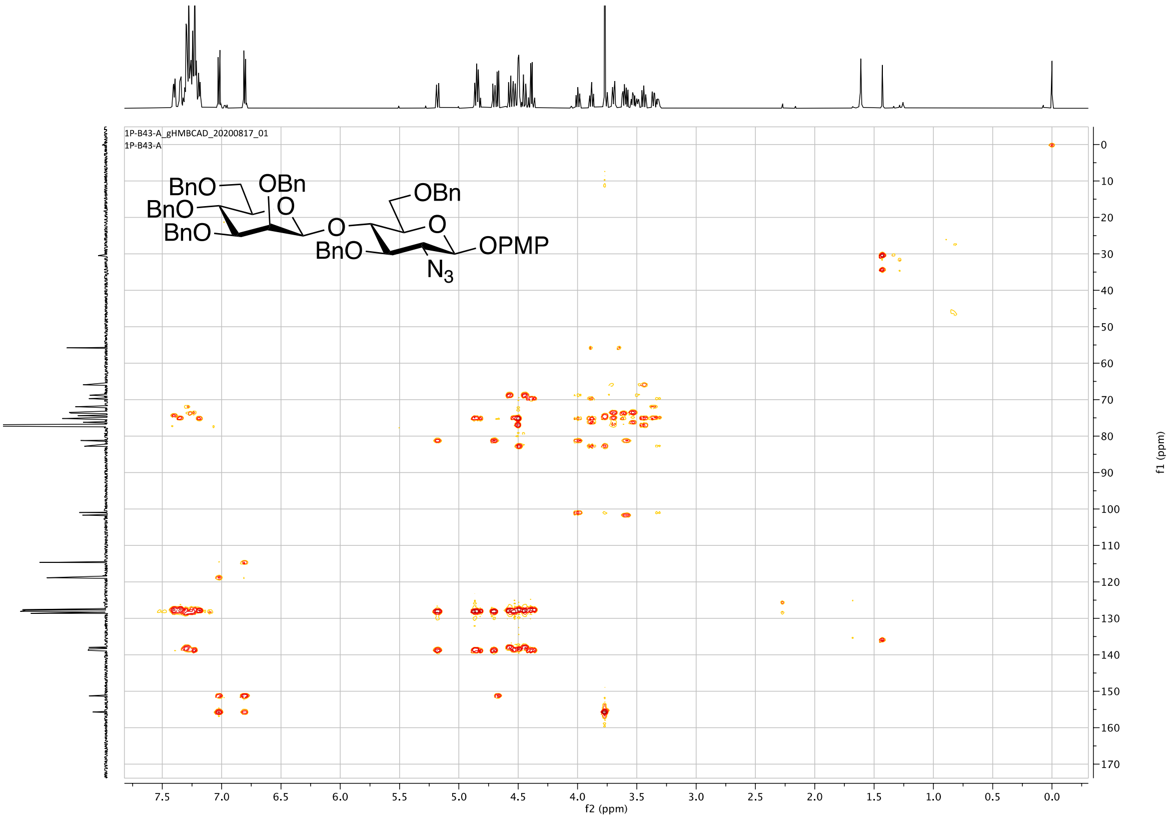

^{1}H NMR (500 MHz, Chloroform-*d*) 5b

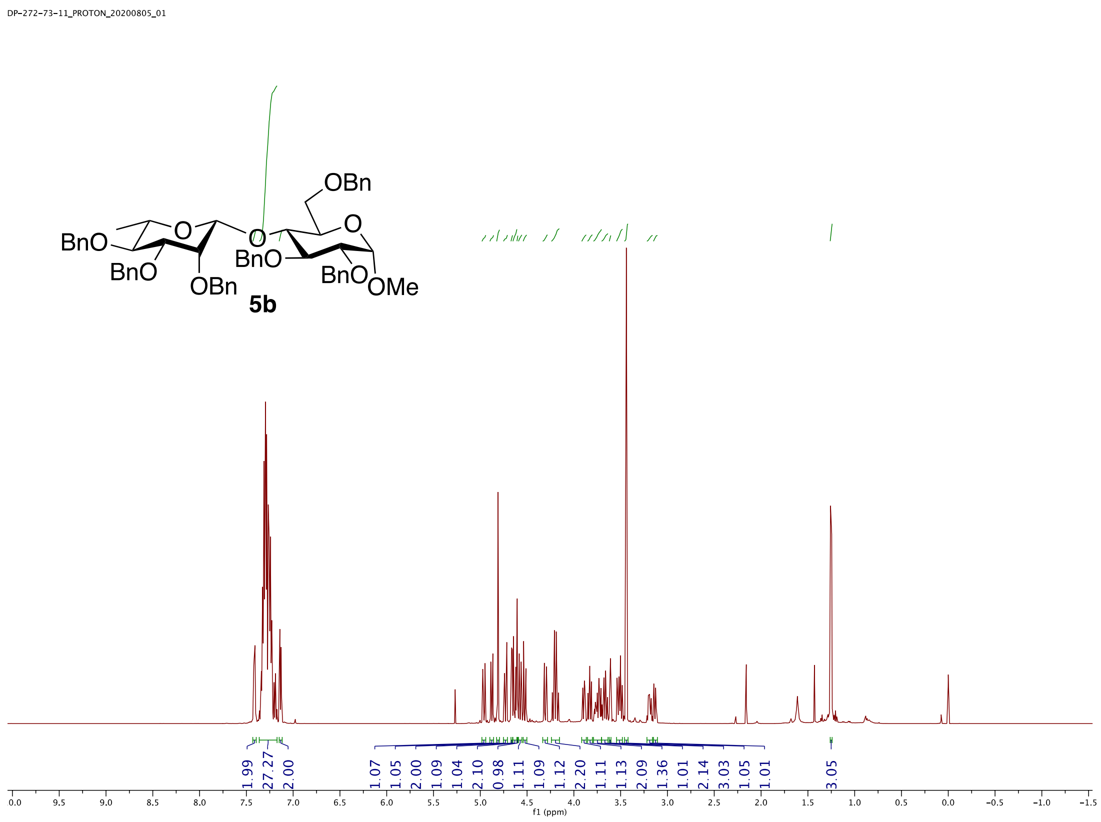

^{13}C NMR (126 MHz, Chloroform-*d*) 5b

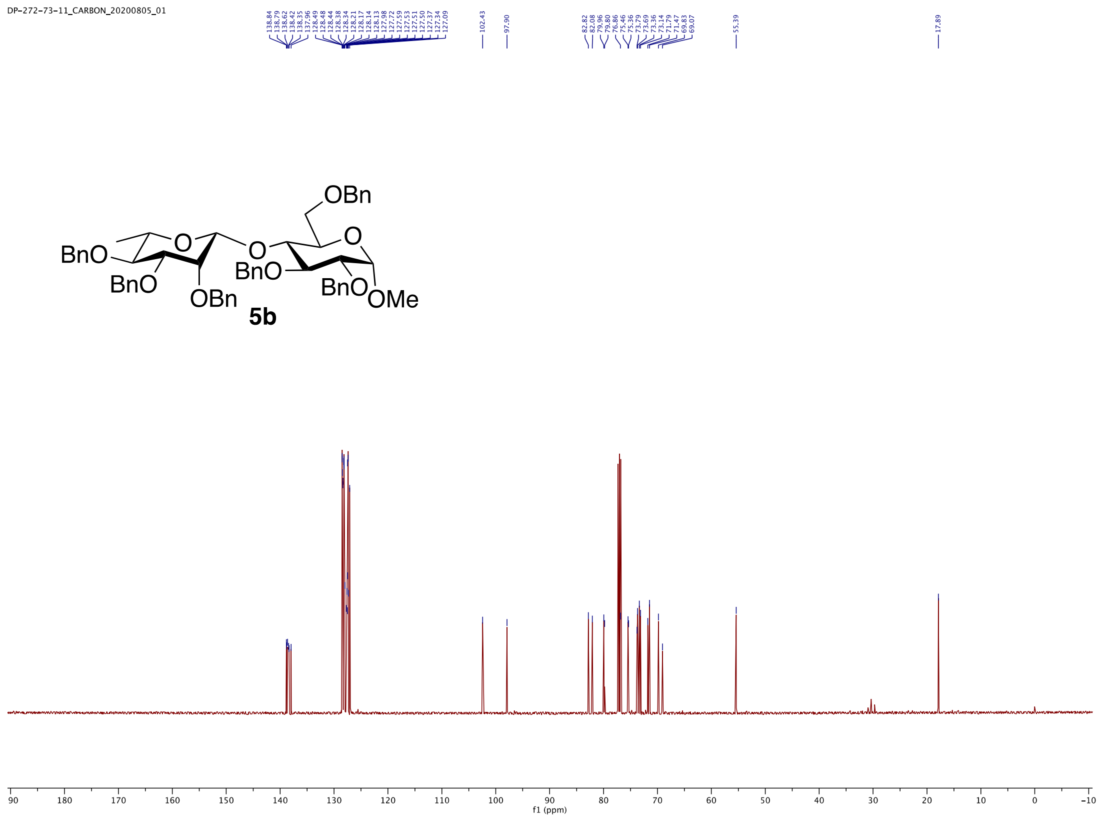

^{13}C - ^{1}H decoupled HSQC (500 x 126 MHz, Chloroform-*d*) 5b

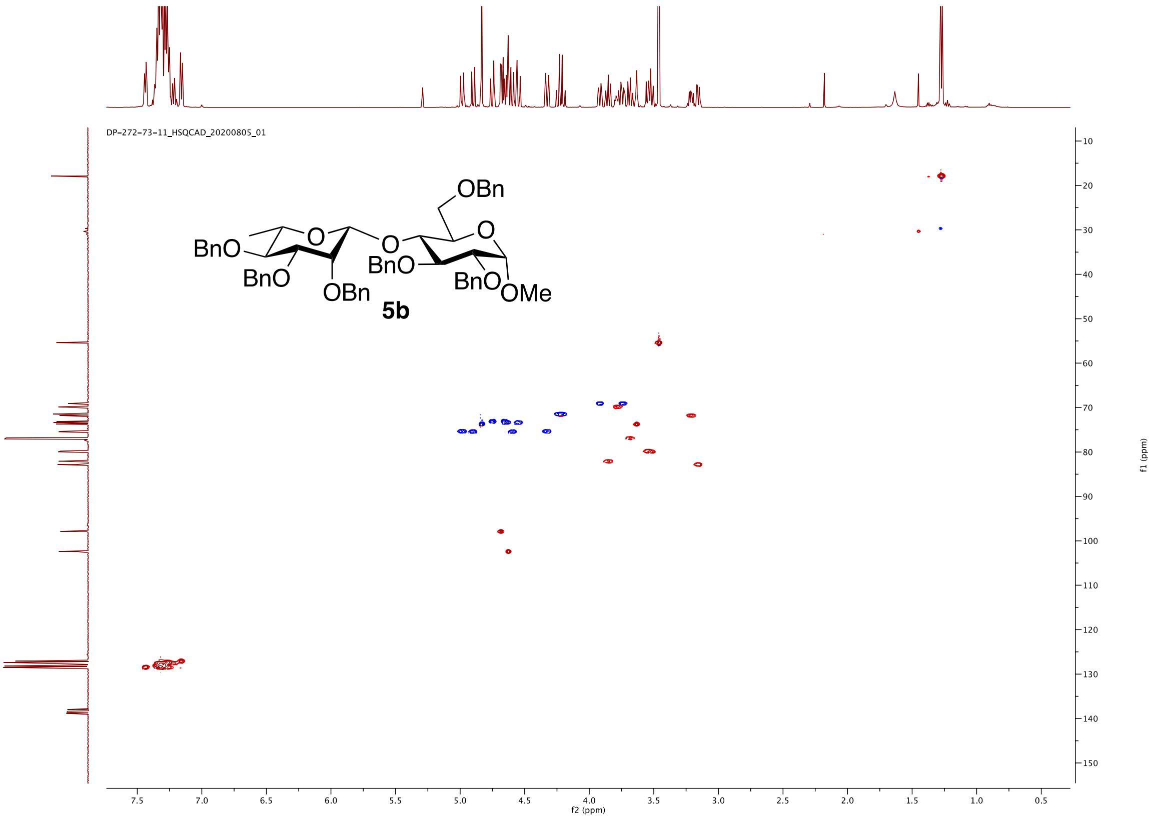

^{13}C - ^{1}H coupled HSQC (500 x 126 MHz, Chloroform-*d*) 5b

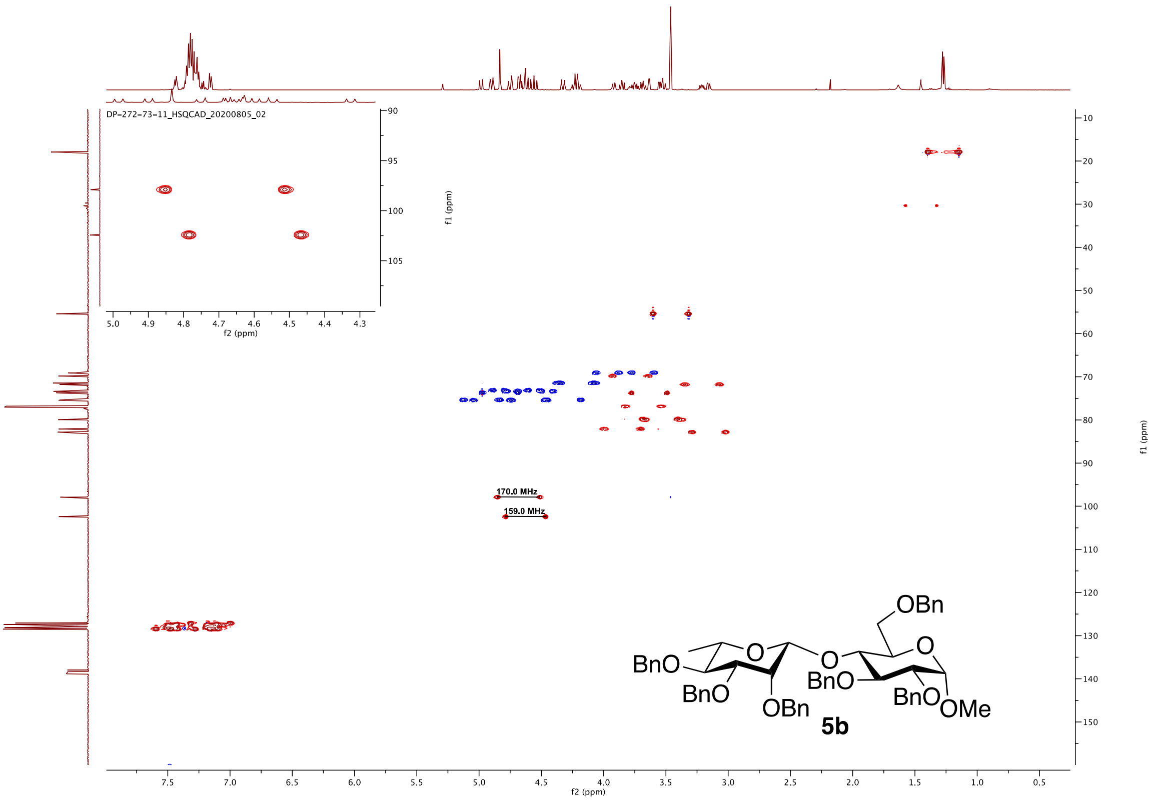

COSY (500 x 500 MHz, Chloroform-*d*) 5b

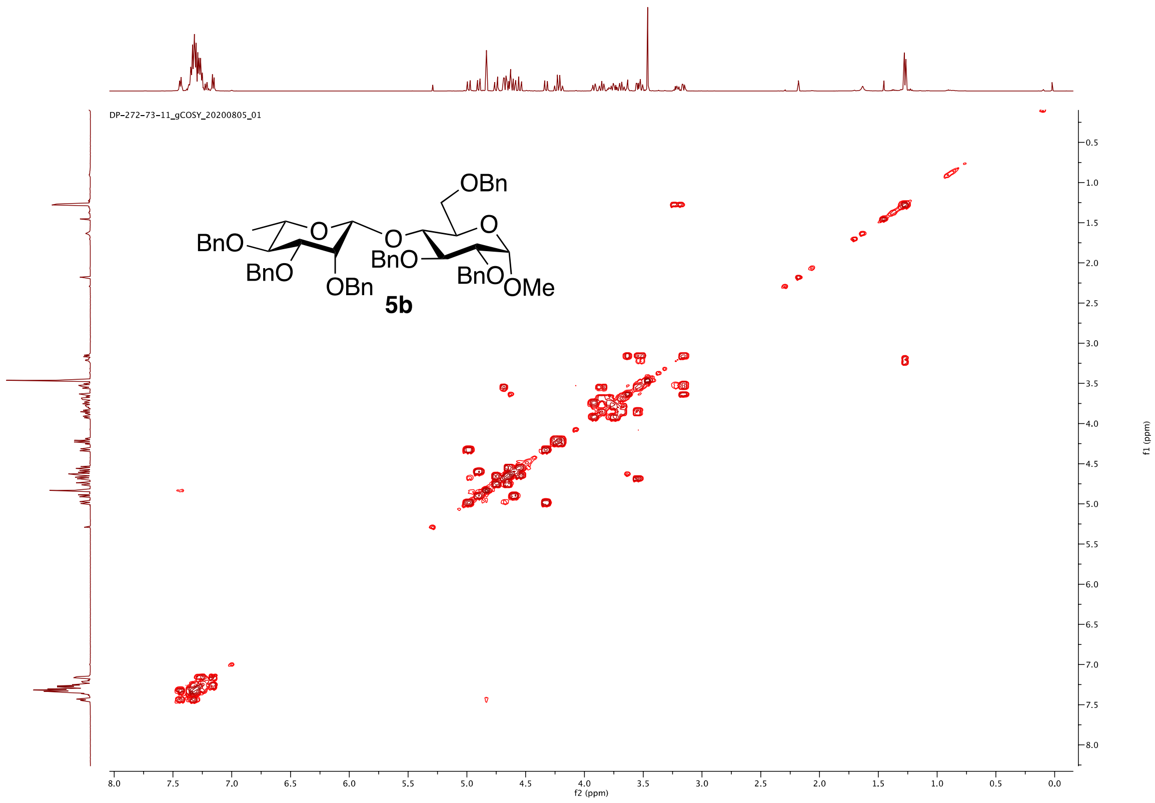

HMBC (500 x 126 MHz, Chloroform-*d*) 5b

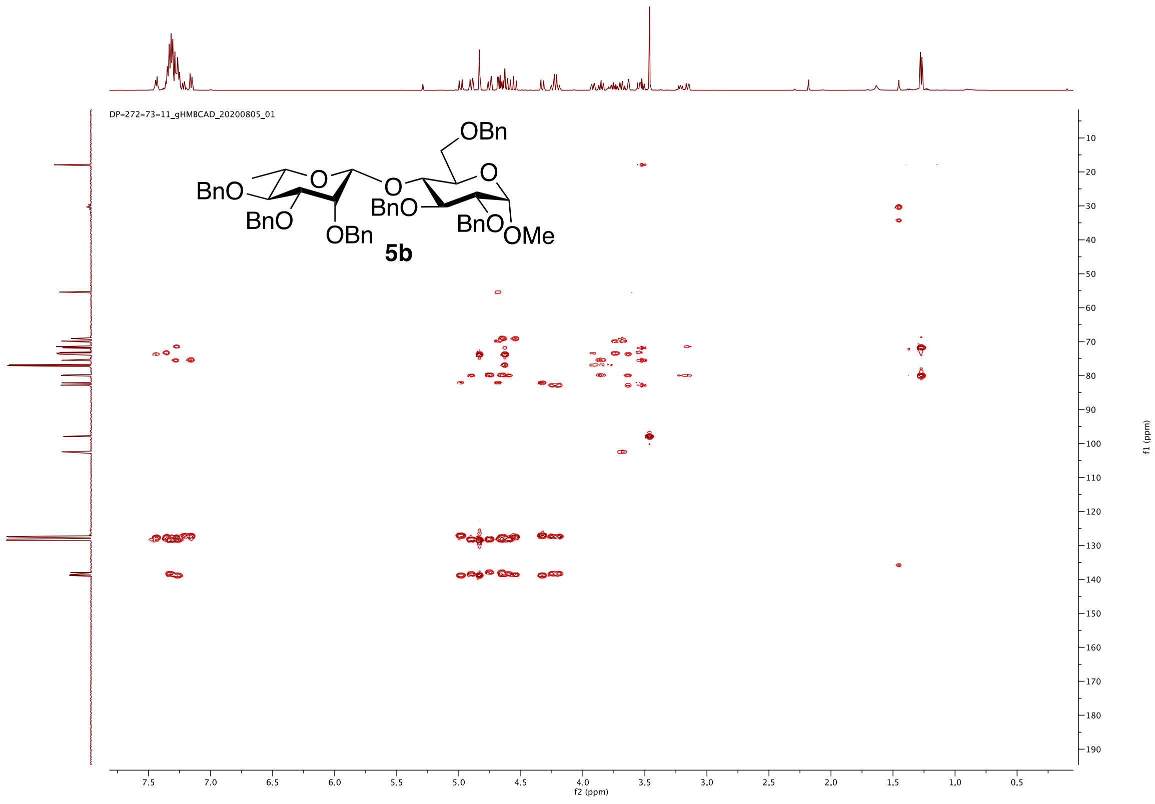

^{1}H NMR (500 MHz, Chloroform-*d*) 5c

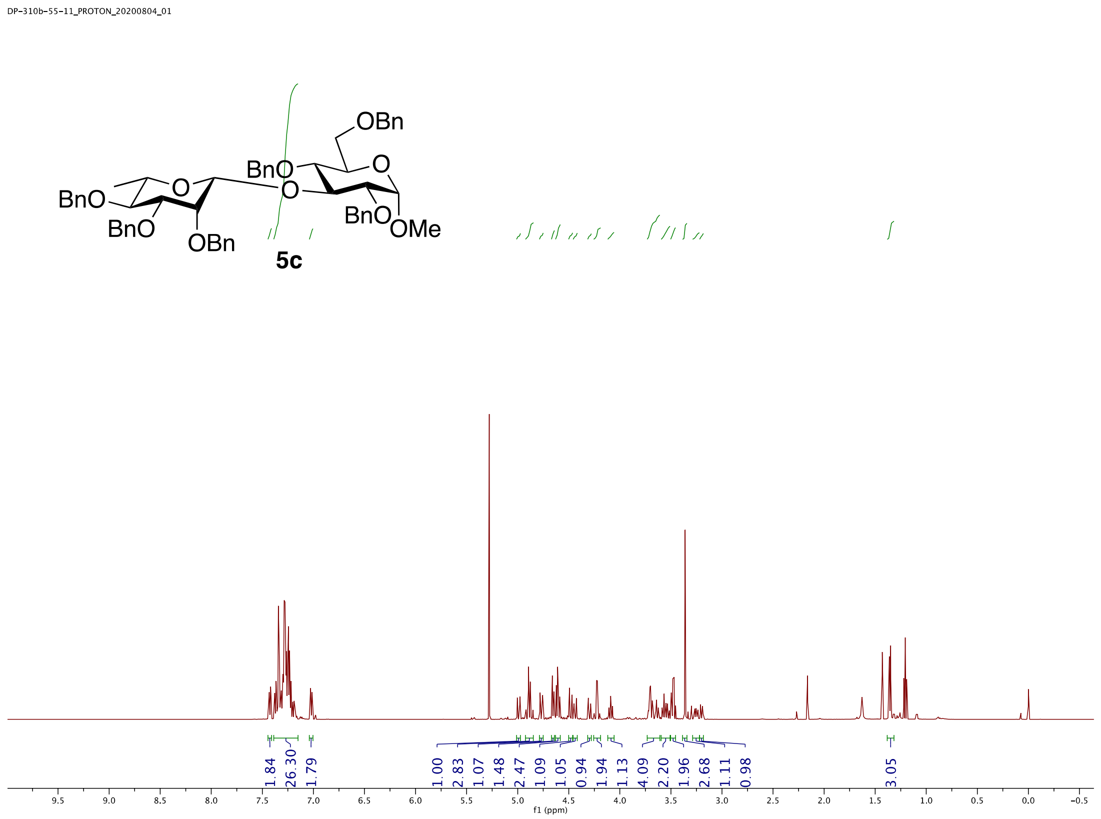

^{13}C NMR (126 MHz, Chloroform-*d*) 5c

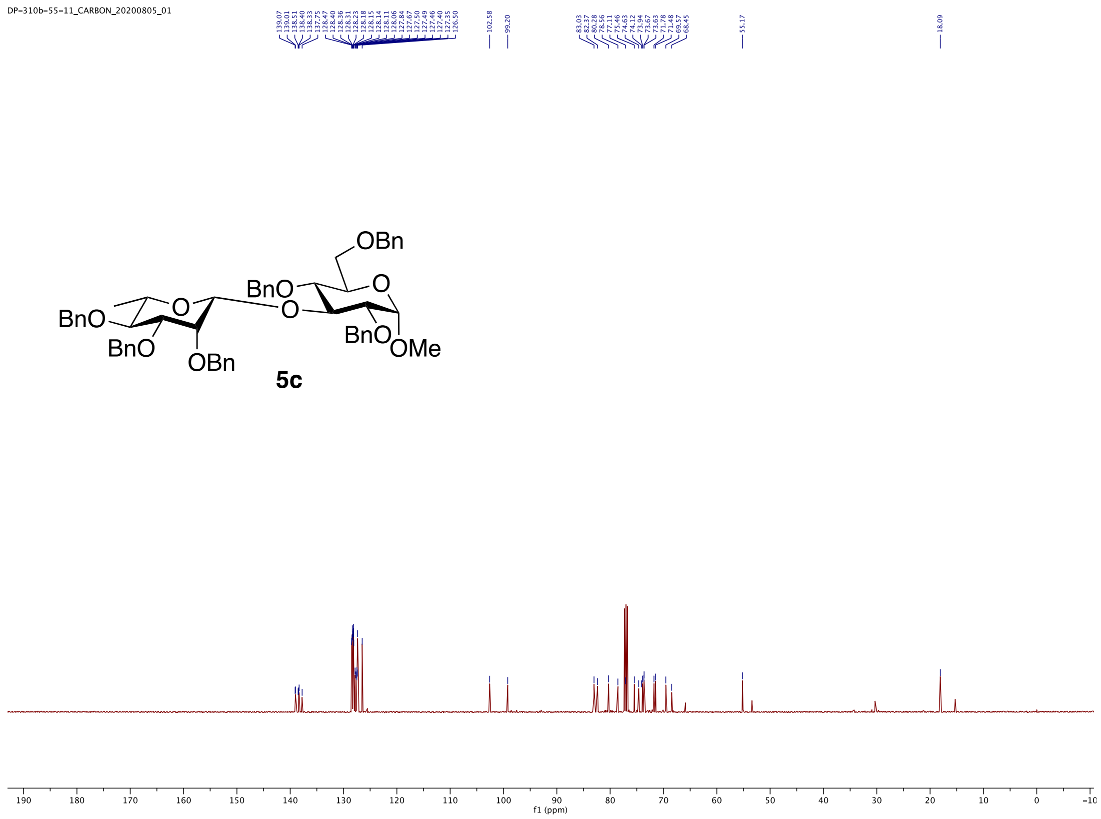

^{13}C - ^{1}H decoupled HSQC (500 x 126 MHz, Chloroform-*d*) 5c

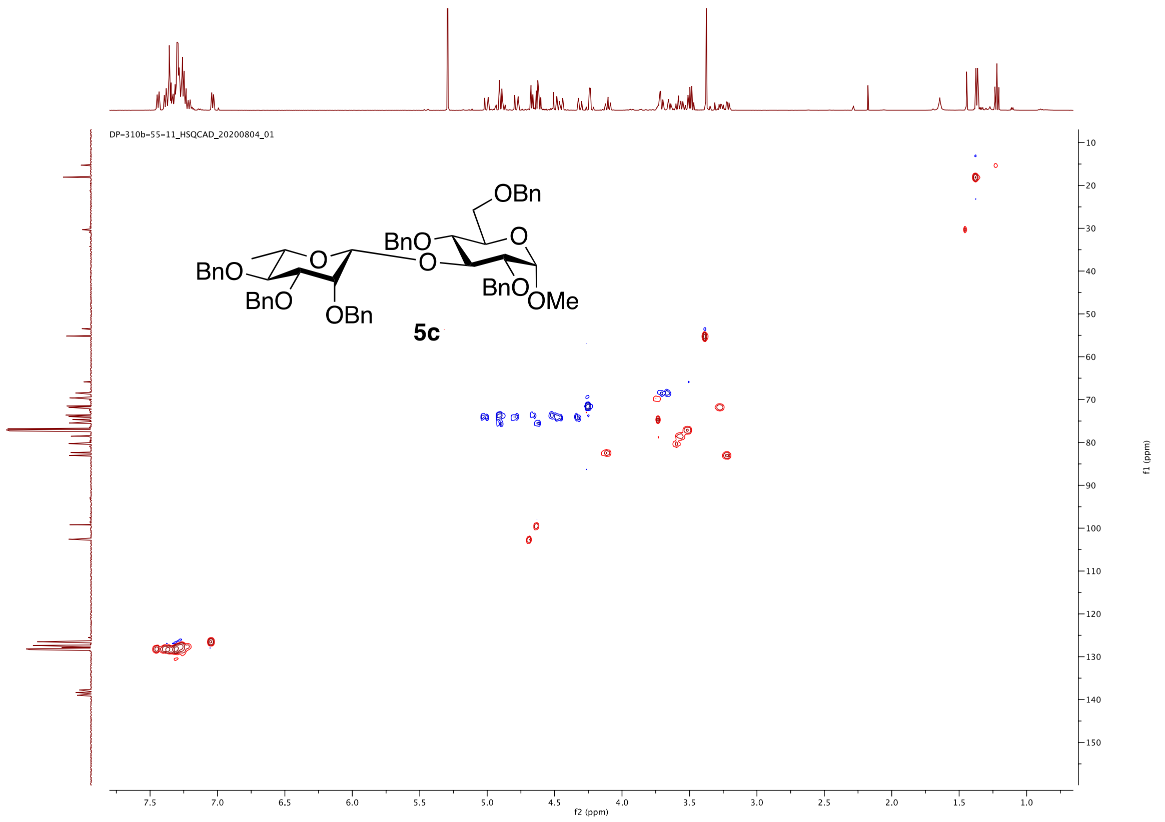

^{13}C - ^{1}H coupled HSQC (500 x 126 MHz, Chloroform-*d*) 5c

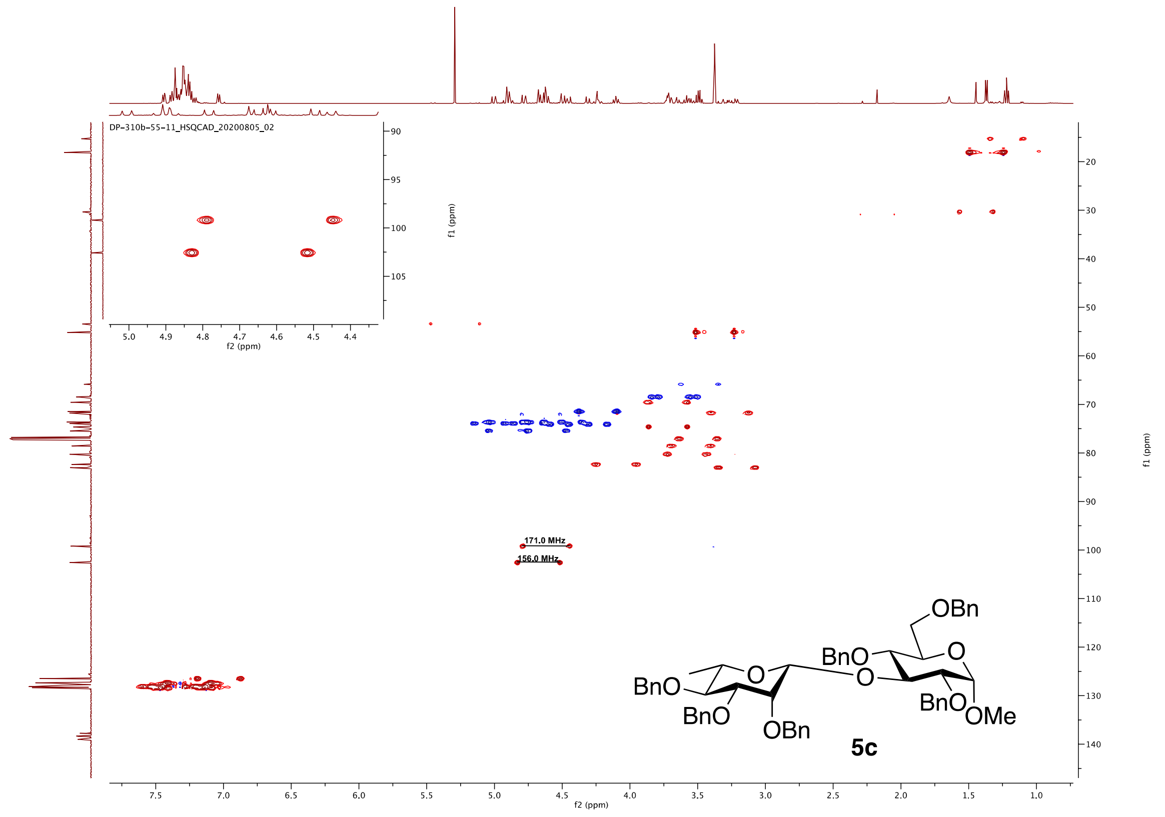

COSY (500 x 500 MHz, Chloroform-*d*) 5c

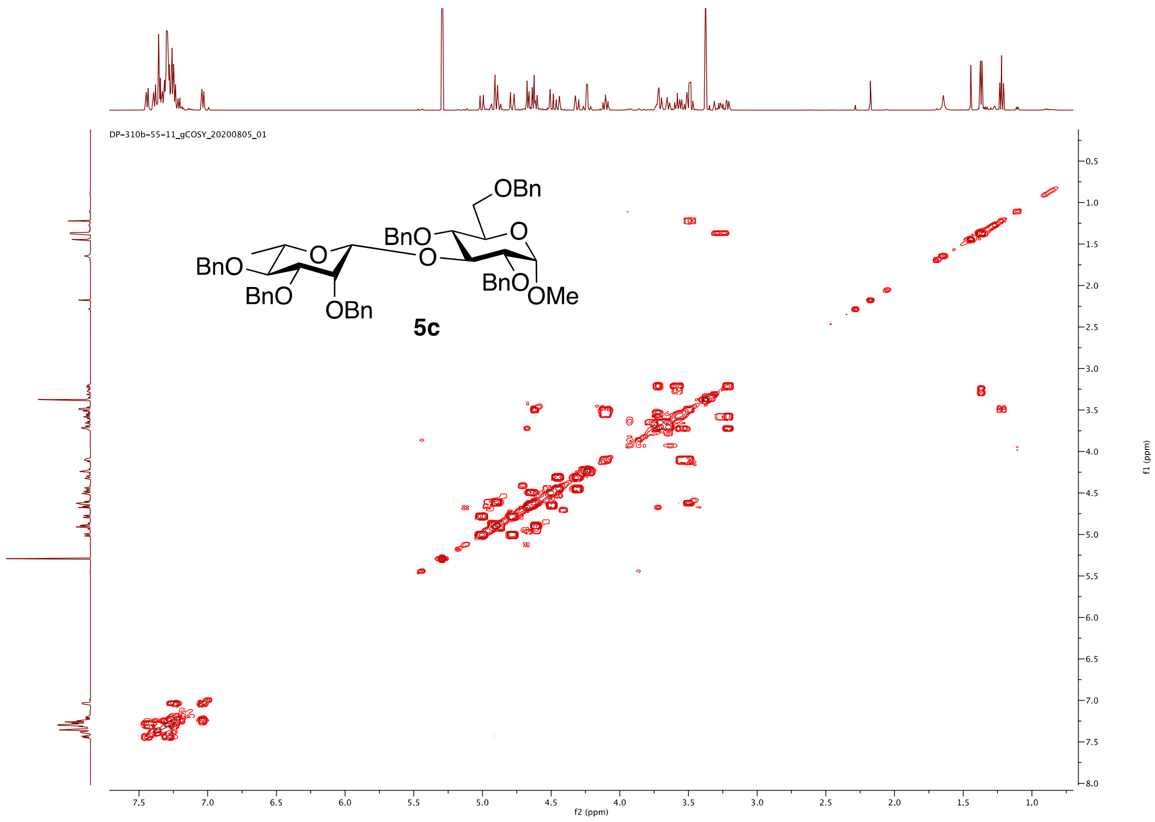

HMBC (500 x 126 MHz, Chloroform-*d*) 5c

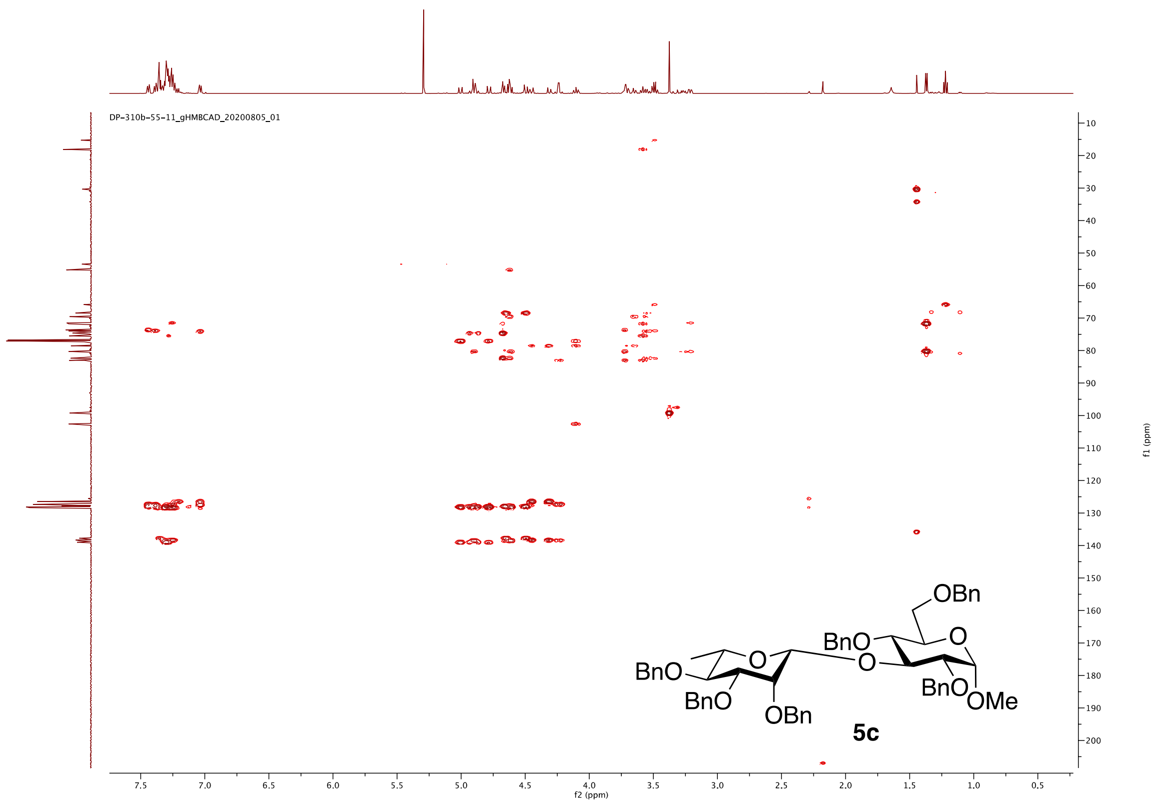

^{1}H NMR (500 MHz, Chloroform-*d*) 5d

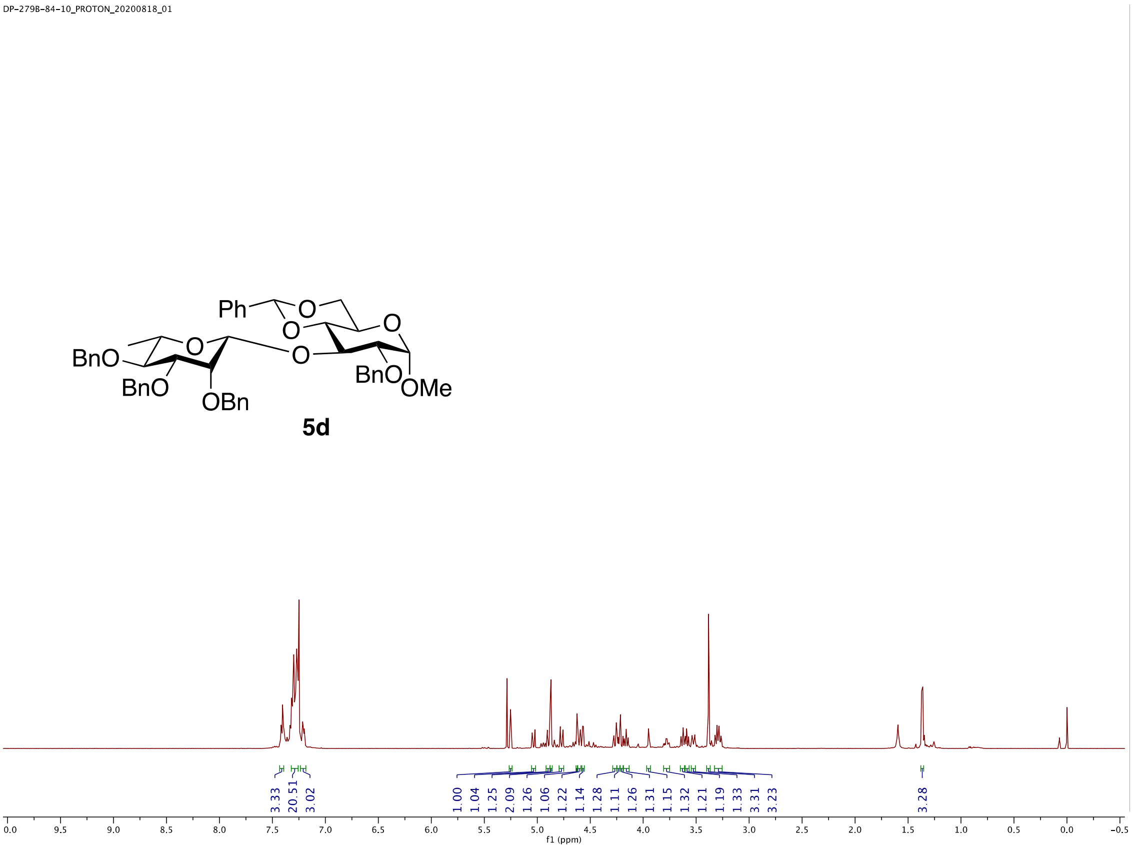

^{13}C NMR (126 MHz, Chloroform-*d*) 5d

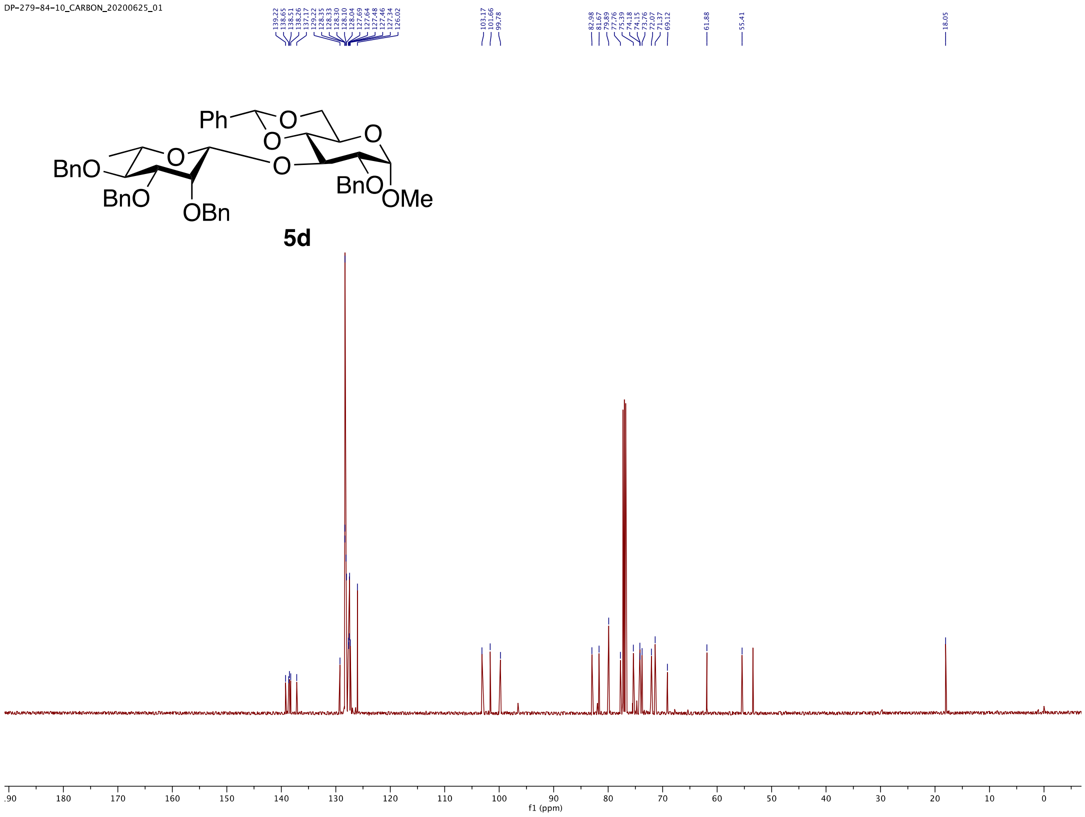

^{13}C - ^{1}H decoupled HSQC (500 x 126 MHz, Chloroform-*d*) 5d

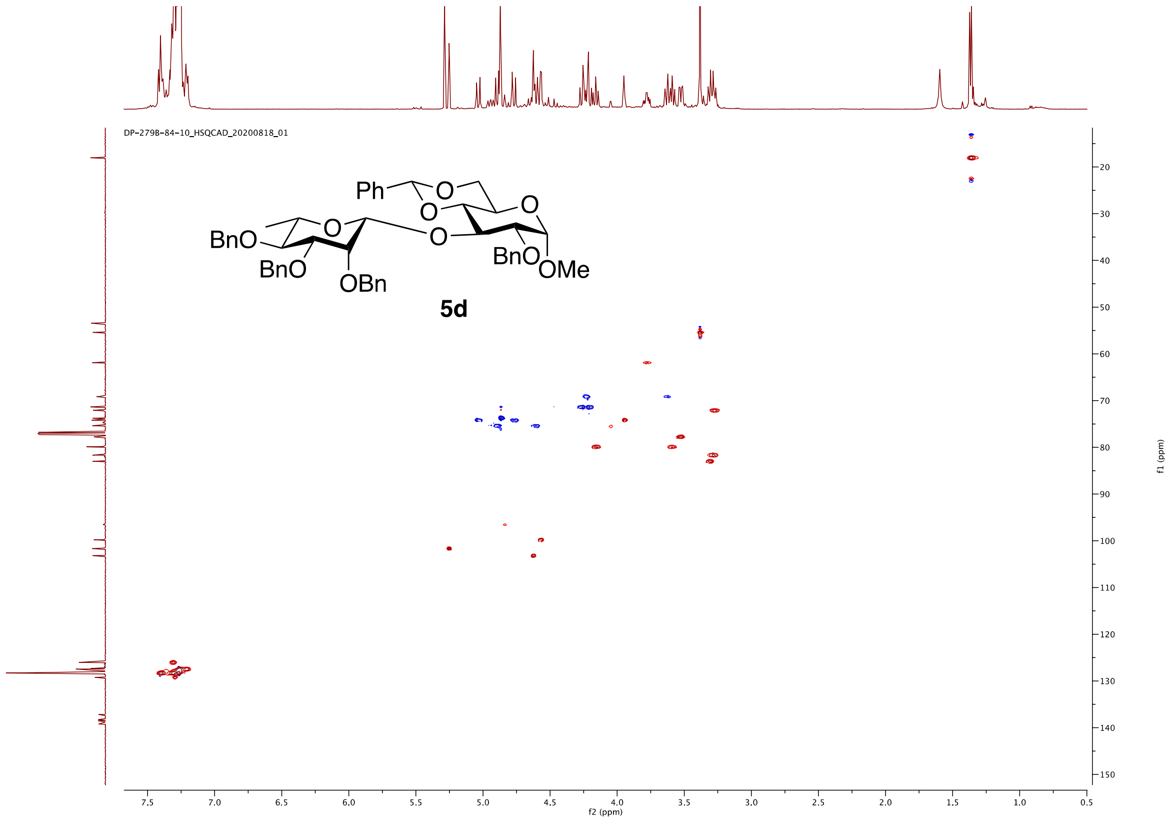

^{13}C - ^{1}H coupled HSQC (500 x 126 MHz, Chloroform-*d*) 5d

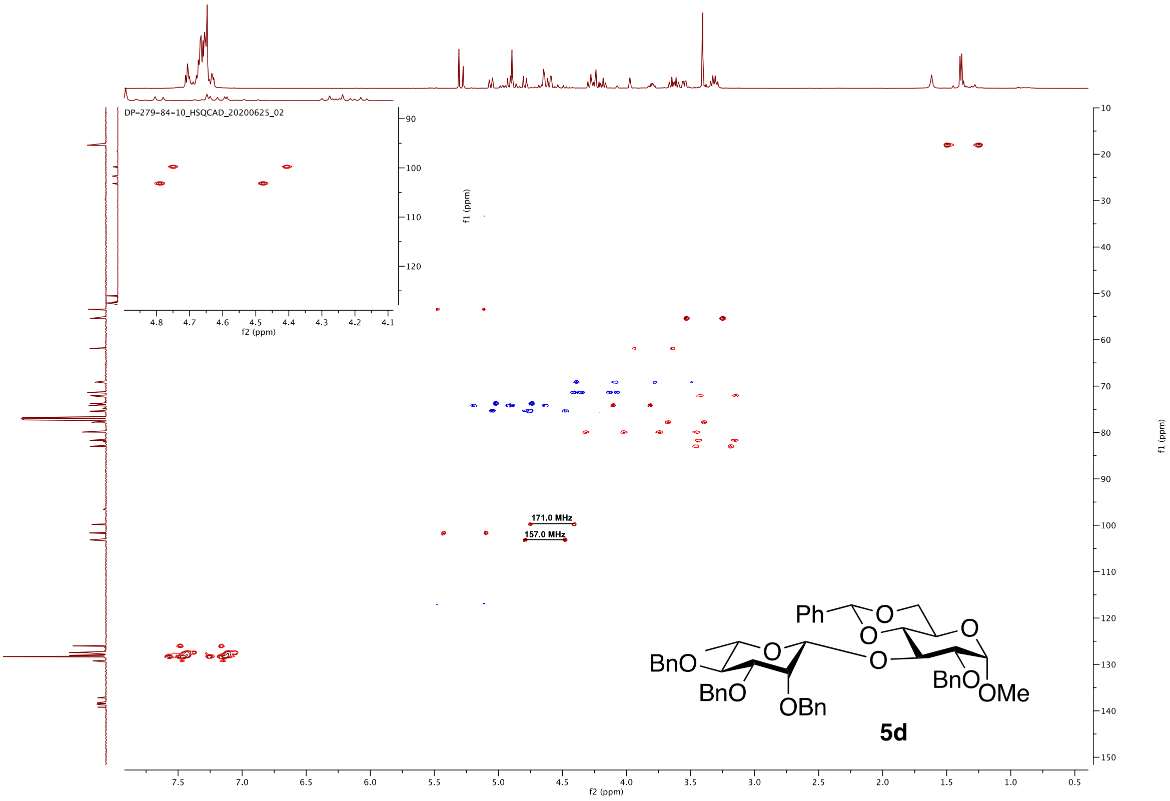

COSY (500 x 500 MHz, Chloroform-*d*) 5d

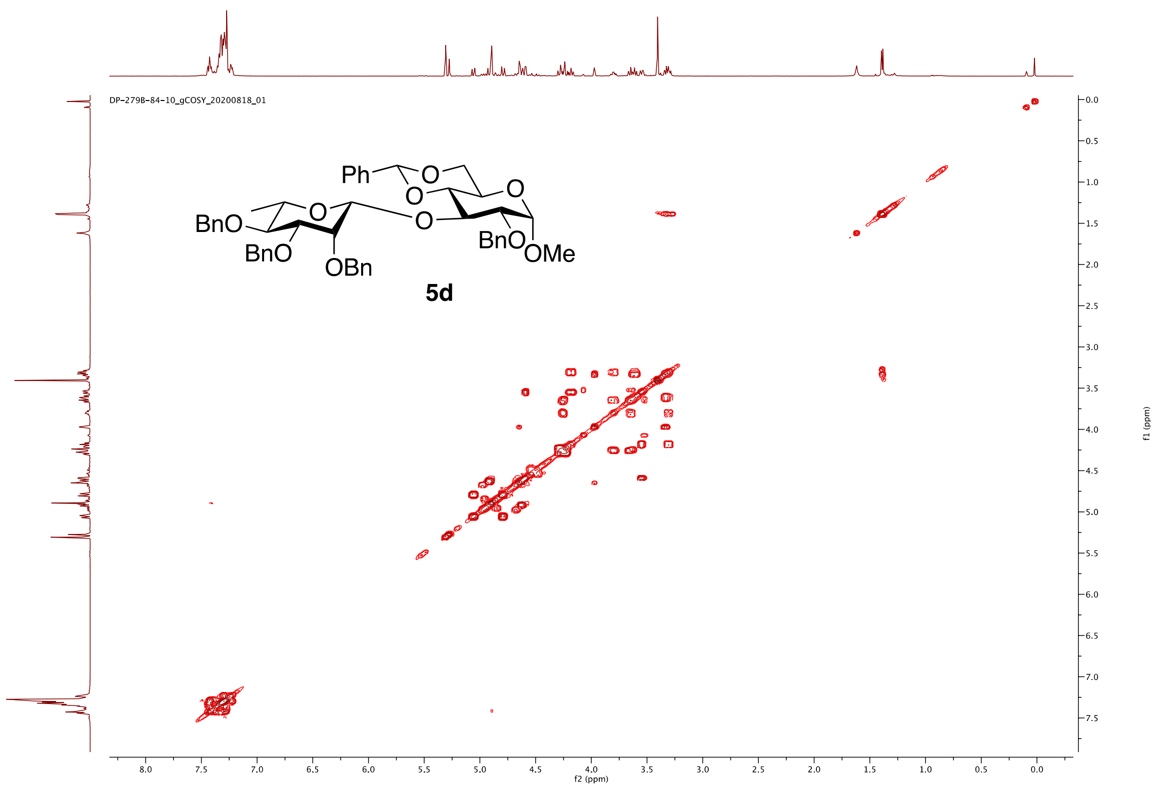

HMBC (500 x 126 MHz, Chloroform-*d*) 5d

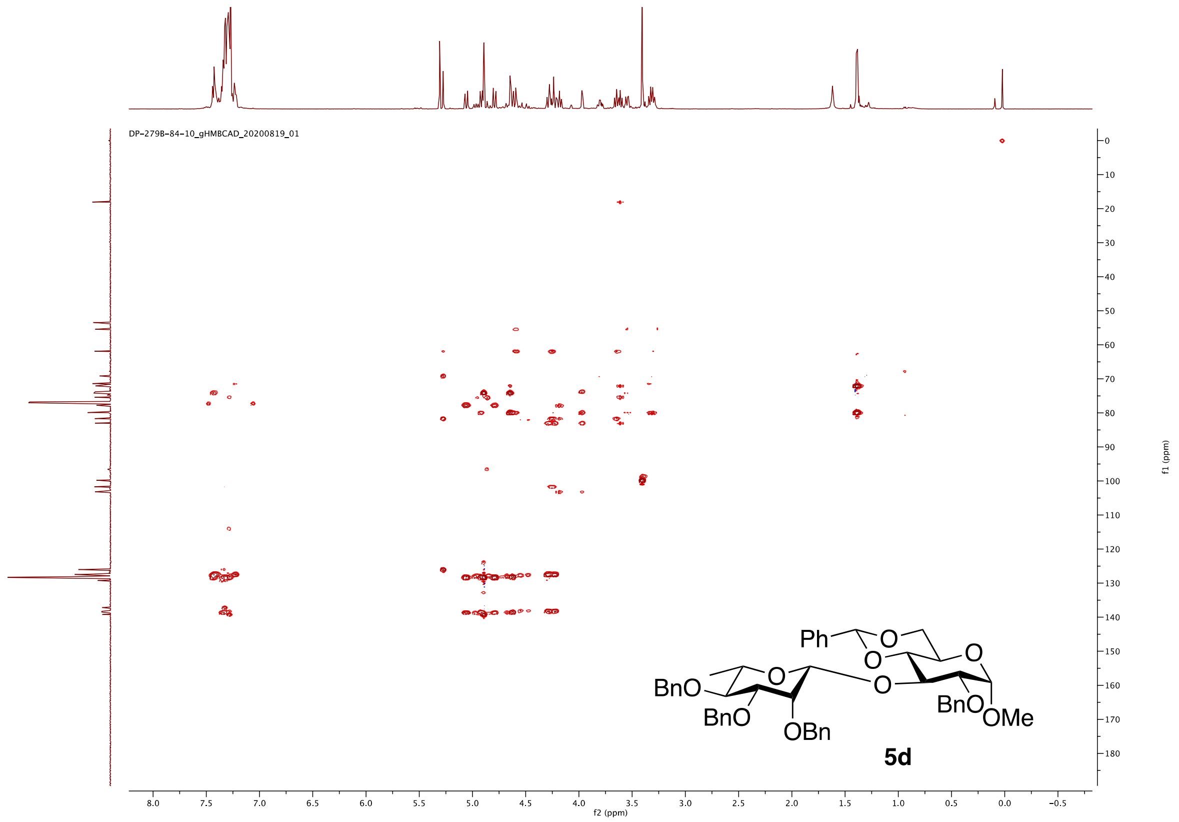

^{1}H NMR (500 MHz, Chloroform-*d*) 5e

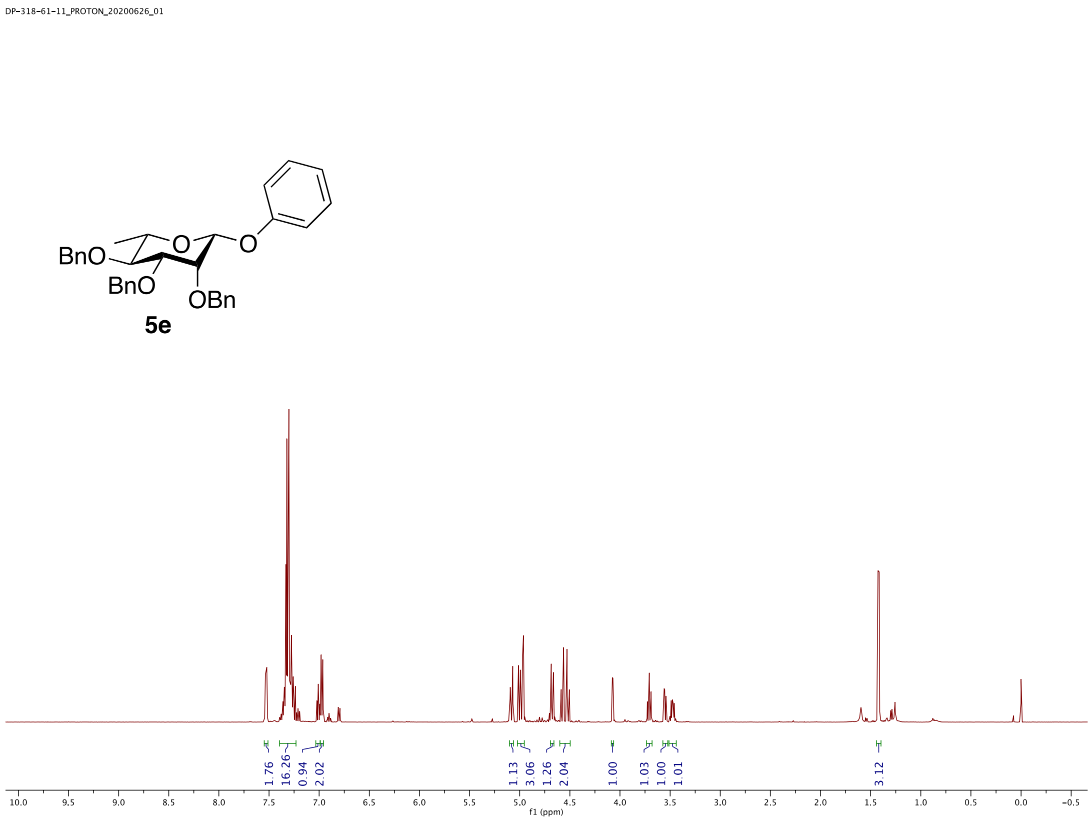

^{13}C NMR (126 MHz, Chloroform-*d*) 5e

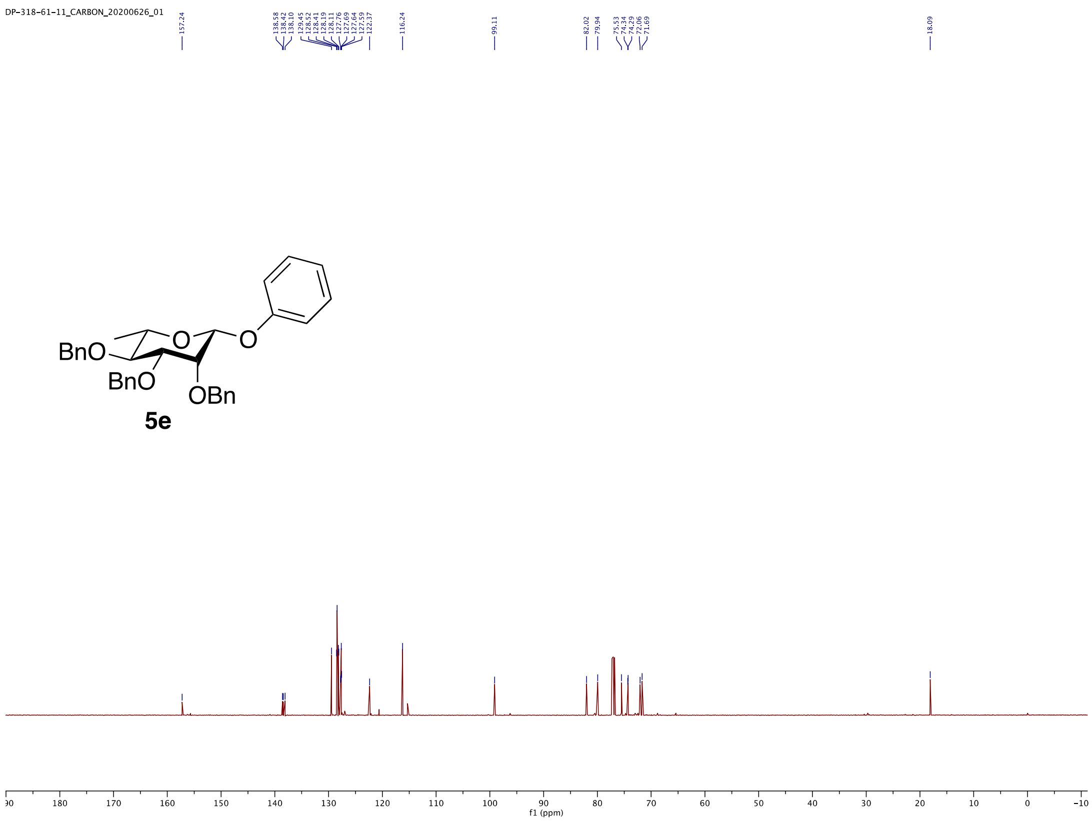

^{13}C - ^{1}H decoupled HSQC (500 x 126 MHz, Chloroform-*d*) 5e

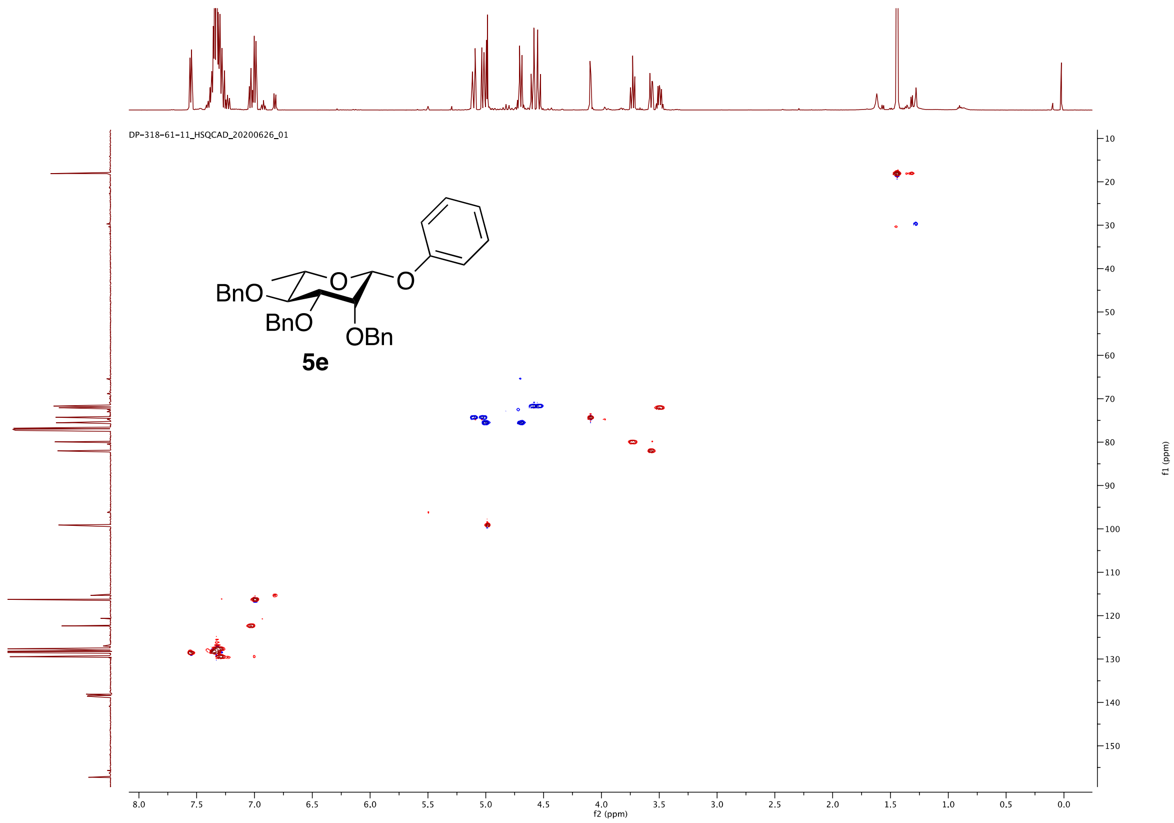

^{13}C - ^{1}H coupled HSQC (500 x 126 MHz, Chloroform-*d*) 5e

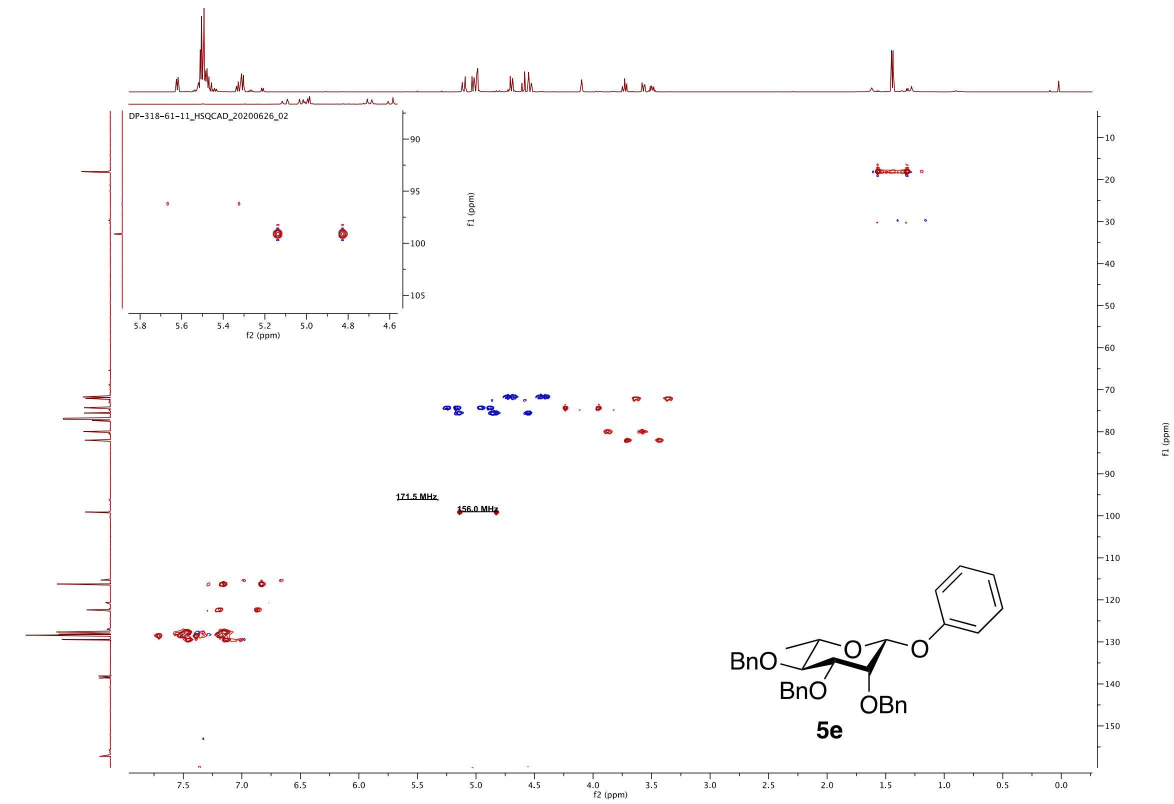

COSY (500 x 500 MHz, Chloroform-*d*) 5e

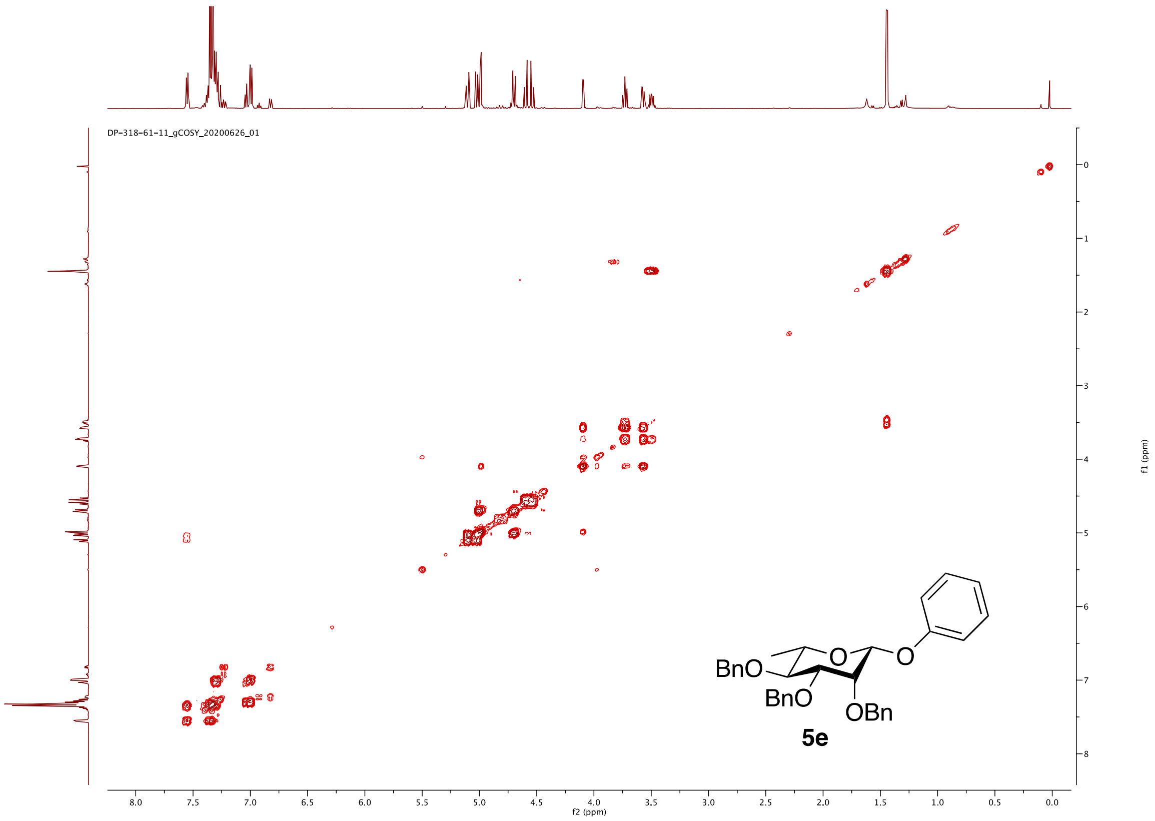

HMBC (500 x 126 MHz, Chloroform-*d*) 5e

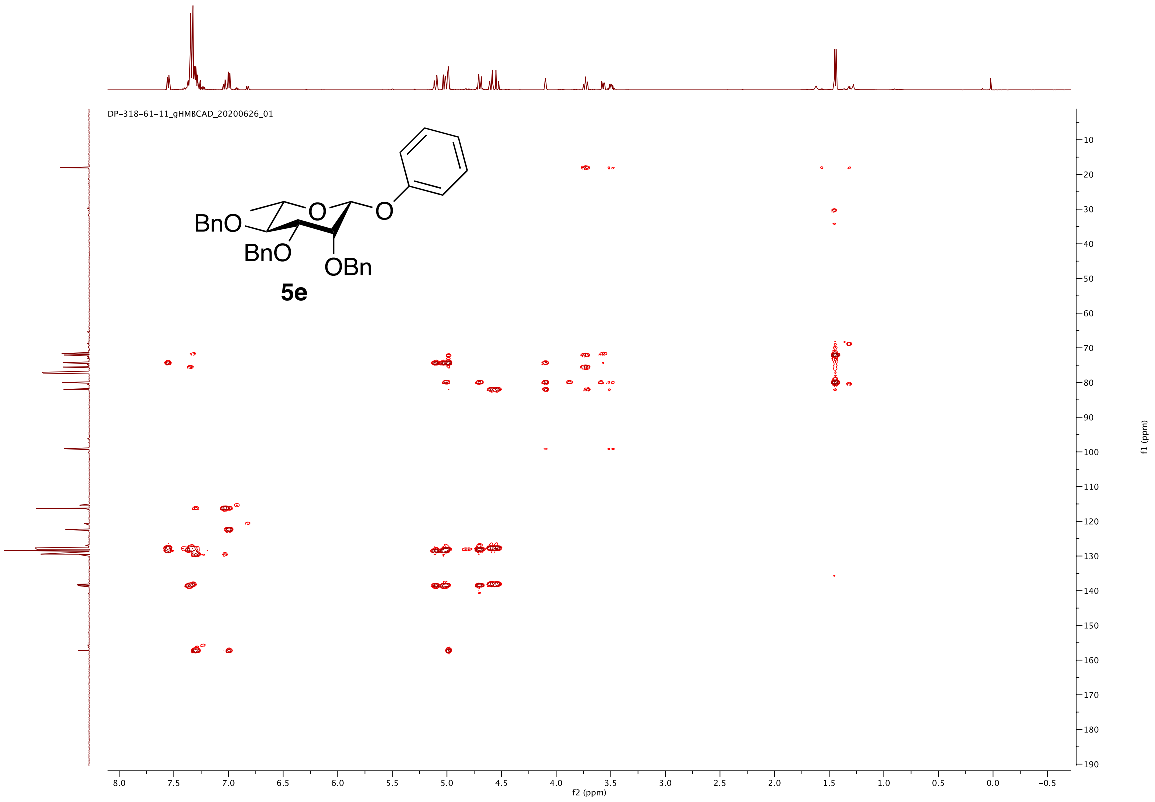

^{1}H NMR (600 MHz, Chloroform-*d*) 5f

DP-330-67-11_PROTON_20200728_01
DP-330-67-11

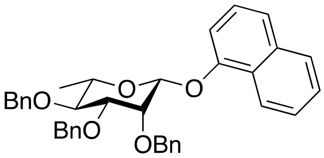

5f

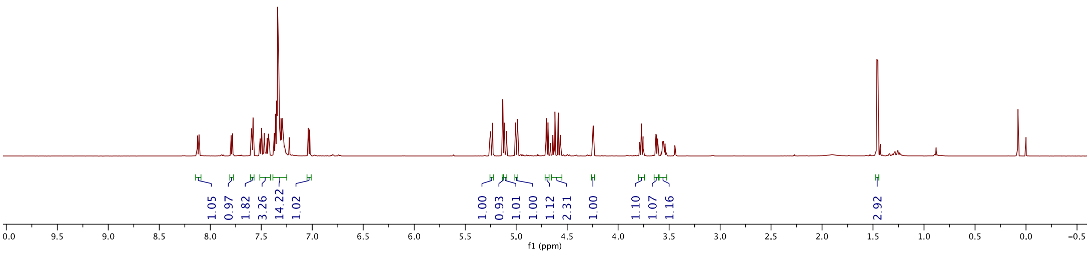

^{3}C NMR (151 MHz, Chloroform-*d*) 5f

DP-330-67-11_CARBON_20200728_01
DP-330-67-11

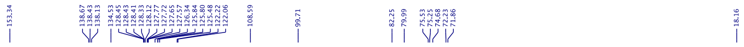

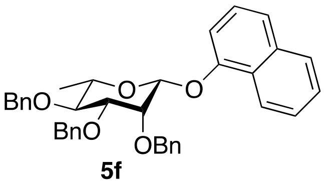

5f

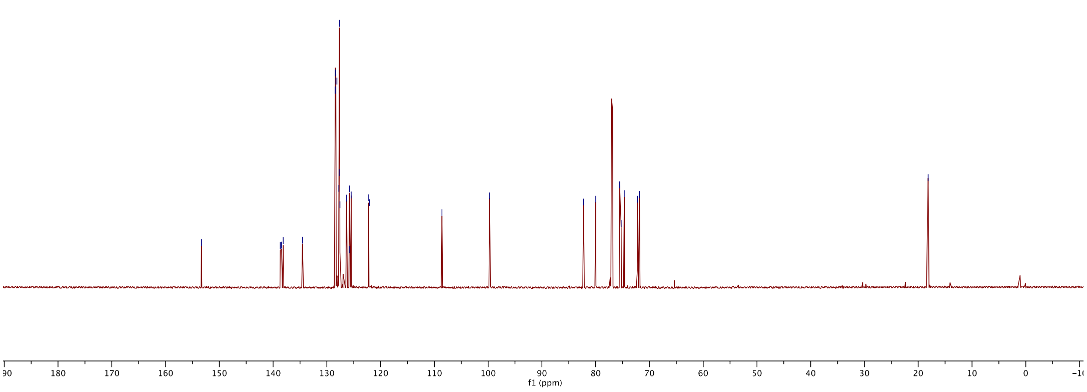

^{13}C - ^{1}H decoupled HSQC (600 x 151 MHz, Chloroform-*d*) 5f

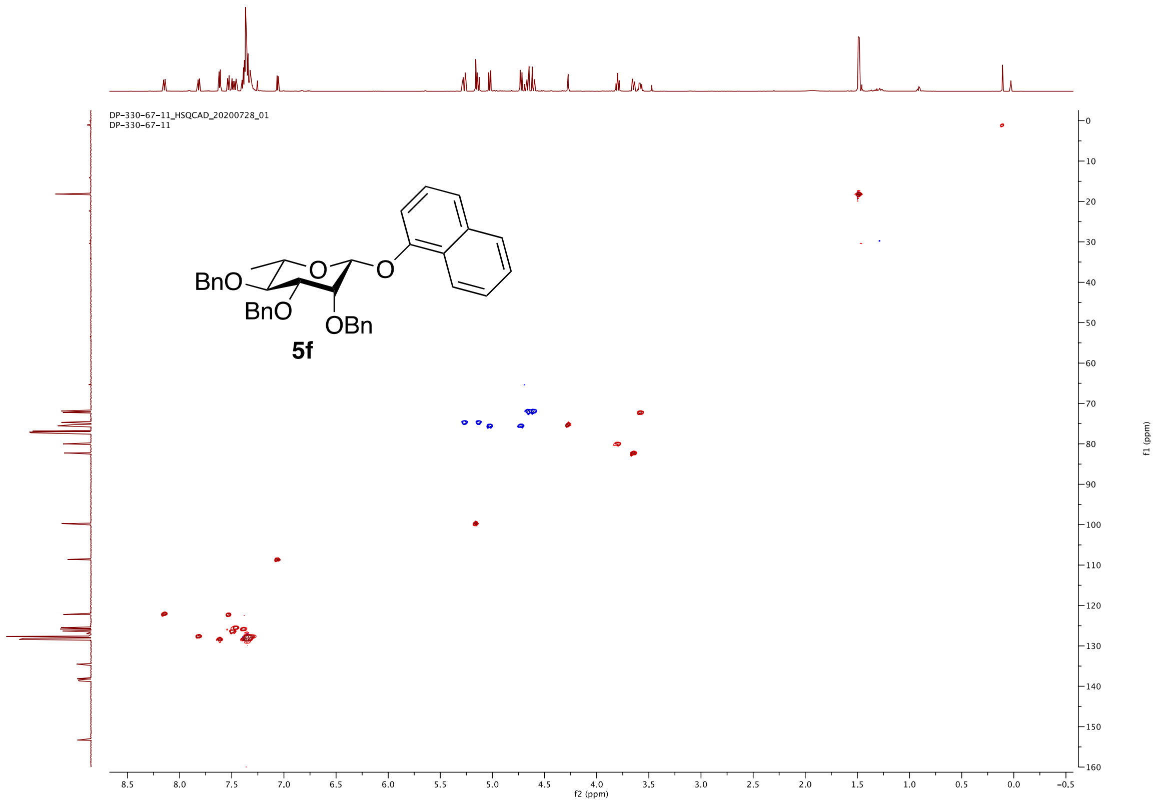

^{13}C - ^{1}H coupled HSQC (600 x 156 MHz, Chloroform-*d*) 5f

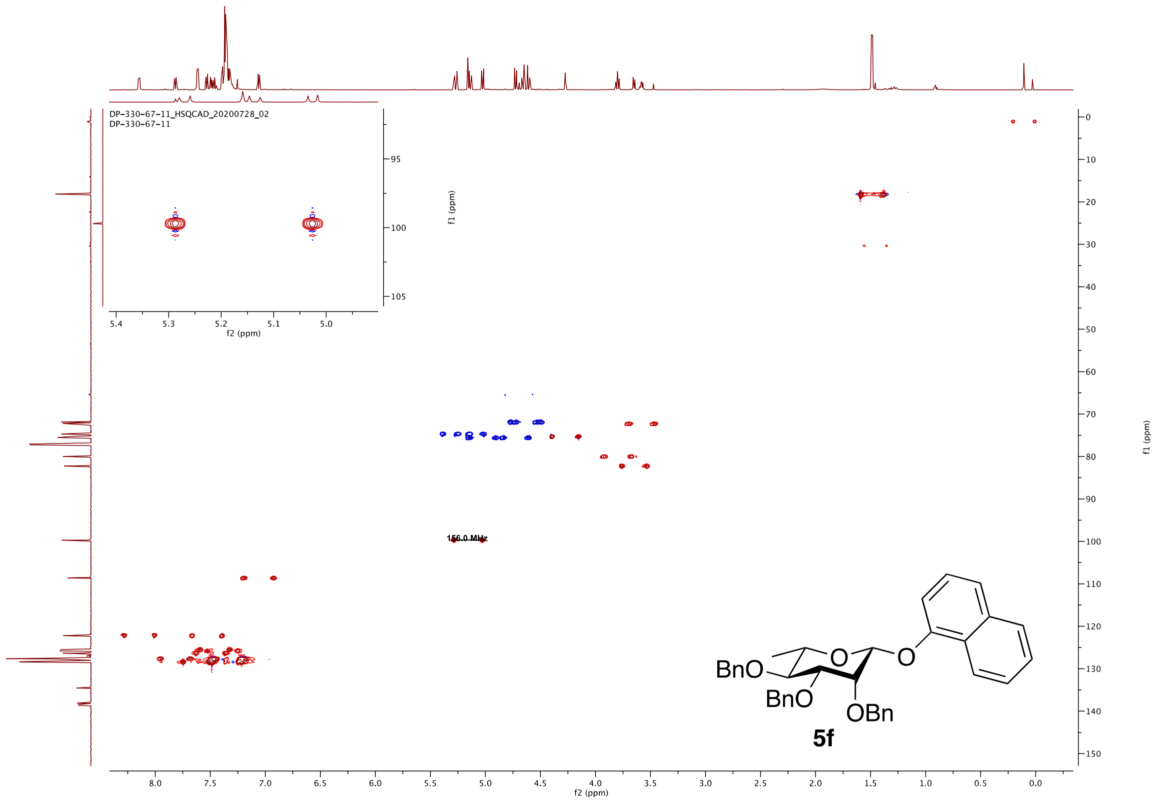

COSY (600 x 600 MHz, Chloroform-*d*) 5f

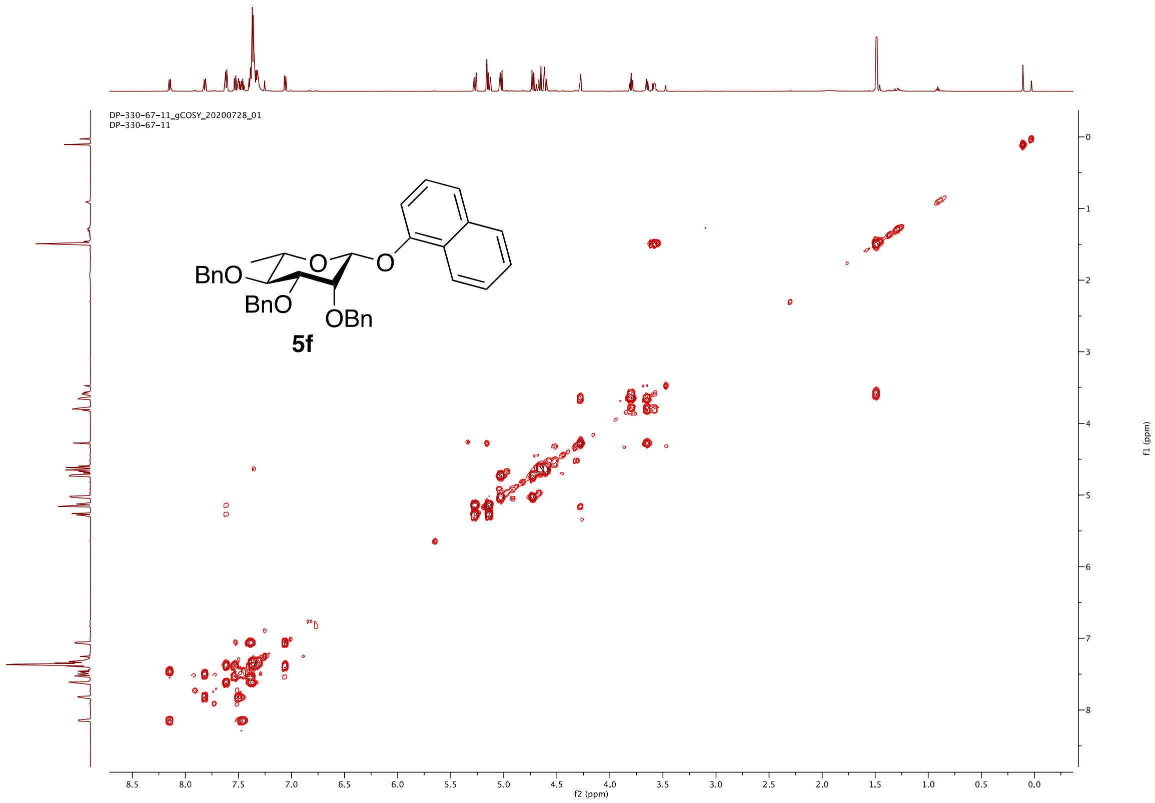

HMBC (600 x 151 MHz, Chloroform-*d*) 5f

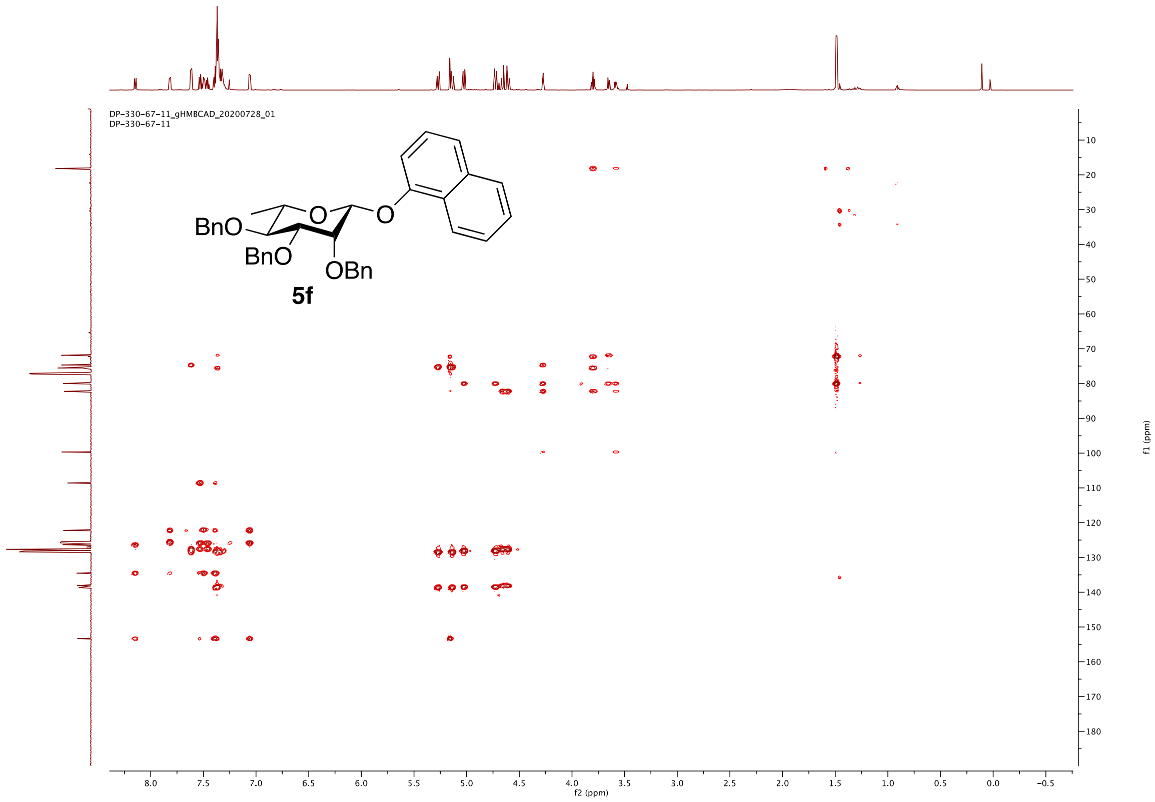

^{1}H NMR (500 MHz, Chloroform-*d*) 5g

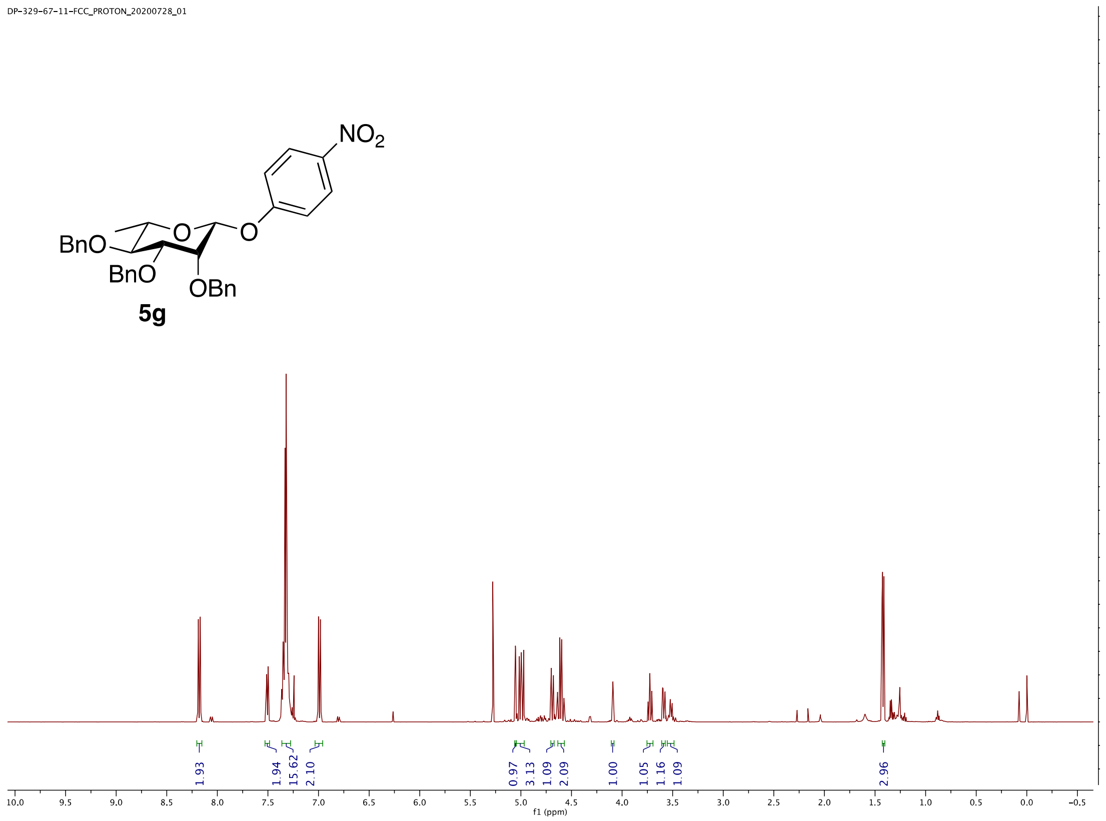

^{13}C NMR (126 MHz, Chloroform-*d*) 5g

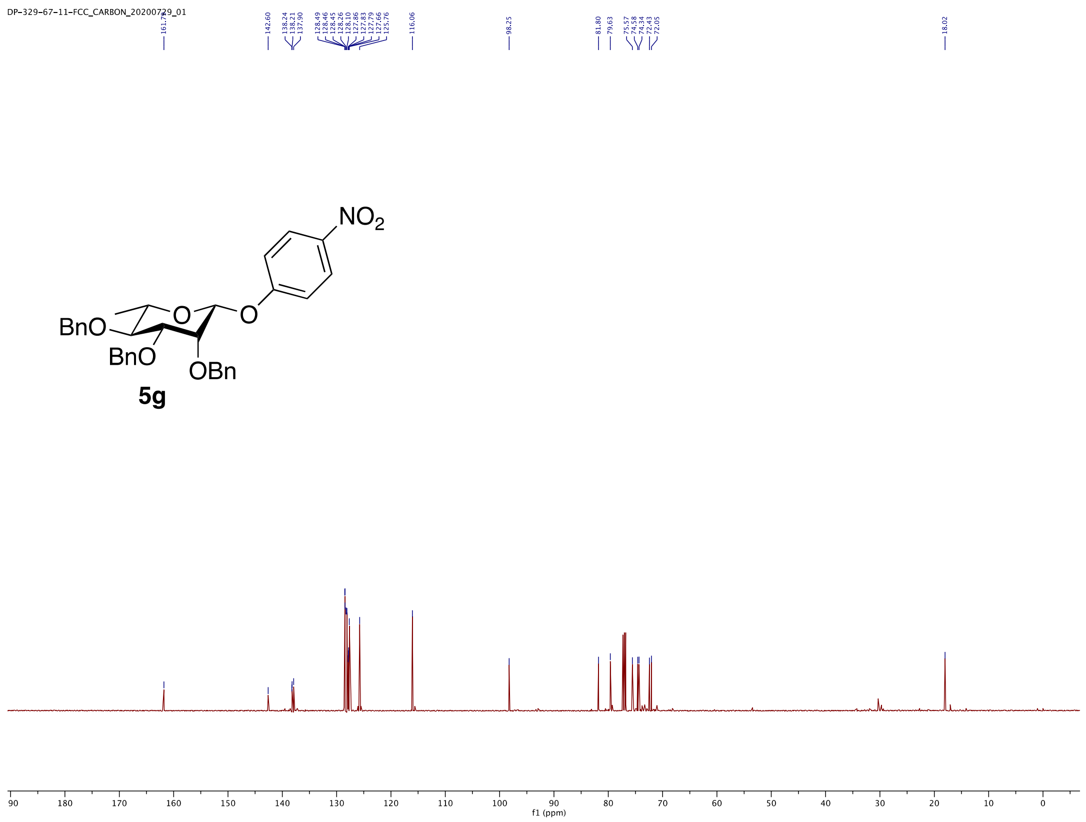

^{13}C - ^{1}H decoupled HSQC (500 x 126 MHz, Chloroform-*d*) 5g

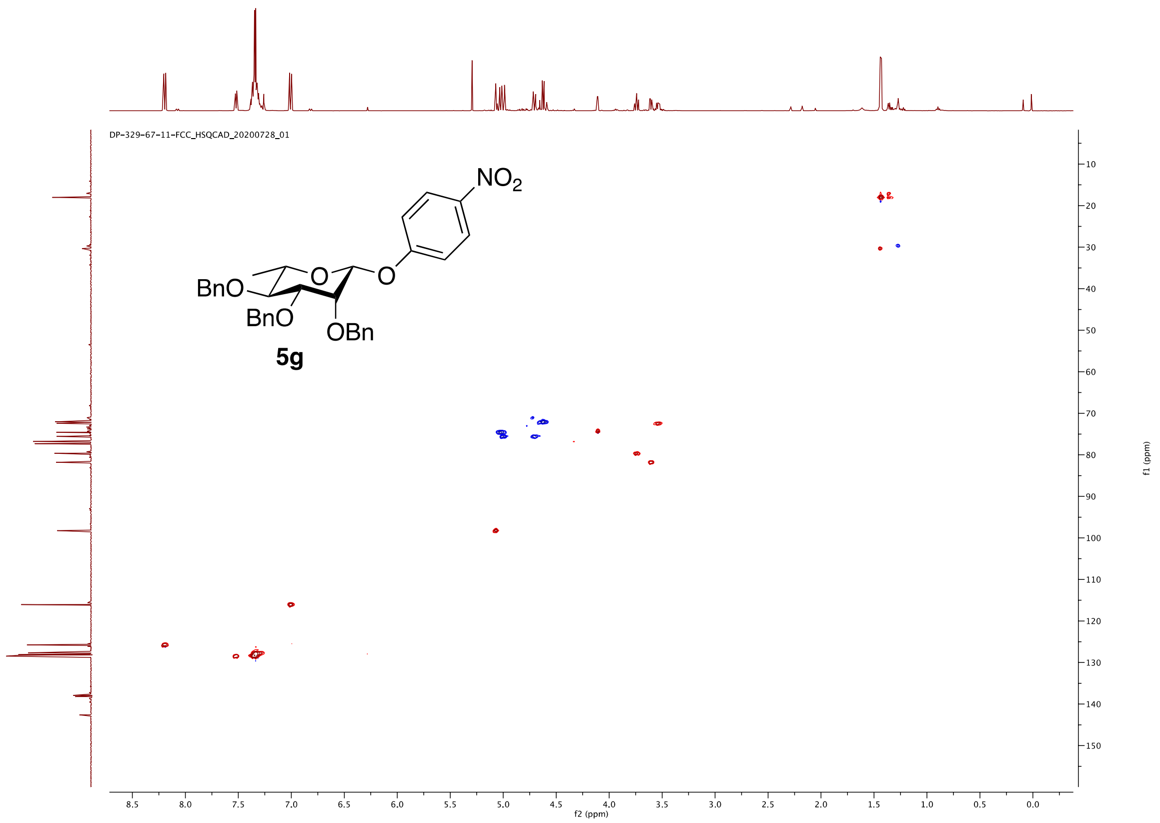

^{13}C - ^{1}H coupled HSQC (500 x 126 MHz, Chloroform-*d*) 5g

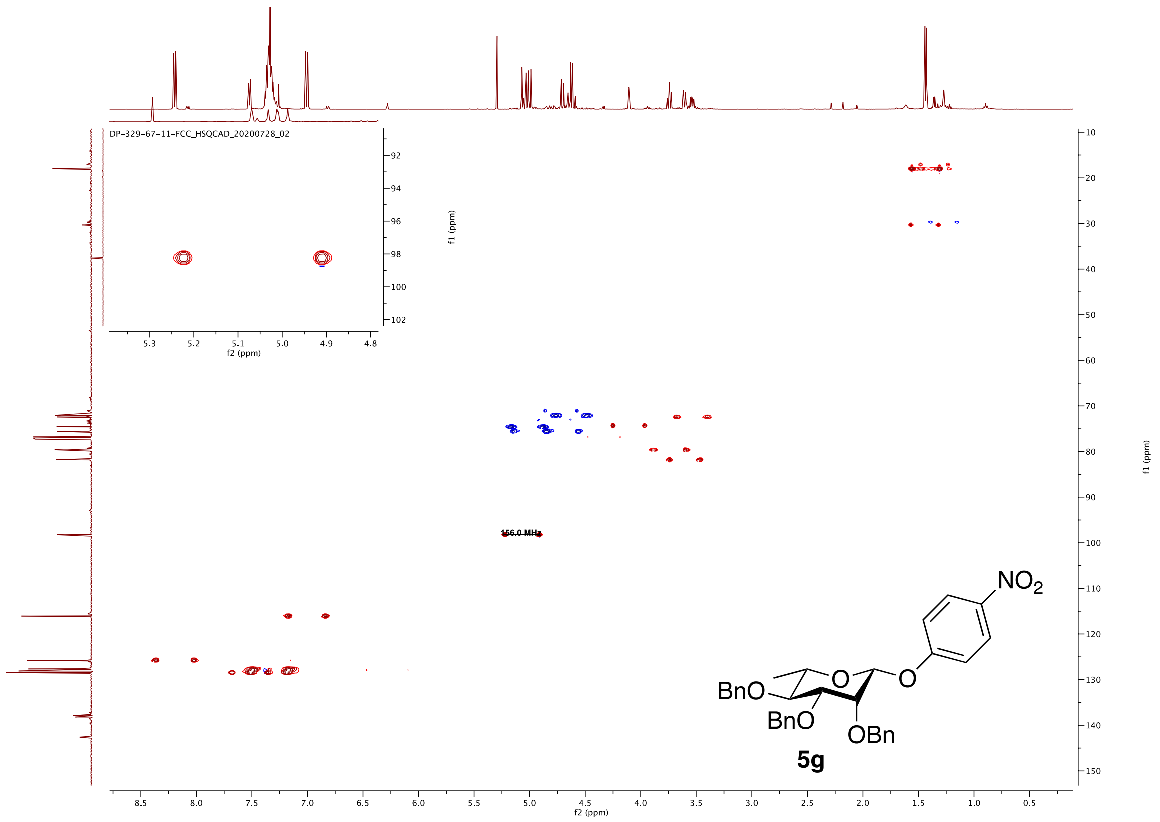

COSY (500 x 500 MHz, Chloroform-*d*) 5g

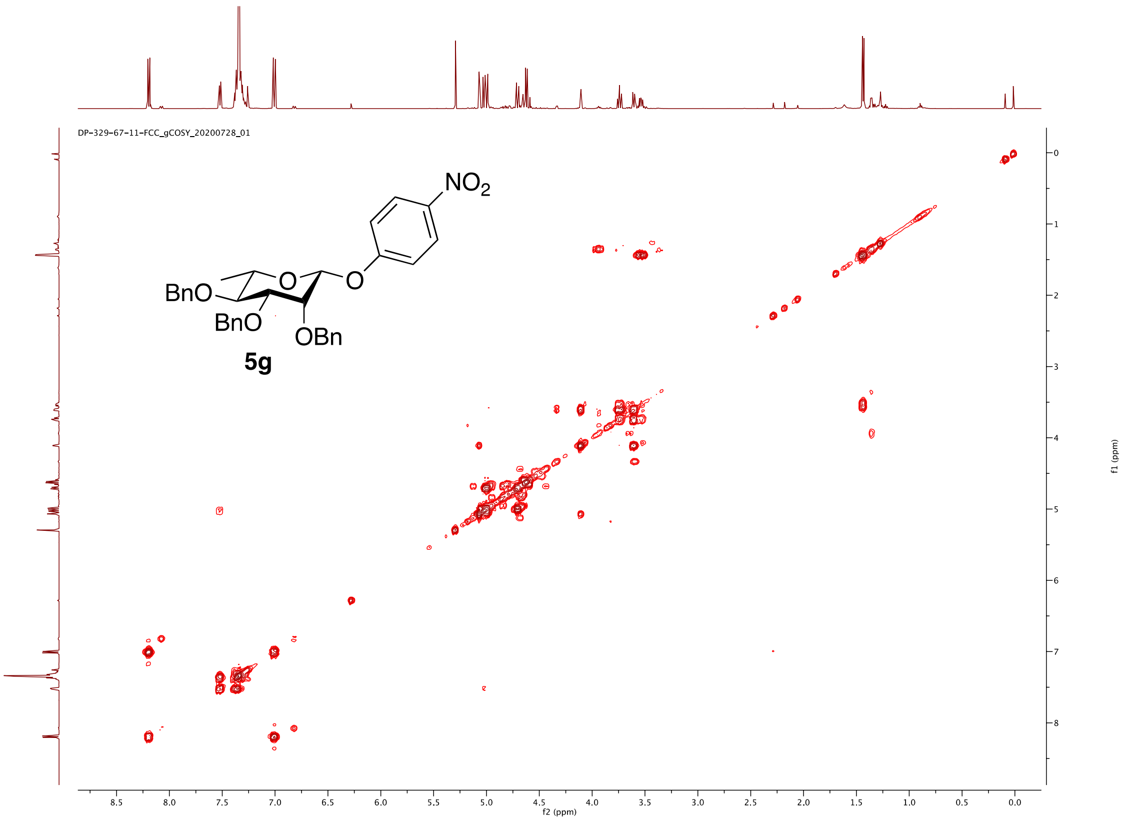

HMBC (500 x 126 MHz, Chloroform-*d*) 5g

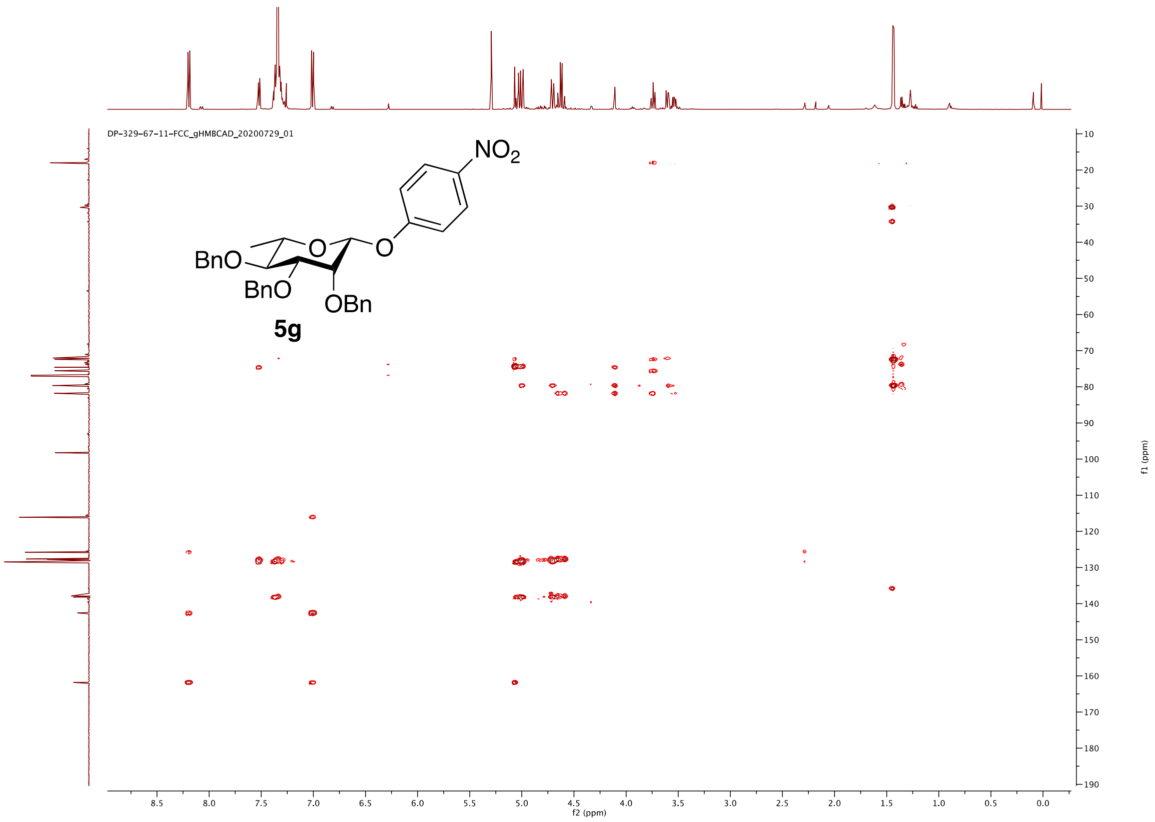

^{1}H NMR (500 MHz, Chloroform-*d*) 5j

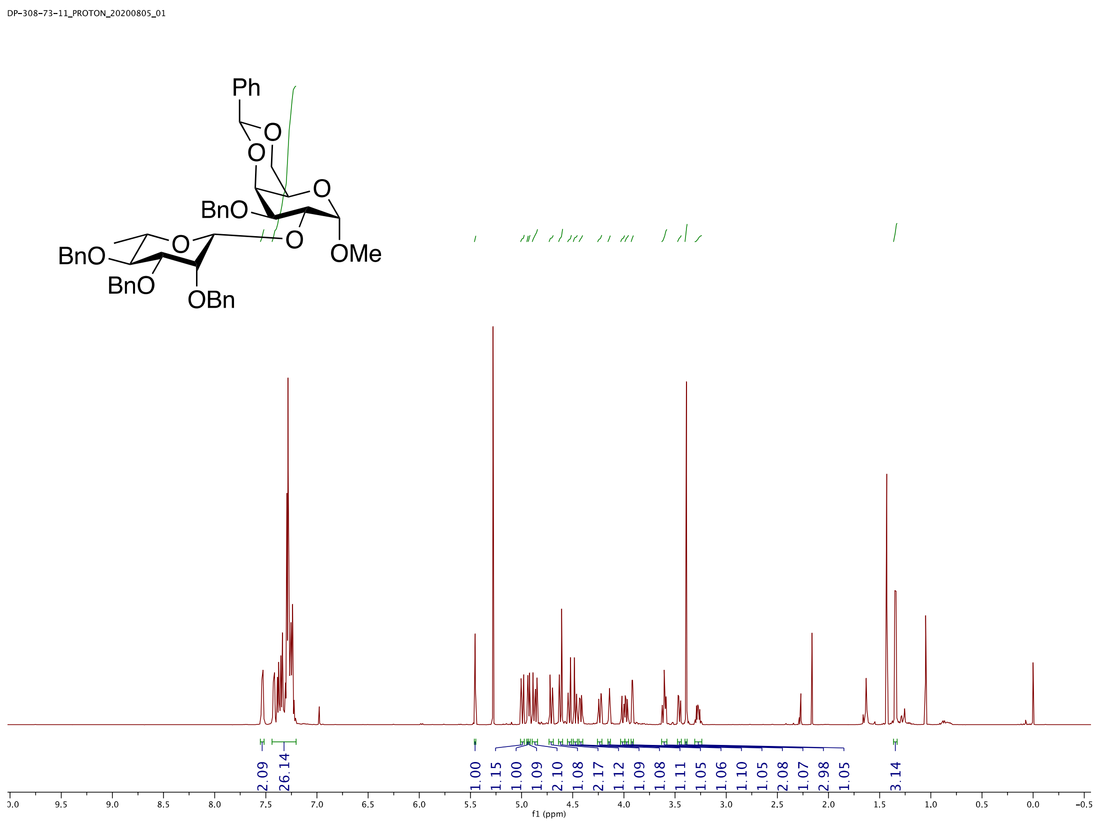

^{13}C NMR (126 MHz, Chloroform-*d*) 5j

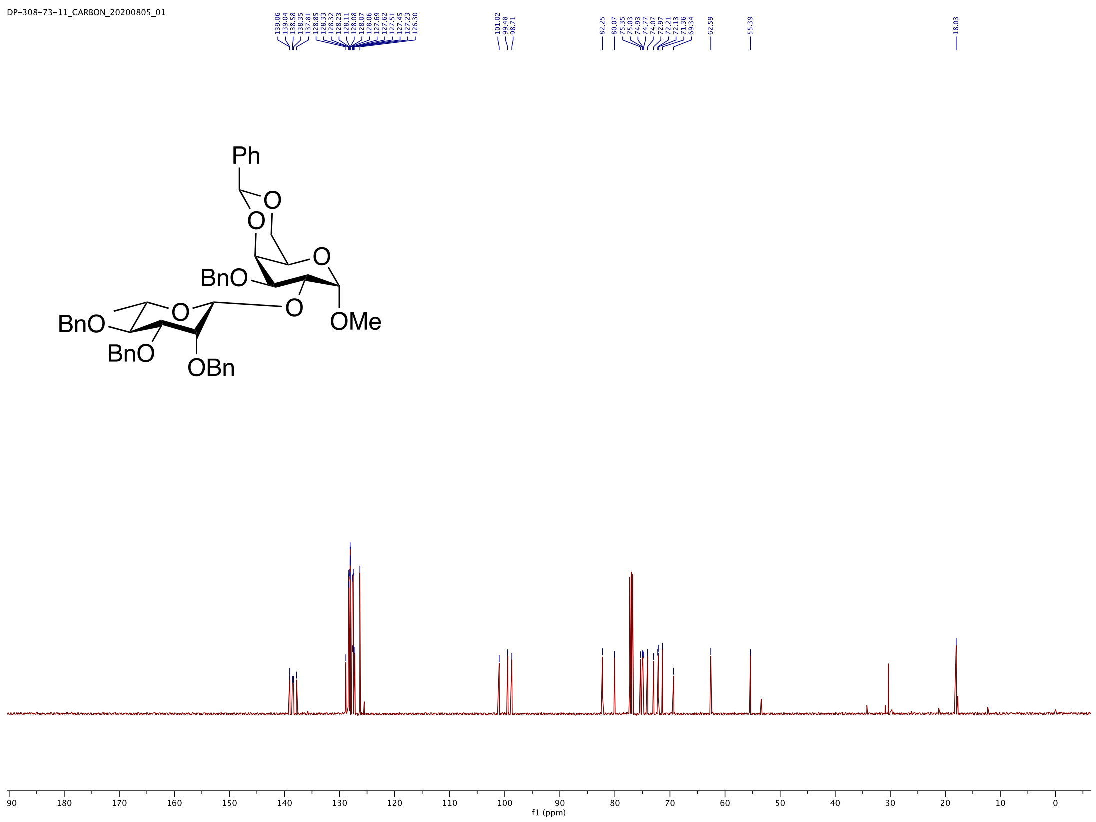

^{13}C - ^{1}H decoupled HSQC (500 x 126 MHz, Chloroform-*d*) 5j

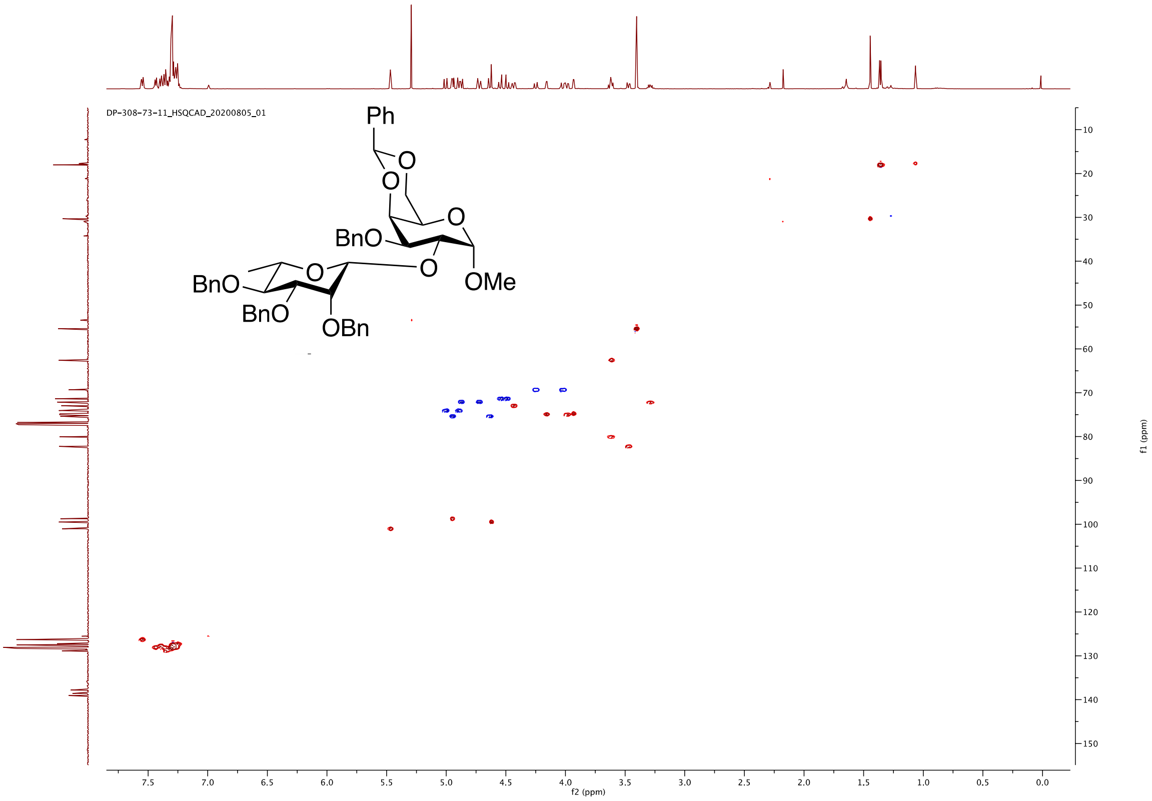

^{13}C - ^{1}H coupled HSQC (500 x 126 MHz, Chloroform-*d*) 5j

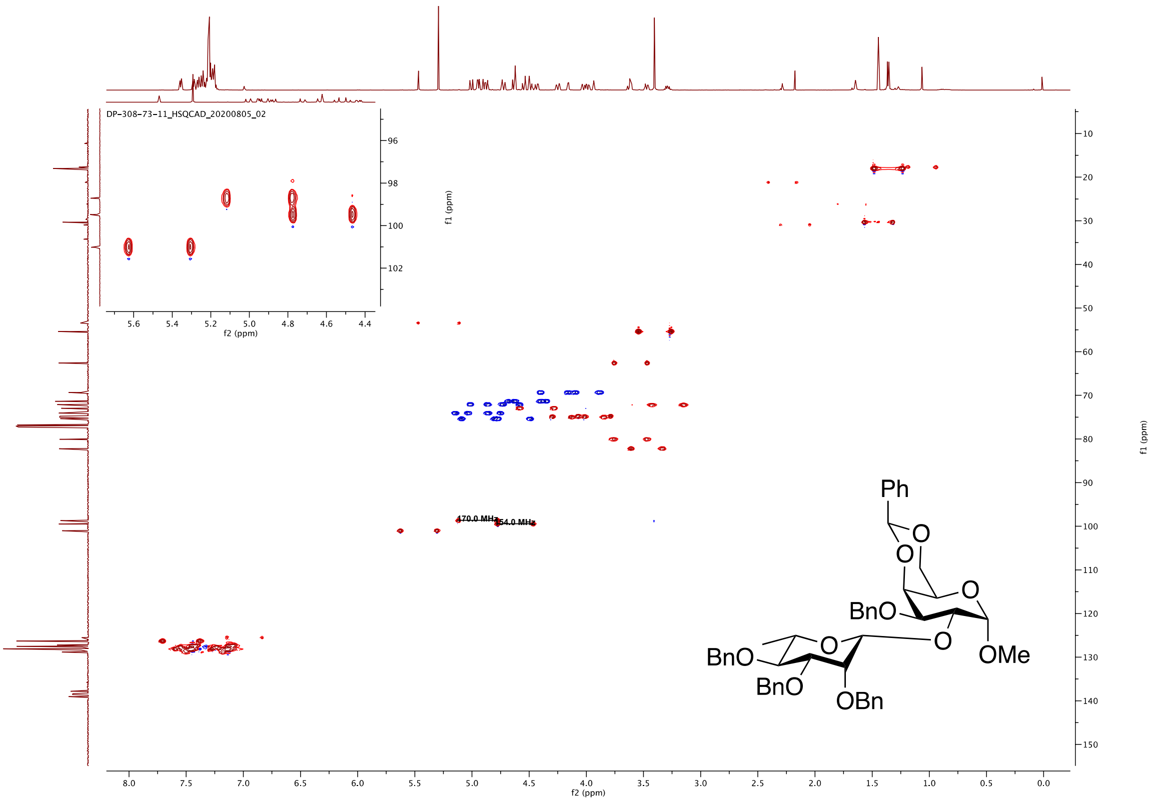

COSY (500 x 500 MHz, Chloroform-*d*) 5j

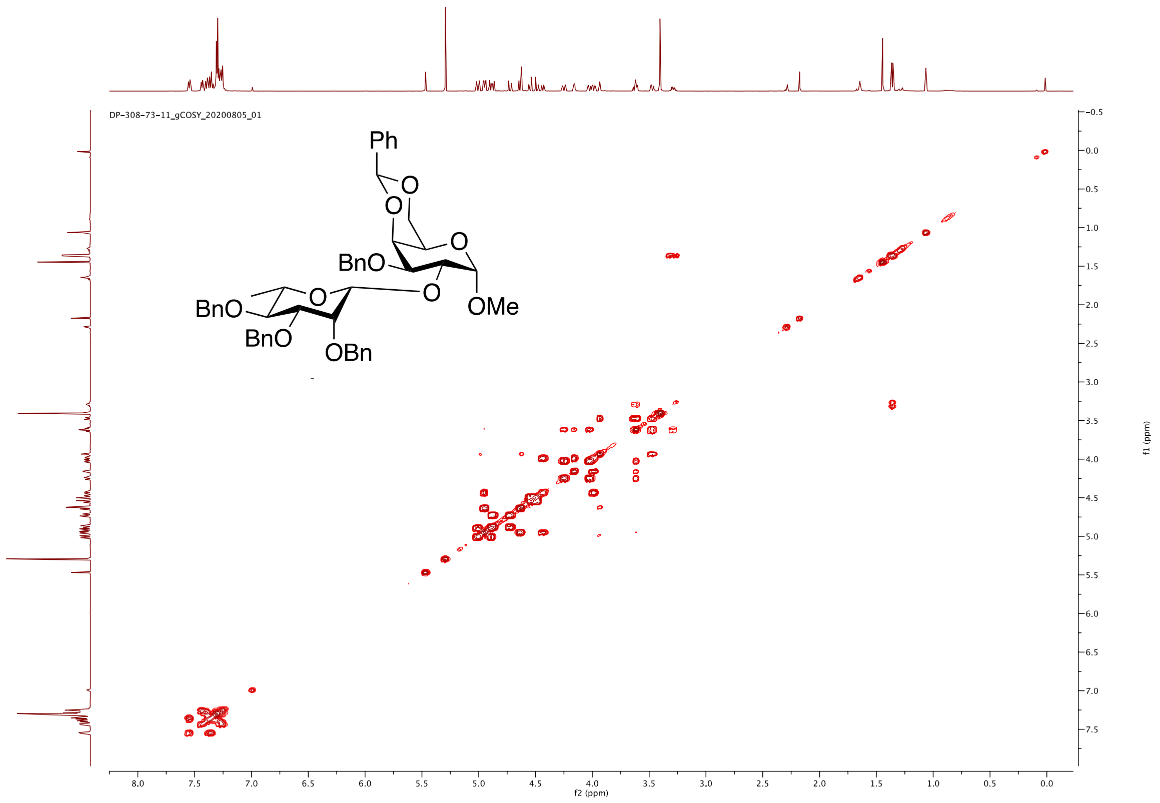

HMBC (500 x 126 MHz, Chloroform-*d*) 5j

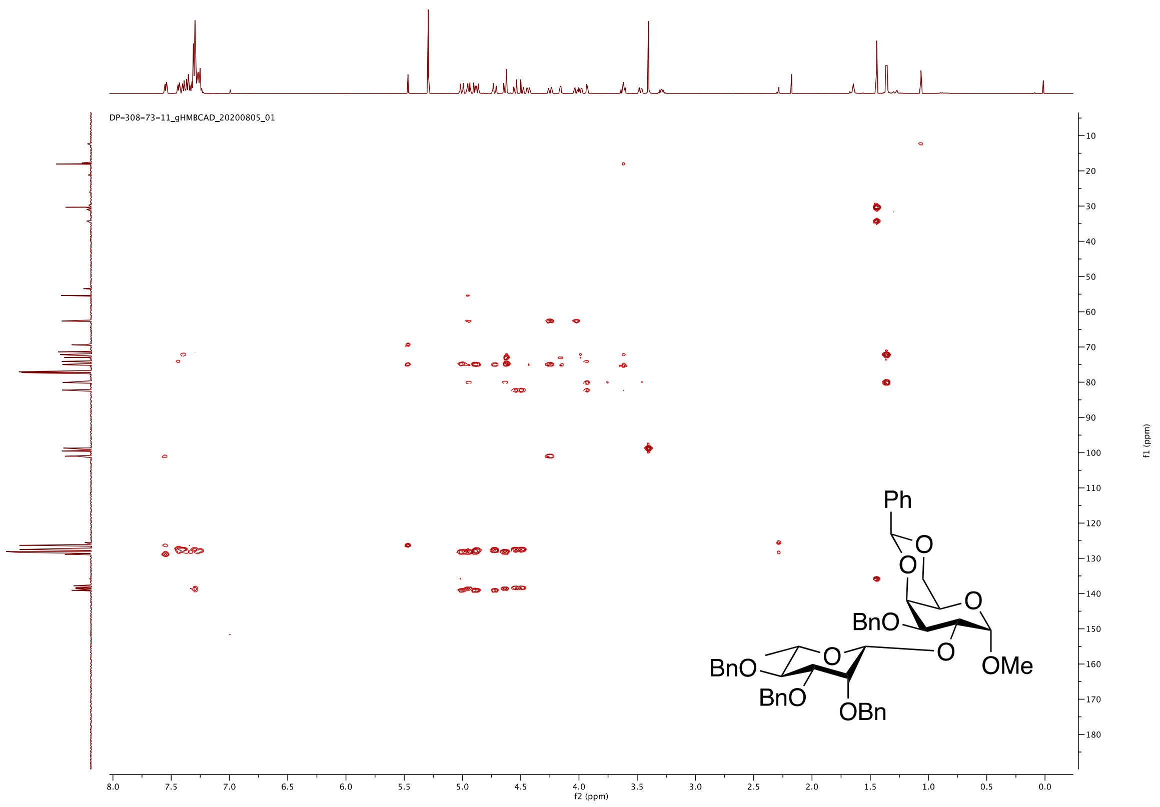

^{1}H NMR (500 MHz, Chloroform-*d*) 5i

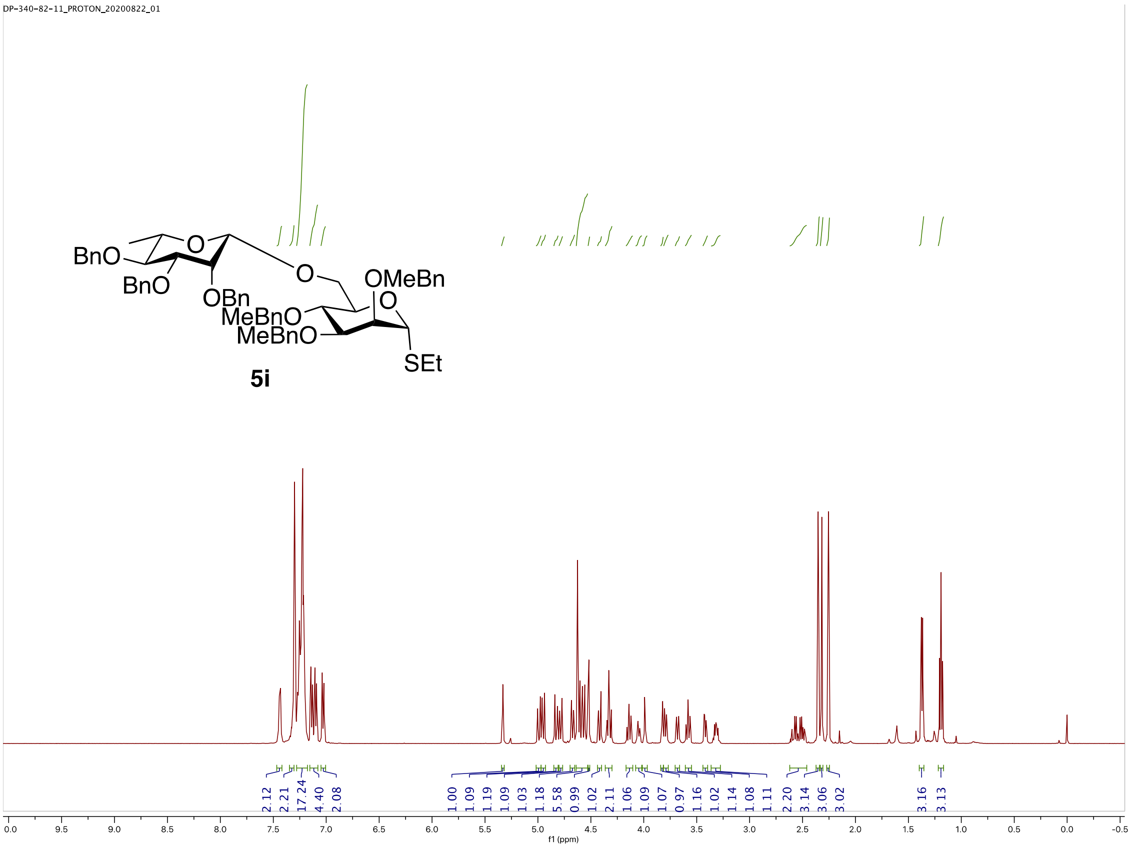

^{13}C NMR (126 MHz, Chloroform-*d*) 5i

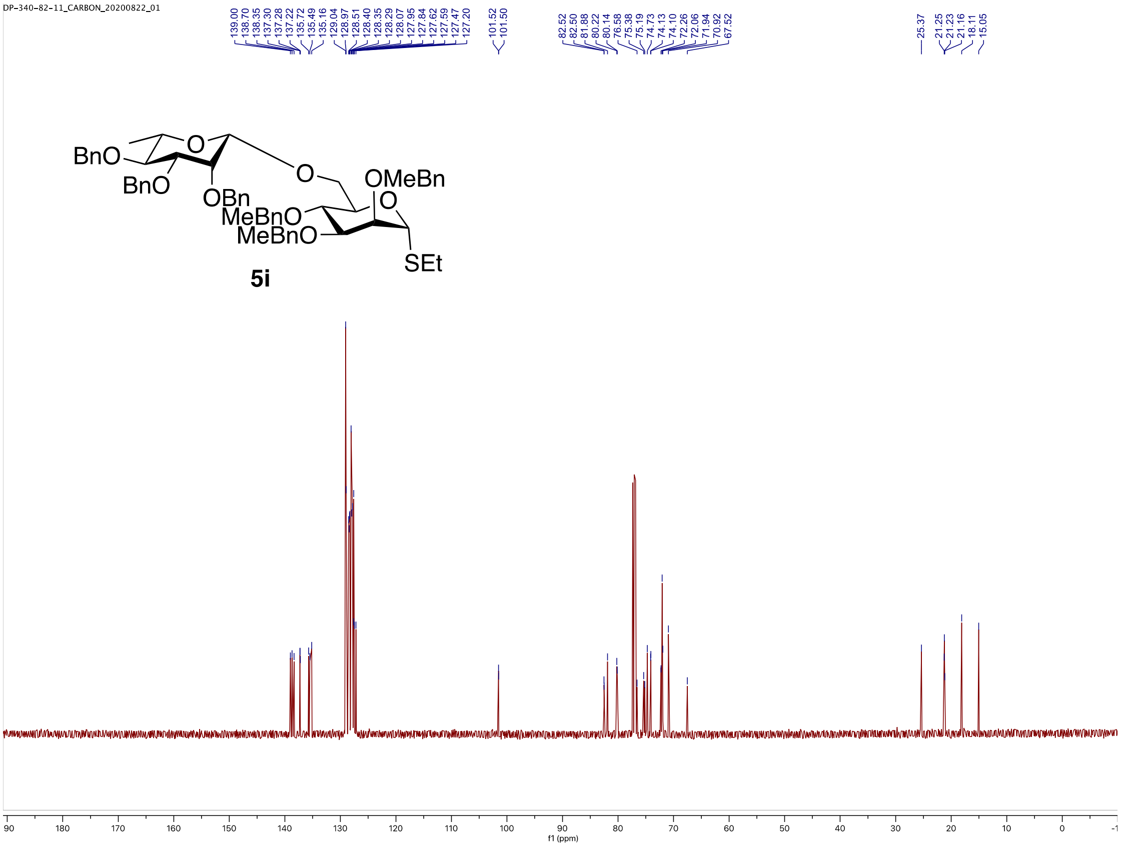

^{13}C - ^{1}H decoupled HSQC (500 x 126 MHz, Chloroform-*d*) 5i

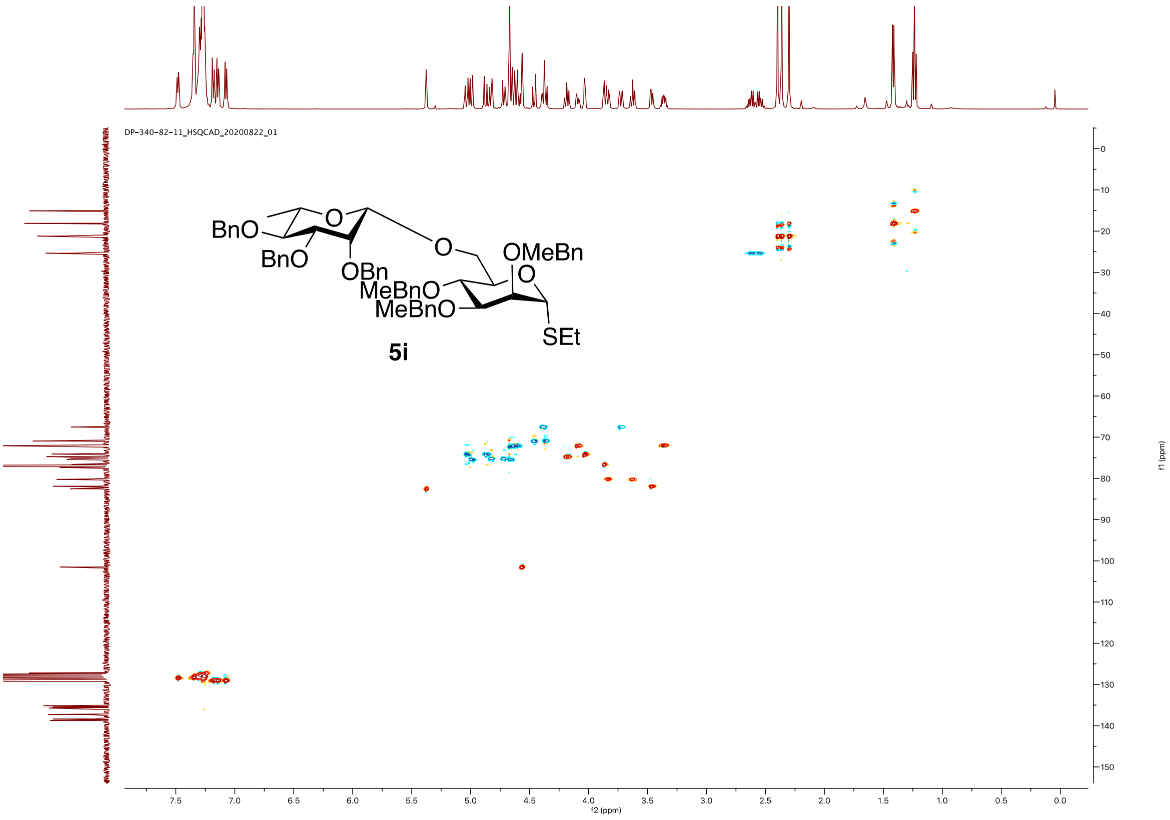

^{13}C - ^{1}H coupled HSQC (500 x 126 MHz, Chloroform-*d*) 5i

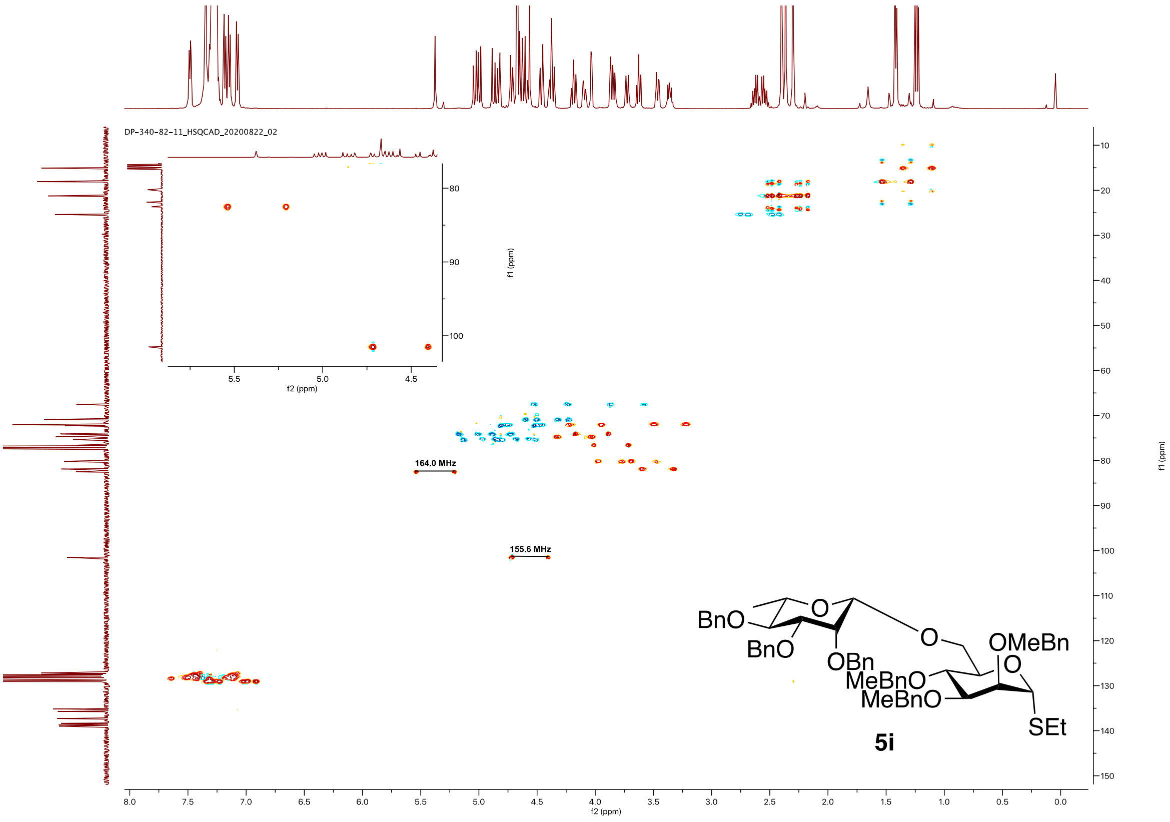

COSY (500 x 500 MHz, Chloroform-*d*) 5i

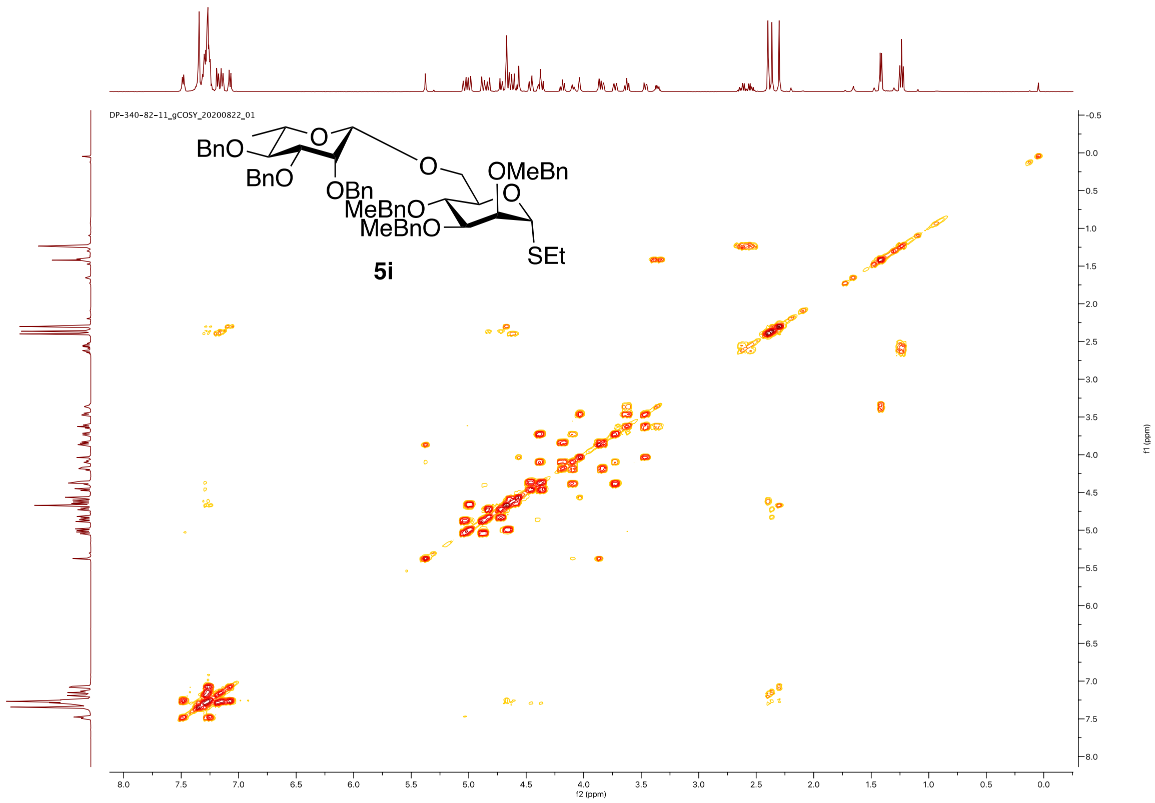

HMBC (500 x 126 MHz, Chloroform-*d*) 5i

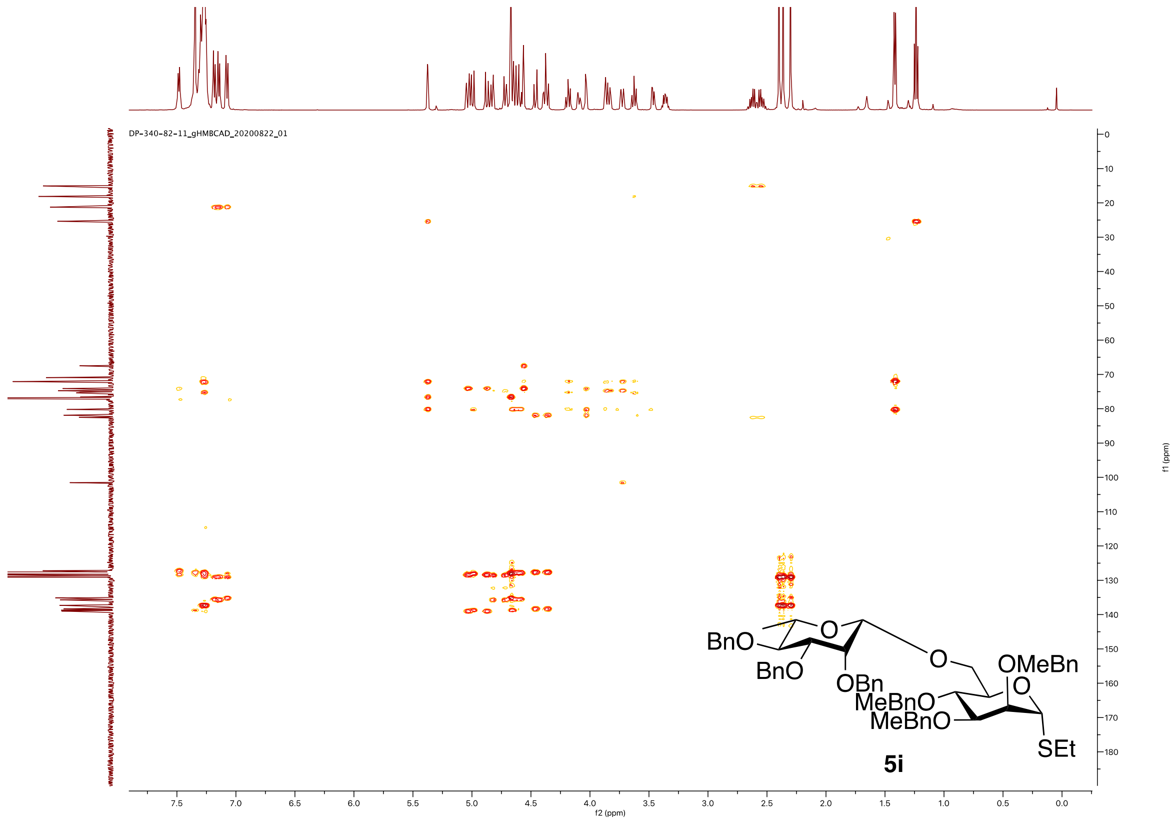

^{1}H NMR (500 MHz, Chloroform-*d*) 5h

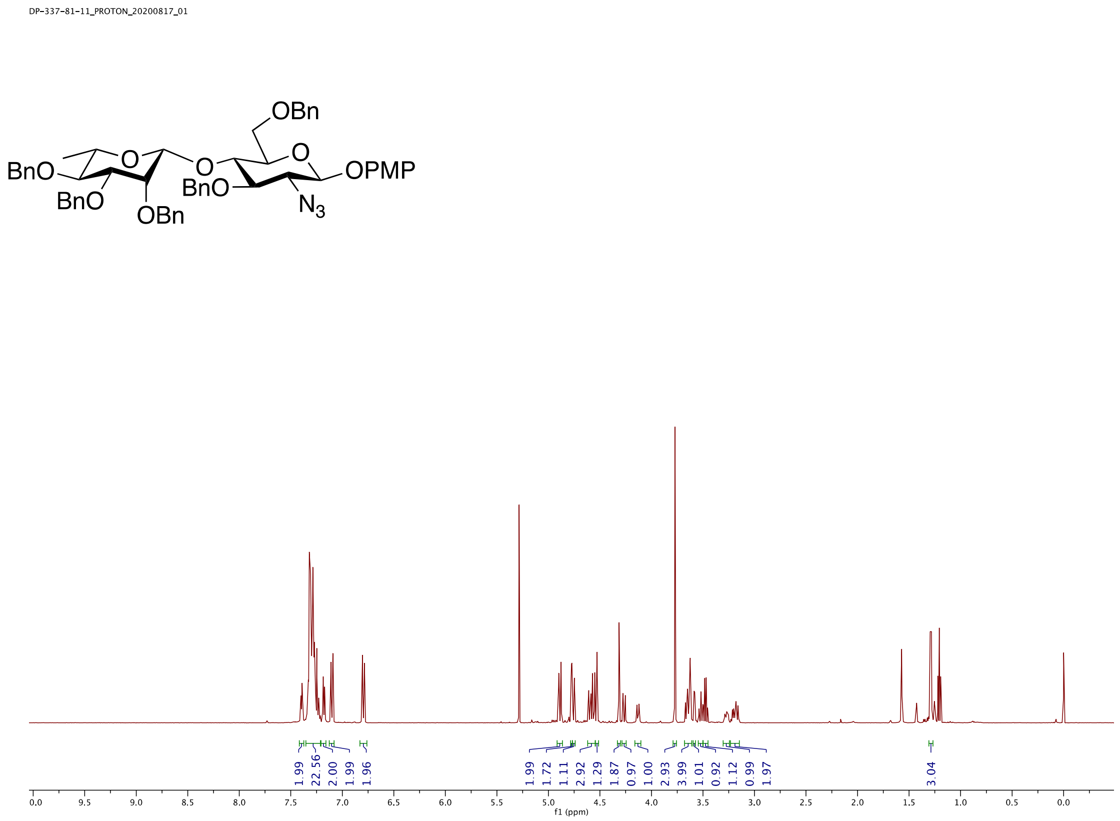

^{13}C NMR (126 MHz, Chloroform-*d*) 5h

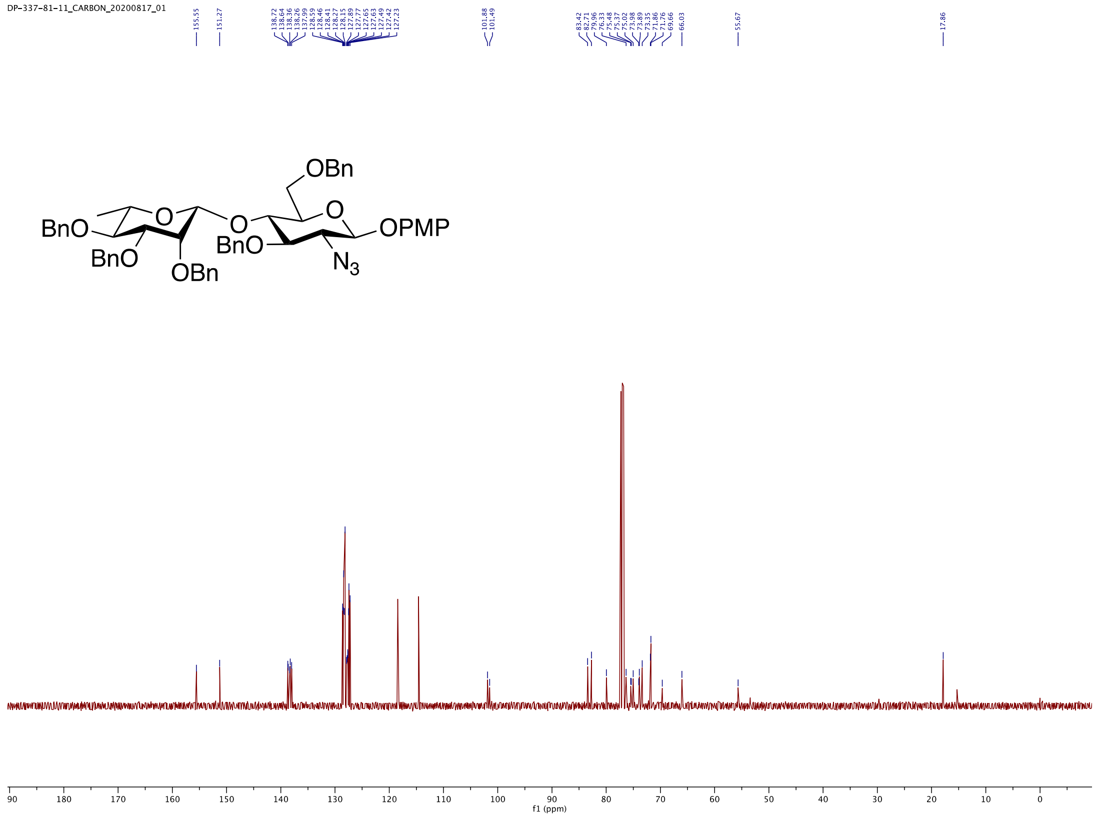

^{13}C - ^{1}H decoupled HSQC (500 x 126 MHz, Chloroform-*d*) 5h

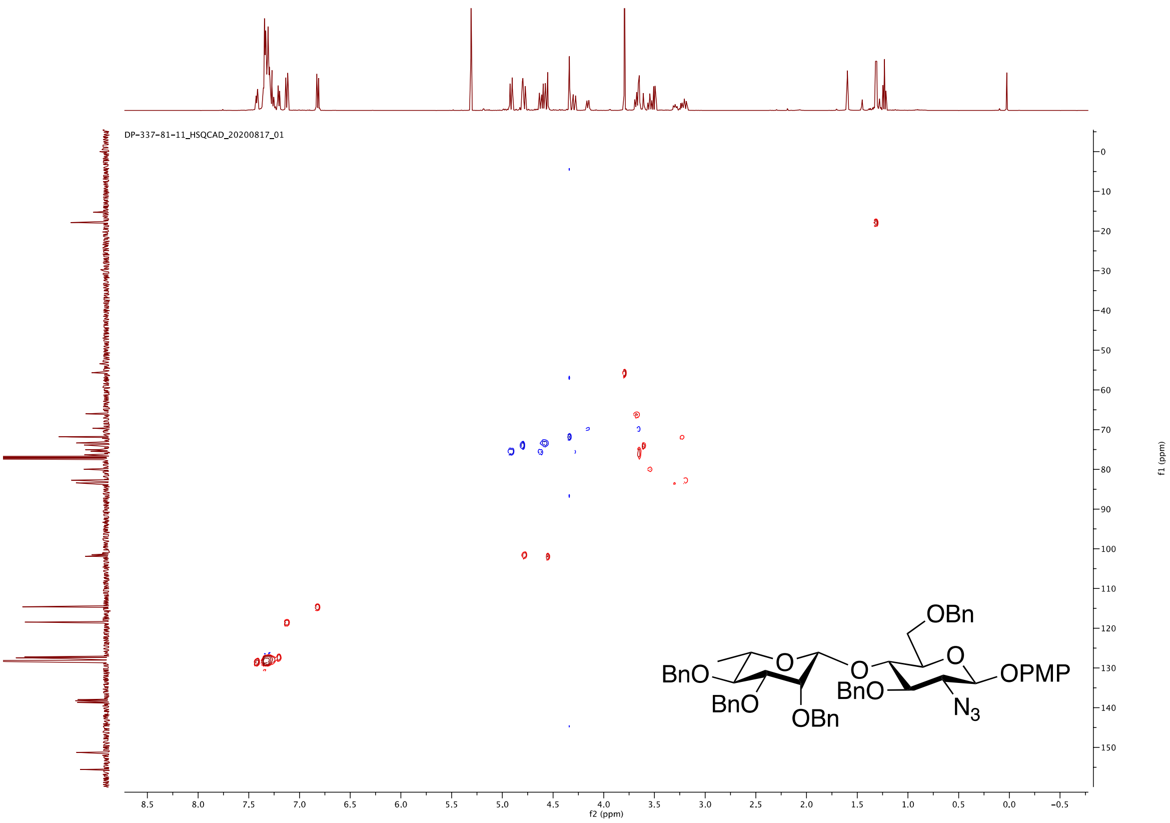

^{13}C - ^{1}H coupled HSQC (500 x 126 MHz, Chloroform-*d*) 5h

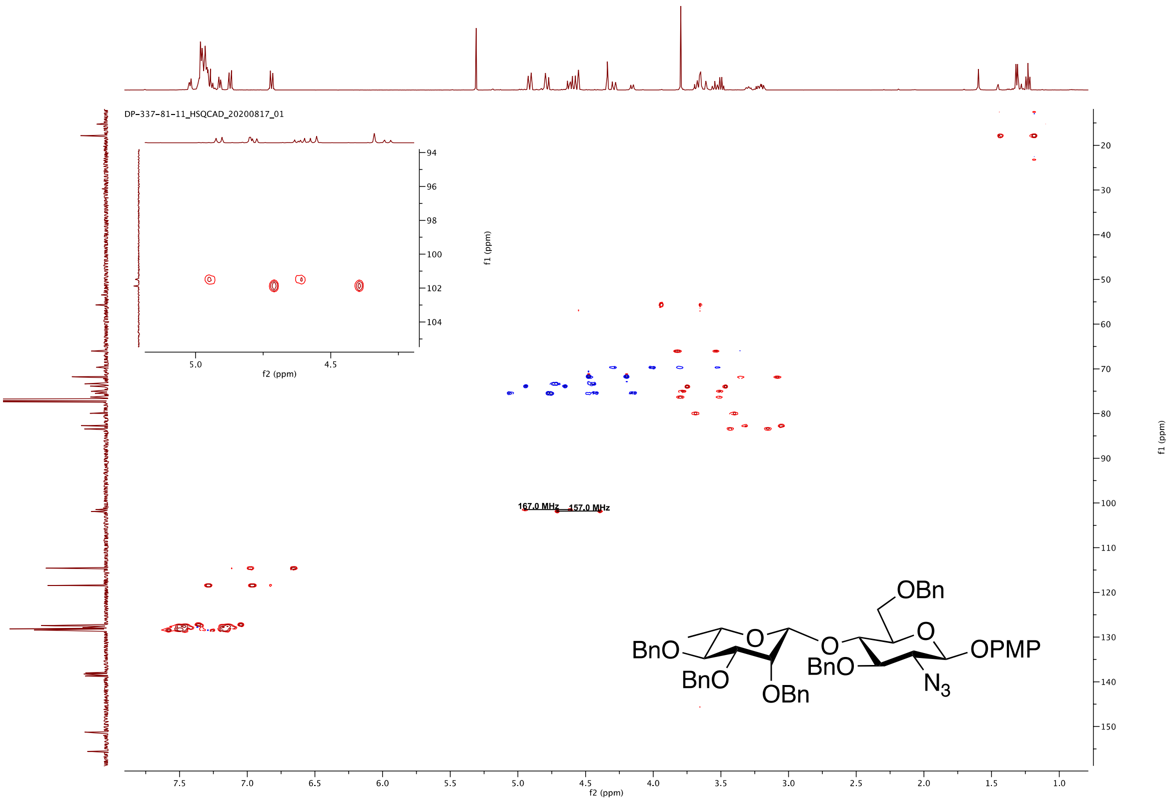

COSY (500 x 500 MHz, Chloroform-*d*) 5h

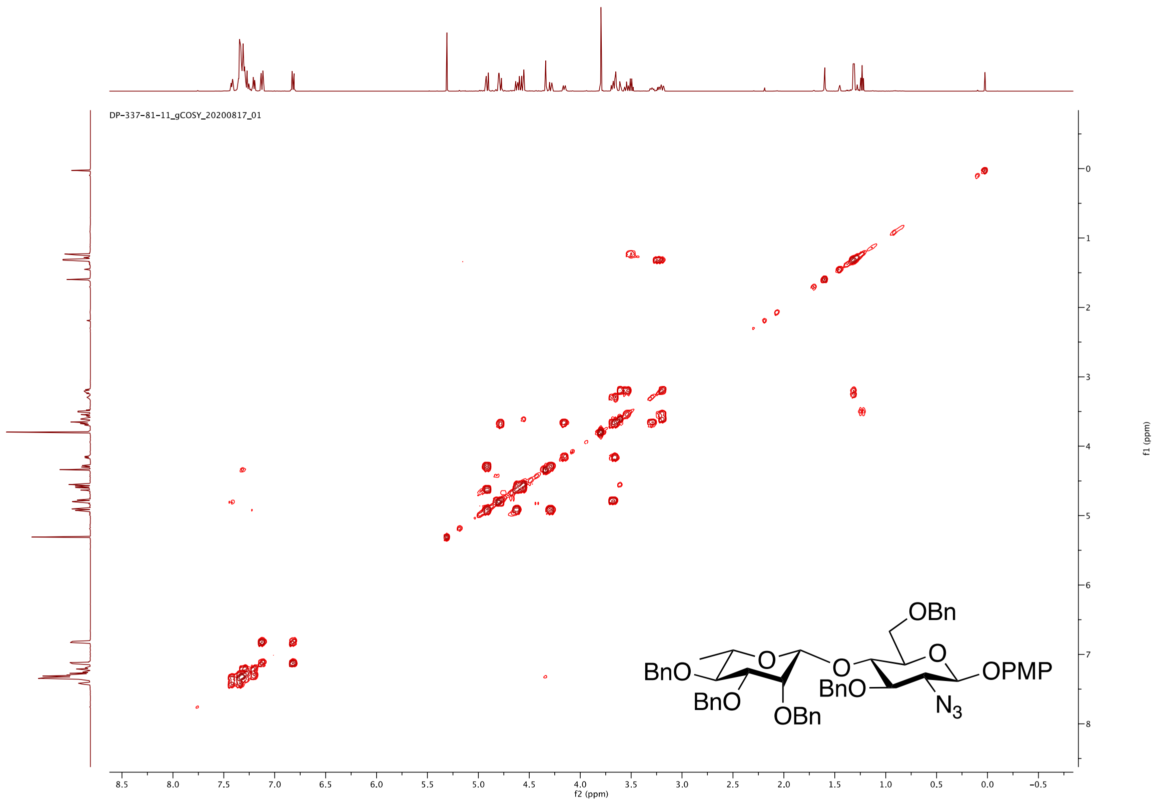

HMBC (500 x 126 MHz, Chloroform-*d*) 5h

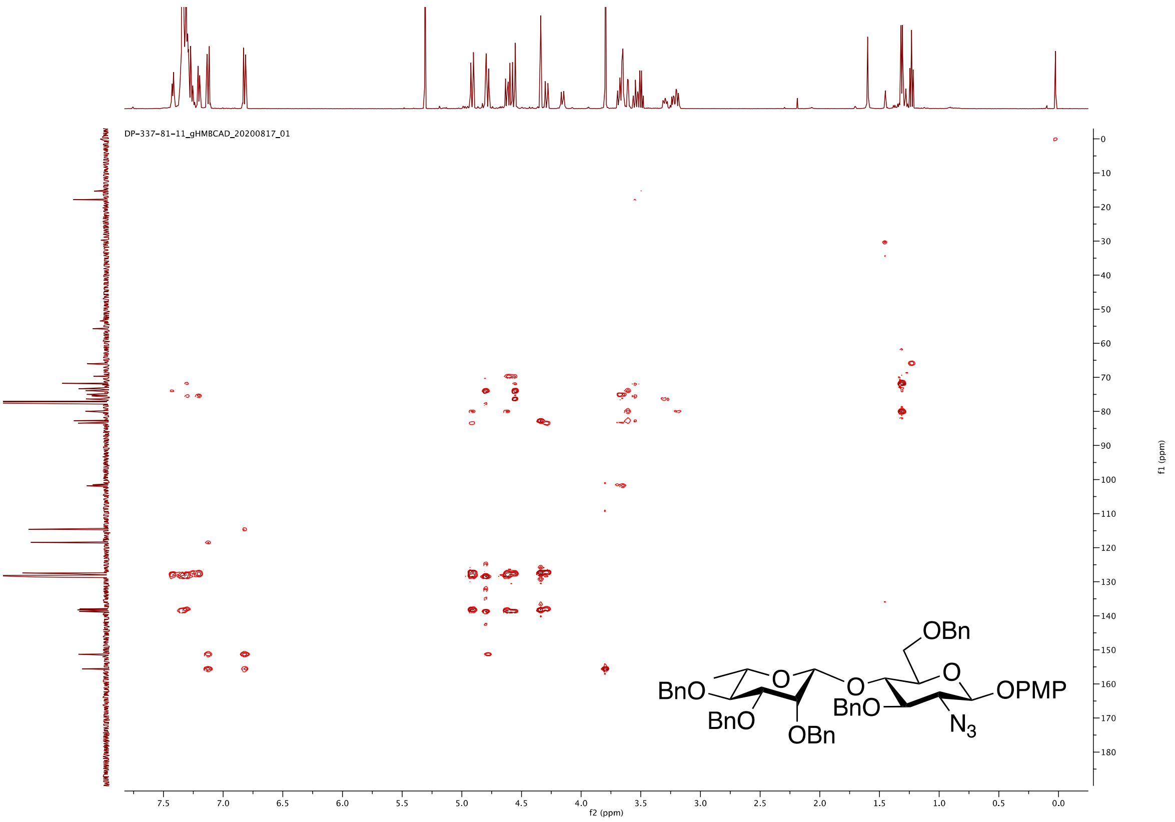

^{1}H NMR (500 MHz, Chloroform-*d*) 5k

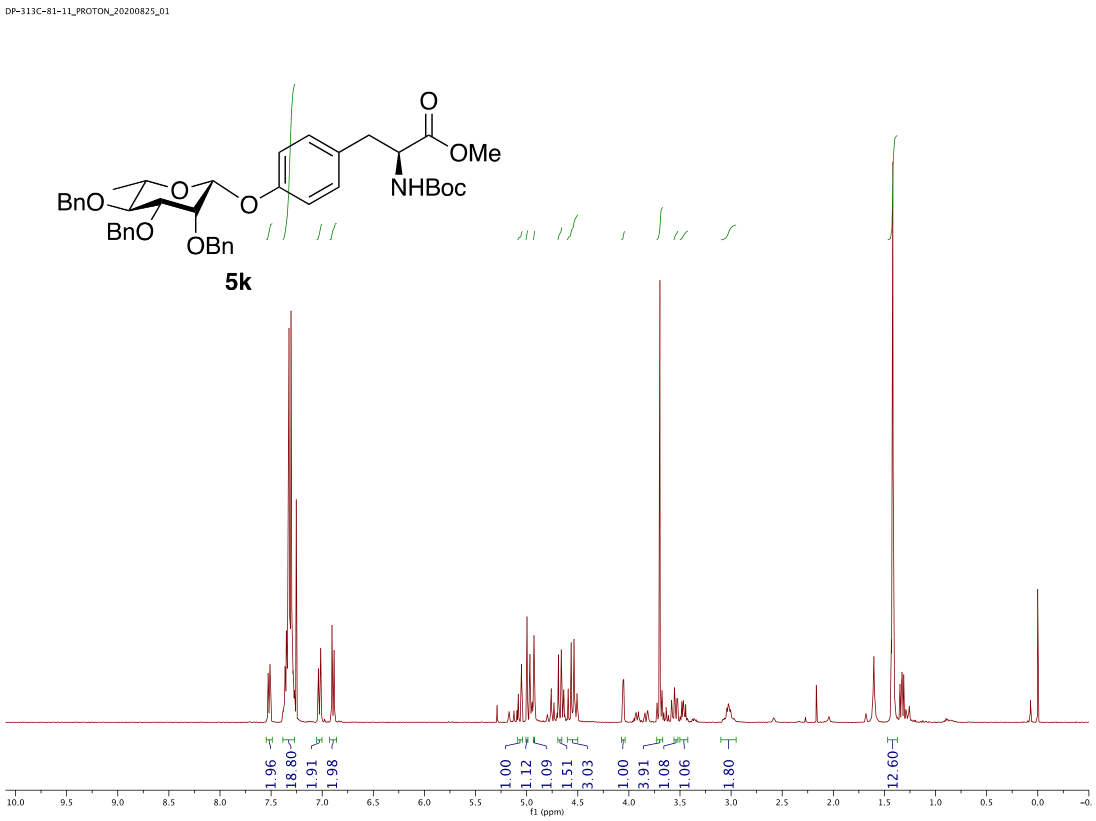

^{13}C (500 MHz, Chloroform-*d*) 5k

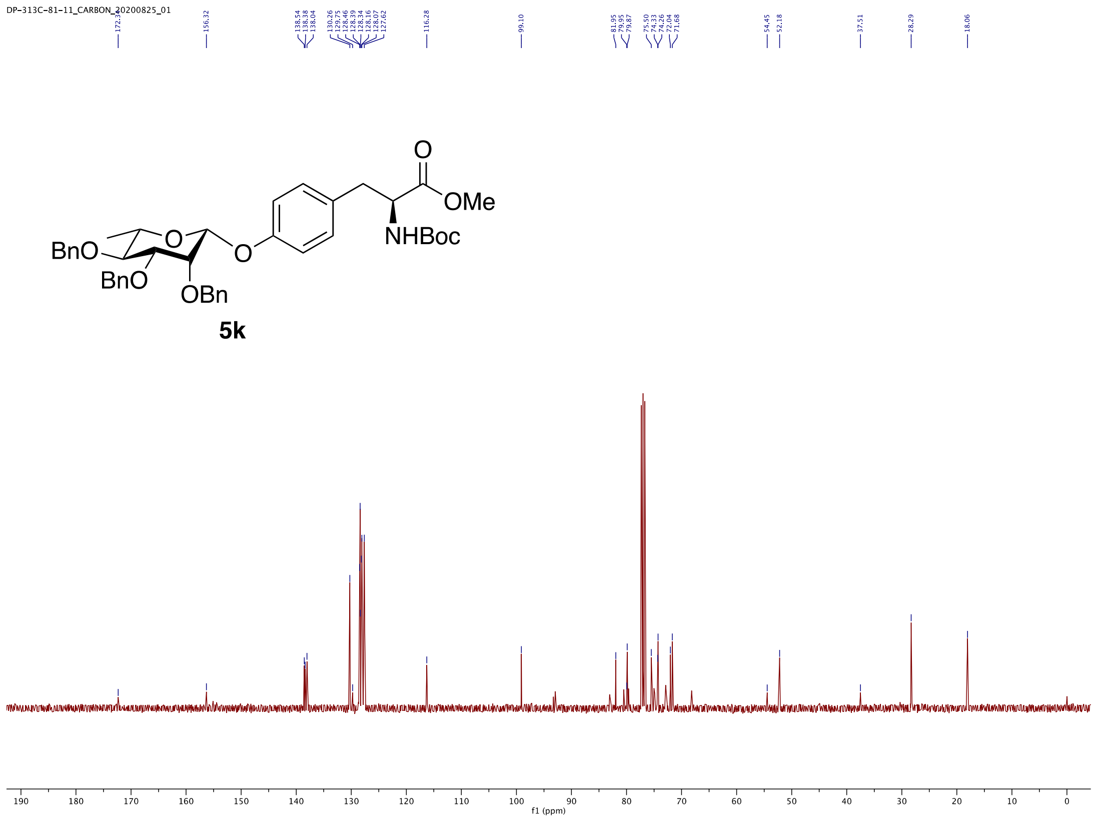

^{13}C - ^{1}H decoupled HSQC (500 x 126 MHz, Chloroform-*d*) 5k

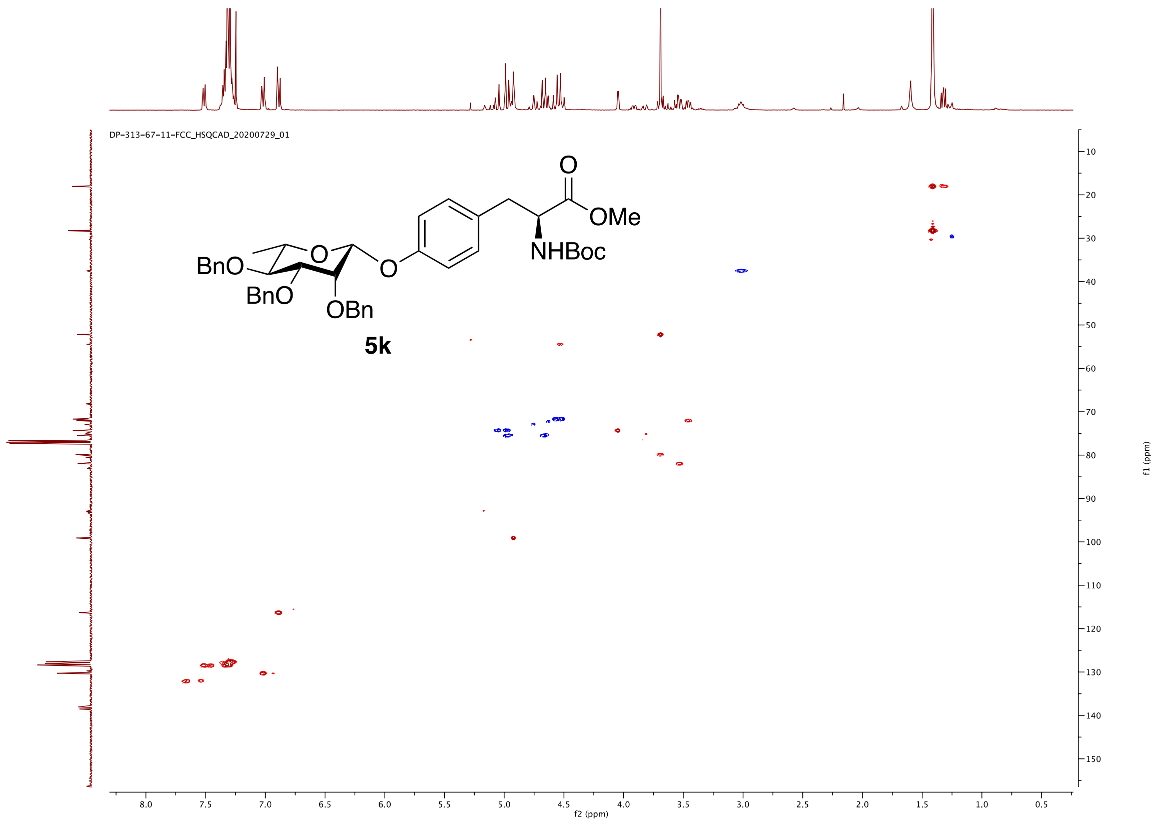

^{13}C - ^{1}H coupled HSQC (500 x 126 MHz, Chloroform-*d*) 5k

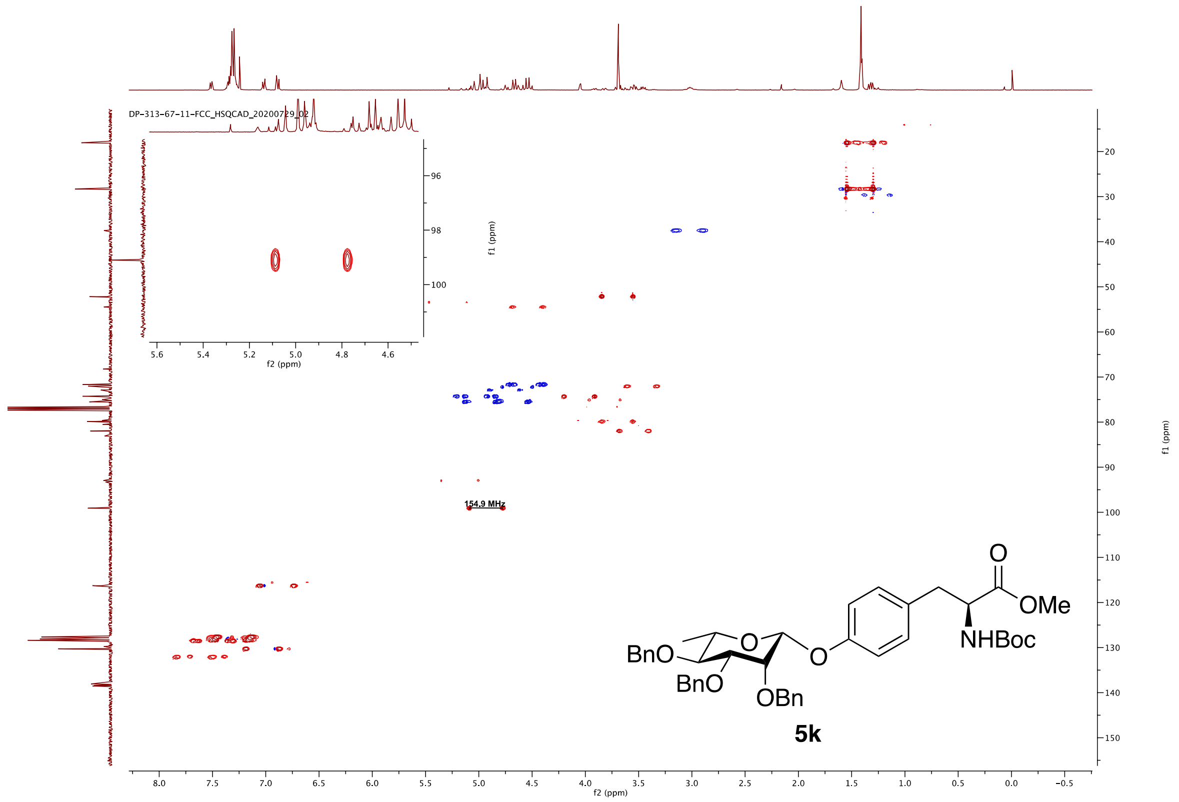

COSY (500 x 500 MHz, Chloroform-*d*) 5k

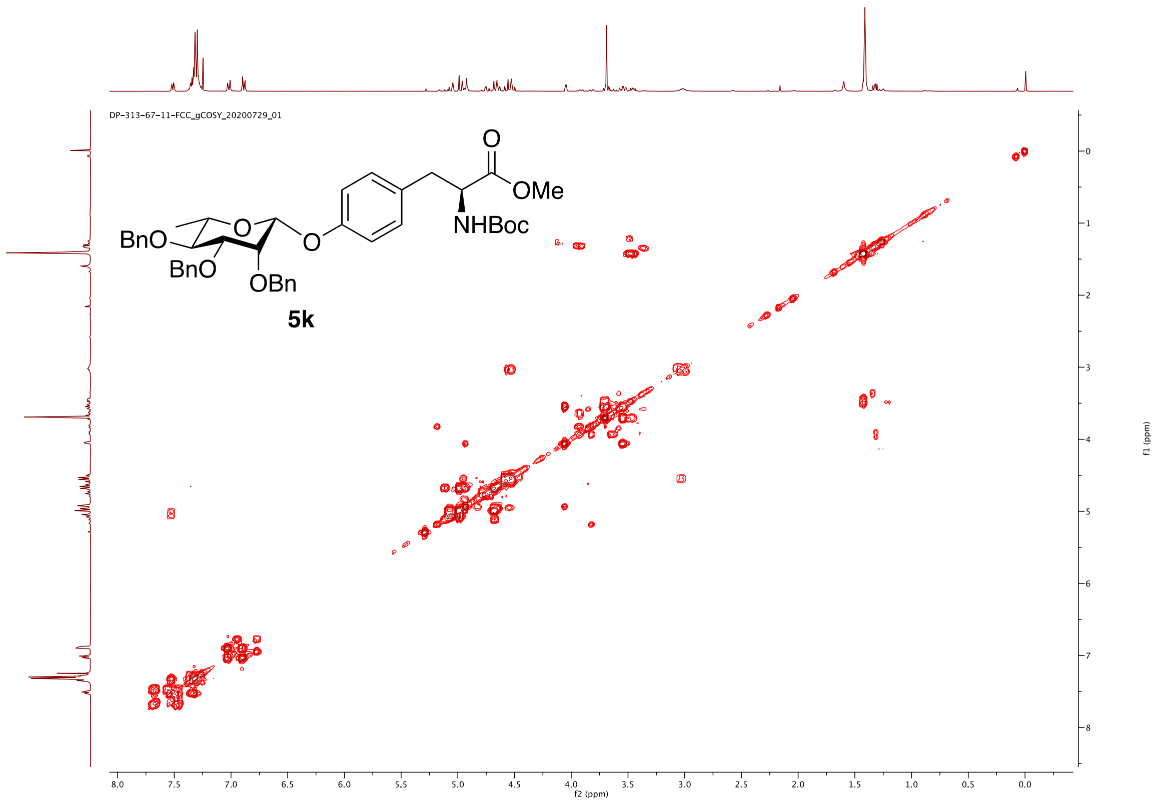

HMBC (500 x 126 MHz, Chloroform-*d*) 5k

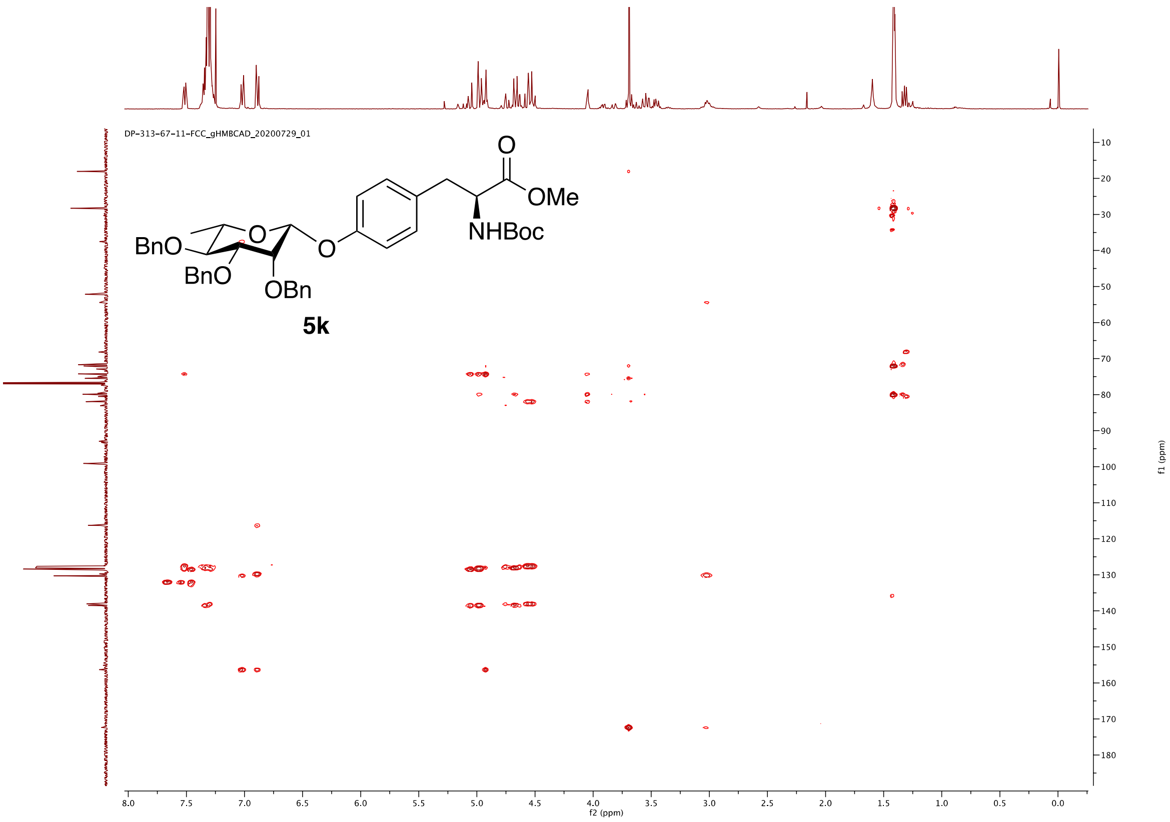

^{1}H NMR (500 MHz, Chloroform-*d*) S50

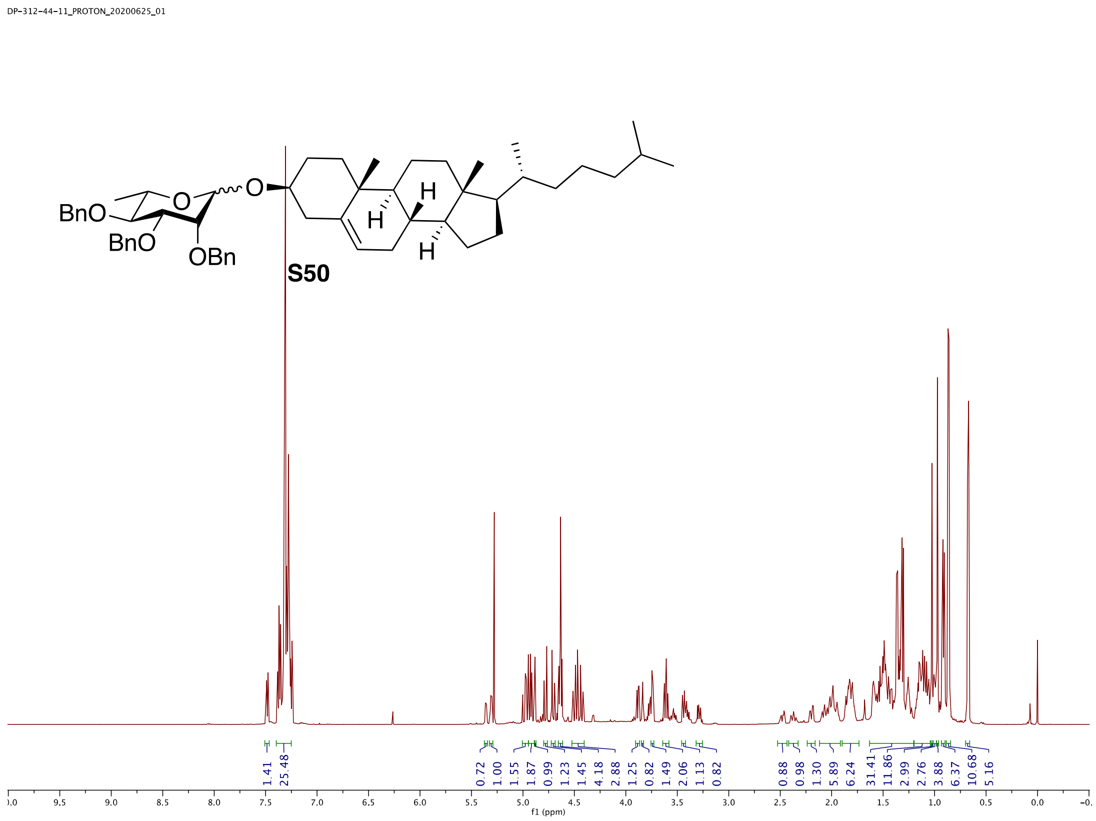

^{13}C NMR (101 MHz, Chloroform-*d*) S50

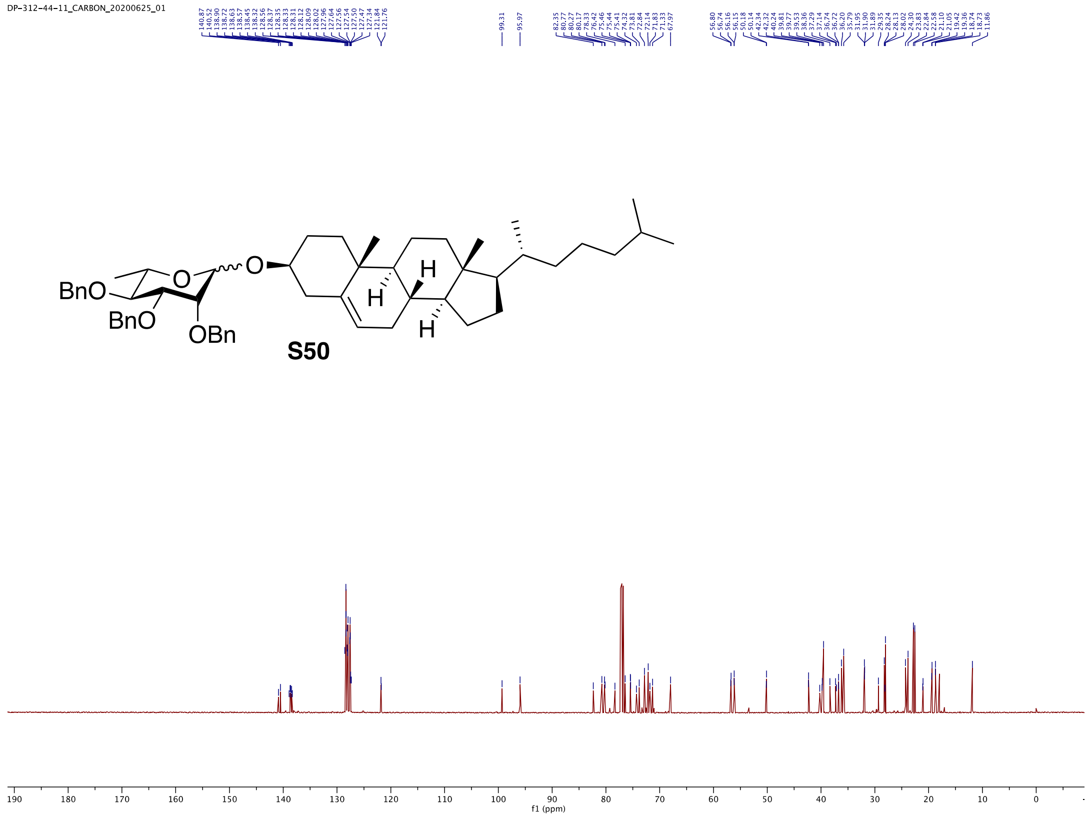

^{13}C - ^{1}H decoupled HSQC (500 x 126 MHz, Chloroform-*d*) S50

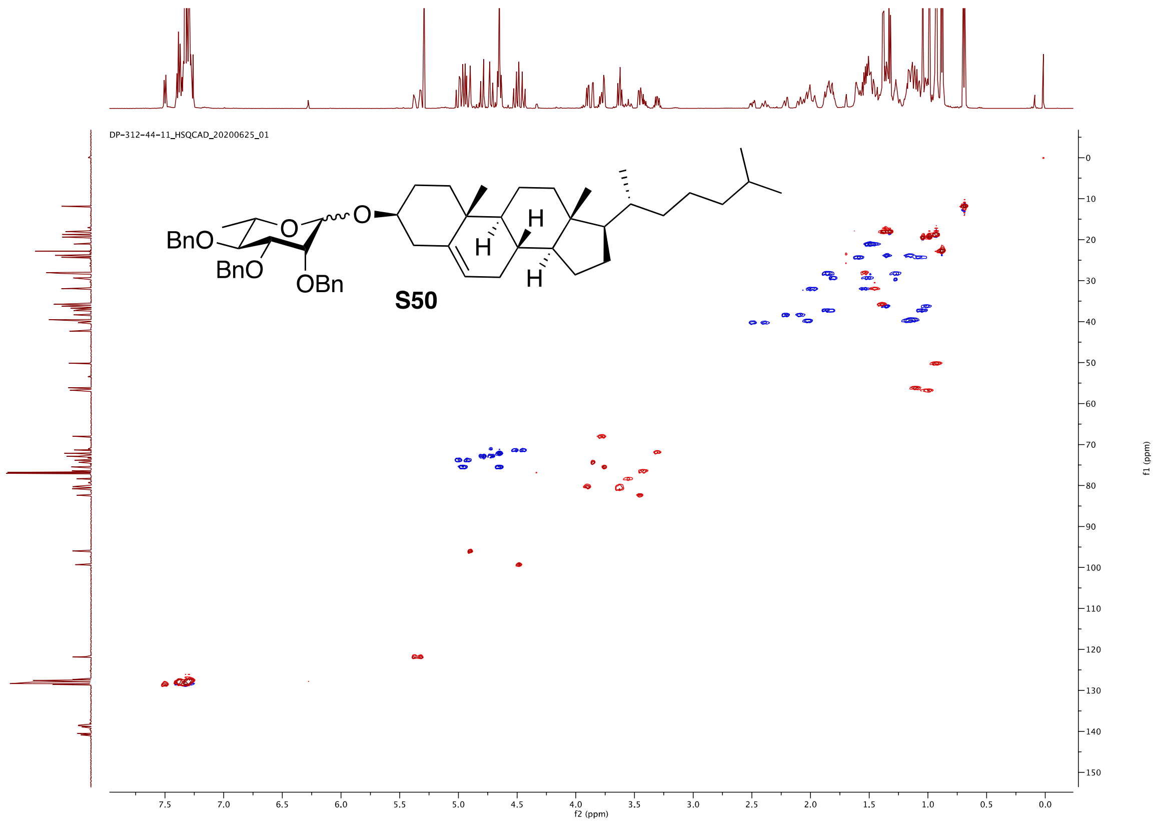

^{13}C - ^{1}H coupled HSQC (500 x 126 MHz, Chloroform-*d*) S50

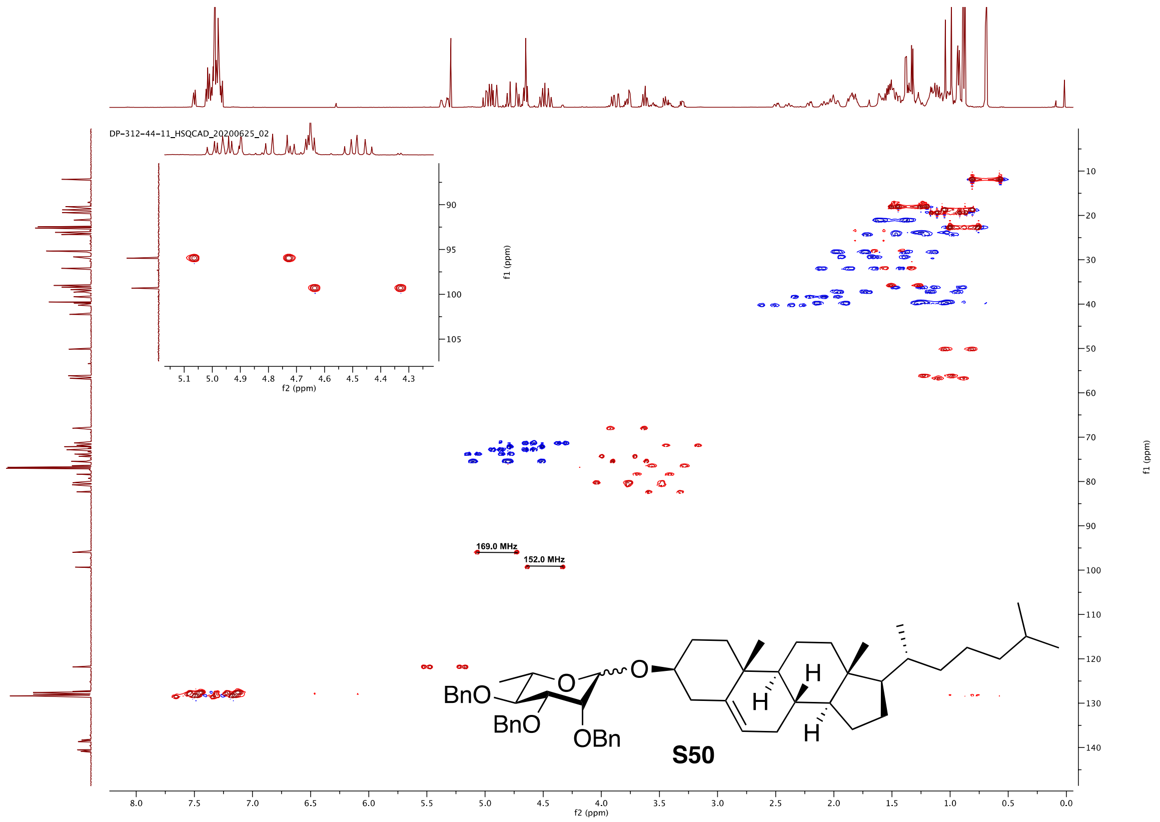

COSY (500 x 500 MHz, Chloroform-*d*) S50

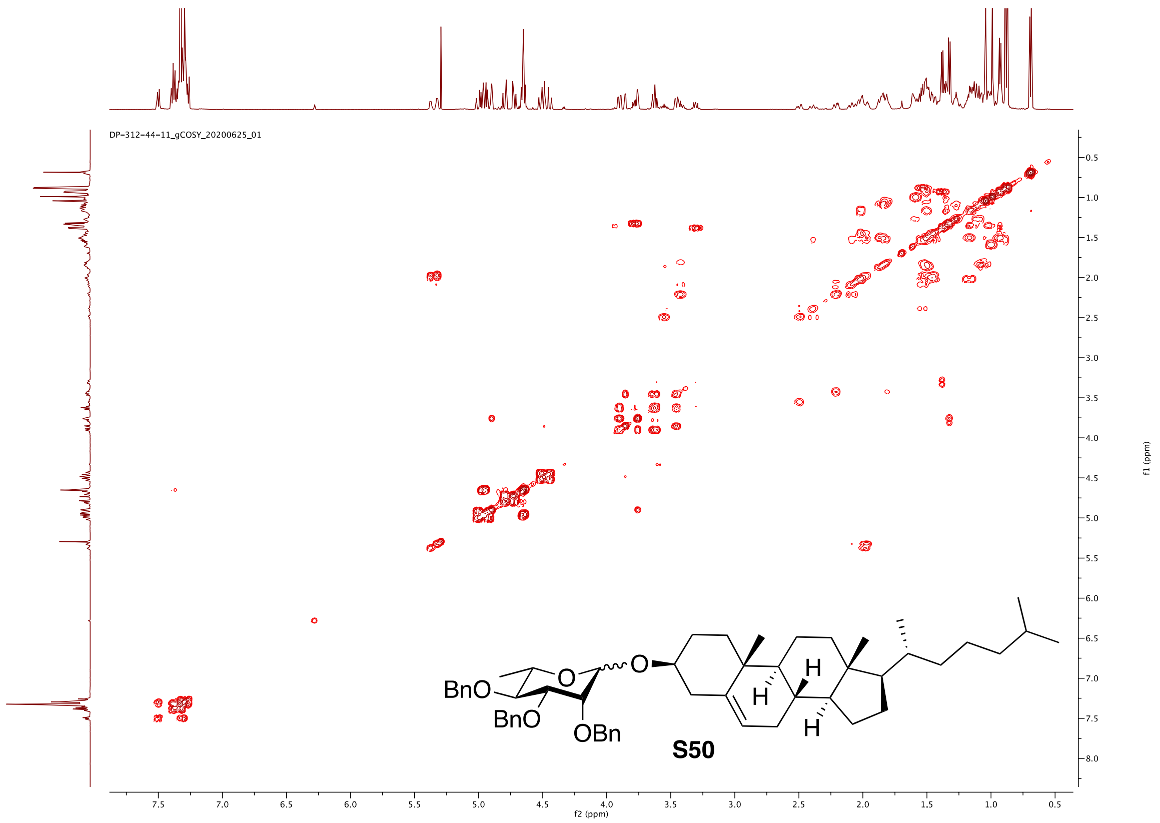

HMBC (500 x 126 MHz, Chloroform-*d*) S50

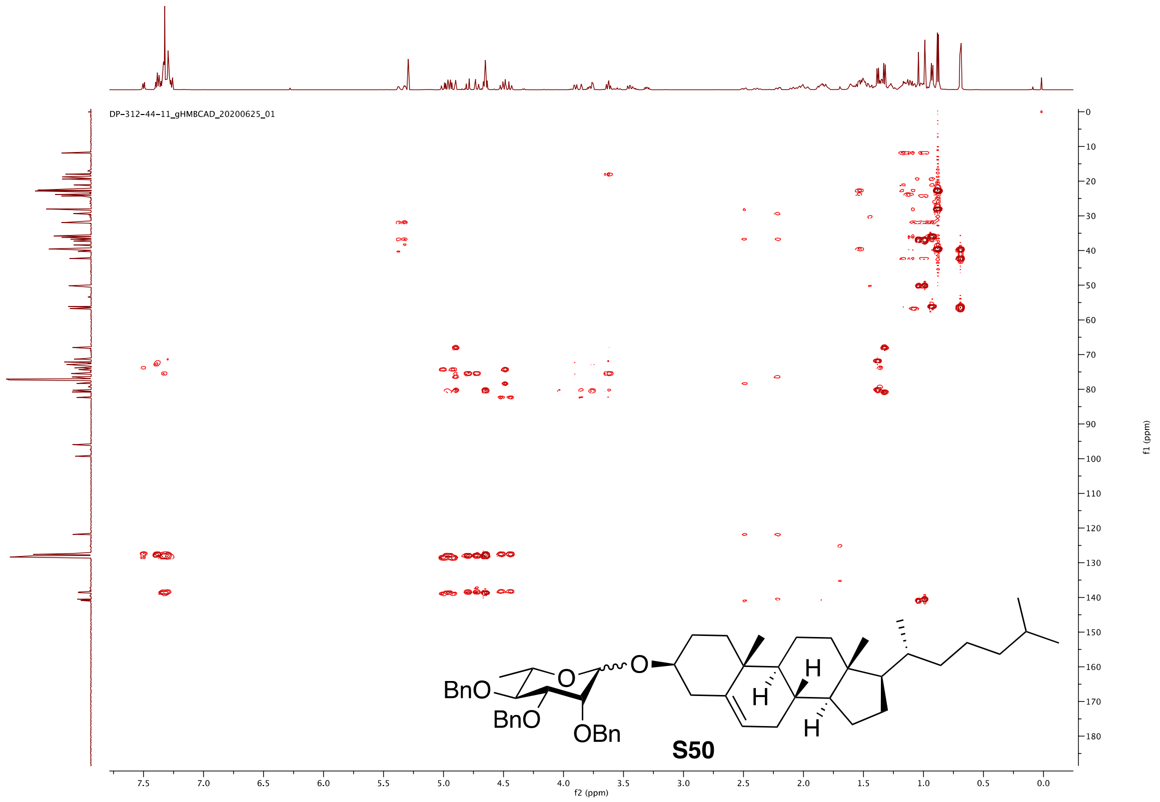

Supplement: SC-012-D1SC01300A-s002 [file SC-012-D1SC01300A-s002.pdf]
